# Genomic Insights Into the Evolution and Demographic History of the SARS-CoV-2 Omicron Variant: Population Genomics Approach

**DOI:** 10.2196/40673

**Published:** 2023-06-12

**Authors:** Kritika M Garg, Vinita Lamba, Balaji Chattopadhyay

**Affiliations:** 1 Department of Biology Ashoka University Sonipat India; 2 Centre for Interdisciplinary Archaeological Research Ashoka University Sonipat India; 3 Trivedi School of Biosciences Ashoka University Sonipat India; 4 J William Fulbright College of Arts and Sciences Department of Biological Sciences University of Arkansas Fayetteville, AR United States

**Keywords:** SARS-CoV-2, Omicron, evolutionary network, population subdivision, genome evolution, COVID-19, microevolution

## Abstract

**Background:**

A thorough understanding of the patterns of genetic subdivision in a pathogen can provide crucial information that is necessary to prevent disease spread. For SARS-CoV-2, the availability of millions of genomes makes this task analytically challenging, and traditional methods for understanding genetic subdivision often fail.

**Objective:**

The aim of our study was to use population genomics methods to identify the subtle subdivisions and demographic history of the Omicron variant, in addition to those captured by the Pango lineage.

**Methods:**

We used a combination of an evolutionary network approach and multivariate statistical protocols to understand the subdivision and spread of the Omicron variant. We identified subdivisions within the BA.1 and BA.2 lineages and further identified the mutations associated with each cluster. We further characterized the overall genomic diversity of the Omicron variant and assessed the selection pressure for each of the genetic clusters identified.

**Results:**

We observed concordant results, using two different methods to understand genetic subdivision. The overall pattern of subdivision in the Omicron variant was in broad agreement with the Pango lineage definition. Further, 1 cluster of the BA.1 lineage and 3 clusters of the BA.2 lineage revealed statistically significant signatures of selection or demographic expansion (Tajima’s D<−2), suggesting the role of microevolutionary processes in the spread of the virus.

**Conclusions:**

We provide an easy framework for assessing the genetic structure and demographic history of SARS-CoV-2, which can be particularly useful for understanding the local history of the virus. We identified important mutations that are advantageous to some lineages of Omicron and aid in the transmission of the virus. This is crucial information for policy makers, as preventive measures can be designed to mitigate further spread based on a holistic understanding of the variability of the virus and the evolutionary processes aiding its spread.

## Introduction

**Figure 1:**
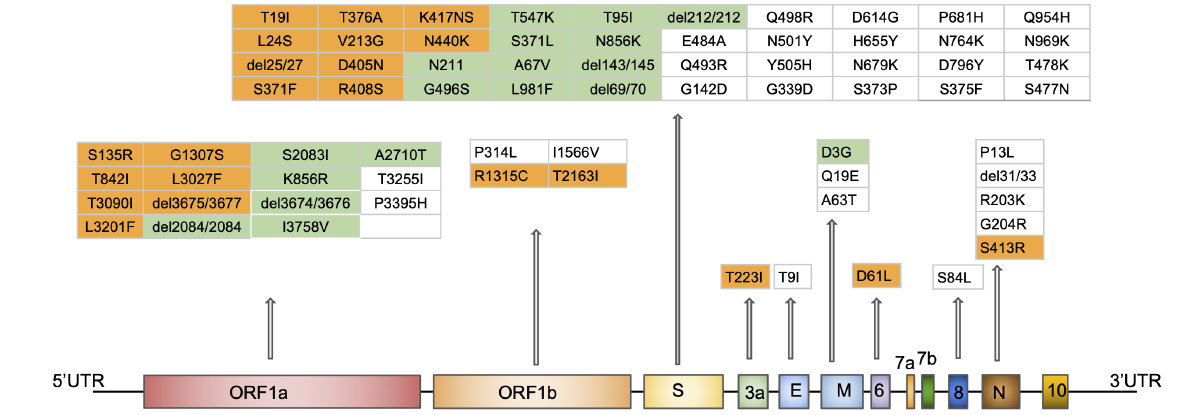
The genome structure of SARS-CoV-2 with known mutations in the Omicron variant highlighted. Mutations unique to the BA.1 lineage are highlighted in green, and those unique to the BA.2 lineage are highlighted in orange. Mutations common to both lineages of Omicron are in plain black font. The list of mutations was obtained from Tzou et al [12]. ORF: open reading frame; UTR: untranslated region.

The past 2 decades have witnessed multiple zoonotic coronavirus outbreaks, with the latest being the COVID-19 outbreak, which was caused by SARS-CoV-2. The virus emerged in Wuhan, China, and it quickly spread across the globe, resulting in more than 6.5 million deaths [1-3]. SARS-CoV-2 is a *Betacoronavirus* with a positive, single-stranded RNA genome. The genome is approximately 30 kilobases in length and encodes for 26 proteins (16 nonstructural, 4 structural, and 6 accessory proteins; Figure 1) [4,5].

Extensive genomic surveillance programs were established across the globe to monitor the evolution of the virus. This resulted in an exponential increase in the number of SARS-CoV-2 genomes that has in turn presented a unique set of challenges for data analysis [6-9]. With over 10 million genome sequences already available, new algorithms are being designed to tackle the deluge of data [6-9]. Most available analytical tools are designed to identify the overall evolutionary relationship between various lineages. However, obtaining a finer-level understanding of the diversity and subdivision within a lineage can provide important insights into pathogen evolution, particularly during ongoing pandemics [6]. Pango lineage classification is one such nomenclature method for identifying fine-scale, phylogenetically relevant clusters of SARS-CoV-2 based on the mutations in the spike protein [6].

In this study, we used population genomics methods to understand the subdivision of the Omicron lineage of SARS-CoV-2 as it spread across the globe, in an attempt to elucidate the evolutionary history of the variant. The Omicron variant was first identified within Botswana, Southern Africa, in November 2021, and within a short span of time, it emerged as the main variant driving SARS-CoV-2 infections across the globe, replacing the Delta variant [10,11]. The Omicron variant was also of immediate concern due to the large number of mutations observed in its spike protein (Figure 1). Among the 60 mutations that this variant accumulated when compared to the reference Wuhan sequence, the majority were concentrated in the spike protein (38 mutations in the BA.1 lineage and 31 mutations in the BA.2 lineage; Figure 1) [12,13]. Some of these mutations increased both the transmission ability and the antibody escape of the virus, allowing the Omicron variant to rapidly spread across the globe [11,13]. Given the high infection rate and rapid spread of the virus across the globe, alternative methods for inspecting fine-scale subdivision and transmission are necessary to understand the evolution of the virus and devise any strategy to reduce its spread. Thus, we investigated the subdivision and demographic history of the BA.1 and BA.2 lineages of Omicron, along with identifying mutations that are correlated with the spread of the virus.

## Methods

### Data Matrix and Cleanup

We downloaded 20,067 SARS-CoV-2 genome sequences belonging to the Omicron lineage, which were available up to January 31, 2022, from the GISAID (Global Initiative on Sharing All Influenza Data) repository (Multimedia Appendices 1 and 2), retaining only high-coverage genomes (<1% undetermined nucleotide bases; <0.05% unique amino acid mutations) and genomes with a collection date for this study. Only sequences obtained from humans were used for all analyses. We retained 20,067 genomes, which were further filtered for quality by using Nextclade CLI (Nextstrain) [14]. Nextclade examines each query sequence for flaws that could suggest sequencing or assembly errors and assigns a score for each sequence. The quality score of a sequence is determined by the number of undetermined bases, ambiguous sites, private mutations, and stop codons. All sequences classified as good-quality sequences by Nextclade were selected for further analysis. We retained 14,002 good-quality sequences, of which most were from Denmark (n=11,272, 80.5%); the rest of the sequences were from 43 countries. We aligned the filtered SARS-CoV-2 genomes to the Wuhan reference genome (accession ID: MN908947.1) in Nextalign CLI [14], using default parameters. Further, we assigned the lineage for each sequence by using the pangolin web server (versions 3.1.20 and 4.0.6; accessed on March 3 and May 6, 2022, respectively) [6].

### Genetic Subdivision Analysis

We used two different approaches to understand the population subdivision within our SARS-CoV-2 data set. For the first approach, we reconstructed an evolutionary network by using the program VENAS (Viral Genome Evolution Network Analysis System) [15]. VENAS can analyze thousands of genomes in a short span of time (a few minutes) and is a useful tool for tracking changes across a transmission chain. It identifies mutations across alignments and constructs a network based on hamming distances. In VENAS, we first estimated the effective parsimony-informative sites and minor allele frequency, using default settings, and retained 5253 genomes. These were then used to construct an evolutionary network, which was viewed in Cytoscape 3.9.1 (Cytoscape Consortium) by using the prefuse force-directed method [16]. Finally, we analyzed the BA.1 (n=260) and BA.2 (n=4993) lineages separately to understand the fine-scale subdivision within each lineage.

For the second approach, we used the discriminant analysis of principal components (DAPC) method to understand the fine-scale subdivision patterns observed in each lineage (based on the Pango lineage definitions mentioned in the *Data Matrix and Cleanup* section). The DAPC is a useful method for detecting subdivision, as it maximizes between-group differences while minimizing within-group variability [17]. It is a relatively fast method for detecting complex subdivision patterns from genomic data. We used the filtered genomes obtained from VENAS and performed a DAPC for both lineages by using the R *adegenet* package (R Foundation for Statistical Computing) [17,18]. We first identified the optimal number of clusters within each data set, using the K-means algorithm, and then performed the DAPC. We further identified the unique mutations for each of the DAPC clusters and only considered mutations that were present in at least 70% of the sequences belonging to the cluster.

### Genomic Diversity and Selection Analysis

To estimate the level of genomic diversity within our data set, we characterized all substitutions in reference to the Wuhan genome by using VENAS. We considered all 14,003 good-quality sequences and identified the mutations for the 12 functional open reading frames (ORFs). We further estimated Tajima’s D values for the spike protein sequences for all of the clusters identified in the DAPC, using MEGA (Molecular Evolutionary Genetics Analysis; version 10.2.6 [Pennsylvania State University]) [19]. The Tajima’s D test is widely used to identify signatures of microevolutionary forces, such as population fluctuations and selections acting upon populations. We estimated the Tajima’s D value for each genetic cluster identified by the DAPC to avoid confounding effects of population subdivision.

## Results

### Genetic Subdivision

**Figure 2:**
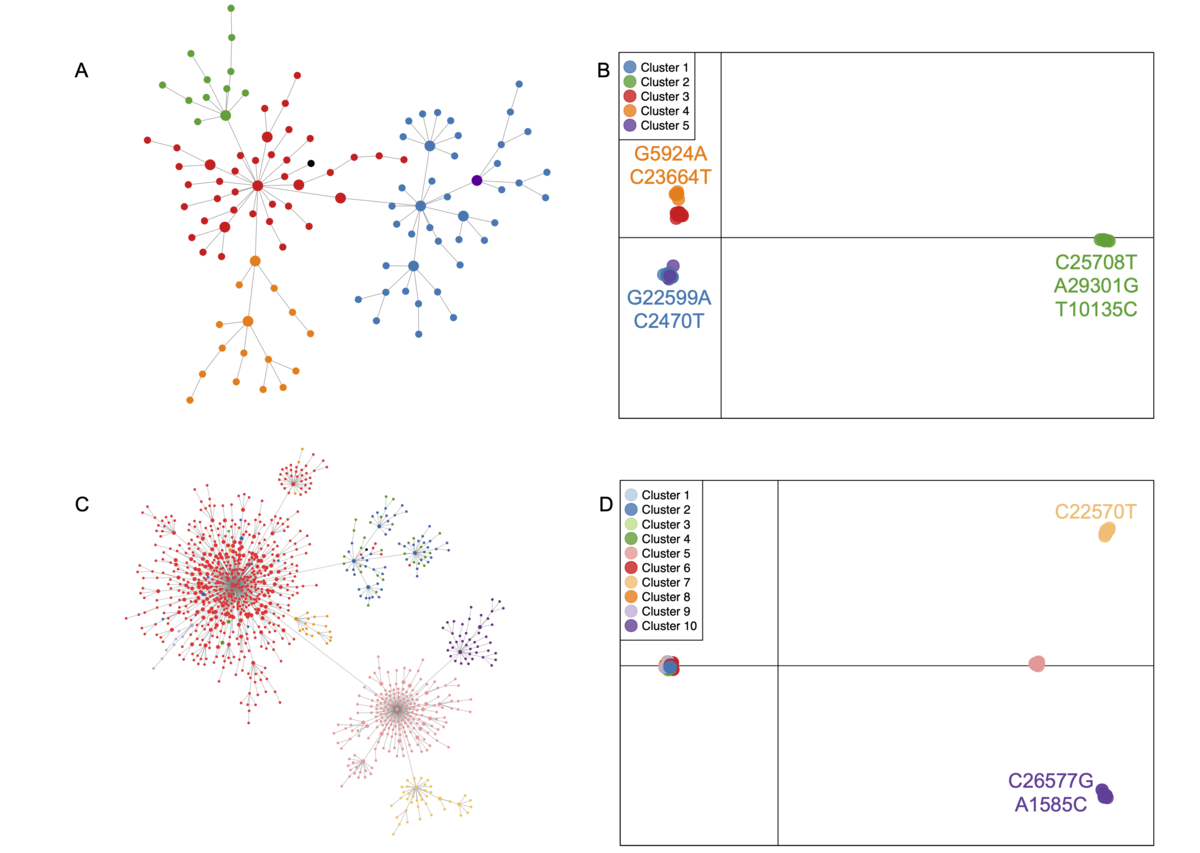
The genetic subdivision observed in the Omicron lineage of SARS-CoV-2 based on haplotype networks (panel A: BA.1 lineage; panel C: BA.2 lineage). Panels B and D depict observed genetic subdivision based on the discriminant analysis of principal components (DAPC) for the BA.1 and BA.2 lineages, respectively. The Wuhan sequence is denoted by the black-colored node in panels A and C. Private mutations, if any, are depicted in the DAPC plot. A private mutation must be present in at least 70% of the sequences within the cluster.

We observed signatures of genetic subdivision in the BA.1 and BA.2 lineages of the Omicron variant. The patterns were broadly concordant between both approaches—the evolutionary network approach conducted in VENAS and the statistical approach using the DAPC. Although VENAS produced numerous nodes and groups (BA.1: n=111; BA.2: n=1046), these were nested within fewer, broader subdivisions retrieved by the DAPC (Figure 2). We identified 5 clusters for the BA.1 lineage and 10 clusters for the BA.2 lineage, using the DAPC. However, when we visualized our results, we observed that 2 clusters for the BA.1 lineage were clubbed together, and 4 clusters for the BA.2 lineage were clubbed together (Figure 2B and Figure 2D). Further, no sequences were assigned to clusters 1 and 3 for the BA.2 lineage. Thus, effectively, only 4 clusters for the BA.1 and BA.2 lineages were identified by the DAPC method. Private mutations were identified for 5 clusters (Figure 2B and Figure 2D).

### Genomic Diversity

A detailed inspection of the pattern of substitution among the study genomes revealed that, as expected, the spike protein, ORF1a, and ORF1b harbored the maximum number of variations (Figure S1 in Multimedia Appendix 1). Gene coding for the envelope protein had the lowest rate of change. The most frequent mutations observed within our panel of genomes were C to T transition and G to T transversion (Figure S2 in Multimedia Appendix 1). Tajima’s D values for the spike protein sequences were negative for all of the DAPC clusters, with significant values observed only for 4 clusters (Tajima’s D<−2; Table 1).

**Table 1:** Tajima’s D estimates for the various clusters that were identified by using the discriminant analysis of principal components.

Cluster ID		Number of sequences	Sampling location	Number of segregating sites	θ	Nucleotide diversity	Tajima’s D
**BA.1 lineage**							
	Cluster 1	41	Belgium, Germany, India, Japan, Mexico, Romania, South Africa, Switzerland, Taiwan, and United States of America	16	0.00293	0.00071	−2.425328^a^
	Cluster 2	12	Denmark, Germany, India, Mexico, and United States of America	7	0.00182	0.00095	−1.866946
	Cluster 3	40	Denmark, England, Germany, India, Japan, Slovenia, South Africa, Switzerland, Taiwan, Thailand, and United States of America	11	0.00203	0.00073	−1.948315
	Cluster 4	16	Denmark, England, Germany, India, Romania, South Africa, Spain, and Switzerland	8	0.00189	0.00105	−1.598913
	Cluster 5	1	South Africa	N/A^b^	N/A	N/A	N/A
**BA.2 lineage**							
	Cluster 1	0	N/A	N/A	N/A	N/A	N/A
	Cluster 2	61	Australia, Denmark, India, Singapore, and South Africa	8	0.001343	0.00028	−2.08083^a^
	Cluster 3	0	N/A	N/A	N/A	N/A	N/A
	Cluster 4	31	Denmark, India, Norway, and Singapore	2	0.000393	0.00010	−1.50558
	Cluster 5	207	Denmark and Singapore	19	0.002527	0.00032	−2.31247^a^
	Cluster 6	632	Denmark, Singapore, and South Africa	50	0.005591	0.00027	−2.59423^a^
	Cluster 7	40	Denmark	3	0.000554	0.00019	−1.4309
	Cluster 8	22	Denmark	1	0.000215	0.00007	−1.16240
	Cluster 9	8	Denmark	1	0.000303	0.00020	−1.05482
	Cluster 10	44	Denmark	3	0.000542	0.00027	−1.07839

^a^Tajima’s D values of <−2 indicate significant demographic expansion or selection.

^b^N/A: not applicable.

## Discussion

### Study Overview

In this study, we investigated the effectiveness of population genomics methods to identify the fine-scale structure and demographic history of the Omicron lineage during the initial spread of the virus. Our study also highlights the utility of population genomics methods in handling large data sets and provides an analytical framework for future studies, which will help with understanding the genetic substructuring of the virus and identifying mutations that are potentially advantageous to the spread of the virus.

### Fine-Scale Subdivision Within the Omicron Variant

Using a combination of population genetics methods, this study revealed cryptic, fine-scale substructuring within our data set. We observed a similar pattern of subdivision for each Omicron lineage, using both VENAS and the DAPC (Figure 2). Although both methods use different approaches, together they provide a robust understanding of the finer subdivision patterns within fast-evolving lineages. At the start of this study, Pango lineage definition 3.1.20 was available, which had divided the Omicron sequences into the BA.1, BA.1.1, and BA.2 lineages, and our population genomics–based clustering identified finer-level subdivision within these lineages. With the updated Pango lineage version 4.0.6, there is now a broad agreement in the lineage definitions between our methodology (DAPC and VENAS) and the Pango lineage.

We identified cluster-defining mutations that were later selected for Pango lineage definitions, such as the G22599A and G5924A mutations for BA.1.1 (clusters 1 and 5 in the DAPC) and BA.1.17 (cluster 4 in the DAPC), respectively (Figure 2B). The subdivision observed in our study, as well as some of the cluster-defining mutations (G5924A, G22599A, C2470T, and A29301G), also agrees with recent phylogenetic reconstructions (Figure 2B) [20,21].

We also recovered signatures of fine-scale subdivision within the updated Pango (version 4.0.6) definitions. For example, DAPC clusters 5, 7, and 10, which are all part of the BA.2.9 lineage from Denmark (Figure 2D), were segregated based on 3 unique mutations (Figure 2D). The mutation C22570T is unique to cluster 7, and the mutations C26577G and A1585C are unique to cluster 10 within our data set (Figure 2D). Thus, our analytical regime could not only retrieve cluster-defining mutations in agreement with other methods but also identify finer subdivisions within existing Pango definitions and associated unique mutations.

### Selection and Demographic History of the Omicron Variant

Tests for selection revealed that the evolution of the Omicron lineage could be attributed to microevolutionary processes, such as selection and demographic expansion. We used Tajima’s D values to test for deviation of the identified clusters from neutrality. A negative Tajima’s D value reflects a deficit of haplotypes in comparison to the number of segregating sites and is indicative of a recent selective sweep or a population expansion [22]. Significant negative Tajima’s D values were observed for a subset of the DAPC clusters (BA.1 lineage: cluster 1; BA.2 lineage: clusters 2, 5, and 6; Table 1), suggesting that these clusters have undergone rapid expansion, experienced recent selective sweeps, or both. These attributes are indicators of greater transmissibility and thus make these clusters potential targets for surveillance and monitoring programs. For example, the spike protein mutation G22599A (S:R346K) is implicated in providing a transmission advantage and the antibody escape ability to the BA.1.1 variant. Although the population genomics framework adopted in our study identified this diagnostic mutation, which defines cluster 1 of the BA.1 lineage, the test for deviations from neutrality returned a significant negative value only for this cluster (Tajima’s D=**−**2.425328), indicating the selective advantage, as well as signals of population expansion, of this cluster across the globe (Figure 2B) [23-25]. In addition to G22599A (S:R346K), we also identified the mutation C23664T (S:A701V), which, in conjunction with S:N501Y, provides a mild advantage to the virus by increasing the rate of infection [20]. However, the C23664T mutation is not unique to the Omicron lineage and is also observed in other SARS-CoV-2 variants of concern [20].

Interestingly, cluster 2 of the BA.1 lineage, which did not exhibit a signature of expansion or selection, also harbors 3 unique mutations (C25708T, A29301G, and T10135C), which have been independently identified as suppressor mutations associated with a reduction in the spread of the virus [26] (Figure 2A and Figure 2B; Table 1).

Future research efforts can use a similar analytical framework to swiftly identify mutations that are important for virus evolution, of which some might play an important role in facilitating the spread of a virus, while others may be detrimental to its transmission. We demonstrated that a combination of population genomics methods can be used to recover subtle variations within established lineage definitions and potentially aid in finding variants of concern. The identification of such target mutations is necessary from an epidemiological standpoint, as well as for vaccine development. This study provides an easy analytical framework that can be used by policy makers to identify variants of potential concern and understand the local demographic history and spread of a virus, thereby facilitating disease mitigation.

### Conclusion

We provide an easy, computationally tractable framework for understanding the genetic subdivision and demographic history of SARS-CoV-2. Our framework can be quickly implemented to identify potentially important mutations that may be driving the spread of the virus. Such information can be very useful for deciphering the pattern of movement of variants and determining correlations with the local history of an outbreak.

## Appendix

Multimedia Appendix 1 Supporting information.

Multimedia Appendix 2 Acknowledgment table.

## Abbreviations

DAPC: discriminant analysis of principal components
GISAID: Global Initiative on Sharing All Influenza Data
MEGA: Molecular Evolutionary Genetics Analysis
ORF: open reading frame
VENAS: Viral Genome Evolution Network Analysis System

## Data Availability

The SARS-CoV-2 sequences analyzed during this study are available in the GISAID (Global Initiative on Sharing All Influenza Data) repository. Details of the sequences are available in Multimedia Appendix 2.
