# Supplementary material for: Genomic Insights Into the Evolution and Demographic History of the SARS-CoV-2 Omicron Variant: Population Genomics Approach
Source: JMIR Bioinform Biotechnol. 2023 Jun 12;4:e40673. doi: 10.2196/40673 (PMC10331448; doi:10.2196/40673)
Supplement: Multimedia Appendix 1 [file bioinform_v4i1e40673_app1.docx]

**Population genomic insights into the evolution of the SARS-CoV-2 Omicron variant**

**FIGURES**

Figure S1: Distribution of the number of mutations for each open reading frame of SARS-CoV-2 sequences belonging to omicron lineage analyzed in this study. We characterized the mutation in reference to the Wuhan genome.

Figure S2: Frequency distribution of the number of A) transitions, and B) transversion for each open reading frame of SARS-CoV-2 sequences belonging to omicron lineage analyzed in this study. We characterized the mutation in reference to the Wuhan genome.
