# Supplementary material for: Genomic Insights Into the Evolution and Demographic History of the SARS-CoV-2 Omicron Variant: Population Genomics Approach
Source: JMIR Bioinform Biotechnol. 2023 Jun 12;4:e40673. doi: 10.2196/40673 (PMC10331448; doi:10.2196/40673)
Supplement: Multimedia Appendix 2 [file bioinform_v4i1e40673_app2.pdf]

We gratefully acknowledge the following Authors from the Originating laboratories responsible for obtaining the specimens, as well as the Submitting laboratories where the genome data were generated and shared via GISAID, on which this research is based.

All Submitters of data may be contacted directly via www.gisaid.org

Authors are sorted alphabetically.

Accession ID	Originating Laboratory	Submitting Laboratory	Authors
EPI_ISL_7543686	2 Military Hospital wc MAA	NHLS/UCT	Arash Iranzadeh; Bruna Galvao; Carolyn Williamson; Deelan Doolabh; Diana Hardie; Gert Marais; Innocent Mudau; Luicer Olubayo; Lynn Tyers; Marvin Hsiao; Nokuzola Mbhele; Rageema Joseph; Stephen Korsman
EPI_ISL_7987418	4Cyte Pathology	NSW Health Pathology - Institute of Clinical Pathology and Medical Research; Westmead Hospital; University of Sydney	Arnott A.; Draper J.; Gall M.; Martinez E.; Rockett R.; Sintchenko V.; on behalf of ICPMR
EPI_ISL_7929179	A. Krumbholz, Labor Dr. Krause und Kollegen MVZ GmbH, Kiel	Charité Universitätsmedizin Berlin, Institut für Virologie	Andi Krumbholz; Barbara Mühlemann; Christian Drosten; Julia Schneider; Julia Tesch; Jörn Beheim-Schwarzbach; Talitha Veith; Terry Jones; Tobias Bleicker; Victor M Corman
EPI_ISL_8077636, EPI_ISL_8078464, EPI_ISL_8149646, EPI_ISL_8149727, EPI_ISL_8150203	Aegis Sciences Corporation	Centers for Disease Control and Prevention Division of Viral Diseases, Pathogen Discovery	Alec Vest; Benjamin Rambo-Martin; Christopher Gulvick; Clinton Paden; Cyndi Clark; Dakota Howard; Dhwani Batra; Dillon Nall; Duncan MacCannell; Erisa Sula; Ethan Sanders; Holly Houdeshell; Jason Caravas; Kristine Lacek; Matthew Hardison; Matthew Schmerer; Ola Kvalvaag; Patrick Campbell; Peter Cook; Rob Case; Scott Sammons; Shatavia Morrison; Shaun Westlund; Tymeckia Kendall; Victoria Caban Figueroa; Vikramsinha Ghorpade; Yvette Unoarumhi
EPI_ISL_8056916	Alfa Health Care Diagnostic Center	National Public Health Laboratory	National Public Health Laboratory Team/ Alfa Health Care Diagnosyic Center
EPI_ISL_8015285	Analytica	Institute of Medical Virology, University of Zurich	Alexandra Trkola; Annette Audigé; Catharine Aquino; Cyril Shah; Daniel Ehrsam; Gabriela Ziltener; Guido Bloemberg; Hubert Rehrauer; Isabel Stürmer; Joel Wirz; Jon Huder; Jürg Böni; Kevin Steiner; Maria Grünberg; Maryam Zaheri; Michael Huber; Riccarda Capaul; Stefan Schmutz; Verena Kufner; Weihong Qi
EPI_ISL_8132322	Arcispedale Santa Maria Nuova Autoimmunita Allergologia e Biotecnologie Innovative""	Istituto Zooprofilattico Sperimentale della Lombardia e dell'Emilia Romagna (IZSLER), Risk Analysis and Genomic Epidemiology Unit	Erika Scaltriti; Ilaria Menozzi; Marina Morganti; Stefano Pongolini
EPI_ISL_7616000	Ayass Bioscience LLC	Ayass Bioscience LLC	Kevin Zhu; Lina Abi Mosleh; Mohamad Ammar Ayass; Natalya Griko; Nazanin Taheri
EPI_ISL_8132350, EPI_ISL_8132365	Azienda Ospedaliero - Universitaria di Modena Policlinico - Virologia e Microbiologia Molecolare""	Istituto Zooprofilattico Sperimentale della Lombardia e dell'Emilia Romagna (IZSLER), Risk Analysis and Genomic Epidemiology Unit	Erika Scaltriti; Ilaria Menozzi; Marina Morganti; Stefano Pongolini
EPI_ISL_8132317, EPI_ISL_8134455	Azienda Sanitaria Locale di Piacenza - Presidio Ospedaliero - Laboratorio di Microbiologia""	Istituto Zooprofilattico Sperimentale della Lombardia e dell'Emilia Romagna (IZSLER), Risk Analysis and Genomic Epidemiology Unit	Erika Scaltriti; Ilaria Menozzi; Marina Morganti; Stefano Pongolini
EPI_ISL_7196120, EPI_ISL_7196121	Bidh Lab	National Public Health Laboratory	Bidh Laboartory; National Public Health Laboratory Team
EPI_ISL_7566330, EPI_ISL_7566360	Botswana Harvard HIV Reference Laboratory	Botswana Harvard AIDS Institute Partnership, Plot 1836 North Ring Road, Princess Marina Hospital, Gaborone	Boitumelo Zuze; Botshelo Radibe; Dorcas Maruapula; Doreen Ditshwanelo; Joseph Makhema; Keoratile Ntshambiwa; Kgomotso Moruisi; Legodile Kooepile; Mosepele Mosepele; Mphaphi B. Mbulawa; Ontlametse T. Bareng; Pamela Smith-Lawrence; Roger Shapiro; Sefetogi Ramaologa; Shahin Lockman; Sikhulile Moyo; Simani Gaseitsiwe; Thongbotho Mphoyakgosi; Wonderful T. Choga
EPI_ISL_7834399, EPI_ISL_7834407, EPI_ISL_7834409, EPI_ISL_7834416, EPI_ISL_7834419, EPI_ISL_7834422, EPI_ISL_7834424, EPI_ISL_7834425, EPI_ISL_7834426, EPI_ISL_7834432, EPI_ISL_7834433, EPI_ISL_7834434, EPI_ISL_7834437, EPI_ISL_7834438, EPI_ISL_7834439, EPI_ISL_7834440, EPI_ISL_7834441, EPI_ISL_7834442, EPI_ISL_7834443, EPI_ISL_7834444, EPI_ISL_7834446, EPI_ISL_7834447, EPI_ISL_7834449, EPI_ISL_7834451, EPI_ISL_7834452, EPI_ISL_7834453, EPI_ISL_7834455, EPI_ISL_7834456, EPI_ISL_7834457, EPI_ISL_7834458, EPI_ISL_7834459, EPI_ISL_7834461, EPI_ISL_7834462, EPI_ISL_7834463, EPI_ISL_7834465, EPI_ISL_7834466, EPI_ISL_7834467, EPI_ISL_7834468, EPI_ISL_7834472, EPI_ISL_7834473, EPI_ISL_7834475, EPI_ISL_7834476, EPI_ISL_7834477, EPI_ISL_7834480, EPI_ISL_7834481, EPI_ISL_7834482, EPI_ISL_7834493, EPI_ISL_7834502, EPI_ISL_7834514, EPI_ISL_7834555, EPI_ISL_7834590, EPI_ISL_7834591, EPI_ISL_7834600			
see above	Botswana Harvard HIV Reference Laboratory	Botswana Harvard HIV Reference Laboratory	Boitumelo Zuze; Botshelo Radibe; Dorcas Maruapula; Joseph Makhema; Keoratile Ntshambiwa; Kgomotso Moruisi; Legodile Kooepile; Mosepele Mosepele; Mphaphi B. Mbulawa; Ontlametse T. Bareng; Pamela Smith-Lawrence; Roger Shapiro; Sefetogi Ramaologa; Shahin Lockman; Sikhulile Moyo; Simani Gaseitsiwe; Thongbotho Mphoyakgosi; Wonderful T. Choga
EPI_ISL_7747509	CAPRISA_HILLCREST	CERI, Centre for Epidemic Response and Innvoation, Stellenbosch University and KRISP, KZN Research Innovation and Sequencing Platform, UKZN.	Aida Sivro; Arisha Maharaj; Giandhari J; Naidoo Y; Natasha Samsunder; Pillay S; Ramphal U; Ramphal Y; San JE; Tegally H; Tshiabuila D; Wilkinson E; de Oliveira T
EPI_ISL_8109017	CHC Dakor	Gujarat Biotechnology Research Centre	Akhilesh Modi; Apurvasinh Puvar; Bhadreshsinh Gohil; Chaitanya Joshi; Disha Vora; Janvi Raval; Jaykumar Rangani; Madhvi Joshi; Nimesh Patel; Nitin Savaliya; Nitin Shukla; Priyank Chavda; Ramesh Pandit; Roshani Mishra; Sonal Sharma; Tasnim Trivedi; Zarna Patel
EPI_ISL_7337481, EPI_ISL_7337497	Charlotte Maxeke Johannesburg Academic Hospital	National Institute for Communicable Diseases of the National Health Laboratory Service	Amoako DG; Bhiman JN; Everatt J; Ismail A; Mahlangu B; Mnguni A; Mohale T; Ntuli N; Scheepers C
EPI_ISL_7988984, EPI_ISL_7988985, EPI_ISL_8099510	Clinical Microbiology Laboratory, Tel Aviv Sourasky Medical Center	Clinical Microbiology Laboratory, Tel Aviv Sourasky Medical Center	Alon Ziv; Amos Adler; Katya Levytskyi; Lior Handler; Ora Halutz
EPI_ISL_7829627	Clinique Saint-Pierre Ottignies	UCLouvain/IREC/MBLG-CTMA	Benoit Kabamba Mukadi; Bertrand Bearzatto; Jean-Luc Gala; Nicolas Pinte; Paul Blanpain; Simon Ophélie; Valentin Coste
EPI_ISL_7829625, EPI_ISL_7829626, EPI_ISL_7829631	Cliniques universitaires Saint-Luc	UCLouvain/IREC/MBLG-CTMA	Benoit Kabamba Mukadi; Bertrand Bearzatto; Jean-Luc Gala; Nicolas Pinte; Paul Blanpain; Simon Ophélie; Valentin Coste
EPI_ISL_8122388	Colorado Department of Public Health and Environment	Colorado Department of Public Health and Environment	Alexandria Rossheim; Diana Ir; Emily A. Travanty; Laura Bankers; Mandy Waters; Michael A. Martin; Molly C. Hetherington-Rauth; Sarah Elizabeth Totten; Shannon R. Matzinger
EPI_ISL_7906576, EPI_ISL_8002167, EPI_ISL_8002210, EPI_ISL_8053581, EPI_ISL_8053663, EPI_ISL_8053917, EPI_ISL_8134681, EPI_ISL_8135005, EPI_ISL_8135069, EPI_ISL_8135113, EPI_ISL_8135280, EPI_ISL_8135299, EPI_ISL_8135305, EPI_ISL_8135486, EPI_ISL_8135495, EPI_ISL_8135593, EPI_ISL_8135685, EPI_ISL_8135710, EPI_ISL_8135886, EPI_ISL_8136001, EPI_ISL_8136019, EPI_ISL_8136050, EPI_ISL_8136091, EPI_ISL_8136635, EPI_ISL_8136749, EPI_ISL_8136811, EPI_ISL_8136950, EPI_ISL_8137224, EPI_ISL_8137308, EPI_ISL_8137566, EPI_ISL_8137955, EPI_ISL_8138431, EPI_ISL_8138663, EPI_ISL_8138852, EPI_ISL_8139086, EPI_ISL_8139268			
see above	Department of Bacteria, Parasites and Fungi, Statens Serum Institut, Copenhagen, Denmark	Statens Serum Institut Bioinformatics and Microbial Genomics	Danish Covid-19 Genome Consortium
EPI_ISL_8020345	Department of Clinical Microbiology	GIGA Medical Genomics	Bouchra Boujemla; Claire Gourzonès; Cécile Meex; Keith Durkin; Laurent Gillet; Maria Artesi; Marie-Pierre Hayette; Nadine Cambisano; Nathalie Renotte; Olivier Ek; Sébastien Bontems; Vincent Bours
EPI_ISL_7910635	Department of Clinical Microbiology and Center for Genomic Medicine, Rigshospitalet, Copenhagen, Denmark	Statens Serum Institut Bioinformatics and Microbial Genomics	Danish Covid-19 Genome Consortium
EPI_ISL_7192734	Department of Health Technology and Informatics, The Hong Kong Polytechnic University	Department of Health Technology and Informatics, The Hong Kong Polytechnic University	Alan Ka-Lun Wu; Alex Yat-Man Ho; Barry Kin-Chung Wong; Chloe Toi-Mei Chan; David Ho-Keung Shum; Denise Sze-Hang Wong; Gilman Kit-Hang Siu; Hiu-Yin Lao; Hoi-Ching Jim; Ivan Tak-Fai Wong; Jake Siu-Lun Leung; Kam-Tong Yip; Kenneth Siu-Sing Leung; Kingsley King-Gee Tam; Kitty Sau-Chun Fung; Kristine Luk; Lam-Kwong Lee; Miranda Chong-Yee Yau; Sandy Ka-Yee Chau; Shea Ping Yip; Tak-Lun Que; Timothy Ting-Leung Ng; Wing Cheong Yam; Wing-Hei Lo; Wing-Kin To; Yvette Wai-Man Lai
EPI_ISL_7623676	Dept of Microbilogy, Maulana Azad Medical College	J6094_MAMC_LNH	Avinash Lomash; Mohammad Faruq; Oves Siddiqui; SCOG_MAMC_LNH; Seema Kapoor. Sunil Kumar Polipalli; Somesh Kumar; Suresh Kumar
EPI_ISL_8063678	Fondazione IRCCS Ca' Granda Ospedale Maggiore Policlinico	Fondazione IRCCS Ca' Granda Ospedale Maggiore Policlinico	Ferruccio Ceriotti; Sara Uceda Renteria
EPI_ISL_8060345, EPI_ISL_8060974, EPI_ISL_8080063, EPI_ISL_8091398, EPI_ISL_8157487	Fulgent Genetics	Centers for Disease Control and Prevention Division of Viral Diseases, Pathogen Discovery	Becky Tsai; Benafsh Sapra; Benjamin Rambo-Martin; Christopher Gulvick; Clinton Paden; Dakota Howard; Dhwani Batra; Doreen Ng; Duncan MacCannell; Erisa Sula; Harry Gao; James Xie; Jason Caravas; John Gao; Joseph Fierro; Kristine Lacek; Matthew Schmerer; Mickey Li; Peter Cook; Scott Sammons; Shatavia Morrison; Tymeckia Kendall; Victoria Caban Figueroa; Yan Meng; Yvette Unoarumhi
EPI_ISL_8020172	Gesundheitsdirektion Zürich	Institute of Medical Virology, University of Zurich	Alexandra Trkola; Annette Audigé; Catharine Aquino; Cyril Shah; Daniel Ehrsam; Gabriela Ziltener; Guido Bloemberg; Hubert Rehrauer; Isabel Stürmer; Joel Wirz; Jon Huder; Jürg Böni; Kevin Steiner; Maria Grünberg; Maryam Zaheri; Michael Huber; Riccarda Capaul; Stefan Schmutz; Verena Kufner; Weihong Qi
EPI_ISL_7740761	HELEN JOSEPH LABORATORY	National Institute for Communicable Diseases of the National Health Laboratory Service	Amoako DG; Bhiman JN; Everatt J; Ismail A; Mahlangu B; Mnguni A; Mohale T; Ntuli N; Scheepers C; Wolter N
EPI_ISL_7337441	Helen Joseph Laboratory	National Institute for Communicable Diseases of the National Health Laboratory Service	Amoako DG; Bhiman JN; Everatt J; Ismail A; Mahlangu B; Mnguni A; Mohale T; Ntuli N; Scheepers C
EPI_ISL_7265104, EPI_ISL_7749559	Histopath	NSW Health Pathology - Institute of Clinical Pathology and Medical Research; Westmead Hospital; University of Sydney	Arnott A.; Draper J.; Gall M.; Martinez E.; Rockett R.; Sintchenko V.; on behalf of ICPMR
EPI_ISL_8002519	Hospital General Universitario Gregorio Marañón	Hospital General Universitario Gregorio Marañón	Cristina Rodriguez-Grande; Darío García de Viedma; Jorge Rodríguez-Grande; Julia Suárez; Laura Pérez-Lago; Marta Herranz Martin; Patricia Muñoz; Pedro Sola Campoy; Pilar Catalán; Sergio Buenestado Serrano
EPI_ISL_7734081, EPI_ISL_7977800, EPI_ISL_7978212, EPI_ISL_7978248, EPI_ISL_7978362, EPI_ISL_7978363, EPI_ISL_7978635, EPI_ISL_7978638, EPI_ISL_7979184, EPI_ISL_7979535, EPI_ISL_8030372			
see above	Houston Methodist Hospital	Houston Methodist Hospital	Akanksha Batajoo; Ilya J. Finkelstein; James J. Davis; Jessica Cambric; Jimmy Gollihar; Kristina Reppond; Layne Pruitt; Madison N. Shyer; Marcus Nguyen; Matthew Ojeda Saavedra; Paul A. Christensen; Prasanti Yerramilli; Randall J. Olsen; Rashi Thakur; Regan Mangham; Richard Snehall; Robert Olson; Ryan Gadd; S. Wesley Long; Sindy Pena; Sishir Subedi; and James M. Musser
EPI_ISL_8119611	IZSM-Virologia	Istituto Zooprofilattico Sperimentale del Mezzogiorno - Unità Operativa Complessa di Virologia	Antonio Limone; Claudio de Martinis; Ester De Carlo; Giovanna Fusco; Loredana Cozzolino; Lorena Cardillo; Maurizio Viscardi
EPI_ISL_7496734	Idaho Bureau of Laboratories	Idaho Bureau of Laboratories	Aimee Ceniseros; Christian Loera; Christopher Ball; Matthew Charles Burns; R. Beukelman; Robert L. Voermans
EPI_ISL_7381102	Indian Council of Medical Research-National Institute of Virology, Microbial Containment Complex	Indian Council of Medical Research-National Institute of Virology, Microbial Containment Complex	Pragya D. Yadav
EPI_ISL_8100211, EPI_ISL_8100260	Institute of Microbiology and Immunology,	Institute of Microbiology and Immunology, Faculty of	Alen Suljič; Andraž Celar; Domen Lazar; Doroteja Vlaj; Mario Poljak; Miša Korva; Patricija Pozvek; Samo Zakotnik; Tatjana Avšič – Županc; Tina Gabrovšek; Tina Živič; Tomaž Mark Zorec; Špela Pleh

	Faculty of Medicine, University of Ljubljana	Medicine, University of Ljubljana	
EPI_ISL_7955281	Integrated Covid Hub North East	Wellcome Sanger Institute for the COVID-19 Genomics UK (COG-UK) Consortium	Cordelia Langford; David K. Jackson; Dominic Kwiatkowski; Ewan Harrison; Ian Johnston; Integrated Covid Hub North East and Alex Alderton; Jeffrey Barrett; John Sillitoe on behalf of the Wellcome Sanger Institute COVID-19 Surveillance Team; Roberto Amato; Sonia Goncalves
EPI_ISL_7632052, EPI_ISL_8015288, EPI_ISL_8120111	Invenimus AG	Institute of Medical Virology, University of Zurich	Alexandra Trkola; Annette Audigé; Catharine Aquino; Cyril Shah; Daniel Ehrsam; Gabriela Ziltener; Guido Bloemberg; Hubert Rehrauer; Isabel Stürmer; Joel Wirz; Jon Huder; Jürg Böni; Kevin Steiner; Maria Grünberg; Maryam Zaheri; Michael Huber; Riccarda Capaul; Stefan Schmutz; Verena Kufner; Weihong Qi
EPI_ISL_7789703, EPI_ISL_7790175, EPI_ISL_7982412	KU Leuven, Rega Institute, Clinical and Epidemiological Virology	KU Leuven, Rega Institute, Clinical and Epidemiological Virology	Bert Vanmechelen; Joan Marti-Carerras; Piet Maes; Tony Wawina-Bokalanga
EPI_ISL_8015322, EPI_ISL_8015323, EPI_ISL_8119804	Kantonsspital Aarau	Institute of Medical Virology, University of Zurich	Alexandra Trkola; Annette Audigé; Catharine Aquino; Cyril Shah; Daniel Ehrsam; Gabriela Ziltener; Guido Bloemberg; Hubert Rehrauer; Isabel Stürmer; Joel Wirz; Jon Huder; Jürg Böni; Kevin Steiner; Maria Grünberg; Maryam Zaheri; Michael Huber; Riccarda Capaul; Stefan Schmutz; Verena Kufner; Weihong Qi
EPI_ISL_8015340, EPI_ISL_8015409, EPI_ISL_8120125	Kantonsspital Winterthur	Institute of Medical Virology, University of Zurich	Alexandra Trkola; Annette Audigé; Catharine Aquino; Cyril Shah; Daniel Ehrsam; Gabriela Ziltener; Guido Bloemberg; Hubert Rehrauer; Isabel Stürmer; Joel Wirz; Jon Huder; Jürg Böni; Kevin Steiner; Maria Grünberg; Maryam Zaheri; Michael Huber; Riccarda Capaul; Stefan Schmutz; Verena Kufner; Weihong Qi
EPI_ISL_8097223, EPI_ISL_8097234, EPI_ISL_8097249, EPI_ISL_8097255, EPI_ISL_8097263, EPI_ISL_8097265, EPI_ISL_8097268, EPI_ISL_8097272, EPI_ISL_8097273, EPI_ISL_8097276, EPI_ISL_8097279, EPI_ISL_8097282			
see above	Kasane Primary Hospital Laboratory	Botswana Harvard HIV Reference Laboratory	Agnes Karutwaeng; Boitumelo Zuze; Botshelo Radibe; Dorcas Maruapula; Joseph Makhema; Keoratile Ntshambiwa; Legodile Kooepile; Mosepele Mosepele; Mphapi B. Mbulawa; Ontlametse T. Bareng; Roger Shapiro; Rose Munyere; Sefetogi Ramaologa; Shahin Lockman; Sikhulile Moyo; Simani Gaseitsiwe; Thela Tefelo; Thongbotho Mphoyakgosi; Wonderful T. Choga
EPI_ISL_7986436, EPI_ISL_7986439	Kasturba Hospital Molecular Lab - INSACOG	Kasturba hospital WGS	Jayanthi Shastri; Vidushi Chitalia
EPI_ISL_7701129	LANCET LABORATORY	National Institute for Communicable Diseases of the National Health Laboratory Service	Amoako DG; Bhiman JN; Everatt J; Ismail A; Mahlangu B; Mnguni A; Mohale T; Ntuli N; Scheepers C; Wolter N
EPI_ISL_7651289	Laboratory of Molecular Biology and Cancer Immunology, Faculty of Sciences, Lebanese University	Microbial Pathogenomics Lab - LAU	Alissar Zaghlout; Bassam Badran; Fadi Abdel Sater; Georgi Merhi; Jad Koweyes; Nada Ghosn; Rawan Makki; Sima tokajian
EPI_ISL_7834554	Lancet Laboratories Gaborone	Botswana Harvard HIV Reference Laboratory	Boitumelo Zuze; Botshelo Radibe; Dorcas Maruapula; Ellen Nkitse; Joseph Makhema; Keoratile Ntshambiwa; Kgomotso Moruisi; Legodile Kooepile; Leyi Ncube; Mosana Moyo; Mosepele Mosepele; Mphaphi B. Mbulawa; Ontlametse T. Bareng; Pamela Smith-Lawrence; Roger Shapiro; Sefetogi Ramaologa; Shahin Lockman; Sikhulile Moyo; Simani Gaseitsiwe; Thongbotho Mphoyakgosi; Wonderful T. Choga
EPI_ISL_7682986, EPI_ISL_7682990	Lighthouse Lab in Glasgow	Wellcome Sanger Institute for the COVID-19 Genomics UK (COG-UK) Consortium	Anna Dominiczak and Alex Alderton; Carol Clugston; Cordelia Langford; David Gray; David K. Jackson; Dominic Kwiatkowski; Ewan Harrison; Harper VanSteenhouse; Ian Johnston; Jeffrey Barrett; John Sillitoe on behalf of the Wellcome Sanger Institute COVID-19 Surveillance Team; Roberto Amato; Sonia Goncalves; Yumi Kasai
EPI_ISL_7864703	Lok Kayak Hospital	Lok Kayak Hospital	Avinash Lomash; Mohammed FARUQ; Oves Siddiqui; Prashanth Suravajhala SURESH Kumar; Somesh Kumar; Sunil k Polipalli; seema Kapoor and SCOG_MAMC_LNH Lab
EPI_ISL_7877006, EPI_ISL_7877026, EPI_ISL_7877093, EPI_ISL_7877115, EPI_ISL_7877191, EPI_ISL_7877201, EPI_ISL_7877202, EPI_ISL_7877203, EPI_ISL_7877297, EPI_ISL_7889640, EPI_ISL_7889641			
see above	Lok Nayak Hospital	Lok Nayak Hospital	; Avinash Lomash; Lok Nayak Hospital; Mohammed FARUQ; Oves Siddiqui; Prashanth Suravajhala; SURESH Kumar; Somesh Kumar; Sunil k Polipalli; seema Kapoor and Genome Sequencing Lab
EPI_ISL_7876997	Lok Nayak Hospital, Delhi	Lok Nayak Hospital, metro Station Central, Jawaharlal Nehru Marg, Near Delhi Gate, New Delhi, Delhi 110002	Avinash Lomash; Lok Nayak Hospital; Mohammed FARUQ; Oves Siddiqui; Prashanth Suravajhala; SURESH Kumar; Somesh Kumar; Sunil k Polipalli; seema Kapoor and Genome Sequencing Lab
EPI_ISL_8123968	MS PHL	MS PHL	Beata Karowlewicz; Daphne Ware
EPI_ISL_7433660	Medizinisch-Diagnostisches Labor Kempten allgäulab	Robert Koch Institute	
EPI_ISL_7620911	Medlab Pathology	NSW Health Pathology - Institute of Clinical Pathology and Medical Research; Westmead Hospital; University of Sydney	Arnott A.; Draper J.; Gall M.; Martinez E.; Rockett R.; Sintchenko V.; on behalf of ICPMR
EPI_ISL_6854346, EPI_ISL_6854347, EPI_ISL_6854348	Microbiologia e Virologia Cotugno	Microbiologia e Virologia Cotugno	Antonio Canonico; Antonio Fascione; Claudia Tiberio; Enza Mallardo; Francesco Nappo; Giovanni D'Auria; Giuseppe di Gennaro; Ilaria Cavallaro; Luigi Atripaldi
EPI_ISL_7552702, EPI_ISL_7552703, EPI_ISL_7552707	National Health Laboratory	Botswana Harvard AIDS Institute Partnership, Plot 1836 North Ring Road, Princess Marina Hospital, Gaborone	Boitumelo Zuze; Botshelo Radibe; Dorcas Maruapula; Doreen Ditshwanelo; Joseph Makhema; Keoratile Ntshambiwa; Kgomotso Moruisi; Legodile Kooepile; Mosepele Mosepele; Mphaphi B. Mbulawa; Ontlametse T. Bareng; Pamela Smith-Lawrence; Roger Shapiro; Sefetogi Ramaologa; Shahin Lockman; Sikhulile Moyo; Simani Gaseitsiwe; Thongbotho Mphoyakgosi; Wonderful T. Choga
EPI_ISL_7545782, EPI_ISL_7747500	National Health Laboratory Service, Kwazulu-Natal, South Africa	CERI, Centre for Epidemic Response and Innvoation, Stellenbosch University and KRISP, KZN Research Innovation and Sequencing Platform, UKZN.	Arisha Maharaj; Giandhari J; Naidoo Y; Nokukhanya Mdlalose; Pillay S; Ramphal U; Ramphal Y; San JE; Tegally H; Tshiabuila D; Wilkinson E; de Oliveira T
EPI_ISL_7969370	National Platform bis UMONS/Jolimont	National Platform bis UMONS/Jolimont	Eric Tarantino; Florian Juszczak; Gautier Detry; Guillaume Bayon-Vicente; Laetitia Gheysen; Ruddy Wattiez
EPI_ISL_8092780, EPI_ISL_8092783, EPI_ISL_8112317, EPI_ISL_8112348	National Public Health Laboratory, National Centre for Infectious Diseases	National Public Health Laboratory, National Centre for Infectious Diseases	Benny Yeo; Katherine Ching; Lin Cui; Raymond Tzer Pin Lin; Royce Ang; Samuel Loo; Zhenyang Zhou
EPI_ISL_8065975	Noguchi Memorial Institute for Medical Research, University of Ghana, Legon, Ghana	Noguchi Memorial Institute for Medical Research, University of Ghana, Legon, Ghana	Bright Adu; Hilda Opoku Frempong; John K. Odoom; Joseph K. H. Bonney; Joyce Appiah-Kubi; Keren O. Attiku; Mohktar; Peace Uche; Quaneeta; Seyram B. Avonyo
EPI_ISL_8097205	Nyanyangbwe HIV Reference Laboratory	Botswana Harvard HIV Reference Laboratory	Boitumelo Zuze; Botshelo Radibe; David Matema; Dorcas Maruapula; Joseph Makhema; Keoratile Ntshambiwa; Legodile Kooepile; Lindani Bochena; Mosepele Mosepele; Mphapi B. Mbulawa; Ontlametse T. Bareng; Roger Shapiro; Sefetogi Ramaologa; Shahin Lockman; Sikhulile Moyo; Simani Gaseitsiwe; Thela Tefelo; Thongbotho Mphoyakgosi; Wonderful T. Choga
EPI_ISL_7552440, EPI_ISL_7799914, EPI_ISL_7799945, EPI_ISL_8083804, EPI_ISL_8083805, EPI_ISL_8083815, EPI_ISL_8083816, EPI_ISL_8083822, EPI_ISL_8083823, EPI_ISL_8083825, EPI_ISL_8083826, EPI_ISL_8083830, EPI_ISL_8083832, EPI_ISL_8083833, EPI_ISL_8083835, EPI_ISL_8083836, EPI_ISL_8083839, EPI_ISL_8083843, EPI_ISL_8083844, EPI_ISL_8083846, EPI_ISL_8083850, EPI_ISL_8083852, EPI_ISL_8083853, EPI_ISL_8083854, EPI_ISL_8083855, EPI_ISL_8083860, EPI_ISL_8083864, EPI_ISL_8083867, EPI_ISL_8083868, EPI_ISL_8083870, EPI_ISL_8083872, EPI_ISL_8083873, EPI_ISL_8083874, EPI_ISL_8083875, EPI_ISL_8083876, EPI_ISL_8083878, EPI_ISL_8083879, EPI_ISL_8083880, EPI_ISL_8083882, EPI_ISL_8083884, EPI_ISL_8083888, EPI_ISL_8083889, EPI_ISL_8083890, EPI_ISL_8083894, EPI_ISL_8083896, EPI_ISL_8083900, EPI_ISL_8083901, EPI_ISL_8083902, EPI_ISL_8083905, EPI_ISL_8083906, EPI_ISL_8083907, EPI_ISL_8083908, EPI_ISL_8083909			
see above	Ochsner Health	BioInfoExperts	Amy Feehan; Ben Lain; Chris Huston; David J. Nolan; Judy Crabtree; Julia-Garcia-Diaz; Lucio Miele; Rebecca Rose; Samual Moot; Susanna L. Lamers; Tessa LaFleur
EPI_ISL_8142222	P.D.U Medical College Rajkot	Gujarat Biotechnology Research Centre	Akhilesh Modi; Apurvasinh Puvar; Bhadreshsinh Gohil; Chaitanya Joshi; Disha Vora; Janvi Raval; Jaykumar Rangani; Madhvi Joshi; Madhvlika Mistry; Nimesh Patel; Nitin Savaliya; Nitin Shukla; Priyank Chavda; Ramesh Pandit; Roshani Mishra; Sonal Sharma; Tasnim Trivedi; Zarna Patel
EPI_ISL_7190366, EPI_ISL_8132552, EPI_ISL_8132660, EPI_ISL_8132661, EPI_ISL_8132662, EPI_ISL_8132663, EPI_ISL_8132665, EPI_ISL_8132667, EPI_ISL_8132668, EPI_ISL_8132669, EPI_ISL_8132674, EPI_ISL_8132677, EPI_ISL_8132678, EPI_ISL_8132681, EPI_ISL_8132683, EPI_ISL_8132686, EPI_ISL_8132687, EPI_ISL_8132689, EPI_ISL_8132690, EPI_ISL_8132691, EPI_ISL_8132692, EPI_ISL_8132693, EPI_ISL_8132694, EPI_ISL_8132696, EPI_ISL_8132697, EPI_ISL_8132698, EPI_ISL_8132700, EPI_ISL_8132701, EPI_ISL_8132734, EPI_ISL_8132736, EPI_ISL_8132737, EPI_ISL_8132740, EPI_ISL_8132741, EPI_ISL_8132743, EPI_ISL_8132747, EPI_ISL_8132748, EPI_ISL_8132752, EPI_ISL_8132755, EPI_ISL_8132756, EPI_ISL_8132758, EPI_ISL_8132764, EPI_ISL_8132765, EPI_ISL_8132767, EPI_ISL_8132770, EPI_ISL_8132773, EPI_ISL_8132777, EPI_ISL_8132778, EPI_ISL_8132782, EPI_ISL_8132783, EPI_ISL_8132787, EPI_ISL_8132788, EPI_ISL_8132789, EPI_ISL_8132792, EPI_ISL_8132793, EPI_ISL_8132794, EPI_ISL_8132795, EPI_ISL_8132797, EPI_ISL_8132800, EPI_ISL_8132805, EPI_ISL_8132806, EPI_ISL_8132808, EPI_ISL_8132810, EPI_ISL_8132811, EPI_ISL_8132813, EPI_ISL_8132814, EPI_ISL_8132817, EPI_ISL_8132819, EPI_ISL_8132821, EPI_ISL_8132822, EPI_ISL_8132823, EPI_ISL_8132825, EPI_ISL_8132826, EPI_ISL_8132827, EPI_ISL_8132829, EPI_ISL_8132830, EPI_ISL_8132831, EPI_ISL_8132832, EPI_ISL_8132834, EPI_ISL_8132835, EPI_ISL_8132836, EPI_ISL_8132837, EPI_ISL_8132885			
see above	PHV-FSS	PHV-FSS	Chenwei Wang on behalf of Q-PHIRE Genomics
EPI_ISL_7834572	Palapye Primary Hospital Laboratory	Botswana Harvard HIV Reference Laboratory	Boitumelo Zuze; Botshelo Radibe; Dorcas Maruapula; Joseph Makhema; Keoratile Ntshambiwa; Kgomotso Moruisi; Legodile Kooepile; Mosepele Mosepele; Mphaphi B. Mbulawa; Ontlametse T. Bareng; Pamela Smith-Lawrence; Roger Shapiro; Sefetogi Ramaologa; Shahin Lockman; Sikhulile Moyo; Simani Gaseitsiwe; Thongbotho Mphoyakgosi; Wonderful T. Choga
EPI_ISL_7987968	Pathology North - Hunter - NSW Health Pathology	NSW Health Pathology - Institute of Clinical Pathology and Medical Research; Westmead Hospital; University of Sydney	Arnott A.; Draper J.; Gall M.; Martinez E.; Rockett R.; Sintchenko V.; on behalf of ICPMR
EPI_ISL_8124579	Plateforme de testing Namuroise	Plateforme de testing Namuroise	Degosserie Jonathan; Demars Aurore; Denis Olivier; Gilliard Nicolas; Maschietto Céline; Mullier François; Nobis Chloé; Otto Gaetan
EPI_ISL_8144416	Premier Laboratory Services	BioInfoExperts	Amy Feehan; Ben Lain; Chris Huston; David J. Nolan; Judy Crabtree; Julia-Garcia-Diaz; Lucio Miele; Rebecca Rose; Samual Moot; Susanna L. Lamers; Tessa LaFleur
EPI_ISL_7703322	Premier Laboratory Services	BioInfoExperts LLC	Cheryl Metzler; David Nolan; Rebecca Rose; Susanna Lamers; Valerie Murray
EPI_ISL_7345322, EPI_ISL_7464049, EPI_ISL_7464059, EPI_ISL_7509826, EPI_ISL_7509828, EPI_ISL_7509830, EPI_ISL_7563604, EPI_ISL_7583354, EPI_ISL_7583375, EPI_ISL_7583411, EPI_ISL_7685061, EPI_ISL_7725851, EPI_ISL_7725891, EPI_ISL_7725913, EPI_ISL_7725915, EPI_ISL_7725927, EPI_ISL_7843337, EPI_ISL_7843349, EPI_ISL_7843355, EPI_ISL_7992659, EPI_ISL_8090517			
see above	Respiratory Virus Unit, Microbiology Services Colindale, Public Health England	COVID-19 Genomics UK (COG-UK) Consortium	PHE Covid Sequencing Team
EPI_ISL_7862534	Rosalind Franklin Laboratory	Wellcome Sanger Institute for the COVID-19 Genomics UK (COG-UK) Consortium	Cordelia Langford; David K. Jackson; Dominic Kwiatkowski; Donald Fraser; Ewan Harrison; Ian Johnston; Jeffrey Barrett; John Sillitoe on behalf of the Wellcome Sanger Institute COVID-19 Surveillance Team; Rob Howes; Roberto Amato; Sonia Goncalves; Suki Lee; The Rosalind Franklin Laboratory and Alex Alderton
EPI_ISL_8112531, EPI_ISL_8112532, EPI_ISL_8112533, EPI_ISL_8112534, EPI_ISL_8112535, EPI_ISL_8112536, EPI_ISL_8112537, EPI_ISL_8112538, EPI_ISL_8112539, EPI_ISL_8112540, EPI_ISL_8112541, EPI_ISL_8112542, EPI_ISL_8112543, EPI_ISL_8112544, EPI_ISL_8112545, EPI_ISL_8112546, EPI_ISL_8112547, EPI_ISL_8112548, EPI_ISL_8112549, EPI_ISL_8112563			
see above	Servicio Microbiología Hospital La Paz	Servicio Microbiología Hospital La Paz	Elie Dahdouh; Fernando Lázaro; Iván Bloise; Jesús Mingorance Cruz; Rubén Cáceres
EPI_ISL_7625809, EPI_ISL_7896816, EPI_ISL_7896817, EPI_ISL_7896818, EPI_ISL_7896819, EPI_ISL_7896820, EPI_ISL_7896822, EPI_ISL_7896831			
see above	Servicio de Microbiologia Hospital Ramon y Cajal	Servicio de Microbiologia Hospital Ramon y Cajal	Galan JC; Martinez L. Abreu M; Ponce M; y Gonzalez-Alba JM
EPI_ISL_7632035, EPI_ISL_7632045, EPI_ISL_8020218, EPI_ISL_8020223, EPI_ISL_8020226, EPI_ISL_8119786, EPI_ISL_8119794, EPI_ISL_8119796, EPI_ISL_8119797, EPI_ISL_8119798, EPI_ISL_8119799, EPI_ISL_8119800			
see above	Stadtspital Triemli	Institute of Medical Virology, University of Zurich	Alexandra Trkola; Annette Audigé; Catharine Aquino; Cyril Shah; Daniel Ehrsam; Gabriela Ziltener; Guido Bloemberg; Hubert Rehrauer; Isabel Stürmer; Joel Wirz; Jon Huder; Jürg Böni; Kevin Steiner; Maria Grünberg; Maryam Zaheri; Michael Huber; Riccarda Capaul; Stefan Schmutz; Verena Kufner; Weihong Qi
EPI_ISL_7445855	State Hygienic Laboratory at the University of Iowa	State Hygienic Laboratory at the University of Iowa	Alankar Kampoowale; Anna Yakos; Cindy Toll; Davis Rieckenberg; Erik Twait; Jeff Benfer; Kris Eveland; Krishnaveni Sompallae; Kristen Zanon; Mariah Knutson; Mohammed Allam; Valerie Reeb; Wes Hottel
EPI_ISL_7621663, EPI_ISL_7621668	Sydney South West Pathology Service (SSWPS) - Liverpool Hospital - NSW Health Pathology	NSW Health Pathology - Institute of Clinical Pathology and Medical Research; Westmead Hospital; University of Sydney	Arnott A.; Draper J.; Gall M.; Martinez E.; Rockett R.; Sintchenko V.; on behalf of ICPMR
EPI_ISL_7830897, EPI_ISL_8128055, EPI_ISL_8131707, EPI_ISL_8131768, EPI_ISL_8131770, EPI_ISL_8131794, EPI_ISL_8131832, EPI_ISL_8131883			
see above	UW Virology Lab	UW Virology Lab	Alexander Greninger; Hong Xie; Isabel Arnould; Keith R Jerome; Meei-Li Huang; Nathan Breit; Patrick Mathias; Pavitra Roychoudhury; Pooneh Hajian; Robert J. Livingston; Sean Ellis; Seffir T. Wendm; Shah Mohamed Bakhash
EPI_ISL_7696303, EPI_ISL_7983388, EPI_ISL_7983537	University of Liège COVID-19 testing center	GIGA Medical Genomics	Bouchra Boujemla; Claire Gourzonès; Cécile Meex; Keith Durkin; Laurent Gillet; Maria Artesi; Marie-Pierre Hayette; Nadine Cambisano; Nathalie Renotte; Olivier Ek; Sébastien Bontems; Vincent Bours

EPI_ISL_8092797, EPI_ISL_8092799, EPI_ISL_8092800, EPI_ISL_8092807	University of Oregon COVID-19 MAP Laboratory	University of Oregon Genomics and Cell Charactericzation Core Facility (GC3F)	Ariana White; Demi Glidden; Douglas Turnbull; Jason Carriere; Jason Sydes; Jeff Bishop; Megan Criss; Peter Batzel; Tina Arredondo
EPI_ISL_8015470, EPI_ISL_8015502, EPI_ISL_8020107, EPI_ISL_8119785, EPI_ISL_8119790, EPI_ISL_8119793	Universität Zürich	Institute of Medical Virology, University of Zurich	Alexandra Trkola; Annette Audigé; Catharine Aquino; Cyril Shah; Daniel Ehrsam; Gabriela Ziltener; Guido Bloemberg; Hubert Rehrauer; Isabel Stürmer; Joel Wirz; Jon Huder; Jürg Böni; Kevin Steiner; Maria Grünberg; Maryam Zaheri; Michael Huber; Riccarda Capaul; Stefan Schmutz; Verena Kufner; Weihong Qi
EPI_ISL_8119773, EPI_ISL_8120214	Universitäts-Kinderspital Zürich	Institute of Medical Virology, University of Zurich	Alexandra Trkola; Annette Audigé; Catharine Aquino; Cyril Shah; Daniel Ehrsam; Gabriela Ziltener; Guido Bloemberg; Hubert Rehrauer; Isabel Stürmer; Joel Wirz; Jon Huder; Jürg Böni; Kevin Steiner; Maria Grünberg; Maryam Zaheri; Michael Huber; Riccarda Capaul; Stefan Schmutz; Verena Kufner; Weihong Qi
EPI_ISL_8015421, EPI_ISL_8015430, EPI_ISL_8015435, EPI_ISL_8015487, EPI_ISL_8015488, EPI_ISL_8015517, EPI_ISL_8020132, EPI_ISL_8020177, EPI_ISL_8020180, EPI_ISL_8020186, EPI_ISL_8020187, EPI_ISL_8020196, EPI_ISL_8120107, EPI_ISL_8120108, EPI_ISL_8120109, EPI_ISL_8120112, EPI_ISL_8120113, EPI_ISL_8120198, EPI_ISL_8120199, EPI_ISL_8120200, EPI_ISL_8120201, EPI_ISL_8120203, EPI_ISL_8120204, EPI_ISL_8120205, EPI_ISL_8120206, EPI_ISL_8120207, EPI_ISL_8120211, EPI_ISL_8120221, EPI_ISL_8120222, EPI_ISL_8120224, EPI_ISL_8120226			
see above	UniversitätsSpital Zürich	Institute of Medical Virology, University of Zurich	Alexandra Trkola; Annette Audigé; Catharine Aquino; Cyril Shah; Daniel Ehrsam; Gabriela Ziltener; Guido Bloemberg; Hubert Rehrauer; Isabel Stürmer; Joel Wirz; Jon Huder; Jürg Böni; Kevin Steiner; Maria Grünberg; Maryam Zaheri; Michael Huber; Riccarda Capaul; Stefan Schmutz; Verena Kufner; Weihong Qi
EPI_ISL_7566875	Viollier AG	Department of Biosystems Science and Engineering, ETH Zürich	Andrea Patrignani; Andrea Patrizia Salzmann; Andreia Cabral de Gouvea; Catharine Aquino Fournier; Chaoran Chen; Christian Urban; Christiane Beckmann; Christoph Noppen; Daniel Ehrsam; David Dreifuss; Doris Popovic; Franziska Singer; Griffin White; Hai Bui; Henriette Kurth; Isabel Stürmer; Ivan Topolsky; Jay Tracy; Kim Philipp Jablonski; Lara Fuhrmann; Laura Neff; Lennart Opitz; Louis du Plessis; Maria Domenica Moccia; Matteo Carrara; Maurice Redondo; Niko Beerenwinkel; Olivier Kobel; Pelin Icer; Ralph Schlapbach; Sarah Nadeau; Shuqing Yu; Simon Grüter; Tanja Stadler; Timothy Sykes
EPI_ISL_7285845, EPI_ISL_7285846	Virology Laboratory, Scientific Department, Army Medical Center	Virology Laboratory, Scientific Department, Army Medical Center	Anella Monte; Anna Anselmo; Antonella Fortunato; Filippo Molinari; Florigio Lista; Francesco Giordani; Giancarlo Petralito; Giandomenico Cerreto; Giulia Campoli; Lucia Nicosia; Marzia Cavalli; Pietro Marco D'Angelo; Riccardo De Sanctis; Rossella Brandi; Silvia Fillo; Vanessa Vera Fain
EPI_ISL_8128502	ZARV/NHLS, Department Medical Virology, University of Pretoria	CERI, Centre for Epidemic Response and Innvoation, Stellenbosch University and KRISP, KZN Research Innovation and Sequencing Platform, UKZN.	Adriano Mendes; Amy Strydom; Arisha Maharaj; Giandhari J; Micheala Davids; Naidoo Y; Pillay S; Ramphal U; Ramphal Y; San JE; Sim Mayaphi and Marietjie Venter; Tegally H; Tshiabuila D; Wilkinson E; de Oliveira T
EPI_ISL_8020197	Zentrallabor Zürich	Institute of Medical Virology, University of Zurich	Alexandra Trkola; Annette Audigé; Catharine Aquino; Cyril Shah; Daniel Ehrsam; Gabriela Ziltener; Guido Bloemberg; Hubert Rehrauer; Isabel Stürmer; Joel Wirz; Jon Huder; Jürg Böni; Kevin Steiner; Maria Grünberg; Maryam Zaheri; Michael Huber; Riccarda Capaul; Stefan Schmutz; Verena Kufner; Weihong Qi

We gratefully acknowledge the following Authors from the Originating laboratories responsible for obtaining the specimens, as well as the Submitting laboratories where the genome data were generated and shared via GISAID, on which this research is based.

All Submitters of data may be contacted directly via www.gisaid.org

Authors are sorted alphabetically.

Accession ID	Originating Laboratory	Submitting Laboratory	Authors
EPI_ISL_8285616	ADUGODI DISPENSARY	National Center for Biological Sciences, TIFR - Rockefeller Foundation	Awadhesh Pandit; Bhagyashree Madhav Shelar; Darshan Sreenivas; Dimple Notani; Lakshminarayanan CP; Manisha Bharadwaj; Satyajit Mayor; Uma Ramakrishnan
EPI_ISL_8307826, EPI_ISL_8308252, EPI_ISL_8311241, EPI_ISL_8311243	Aegis Sciences Corporation	Centers for Disease Control and Prevention Division of Viral Diseases, Pathogen Discovery	Alec Vest; Benjamin Rambo-Martin; Christopher Gulvick; Clinton Paden; Cyndi Clark; Dakota Howard; Dhwani Batra; Dillon Nall; Duncan MacCannell; Erisa Sula; Ethan Sanders; Holly Houdeshell; Jason Caravas; Kristine Lacek; Matthew Hardison; Matthew Schmerer; Ola Kvalvaag; Patrick Campbell; Peter Cook; Rob Case; Scott Sammons; Shatavia Morrison; Shaun Westlund; Tymeckia Kendall; Victoria Caban Figueroa; Vikramsinha Ghorpade; Yvette Unoarumhi
EPI_ISL_8281485, EPI_ISL_8281542, EPI_ISL_8281566	Allergy, Immunology and Cell Biology Unit (AICBU)	Allergy, Immunology and Cell Biology Unit (AICBU)	Ayesha Wijesinghe; Chandima Jeewandara; Deshni Jayathilaka; Dinuka Ariyaratne; Diyanath Ranasinghe; Dumni Guasinghe; Farha Bary; Gathsaurie Neelika Malavige; Heshan Kuruppu; Tibutius Thanesh
EPI_ISL_8274562	Berkshire and Surrey Pathology Services Lighthouse Laboratory	Wellcome Sanger Institute for the COVID-19 Genomics UK (COG-UK) Consortium	Berkshire and Surrey Pathology Services Lighthouse Laboratory and Alex Alderton; Cordelia Langford; David K. Jackson; Dominic Kwiatkowski; Ewan Harrison; Ian Johnston; Jeffrey Barrett; John Sillitoe on behalf of the Wellcome Sanger Institute COVID-19 Surveillance Team; Roberto Amato; Sonia Goncalves
EPI_ISL_8312616	BioneXt Lab	Laboratoire national de sante, Microbiology, Microbial Genomics Platform	Anke Wienecke-Baldacchino; Catherine Ragimbeau; Elodie Solarino; Fatu Djabi; Jessica Tapp; Lise Pignon; Raoul Salmon; Sibel Berger; Tamir Abdelrahman; Thibault Ferrandon; Virginie Jover
EPI_ISL_8294239	CHTMAD	Instituto Nacional de Saude (INSA)	Borges et al
EPI_ISL_8314702	CHU Purpan - Laboratoire de Virologie - Institut Federatif de Biologie	CHU Purpan - Laboratoire de Virologie - Institut Fédératif de Biologie	Boyer P.; Carcenac R.; Ferrer V.; Harter A.; Izopet J.; Jeanne N.; Latour J.; Ranger N.; Tremeaux P.
EPI_ISL_8206925, EPI_ISL_8206972	Clinical Microbiology Laboratory, Tel Aviv Sourasky Medical Center	Clinical Microbiology Laboratory, Tel Aviv Sourasky Medical Center	Alon Ziv; Amos Adler; Katya Levytskyi; Lior Handler; Ora Halutz
EPI_ISL_8317093	Cliniques universitaires Saint-Luc	UCLouvain/IREC/MBLG-CTMA	Benoit Kabamba Mukadi; Bertrand Bearzatto; Jean-Luc Gala; Nicolas Pinte; Paul Blanpain; Simon Ophélie; Valentin Coste
EPI_ISL_8158100, EPI_ISL_8158748, EPI_ISL_8159764, EPI_ISL_8186098, EPI_ISL_8186108	Cytocheck Laboratory LLC	MAWD Pathology Group, PA	Kelli Fayard; Matison Lewis; Pradip Manna PhD; Shelby Herner; Stephanie Romanowicz
EPI_ISL_8170859, EPI_ISL_8170887, EPI_ISL_8170916, EPI_ISL_8170947, EPI_ISL_8171081, EPI_ISL_8171085, EPI_ISL_8171165, EPI_ISL_8171189, EPI_ISL_8171210, EPI_ISL_8171306, EPI_ISL_8171336, EPI_ISL_8171472, EPI_ISL_8171474, EPI_ISL_8171498, EPI_ISL_8171700, EPI_ISL_8171712, EPI_ISL_8171770, EPI_ISL_8172004, EPI_ISL_8172098, EPI_ISL_8172115, EPI_ISL_8172152, EPI_ISL_8172167, EPI_ISL_8172173, EPI_ISL_8172318, EPI_ISL_8200536, EPI_ISL_8200559, EPI_ISL_8200570, EPI_ISL_8200585, EPI_ISL_8200606, EPI_ISL_8200608, EPI_ISL_8200613, EPI_ISL_8200650, EPI_ISL_8200658, EPI_ISL_8200697, EPI_ISL_8200709, EPI_ISL_8200746, EPI_ISL_8200758, EPI_ISL_8200789, EPI_ISL_8200795, EPI_ISL_8200885, EPI_ISL_8200913, EPI_ISL_8200918, EPI_ISL_8200964, EPI_ISL_8201020, EPI_ISL_8201041, EPI_ISL_8201176, EPI_ISL_8201193, EPI_ISL_8201235, EPI_ISL_8201238, EPI_ISL_8201246, EPI_ISL_8201249, EPI_ISL_8201252, EPI_ISL_8201264, EPI_ISL_8201269, EPI_ISL_8201288, EPI_ISL_8201357, EPI_ISL_8201360, EPI_ISL_8201379, EPI_ISL_8201398, EPI_ISL_8201408, EPI_ISL_8201447, EPI_ISL_8201501, EPI_ISL_8201529, EPI_ISL_8201543, EPI_ISL_8201573, EPI_ISL_8201574, EPI_ISL_8201590, EPI_ISL_8201598, EPI_ISL_8201724, EPI_ISL_8201727, EPI_ISL_8201760, EPI_ISL_8201763, EPI_ISL_8201766, EPI_ISL_8201769, EPI_ISL_8201791, EPI_ISL_8201803, EPI_ISL_8201812, EPI_ISL_8201825, EPI_ISL_8201833, EPI_ISL_8201850, EPI_ISL_8201869, EPI_ISL_8201946, EPI_ISL_8201960, EPI_ISL_8202006, EPI_ISL_8202023, EPI_ISL_8202121, EPI_ISL_8202132, EPI_ISL_8202141, EPI_ISL_8202166, EPI_ISL_8202174, EPI_ISL_8202227, EPI_ISL_8202240, EPI_ISL_8202287, EPI_ISL_8202349, EPI_ISL_8202351, EPI_ISL_8202357, EPI_ISL_8202422, EPI_ISL_8202430, EPI_ISL_8202434, EPI_ISL_8202435, EPI_ISL_8202451, EPI_ISL_8202478, EPI_ISL_8202510, EPI_ISL_8202512, EPI_ISL_8202549, EPI_ISL_8202567, EPI_ISL_8202574, EPI_ISL_8202626, EPI_ISL_8202846, EPI_ISL_8202860, EPI_ISL_8202872, EPI_ISL_8202885, EPI_ISL_8202912, EPI_ISL_8202953, EPI_ISL_8249423, EPI_ISL_8249427, EPI_ISL_8249534, EPI_ISL_8249540, EPI_ISL_8249544, EPI_ISL_8249572, EPI_ISL_8249603, EPI_ISL_8249605, EPI_ISL_8249624, EPI_ISL_8249716, EPI_ISL_8249719, EPI_ISL_8249725, EPI_ISL_8249734, EPI_ISL_8249752, EPI_ISL_8249798, EPI_ISL_8249805, EPI_ISL_8249888, EPI_ISL_8249938, EPI_ISL_8249939, EPI_ISL_8249969, EPI_ISL_8249990, EPI_ISL_8249998, EPI_ISL_8250001, EPI_ISL_8250093, EPI_ISL_8250094, EPI_ISL_8250108, EPI_ISL_8250147, EPI_ISL_8250159, EPI_ISL_8250179, EPI_ISL_8250181, EPI_ISL_8250225, EPI_ISL_8250226, EPI_ISL_8250268, EPI_ISL_8250293, EPI_ISL_8250311, EPI_ISL_8250321, EPI_ISL_8250357, EPI_ISL_8250510, EPI_ISL_8250664, EPI_ISL_8250687, EPI_ISL_8250703, EPI_ISL_8250795, EPI_ISL_8250798, EPI_ISL_8250799, EPI_ISL_8250824, EPI_ISL_8250834, EPI_ISL_8250837, EPI_ISL_8250909, EPI_ISL_8250982, EPI_ISL_8250999, EPI_ISL_8251013, EPI_ISL_8251017, EPI_ISL_8251029, EPI_ISL_8251592, EPI_ISL_8251603, EPI_ISL_8251634, EPI_ISL_8251644, EPI_ISL_8251679			
see above	Department of Bacteria, Parasites and Fungi, Statens Serum Institut, Copenhagen, Denmark	Statens Serum Institut Bioinformatics and Microbial Genomics	Danish Covid-19 Genome Consortium
EPI_ISL_8185870	Department of Clinical Microbiology	GIGA Medical Genomics	Bouchra Boujemla; Claire Gourzonès; Cécile Meex; Keith Durkin; Laurent Gillet; Maria Artesi; Marie-Pierre Hayette; Nadine Cambisano; Nathalie Renotte; Olivier Ek; Sébastien Bontems; Vincent Bours
EPI_ISL_8183263, EPI_ISL_8290748	Fulgent Genetics	Centers for Disease Control and Prevention Division of Viral Diseases, Pathogen Discovery	Becky Tsai; Benafsh Sapra; Benjamin Rambo-Martin; Christopher Gulvick; Clinton Paden; Dakota Howard; Dhwani Batra; Doreen Ng; Duncan MacCannell; Erisa Sula; Harry Gao; James Xie; Jason Caravas; John Gao; Joseph Fierro; Kristine Lacek; Matthew Schmerer; Mickey Li; Peter Cook; Scott Sammons; Shatavia Morrison; Tymeckia Kendall; Victoria Caban Figueroa; Yan Meng; Yvette Unoarumhi
EPI_ISL_8260969, EPI_ISL_8261006, EPI_ISL_8261017, EPI_ISL_8261025, EPI_ISL_8261030, EPI_ISL_8261144, EPI_ISL_8261145, EPI_ISL_8261157, EPI_ISL_8261159, EPI_ISL_8261164, EPI_ISL_8261170, EPI_ISL_8261175, EPI_ISL_8261183, EPI_ISL_8261188, EPI_ISL_8261206, EPI_ISL_8261210, EPI_ISL_8261294, EPI_ISL_8261300			
see above	Infinity Biologix	Centers for Disease Control and Prevention Division of Viral Diseases, Pathogen Discovery	Benjamin Rambo-Martin; Chirayu Goswami; Christian Bixby; Christopher Gulvick; Clinton Paden; Dakota Howard; Dhwani Batra; Duncan MacCannell; Erisa Sula; Jason Caravas; Jonathan Schultz; Kristine Lacek; Matthew Schmerer; Peter Cook; Robin Grimwood; Russ Hager; Scott Sammons; Shatavia Morrison; Tymeckia Kendall; Victoria Caban Figueroa; Yihe Wang; Yvette Unoarumhi
EPI_ISL_8159888	Johns Hopkins Hospital Department of Pathology	Johns Hopkins Hospital Department of Pathology	Amary Fall; C. Paul Morris; David Gaston; Heba H. Mostafa; Julie M. Norton; Matthew Schwartz; Michael Forman; Raghda Eldesouki
EPI_ISL_8183503, EPI_ISL_8184726, EPI_ISL_8184728, EPI_ISL_8317248, EPI_ISL_8317271	LABOPAT	LABOPAT	Cynthia Penaloza; Luis Mendoza; Silvia Montilla
EPI_ISL_8222049	Labor Dr. Wisplinghoff - Köln	Robert Koch Institute	
EPI_ISL_8182911	Laboratory for Molecular Diagnostics,IPHMN	Laboratory for Molecular Diagnostics,IPHMN	Danijela Vujošević; Marija Govedarica; Rejhan Hot
EPI_ISL_8274098, EPI_ISL_8275396	Lighthouse Lab in Alderley Park	Wellcome Sanger Institute for the COVID-19 Genomics UK (COG-UK) Consortium	Cordelia Langford; David K. Jackson; Dominic Kwiatkowski; Ewan Harrison; Ian Johnston; Jacquelyn Wynn; Jeffrey Barrett; John Sillitoe on behalf of the Wellcome Sanger Institute COVID-19 Surveillance Team; Mairead Hyland; Roberto Amato; Sonia Goncalves; The Lighthouse Lab in Alderley Park and Alex Alderton
EPI_ISL_8276469, EPI_ISL_8280437, EPI_ISL_8280529	Lighthouse Lab in Glasgow	Wellcome Sanger Institute for the COVID-19 Genomics UK (COG-UK) Consortium	Anna Dominiczak and Alex Alderton; Carol Clugston; Cordelia Langford; David Gray; David K. Jackson; Dominic Kwiatkowski; Ewan Harrison; Harper VanSteenhouse; Ian Johnston; Jeffrey Barrett; John Sillitoe on behalf of the Wellcome Sanger Institute COVID-19 Surveillance Team; Roberto Amato; Sonia Goncalves; Yumi Kasai
EPI_ISL_8272463, EPI_ISL_8273411, EPI_ISL_8281329	Lighthouse Lab in Milton Keynes	Wellcome Sanger Institute for the COVID-19 Genomics UK (COG-UK) Consortium	Cordelia Langford; David K. Jackson; Dominic Kwiatkowski; Ewan Harrison; Ian Johnston; Jeffrey Barrett; John Sillitoe on behalf of the Wellcome Sanger Institute COVID-19 Surveillance Team; Roberto Amato; Sonia Goncalves; The Lighthouse Lab in Milton Keynes and Alex Alderton
EPI_ISL_8280527	Lighthouse Laboratory Plymouth	Wellcome Sanger Institute for the COVID-19 Genomics UK (COG-UK) Consortium	Cordelia Langford; David K. Jackson; Dominic Kwiatkowski; Ewan Harrison; Ian Johnston; Jeffrey Barrett; John Sillitoe on behalf of the Wellcome Sanger Institute COVID-19 Surveillance Team; Lighthouse Laboratory Plymouth and Alex Alderton; Roberto Amato; Sonia Goncalves
EPI_ISL_8182744, EPI_ISL_8186596, EPI_ISL_8186689	MAWD Pathology Group, PA	MAWD Pathology Group, PA	Kelli Fayard; Kelsi Eastman; Matison Lewis; Pradip Manna PhD; Shelby Herner; Stephanie Romanowicz
EPI_ISL_8285588	MEDALL CLUMAX DIAGNOSTICS/BBMP	National Center for Biological Sciences, TIFR - Rockefeller Foundation	Awadhesh Pandit; Bhagyashree Madhav Shelar; Darshan Sreenivas; Dimple Notani; Lakshminarayanan CP; Manisha Bharadwaj; Satyajit Mayor; Uma Ramakrishnan
EPI_ISL_8307323, EPI_ISL_8307325, EPI_ISL_8307332	MRC/UVRI & LSHTM Uganda Research Unit, Uganda Virus Research Institute	MRC/UVRI & LSHTM Uganda Research Unit, Uganda Virus Research Institute	Alfred Ssekagiri; Deogratius Ssemwanga; Hamidah Namagembe; Henry Kyobe-Bbosa; Isaac Ssewanyana; Jocelyn Kiconco; John Kayiwa; Julius Lutwama; Nicholas Bbosa; Pontiano Kaleebu; Ronald Kiiza; Susan Nabadda; Tom Lutalo
EPI_ISL_8309860, EPI_ISL_8309978	Mako Medical	Centers for Disease Control and Prevention Division of Viral Diseases, Pathogen Discovery	Benjamin Rambo-Martin; Christopher Gulvick; Clinton Paden; Dakota Howard; Dhwani Batra; Duncan MacCannell; Erisa Sula; Jason Caravas; Kristine Lacek; Lauren Moon; Matthew Schmerer; Matthew Tugwell; Peter Cook; Scott Sammons; Shatavia Morrison; Tymeckia Kendall; Victoria Caban Figueroa; Yvette Unoarumhi
EPI_ISL_8229937, EPI_ISL_8229951, EPI_ISL_8229963, EPI_ISL_8229982, EPI_ISL_8229990, EPI_ISL_8229996, EPI_ISL_8249273, EPI_ISL_8249298			
see above	Medizinisch-Diagnostisches Labor Kempten allgäulab	Robert Koch Institute	
EPI_ISL_8308168, EPI_ISL_8308189, EPI_ISL_8308194	Michigan Department of Health and Human Services, Bureau of Laboratories	Michigan Department of Health and Human Services, Bureau of Laboratories	Blankenship HM; Riner D; Soehnlen MK
EPI_ISL_8188943	Middlemore Hospital	Institute of Environmental Science and Research (ESR)	Anja Werno; Antje van der Linden; Arlo Upton; Chris Mansell; Clare Gebbie; David Hammer; Dhanisha Patel; Dragana Drinkovic; Erasmus Smit; Gary McAuliffe; Hana Sofia Andersson; Hermes Perez; James Ussher; Jill Sherwood; Jing Wang; Joep de Ligt; Josh Freeman; Julia Howard; Juliet Elvy; Lauren Jelly; Mary DeAlmeida; Matt Blakiston; Matt Storey; Matthew Rogers; Max Bloomfield; Michael Addidle; Michelle Balm; Muhammad Faisal; Nikki Freed; Olin Silander; Olivia Stroeven; Rachel Boyle; Sally Roberts; SallyAnn Harbison; Sarah Cockerton; Sarah Jefferies; Sharmini Muttaiyah; Susan Morpeth; Susan Taylor; Timothy Blackmore; Vani Sathyendran; Veronica Playle; Virginia Hope; Xiaoyun Ren
EPI_ISL_8164601, EPI_ISL_8164656,	Ministry of Health Turkey	Ministry of Health Turkey	Fatma Bayrakdar; Gülay Korukluoğlu; Süleyman Yalcin; Yasemin Coşgun

EPI_ISL_8164657			
EPI_ISL_8172051, EPI_ISL_8288172	Originating lab: Wales Specialist Virology Centre Sequencing lab: Pathogen Genomics Unit	Public Health Wales Microbiology Cardiff Wales Specialist Virology Centre	Alec Birchley; Alexander Adams; Amy Gaskin; Angela Marchbank; Bree Gatica-Wilcox; Catherine Moore; Jason Coombes; Joanne Watkins; Joel Southgate; Johnathan Evans; Laura Gifford; Lauren Gilbert; Lee Graham; Malorie Perry; Matthew Bull; Nicole Pacchiarini; Sally Corden; Sara Kumziene-Summerhayes; Sara Rey; Sarah Taylor; Simon Cottrell; Sophie Jones; Tom Connor
EPI_ISL_8242901, EPI_ISL_8242906, EPI_ISL_8287185, EPI_ISL_8287224	Respiratory Virus Unit, Microbiology Services Colindale, Public Health England	COVID-19 Genomics UK (COG-UK) Consortium	PHE Covid Sequencing Team
EPI_ISL_8185869	SYNLAB	GIGA Medical Genomics	Bouchra Boujemla; Claire Gourzonès; Cécile Meex; Keith Durkin; Laurent Gillet; Maria Artesi; Marie-Pierre Hayette; Nadine Cambisano; Nathalie Renotte; Olivier Ek; Sébastien Bontems; Vincent Bours
EPI_ISL_8185077	Sema4	Sema4	Angela Yannes; Bisram Deocharan; Feras Hantash; Matthew Capozziello; Rui Gu; Victoria Parkington; Zdenek Markovic
EPI_ISL_8294887, EPI_ISL_8318769	Tulane University School of Medicine	Tulane University School of Medicine	Di Tian
EPI_ISL_8163266, EPI_ISL_8163275, EPI_ISL_8163276, EPI_ISL_8163281, EPI_ISL_8163282, EPI_ISL_8163300, EPI_ISL_8163304, EPI_ISL_8163321, EPI_ISL_8163341, EPI_ISL_8163366			
see above	UW Virology Lab	UW Virology Lab	Alexander Greninger; Hong Xie; Isabel Arnould; Keith R Jerome; Meei-Li Huang; Nathan Breit; Patrick Mathias; Pavitra Roychoudhury; Pooneh Hajian; Robert J. Livingston; Sean Ellis; Seffir T. Wendm; Shah Mohamed Bakhash

We gratefully acknowledge the following Authors from the Originating laboratories responsible for obtaining the specimens, as well as the Submitting laboratories where the genome data were generated and shared via GISAID, on which this research is based.

All Submitters of data may be contacted directly via www.gisaid.org

Authors are sorted alphabetically.

Accession ID	Originating Laboratory	Submitting Laboratory	Authors
EPI_ISL_8352208, EPI_ISL_8356546, EPI_ISL_8356663, EPI_ISL_8358068, EPI_ISL_8370307, EPI_ISL_8394586, EPI_ISL_8395229			
see above	Aegis Sciences Corporation	Centers for Disease Control and Prevention Division of Viral Diseases, Pathogen Discovery	Alec Vest; Benjamin Rambo-Martin; Christopher Gulvick; Clinton Paden; Cyndi Clark; Dakota Howard; Dhwani Batra; Dillon Nall; Duncan MacCannell; Erisa Sula; Ethan Sanders; Holly Houdeshell; Jason Caravas; Kristine Lacek; Matthew Hardison; Matthew Schmerer; Ola Kvalvaag; Patrick Campbell; Peter Cook; Rob Case; Scott Sammons; Shatavia Morrison; Shaun Westlund; Tymeckia Kendall; Victoria Caban Figueroa; Vikramsinha Ghorpade; Yvette Unoarumhi
EPI_ISL_8338494	Azienda Ospedaliero - Universitaria di Modena Policlinico - Virologia e Microbiologia Molecolare""	Istituto Zooprofilattico Sperimentale della Lombardia e dell'Emilia Romagna (IZSLER), Risk Analysis and Genomic Epidemiology Unit	Erika Scaltriti; Ilaria Menozzi; Marina Morganti; Stefano Pongolini
EPI_ISL_8379784	Bayerisches Landesamt fuer Gesundheit und Lebensmittelsicherheit (LGL)	Bayerisches Landesamt fuer Gesundheit und Lebensmittelsicherheit (LGL)	Alexandra Dangel; Andreas Sing; Annika Sprenger; Carola Berger; Laura Weise; Nikolaus Ackermann; Sabrina Hepner
EPI_ISL_8354549	CDPH VBL	California Department of Public Health	CDPH COVIDNet
EPI_ISL_8401203	CHU UCL Namur, Avenue G. Thérasse 1, 5530 Yvoir, Belgium	Plateforme de testing Namuroise	Degosserie Jonathan; Demars Aurore; Denis Olivier; Laurent Hélène; Maschietto Céline; Mullier François; Nobis Chloé; Otto Gaetan
EPI_ISL_8378658	Clinical Virology	Clinical Bacteriology, University Hospital Basel	Adrian Egli; Alfredo Mari; Fanny Wegner; Hans Hirsch; Helena MB Seth-Smith; Julia Bielicki; Karoline Leuzinger; Manuel Battegay; Tim Roloff
EPI_ISL_8378837	Colorado Department of Public Health and Environment	Colorado Department of Public Health and Environment	Alexandria Rossheim; Arianna Smith; Diana Ir; Emily A. Travanty; Laura Bankers; Mandy Waters; Michael Martin; Molly C. Hetherington-Rauth; Shannon R. Matzinger
EPI_ISL_8344062, EPI_ISL_8344068, EPI_ISL_8344077, EPI_ISL_8344098, EPI_ISL_8344151, EPI_ISL_8344154, EPI_ISL_8344171, EPI_ISL_8344172, EPI_ISL_8344182, EPI_ISL_8344200, EPI_ISL_8344204, EPI_ISL_8344207, EPI_ISL_8344226, EPI_ISL_8344242, EPI_ISL_8344272, EPI_ISL_8344294, EPI_ISL_8344329, EPI_ISL_8344341, EPI_ISL_8344346, EPI_ISL_8344368, EPI_ISL_8344380, EPI_ISL_8344397, EPI_ISL_8344436, EPI_ISL_8344440, EPI_ISL_8344449, EPI_ISL_8344458, EPI_ISL_8344480, EPI_ISL_8344489, EPI_ISL_8344493, EPI_ISL_8344523, EPI_ISL_8344526, EPI_ISL_8344527, EPI_ISL_8344534, EPI_ISL_8344562, EPI_ISL_8344564, EPI_ISL_8344660, EPI_ISL_8344692, EPI_ISL_8344697, EPI_ISL_8344700, EPI_ISL_8344739, EPI_ISL_8344744, EPI_ISL_8344745, EPI_ISL_8344755, EPI_ISL_8344769, EPI_ISL_8344781, EPI_ISL_8344897, EPI_ISL_8344903, EPI_ISL_8344936, EPI_ISL_8344957, EPI_ISL_8344963, EPI_ISL_8344992, EPI_ISL_8345013, EPI_ISL_8345024, EPI_ISL_8345047, EPI_ISL_8345186, EPI_ISL_8345207, EPI_ISL_8345214, EPI_ISL_8345232, EPI_ISL_8345234, EPI_ISL_8345237, EPI_ISL_8345254, EPI_ISL_8345258, EPI_ISL_8345311, EPI_ISL_8345319, EPI_ISL_8345328, EPI_ISL_8345347, EPI_ISL_8345432, EPI_ISL_8345514, EPI_ISL_8345533, EPI_ISL_8345538, EPI_ISL_8345568, EPI_ISL_8345599, EPI_ISL_8345627, EPI_ISL_8345637, EPI_ISL_8345653, EPI_ISL_8345772, EPI_ISL_8345775, EPI_ISL_8345802, EPI_ISL_8345854, EPI_ISL_8345914, EPI_ISL_8345921, EPI_ISL_8345923, EPI_ISL_8345928, EPI_ISL_8345930, EPI_ISL_8345934, EPI_ISL_8345977, EPI_ISL_8345990, EPI_ISL_8345999, EPI_ISL_8346020, EPI_ISL_8346023, EPI_ISL_8346030, EPI_ISL_8346041, EPI_ISL_8346097, EPI_ISL_8346102, EPI_ISL_8346126, EPI_ISL_8346204, EPI_ISL_8346206, EPI_ISL_8346273, EPI_ISL_8346283, EPI_ISL_8346317, EPI_ISL_8346348, EPI_ISL_8346360, EPI_ISL_8346370, EPI_ISL_8346381, EPI_ISL_8346416, EPI_ISL_8346462, EPI_ISL_8346477, EPI_ISL_8346478, EPI_ISL_8346511, EPI_ISL_8346598, EPI_ISL_8346604, EPI_ISL_8346607, EPI_ISL_8346620, EPI_ISL_8346627, EPI_ISL_8346645, EPI_ISL_8346646, EPI_ISL_8346685, EPI_ISL_8346687, EPI_ISL_8346719, EPI_ISL_8346749, EPI_ISL_8346768, EPI_ISL_8346770, EPI_ISL_8346780, EPI_ISL_8346782, EPI_ISL_8346786, EPI_ISL_8346812, EPI_ISL_8346821, EPI_ISL_8346824, EPI_ISL_8346856, EPI_ISL_8346862, EPI_ISL_8346866, EPI_ISL_8346885, EPI_ISL_8346890, EPI_ISL_8346899, EPI_ISL_8346912, EPI_ISL_8346921, EPI_ISL_8346927, EPI_ISL_8346937, EPI_ISL_8346951, EPI_ISL_8347027, EPI_ISL_8347055, EPI_ISL_8347064, EPI_ISL_8347066, EPI_ISL_8347087, EPI_ISL_8347106, EPI_ISL_8347137, EPI_ISL_8347147, EPI_ISL_8347179, EPI_ISL_8347187, EPI_ISL_8347204, EPI_ISL_8347241, EPI_ISL_8347351, EPI_ISL_8347371, EPI_ISL_8347392, EPI_ISL_8347396, EPI_ISL_8347398, EPI_ISL_8347427, EPI_ISL_8347442, EPI_ISL_8347446, EPI_ISL_8347448, EPI_ISL_8347467, EPI_ISL_8347476, EPI_ISL_8347521, EPI_ISL_8347538, EPI_ISL_8347706, EPI_ISL_8347713, EPI_ISL_8381420, EPI_ISL_8381436, EPI_ISL_8381456, EPI_ISL_8381463, EPI_ISL_8381467, EPI_ISL_8381480, EPI_ISL_8381494, EPI_ISL_8381504, EPI_ISL_8381506, EPI_ISL_8381509, EPI_ISL_8381574, EPI_ISL_8381581, EPI_ISL_8381589, EPI_ISL_8381621, EPI_ISL_8381642, EPI_ISL_8381643, EPI_ISL_8381652, EPI_ISL_8381670, EPI_ISL_8381677, EPI_ISL_8381685, EPI_ISL_8381690, EPI_ISL_8381703, EPI_ISL_8381713, EPI_ISL_8381722, EPI_ISL_8382103, EPI_ISL_8382106, EPI_ISL_8382114, EPI_ISL_8382128, EPI_ISL_8382131, EPI_ISL_8382132, EPI_ISL_8382143, EPI_ISL_8382148, EPI_ISL_8382173, EPI_ISL_8382180, EPI_ISL_8382192, EPI_ISL_8382196, EPI_ISL_8382215, EPI_ISL_8382247, EPI_ISL_8382252, EPI_ISL_8382253, EPI_ISL_8382261, EPI_ISL_8382277, EPI_ISL_8382285, EPI_ISL_8382314, EPI_ISL_8382329, EPI_ISL_8382350, EPI_ISL_8382354, EPI_ISL_8382359, EPI_ISL_8382383, EPI_ISL_8382394, EPI_ISL_8382415, EPI_ISL_8382434, EPI_ISL_8382441, EPI_ISL_8382451, EPI_ISL_8382463, EPI_ISL_8382464, EPI_ISL_8382467, EPI_ISL_8382490, EPI_ISL_8382504, EPI_ISL_8382509, EPI_ISL_8382523, EPI_ISL_8382555, EPI_ISL_8382565, EPI_ISL_8382577, EPI_ISL_8382589, EPI_ISL_8382600, EPI_ISL_8382604, EPI_ISL_8382635, EPI_ISL_8382639, EPI_ISL_8382649, EPI_ISL_8382650, EPI_ISL_8382661, EPI_ISL_8382716, EPI_ISL_8382745, EPI_ISL_8382757, EPI_ISL_8382784, EPI_ISL_8382791, EPI_ISL_8382794, EPI_ISL_8382800, EPI_ISL_8382802, EPI_ISL_8382811, EPI_ISL_8382820, EPI_ISL_8382823, EPI_ISL_8382829, EPI_ISL_8382834, EPI_ISL_8382840			
see above	Department of Bacteria, Parasites and Fungi, Statens Serum Institut, Copenhagen, Denmark	Statens Serum Institut Bioinformatics and Microbial Genomics	Danish Covid-19 Genome Consortium
EPI_ISL_8380408, EPI_ISL_8380410	Gandhi Medical College and Hospital (GMCH), Secunderabad	NIV Influenza	D.R.Manisha Rani; Dr.G.Sushma Rajya Lakshmi; Dr.K.Nagamani; Dr.Sunitha Pakalapaty
EPI_ISL_8329270, EPI_ISL_8329272, EPI_ISL_8329274, EPI_ISL_8329277, EPI_ISL_8329279, EPI_ISL_8329280, EPI_ISL_8329282, EPI_ISL_8329286, EPI_ISL_8329292, EPI_ISL_8329293, EPI_ISL_8329294, EPI_ISL_8329298, EPI_ISL_8329300, EPI_ISL_8329303, EPI_ISL_8329304, EPI_ISL_8329305, EPI_ISL_8329306, EPI_ISL_8329307, EPI_ISL_8329308, EPI_ISL_8329310, EPI_ISL_8329313, EPI_ISL_8329316, EPI_ISL_8329319, EPI_ISL_8329323, EPI_ISL_8329325, EPI_ISL_8329328, EPI_ISL_8329332, EPI_ISL_8329336, EPI_ISL_8329342, EPI_ISL_8329343, EPI_ISL_8329345, EPI_ISL_8329348, EPI_ISL_8329349, EPI_ISL_8329350, EPI_ISL_8329351, EPI_ISL_8329352, EPI_ISL_8329353, EPI_ISL_8329354, EPI_ISL_8329357			
see above	Idaho Bureau of Laboratories	Idaho Bureau of Laboratories	Aimee Ceniseros; Christian Loera; Christopher Ball; Matthew Charles Burns; R. Beukelman; Robert L. Voermans
EPI_ISL_8364636, EPI_ISL_8365040	Laboratory Corporation of America	Centers for Disease Control and Prevention Division of Viral Diseases, Pathogen Discovery	Amanda Douglas; Amanda Suchanek; Andrea Throop; Ayla Burns; Benjamin Rambo-Martin; Bobbi Croy; Brian Krueger; Brian Norvell; Christopher Gulvick; Christos Petropoulos; Clinton Paden; Craig Lukasik; Dakota Howard; Debbie Boles; Dhwani Batra; Duncan MacCannell; Eyad Almasri; Goran Stevovic; Howard Engler; Hrushikesh Deshmukh; Jake Humphrey; Jana Schroth; Jason Caravas; Joe Voshell; John Pruitt; Jonathan Meltzer; Jonathan Williams; Kimberly Wagner; Kristine Lacek; Lax Iyer; Lisa Pfefferle; Lyndon Tilson; Manoj Jain; Marcia Eisenberg; Mary Cristobal; Mary Williamson; Matthew Robinson; Matthew Schmerer; Michael Levandoski; Mike Sapeta; Mindy Nye; Minoo Agarwal; Mohan Kolli; Nuthawin Charoensri; Oren Cohen; Peter Cook; Prashant Gupta; Qian Zeng; Rama Ghatti; Scott Parker; Scott Ryan; Scott Sammons; Shatavia Morrison; Stanley Letovsky; Steven Ragan; Suresh Selvaraju; Susan Countryman; Susan Hicks; Suzanne Dale; Thomas Urban; Tim Kuphal; Tricia Zwiefelhofer; Tymeckia Kendall; Victoria Caban Figueroa; Vincent Drouillon; Yvette Unoarumhi
EPI_ISL_8371879, EPI_ISL_8372975	MAWD Pathology Group, PA	MAWD Pathology Group, PA	Kelli Fayard; Matison Lewis; Pradip Manna PhD; Shelby Herner; Stephanie Romanowicz
EPI_ISL_8336261, EPI_ISL_8336431, EPI_ISL_8336432, EPI_ISL_8336433, EPI_ISL_8336434, EPI_ISL_8336435, EPI_ISL_8336439, EPI_ISL_8336441			
see above	Ministry of Health Turkey	Ministry of Health Turkey	Fatma Bayrakdar; Gülay Korukluoğlu; Gültekin Ünal; Süleyman Yalcin; Yasemin Coşgun
EPI_ISL_8384065, EPI_ISL_8384066, EPI_ISL_8384067, EPI_ISL_8384068, EPI_ISL_8384069, EPI_ISL_8384070, EPI_ISL_8384071, EPI_ISL_8384072			
see above	National Institute of Public Health	National Institute of Public Health	Alexander Nagy; Helena Jirincova; Jan Moskalyk; Jaromira Vecerova; Timotej Suri
EPI_ISL_8380869	Servicio Microbiología Hospital La Paz	Servicio Microbiología Hospital La Paz	Elie Dahdouh; Fernando Lázaro; Iván Bloise; Jesús Mingorance Cruz; Rubén Cáceres
EPI_ISL_8388410	StarMed Healthcare	UNC Charlotte Environmental Monitoring Laboratory	Cynthia Gibas; Jannatul Ferdous; Jessica Schlueter; Juan Bolanos; Kevin Lambirth; Samuel Kunkleman; Torri Weathers
EPI_ISL_8360501, EPI_ISL_8360516, EPI_ISL_8360598, EPI_ISL_8360650	State Laboratories Division, Hawaii State Department of Health	State Laboratories Division, Hawaii State Department of Health	Ayana Garnet; Briana Ofilas; Cheryl-lynn Daquip; Cheyenne Barela; Daniel Strange; Drew Kuwazaki; Edward Desmond; Jeffrey Au; Mark Nagata; Pamela O'Brien; Razvan Sultana; Remedios Gose; Samantha Cotter; Samantha Sruba
EPI_ISL_8382062	Sweden, Vastragotaland, Unilabs	Unilabs, Eskilstuna, Sweden	Emma Arvidsson
EPI_ISL_8354884, EPI_ISL_8355008	UW Virology Lab	UW Virology Lab	Alexander Greninger; Hong Xie; Isabel Arnould; Keith R Jerome; Meei-Li Huang; Nathan Breit; Patrick Mathias; Pavitra Roychoudhury; Pooneh Hajian; Ricardo Perez; Robert J. Livingston; Sean Ellis; Seffir T. Wendm; Shah Mohamed Bakhash
EPI_ISL_8360079	University of New Mexico Hospital	Center for Global Health, University of New Mexico Health Sciences Center	Darrell Dinwiddie; Daryl Domman; Jesse Young; Jon Femling; Justin Bacca; Kurt Schwalm; Valerie Morley
EPI_ISL_8379873	WHO National Influenza Centre Russian Federation	WHO National Influenza Centre Russian Federation	Andrey Komissarov; Artem Fadeev; Daria Danilenko; Dmitry Lioznov; Elena Nabieva; Georgii Bazykin; Kirill Varchenko; Ksenia Safina; Kseniya Komissarova; Maria Pisareva; Mikhail Bakaev; Nikita Yolshin; Oula Mansour; Tamila Musaeva; Veronika Eder

We gratefully acknowledge the following Authors from the Originating laboratories responsible for obtaining the specimens, as well as the Submitting laboratories where the genome data were generated and shared via GISAID, on which this research is based.

All Submitters of data may be contacted directly via www.gisaid.org

Authors are sorted alphabetically.

Acknowledgement EPI_SET Identifier: EPI_SET_20220527xa

Accession ID	Originating Laboratory	Submitting Laboratory	Authors
EPI_ISL_8424966, EPI_ISL_8425144, EPI_ISL_8426200	Aegis Sciences Corporation	Centers for Disease Control and Prevention Division of Viral Diseases, Pathogen Discovery	Alec Vest; Benjamin Rambo-Martin; Christopher Gulvick; Clinton Paden; Cyndi Clark; Dakota Howard; Dhwani Batra; Dillon Nall; Duncan MacCannell; Erisa Sula; Ethan Sanders; Holly Houdeshell; Jason Caravas; Kristine Lacek; Matthew Hardison; Matthew Schmerer; Ola Kvalvaag; Patrick Campbell; Peter Cook; Rob Case; Scott Sammons; Shatavia Morrison; Shaun Westlund; Tymeckia Kendall; Victoria Caban Figueroa; Vikramsinha Ghorpade; Yvette Unoarumhi
EPI_ISL_8417976, EPI_ISL_8417980, EPI_ISL_8417981, EPI_ISL_8417983, EPI_ISL_8417990, EPI_ISL_8417994, EPI_ISL_8417999, EPI_ISL_8418107, EPI_ISL_8418122, EPI_ISL_8418124, EPI_ISL_8418127, EPI_ISL_8418135, EPI_ISL_8418142, EPI_ISL_8418144, EPI_ISL_8418160, EPI_ISL_8418161, EPI_ISL_8418172, EPI_ISL_8418173, EPI_ISL_8418175, EPI_ISL_8418180, EPI_ISL_8418182, EPI_ISL_8418185, EPI_ISL_8418186, EPI_ISL_8418187, EPI_ISL_8418208, EPI_ISL_8418216, EPI_ISL_8418217, EPI_ISL_8418223, EPI_ISL_8418231, EPI_ISL_8418234, EPI_ISL_8418237, EPI_ISL_8418241, EPI_ISL_8418244, EPI_ISL_8418246, EPI_ISL_8418251, EPI_ISL_8418258, EPI_ISL_8418260, EPI_ISL_8418270, EPI_ISL_8418271, EPI_ISL_8418281, EPI_ISL_8418282, EPI_ISL_8418329, EPI_ISL_8418421, EPI_ISL_8418498, EPI_ISL_8418502, EPI_ISL_8418513, EPI_ISL_8418522, EPI_ISL_8418527, EPI_ISL_8418528, EPI_ISL_8418537, EPI_ISL_8418538, EPI_ISL_8418546, EPI_ISL_8418557, EPI_ISL_8418562, EPI_ISL_8418566, EPI_ISL_8418577, EPI_ISL_8418578, EPI_ISL_8418587, EPI_ISL_8418588, EPI_ISL_8418593, EPI_ISL_8418598, EPI_ISL_8418600, EPI_ISL_8418603, EPI_ISL_8418614, EPI_ISL_8418619, EPI_ISL_8418628, EPI_ISL_8418638, EPI_ISL_8418639, EPI_ISL_8418648, EPI_ISL_8418656, EPI_ISL_8418661, EPI_ISL_8418662, EPI_ISL_8418676, EPI_ISL_8418678, EPI_ISL_8418679, EPI_ISL_8418693, EPI_ISL_8418704, EPI_ISL_8418705, EPI_ISL_8418706, EPI_ISL_8418718, EPI_ISL_8418731, EPI_ISL_8418762, EPI_ISL_8418764, EPI_ISL_8418767, EPI_ISL_8418773, EPI_ISL_8418787, EPI_ISL_8418827, EPI_ISL_8418830, EPI_ISL_8418843, EPI_ISL_8418853, EPI_ISL_8418856, EPI_ISL_8418859, EPI_ISL_8418875, EPI_ISL_8418900, EPI_ISL_8418917, EPI_ISL_8418922, EPI_ISL_8418936, EPI_ISL_8418938, EPI_ISL_8418945, EPI_ISL_8418948, EPI_ISL_8418953, EPI_ISL_8418961, EPI_ISL_8418968, EPI_ISL_8418969, EPI_ISL_8418972, EPI_ISL_8418975, EPI_ISL_8418986, EPI_ISL_8418987, EPI_ISL_8418989, EPI_ISL_8418990, EPI_ISL_8418996, EPI_ISL_8418999, EPI_ISL_8419005, EPI_ISL_8419027, EPI_ISL_8419032, EPI_ISL_8419052, EPI_ISL_8419062, EPI_ISL_8419075, EPI_ISL_8419077, EPI_ISL_8419078, EPI_ISL_8419094, EPI_ISL_8419095, EPI_ISL_8419099, EPI_ISL_8419115, EPI_ISL_8419117, EPI_ISL_8419126, EPI_ISL_8419142, EPI_ISL_8419145, EPI_ISL_8419152, EPI_ISL_8419153, EPI_ISL_8419161, EPI_ISL_8419180, EPI_ISL_8419181, EPI_ISL_8419182, EPI_ISL_8419188, EPI_ISL_8419192, EPI_ISL_8419211, EPI_ISL_8419213, EPI_ISL_8419224, EPI_ISL_8419226, EPI_ISL_8419237, EPI_ISL_8419241, EPI_ISL_8419243, EPI_ISL_8419252, EPI_ISL_8419269, EPI_ISL_8419278, EPI_ISL_8419285, EPI_ISL_8419289, EPI_ISL_8419290, EPI_ISL_8419292, EPI_ISL_8419302, EPI_ISL_8419303, EPI_ISL_8419305, EPI_ISL_8419309, EPI_ISL_8419314, EPI_ISL_8419324, EPI_ISL_8419329, EPI_ISL_8419332, EPI_ISL_8419336, EPI_ISL_8419342, EPI_ISL_8419355, EPI_ISL_8419356, EPI_ISL_8419360, EPI_ISL_8419367, EPI_ISL_8419368, EPI_ISL_8419378, EPI_ISL_8419379, EPI_ISL_8419383, EPI_ISL_8419386, EPI_ISL_8419388, EPI_ISL_8419395, EPI_ISL_8419396, EPI_ISL_8419406, EPI_ISL_8419411, EPI_ISL_8419415, EPI_ISL_8419426, EPI_ISL_8419429, EPI_ISL_8419442, EPI_ISL_8419447, EPI_ISL_8419454, EPI_ISL_8419459, EPI_ISL_8419465, EPI_ISL_8419468, EPI_ISL_8419475, EPI_ISL_8419477, EPI_ISL_8419481, EPI_ISL_8419499, EPI_ISL_8419501, EPI_ISL_8419505, EPI_ISL_8419516, EPI_ISL_8419518, EPI_ISL_8419558, EPI_ISL_8419589, EPI_ISL_8419667, EPI_ISL_8419673, EPI_ISL_8419682, EPI_ISL_8419697, EPI_ISL_8419699, EPI_ISL_8419702, EPI_ISL_8419703, EPI_ISL_8419704, EPI_ISL_8419716, EPI_ISL_8419720, EPI_ISL_8419727, EPI_ISL_8419733, EPI_ISL_8419745, EPI_ISL_8419752, EPI_ISL_8419766, EPI_ISL_8419771, EPI_ISL_8419774, EPI_ISL_8419778, EPI_ISL_8419779, EPI_ISL_8419786, EPI_ISL_8419787, EPI_ISL_8419790, EPI_ISL_8419806, EPI_ISL_8419841, EPI_ISL_8419847, EPI_ISL_8419850, EPI_ISL_8419857, EPI_ISL_8419872, EPI_ISL_8419873, EPI_ISL_8419875, EPI_ISL_8419882, EPI_ISL_8419884, EPI_ISL_8419888, EPI_ISL_8419905, EPI_ISL_8419910, EPI_ISL_8419915, EPI_ISL_8419932, EPI_ISL_8419933, EPI_ISL_8419936, EPI_ISL_8419937, EPI_ISL_8419938, EPI_ISL_8419947, EPI_ISL_8419948, EPI_ISL_8420649, EPI_ISL_8420658, EPI_ISL_8420669, EPI_ISL_8420681, EPI_ISL_8420684, EPI_ISL_8420685, EPI_ISL_8420691, EPI_ISL_8420712			
see above	Department of Bacteria, Parasites and Fungi, Statens Serum Institut, Copenhagen, Denmark	Statens Serum Institut Bioinformatics and Microbial Genomics	Danish Covid-19 Genome Consortium
EPI_ISL_8414381	MVZ Labor Dr. Fenner und Kollegen (Standort Hamburg)	Robert Koch Institute	
EPI_ISL_8421377	Medical Virology and BSL3 Laboratory	Centre de Séquençage Génomique	Abbad Anas; Amalou Ghita; Anga Latifa; Barakat Abdelhamid; Bouzidi Aymane; Charoute Hicham; Chgouri Fatima; Dersi Noureddine; EL Hamouchi Adil; El Oualid Abdelmjid; Faouzi Abdellah; Harmak Houda; Maaroufi Abderrahmane; Nadifiyine Saloua; Nourlil Jalal; Omondi Francis Carey; Somda Soro Georgina Charlene; Zemmouri Faouzia; Zouheir Yassine
EPI_ISL_8414362, EPI_ISL_8414363	National Institute of Public Health	National Institute of Public Health	Alexander Nagy; Helena Jirincova; Jan Moskalyk; Jaromira Vecerova; Timotej Suri
EPI_ISL_8410970	SYNLAB MVZ Leverkusen	Robert Koch Institute	
EPI_ISL_8427960, EPI_ISL_8428054	The New York Center for Travel and Tropical Medicine	Biotia	Christopher Mason; David Danko; Dorottya Nagy-Szakal; Mara Couto-Rodriguez; Marilyne Debieu; Niamh O'Hara; Xavier Jirau Serrano

We gratefully acknowledge the following Authors from the Originating laboratories responsible for obtaining the specimens, as well as the Submitting laboratories where the genome data were generated and shared via GISAID, on which this research is based.

All Submitters of data may be contacted directly via www.gisaid.org

Authors are sorted alphabetically.

Acknowledgement EPI_SET Identifier: EPI_SET_20220527ng

Accession ID	Originating Laboratory	Submitting Laboratory	Authors
EPI_ISL_8552074, EPI_ISL_8552075, EPI_ISL_8552076, EPI_ISL_8552084, EPI_ISL_8552086, EPI_ISL_8552090, EPI_ISL_8552098, EPI_ISL_8554348, EPI_ISL_8555368, EPI_ISL_8555369, EPI_ISL_8555536, EPI_ISL_8555537			
see above	Aegis Sciences Corporation	Centers for Disease Control and Prevention Division of Viral Diseases, Pathogen Discovery	Alec Vest; Benjamin Rambo-Martin; Christopher Gulvick; Clinton Paden; Cyndi Clark; Dakota Howard; Dhwani Batra; Dillon Nall; Duncan MacCannell; Erisa Sula; Ethan Sanders; Holly Houdeshell; Jason Caravas; Kristine Lacek; Matthew Hardison; Matthew Schmerer; Ola Kvalvaag; Patrick Campbell; Peter Cook; Rob Case; Scott Sammons; Shatavia Morrison; Shaun Westlund; Tymeckia Kendall; Victoria Caban Figueroa; Vikramsinha Ghorpade; Yvette Unoarumhi
EPI_ISL_8543197, EPI_ISL_8543200, EPI_ISL_8543202, EPI_ISL_8543208	Arcispedale Santa Maria Nuova Autoimmunita Allergologia e Biotecnologie Innovative""	Istituto Zooprofilattico Sperimentale della Lombardia e dell'Emilia Romagna (IZSLER), Risk Analysis and Genomic Epidemiology Unit	Erika Scaltriti; Ilaria Menozzi; Marina Morganti; Stefano Pongolini
EPI_ISL_8509194	Arizona State University	Arizona State University	Efrem S. Lim; James C. Hu; Joshua LaBaer; LaRinda A. Holland; Matthew F. Smith; Nathaniel Johnson; Regan A. Sullins; Steven C. Holland; Vel Murugan
EPI_ISL_8543243, EPI_ISL_8543245	Azienda Ospedaliero - Universitaria di Modena Policlinico - Virologia e Microbiologia Molecolare""	Istituto Zooprofilattico Sperimentale della Lombardia e dell'Emilia Romagna (IZSLER), Risk Analysis and Genomic Epidemiology Unit	Erika Scaltriti; Ilaria Menozzi; Marina Morganti; Stefano Pongolini
EPI_ISL_8519792, EPI_ISL_8519793	Bidh Lab	National Public Health Laboratory	National Public Health Labaoratory Team/Bidh Lab Team
EPI_ISL_8547008	Clinical Microbiology Laboratory, Tel Aviv Sourasky Medical Center	Clinical Microbiology Laboratory, Tel Aviv Sourasky Medical Center	Alon Ziv; Amos Adler; Goel Morad; Katya Levytskyi; Lior Handler; Ora Halutz
EPI_ISL_8515049, EPI_ISL_8555439, EPI_ISL_8555440, EPI_ISL_8555441	Colorado Department of Public Health and Environment	Colorado Department of Public Health and Environment	Alexandria Rossheim; Arianna Smith; Diana Ir; Emily A. Travanty; Laura Bankers; Mandy Waters; Michael Martin; Molly C. Hetherington-Rauth; Shannon R. Matzinger
EPI_ISL_8528767, EPI_ISL_8528771, EPI_ISL_8528816, EPI_ISL_8528823, EPI_ISL_8528827, EPI_ISL_8528832, EPI_ISL_8528836, EPI_ISL_8528838, EPI_ISL_8528839, EPI_ISL_8528842, EPI_ISL_8528845, EPI_ISL_8528853, EPI_ISL_8528854, EPI_ISL_8528870, EPI_ISL_8528876, EPI_ISL_8528879, EPI_ISL_8528880, EPI_ISL_8528883, EPI_ISL_8528885, EPI_ISL_8528886, EPI_ISL_8528888, EPI_ISL_8528896, EPI_ISL_8528897, EPI_ISL_8528912, EPI_ISL_8528926, EPI_ISL_8528929, EPI_ISL_8528933, EPI_ISL_8528937, EPI_ISL_8528938, EPI_ISL_8528939, EPI_ISL_8528940, EPI_ISL_8528944, EPI_ISL_8528954, EPI_ISL_8528961, EPI_ISL_8528973, EPI_ISL_8528974, EPI_ISL_8528976, EPI_ISL_8528979, EPI_ISL_8528989, EPI_ISL_8528998, EPI_ISL_8529001, EPI_ISL_8529022, EPI_ISL_8529031, EPI_ISL_8529032, EPI_ISL_8529037, EPI_ISL_8529041, EPI_ISL_8529060, EPI_ISL_8529069, EPI_ISL_8529074, EPI_ISL_8529075, EPI_ISL_8529076, EPI_ISL_8529099, EPI_ISL_8529105, EPI_ISL_8529108, EPI_ISL_8529112, EPI_ISL_8529121, EPI_ISL_8529134, EPI_ISL_8529136, EPI_ISL_8529139, EPI_ISL_8529142, EPI_ISL_8529144, EPI_ISL_8529147, EPI_ISL_8529148, EPI_ISL_8529165, EPI_ISL_8529166, EPI_ISL_8529172, EPI_ISL_8529176, EPI_ISL_8529181, EPI_ISL_8529187, EPI_ISL_8529191, EPI_ISL_8529194, EPI_ISL_8529204, EPI_ISL_8529210, EPI_ISL_8529212, EPI_ISL_8529228, EPI_ISL_8529233, EPI_ISL_8529244, EPI_ISL_8529268, EPI_ISL_8529269, EPI_ISL_8529279, EPI_ISL_8529301, EPI_ISL_8529309, EPI_ISL_8529320, EPI_ISL_8529321, EPI_ISL_8529322, EPI_ISL_8529324, EPI_ISL_8529325, EPI_ISL_8529326, EPI_ISL_8529339, EPI_ISL_8529345, EPI_ISL_8529349, EPI_ISL_8529357, EPI_ISL_8529362, EPI_ISL_8529367, EPI_ISL_8529368, EPI_ISL_8529369, EPI_ISL_8529377, EPI_ISL_8529398, EPI_ISL_8529402, EPI_ISL_8529404, EPI_ISL_8529408, EPI_ISL_8529415, EPI_ISL_8529427, EPI_ISL_8529428, EPI_ISL_8529430, EPI_ISL_8529435, EPI_ISL_8529443, EPI_ISL_8529444, EPI_ISL_8529451, EPI_ISL_8529458, EPI_ISL_8529461, EPI_ISL_8529462, EPI_ISL_8529466, EPI_ISL_8529471, EPI_ISL_8529477, EPI_ISL_8529479, EPI_ISL_8529501, EPI_ISL_8529506, EPI_ISL_8529512, EPI_ISL_8529518, EPI_ISL_8529519, EPI_ISL_8529523, EPI_ISL_8529524, EPI_ISL_8529525, EPI_ISL_8529527, EPI_ISL_8529528, EPI_ISL_8529529, EPI_ISL_8529540, EPI_ISL_8529543, EPI_ISL_8529562, EPI_ISL_8529563, EPI_ISL_8529567, EPI_ISL_8529569, EPI_ISL_8529579, EPI_ISL_8529580, EPI_ISL_8529584, EPI_ISL_8529587, EPI_ISL_8529591, EPI_ISL_8529598, EPI_ISL_8529620, EPI_ISL_8529622, EPI_ISL_8529634, EPI_ISL_8529635, EPI_ISL_8529647, EPI_ISL_8529648, EPI_ISL_8529659, EPI_ISL_8529665, EPI_ISL_8529673, EPI_ISL_8529675, EPI_ISL_8529680, EPI_ISL_8529688, EPI_ISL_8529694, EPI_ISL_8529702, EPI_ISL_8529708, EPI_ISL_8529709, EPI_ISL_8529711, EPI_ISL_8529718, EPI_ISL_8529726, EPI_ISL_8529727, EPI_ISL_8529733, EPI_ISL_8529735, EPI_ISL_8529736, EPI_ISL_8529749, EPI_ISL_8529754, EPI_ISL_8529784, EPI_ISL_8529806, EPI_ISL_8529815, EPI_ISL_8529817, EPI_ISL_8529820, EPI_ISL_8529846, EPI_ISL_8529875, EPI_ISL_8529878, EPI_ISL_8529889, EPI_ISL_8529891, EPI_ISL_8529893, EPI_ISL_8529897, EPI_ISL_8529904, EPI_ISL_8529905, EPI_ISL_8529907, EPI_ISL_8529911, EPI_ISL_8529918, EPI_ISL_8529922, EPI_ISL_8529930, EPI_ISL_8529943, EPI_ISL_8529955, EPI_ISL_8529989, EPI_ISL_8529991, EPI_ISL_8529998, EPI_ISL_8530000, EPI_ISL_8530002, EPI_ISL_8530017, EPI_ISL_8530020, EPI_ISL_8530022, EPI_ISL_8530025, EPI_ISL_8530035, EPI_ISL_8530041, EPI_ISL_8530042, EPI_ISL_8530045, EPI_ISL_8530063, EPI_ISL_8530064, EPI_ISL_8530069			
see above	Department of Bacteria, Parasites and Fungi, Statens Serum Institut, Copenhagen, Denmark	Statens Serum Institut Bioinformatics and Microbial Genomics	Danish Covid-19 Genome Consortium
EPI_ISL_8508635	Dianalabs SA	Genesupport	Geraldine Jost; Katia Jaton; Nadia Liassine; Tanguy ARAUD
EPI_ISL_8504750, EPI_ISL_8504754	Fulgent Genetics	Centers for Disease Control and Prevention Division of Viral Diseases, Pathogen Discovery	Becky Tsai; Benafsh Sapra; Benjamin Rambo-Martin; Christopher Gulvick; Clinton Paden; Dakota Howard; Dhwani Batra; Doreen Ng; Duncan MacCannell; Erisa Sula; Harry Gao; James Xie; Jason Caravas; John Gao; Joseph Fierro; Kristine Lacek; Matthew Schmerer; Mickey Li; Peter Cook; Scott Sammons; Shatavia Morrison; Tymeckia Kendall; Victoria Caban Figueroa; Yan Meng; Yvette Unoarumhi
EPI_ISL_8527502, EPI_ISL_8527509, EPI_ISL_8527512, EPI_ISL_8527524, EPI_ISL_8527529, EPI_ISL_8527533, EPI_ISL_8527536, EPI_ISL_8527537, EPI_ISL_8527542, EPI_ISL_8527543, EPI_ISL_8527548, EPI_ISL_8527550, EPI_ISL_8527552, EPI_ISL_8527554, EPI_ISL_8527564, EPI_ISL_8527568, EPI_ISL_8527571, EPI_ISL_8527572, EPI_ISL_8527573, EPI_ISL_8527574, EPI_ISL_8527575, EPI_ISL_8527578, EPI_ISL_8527579, EPI_ISL_8527580, EPI_ISL_8527585, EPI_ISL_8527586, EPI_ISL_8527589, EPI_ISL_8527591			
see above	Gandhi Medical College and Hospital (GMCH), Secunderabad	NIV Influenza	D.R.Manisha Rani; Dr.G.Sushma Rajya Lakshmi; Dr.K.Nagamani; Dr.Sunitha Pakalapaty
EPI_ISL_8547703, EPI_ISL_8547714, EPI_ISL_8547723, EPI_ISL_8547724, EPI_ISL_8547736, EPI_ISL_8547746, EPI_ISL_8547858, EPI_ISL_8547987, EPI_ISL_8548249, EPI_ISL_8548251, EPI_ISL_8548252, EPI_ISL_8548253, EPI_ISL_8548261, EPI_ISL_8548264			
see above	Helix	Centers for Disease Control and Prevention Division of Viral Diseases, Pathogen Discovery	Benjamin Rambo-Martin; Christopher Gulvick; Clinton Paden; Dakota Howard; Dhwani Batra; Duncan MacCannell; Erisa Sula; Helix CA; Jason Caravas; Kristine Lacek; Matthew Schmerer; Peter Cook; Scott Sammons; Shatavia Morrison; Tymeckia Kendall; Victoria Caban Figueroa; Yvette Unoarumhi
EPI_ISL_8553407	Hospital Universitari Vall d'Hebron - Vall d'Hebron Institut de Recerca	Hospital Universitari Vall d'Hebron - Vall d'Hebron Institut de Recerca	Alejandra González-Sánchez; Andrés Antón; Ariadna Rando; Carla Castillo; Cristina Andrés; Damir Garcia-Cehic; Josep Quer; Juliana Esperalba; Karen García; Maria Carmen Martin; Maria Gema Codina; Maria Piñana; Rodrigo Vásquez; Tomàs Pumarola
EPI_ISL_8525201, EPI_ISL_8525219, EPI_ISL_8525234	Indira Gandhi Memorial Hospital	Indira Gandhi Memorial Hospital	D. Fathmath Nazla Rafeeq; Dr. Ibrahim Afzal; Mr. Ibrahim Nishan Ahmed; Ms. Aishath Shuhudha; Ms. Aminath Shazleena Abdul Rahman; Ms. Soafy Mohamed
EPI_ISL_8505377	Infinity Biologix	Centers for Disease Control and Prevention Division of Viral Diseases, Pathogen Discovery	Benjamin Rambo-Martin; Chirayu Goswami; Christian Bixby; Christopher Gulvick; Clinton Paden; Dakota Howard; Dhwani Batra; Duncan MacCannell; Erisa Sula; Jason Caravas; Jonathan Schultz; Kristine Lacek; Matthew Schmerer; Peter Cook; Robin Grimwood; Russ Hager; Scott Sammons; Shatavia Morrison; Tymeckia Kendall; Victoria Caban Figueroa; Yihe Wang; Yvette Unoarumhi
EPI_ISL_8538550	Institute for Infectious Diseases	Institute for Infectious Diseases, University of Bern	Alban Ramette; Christian Baumann; Cora Sägesser; Franziska Suter-Riniker; Loïc Borcard; Miguel A Terrazos Miani; Nicole Liechti; Pascal Bittel; Peter Keller; Sonja Gempeler; Stefan Neuenschwander; Stephen L Leib
EPI_ISL_8519789	Interpid Nepal	National Public Health Laboratory	National Public Health Labaoratory Team/Intrepid Lab Team
EPI_ISL_8519794	Kathmandu Medical College	National Public Health Laboratory	National Public Health Laboartory Team/Kathmandu Medical College Team
EPI_ISL_8515274, EPI_ISL_8515275, EPI_ISL_8515276, EPI_ISL_8515279, EPI_ISL_8515286, EPI_ISL_8515287, EPI_ISL_8515289, EPI_ISL_8515293, EPI_ISL_8515302, EPI_ISL_8515324, EPI_ISL_8515325, EPI_ISL_8515332, EPI_ISL_8515342, EPI_ISL_8515348, EPI_ISL_8515349, EPI_ISL_8515377, EPI_ISL_8515381, EPI_ISL_8515382, EPI_ISL_8515383, EPI_ISL_8515398, EPI_ISL_8515399, EPI_ISL_8515401, EPI_ISL_8515402, EPI_ISL_8515410, EPI_ISL_8515411, EPI_ISL_8515412, EPI_ISL_8515414, EPI_ISL_8515415, EPI_ISL_8515422, EPI_ISL_8515423, EPI_ISL_8515428, EPI_ISL_8515432, EPI_ISL_8515433, EPI_ISL_8515435, EPI_ISL_8515436, EPI_ISL_8515438, EPI_ISL_8515439, EPI_ISL_8515441, EPI_ISL_8515443, EPI_ISL_8515445, EPI_ISL_8515448, EPI_ISL_8515451, EPI_ISL_8515452, EPI_ISL_8515455, EPI_ISL_8515457, EPI_ISL_8515464, EPI_ISL_8515467, EPI_ISL_8515468, EPI_ISL_8515470, EPI_ISL_8515472, EPI_ISL_8515473, EPI_ISL_8515475, EPI_ISL_8515477, EPI_ISL_8544443, EPI_ISL_8544444, EPI_ISL_8544445, EPI_ISL_8544446, EPI_ISL_8544447, EPI_ISL_8544448, EPI_ISL_8544449, EPI_ISL_8544450, EPI_ISL_8544451, EPI_ISL_8544452, EPI_ISL_8544453, EPI_ISL_8544454, EPI_ISL_8544455, EPI_ISL_8544456, EPI_ISL_8544457, EPI_ISL_8544458, EPI_ISL_8544463, EPI_ISL_8544464, EPI_ISL_8544465, EPI_ISL_8544466, EPI_ISL_8544467, EPI_ISL_8544468, EPI_ISL_8544469, EPI_ISL_8544470, EPI_ISL_8544471, EPI_ISL_8544472, EPI_ISL_8544473, EPI_ISL_8544474, EPI_ISL_8544475			
see above	Lab. Microbiologia e Virologia Cotugno A.O. dei Colli	TIGEM	Antonio Grimaldi Patrizia Annunziata Francesco Panariello Claudia Tiberio Teresa Giuliano Valentina Bouche Chiara Colantuono Michela Daniele Lucio Di Filippo Anna Manfredi Marcello Salvi Antonio Limone Luigi Atripaldi Andrea Ballabio Davide Cacchiarelli
EPI_ISL_8546646, EPI_ISL_8546647	Labo Analyses Med	National Reference Center for Viruses of Respiratory Infections, Institut Pasteur, Paris	Angela Brisebarre; Camille Capel; Christophe Malabat; Corinne Maufrais; Etienne Simon-Lorière; Fabienne HUET; Frédéric Lemoine; Julien Fumey; Laurence PROTS; Louise Lefrançois; Marion Barbet; Maud Vanpeene; Méline Bizard; Slim El Khiari; Sylvie Behillil; Sylvie Van der Werf; Vincent Enouf
EPI_ISL_8547156	Laboratorio Analisi Osp. Città di Castello - Azienda USL Umbria1	Istituto Zooprofilattico Sperimentale dell'Abruzzo e Molise "G. Caporale"	Ancora M; Biagetti M; Calistri P; Cammà C; Curini V; Delli Compagni E; Di Domenico M; Di Pasquale A; Giammarioli M; Lorusso A; Malagigi V; Mangone I; Marcacci M; Puglia I; Rinaldi A; Savini G; Scialabba S; Tacconi P
EPI_ISL_8533328, EPI_ISL_8533337, EPI_ISL_8533338, EPI_ISL_8533359	Lighthouse Lab in Glasgow	Wellcome Sanger Institute for the COVID-19 Genomics UK (COG-UK) Consortium	Anna Dominiczak and Alex Alderton; Carol Clugston; Cordelia Langford; David Gray; David K. Jackson; Dominic Kwiatkowski; Ewan Harrison; Harper VanSteenhouse; Ian Johnston; Jeffrey Barrett; John Sillitoe on behalf of the Wellcome Sanger Institute COVID-19 Surveillance Team; Roberto Amato; Sonia Goncalves; Yumi Kasai
EPI_ISL_8523907	Medical Virology and BSL3 Laboratory	Centre de Séquençage Génomique	Abbad Anas; Amalou Ghita; Anga Latifa; Barakat Abdelhamid; Bouzidi Aymane; Charoute Hicham; Chgouri Fatima; Dersi Noureddine; EL Hamouchi Adil; El Oualid Abdelmjid; Faouzi Abdellah; Harmak Houda; Maaroufi Abderrahmane; Nadifiyine Saloua; Nourlil Jalal; Omondi Francis Carey; Somda Soro Georgina Charlene; Zemmouri Faouzia; Zouheir Yassine
EPI_ISL_8556454, EPI_ISL_8556456, EPI_ISL_8556458, EPI_ISL_8556473, EPI_ISL_8556502	Michigan Department of Health and Human Services, Bureau of Laboratories	Michigan Department of Health and Human Services, Bureau of Laboratories	Blankenship HM; Riner D; Soehnlen MK
EPI_ISL_8513840, EPI_ISL_8513858, EPI_ISL_8513930, EPI_ISL_8514024, EPI_ISL_8514025, EPI_ISL_8514026, EPI_ISL_8514105			
see above	Ministry of Health Turkey	Ministry of Health Turkey	Fatma Bayrakdar; Gulay Korukluoglu; Suleyman Yalcin; Yasemin Cosgun
EPI_ISL_8509383, EPI_ISL_8509390, EPI_ISL_8509403, EPI_ISL_8509405, EPI_ISL_8509416, EPI_ISL_8509420	Molecular Biology Laboratory, Vidant Medical Center	Brody Integrative Genomics Core, East Carolina University	Changhong Yin; Heather Duncan; John T. Fallon; Kimberly P. Briley; Weihua Huang
EPI_ISL_8540327, EPI_ISL_8540328	National Hematology and Transfusiology Center, Department of Medical Genetics	National Hematology and Transfusiology Center, Department of Medical Genetics	Agha Rza Aghayev
EPI_ISL_8519771	National Public Health Laboratory	National Public Health Laboratory	National Public Health Labaoratory Team
EPI_ISL_8519790	Nepal Korea Friendship Hospital	National Public Health Laboratory	National Public Health Labaoratory Team/Nepal Korea Friendship Hospital Team
EPI_ISL_8519772	PPHL-5	National Public Health Laboratory	National Public Health Labaoratory Team//Provincial Public Health Laboratory -5 COVID Lab Team
EPI_ISL_8519773	Patan Hospital	National Public Health Laboratory	National Public Health Labaoratory Team /PATAN Hospital
EPI_ISL_8557231, EPI_ISL_8557382	Quest Diagnostics Incorporated	Centers for Disease Control and Prevention Division of Viral Diseases, Pathogen Discovery	A. Gerasimova; A. Perez; B. Anderson; Benjamin Rambo-Martin; Christopher Gulvick; Clinton Paden; Dakota Howard; Dhwani Batra; Duncan MacCannell; Erisa Sula; F. Lacbawan; I. Shlyakhter; Jason Caravas; K. Livingston; Kristine Lacek; L. Bernstein; M. Hua; Matthew Schmerer; P. Tanpaiboon; Peter Cook; R. Kagan; R. Owen; R. Rolando; S. Rosenthal; Scott Sammons; Shatavia Morrison; Tymeckia Kendall; Victoria Caban Figueroa; Y. Liu; Yvette Unoarumhi
EPI_ISL_8488281, EPI_ISL_8488283, EPI_ISL_8488289, EPI_ISL_8488292, EPI_ISL_8517222, EPI_ISL_8517384, EPI_ISL_8517424, EPI_ISL_8539753, EPI_ISL_8539761, EPI_ISL_8539773, EPI_ISL_8539784, EPI_ISL_8539787, EPI_ISL_8539864, EPI_ISL_8539916			
see above	Respiratory Virus Unit, Microbiology Services Colindale, Public Health England	COVID-19 Genomics UK (COG-UK) Consortium	PHE Covid Sequencing Team
EPI_ISL_8519791	Shahid Gangalal Heart Center	National Public Health Laboratory	National Public Health Labaoratory Team/Shahid Gangalal Heart center Team
EPI_ISL_8519774, EPI_ISL_8519776, EPI_ISL_8519777, EPI_ISL_8519779, EPI_ISL_8519780, EPI_ISL_8519781, EPI_ISL_8519782, EPI_ISL_8519783, EPI_ISL_8519784, EPI_ISL_8519785, EPI_ISL_8519786, EPI_ISL_8519787, EPI_ISL_8519788			

see above	Shree Birendra Hospital	National Public Health Laboratory	National Public Health Labaoratory Team/Shree Birendra Hospital COVID -19 Lab team
EPI_ISL_8494337	Supratech Micropath Laboratory Research Institute Pvt Ltd, Ahmedabad	Gujarat Biotechnology Research Centre	Akhilesh Modi; Apurvasinh Puvar; Bhadreshsinh Gohil; Chaitanya Joshi; Disha Vora; Janvi Raval; Jaykumar Rangani; Madhvi Joshi; Nimesh Patel; Nitin Savaliya; Nitin Shukla; Priyank Chavda; Ramesh Pandit; Roshani Mishra; Shiva; Sonal Sharma; Tasnim Trivedi; Zarna Patel
EPI_ISL_8487263, EPI_ISL_8487371	The Jackson Laboratory	The Jackson Laboratory	Adams M; Kelly K; Li L; Long E; Omerza G; Renzette N
EPI_ISL_8519196, EPI_ISL_8519205, EPI_ISL_8519227	UW Virology Lab	UW Virology Lab	Alexander Greninger; Hong Xie; Isabel Arnould; Keith R Jerome; Meei-Li Huang; Nathan Breit; Patrick Mathias; Pavitra Roychoudhury; Pooneh Hajian; Ricardo Perez; Robert J. Livingston; Sean Ellis; Seffir T. Wendm; Shah Mohamed Bakhash
EPI_ISL_8519054	University of New Mexico Hospital	Center for Global Health, University of New Mexico Health Sciences Center	Darrell Dinwiddie; Daryl Domman; Jesse Young; Jon Femling; Justin Bacca; Kurt Schwalm; Valerie Morley
EPI_ISL_8543599	University of Wisconsin-Madison AIDS Vaccine Research Laboratories	University of Wisconsin-Madison AIDS Vaccine Research Laboratories	Gage Moreno; Katarina Braun; et al. AIDS Vaccine Research Laboratories

We gratefully acknowledge the following Authors from the Originating laboratories responsible for obtaining the specimens, as well as the Submitting laboratories where the genome data were generated and shared via GISAID, on which this research is based.

All Submitters of data may be contacted directly via www.gisaid.org

Authors are sorted alphabetically.

Acknowledgement EPI_SET Identifier: EPI_SET_20220527ws

Accession ID	Originating Laboratory	Submitting Laboratory	Authors
EPI_ISL_8568509	Arcispedale Santa Maria Nuova Autoimmunita Allergologia e Biotecnologie Innovative""	Istituto Zooprofilattico Sperimentale della Lombardia e dell'Emilia Romagna (IZSLER), Risk Analysis and Genomic Epidemiology Unit	Erika Scaltriti; Ilaria Menozzi; Marina Morganti; Stefano Pongolini
EPI_ISL_8568510	Azienda Sanitaria Locale di Piacenza - Presidio Ospedaliero - Laboratorio di Microbiologia""	Istituto Zooprofilattico Sperimentale della Lombardia e dell'Emilia Romagna (IZSLER), Risk Analysis and Genomic Epidemiology Unit	Erika Scaltriti; Ilaria Menozzi; Marina Morganti; Stefano Pongolini
EPI_ISL_8564603, EPI_ISL_8564609, EPI_ISL_8564615, EPI_ISL_8564665, EPI_ISL_8564674, EPI_ISL_8564687, EPI_ISL_8564697, EPI_ISL_8564713, EPI_ISL_8564730, EPI_ISL_8564732, EPI_ISL_8564744, EPI_ISL_8564752, EPI_ISL_8564763, EPI_ISL_8564766, EPI_ISL_8564773, EPI_ISL_8564778, EPI_ISL_8564782, EPI_ISL_8564817, EPI_ISL_8564830, EPI_ISL_8564842, EPI_ISL_8564879, EPI_ISL_8564904, EPI_ISL_8564911, EPI_ISL_8564917, EPI_ISL_8564928, EPI_ISL_8564932, EPI_ISL_8564933, EPI_ISL_8564935, EPI_ISL_8565526, EPI_ISL_8565533, EPI_ISL_8565535, EPI_ISL_8565576, EPI_ISL_8565588, EPI_ISL_8565589, EPI_ISL_8565591			
see above	Curative Labs	Curative Labs	Elias L. Salfati; Eugenia Khorosheva; George Way; J.Cesar Ignacio-Espinoza; Janet Chen; Mikhail Hanewich-Hollatz; Nabjot Sandhu; Sophia Quasem; Vladimir Slepnev; Zhiyi Xie
EPI_ISL_8578158, EPI_ISL_8578167, EPI_ISL_8578168, EPI_ISL_8578177, EPI_ISL_8578182, EPI_ISL_8578189, EPI_ISL_8578190, EPI_ISL_8578202, EPI_ISL_8578203, EPI_ISL_8578205, EPI_ISL_8578209, EPI_ISL_8578213, EPI_ISL_8578218, EPI_ISL_8578254, EPI_ISL_8578258, EPI_ISL_8578259, EPI_ISL_8578264, EPI_ISL_8578270, EPI_ISL_8578373, EPI_ISL_8578374, EPI_ISL_8578383, EPI_ISL_8578386, EPI_ISL_8578393, EPI_ISL_8578396, EPI_ISL_8578406, EPI_ISL_8578409, EPI_ISL_8578410, EPI_ISL_8578415, EPI_ISL_8578424, EPI_ISL_8578425, EPI_ISL_8578426, EPI_ISL_8578428, EPI_ISL_8578446, EPI_ISL_8578454, EPI_ISL_8578459, EPI_ISL_8578461, EPI_ISL_8578472, EPI_ISL_8578474, EPI_ISL_8578484, EPI_ISL_8578485, EPI_ISL_8578488, EPI_ISL_8578499, EPI_ISL_8578507, EPI_ISL_8578517, EPI_ISL_8578521, EPI_ISL_8578522, EPI_ISL_8578528, EPI_ISL_8578529, EPI_ISL_8578535, EPI_ISL_8578536, EPI_ISL_8578545, EPI_ISL_8578547, EPI_ISL_8578552, EPI_ISL_8578558, EPI_ISL_8578570, EPI_ISL_8578576, EPI_ISL_8578580, EPI_ISL_8578590, EPI_ISL_8578606, EPI_ISL_8578615, EPI_ISL_8578618, EPI_ISL_8578622, EPI_ISL_8578629, EPI_ISL_8578631, EPI_ISL_8578654, EPI_ISL_8578660, EPI_ISL_8578672, EPI_ISL_8578675, EPI_ISL_8578677, EPI_ISL_8578678, EPI_ISL_8578680, EPI_ISL_8578686, EPI_ISL_8578689, EPI_ISL_8578695, EPI_ISL_8578699, EPI_ISL_8578706, EPI_ISL_8578721, EPI_ISL_8578723, EPI_ISL_8578735, EPI_ISL_8578742, EPI_ISL_8578747, EPI_ISL_8578748, EPI_ISL_8578753, EPI_ISL_8578761, EPI_ISL_8578769, EPI_ISL_8578770, EPI_ISL_8578807, EPI_ISL_8578811, EPI_ISL_8578814, EPI_ISL_8578815, EPI_ISL_8578823, EPI_ISL_8578832, EPI_ISL_8578843, EPI_ISL_8578844, EPI_ISL_8578849, EPI_ISL_8578866, EPI_ISL_8578868, EPI_ISL_8578870, EPI_ISL_8578874, EPI_ISL_8578885, EPI_ISL_8578892, EPI_ISL_8578896, EPI_ISL_8578908, EPI_ISL_8578912, EPI_ISL_8578933, EPI_ISL_8578937, EPI_ISL_8578938, EPI_ISL_8578943, EPI_ISL_8578963, EPI_ISL_8578964, EPI_ISL_8578969, EPI_ISL_8578982, EPI_ISL_8578989, EPI_ISL_8578998, EPI_ISL_8579013, EPI_ISL_8579015, EPI_ISL_8579017, EPI_ISL_8579019, EPI_ISL_8579028, EPI_ISL_8579030, EPI_ISL_8579031, EPI_ISL_8579033, EPI_ISL_8579034, EPI_ISL_8579039, EPI_ISL_8579045, EPI_ISL_8579046, EPI_ISL_8579047, EPI_ISL_8579056, EPI_ISL_8579058, EPI_ISL_8579065, EPI_ISL_8579078, EPI_ISL_8579083, EPI_ISL_8579103, EPI_ISL_8579112, EPI_ISL_8579116, EPI_ISL_8579125, EPI_ISL_8579130, EPI_ISL_8579145, EPI_ISL_8579151, EPI_ISL_8579152, EPI_ISL_8579154, EPI_ISL_8579158, EPI_ISL_8579161, EPI_ISL_8579164, EPI_ISL_8579167, EPI_ISL_8579172, EPI_ISL_8579180, EPI_ISL_8579181, EPI_ISL_8579186, EPI_ISL_8579187, EPI_ISL_8579190, EPI_ISL_8579211, EPI_ISL_8579222, EPI_ISL_8579228, EPI_ISL_8579242, EPI_ISL_8579244, EPI_ISL_8579246, EPI_ISL_8579248, EPI_ISL_8579253, EPI_ISL_8579260, EPI_ISL_8579263, EPI_ISL_8579280, EPI_ISL_8579281, EPI_ISL_8579289, EPI_ISL_8579298, EPI_ISL_8579303, EPI_ISL_8579304, EPI_ISL_8579310, EPI_ISL_8579327, EPI_ISL_8579333, EPI_ISL_8579338, EPI_ISL_8579339, EPI_ISL_8579349, EPI_ISL_8579351, EPI_ISL_8579353, EPI_ISL_8579357, EPI_ISL_8579359, EPI_ISL_8579363, EPI_ISL_8579367, EPI_ISL_8579372, EPI_ISL_8579376, EPI_ISL_8579382, EPI_ISL_8579392, EPI_ISL_8579399, EPI_ISL_8579403, EPI_ISL_8579406, EPI_ISL_8579407, EPI_ISL_8579411, EPI_ISL_8579414, EPI_ISL_8579416, EPI_ISL_8579419, EPI_ISL_8579426, EPI_ISL_8579429, EPI_ISL_8579440, EPI_ISL_8579446, EPI_ISL_8579447, EPI_ISL_8579451, EPI_ISL_8579459, EPI_ISL_8579460, EPI_ISL_8579462, EPI_ISL_8579474, EPI_ISL_8579476, EPI_ISL_8579479, EPI_ISL_8579490, EPI_ISL_8579500, EPI_ISL_8579501, EPI_ISL_8579514, EPI_ISL_8579519, EPI_ISL_8579521, EPI_ISL_8579540, EPI_ISL_8579551, EPI_ISL_8579556, EPI_ISL_8579557, EPI_ISL_8579564, EPI_ISL_8579566, EPI_ISL_8579576, EPI_ISL_8579586, EPI_ISL_8579587, EPI_ISL_8579588, EPI_ISL_8579599, EPI_ISL_8579600, EPI_ISL_8579602, EPI_ISL_8579605, EPI_ISL_8579606, EPI_ISL_8579610, EPI_ISL_8579614, EPI_ISL_8579619, EPI_ISL_8579634, EPI_ISL_8579641, EPI_ISL_8579644, EPI_ISL_8579645, EPI_ISL_8579649, EPI_ISL_8579657, EPI_ISL_8579944, EPI_ISL_8580301, EPI_ISL_8580507, EPI_ISL_8580513, EPI_ISL_8580529, EPI_ISL_8580557, EPI_ISL_8580558, EPI_ISL_8580559, EPI_ISL_8580560, EPI_ISL_8580561, EPI_ISL_8580566, EPI_ISL_8580570, EPI_ISL_8580584, EPI_ISL_8580606, EPI_ISL_8580610, EPI_ISL_8580617, EPI_ISL_8580639, EPI_ISL_8580641, EPI_ISL_8580653, EPI_ISL_8580671, EPI_ISL_8580677, EPI_ISL_8580691, EPI_ISL_8580699, EPI_ISL_8580709, EPI_ISL_8580715, EPI_ISL_8580717, EPI_ISL_8580728, EPI_ISL_8580749, EPI_ISL_8580753, EPI_ISL_8580760, EPI_ISL_8580762, EPI_ISL_8580770, EPI_ISL_8580775, EPI_ISL_8580795, EPI_ISL_8580801, EPI_ISL_8580806, EPI_ISL_8580810, EPI_ISL_8580822, EPI_ISL_8580826, EPI_ISL_8580841, EPI_ISL_8580845, EPI_ISL_8580846, EPI_ISL_8580847, EPI_ISL_8580853, EPI_ISL_8580866, EPI_ISL_8580871, EPI_ISL_8580880, EPI_ISL_8580881, EPI_ISL_8580886, EPI_ISL_8580892, EPI_ISL_8580894, EPI_ISL_8580898, EPI_ISL_8580900, EPI_ISL_8580904, EPI_ISL_8580908, EPI_ISL_8580912, EPI_ISL_8580917, EPI_ISL_8580919, EPI_ISL_8580929, EPI_ISL_8580933, EPI_ISL_8580944, EPI_ISL_8580948, EPI_ISL_8580950, EPI_ISL_8580958, EPI_ISL_8580961, EPI_ISL_8580970, EPI_ISL_8580994, EPI_ISL_8581005, EPI_ISL_8581009, EPI_ISL_8581019, EPI_ISL_8581045, EPI_ISL_8581050, EPI_ISL_8581070, EPI_ISL_8581079, EPI_ISL_8581081, EPI_ISL_8581091, EPI_ISL_8581092, EPI_ISL_8581097, EPI_ISL_8581100, EPI_ISL_8581103, EPI_ISL_8581142, EPI_ISL_8581143, EPI_ISL_8581145, EPI_ISL_8581150, EPI_ISL_8581152, EPI_ISL_8581153, EPI_ISL_8581154, EPI_ISL_8581161, EPI_ISL_8581167, EPI_ISL_8581183, EPI_ISL_8581186, EPI_ISL_8581192, EPI_ISL_8581195, EPI_ISL_8581199, EPI_ISL_8581201, EPI_ISL_8581206, EPI_ISL_8581208, EPI_ISL_8581210, EPI_ISL_8581212, EPI_ISL_8581222, EPI_ISL_8581227, EPI_ISL_8581229, EPI_ISL_8581232, EPI_ISL_8581245, EPI_ISL_8581254, EPI_ISL_8581256, EPI_ISL_8581260, EPI_ISL_8581263, EPI_ISL_8581265, EPI_ISL_8581272, EPI_ISL_8581275, EPI_ISL_8581277, EPI_ISL_8581286, EPI_ISL_8581291, EPI_ISL_8581302, EPI_ISL_8581317, EPI_ISL_8581323, EPI_ISL_8581331, EPI_ISL_8581334, EPI_ISL_8581339, EPI_ISL_8581341, EPI_ISL_8581344, EPI_ISL_8581347, EPI_ISL_8581348, EPI_ISL_8581351, EPI_ISL_8581353, EPI_ISL_8581358, EPI_ISL_8581361, EPI_ISL_8581373, EPI_ISL_8581376, EPI_ISL_8581386, EPI_ISL_8581388, EPI_ISL_8581421, EPI_ISL_8581426			
see above	Department of Bacteria, Parasites and Fungi, Statens Serum Institut, Copenhagen, Denmark	Statens Serum Institut Bioinformatics and Microbial Genomics	Danish Covid-19 Genome Consortium
EPI_ISL_8578469	Department of Clinical Microbiology and Center for Genomic Medicine, Rigshospitalet, Copenhagen, Denmark	Statens Serum Institut Bioinformatics and Microbial Genomics	Danish Covid-19 Genome Consortium
EPI_ISL_8565890, EPI_ISL_8565986, EPI_ISL_8566049, EPI_ISL_8566068, EPI_ISL_8566070, EPI_ISL_8566074, EPI_ISL_8566093, EPI_ISL_8566124, EPI_ISL_8566163, EPI_ISL_8566164, EPI_ISL_8566165, EPI_ISL_8566166, EPI_ISL_8566185, EPI_ISL_8566201, EPI_ISL_8566202, EPI_ISL_8566204, EPI_ISL_8566205, EPI_ISL_8566206, EPI_ISL_8566208, EPI_ISL_8566219, EPI_ISL_8566246, EPI_ISL_8566247, EPI_ISL_8566248, EPI_ISL_8566249, EPI_ISL_8568575, EPI_ISL_8568637, EPI_ISL_8568644, EPI_ISL_8568648, EPI_ISL_8568685, EPI_ISL_8568711, EPI_ISL_8568716, EPI_ISL_8568719, EPI_ISL_8568737, EPI_ISL_8568757, EPI_ISL_8568842, EPI_ISL_8568853, EPI_ISL_8568868, EPI_ISL_8568887, EPI_ISL_8568910, EPI_ISL_8568927, EPI_ISL_8568932, EPI_ISL_8568939, EPI_ISL_8568951, EPI_ISL_8568961, EPI_ISL_8568962, EPI_ISL_8568972			
see above	Institute of Microbiology and Immunology, Faculty of Medicine, University of Ljubljana	Institute of Microbiology and Immunology, Faculty of Medicine, University of Ljubljana	Alen Suljič; Andraž Celar; Domen Lazar; Doroteja Vlaj; Mario Poljak; Miša Korva; Patricija Pozvek; Samo Zakotnik; Tatjana Avšič – Županc; Tina Gabrovšek; Tina Živič; Tomaž Mark Zorec; Špela Pleh
EPI_ISL_8576550, EPI_ISL_8576583, EPI_ISL_8576592, EPI_ISL_8576739, EPI_ISL_8576806, EPI_ISL_8576817, EPI_ISL_8576841, EPI_ISL_8576860, EPI_ISL_8576897, EPI_ISL_8576928, EPI_ISL_8576955, EPI_ISL_8576982, EPI_ISL_8577004, EPI_ISL_8577008, EPI_ISL_8577051, EPI_ISL_8577066			
see above	Molecular Biology Laboratory, Vidant Medical Center	Brody Integrative Genomics Core, East Carolina University	Changhong Yin; Heather Duncan; John T. Fallon; Kimberly P. Briley; Weihua Huang
EPI_ISL_8562554, EPI_ISL_8562558, EPI_ISL_8562560, EPI_ISL_8562561, EPI_ISL_8562562, EPI_ISL_8562566, EPI_ISL_8562592, EPI_ISL_8562593, EPI_ISL_8562594, EPI_ISL_8562638, EPI_ISL_8562640, EPI_ISL_8562661, EPI_ISL_8562665, EPI_ISL_8566891			
see above	National Public Health Laboratory, National Centre for Infectious Diseases	National Public Health Laboratory, National Centre for Infectious Diseases	Benny Yeo; Lin Cui; Raymond Tzer Pin Lin; Royce Ang; Samuel Loo; Zhenyang Zhou
EPI_ISL_8559646, EPI_ISL_8559670, EPI_ISL_8559900, EPI_ISL_8560013, EPI_ISL_8560180, EPI_ISL_8560294, EPI_ISL_8560301, EPI_ISL_8560339, EPI_ISL_8560340, EPI_ISL_8560342, EPI_ISL_8560357, EPI_ISL_8560423, EPI_ISL_8560425, EPI_ISL_8560428, EPI_ISL_8560490, EPI_ISL_8560498, EPI_ISL_8560500			
see above	SARS-CoV-2 testing team, National Institute of Infectious Diseases	Pathogen Genomics Center, National Institute of Infectious Diseases	Hazuka Y Furihata; Kentaro Itokawa; Makoto Kuroda; Masanori Hashino; Masumichi Saito; Naomi Nojiri; Nozomu Hanaoka; Rina Tanaka; Tsuguto Fujimoto; Tsuyoshi Sekizuka
EPI_ISL_8565215	University of New Mexico Hospital	Center for Global Health, University of New Mexico Health Sciences Center	Darrell Dinwiddie; Daryl Domman; Jesse Young; Jon Femling; Justin Bacca; Kurt Schwalm; Valerie Morley

We gratefully acknowledge the following Authors from the Originating laboratories responsible for obtaining the specimens, as well as the Submitting laboratories where the genome data were generated and shared via GISAID, on which this research is based.

All Submitters of data may be contacted directly via www.gisaid.org

Authors are sorted alphabetically.

Accession ID	Originating Laboratory	Submitting Laboratory	Authors
EPI_ISL_8593661	City of Milwaukee Health Department Laboratory	City of Milwaukee Health Department Laboratory	Amy Bauer; Joshua Weiner; Manjeet Khubbar; Nandhakumar Balakrishnan; Sanjib Bhattacharyya
EPI_ISL_8594433, EPI_ISL_8594435, EPI_ISL_8594436, EPI_ISL_8594437	Cliniques universitaires Saint-Luc	UCLouvain/IREC/MBLG-CTMA	Benoit Kabamba Mukadi; Bertrand Bearzatto; Jean-Luc Gala; Nicolas Pinte; Paul Blanpain; Simon Ophélie; Valentin Coste
EPI_ISL_8583616, EPI_ISL_8583617, EPI_ISL_8583622, EPI_ISL_8583623, EPI_ISL_8583628, EPI_ISL_8583629, EPI_ISL_8583631, EPI_ISL_8583633, EPI_ISL_8583638, EPI_ISL_8583640, EPI_ISL_8583641, EPI_ISL_8583642, EPI_ISL_8583646, EPI_ISL_8583654, EPI_ISL_8583670, EPI_ISL_8583675, EPI_ISL_8583683, EPI_ISL_8583686, EPI_ISL_8583690, EPI_ISL_8583701, EPI_ISL_8583704, EPI_ISL_8583707, EPI_ISL_8583709, EPI_ISL_8583710, EPI_ISL_8583714, EPI_ISL_8583718, EPI_ISL_8583719, EPI_ISL_8583720, EPI_ISL_8583729, EPI_ISL_8583733, EPI_ISL_8583739, EPI_ISL_8583744			
see above	Department of Bacteria, Parasites and Fungi, Statens Serum Institut, Copenhagen, Denmark	Statens Serum Institut Bioinformatics and Microbial Genomics	Danish Covid-19 Genome Consortium
EPI_ISL_8596354	Edmonton Provincial Lab	Public Health Agency of Canada (PHAC) National Microbiology Laboratory	Buss; Croxen M; Deo A; Dieu P; E; Ferrato C; Gill K; Khan F; Koleva P; Li V; Lloyd C; Lynch T; Ma R; Murphy S; Pabbaraju K; Shokoples S; Thayer J; Tipples G; Whitehouse M; Wong A; Yu C; Zelyas N
EPI_ISL_8593220	Fulgent Genetics	Centers for Disease Control and Prevention Division of Viral Diseases, Pathogen Discovery	Becky Tsai; Benafsh Sapra; Benjamin Rambo-Martin; Christopher Gulvick; Clinton Paden; Dakota Howard; Dhwani Batra; Doreen Ng; Duncan MacCannell; Erisa Sula; Harry Gao; James Xie; Jason Caravas; John Gao; Joseph Fierro; Kristine Lacek; Matthew Schmerer; Mickey Li; Peter Cook; Scott Sammons; Shatavia Morrison; Tymeckia Kendall; Victoria Caban Figueroa; Yan Meng; Yvette Unoarumhi
EPI_ISL_8589447	Jessa	Jessa	Severine Berden et al. on behalf of the Jessa_cmdLab
EPI_ISL_8593606, EPI_ISL_8593607, EPI_ISL_8593608	Kansas Health and Environmental Lab	Kansas Health and Environmental Lab	Amanda Bradley; Carrie Welch; Gary Buruss; Jonathan Barnell; Mike Grose; Victor Anderson; and Phil Adam
EPI_ISL_8591441, EPI_ISL_8591446, EPI_ISL_8591447	Michigan Department of Health and Human Services, Bureau of Laboratories	Michigan Department of Health and Human Services, Bureau of Laboratories	Blankenship HM; Riner D; Soehnlen MK
EPI_ISL_8584134	Mon Health Medical Center	WVU and Marshall University Combined Genomics Core Facilities	James Denvir; Peter Perrotta; Peter Stoilov; Ryan Percifield; Wesley Kimble
EPI_ISL_8593976	Pandemic Response Lab - NYC	Pandemic Response Lab, R&D	Alex Carpio; Cybill del Castillo; Dylan Law; Haiping Hao; Henry Lee; Isabel Fernandez Escapa; Jon Laurent; Melissa Hopkins; Michael Hammerling; Pradeep Bugga; Shinyoung Clair Kang; Sol Rey; William Ward
EPI_ISL_8594403	Regional Medical Sciences Center 7 Khon Kaen	National Institute of Health, Department of Medical Sciences, Ministry of Public Health, Thailand	Archawin Rojanawiwat; Natchaya Khiadsang; Nuttida Thongpramul; Pakorn Piromtong; Pilailuk Okada; Sirikanda Wimol; Siripaporn Phuygun; Sunthareeya Waicharoen; Suratchana Mitrat; Thanutsapa Thanadachakul
EPI_ISL_8594402	World Medical Hospital	National Institute of Health, Department of Medical Sciences, Ministry of Public Health, Thailand	Archawin Rojanawiwat; Natchaya Khiadsang; Nuttida Thongpramul; Pakorn Piromtong; Pilailuk Okada; Sirikanda Wimol; Siripaporn Phuygun; Sunthareeya Waicharoen; Suratchana Mitrat; Thanutsapa Thanadachakul

We gratefully acknowledge the following Authors from the Originating laboratories responsible for obtaining the specimens, as well as the Submitting laboratories where the genome data were generated and shared via GISAID, on which this research is based.

All Submitters of data may be contacted directly via www.gisaid.org

Authors are sorted alphabetically.

Acknowledgement EPI_SET Identifier: EPI_SET_20220527en

Accession ID	Originating Laboratory	Submitting Laboratory	Authors
EPI_ISL_8583325, EPI_ISL_8583331, EPI_ISL_8583345, EPI_ISL_8583348, EPI_ISL_8583349, EPI_ISL_8583351, EPI_ISL_8583359, EPI_ISL_8583360, EPI_ISL_8583363, EPI_ISL_8583376, EPI_ISL_8583378, EPI_ISL_8583385, EPI_ISL_8583395, EPI_ISL_8583398, EPI_ISL_8583416, EPI_ISL_8583423, EPI_ISL_8583434, EPI_ISL_8583435, EPI_ISL_8583436, EPI_ISL_8583450, EPI_ISL_8583460, EPI_ISL_8583468, EPI_ISL_8583476, EPI_ISL_8583483, EPI_ISL_8583487, EPI_ISL_8583488, EPI_ISL_8583500, EPI_ISL_8583509, EPI_ISL_8583516, EPI_ISL_8583518, EPI_ISL_8583520, EPI_ISL_8583527, EPI_ISL_8583544, EPI_ISL_8583547, EPI_ISL_8583553, EPI_ISL_8583555, EPI_ISL_8583562, EPI_ISL_8583565, EPI_ISL_8583571, EPI_ISL_8583576, EPI_ISL_8583580, EPI_ISL_8583583, EPI_ISL_8583584, EPI_ISL_8583588, EPI_ISL_8583592, EPI_ISL_8583595, EPI_ISL_8583596, EPI_ISL_8583597, EPI_ISL_8583598, EPI_ISL_8583604			
see above	Department of Bacteria, Parasites and Fungi, Statens Serum Institut, Copenhagen, Denmark	Statens Serum Institut Bioinformatics and Microbial Genomics	Danish Covid-19 Genome Consortium

We gratefully acknowledge the following Authors from the Originating laboratories responsible for obtaining the specimens, as well as the Submitting laboratories where the genome data were generated and shared via GISAID, on which this research is based.

All Submitters of data may be contacted directly via www.gisaid.org

Authors are sorted alphabetically.

Accession ID	Originating Laboratory	Submitting Laboratory	Authors
EPI_ISL_8582960, EPI_ISL_8582962, EPI_ISL_8582976, EPI_ISL_8582981, EPI_ISL_8582987, EPI_ISL_8582990, EPI_ISL_8582991, EPI_ISL_8582992, EPI_ISL_8582998, EPI_ISL_8583009, EPI_ISL_8583012, EPI_ISL_8583018, EPI_ISL_8583022, EPI_ISL_8583036, EPI_ISL_8583038, EPI_ISL_8583043, EPI_ISL_8583047, EPI_ISL_8583061, EPI_ISL_8583064, EPI_ISL_8583067, EPI_ISL_8583081, EPI_ISL_8583103, EPI_ISL_8583110, EPI_ISL_8583116, EPI_ISL_8583130, EPI_ISL_8583131, EPI_ISL_8583138, EPI_ISL_8583139, EPI_ISL_8583145, EPI_ISL_8583146, EPI_ISL_8583158, EPI_ISL_8583161, EPI_ISL_8583162, EPI_ISL_8583163, EPI_ISL_8583169, EPI_ISL_8583171, EPI_ISL_8583173, EPI_ISL_8583175, EPI_ISL_8583192, EPI_ISL_8583194, EPI_ISL_8583203, EPI_ISL_8583213, EPI_ISL_8583222, EPI_ISL_8583229, EPI_ISL_8583230, EPI_ISL_8583240, EPI_ISL_8583293, EPI_ISL_8583296, EPI_ISL_8583297, EPI_ISL_8583315			
see above	Department of Bacteria, Parasites and Fungi, Statens Serum Institut, Copenhagen, Denmark	Statens Serum Institut Bioinformatics and Microbial Genomics	Danish Covid-19 Genome Consortium

We gratefully acknowledge the following Authors from the Originating laboratories responsible for obtaining the specimens, as well as the Submitting laboratories where the genome data were generated and shared via GISAID, on which this research is based.

All Submitters of data may be contacted directly via www.gisaid.org

Authors are sorted alphabetically.

Accession ID	Originating Laboratory	Submitting Laboratory	Authors
EPI_ISL_8582673, EPI_ISL_8582674, EPI_ISL_8582684, EPI_ISL_8582693, EPI_ISL_8582698, EPI_ISL_8582700, EPI_ISL_8582701, EPI_ISL_8582706, EPI_ISL_8582720, EPI_ISL_8582723, EPI_ISL_8582725, EPI_ISL_8582727, EPI_ISL_8582733, EPI_ISL_8582744, EPI_ISL_8582751, EPI_ISL_8582753, EPI_ISL_8582765, EPI_ISL_8582766, EPI_ISL_8582768, EPI_ISL_8582772, EPI_ISL_8582775, EPI_ISL_8582777, EPI_ISL_8582782, EPI_ISL_8582785, EPI_ISL_8582786, EPI_ISL_8582788, EPI_ISL_8582795, EPI_ISL_8582801, EPI_ISL_8582805, EPI_ISL_8582818, EPI_ISL_8582821, EPI_ISL_8582828, EPI_ISL_8582853, EPI_ISL_8582855, EPI_ISL_8582859, EPI_ISL_8582863, EPI_ISL_8582869, EPI_ISL_8582877, EPI_ISL_8582881, EPI_ISL_8582891, EPI_ISL_8582893, EPI_ISL_8582914, EPI_ISL_8582918, EPI_ISL_8582919, EPI_ISL_8582921, EPI_ISL_8582926, EPI_ISL_8582932, EPI_ISL_8582934, EPI_ISL_8582943, EPI_ISL_8582944			
see above	Department of Bacteria, Parasites and Fungi, Statens Serum Institut, Copenhagen, Denmark	Statens Serum Institut Bioinformatics and Microbial Genomics	Danish Covid-19 Genome Consortium

We gratefully acknowledge the following Authors from the Originating laboratories responsible for obtaining the specimens, as well as the Submitting laboratories where the genome data were generated and shared via GISAID, on which this research is based.

All Submitters of data may be contacted directly via www.gisaid.org

Authors are sorted alphabetically.

Accession ID	Originating Laboratory	Submitting Laboratory	Authors
EPI_ISL_8582400, EPI_ISL_8582405, EPI_ISL_8582414, EPI_ISL_8582415, EPI_ISL_8582435, EPI_ISL_8582440, EPI_ISL_8582443, EPI_ISL_8582444, EPI_ISL_8582449, EPI_ISL_8582450, EPI_ISL_8582456, EPI_ISL_8582458, EPI_ISL_8582469, EPI_ISL_8582482, EPI_ISL_8582484, EPI_ISL_8582487, EPI_ISL_8582497, EPI_ISL_8582503, EPI_ISL_8582504, EPI_ISL_8582510, EPI_ISL_8582516, EPI_ISL_8582518, EPI_ISL_8582527, EPI_ISL_8582530, EPI_ISL_8582534, EPI_ISL_8582536, EPI_ISL_8582541, EPI_ISL_8582547, EPI_ISL_8582550, EPI_ISL_8582551, EPI_ISL_8582557, EPI_ISL_8582564, EPI_ISL_8582567, EPI_ISL_8582577, EPI_ISL_8582579, EPI_ISL_8582583, EPI_ISL_8582594, EPI_ISL_8582603, EPI_ISL_8582605, EPI_ISL_8582606, EPI_ISL_8582612, EPI_ISL_8582622, EPI_ISL_8582626, EPI_ISL_8582633, EPI_ISL_8582637, EPI_ISL_8582639, EPI_ISL_8582645, EPI_ISL_8582650, EPI_ISL_8582657, EPI_ISL_8582668			
see above	Department of Bacteria, Parasites and Fungi, Statens Serum Institut, Copenhagen, Denmark	Statens Serum Institut Bioinformatics and Microbial Genomics	Danish Covid-19 Genome Consortium

We gratefully acknowledge the following Authors from the Originating laboratories responsible for obtaining the specimens, as well as the Submitting laboratories where the genome data were generated and shared via GISAID, on which this research is based.

All Submitters of data may be contacted directly via www.gisaid.org

Authors are sorted alphabetically.

Acknowledgement EPI_SET Identifier: EPI_SET_20220527sq

Accession ID	Originating Laboratory	Submitting Laboratory	Authors
EPI_ISL_8582142, EPI_ISL_8582172, EPI_ISL_8582178, EPI_ISL_8582187, EPI_ISL_8582188, EPI_ISL_8582200, EPI_ISL_8582202, EPI_ISL_8582204, EPI_ISL_8582214, EPI_ISL_8582217, EPI_ISL_8582218, EPI_ISL_8582219, EPI_ISL_8582224, EPI_ISL_8582238, EPI_ISL_8582240, EPI_ISL_8582244, EPI_ISL_8582245, EPI_ISL_8582247, EPI_ISL_8582248, EPI_ISL_8582249, EPI_ISL_8582252, EPI_ISL_8582258, EPI_ISL_8582259, EPI_ISL_8582265, EPI_ISL_8582271, EPI_ISL_8582272, EPI_ISL_8582273, EPI_ISL_8582274, EPI_ISL_8582275, EPI_ISL_8582279, EPI_ISL_8582290, EPI_ISL_8582296, EPI_ISL_8582304, EPI_ISL_8582307, EPI_ISL_8582308, EPI_ISL_8582318, EPI_ISL_8582321, EPI_ISL_8582327, EPI_ISL_8582328, EPI_ISL_8582329, EPI_ISL_8582330, EPI_ISL_8582337, EPI_ISL_8582344, EPI_ISL_8582347, EPI_ISL_8582351, EPI_ISL_8582357, EPI_ISL_8582358, EPI_ISL_8582362, EPI_ISL_8582378, EPI_ISL_8582393			
see above	Department of Bacteria, Parasites and Fungi, Statens Serum Institut, Copenhagen, Denmark	Statens Serum Institut Bioinformatics and Microbial Genomics	Danish Covid-19 Genome Consortium

We gratefully acknowledge the following Authors from the Originating laboratories responsible for obtaining the specimens, as well as the Submitting laboratories where the genome data were generated and shared via GISAID, on which this research is based.

All Submitters of data may be contacted directly via www.gisaid.org

Authors are sorted alphabetically.

Accession ID	Originating Laboratory	Submitting Laboratory	Authors
EPI_ISL_8582031, EPI_ISL_8582034, EPI_ISL_8582057, EPI_ISL_8582091, EPI_ISL_8582107, EPI_ISL_8582113, EPI_ISL_8582136, EPI_ISL_8582139			
see above	Department of Bacteria, Parasites and Fungi, Statens Serum Institut, Copenhagen, Denmark	Statens Serum Institut Bioinformatics and Microbial Genomics	Danish Covid-19 Genome Consortium

We gratefully acknowledge the following Authors from the Originating laboratories responsible for obtaining the specimens, as well as the Submitting laboratories where the genome data were generated and shared via GISAID, on which this research is based.

All Submitters of data may be contacted directly via www.gisaid.org

Authors are sorted alphabetically.

Accession ID	Originating Laboratory	Submitting Laboratory	Authors
EPI_ISL_8604897, EPI_ISL_8604898, EPI_ISL_8604899, EPI_ISL_8604900, EPI_ISL_8604905, EPI_ISL_8604908, EPI_ISL_8604909, EPI_ISL_8604913, EPI_ISL_8604914, EPI_ISL_8604915, EPI_ISL_8604917, EPI_ISL_8604918, EPI_ISL_8604919, EPI_ISL_8604920, EPI_ISL_8604922, EPI_ISL_8604926, EPI_ISL_8604927, EPI_ISL_8604928, EPI_ISL_8604929, EPI_ISL_8604930, EPI_ISL_8604931, EPI_ISL_8604932, EPI_ISL_8604933, EPI_ISL_8604934, EPI_ISL_8604936			
see above	AHRI	CERI, Centre for Epidemic Response and Innvoation, Stellenbosch University and KRISP, KZN Research Innovation and Sequencing Platform, UKZN.	Bernstein M; Cele S; Giandhari J; Karim F; Khan K; Moir M; Naidoo Y; Pillay S; Ramphal U; Ramphal Y; San JE; Sigal A; Tegally H; Tshiabuila D; Wilkinson E; de Oliveira T; van Wyk S
EPI_ISL_8607974, EPI_ISL_8608035, EPI_ISL_8608121	Botswana Harvard HIV Reference Laboratory	Botswana Harvard HIV Reference Laboratory	Boitumelo Zuze; Botshelo Radibe; Dorcas Maruapula; Joseph Makhema; Keoratile Ntshambiwa; Kgomotso Moruisi; Legodile Kooepile; Mosepele Mosepele; Mphaphi B. Mbulawa; Ontlametse T. Bareng; Pamela Smith-Lawrence; Roger Shapiro; Sefetogi Ramaologa; Shahin Lockman; Sikhulile Moyo; Simani Gaseitsiwe; Thongbotho Mphoyakgosi; Wonderful T. Choga
EPI_ISL_8610825, EPI_ISL_8610838, EPI_ISL_8610866, EPI_ISL_8610875, EPI_ISL_8610879, EPI_ISL_8610887, EPI_ISL_8610904, EPI_ISL_8610948, EPI_ISL_8610955, EPI_ISL_8610958, EPI_ISL_8610972, EPI_ISL_8610981, EPI_ISL_8610986, EPI_ISL_8611012, EPI_ISL_8611027, EPI_ISL_8611046, EPI_ISL_8611058, EPI_ISL_8611061, EPI_ISL_8611069, EPI_ISL_8611085, EPI_ISL_8611124, EPI_ISL_8611140, EPI_ISL_8611164, EPI_ISL_8611173, EPI_ISL_8611208, EPI_ISL_8611258, EPI_ISL_8611275, EPI_ISL_8611293, EPI_ISL_8611321, EPI_ISL_8611339, EPI_ISL_8611349, EPI_ISL_8611361, EPI_ISL_8611365, EPI_ISL_8611377, EPI_ISL_8611379, EPI_ISL_8611394, EPI_ISL_8611453, EPI_ISL_8611493, EPI_ISL_8611500, EPI_ISL_8611528, EPI_ISL_8611532, EPI_ISL_8611540, EPI_ISL_8611547, EPI_ISL_8611550, EPI_ISL_8611569, EPI_ISL_8611599, EPI_ISL_8611605, EPI_ISL_8611616, EPI_ISL_8611629, EPI_ISL_8611641, EPI_ISL_8611655, EPI_ISL_8611658, EPI_ISL_8611667, EPI_ISL_8611670, EPI_ISL_8611685, EPI_ISL_8611690, EPI_ISL_8611698, EPI_ISL_8611735, EPI_ISL_8611759, EPI_ISL_8611770, EPI_ISL_8611775, EPI_ISL_8611778, EPI_ISL_8611783, EPI_ISL_8611787, EPI_ISL_8611796, EPI_ISL_8611805, EPI_ISL_8611810, EPI_ISL_8611816, EPI_ISL_8611818, EPI_ISL_8611819, EPI_ISL_8611827, EPI_ISL_8611840, EPI_ISL_8611857, EPI_ISL_8611864, EPI_ISL_8611889, EPI_ISL_8611895, EPI_ISL_8611898, EPI_ISL_8611904, EPI_ISL_8611913, EPI_ISL_8611917, EPI_ISL_8612031, EPI_ISL_8612035, EPI_ISL_8612043, EPI_ISL_8612056, EPI_ISL_8612070, EPI_ISL_8612074, EPI_ISL_8612078, EPI_ISL_8612089, EPI_ISL_8612100, EPI_ISL_8612111, EPI_ISL_8612159, EPI_ISL_8612161, EPI_ISL_8612167, EPI_ISL_8612171, EPI_ISL_8612183, EPI_ISL_8612200, EPI_ISL_8612203, EPI_ISL_8612216, EPI_ISL_8612224, EPI_ISL_8612271, EPI_ISL_8612275, EPI_ISL_8612284, EPI_ISL_8612301, EPI_ISL_8612306, EPI_ISL_8612323, EPI_ISL_8612325, EPI_ISL_8612341, EPI_ISL_8612346, EPI_ISL_8612371, EPI_ISL_8612379, EPI_ISL_8612410, EPI_ISL_8612412, EPI_ISL_8612430, EPI_ISL_8612450, EPI_ISL_8612465, EPI_ISL_8612472, EPI_ISL_8612491, EPI_ISL_8612500, EPI_ISL_8612529, EPI_ISL_8612531, EPI_ISL_8612533, EPI_ISL_8612540, EPI_ISL_8612546, EPI_ISL_8612547, EPI_ISL_8612573, EPI_ISL_8612577, EPI_ISL_8612580, EPI_ISL_8612583, EPI_ISL_8612588, EPI_ISL_8612591, EPI_ISL_8612607, EPI_ISL_8612617, EPI_ISL_8612649, EPI_ISL_8612662, EPI_ISL_8612704, EPI_ISL_8612726, EPI_ISL_8612730, EPI_ISL_8612744, EPI_ISL_8612754, EPI_ISL_8612776, EPI_ISL_8612778, EPI_ISL_8612795, EPI_ISL_8612806, EPI_ISL_8613830, EPI_ISL_8613832, EPI_ISL_8613872, EPI_ISL_8613888, EPI_ISL_8613902, EPI_ISL_8613930, EPI_ISL_8613938, EPI_ISL_8613939, EPI_ISL_8613959, EPI_ISL_8613969, EPI_ISL_8613979, EPI_ISL_8613987, EPI_ISL_8614023, EPI_ISL_8614027, EPI_ISL_8614034, EPI_ISL_8614042, EPI_ISL_8614054, EPI_ISL_8614071, EPI_ISL_8614088, EPI_ISL_8614106, EPI_ISL_8614126, EPI_ISL_8614131, EPI_ISL_8614150, EPI_ISL_8614183, EPI_ISL_8614234, EPI_ISL_8614245, EPI_ISL_8614288, EPI_ISL_8614339, EPI_ISL_8614365, EPI_ISL_8614369, EPI_ISL_8614409, EPI_ISL_8614444, EPI_ISL_8614448, EPI_ISL_8614452, EPI_ISL_8614472, EPI_ISL_8614495, EPI_ISL_8614501, EPI_ISL_8614508, EPI_ISL_8614518, EPI_ISL_8614537, EPI_ISL_8614545, EPI_ISL_8614560, EPI_ISL_8614590, EPI_ISL_8614615, EPI_ISL_8614619, EPI_ISL_8614634, EPI_ISL_8614658, EPI_ISL_8614664, EPI_ISL_8614668, EPI_ISL_8614710, EPI_ISL_8614749, EPI_ISL_8614753, EPI_ISL_8614763, EPI_ISL_8614780, EPI_ISL_8614788, EPI_ISL_8614791, EPI_ISL_8614808, EPI_ISL_8614812, EPI_ISL_8614885			
see above	Department of Bacteria, Parasites and Fungi, Statens Serum Institut, Copenhagen, Denmark	Statens Serum Institut Bioinformatics and Microbial Genomics	Danish Covid-19 Genome Consortium
EPI_ISL_8623424	Department of Clinical Microbiology	GIGA Medical Genomics	Bouchra Boujemla; Claire Gourzonès; Cécile Meex; Keith Durkin; Laurent Gillet; Maria Artesi; Marie-Pierre Hayette; Nadine Cambisano; Nathalie Renotte; Olivier Ek; Sébastien Bontems; Vincent Bours
EPI_ISL_8607570, EPI_ISL_8607573, EPI_ISL_8607605, EPI_ISL_8607606, EPI_ISL_8607607, EPI_ISL_8607608, EPI_ISL_8607609, EPI_ISL_8608254, EPI_ISL_8608268, EPI_ISL_8625485, EPI_ISL_8625586, EPI_ISL_8629990, EPI_ISL_8631251			
see above	Fulgent Genetics	Centers for Disease Control and Prevention Division of Viral Diseases, Pathogen Discovery	Becky Tsai; Benafsh Sapra; Benjamin Rambo-Martin; Christopher Gulvick; Clinton Paden; Dakota Howard; Dhwani Batra; Doreen Ng; Duncan MacCannell; Erisa Sula; Harry Gao; James Xie; Jason Caravas; John Gao; Joseph Fierro; Kristine Lacek; Matthew Schmerer; Mickey Li; Peter Cook; Scott Sammons; Shatavia Morrison; Tymeckia Kendall; Victoria Caban Figueroa; Yan Meng; Yvette Unoarumhi
EPI_ISL_8605571	GENOME CENTER, Jashore University of Science and Technology	GENOME CENTER, Jashore University of Science and Technology	Hassan M. Al-Emran; Iqbal Kabir Jahid; Md. Ali Ahasan Setu; Md. Anwar Hossain; Md. Rasel Parvez; Md. Shaminur Rahman Prosanto Kumar Das; Md. Shazid Hasan; Shovon Lal Sarkar; Toukir Ahammed
EPI_ISL_8605841	GENOME CENTER, Jashore University of Science and Technology.	GENOME CENTER, Jashore University of Science and Technology.	Hassan M. Al-Emran; Iqbal Kabir Jahid; Md. Ali Ahasan Setu; Md. Anwar Hossain; Md. Rasel Parvez; Md. Shaminur Rahman Prosanto Kumar Das; Md. Shazid Hasan; Shovon Lal Sarkar; Toukir Ahammed
EPI_ISL_8619211, EPI_ISL_8619216, EPI_ISL_8619222, EPI_ISL_8619224, EPI_ISL_8619225, EPI_ISL_8619233, EPI_ISL_8619234			
see above	Hannover Medical School, Institute of Virology	Hannover Medical School, Institute of Virology	Lars Steinbrück
EPI_ISL_8598733	Hospital Carmen Y S. Ochoa	Laboratorio de Virología HUCA	; Alba L; Alvarez-Arguelles ME; Boga JA; Costales I; Coto E; González-Alba JM; Gómez de Oña J; Martín-Rodríguez G; Melón S; Perez-Martínez Z; Rojo S; Sandoval M
EPI_ISL_8598725, EPI_ISL_8598726, EPI_ISL_8598728, EPI_ISL_8598729, EPI_ISL_8598730, EPI_ISL_8598731, EPI_ISL_8598732, EPI_ISL_8598735, EPI_ISL_8598739, EPI_ISL_8598743			
see above	Hospital Universitario Central de Asturias	Laboratorio de Virología HUCA	; Alba L; Alvarez-Arguelles ME; Boga JA; Costales I; Coto E; González-Alba JM; Gómez de Oña J; Martín-Rodríguez G; Melón S; Perez-Martínez Z; Rojo S; Sandoval M
EPI_ISL_8629108, EPI_ISL_8629109, EPI_ISL_8629110, EPI_ISL_8629111, EPI_ISL_8629112, EPI_ISL_8629113, EPI_ISL_8629114, EPI_ISL_8629115, EPI_ISL_8629116, EPI_ISL_8629117, EPI_ISL_8629118, EPI_ISL_8629119, EPI_ISL_8629120, EPI_ISL_8629121, EPI_ISL_8629123, EPI_ISL_8629124, EPI_ISL_8629125, EPI_ISL_8629126, EPI_ISL_8629127, EPI_ISL_8629128, EPI_ISL_8629129, EPI_ISL_8629130, EPI_ISL_8629131, EPI_ISL_8629132, EPI_ISL_8629133, EPI_ISL_8629134, EPI_ISL_8629135, EPI_ISL_8629136, EPI_ISL_8629137, EPI_ISL_8629139, EPI_ISL_8629140, EPI_ISL_8629141, EPI_ISL_8629142, EPI_ISL_8629143, EPI_ISL_8629144, EPI_ISL_8629145, EPI_ISL_8629146, EPI_ISL_8629147, EPI_ISL_8629148, EPI_ISL_8629149, EPI_ISL_8629152, EPI_ISL_8629153, EPI_ISL_8629154, EPI_ISL_8629155, EPI_ISL_8629156, EPI_ISL_8629157, EPI_ISL_8629158, EPI_ISL_8629159, EPI_ISL_8629160, EPI_ISL_8629161, EPI_ISL_8629162, EPI_ISL_8629163, EPI_ISL_8629164, EPI_ISL_8629165, EPI_ISL_8629166, EPI_ISL_8629167, EPI_ISL_8629168, EPI_ISL_8629169, EPI_ISL_8629170, EPI_ISL_8629171, EPI_ISL_8629172, EPI_ISL_8629173, EPI_ISL_8629228, EPI_ISL_8629229, EPI_ISL_8629230, EPI_ISL_8629232, EPI_ISL_8629233, EPI_ISL_8629234, EPI_ISL_8629236, EPI_ISL_8629237, EPI_ISL_8629238, EPI_ISL_8629248, EPI_ISL_8629249, EPI_ISL_8629250, EPI_ISL_8629251, EPI_ISL_8629254, EPI_ISL_8629256, EPI_ISL_8629260, EPI_ISL_8629261, EPI_ISL_8629262, EPI_ISL_8629263, EPI_ISL_8629264, EPI_ISL_8629265, EPI_ISL_8629266, EPI_ISL_8629267, EPI_ISL_8629268, EPI_ISL_8629269, EPI_ISL_8629270, EPI_ISL_8629272, EPI_ISL_8629273			
see above	Idaho Bureau of Laboratories	Idaho Bureau of Laboratories	"R. Beukelman; Aimee Ceniseros; Christian Loera; Christopher Ball"; Matthew Charles Burns; Robert L. Voermans
EPI_ISL_8601617, EPI_ISL_8602634, EPI_ISL_8603144, EPI_ISL_8603202, EPI_ISL_8603211	Laboratory Corporation of America	Centers for Disease Control and Prevention Division of Viral Diseases, Pathogen Discovery	Amanda Douglas; Amanda Suchanek; Andrea Throop; Ayla Burns; Benjamin Rambo-Martin; Bobbi Croy; Brian Krueger; Brian Norvell; Christopher Gulvick; Christos Petropoulos; Clinton Paden; Craig Lukasik; Dakota Howard; Debbie Boles; Dhwani Batra; Duncan MacCannell; Eyad Almasri; Goran Stevovic; Howard Engler; Hrushikesh Deshmukh; Jake Humphrey; Jana Schroth; Jason Caravas; Joe Voshell; John Pruitt; Jonathan Meltzer; Jonathan Williams; Kimberly Wagner; Kristine Lacek; Lax Iyer; Lisa Pfefferle; Lyndon Tilson; Manoj Jain; Marcia Eisenberg; Mary Cristobal; Mary Williamson; Matthew Robinson; Matthew Schmerer; Michael Levandoski; Mike Sapeta; Mindy Nye; Minoo Agarwal; Mohan Kolli; Nuthawin Charoensri; Oren Cohen; Peter Cook; Prashant Gupta; Qian Zeng; Rama Ghatti; Scott Parker; Scott Ryan; Scott Sammons; Shatavia Morrison; Stanley Letovsky; Steven Ragan; Suresh Selvaraju; Susan Countryman; Susan Hicks; Suzanne Dale; Thomas Urban; Tim Kuphal; Tricia Zwiefelhofer; Tymeckia Kendall; Victoria Caban Figueroa; Vincent Drouillon; Yvette Unoarumhi
EPI_ISL_8623347	National Platform bis COVID ULB-IBC	National Platform bis COVID ULB-IBC	Arnaud Marchant; Benoit Haerlingen; Coralie Henin; Marie-Luce Delforge; Ricardo De Mendonça
EPI_ISL_8597860	New South Wales Health Pathology Royal Prince Alfred Hospital	Microbiology RPAH	Au, J.; Bull, R.; Deveson, I.; Foster, C.; Rawlinson, W.; Ruiz Silva, M.; Van Hal, S.
EPI_ISL_8598240, EPI_ISL_8598245, EPI_ISL_8598248, EPI_ISL_8598252, EPI_ISL_8598254, EPI_ISL_8598265, EPI_ISL_8598266, EPI_ISL_8598275, EPI_ISL_8598278, EPI_ISL_8598279, EPI_ISL_8598281, EPI_ISL_8598284, EPI_ISL_8598288, EPI_ISL_8598289, EPI_ISL_8598305, EPI_ISL_8598309, EPI_ISL_8598310, EPI_ISL_8598312, EPI_ISL_8598331, EPI_ISL_8598332, EPI_ISL_8598338, EPI_ISL_8598353, EPI_ISL_8598360, EPI_ISL_8598361, EPI_ISL_8598363, EPI_ISL_8598364, EPI_ISL_8598365, EPI_ISL_8598366, EPI_ISL_8598374, EPI_ISL_8598379, EPI_ISL_8598383, EPI_ISL_8598388, EPI_ISL_8598391, EPI_ISL_8598394, EPI_ISL_8598396, EPI_ISL_8598397, EPI_ISL_8598405			
see above	PHV-FSS	PHV-FSS	Chenwei Wang on behalf of Q-PHIRE Genomics
EPI_ISL_8607795	PO AVEZZANO - U.O.C. Laboratorio Analisi	Istituto Zooprofilattico Sperimentale dell'Abruzzo e Molise "G. Caporale"	Ancora M; Calistri P; Cammà C; Caporale M; Curini V; Delli Compagni E; Di Domenico M; Di Lollo Valeria; Di Pasquale A; Lorusso A; Mangone I; Marcacci M; Puglia I; Rinaldi A; Savini G; Scialabba S
EPI_ISL_8603748	Pejabat Kesihatan KLIA	iPROMISE, UiTM	Ariza Adnan; Fadzilah Mohd Nor; Lim Wai Feng; Mohd Asif Mohd Sukri; Mohd Nur Fakhruzzaman Noorizhab; Mohd Zaki Salleh; Sazzli Shahlan Kassim; Siti Farah Alwani Mohd Nawi; Siti Hamimah Sheikh Abdul Kadir; Teh Lay Kek; Wang Seok Mui
EPI_ISL_8625876, EPI_ISL_8625877, EPI_ISL_8625878, EPI_ISL_8625879, EPI_ISL_8625881	Regional Hospital Liberec	Regional Hospital Liberec	Iva Dolinova; Katerina Arientova; Katerina Stillerova; Martin Kracik; Tomas Zajic
EPI_ISL_8616009	SK-Roy Romanow Provincial Laboratory	Saskatchewan - Roy Romanow Provincial Laboratory (RRPL)	Alanna Senecal; Amanda Lang; Jessica Minion; Kara Loos; Keith MacKenzie; Meredith Faires; Rachel DePaulo; Roy Romanow Provincial Laboratory - Molecular Diagnostics; Ryan McDonald
EPI_ISL_8605618, EPI_ISL_8621950, EPI_ISL_8622036	UW Virology Lab	UW Virology Lab	Alexander Greninger; Hong Xie; Isabel Arnould; Keith R Jerome; Meei-Li Huang; Nathan Breit; Patrick Mathias; Pavitra Roychoudhury; Pooneh Hajian; Ricardo Perez; Robert J. Livingston; Sean Ellis; Seffir T. Wendm; Shah Mohamed Bakhash
EPI_ISL_8620724	Utah Public Health Laboratory	Utah Public Health Laboratory	Erin L. Young; John Arnn; Kelly F. Oakeson; Olinto Linares-Perdomo; Pooja Gupta; Tom Iverson
EPI_ISL_8636462, EPI_ISL_8636482	ZARV/NHLS, Department Medical Virology, University of Pretoria	CERI, Centre for Epidemic Response and Innvoation, Stellenbosch University and KRISP, KZN Research Innovation and Sequencing Platform, UKZN.	Adriano Mendes; Amy Strydom; Giandhari J; Micheala Davids; Moir M; Naidoo Y; Pillay S; Ramphal U; Ramphal Y; San JE; Sim Mayaphi and Marietjie Venter; Tegally H; Tshiabuila D; Van Wyk S; Wilkinson E; de Oliveira T

We gratefully acknowledge the following Authors from the Originating laboratories responsible for obtaining the specimens, as well as the Submitting laboratories where the genome data were generated and shared via GISAID, on which this research is based.

All Submitters of data may be contacted directly via www.gisaid.org

Authors are sorted alphabetically.

Acknowledgement EPI_SET Identifier: EPI_SET_20220527ga

Accession ID	Originating Laboratory	Submitting Laboratory	Authors
EPI_ISL_8637800, EPI_ISL_8637814, EPI_ISL_8637817	AP SSO	CSIR-Centre for Cellular and Molecular Biology-INSACOG	Amareshwar Vodapalli; Ara Sreenivas; Archana Bharadwaj Siva; B Himasri; Divya Tej Sowpati; Jandhyala Sai Krishna; Karthik Bharadwaj Tallapaka; Lamuk Zaveri; Malini Nemalikanti; Priya Nurkurthy; Rakesh K Mishra; Shreekant Verma; Sreelekshmi MS; Sumedha Avadhanula; Surabhi Srivastava; Tulasi Nagabandi; Valli Nagalakshmi Undamatla; Vidhyadhari Methuku
EPI_ISL_8637640	CSIR-National Environmental Engineering Research Institute	CSIR-Centre for Cellular and Molecular Biology-INSACOG	Amareshwar Vodapalli; Ara Sreenivas; Archana Bharadwaj Siva; B Himasri; Divya Tej Sowpati; Karthik Bharadwaj Tallapaka; Krishna Khairnar; Lamuk Zaveri; Malini Nemalikanti; Priya Nurkurthy; Rakesh K Mishra; Shreekant Verma; Sreelekshmi MS; Sumedha Avadhanula; Surabhi Srivastava; Tulasi Nagabandi; Valli Nagalakshmi Undamatla; Vidhyadhari Methuku
EPI_ISL_8653120, EPI_ISL_8653178, EPI_ISL_8653181, EPI_ISL_8653190, EPI_ISL_8653193, EPI_ISL_8653196, EPI_ISL_8653208, EPI_ISL_8653211, EPI_ISL_8653213, EPI_ISL_8653254, EPI_ISL_8653257, EPI_ISL_8653284, EPI_ISL_8653288, EPI_ISL_8653311, EPI_ISL_8653328, EPI_ISL_8653331, EPI_ISL_8653345, EPI_ISL_8653352, EPI_ISL_8653363, EPI_ISL_8653367, EPI_ISL_8653382, EPI_ISL_8653384, EPI_ISL_8653399, EPI_ISL_8653409, EPI_ISL_8653437, EPI_ISL_8653478, EPI_ISL_8653499, EPI_ISL_8653508, EPI_ISL_8653519, EPI_ISL_8653522, EPI_ISL_8653525, EPI_ISL_8653543, EPI_ISL_8653555, EPI_ISL_8653591, EPI_ISL_8653600, EPI_ISL_8653622, EPI_ISL_8653630, EPI_ISL_8653633, EPI_ISL_8653642, EPI_ISL_8653645, EPI_ISL_8653653, EPI_ISL_8653661, EPI_ISL_8653674, EPI_ISL_8653696, EPI_ISL_8653699, EPI_ISL_8653701, EPI_ISL_8653711, EPI_ISL_8653717, EPI_ISL_8653723, EPI_ISL_8653737, EPI_ISL_8653743, EPI_ISL_8653760, EPI_ISL_8653763, EPI_ISL_8653792, EPI_ISL_8653808, EPI_ISL_8653814, EPI_ISL_8653834, EPI_ISL_8653843, EPI_ISL_8653846, EPI_ISL_8653849, EPI_ISL_8653852, EPI_ISL_8653858, EPI_ISL_8653881, EPI_ISL_8653899, EPI_ISL_8653905, EPI_ISL_8653908, EPI_ISL_8653942, EPI_ISL_8653961, EPI_ISL_8653980, EPI_ISL_8653983, EPI_ISL_8653986, EPI_ISL_8653991, EPI_ISL_8653996, EPI_ISL_8654012, EPI_ISL_8654027, EPI_ISL_8654029, EPI_ISL_8654047, EPI_ISL_8654052, EPI_ISL_8654055, EPI_ISL_8654063, EPI_ISL_8654091, EPI_ISL_8654093, EPI_ISL_8654100, EPI_ISL_8654109, EPI_ISL_8654120, EPI_ISL_8654134, EPI_ISL_8654149, EPI_ISL_8654152, EPI_ISL_8654153, EPI_ISL_8654185, EPI_ISL_8654191, EPI_ISL_8654198, EPI_ISL_8654206, EPI_ISL_8654221, EPI_ISL_8654230, EPI_ISL_8654233, EPI_ISL_8654246, EPI_ISL_8654261, EPI_ISL_8654272, EPI_ISL_8654281, EPI_ISL_8654295, EPI_ISL_8654335, EPI_ISL_8654340, EPI_ISL_8654352, EPI_ISL_8654358, EPI_ISL_8654361, EPI_ISL_8654379, EPI_ISL_8654395, EPI_ISL_8654404, EPI_ISL_8654407, EPI_ISL_8654410, EPI_ISL_8654432, EPI_ISL_8654460, EPI_ISL_8654466, EPI_ISL_8654472, EPI_ISL_8654474, EPI_ISL_8654478, EPI_ISL_8654493, EPI_ISL_8654498, EPI_ISL_8654544, EPI_ISL_8654562, EPI_ISL_8654565, EPI_ISL_8654574, EPI_ISL_8654584, EPI_ISL_8654599, EPI_ISL_8654609, EPI_ISL_8654630, EPI_ISL_8654643, EPI_ISL_8654680, EPI_ISL_8654713, EPI_ISL_8654725, EPI_ISL_8654733, EPI_ISL_8654750, EPI_ISL_8654764, EPI_ISL_8654767, EPI_ISL_8654773, EPI_ISL_8654795, EPI_ISL_8654802, EPI_ISL_8654804, EPI_ISL_8654807, EPI_ISL_8654813, EPI_ISL_8654814, EPI_ISL_8654815, EPI_ISL_8654819, EPI_ISL_8654821, EPI_ISL_8654832, EPI_ISL_8654851, EPI_ISL_8654859, EPI_ISL_8654873, EPI_ISL_8654882, EPI_ISL_8654897, EPI_ISL_8654907, EPI_ISL_8654914, EPI_ISL_8654956, EPI_ISL_8654994, EPI_ISL_8655008, EPI_ISL_8655013, EPI_ISL_8655031, EPI_ISL_8655033, EPI_ISL_8655060, EPI_ISL_8655069, EPI_ISL_8655077, EPI_ISL_8655083, EPI_ISL_8655098, EPI_ISL_8655109, EPI_ISL_8655117, EPI_ISL_8655173, EPI_ISL_8655189, EPI_ISL_8655192, EPI_ISL_8655204, EPI_ISL_8655217, EPI_ISL_8655260, EPI_ISL_8655266, EPI_ISL_8655275, EPI_ISL_8655283, EPI_ISL_8655295, EPI_ISL_8655305, EPI_ISL_8655307, EPI_ISL_8655312, EPI_ISL_8655324, EPI_ISL_8655385, EPI_ISL_8655391, EPI_ISL_8655403, EPI_ISL_8655418, EPI_ISL_8655421, EPI_ISL_8655447, EPI_ISL_8655455, EPI_ISL_8655470, EPI_ISL_8655482, EPI_ISL_8655508, EPI_ISL_8655529, EPI_ISL_8655547, EPI_ISL_8655562, EPI_ISL_8655574, EPI_ISL_8655581, EPI_ISL_8655593, EPI_ISL_8655595, EPI_ISL_8655604, EPI_ISL_8655605, EPI_ISL_8655611, EPI_ISL_8655617, EPI_ISL_8655622, EPI_ISL_8655623, EPI_ISL_8655628, EPI_ISL_8655635, EPI_ISL_8655641, EPI_ISL_8655643, EPI_ISL_8655646, EPI_ISL_8655661, EPI_ISL_8655664, EPI_ISL_8655667, EPI_ISL_8655732, EPI_ISL_8655735, EPI_ISL_8655752, EPI_ISL_8655770, EPI_ISL_8655773, EPI_ISL_8655794, EPI_ISL_8655803, EPI_ISL_8655806, EPI_ISL_8655818, EPI_ISL_8655836, EPI_ISL_8655851, EPI_ISL_8655871, EPI_ISL_8655876, EPI_ISL_8655901, EPI_ISL_8655909, EPI_ISL_8655921, EPI_ISL_8655932, EPI_ISL_8655944, EPI_ISL_8655956, EPI_ISL_8655962, EPI_ISL_8655966, EPI_ISL_8655974, EPI_ISL_8656009, EPI_ISL_8656012, EPI_ISL_8656017, EPI_ISL_8656027, EPI_ISL_8656033, EPI_ISL_8656046, EPI_ISL_8656074, EPI_ISL_8656094, EPI_ISL_8656103, EPI_ISL_8656131, EPI_ISL_8656156, EPI_ISL_8656163, EPI_ISL_8656166, EPI_ISL_8656172, EPI_ISL_8656184, EPI_ISL_8656188, EPI_ISL_8656198, EPI_ISL_8656201, EPI_ISL_8656217, EPI_ISL_8656218, EPI_ISL_8656232, EPI_ISL_8656240, EPI_ISL_8656248, EPI_ISL_8656289, EPI_ISL_8656293, EPI_ISL_8656308, EPI_ISL_8656328, EPI_ISL_8656336, EPI_ISL_8656345, EPI_ISL_8656351, EPI_ISL_8656354, EPI_ISL_8656365, EPI_ISL_8656372, EPI_ISL_8656400, EPI_ISL_8656406, EPI_ISL_8656407, EPI_ISL_8656592, EPI_ISL_8656598, EPI_ISL_8656602, EPI_ISL_8656609, EPI_ISL_8656613, EPI_ISL_8656616, EPI_ISL_8656621, EPI_ISL_8656627, EPI_ISL_8656630, EPI_ISL_8656632, EPI_ISL_8656637, EPI_ISL_8656654, EPI_ISL_8656679, EPI_ISL_8656682, EPI_ISL_8656685, EPI_ISL_8656693, EPI_ISL_8656708, EPI_ISL_8656738, EPI_ISL_8656767, EPI_ISL_8656777, EPI_ISL_8656812, EPI_ISL_8656849, EPI_ISL_8656852, EPI_ISL_8656885, EPI_ISL_8656894, EPI_ISL_8656900, EPI_ISL_8656904, EPI_ISL_8656937, EPI_ISL_8656963, EPI_ISL_8656965, EPI_ISL_8656969, EPI_ISL_8656972, EPI_ISL_8656978, EPI_ISL_8657001, EPI_ISL_8657042, EPI_ISL_8657068, EPI_ISL_8657070, EPI_ISL_8657082, EPI_ISL_8657110, EPI_ISL_8657142, EPI_ISL_8657148, EPI_ISL_8657154, EPI_ISL_8657156, EPI_ISL_8657167, EPI_ISL_8657199, EPI_ISL_8657208, EPI_ISL_8657226, EPI_ISL_8657232, EPI_ISL_8657239, EPI_ISL_8657241, EPI_ISL_8657244, EPI_ISL_8657247, EPI_ISL_8657269, EPI_ISL_8657280, EPI_ISL_8657287, EPI_ISL_8657307, EPI_ISL_8657315, EPI_ISL_8657339, EPI_ISL_8657367, EPI_ISL_8657378, EPI_ISL_8657392, EPI_ISL_8657394, EPI_ISL_8657399, EPI_ISL_8657400, EPI_ISL_8657401, EPI_ISL_8657402, EPI_ISL_8657587, EPI_ISL_8657594, EPI_ISL_8657608, EPI_ISL_8657623, EPI_ISL_8657640, EPI_ISL_8657648, EPI_ISL_8657654, EPI_ISL_8657660, EPI_ISL_8657699, EPI_ISL_8657710, EPI_ISL_8657716, EPI_ISL_8657728, EPI_ISL_8657736, EPI_ISL_8657746, EPI_ISL_8657774, EPI_ISL_8657780, EPI_ISL_8657797, EPI_ISL_8657805, EPI_ISL_8657811, EPI_ISL_8657817, EPI_ISL_8657820, EPI_ISL_8657826, EPI_ISL_8657831, EPI_ISL_8657838, EPI_ISL_8657852, EPI_ISL_8657866, EPI_ISL_8657903, EPI_ISL_8657909, EPI_ISL_8657915, EPI_ISL_8657923, EPI_ISL_8657925, EPI_ISL_8657928, EPI_ISL_8657942, EPI_ISL_8657951, EPI_ISL_8657958, EPI_ISL_8657963, EPI_ISL_8657973, EPI_ISL_8657978, EPI_ISL_8657987, EPI_ISL_8657993, EPI_ISL_8657999, EPI_ISL_8658021, EPI_ISL_8658024, EPI_ISL_8658028, EPI_ISL_8658033, EPI_ISL_8658057, EPI_ISL_8658090, EPI_ISL_8658093, EPI_ISL_8658102, EPI_ISL_8658108, EPI_ISL_8658125, EPI_ISL_8658144, EPI_ISL_8658148, EPI_ISL_8658154, EPI_ISL_8658159, EPI_ISL_8658167, EPI_ISL_8658173, EPI_ISL_8658200, EPI_ISL_8658205, EPI_ISL_8658227, EPI_ISL_8658267, EPI_ISL_8658272, EPI_ISL_8658283, EPI_ISL_8658299, EPI_ISL_8658313, EPI_ISL_8658316, EPI_ISL_8658323, EPI_ISL_8658326, EPI_ISL_8658354, EPI_ISL_8658363, EPI_ISL_8658644, EPI_ISL_8658647, EPI_ISL_8658649, EPI_ISL_8658652, EPI_ISL_8658660, EPI_ISL_8658666, EPI_ISL_8658668, EPI_ISL_8658671, EPI_ISL_8658681, EPI_ISL_8658696, EPI_ISL_8658707, EPI_ISL_8658719, EPI_ISL_8658737, EPI_ISL_8658755, EPI_ISL_8658766, EPI_ISL_8658769, EPI_ISL_8658816, EPI_ISL_8658830, EPI_ISL_8658833, EPI_ISL_8658850, EPI_ISL_8658853, EPI_ISL_8658866, EPI_ISL_8658880, EPI_ISL_8658889, EPI_ISL_8658895, EPI_ISL_8658897, EPI_ISL_8658906, EPI_ISL_8658911, EPI_ISL_8658917, EPI_ISL_8658926, EPI_ISL_8658941, EPI_ISL_8658947, EPI_ISL_8658961, EPI_ISL_8658974, EPI_ISL_8658980, EPI_ISL_8658983, EPI_ISL_8658991, EPI_ISL_8659004, EPI_ISL_8659009, EPI_ISL_8659014, EPI_ISL_8659041, EPI_ISL_8659063, EPI_ISL_8659081, EPI_ISL_8659085, EPI_ISL_8659088, EPI_ISL_8659093, EPI_ISL_8659096, EPI_ISL_8659102, EPI_ISL_8659107, EPI_ISL_8659126			
see above	Department of Bacteria, Parasites and Fungi, Statens Serum Institut, Copenhagen, Denmark	Statens Serum Institut Bioinformatics and Microbial Genomics	Danish Covid-19 Genome Consortium
EPI_ISL_8648923	Dept. of Microbiology and Infection Control, Akershus University Hospital HF	Dept. of Microbiology and Infection Control, Akershus University Hospital HF	Alexander Hesselberg Løvestad; Hanne Berggreen; Hege Vangstein Aamot
EPI_ISL_8648853, EPI_ISL_8648854	HOSPITAL DE CABUEÑES	Laboratorio de Virología HUCA	; Alba L; Alvarez-Arguelles ME; Boga JA; Costales I; Coto E; González-Alba JM; Gómez de Oña J; Martín-Rodríguez G; Melón S; Perez-Martínez Z; Rojo S; Sandoval M
EPI_ISL_8648851, EPI_ISL_8648859	HOSPITAL UNIVERSITARIO CENTRAL DE ASTURIAS	Laboratorio de Virología HUCA	; Alba L; Alvarez-Arguelles ME; Boga JA; Costales I; Coto E; González-Alba JM; Gómez de Oña J; Martín-Rodríguez G; Melón S; Perez-Martínez Z; Rojo S; Sandoval M
EPI_ISL_8668943	Klinikum Stuttgart	Robert Koch Institute	
EPI_ISL_8651228	Lighthouse Lab in Alderley Park	Wellcome Sanger Institute for the COVID-19 Genomics UK (COG-UK) Consortium	Cordelia Langford; David K. Jackson; Dominic Kwiatkowski; Ewan Harrison; Ian Johnston; Jacquelyn Wynn; Jeffrey Barrett; John Sillitoe on behalf of the Wellcome Sanger Institute COVID-19 Surveillance Team; Mairead Hyland; Roberto Amato; Sonia Goncalves; The Lighthouse Lab in Alderley Park and Alex Alderton
EPI_ISL_8651229	Lighthouse Lab in Milton Keynes	Wellcome Sanger Institute for the COVID-19 Genomics UK (COG-UK) Consortium	Cordelia Langford; David K. Jackson; Dominic Kwiatkowski; Ewan Harrison; Ian Johnston; Jeffrey Barrett; John Sillitoe on behalf of the Wellcome Sanger Institute COVID-19 Surveillance Team; Roberto Amato; Sonia Goncalves; The Lighthouse Lab in Milton Keynes and Alex Alderton
EPI_ISL_8637669, EPI_ISL_8637811, EPI_ISL_8637838	MapMyGenome	CSIR-Centre for Cellular and Molecular Biology-INSACOG	Amareshwar Vodapalli; Anuradha Acharya; Ara Sreenivas; Archana Bharadwaj Siva; B Himasri; Divya Tej Sowpati; Jandhyala Sai Krishna; Karthik Bharadwaj Tallapaka; Lamuk Zaveri; M. Aravind Kumar; Malini Nemalikanti; Priya Nurkurthy; Rakesh K Mishra; S.P.S. Satish; Sandhya Kiran; Shreekant Verma; Sreelekshmi MS; Sumedha Avadhanula; Surabhi Srivastava; Tulasi Nagabandi; Valli Nagalakshmi Undamatla; Vidhyadhari Methuku
EPI_ISL_8650134	Normah Medical Specialist Centre, Kuching	Institute of Health and Community Medicine	Chan Chia Jui; Chua Hock Hin; David Perera; Ikwannuddin Y; Ooi Mong How; Tonnii Sia Loong Loong; Wong Jyn Shan
EPI_ISL_8650409, EPI_ISL_8650410, EPI_ISL_8650411, EPI_ISL_8650412, EPI_ISL_8650414	SARS-CoV-2 Sequencing Castilla y Leon-Spain Consortium	SARS-CoV-2 Sequencing Castilla y Leon-Spain Consortium	Antonio Orduña-Domingo; Carlos Fuster Foz; Carmen Aldea-Mansilla; Carmen Gimeno Crespo; David Abad; Gabriel March Rosello; Gregoria Megías Lobón; Jose María Eiros Bouza; M. Isabel Fernandez-Natal; Marta Dominguez-Gil; Marta Hernandez; María Antonia García Castro; Mª Fe Brezmes-Valdivieso; Noelia Arenal Andrés; Silvia Rojo; Sonsoles Garcinuño Pérez
EPI_ISL_8650133, EPI_ISL_8650135, EPI_ISL_8650136, EPI_ISL_8650137	Sarawak General Hospital (Kuching)	Institute of Health and Community Medicine	Chan Chia Jui; Chua Hock Hin; David Perera; Ooi Mong How; Tonnii Sia Loong Loong; Wong Jyn Shan

We gratefully acknowledge the following Authors from the Originating laboratories responsible for obtaining the specimens, as well as the Submitting laboratories where the genome data were generated and shared via GISAID, on which this research is based.

All Submitters of data may be contacted directly via www.gisaid.org

Authors are sorted alphabetically.

Acknowledgement EPI_SET Identifier: EPI_SET_20220527nm

Accession ID	Originating Laboratory	Submitting Laboratory	Authors
EPI_ISL_8685691, EPI_ISL_8685692, EPI_ISL_8685693, EPI_ISL_8685697, EPI_ISL_8686761, EPI_ISL_8686819	Aegis Sciences Corporation	Centers for Disease Control and Prevention Division of Viral Diseases, Pathogen Discovery	Alec Vest; Benjamin Rambo-Martin; Christopher Gulvick; Clinton Paden; Cyndi Clark; Dakota Howard; Dhwani Batra; Dillon Nall; Duncan MacCannell; Erisa Sula; Ethan Sanders; Holly Houdeshell; Jason Caravas; Kristine Lacek; Matthew Hardison; Matthew Schmerer; Ola Kvalvaag; Patrick Campbell; Peter Cook; Rob Case; Scott Sammons; Shatavia Morrison; Shaun Westlund; Tymeckia Kendall; Victoria Caban Figueroa; Vikramsinha Ghorpade; Yvette Unoarumhi
EPI_ISL_8685512, EPI_ISL_8685513	LADR Zentrallabor DR. Kramer & Kollegen Geesthacht	Robert Koch Institute	
EPI_ISL_8687362, EPI_ISL_8687363, EPI_ISL_8687364, EPI_ISL_8687365, EPI_ISL_8687366, EPI_ISL_8687367, EPI_ISL_8687370, EPI_ISL_8687372, EPI_ISL_8687373, EPI_ISL_8687374, EPI_ISL_8687375, EPI_ISL_8687376, EPI_ISL_8687378, EPI_ISL_8687379, EPI_ISL_8687380, EPI_ISL_8687381, EPI_ISL_8687383, EPI_ISL_8687384, EPI_ISL_8687385, EPI_ISL_8687386, EPI_ISL_8687387, EPI_ISL_8687388, EPI_ISL_8687389, EPI_ISL_8687390, EPI_ISL_8687392, EPI_ISL_8687393, EPI_ISL_8687394, EPI_ISL_8687395, EPI_ISL_8687396, EPI_ISL_8687397, EPI_ISL_8687398, EPI_ISL_8687400, EPI_ISL_8687403, EPI_ISL_8687404, EPI_ISL_8687408, EPI_ISL_8687409, EPI_ISL_8687410, EPI_ISL_8687412, EPI_ISL_8687416, EPI_ISL_8687418, EPI_ISL_8687419, EPI_ISL_8687420			
see above	Ochsner Health	BioInfoExperts	Amy Feehan; Ben Lain; Chris Huston; David J. Nolan; Judy Crabtree; Julia-Garcia-Diaz; Lucio Miele; Rebecca Rose; Samual Moot; Susanna L. Lamers; Tessa LaFleur

We gratefully acknowledge the following Authors from the Originating laboratories responsible for obtaining the specimens, as well as the Submitting laboratories where the genome data were generated and shared via GISAID, on which this research is based.

All Submitters of data may be contacted directly via www.gisaid.org

Authors are sorted alphabetically.

Accession ID	Originating Laboratory	Submitting Laboratory	Authors
EPI_ISL_8684305, EPI_ISL_8684311	Invenimus AG	Institute of Medical Virology, University of Zurich	Alexandra Trkola; Annette Audigé; Catharine Aquino; Cyril Shah; Daniel Ehrsam; Gabriela Ziltener; Guido Bloemberg; Hubert Rehrauer; Isabel Stürmer; Joel Wirz; Jon Huder; Jürg Böni; Kevin Steiner; Maria Grünberg; Maryam Zaheri; Michael Huber; Riccarda Capaul; Stefan Schmutz; Verena Kufner; Weihong Qi
EPI_ISL_8684213, EPI_ISL_8684225, EPI_ISL_8684231, EPI_ISL_8684238, EPI_ISL_8684240, EPI_ISL_8684243, EPI_ISL_8684244, EPI_ISL_8684245			
see above	Kantonsspital Aarau	Institute of Medical Virology, University of Zurich	Alexandra Trkola; Annette Audigé; Catharine Aquino; Cyril Shah; Daniel Ehrsam; Gabriela Ziltener; Guido Bloemberg; Hubert Rehrauer; Isabel Stürmer; Joel Wirz; Jon Huder; Jürg Böni; Kevin Steiner; Maria Grünberg; Maryam Zaheri; Michael Huber; Riccarda Capaul; Stefan Schmutz; Verena Kufner; Weihong Qi
EPI_ISL_8684370, EPI_ISL_8684386, EPI_ISL_8684387, EPI_ISL_8684392, EPI_ISL_8684398	Kantonsspital Winterthur	Institute of Medical Virology, University of Zurich	Alexandra Trkola; Annette Audigé; Catharine Aquino; Cyril Shah; Daniel Ehrsam; Gabriela Ziltener; Guido Bloemberg; Hubert Rehrauer; Isabel Stürmer; Joel Wirz; Jon Huder; Jürg Böni; Kevin Steiner; Maria Grünberg; Maryam Zaheri; Michael Huber; Riccarda Capaul; Stefan Schmutz; Verena Kufner; Weihong Qi
EPI_ISL_8685507, EPI_ISL_8685509	LADR Zentrallabor DR. Kramer & Kollegen Geesthacht	Robert Koch Institute	
EPI_ISL_8684246	Stadtspital Waid	Institute of Medical Virology, University of Zurich	Alexandra Trkola; Annette Audigé; Catharine Aquino; Cyril Shah; Daniel Ehrsam; Gabriela Ziltener; Guido Bloemberg; Hubert Rehrauer; Isabel Stürmer; Joel Wirz; Jon Huder; Jürg Böni; Kevin Steiner; Maria Grünberg; Maryam Zaheri; Michael Huber; Riccarda Capaul; Stefan Schmutz; Verena Kufner; Weihong Qi
EPI_ISL_8684362, EPI_ISL_8684363, EPI_ISL_8685314, EPI_ISL_8685323, EPI_ISL_8685330	Universität Zürich	Institute of Medical Virology, University of Zurich	Alexandra Trkola; Annette Audigé; Catharine Aquino; Cyril Shah; Daniel Ehrsam; Gabriela Ziltener; Guido Bloemberg; Hubert Rehrauer; Isabel Stürmer; Joel Wirz; Jon Huder; Jürg Böni; Kevin Steiner; Maria Grünberg; Maryam Zaheri; Michael Huber; Riccarda Capaul; Stefan Schmutz; Verena Kufner; Weihong Qi
EPI_ISL_8684260, EPI_ISL_8684328, EPI_ISL_8684340, EPI_ISL_8684346, EPI_ISL_8684364, EPI_ISL_8684433, EPI_ISL_8685227, EPI_ISL_8685245			
see above	Universitäts-Kinderspital Zürich	Institute of Medical Virology, University of Zurich	Alexandra Trkola; Annette Audigé; Catharine Aquino; Cyril Shah; Daniel Ehrsam; Gabriela Ziltener; Guido Bloemberg; Hubert Rehrauer; Isabel Stürmer; Joel Wirz; Jon Huder; Jürg Böni; Kevin Steiner; Maria Grünberg; Maryam Zaheri; Michael Huber; Riccarda Capaul; Stefan Schmutz; Verena Kufner; Weihong Qi
EPI_ISL_8684264, EPI_ISL_8684270, EPI_ISL_8684275, EPI_ISL_8684278, EPI_ISL_8684281, EPI_ISL_8684287, EPI_ISL_8684293, EPI_ISL_8684421, EPI_ISL_8684425, EPI_ISL_8684428, EPI_ISL_8685251, EPI_ISL_8685257, EPI_ISL_8685263, EPI_ISL_8685375, EPI_ISL_8685376, EPI_ISL_8685377, EPI_ISL_8685378, EPI_ISL_8685379			
see above	UniversitätsSpital Zürich	Institute of Medical Virology, University of Zurich	Alexandra Trkola; Annette Audigé; Catharine Aquino; Cyril Shah; Daniel Ehrsam; Gabriela Ziltener; Guido Bloemberg; Hubert Rehrauer; Isabel Stürmer; Joel Wirz; Jon Huder; Jürg Böni; Kevin Steiner; Maria Grünberg; Maryam Zaheri; Michael Huber; Riccarda Capaul; Stefan Schmutz; Verena Kufner; Weihong Qi
EPI_ISL_8684429	Zentrallabor Zürich	Institute of Medical Virology, University of Zurich	Alexandra Trkola; Annette Audigé; Catharine Aquino; Cyril Shah; Daniel Ehrsam; Gabriela Ziltener; Guido Bloemberg; Hubert Rehrauer; Isabel Stürmer; Joel Wirz; Jon Huder; Jürg Böni; Kevin Steiner; Maria Grünberg; Maryam Zaheri; Michael Huber; Riccarda Capaul; Stefan Schmutz; Verena Kufner; Weihong Qi

We gratefully acknowledge the following Authors from the Originating laboratories responsible for obtaining the specimens, as well as the Submitting laboratories where the genome data were generated and shared via GISAID, on which this research is based.

All Submitters of data may be contacted directly via www.gisaid.org

Authors are sorted alphabetically.

Accession ID	Originating Laboratory	Submitting Laboratory	Authors
EPI_ISL_8684196	Invenimus AG	Institute of Medical Virology, University of Zurich	Alexandra Trkola; Annette Audigé; Catharine Aquino; Cyril Shah; Daniel Ehrsam; Gabriela Ziltener; Guido Bloemberg; Hubert Rehrauer; Isabel Stürmer; Joel Wirz; Jon Huder; Jürg Böni; Kevin Steiner; Maria Grünberg; Maryam Zaheri; Michael Huber; Riccarda Capaul; Stefan Schmutz; Verena Kufner; Weihong Qi
EPI_ISL_8682545, EPI_ISL_8682555	Kantonsspital Baden AG	Institute of Medical Virology, University of Zurich	Alexandra Trkola; Annette Audigé; Catharine Aquino; Cyril Shah; Daniel Ehrsam; Gabriela Ziltener; Guido Bloemberg; Hubert Rehrauer; Isabel Stürmer; Joel Wirz; Jon Huder; Jürg Böni; Kevin Steiner; Maria Grünberg; Maryam Zaheri; Michael Huber; Riccarda Capaul; Stefan Schmutz; Verena Kufner; Weihong Qi
EPI_ISL_8682198, EPI_ISL_8682443, EPI_ISL_8682473, EPI_ISL_8682477, EPI_ISL_8682482, EPI_ISL_8682483, EPI_ISL_8682487, EPI_ISL_8682495, EPI_ISL_8682519, EPI_ISL_8682524			
see above	Kantonsspital Winterthur	Institute of Medical Virology, University of Zurich	Alexandra Trkola; Annette Audigé; Catharine Aquino; Cyril Shah; Daniel Ehrsam; Gabriela Ziltener; Guido Bloemberg; Hubert Rehrauer; Isabel Stürmer; Joel Wirz; Jon Huder; Jürg Böni; Kevin Steiner; Maria Grünberg; Maryam Zaheri; Michael Huber; Riccarda Capaul; Stefan Schmutz; Verena Kufner; Weihong Qi
EPI_ISL_8682669, EPI_ISL_8683655	Stadtspital Waid	Institute of Medical Virology, University of Zurich	Alexandra Trkola; Annette Audigé; Catharine Aquino; Cyril Shah; Daniel Ehrsam; Gabriela Ziltener; Guido Bloemberg; Hubert Rehrauer; Isabel Stürmer; Joel Wirz; Jon Huder; Jürg Böni; Kevin Steiner; Maria Grünberg; Maryam Zaheri; Michael Huber; Riccarda Capaul; Stefan Schmutz; Verena Kufner; Weihong Qi
EPI_ISL_8682563, EPI_ISL_8682573, EPI_ISL_8683696, EPI_ISL_8683701	Universität Zürich	Institute of Medical Virology, University of Zurich	Alexandra Trkola; Annette Audigé; Catharine Aquino; Cyril Shah; Daniel Ehrsam; Gabriela Ziltener; Guido Bloemberg; Hubert Rehrauer; Isabel Stürmer; Joel Wirz; Jon Huder; Jürg Böni; Kevin Steiner; Maria Grünberg; Maryam Zaheri; Michael Huber; Riccarda Capaul; Stefan Schmutz; Verena Kufner; Weihong Qi
EPI_ISL_8682327, EPI_ISL_8682373, EPI_ISL_8682379, EPI_ISL_8682387, EPI_ISL_8682396, EPI_ISL_8682403, EPI_ISL_8682420, EPI_ISL_8682429, EPI_ISL_8682534, EPI_ISL_8682612, EPI_ISL_8682619, EPI_ISL_8682643, EPI_ISL_8682667, EPI_ISL_8682697, EPI_ISL_8684122, EPI_ISL_8684133			
see above	Universitäts-Kinderspital Zürich	Institute of Medical Virology, University of Zurich	Alexandra Trkola; Annette Audigé; Catharine Aquino; Cyril Shah; Daniel Ehrsam; Gabriela Ziltener; Guido Bloemberg; Hubert Rehrauer; Isabel Stürmer; Joel Wirz; Jon Huder; Jürg Böni; Kevin Steiner; Maria Grünberg; Maryam Zaheri; Michael Huber; Riccarda Capaul; Stefan Schmutz; Verena Kufner; Weihong Qi
EPI_ISL_8682216, EPI_ISL_8682262, EPI_ISL_8682276, EPI_ISL_8682285, EPI_ISL_8682294, EPI_ISL_8682303, EPI_ISL_8682311, EPI_ISL_8682319, EPI_ISL_8682324, EPI_ISL_8682723, EPI_ISL_8682729, EPI_ISL_8683712, EPI_ISL_8684155			
see above	UniversitätsSpital Zürich	Institute of Medical Virology, University of Zurich	Alexandra Trkola; Annette Audigé; Catharine Aquino; Cyril Shah; Daniel Ehrsam; Gabriela Ziltener; Guido Bloemberg; Hubert Rehrauer; Isabel Stürmer; Joel Wirz; Jon Huder; Jürg Böni; Kevin Steiner; Maria Grünberg; Maryam Zaheri; Michael Huber; Riccarda Capaul; Stefan Schmutz; Verena Kufner; Weihong Qi
EPI_ISL_8683806	Valais Hospital, Central Institute	Valais Hospital, Central Institute	Alexis Dumoulin; Cedric Howald; Deborah Penet; Henri Pegeot; Ioannis Xenarios; Keith Harshman; Lorenzo Cerutti; Melyssa Elies
EPI_ISL_8684184	Zentrallabor Zürich	Institute of Medical Virology, University of Zurich	Alexandra Trkola; Annette Audigé; Catharine Aquino; Cyril Shah; Daniel Ehrsam; Gabriela Ziltener; Guido Bloemberg; Hubert Rehrauer; Isabel Stürmer; Joel Wirz; Jon Huder; Jürg Böni; Kevin Steiner; Maria Grünberg; Maryam Zaheri; Michael Huber; Riccarda Capaul; Stefan Schmutz; Verena Kufner; Weihong Qi

We gratefully acknowledge the following Authors from the Originating laboratories responsible for obtaining the specimens, as well as the Submitting laboratories where the genome data were generated and shared via GISAID, on which this research is based.

All Submitters of data may be contacted directly via www.gisaid.org

Authors are sorted alphabetically.

Acknowledgement EPI_SET Identifier: EPI_SET_20220527uq

Accession ID	Originating Laboratory	Submitting Laboratory	Authors
EPI_ISL_8672912	Infinity Biologix	Centers for Disease Control and Prevention Division of Viral Diseases, Pathogen Discovery	Benjamin Rambo-Martin; Chirayu Goswami; Christian Bixby; Christopher Gulvick; Clinton Paden; Dakota Howard; Dhwani Batra; Duncan MacCannell; Erisa Sula; Jason Caravas; Jonathan Schultz; Kristine Lacek; Matthew Schmerer; Peter Cook; Robin Grimwood; Russ Hager; Scott Sammons; Shatavia Morrison; Tymeckia Kendall; Victoria Caban Figueroa; Yihe Wang; Yvette Unoarumhi
EPI_ISL_8668943, EPI_ISL_8668945, EPI_ISL_8668946, EPI_ISL_8668947, EPI_ISL_8668948, EPI_ISL_8668950, EPI_ISL_8668951, EPI_ISL_8668953, EPI_ISL_8668954, EPI_ISL_8668955, EPI_ISL_8668958, EPI_ISL_8668959, EPI_ISL_8668960, EPI_ISL_8668961, EPI_ISL_8668963, EPI_ISL_8668964, EPI_ISL_8668965, EPI_ISL_8668966, EPI_ISL_8668967, EPI_ISL_8668968, EPI_ISL_8668969, EPI_ISL_8668970			
see above	Klinikum Stuttgart	Robert Koch Institute	
EPI_ISL_8670505, EPI_ISL_8671355, EPI_ISL_8672018, EPI_ISL_8672560	LABOPAT	LABOPAT	Cynthia Penaloza; Luis Mendoza; Silvia Montilla
EPI_ISL_8680564	Laborarztpraxis Dres. med. Walther Weindel & Kollegen	Robert Koch Institute	
EPI_ISL_8674176, EPI_ISL_8674177, EPI_ISL_8674178	MVZ Labor Krone GbR	Robert Koch Institute	
EPI_ISL_8679094	Sonic - Bioscientia - MVZ Labor Saar GmbH	Robert Koch Institute	
EPI_ISL_8681459, EPI_ISL_8681559	Stadtspital Waid	Institute of Medical Virology, University of Zurich	Alexandra Trkola; Annette Audigé; Catharine Aquino; Cyril Shah; Daniel Ehrsam; Gabriela Ziltener; Guido Bloemberg; Hubert Rehrauer; Isabel Stürmer; Joel Wirz; Jon Huder; Jürg Böni; Kevin Steiner; Maria Grünberg; Maryam Zaheri; Michael Huber; Riccarda Capaul; Stefan Schmutz; Verena Kufner; Weihong Qi
EPI_ISL_8681454, EPI_ISL_8681466, EPI_ISL_8681480, EPI_ISL_8681505, EPI_ISL_8681553	Universität Zürich	Institute of Medical Virology, University of Zurich	Alexandra Trkola; Annette Audigé; Catharine Aquino; Cyril Shah; Daniel Ehrsam; Gabriela Ziltener; Guido Bloemberg; Hubert Rehrauer; Isabel Stürmer; Joel Wirz; Jon Huder; Jürg Böni; Kevin Steiner; Maria Grünberg; Maryam Zaheri; Michael Huber; Riccarda Capaul; Stefan Schmutz; Verena Kufner; Weihong Qi
EPI_ISL_8681448, EPI_ISL_8681566, EPI_ISL_8681575, EPI_ISL_8681580	Universitäts-Kinderspital Zürich	Institute of Medical Virology, University of Zurich	Alexandra Trkola; Annette Audigé; Catharine Aquino; Cyril Shah; Daniel Ehrsam; Gabriela Ziltener; Guido Bloemberg; Hubert Rehrauer; Isabel Stürmer; Joel Wirz; Jon Huder; Jürg Böni; Kevin Steiner; Maria Grünberg; Maryam Zaheri; Michael Huber; Riccarda Capaul; Stefan Schmutz; Verena Kufner; Weihong Qi
EPI_ISL_8681497, EPI_ISL_8681519, EPI_ISL_8681526, EPI_ISL_8681538, EPI_ISL_8681621, EPI_ISL_8682183, EPI_ISL_8682189			
see above	UniversitätsSpital Zürich	Institute of Medical Virology, University of Zurich	Alexandra Trkola; Annette Audigé; Catharine Aquino; Cyril Shah; Daniel Ehrsam; Gabriela Ziltener; Guido Bloemberg; Hubert Rehrauer; Isabel Stürmer; Joel Wirz; Jon Huder; Jürg Böni; Kevin Steiner; Maria Grünberg; Maryam Zaheri; Michael Huber; Riccarda Capaul; Stefan Schmutz; Verena Kufner; Weihong Qi

We gratefully acknowledge the following Authors from the Originating laboratories responsible for obtaining the specimens, as well as the Submitting laboratories where the genome data were generated and shared via GISAID, on which this research is based.

All Submitters of data may be contacted directly via www.gisaid.org

Authors are sorted alphabetically.

Accession ID	Originating Laboratory	Submitting Laboratory	Authors
EPI_ISL_8660509	Clinique Saint-Pierre Ottignies	UCLouvain/IREC/MBLG-CTMA	Benoit Kabamba Mukadi; Bertrand Bearzatto; Jean-Luc Gala; Nicolas Pinte; Paul Blanpain; Simon Ophélie; Valentin Coste
EPI_ISL_8660507, EPI_ISL_8660508	Cliniques universitaires Saint-Luc	UCLouvain/IREC/MBLG-CTMA	Benoit Kabamba Mukadi; Bertrand Bearzatto; Jean-Luc Gala; Nicolas Pinte; Paul Blanpain; Simon Ophélie; Valentin Coste
EPI_ISL_8659865, EPI_ISL_8659893, EPI_ISL_8659930, EPI_ISL_8659936, EPI_ISL_8659955, EPI_ISL_8660005, EPI_ISL_8660010, EPI_ISL_8660023, EPI_ISL_8660055, EPI_ISL_8660084, EPI_ISL_8660102, EPI_ISL_8660142, EPI_ISL_8660145, EPI_ISL_8660162, EPI_ISL_8660171, EPI_ISL_8660190, EPI_ISL_8660193, EPI_ISL_8660199, EPI_ISL_8660203, EPI_ISL_8660205, EPI_ISL_8660206, EPI_ISL_8660210, EPI_ISL_8660213, EPI_ISL_8660226, EPI_ISL_8660231, EPI_ISL_8660247, EPI_ISL_8660253, EPI_ISL_8660279, EPI_ISL_8660286			
see above	Department of Bacteria, Parasites and Fungi, Statens Serum Institut, Copenhagen, Denmark	Statens Serum Institut Bioinformatics and Microbial Genomics	Danish Covid-19 Genome Consortium
EPI_ISL_8668942	Klinikum Stuttgart	Robert Koch Institute	
EPI_ISL_8665271, EPI_ISL_8665273, EPI_ISL_8665274, EPI_ISL_8665275, EPI_ISL_8665277, EPI_ISL_8665278, EPI_ISL_8665280, EPI_ISL_8665281			
see above	Laboratorio de Pesquisa em Virologia, FAMERP, SJRP	Laboratorio de Pesquisa em Virologia, FAMERP, SJRP	Beatriz de Carvalho Marques; Cecília Artico Banho; Cíntia Bittar; Fábio Sossai Possebon; Guilherme Campos; Helena Lage Ferreira; Jorge A. Petroli Marchesi; João Pessoa Araújo Jr.; Leila Sabrina Ullmann; Lívia Sacchetto; Maisa C. Pereira Parra; Marília Moraes; Maurício L. Nogueira.; Paula Rahal; Paulo Inacio da Costa
EPI_ISL_8665230, EPI_ISL_8665242, EPI_ISL_8665300, EPI_ISL_8665319, EPI_ISL_8665344, EPI_ISL_8665617, EPI_ISL_8665682			
see above	Medizinisch-Diagnostisches Labor Kempten allgäulab	Robert Koch Institute	
EPI_ISL_8668093	SYNLAB Jena Oncoscreen	Robert Koch Institute	
EPI_ISL_8662043	SYNLAB MVZ Hamburg	Robert Koch Institute	

We gratefully acknowledge the following Authors from the Originating laboratories responsible for obtaining the specimens, as well as the Submitting laboratories where the genome data were generated and shared via GISAID, on which this research is based.

All Submitters of data may be contacted directly via www.gisaid.org

Authors are sorted alphabetically.

Accession ID	Originating Laboratory	Submitting Laboratory	Authors
EPI_ISL_8659132, EPI_ISL_8659142, EPI_ISL_8659151, EPI_ISL_8659154, EPI_ISL_8659163, EPI_ISL_8659166, EPI_ISL_8659195, EPI_ISL_8659203, EPI_ISL_8659221, EPI_ISL_8659223, EPI_ISL_8659239, EPI_ISL_8659247, EPI_ISL_8659253, EPI_ISL_8659285, EPI_ISL_8659363, EPI_ISL_8659382, EPI_ISL_8659385, EPI_ISL_8659393, EPI_ISL_8659399, EPI_ISL_8659422, EPI_ISL_8659425, EPI_ISL_8659430, EPI_ISL_8659432, EPI_ISL_8659442, EPI_ISL_8659446, EPI_ISL_8659457, EPI_ISL_8659489, EPI_ISL_8659491, EPI_ISL_8659497, EPI_ISL_8659558, EPI_ISL_8659563, EPI_ISL_8659565, EPI_ISL_8659576, EPI_ISL_8659583, EPI_ISL_8659632, EPI_ISL_8659688, EPI_ISL_8659689, EPI_ISL_8659693, EPI_ISL_8659710, EPI_ISL_8659722, EPI_ISL_8659734, EPI_ISL_8659737, EPI_ISL_8659762, EPI_ISL_8659778, EPI_ISL_8659793, EPI_ISL_8659796, EPI_ISL_8659802, EPI_ISL_8659813, EPI_ISL_8659822, EPI_ISL_8659841			
see above	Department of Bacteria, Parasites and Fungi, Statens Serum Institut, Copenhagen, Denmark	Statens Serum Institut Bioinformatics and Microbial Genomics	Danish Covid-19 Genome Consortium

We gratefully acknowledge the following Authors from the Originating laboratories responsible for obtaining the specimens, as well as the Submitting laboratories where the genome data were generated and shared via GISAID, on which this research is based.

All Submitters of data may be contacted directly via www.gisaid.org

Authors are sorted alphabetically.

Acknowledgement EPI_SET Identifier: EPI_SET_20220527tx

Accession ID	Originating Laboratory	Submitting Laboratory	Authors
EPI_ISL_8694511	Center for Laboratory Medicine	Center for Laboratory Medicine, ZLMSG	Yannick Gerth
EPI_ISL_8700219, EPI_ISL_8700241, EPI_ISL_8700285, EPI_ISL_8700291, EPI_ISL_8700294, EPI_ISL_8700297, EPI_ISL_8700300, EPI_ISL_8700302, EPI_ISL_8700303, EPI_ISL_8700320, EPI_ISL_8700326, EPI_ISL_8700329, EPI_ISL_8700344, EPI_ISL_8700348, EPI_ISL_8700355, EPI_ISL_8700362, EPI_ISL_8700380, EPI_ISL_8700384, EPI_ISL_8700395, EPI_ISL_8700413, EPI_ISL_8700417, EPI_ISL_8700420, EPI_ISL_8700441, EPI_ISL_8700450, EPI_ISL_8700454, EPI_ISL_8700456, EPI_ISL_8700457, EPI_ISL_8700462, EPI_ISL_8700466, EPI_ISL_8700468, EPI_ISL_8700482, EPI_ISL_8700507, EPI_ISL_8700569, EPI_ISL_8700573, EPI_ISL_8700580, EPI_ISL_8701342, EPI_ISL_8701370, EPI_ISL_8701408, EPI_ISL_8701421, EPI_ISL_8701435, EPI_ISL_8701468, EPI_ISL_8701478, EPI_ISL_8701488, EPI_ISL_8701509, EPI_ISL_8701532, EPI_ISL_8701535, EPI_ISL_8701552, EPI_ISL_8701555, EPI_ISL_8701571, EPI_ISL_8701591, EPI_ISL_8701593, EPI_ISL_8701596, EPI_ISL_8701601, EPI_ISL_8701606, EPI_ISL_8701709, EPI_ISL_8701731, EPI_ISL_8701748, EPI_ISL_8701755, EPI_ISL_8701776, EPI_ISL_8701787, EPI_ISL_8701793, EPI_ISL_8701796, EPI_ISL_8701801, EPI_ISL_8701807, EPI_ISL_8701818, EPI_ISL_8701845, EPI_ISL_8701849, EPI_ISL_8701856, EPI_ISL_8701868, EPI_ISL_8701870, EPI_ISL_8701898, EPI_ISL_8701902, EPI_ISL_8701912, EPI_ISL_8701918, EPI_ISL_8701921, EPI_ISL_8701941, EPI_ISL_8701971, EPI_ISL_8701979, EPI_ISL_8701988, EPI_ISL_8702019, EPI_ISL_8702027, EPI_ISL_8702044, EPI_ISL_8702059, EPI_ISL_8702084, EPI_ISL_8702107, EPI_ISL_8702119, EPI_ISL_8702122, EPI_ISL_8702157, EPI_ISL_8702164, EPI_ISL_8702197, EPI_ISL_8702200, EPI_ISL_8702219, EPI_ISL_8702222, EPI_ISL_8702231, EPI_ISL_8702241, EPI_ISL_8702251, EPI_ISL_8702257, EPI_ISL_8702260, EPI_ISL_8702267, EPI_ISL_8702303, EPI_ISL_8702327, EPI_ISL_8702335, EPI_ISL_8702363, EPI_ISL_8702372, EPI_ISL_8702375, EPI_ISL_8702383, EPI_ISL_8702385, EPI_ISL_8702389, EPI_ISL_8702394, EPI_ISL_8702403, EPI_ISL_8702425, EPI_ISL_8702428, EPI_ISL_8702430, EPI_ISL_8702432, EPI_ISL_8702445, EPI_ISL_8702447, EPI_ISL_8702464, EPI_ISL_8702470, EPI_ISL_8702473, EPI_ISL_8702495, EPI_ISL_8702504, EPI_ISL_8702519, EPI_ISL_8702527, EPI_ISL_8702528, EPI_ISL_8702549, EPI_ISL_8702559, EPI_ISL_8702573, EPI_ISL_8702579, EPI_ISL_8702582, EPI_ISL_8702593, EPI_ISL_8702596, EPI_ISL_8702605, EPI_ISL_8702608, EPI_ISL_8702617, EPI_ISL_8702620, EPI_ISL_8702631, EPI_ISL_8702642, EPI_ISL_8702651, EPI_ISL_8702671, EPI_ISL_8702691, EPI_ISL_8702697, EPI_ISL_8702703, EPI_ISL_8702710, EPI_ISL_8702720, EPI_ISL_8702722, EPI_ISL_8702735, EPI_ISL_8702737, EPI_ISL_8702747, EPI_ISL_8702767, EPI_ISL_8702854, EPI_ISL_8702857, EPI_ISL_8702859, EPI_ISL_8702870, EPI_ISL_8702873, EPI_ISL_8702886, EPI_ISL_8702908, EPI_ISL_8702963, EPI_ISL_8702996, EPI_ISL_8703001, EPI_ISL_8703012, EPI_ISL_8703021, EPI_ISL_8703048, EPI_ISL_8703059, EPI_ISL_8703140, EPI_ISL_8703144, EPI_ISL_8703161, EPI_ISL_8703164, EPI_ISL_8703168, EPI_ISL_8703172, EPI_ISL_8703207, EPI_ISL_8703231, EPI_ISL_8703241, EPI_ISL_8703257, EPI_ISL_8703263, EPI_ISL_8703269, EPI_ISL_8703276, EPI_ISL_8703294, EPI_ISL_8703310, EPI_ISL_8703314, EPI_ISL_8703329, EPI_ISL_8703340, EPI_ISL_8703354, EPI_ISL_8703367, EPI_ISL_8703376, EPI_ISL_8703380, EPI_ISL_8703381, EPI_ISL_8703386, EPI_ISL_8703390, EPI_ISL_8703394, EPI_ISL_8703437, EPI_ISL_8703444, EPI_ISL_8703449, EPI_ISL_8703465, EPI_ISL_8703473, EPI_ISL_8703495, EPI_ISL_8703522, EPI_ISL_8703524, EPI_ISL_8703535, EPI_ISL_8703542, EPI_ISL_8703561, EPI_ISL_8703596, EPI_ISL_8703612, EPI_ISL_8703624, EPI_ISL_8703645, EPI_ISL_8703656, EPI_ISL_8703672, EPI_ISL_8703801, EPI_ISL_8703827, EPI_ISL_8704004, EPI_ISL_8704007, EPI_ISL_8704014, EPI_ISL_8704018, EPI_ISL_8704046, EPI_ISL_8704072, EPI_ISL_8704122, EPI_ISL_8704126, EPI_ISL_8704142, EPI_ISL_8704146, EPI_ISL_8704148, EPI_ISL_8704155, EPI_ISL_8704159, EPI_ISL_8704175, EPI_ISL_8704190, EPI_ISL_8704227, EPI_ISL_8704243, EPI_ISL_8704245, EPI_ISL_8704273, EPI_ISL_8704277, EPI_ISL_8704291, EPI_ISL_8704295, EPI_ISL_8704333, EPI_ISL_8704356, EPI_ISL_8704368, EPI_ISL_8704416, EPI_ISL_8704425, EPI_ISL_8704429, EPI_ISL_8704437, EPI_ISL_8704449, EPI_ISL_8704474, EPI_ISL_8704487, EPI_ISL_8704497, EPI_ISL_8704500, EPI_ISL_8704505, EPI_ISL_8704519, EPI_ISL_8704526, EPI_ISL_8704550, EPI_ISL_8704553, EPI_ISL_8704568, EPI_ISL_8704606, EPI_ISL_8704625, EPI_ISL_8704633, EPI_ISL_8704639, EPI_ISL_8704642, EPI_ISL_8704656, EPI_ISL_8704664, EPI_ISL_8704668, EPI_ISL_8704673, EPI_ISL_8704676, EPI_ISL_8704708, EPI_ISL_8704730, EPI_ISL_8704775, EPI_ISL_8704788, EPI_ISL_8704792, EPI_ISL_8704804, EPI_ISL_8704807, EPI_ISL_8704821, EPI_ISL_8704825, EPI_ISL_8704845, EPI_ISL_8704883, EPI_ISL_8704903, EPI_ISL_8704913, EPI_ISL_8704917, EPI_ISL_8704924, EPI_ISL_8704938, EPI_ISL_8704959, EPI_ISL_8705001, EPI_ISL_8705023, EPI_ISL_8705046, EPI_ISL_8705051, EPI_ISL_8705055, EPI_ISL_8705058, EPI_ISL_8705062, EPI_ISL_8705079, EPI_ISL_8705127, EPI_ISL_8705131, EPI_ISL_8705150, EPI_ISL_8705175, EPI_ISL_8705180, EPI_ISL_8705191, EPI_ISL_8705198, EPI_ISL_8705236, EPI_ISL_8705238, EPI_ISL_8705241, EPI_ISL_8705243, EPI_ISL_8705250, EPI_ISL_8705252, EPI_ISL_8705253, EPI_ISL_8705257, EPI_ISL_8705258, EPI_ISL_8705281, EPI_ISL_8705283, EPI_ISL_8705307, EPI_ISL_8705325, EPI_ISL_8705344, EPI_ISL_8705348, EPI_ISL_8705355, EPI_ISL_8705378, EPI_ISL_8705386, EPI_ISL_8705396, EPI_ISL_8705424, EPI_ISL_8705427, EPI_ISL_8705441, EPI_ISL_8705463, EPI_ISL_8705469, EPI_ISL_8705477, EPI_ISL_8705488, EPI_ISL_8705500, EPI_ISL_8705503, EPI_ISL_8705507, EPI_ISL_8705511, EPI_ISL_8705521, EPI_ISL_8705571, EPI_ISL_8705581, EPI_ISL_8705594, EPI_ISL_8705596, EPI_ISL_8705680, EPI_ISL_8705697, EPI_ISL_8705704, EPI_ISL_8705708, EPI_ISL_8705712, EPI_ISL_8705719, EPI_ISL_8705722, EPI_ISL_8705733, EPI_ISL_8705746, EPI_ISL_8705758, EPI_ISL_8705761, EPI_ISL_8705765, EPI_ISL_8705769, EPI_ISL_8705783, EPI_ISL_8705792, EPI_ISL_8705797, EPI_ISL_8705800, EPI_ISL_8705809, EPI_ISL_8705829, EPI_ISL_8705831, EPI_ISL_8705842, EPI_ISL_8705876, EPI_ISL_8705892, EPI_ISL_8705896, EPI_ISL_8705914, EPI_ISL_8705918, EPI_ISL_8705922, EPI_ISL_8705946, EPI_ISL_8705962, EPI_ISL_8705967, EPI_ISL_8705969, EPI_ISL_8705973, EPI_ISL_8705979, EPI_ISL_8706004, EPI_ISL_8706012, EPI_ISL_8706023, EPI_ISL_8706037, EPI_ISL_8706043, EPI_ISL_8706053, EPI_ISL_8706072, EPI_ISL_8706076, EPI_ISL_8706097, EPI_ISL_8706100, EPI_ISL_8706104, EPI_ISL_8706108, EPI_ISL_8706135, EPI_ISL_8706139, EPI_ISL_8706147, EPI_ISL_8706181, EPI_ISL_8706188, EPI_ISL_8706192			
see above	Department of Bacteria, Parasites and Fungi, Statens Serum Institut, Copenhagen, Denmark	Statens Serum Institut Bioinformatics and Microbial Genomics	Danish Covid-19 Genome Consortium
EPI_ISL_8700415	Karolinska University Hospital Huddinge	Karolinska University Hospital	Annika Tiveljung Lindell; Henning Onsbring; Jan Albert; Karina Hentrich; Lynda Eneh; Maria Ropat; Martin Ekman; Natalija Gerasimcik; Robert Dyrdak; Sandra Broddesson; Shambhu Ganeshappa Aralaguppe; Tanja Normark; Tobias Allander; Valtteri Wirta; Zhibing Yun
EPI_ISL_8695191, EPI_ISL_8695335, EPI_ISL_8695913, EPI_ISL_8695958, EPI_ISL_8695967, EPI_ISL_8697860, EPI_ISL_8699738, EPI_ISL_8699837, EPI_ISL_8701499, EPI_ISL_8701569, EPI_ISL_8701595			
see above	Lighthouse Lab in Alderley Park	Wellcome Sanger Institute for the COVID-19 Genomics UK (COG-UK) Consortium	Cordelia Langford; David K. Jackson; Dominic Kwiatkowski; Ewan Harrison; Ian Johnston; Jacquelyn Wynn; Jeffrey Barrett; John Sillitoe on behalf of the Wellcome Sanger Institute COVID-19 Surveillance Team; Mairead Hyland; Roberto Amato; Sonia Goncalves; The Lighthouse Lab in Alderley Park and Alex Alderton
EPI_ISL_8695142, EPI_ISL_8695678, EPI_ISL_8695844, EPI_ISL_8695873, EPI_ISL_8695927, EPI_ISL_8696349, EPI_ISL_8697885, EPI_ISL_8697903, EPI_ISL_8697930, EPI_ISL_8699721, EPI_ISL_8703315, EPI_ISL_8704326, EPI_ISL_8704422, EPI_ISL_8704863, EPI_ISL_8705845, EPI_ISL_8706197			
see above	Lighthouse Lab in Glasgow	Wellcome Sanger Institute for the COVID-19 Genomics UK (COG-UK) Consortium	Anna Dominiczak and Alex Alderton; Carol Clugston; Cordelia Langford; David Gray; David K. Jackson; Dominic Kwiatkowski; Ewan Harrison; Harper VanSteenhouse; Ian Johnston; Jeffrey Barrett; John Sillitoe on behalf of the Wellcome Sanger Institute COVID-19 Surveillance Team; Roberto Amato; Sonia Goncalves; Yumi Kasai
EPI_ISL_8695718, EPI_ISL_8695740, EPI_ISL_8695877, EPI_ISL_8695910, EPI_ISL_8696735, EPI_ISL_8696857, EPI_ISL_8697593, EPI_ISL_8697689, EPI_ISL_8697756, EPI_ISL_8698826, EPI_ISL_8699265, EPI_ISL_8699445, EPI_ISL_8701597, EPI_ISL_8704563			
see above	Lighthouse Lab in Milton Keynes	Wellcome Sanger Institute for the COVID-19 Genomics UK (COG-UK) Consortium	Cordelia Langford; David K. Jackson; Dominic Kwiatkowski; Ewan Harrison; Ian Johnston; Jeffrey Barrett; John Sillitoe on behalf of the Wellcome Sanger Institute COVID-19 Surveillance Team; Roberto Amato; Sonia Goncalves; The Lighthouse Lab in Milton Keynes and Alex Alderton
EPI_ISL_8703863, EPI_ISL_8703864, EPI_ISL_8703865, EPI_ISL_8703866, EPI_ISL_8703867, EPI_ISL_8703868, EPI_ISL_8703869, EPI_ISL_8703870, EPI_ISL_8703871, EPI_ISL_8703872, EPI_ISL_8703873, EPI_ISL_8703874, EPI_ISL_8703875, EPI_ISL_8703876, EPI_ISL_8703877, EPI_ISL_8703878, EPI_ISL_8703879, EPI_ISL_8703880, EPI_ISL_8703881, EPI_ISL_8703882, EPI_ISL_8703883, EPI_ISL_8703884, EPI_ISL_8703885, EPI_ISL_8703886, EPI_ISL_8703887, EPI_ISL_8703888, EPI_ISL_8703889, EPI_ISL_8703890, EPI_ISL_8703891, EPI_ISL_8703892, EPI_ISL_8703893, EPI_ISL_8703895, EPI_ISL_8703896, EPI_ISL_8703897, EPI_ISL_8703898, EPI_ISL_8703899, EPI_ISL_8703900, EPI_ISL_8703901, EPI_ISL_8703902, EPI_ISL_8703903, EPI_ISL_8703904			
see above	National Public Health Laboratory, National Centre for Infectious Diseases	National Public Health Laboratory, National Centre for Infectious Diseases	Benny Yeo; Grace Ngan; Lin Cui; Raymond Tzer Pin Lin; Royce Ang; Samuel Loo; Zhenyang Zhou
EPI_ISL_8696665	Rosalind Franklin Laboratory	Wellcome Sanger Institute for the COVID-19 Genomics UK (COG-UK) Consortium	Cordelia Langford; David K. Jackson; Dominic Kwiatkowski; Donald Fraser; Ewan Harrison; Ian Johnston; Jeffrey Barrett; John Sillitoe on behalf of the Wellcome Sanger Institute COVID-19 Surveillance Team; Rob Howes; Roberto Amato; Sonia Goncalves; Suki Lee; The Rosalind Franklin Laboratory and Alex Alderton
EPI_ISL_8691409	State Laboratories Division, Hawaii State Department of Health	State Laboratories Division, Hawaii State Department of Health	Ayana Garnet; Briana Ofilas; Cheryl-lynn Daquip; Cheyenne Barela; Daniel Strange; Drew Kuwazaki; Edward Desmond; Jeffrey Au; Mark Nagata; Pamela O'Brien; Razvan Sultana; Remedios Gose; Samantha Cotter; Samantha Sruba
EPI_ISL_8699489, EPI_ISL_8699490, EPI_ISL_8699491, EPI_ISL_8699492, EPI_ISL_8699493, EPI_ISL_8699494, EPI_ISL_8699495, EPI_ISL_8699496, EPI_ISL_8699497, EPI_ISL_8699500, EPI_ISL_8699501, EPI_ISL_8699502, EPI_ISL_8699503, EPI_ISL_8699504, EPI_ISL_8699505, EPI_ISL_8699506, EPI_ISL_8699507, EPI_ISL_8699508			
see above	Synlab Eesti OÜ	1. Laboratory of Communicable Diseases (Estonia); 2. Eurofins Genomics Europe Sequencing GmbH	Abroi A.; Avi R.; Dotsenko L.; Epštein J.; Hoidmets D.; Huik K.; Härma M-A.; Jaaniso E.; Kaarna K.; Kallas E.; Koppel I.; Kuzmin I.; Lahesaare A.; Lutsar I.; Metspalu M.; Milani L.; Naaber P.; Niglas H.; Oopkaup O.E.; Pauskar M.; Peterson H.; Päll T.; Ratnik K.; Raudvere U.; Reisberg T.; Sadikova O.; Sepp H.; Shablinskaja A.; Suija H.; Talas U.G.; Truusalu K.

We gratefully acknowledge the following Authors from the Originating laboratories responsible for obtaining the specimens, as well as the Submitting laboratories where the genome data were generated and shared via GISAID, on which this research is based.

All Submitters of data may be contacted directly via www.gisaid.org

Authors are sorted alphabetically.

Accession ID	Originating Laboratory	Submitting Laboratory	Authors
EPI_ISL_8723794	Department of Clinical Microbiology	GIGA Medical Genomics	Bouchra Boujemla; Claire Gourzonès; Cécile Meex; Keith Durkin; Laurent Gillet; Maria Artesi; Marie-Pierre Hayette; Nadine Cambisano; Nathalie Renotte; Olivier Ek; Sébastien Bontems; Vincent Bours
EPI_ISL_8713546, EPI_ISL_8714397, EPI_ISL_8714398, EPI_ISL_8714399	Hrvatski zavod za javno zdravstvo	Hrvatski zavod za javno zdravstvo	Anita Jurić; Dragan Jurić; Irena Tabain; Ivana Ferenčak; Josipa Kuzle
EPI_ISL_8717998, EPI_ISL_8718000, EPI_ISL_8718008, EPI_ISL_8718009, EPI_ISL_8718012, EPI_ISL_8718016, EPI_ISL_8718017, EPI_ISL_8718018, EPI_ISL_8718025, EPI_ISL_8718036, EPI_ISL_8718037, EPI_ISL_8718038, EPI_ISL_8718049, EPI_ISL_8718051			
see above	Idaho Bureau of Laboratories	Idaho Bureau of Laboratories	"R. Beukelman; Aimee Ceniseros; Christian Loera; Christopher Ball"; Matthew Charles Burns; Robert L. Voermans
EPI_ISL_8717063	Jessa	Jessa	Severine Berden et al. on behalf of the Jessa_cmdLab
EPI_ISL_8725069	KKP Kelas 1 Soekarno-Hatta (iLab)	National Institute of Health Research and Development	Arie Ardiansyah Nugraha; Fajar Nur Sulistiyohadi; Hana Apsari Pawestri; Hartanti Dian Ikawati; Kartika Dewi Puspa; Nelly Puspandari; Nur Ika Hariastuti; Putri Widia; Subangkit; Vivi Setiawaty
EPI_ISL_8726190, EPI_ISL_8726191, EPI_ISL_8726192	Kansas Health and Environmental Lab	Kansas Health and Environmental Lab	Amanda Bradley; Carrie Welch; Gary Buruss; Jonathan Barnell; Mike Grose; Victor Anderson; and Phil Adam
EPI_ISL_8713004, EPI_ISL_8713006, EPI_ISL_8713008, EPI_ISL_8713015, EPI_ISL_8713016, EPI_ISL_8713017, EPI_ISL_8713020, EPI_ISL_8713022, EPI_ISL_8713025, EPI_ISL_8713032			
see above	Kasturba Hospital Molecular Lab - INSACOG	Kasturba hospital WGS	Jayanthi Shastri; Vidushi Chitalia
EPI_ISL_8713269, EPI_ISL_8713270, EPI_ISL_8713271	Klinika za infektivne bolesti "Dr. Fran Mihaljević"	Hrvatski zavod za javno zdravstvo	Anita Jurić; Dragan Jurić; Irena Tabain; Ivana Ferenčak; Josipa Kuzle
EPI_ISL_8718383, EPI_ISL_8718435, EPI_ISL_8718448, EPI_ISL_8718556, EPI_ISL_8718557, EPI_ISL_8718558, EPI_ISL_8718559			
see above	Laboratory Corporation of America	Centers for Disease Control and Prevention Division of Viral Diseases, Pathogen Discovery	Amanda Douglas; Amanda Suchanek; Andrea Throop; Ayla Burns; Benjamin Rambo-Martin; Bobbi Croy; Brian Krueger; Brian Norvell; Christopher Gulvick; Christos Petropoulos; Clinton Paden; Craig Lukasik; Dakota Howard; Debbie Boles; Dhwani Batra; Duncan MacCannell; Eyad Almasri; Goran Stevovic; Howard Engler; Hrushikesh Deshmukh; Jake Humphrey; Jana Schroth; Jason Caravas; Joe Voshell; John Pruitt; Jonathan Meltzer; Jonathan Williams; Kimberly Wagner; Kristine Lacek; Lax Iyer; Lisa Pfefferle; Lyndon Tilson; Manoj Jain; Marcia Eisenberg; Mary Cristobal; Mary Williamson; Matthew Robinson; Matthew Schmerer; Michael Levandoski; Mike Sapeta; Mindy Nye; Minoo Agarwal; Mohan Kolli; Nuthawin Charoensri; Oren Cohen; Peter Cook; Prashant Gupta; Qian Zeng; Rama Ghatti; Scott Parker; Scott Ryan; Scott Sammons; Shatavia Morrison; Stanley Letovsky; Steven Ragan; Suresh Selvaraju; Susan Countryman; Susan Hicks; Suzanne Dale; Thomas Urban; Tim Kuphal; Tricia Zwiefelhofer; Tymeckia Kendall; Victoria Caban Figueroa; Vincent Drouillon; Yvette Unoarumhi
EPI_ISL_8713545	Nastavni zavod za javno zdravstvo Primorsko-Goranske županije	Hrvatski zavod za javno zdravstvo	Anita Jurić; Dragan Jurić; Irena Tabain; Ivana Ferenčak; Josipa Kuzle
EPI_ISL_8713549	Opća bolnica Bjelovar	Hrvatski zavod za javno zdravstvo	Anita Jurić; Dragan Jurić; Irena Tabain; Ivana Ferenčak; Josipa Kuzle
EPI_ISL_8713778	Tulare County Public Health Laboratory	Tulare County Public Health Laboratory	Denise L Lopez; Joshua Kootstra
EPI_ISL_8713361, EPI_ISL_8714683	Zavod za javno zdravstvo Dubrovačko-Neretvanske županije	Hrvatski zavod za javno zdravstvo	Anita Jurić; Dragan Jurić; Irena Tabain; Ivana Ferenčak; Josipa Kuzle
EPI_ISL_8713547	Zavod za javno zdravstvo Sisačko-Moslavačke županije	Hrvatski zavod za javno zdravstvo	Anita Jurić; Dragan Jurić; Irena Tabain; Ivana Ferenčak; Josipa Kuzle

We gratefully acknowledge the following Authors from the Originating laboratories responsible for obtaining the specimens, as well as the Submitting laboratories where the genome data were generated and shared via GISAID, on which this research is based.

All Submitters of data may be contacted directly via www.gisaid.org

Authors are sorted alphabetically.

Accession ID	Originating Laboratory	Submitting Laboratory	Authors
EPI_ISL_8707664, EPI_ISL_8707673, EPI_ISL_8707679, EPI_ISL_8707683, EPI_ISL_8707688, EPI_ISL_8707690, EPI_ISL_8707769			
see above	Department of Bacteria, Parasites and Fungi, Statens Serum Institut, Copenhagen, Denmark	Statens Serum Institut Bioinformatics and Microbial Genomics	Danish Covid-19 Genome Consortium
EPI_ISL_8712911, EPI_ISL_8712912, EPI_ISL_8712915, EPI_ISL_8712919, EPI_ISL_8712926, EPI_ISL_8712927, EPI_ISL_8712934, EPI_ISL_8712935, EPI_ISL_8712939, EPI_ISL_8712940, EPI_ISL_8712943, EPI_ISL_8712944, EPI_ISL_8712945, EPI_ISL_8712946, EPI_ISL_8712947, EPI_ISL_8712948, EPI_ISL_8712950, EPI_ISL_8712954, EPI_ISL_8712957, EPI_ISL_8712958, EPI_ISL_8712959, EPI_ISL_8712960, EPI_ISL_8712961, EPI_ISL_8712962, EPI_ISL_8712963, EPI_ISL_8712964, EPI_ISL_8712966, EPI_ISL_8712967, EPI_ISL_8712968, EPI_ISL_8712970, EPI_ISL_8712972, EPI_ISL_8712973, EPI_ISL_8712974, EPI_ISL_8712975, EPI_ISL_8712981, EPI_ISL_8712984, EPI_ISL_8712992, EPI_ISL_8712994, EPI_ISL_8712995, EPI_ISL_8712997, EPI_ISL_8713001, EPI_ISL_8713002, EPI_ISL_8713003			
see above	Kasturba Hospital Molecular Lab - INSACOG	Kasturba hospital WGS	Jayanthi Shastri; Vidushi Chitalia

We gratefully acknowledge the following Authors from the Originating laboratories responsible for obtaining the specimens, as well as the Submitting laboratories where the genome data were generated and shared via GISAID, on which this research is based.

All Submitters of data may be contacted directly via www.gisaid.org

Authors are sorted alphabetically.

Acknowledgement EPI_SET Identifier: EPI_SET_20220527ba

Accession ID	Originating Laboratory	Submitting Laboratory	Authors
EPI_ISL_8707337, EPI_ISL_8707340, EPI_ISL_8707343, EPI_ISL_8707355, EPI_ISL_8707358, EPI_ISL_8707360, EPI_ISL_8707361, EPI_ISL_8707363, EPI_ISL_8707365, EPI_ISL_8707369, EPI_ISL_8707373, EPI_ISL_8707375, EPI_ISL_8707379, EPI_ISL_8707382, EPI_ISL_8707383, EPI_ISL_8707388, EPI_ISL_8707391, EPI_ISL_8707395, EPI_ISL_8707396, EPI_ISL_8707397, EPI_ISL_8707400, EPI_ISL_8707423, EPI_ISL_8707432, EPI_ISL_8707440, EPI_ISL_8707447, EPI_ISL_8707455, EPI_ISL_8707456, EPI_ISL_8707459, EPI_ISL_8707480, EPI_ISL_8707483, EPI_ISL_8707488, EPI_ISL_8707504, EPI_ISL_8707506, EPI_ISL_8707512, EPI_ISL_8707541, EPI_ISL_8707544, EPI_ISL_8707589, EPI_ISL_8707592, EPI_ISL_8707595, EPI_ISL_8707598, EPI_ISL_8707627, EPI_ISL_8707630, EPI_ISL_8707632, EPI_ISL_8707642, EPI_ISL_8707643, EPI_ISL_8707644, EPI_ISL_8707648, EPI_ISL_8707653, EPI_ISL_8707663			
see above	Department of Bacteria, Parasites and Fungi, Statens Serum Institut, Copenhagen, Denmark	Statens Serum Institut Bioinformatics and Microbial Genomics	Danish Covid-19 Genome Consortium
EPI_ISL_8707535	Lighthouse Lab in Glasgow	Wellcome Sanger Institute for the COVID-19 Genomics UK (COG-UK) Consortium	Anna Dominiczak and Alex Alderton; Carol Clugston; Cordelia Langford; David Gray; David K. Jackson; Dominic Kwiatkowski; Ewan Harrison; Harper VanSteenhouse; Ian Johnston; Jeffrey Barrett; John Sillitoe on behalf of the Wellcome Sanger Institute COVID-19 Surveillance Team; Roberto Amato; Sonia Goncalves; Yumi Kasai

We gratefully acknowledge the following Authors from the Originating laboratories responsible for obtaining the specimens, as well as the Submitting laboratories where the genome data were generated and shared via GISAID, on which this research is based.

All Submitters of data may be contacted directly via www.gisaid.org

Authors are sorted alphabetically.

Accession ID	Originating Laboratory	Submitting Laboratory	Authors
EPI_ISL_8706808, EPI_ISL_8706819, EPI_ISL_8706828, EPI_ISL_8706841, EPI_ISL_8706874, EPI_ISL_8706885, EPI_ISL_8706893, EPI_ISL_8706900, EPI_ISL_8706907, EPI_ISL_8706910, EPI_ISL_8706918, EPI_ISL_8706936, EPI_ISL_8706940, EPI_ISL_8706974, EPI_ISL_8706981, EPI_ISL_8707007, EPI_ISL_8707011, EPI_ISL_8707013, EPI_ISL_8707016, EPI_ISL_8707024, EPI_ISL_8707032, EPI_ISL_8707037, EPI_ISL_8707054, EPI_ISL_8707068, EPI_ISL_8707076, EPI_ISL_8707085, EPI_ISL_8707088, EPI_ISL_8707089, EPI_ISL_8707117, EPI_ISL_8707120, EPI_ISL_8707146, EPI_ISL_8707170, EPI_ISL_8707173, EPI_ISL_8707179, EPI_ISL_8707185, EPI_ISL_8707188, EPI_ISL_8707193, EPI_ISL_8707212, EPI_ISL_8707223, EPI_ISL_8707226, EPI_ISL_8707229, EPI_ISL_8707233, EPI_ISL_8707256, EPI_ISL_8707265, EPI_ISL_8707277, EPI_ISL_8707293, EPI_ISL_8707298, EPI_ISL_8707300, EPI_ISL_8707306, EPI_ISL_8707313			
see above	Department of Bacteria, Parasites and Fungi, Statens Serum Institut, Copenhagen, Denmark	Statens Serum Institut Bioinformatics and Microbial Genomics	Danish Covid-19 Genome Consortium

We gratefully acknowledge the following Authors from the Originating laboratories responsible for obtaining the specimens, as well as the Submitting laboratories where the genome data were generated and shared via GISAID, on which this research is based.

All Submitters of data may be contacted directly via www.gisaid.org

Authors are sorted alphabetically.

Accession ID	Originating Laboratory	Submitting Laboratory	Authors
EPI_ISL_8706216, EPI_ISL_8706227, EPI_ISL_8706231, EPI_ISL_8706239, EPI_ISL_8706243, EPI_ISL_8706256, EPI_ISL_8706275, EPI_ISL_8706276, EPI_ISL_8706281, EPI_ISL_8706286, EPI_ISL_8706295, EPI_ISL_8706305, EPI_ISL_8706309, EPI_ISL_8706320, EPI_ISL_8706339, EPI_ISL_8706348, EPI_ISL_8706366, EPI_ISL_8706370, EPI_ISL_8706433, EPI_ISL_8706477, EPI_ISL_8706483, EPI_ISL_8706492, EPI_ISL_8706507, EPI_ISL_8706510, EPI_ISL_8706523, EPI_ISL_8706525, EPI_ISL_8706548, EPI_ISL_8706554, EPI_ISL_8706557, EPI_ISL_8706561, EPI_ISL_8706616, EPI_ISL_8706627, EPI_ISL_8706630, EPI_ISL_8706634, EPI_ISL_8706656, EPI_ISL_8706660, EPI_ISL_8706686, EPI_ISL_8706708, EPI_ISL_8706710, EPI_ISL_8706721, EPI_ISL_8706726, EPI_ISL_8706730, EPI_ISL_8706743, EPI_ISL_8706748, EPI_ISL_8706765, EPI_ISL_8706783, EPI_ISL_8706790, EPI_ISL_8706794, EPI_ISL_8706801			
see above	Department of Bacteria, Parasites and Fungi, Statens Serum Institut, Copenhagen, Denmark	Statens Serum Institut Bioinformatics and Microbial Genomics	Danish Covid-19 Genome Consortium
EPI_ISL_8706738	Lighthouse Laboratory Plymouth	Wellcome Sanger Institute for the COVID-19 Genomics UK (COG-UK) Consortium	Cordelia Langford; David K. Jackson; Dominic Kwiatkowski; Ewan Harrison; Ian Johnston; Jeffrey Barrett; John Sillitoe on behalf of the Wellcome Sanger Institute COVID-19 Surveillance Team; Lighthouse Laboratory Plymouth and Alex Alderton; Roberto Amato; Sonia Goncalves

We gratefully acknowledge the following Authors from the Originating laboratories responsible for obtaining the specimens, as well as the Submitting laboratories where the genome data were generated and shared via GISAID, on which this research is based.

All Submitters of data may be contacted directly via www.gisaid.org

Authors are sorted alphabetically.

Accession ID	Originating Laboratory	Submitting Laboratory	Authors
EPI_ISL_8731067, EPI_ISL_8731069, EPI_ISL_8731074	CDPH VBL	California Department of Public Health	CDPH COVIDNet
EPI_ISL_8750817, EPI_ISL_8750827, EPI_ISL_8750828, EPI_ISL_8750829, EPI_ISL_8750830, EPI_ISL_8750833, EPI_ISL_8750834, EPI_ISL_8750835, EPI_ISL_8750836, EPI_ISL_8750837, EPI_ISL_8750838, EPI_ISL_8750839, EPI_ISL_8750840, EPI_ISL_8750841, EPI_ISL_8750843, EPI_ISL_8750845, EPI_ISL_8750846, EPI_ISL_8750847, EPI_ISL_8750848, EPI_ISL_8750849, EPI_ISL_8750850, EPI_ISL_8750851, EPI_ISL_8750852, EPI_ISL_8750853, EPI_ISL_8750854, EPI_ISL_8750855, EPI_ISL_8750856, EPI_ISL_8750857, EPI_ISL_8750859, EPI_ISL_8750861, EPI_ISL_8750865			
see above	Idaho Bureau of Laboratories	Idaho Bureau of Laboratories	Aimee Ceniseros; Christian Loera; Christopher Ball; Matthew Charles Burns; R. Beukelman; Robert L. Voermans
EPI_ISL_8741961, EPI_ISL_8742089	Lighthouse Lab in Alderley Park	Wellcome Sanger Institute for the COVID-19 Genomics UK (COG-UK) Consortium	Cordelia Langford; David K. Jackson; Dominic Kwiatkowski; Ewan Harrison; Ian Johnston; Jacquelyn Wynn; Jeffrey Barrett; John Sillitoe on behalf of the Wellcome Sanger Institute COVID-19 Surveillance Team; Mairead Hyland; Roberto Amato; Sonia Goncalves; The Lighthouse Lab in Alderley Park and Alex Alderton
EPI_ISL_8739949, EPI_ISL_8741098, EPI_ISL_8741193, EPI_ISL_8741905, EPI_ISL_8742094, EPI_ISL_8742139, EPI_ISL_8743347, EPI_ISL_8743488			
see above	Lighthouse Lab in Glasgow	Wellcome Sanger Institute for the COVID-19 Genomics UK (COG-UK) Consortium	Anna Dominiczak and Alex Alderton; Carol Clugston; Cordelia Langford; David Gray; David K. Jackson; Dominic Kwiatkowski; Ewan Harrison; Harper VanSteenhouse; Ian Johnston; Jeffrey Barrett; John Sillitoe on behalf of the Wellcome Sanger Institute COVID-19 Surveillance Team; Roberto Amato; Sonia Goncalves; Yumi Kasai
EPI_ISL_8743240, EPI_ISL_8743465, EPI_ISL_8743484, EPI_ISL_8743485	Lighthouse Lab in Milton Keynes	Wellcome Sanger Institute for the COVID-19 Genomics UK (COG-UK) Consortium	Cordelia Langford; David K. Jackson; Dominic Kwiatkowski; Ewan Harrison; Ian Johnston; Jeffrey Barrett; John Sillitoe on behalf of the Wellcome Sanger Institute COVID-19 Surveillance Team; Roberto Amato; Sonia Goncalves; The Lighthouse Lab in Milton Keynes and Alex Alderton
EPI_ISL_8739590, EPI_ISL_8739603, EPI_ISL_8739639, EPI_ISL_8739848, EPI_ISL_8739904	Lighthouse Laboratory Plymouth	Wellcome Sanger Institute for the COVID-19 Genomics UK (COG-UK) Consortium	Cordelia Langford; David K. Jackson; Dominic Kwiatkowski; Ewan Harrison; Ian Johnston; Jeffrey Barrett; John Sillitoe on behalf of the Wellcome Sanger Institute COVID-19 Surveillance Team; Lighthouse Laboratory Plymouth and Alex Alderton; Roberto Amato; Sonia Goncalves
EPI_ISL_8730816, EPI_ISL_8730819, EPI_ISL_8730822, EPI_ISL_8730826, EPI_ISL_8730828, EPI_ISL_8730832, EPI_ISL_8730835			
see above	Premier Laboratory Services	BioInfoExperts	Amy Feehan; Ben Lain; Chris Huston; David J. Nolan; Judy Crabtree; Julia-Garcia-Diaz; Lucio Miele; Rebecca Rose; Samual Moot; Susanna L. Lamers; Tessa LaFleur
EPI_ISL_8751571	PrimBio Research Institute LLC	PrimBio Research Institute LLC	Diana Z. Ye; Yang Liu
EPI_ISL_8739601, EPI_ISL_8743432	Rosalind Franklin Laboratory	Wellcome Sanger Institute for the COVID-19 Genomics UK (COG-UK) Consortium	Cordelia Langford; David K. Jackson; Dominic Kwiatkowski; Donald Fraser; Ewan Harrison; Ian Johnston; Jeffrey Barrett; John Sillitoe on behalf of the Wellcome Sanger Institute COVID-19 Surveillance Team; Rob Howes; Roberto Amato; Sonia Goncalves; Suki Lee; The Rosalind Franklin Laboratory and Alex Alderton
EPI_ISL_8744182	Scientific Diagnostic Centre Pvt. Ltd., Ahmedabad	Gujarat Biotechnology Research Centre	Akhilesh Modi; Apurvasinh Puvar; Bhadreshsinh Gohil; Chaitanya Joshi; Disha Vora; Janvi Raval; Jaykumar Rangani; Madhvi Joshi; Moksha Narechania; Nimesh Patel; Nitin Savaliya; NitinShukla; Priyank Chavda; Ramesh Pandit; Roshani Mishra; Sonal Sharma; Tasnim Trivedi; Zarna Patel
EPI_ISL_8727742	State Laboratories Division, Hawaii State Department of Health	State Laboratories Division, Hawaii State Department of Health	Ayana Garnet; Briana Ofilas; Cheryl-lynn Daquip; Cheyenne Barela; Daniel Strange; Drew Kuwazaki; Edward Desmond; Jeffrey Au; Mark Nagata; Pamela O'Brien; Razvan Sultana; Remedios Gose; Samantha Cotter; Samantha Sruba
EPI_ISL_8744253, EPI_ISL_8744274, EPI_ISL_8750077, EPI_ISL_8750095, EPI_ISL_8750103, EPI_ISL_8750240, EPI_ISL_8750270, EPI_ISL_8750308, EPI_ISL_8750356			
see above	UW Virology Lab	UW Virology Lab	Alexander Greninger; Hong Xie; Isabel Arnould; Keith R Jerome; Meei-Li Huang; Nathan Breit; Patrick Mathias; Pavitra Roychoudhury; Pooneh Hajian; Ricardo Perez; Robert J. Livingston; Saraswathi Sathees; Sean Ellis; Seffir T. Wendm; Shah Mohamed Bakhash; Tien V. Nguyen
EPI_ISL_8733062, EPI_ISL_8733065, EPI_ISL_8733067, EPI_ISL_8733070, EPI_ISL_8733071, EPI_ISL_8733076, EPI_ISL_8733077, EPI_ISL_8733079, EPI_ISL_8733080, EPI_ISL_8733081, EPI_ISL_8733086, EPI_ISL_8733089, EPI_ISL_8733091, EPI_ISL_8733093, EPI_ISL_8733099, EPI_ISL_8733108, EPI_ISL_8733115, EPI_ISL_8733122, EPI_ISL_8733123, EPI_ISL_8733126, EPI_ISL_8733128, EPI_ISL_8733130, EPI_ISL_8733131, EPI_ISL_8733132, EPI_ISL_8733135, EPI_ISL_8733140, EPI_ISL_8733144, EPI_ISL_8733145, EPI_ISL_8733146, EPI_ISL_8733148, EPI_ISL_8733150, EPI_ISL_8733151, EPI_ISL_8733160, EPI_ISL_8733162, EPI_ISL_8733163, EPI_ISL_8733165, EPI_ISL_8733173, EPI_ISL_8733183, EPI_ISL_8733185, EPI_ISL_8733186, EPI_ISL_8733187, EPI_ISL_8733188, EPI_ISL_8733190, EPI_ISL_8733191, EPI_ISL_8733192, EPI_ISL_8733193, EPI_ISL_8733194, EPI_ISL_8733195, EPI_ISL_8733196, EPI_ISL_8733197, EPI_ISL_8733198, EPI_ISL_8733201, EPI_ISL_8733202, EPI_ISL_8733203, EPI_ISL_8733205, EPI_ISL_8733206, EPI_ISL_8733207, EPI_ISL_8733208, EPI_ISL_8733210, EPI_ISL_8733211, EPI_ISL_8733212, EPI_ISL_8733213, EPI_ISL_8733214, EPI_ISL_8733215, EPI_ISL_8733217, EPI_ISL_8733218, EPI_ISL_8733221, EPI_ISL_8733222, EPI_ISL_8733223, EPI_ISL_8733225, EPI_ISL_8733227, EPI_ISL_8733228, EPI_ISL_8733232, EPI_ISL_8733234, EPI_ISL_8733240, EPI_ISL_8733243, EPI_ISL_8733244, EPI_ISL_8733247, EPI_ISL_8733251, EPI_ISL_8733254, EPI_ISL_8733255, EPI_ISL_8733256, EPI_ISL_8733259, EPI_ISL_8733260, EPI_ISL_8733261, EPI_ISL_8733262, EPI_ISL_8733263, EPI_ISL_8733264, EPI_ISL_8733267, EPI_ISL_8733271, EPI_ISL_8733276, EPI_ISL_8733277, EPI_ISL_8733284, EPI_ISL_8733285, EPI_ISL_8733302, EPI_ISL_8733303, EPI_ISL_8733305, EPI_ISL_8733307, EPI_ISL_8733308, EPI_ISL_8733309, EPI_ISL_8733310, EPI_ISL_8733322, EPI_ISL_8733333, EPI_ISL_8733344, EPI_ISL_8733345, EPI_ISL_8733346, EPI_ISL_8733347, EPI_ISL_8733348, EPI_ISL_8733349, EPI_ISL_8733351, EPI_ISL_8733356, EPI_ISL_8733357, EPI_ISL_8733358, EPI_ISL_8733369, EPI_ISL_8733375, EPI_ISL_8733379, EPI_ISL_8733380, EPI_ISL_8733381, EPI_ISL_8733383, EPI_ISL_8733388, EPI_ISL_8733389, EPI_ISL_8733392, EPI_ISL_8733395, EPI_ISL_8733397, EPI_ISL_8733398, EPI_ISL_8733402, EPI_ISL_8733403, EPI_ISL_8733405, EPI_ISL_8733406, EPI_ISL_8733409, EPI_ISL_8733410, EPI_ISL_8733423, EPI_ISL_8733425, EPI_ISL_8733430, EPI_ISL_8733433, EPI_ISL_8733434, EPI_ISL_8733438, EPI_ISL_8733439, EPI_ISL_8733441, EPI_ISL_8733442, EPI_ISL_8733448, EPI_ISL_8733454, EPI_ISL_8733462, EPI_ISL_8733465, EPI_ISL_8733466, EPI_ISL_8733474, EPI_ISL_8733544, EPI_ISL_8733568, EPI_ISL_8733618, EPI_ISL_8733622, EPI_ISL_8733623, EPI_ISL_8733624, EPI_ISL_8733625, EPI_ISL_8733626, EPI_ISL_8733627, EPI_ISL_8733628, EPI_ISL_8733630, EPI_ISL_8733631, EPI_ISL_8733633, EPI_ISL_8733638, EPI_ISL_8733641, EPI_ISL_8733642, EPI_ISL_8733645, EPI_ISL_8733646, EPI_ISL_8733652, EPI_ISL_8733656, EPI_ISL_8733667, EPI_ISL_8733676, EPI_ISL_8733678, EPI_ISL_8733686, EPI_ISL_8733693, EPI_ISL_8733699, EPI_ISL_8733703, EPI_ISL_8733712, EPI_ISL_8733715, EPI_ISL_8733716, EPI_ISL_8733719, EPI_ISL_8733721, EPI_ISL_8733722, EPI_ISL_8733724, EPI_ISL_8733726, EPI_ISL_8733730, EPI_ISL_8733733, EPI_ISL_8733735, EPI_ISL_8733737			
see above	University of Oregon COVID-19 MAP Laboratory	University of Oregon Genomics and Cell Charactericzation Core Facility (GC3F)	Ariana White; Demi Glidden; Douglas Turnbull; Jason Carriere; Jason Sydes; Jeff Bishop; Megan Criss; Peter Batzel; Tina Arredondo
EPI_ISL_8751533	Utah Public Health Laboratory	Utah Public Health Laboratory	Erin L. Young; John Arnn; Kelly F. Oakeson; Olinto Linares-Perdomo; Pooja Gupta; Tom Iverson

We gratefully acknowledge the following Authors from the Originating laboratories responsible for obtaining the specimens, as well as the Submitting laboratories where the genome data were generated and shared via GISAID, on which this research is based.

All Submitters of data may be contacted directly via www.gisaid.org

Authors are sorted alphabetically.

Acknowledgement EPI_SET Identifier: EPI_SET_20220527rw

Accession ID	Originating Laboratory	Submitting Laboratory	Authors
EPI_ISL_8754018	Amirabad Virology Lab, Detection Unit	National Influenza Center	A Nejati; Asghar Abdoli; J Yavarian; K Sadeghi; NZ Shafiei Jandaghi; Sevrin Zadheidar and T Mokhtari Azad; V Salimi
EPI_ISL_8754645, EPI_ISL_8754697	Berkshire and Surrey Pathology Services Lighthouse Laboratory	Wellcome Sanger Institute for the COVID-19 Genomics UK (COG-UK) Consortium	Berkshire and Surrey Pathology Services Lighthouse Laboratory and Alex Alderton; Cordelia Langford; David K. Jackson; Dominic Kwiatkowski; Ewan Harrison; Ian Johnston; Jeffrey Barrett; John Sillitoe on behalf of the Wellcome Sanger Institute COVID-19 Surveillance Team; Roberto Amato; Sonia Goncalves
EPI_ISL_8755609	Integrated Covid Hub North East	Wellcome Sanger Institute for the COVID-19 Genomics UK (COG-UK) Consortium	Cordelia Langford; David K. Jackson; Dominic Kwiatkowski; Ewan Harrison; Ian Johnston; Integrated Covid Hub North East and Alex Alderton; Jeffrey Barrett; John Sillitoe on behalf of the Wellcome Sanger Institute COVID-19 Surveillance Team; Roberto Amato; Sonia Goncalves
EPI_ISL_8754374, EPI_ISL_8764986, EPI_ISL_8764987, EPI_ISL_8764988, EPI_ISL_8764999, EPI_ISL_8765002, EPI_ISL_8765011, EPI_ISL_8765014, EPI_ISL_8765028, EPI_ISL_8765030, EPI_ISL_8765031, EPI_ISL_8765033, EPI_ISL_8765034, EPI_ISL_8765037, EPI_ISL_8765039, EPI_ISL_8765041, EPI_ISL_8765045, EPI_ISL_8765046			
see above	Lab. Microbiologia e Virologia Cotugno A.O. dei Colli	TIGEM	Antonio Grimaldi Patrizia Annunziata Francesco Panariello Claudia Tiberio Teresa Giuliano Valentina Bouche Chiara Colantuono Michela Daniele Lucio Di Filippo Anna Manfredi Marcello Salvi Antonio Limone Luigi Atripaldi Andrea Ballabio Davide Cacchiarelli
EPI_ISL_8754576	Lighthouse Lab in Alderley Park	Wellcome Sanger Institute for the COVID-19 Genomics UK (COG-UK) Consortium	Cordelia Langford; David K. Jackson; Dominic Kwiatkowski; Ewan Harrison; Ian Johnston; Jacquelyn Wynn; Jeffrey Barrett; John Sillitoe on behalf of the Wellcome Sanger Institute COVID-19 Surveillance Team; Mairead Hyland; Roberto Amato; Sonia Goncalves; The Lighthouse Lab in Alderley Park and Alex Alderton
EPI_ISL_8751688	NJDOH, Public Health and Environmental Laboratories	NJ_PHEL	Byeong Jeong; Chelsea San Filippo; Dana Woell; Lindsey Bodnar; Maria-Magdalene Pugliese; Mohammad M. Ali; Shiv K. Verma
EPI_ISL_8757535, EPI_ISL_8757536, EPI_ISL_8760108, EPI_ISL_8760117, EPI_ISL_8760121, EPI_ISL_8760127, EPI_ISL_8760144, EPI_ISL_8760148, EPI_ISL_8760165, EPI_ISL_8760169, EPI_ISL_8760215, EPI_ISL_8760235, EPI_ISL_8760241, EPI_ISL_8760841, EPI_ISL_8760844, EPI_ISL_8760872, EPI_ISL_8760883, EPI_ISL_8760950, EPI_ISL_8761055			
see above	Respiratory Virus Unit, Microbiology Services Colindale, Public Health England	COVID-19 Genomics UK (COG-UK) Consortium	PHE Covid Sequencing Team

We gratefully acknowledge the following Authors from the Originating laboratories responsible for obtaining the specimens, as well as the Submitting laboratories where the genome data were generated and shared via GISAID, on which this research is based.

All Submitters of data may be contacted directly via www.gisaid.org

Authors are sorted alphabetically.

Accession ID	Originating Laboratory	Submitting Laboratory	Authors
EPI_ISL_8769739	CNC PATH LAB	ILBS	Arjun Bhugra; Chhagan Bihari Sharma; Ekta Gupta; Pramod Gautam; Rahul Garg; Reshu Agarwal; Shiv Kumar Sarin; Urvinder Kaur; Varun Suroliya
EPI_ISL_8769645	CORE Diagnostics Private Limited	ILBS	Arjun Bhugra; Chhagan Bihari Sharma; Ekta Gupta; Pramod Gautam; Rahul Garg; Reshu Agarwal; Shiv Kumar Sarin; Urvinder Kaur; Varun Suroliya
EPI_ISL_8767333, EPI_ISL_8767339, EPI_ISL_8767340, EPI_ISL_8767342, EPI_ISL_8767349, EPI_ISL_8767352, EPI_ISL_8767358, EPI_ISL_8767359, EPI_ISL_8767361, EPI_ISL_8767364, EPI_ISL_8767368, EPI_ISL_8767369, EPI_ISL_8767372, EPI_ISL_8767374, EPI_ISL_8767378, EPI_ISL_8767397, EPI_ISL_8767399, EPI_ISL_8767401, EPI_ISL_8767405, EPI_ISL_8767406, EPI_ISL_8767407, EPI_ISL_8767410, EPI_ISL_8767413, EPI_ISL_8767414, EPI_ISL_8767417, EPI_ISL_8767425, EPI_ISL_8767427, EPI_ISL_8767430, EPI_ISL_8767432, EPI_ISL_8767434, EPI_ISL_8767439, EPI_ISL_8767440, EPI_ISL_8767442, EPI_ISL_8767448, EPI_ISL_8767449, EPI_ISL_8767451, EPI_ISL_8767452, EPI_ISL_8767453, EPI_ISL_8767455, EPI_ISL_8767456, EPI_ISL_8767460, EPI_ISL_8767467, EPI_ISL_8767471, EPI_ISL_8767473, EPI_ISL_8767474, EPI_ISL_8767476, EPI_ISL_8767479, EPI_ISL_8767482, EPI_ISL_8767483, EPI_ISL_8767486, EPI_ISL_8767502, EPI_ISL_8767505, EPI_ISL_8767508, EPI_ISL_8767516, EPI_ISL_8767518, EPI_ISL_8767519, EPI_ISL_8767521, EPI_ISL_8767522, EPI_ISL_8767528, EPI_ISL_8767530, EPI_ISL_8767533, EPI_ISL_8767535, EPI_ISL_8767540, EPI_ISL_8767542, EPI_ISL_8767546, EPI_ISL_8767548, EPI_ISL_8767549, EPI_ISL_8767553, EPI_ISL_8767554, EPI_ISL_8767557, EPI_ISL_8767559, EPI_ISL_8767560, EPI_ISL_8767561, EPI_ISL_8767563, EPI_ISL_8767567, EPI_ISL_8767568, EPI_ISL_8767570, EPI_ISL_8767576, EPI_ISL_8767577, EPI_ISL_8767579, EPI_ISL_8767586, EPI_ISL_8767587, EPI_ISL_8767588, EPI_ISL_8767593, EPI_ISL_8767594, EPI_ISL_8767595, EPI_ISL_8767600, EPI_ISL_8767603, EPI_ISL_8767604, EPI_ISL_8767605, EPI_ISL_8767608, EPI_ISL_8767614, EPI_ISL_8767615, EPI_ISL_8767616, EPI_ISL_8767617, EPI_ISL_8767625, EPI_ISL_8767627, EPI_ISL_8767633, EPI_ISL_8767634, EPI_ISL_8767637, EPI_ISL_8767647, EPI_ISL_8767652, EPI_ISL_8767656, EPI_ISL_8767657, EPI_ISL_8767660, EPI_ISL_8767662, EPI_ISL_8767664, EPI_ISL_8767669, EPI_ISL_8767671, EPI_ISL_8767675, EPI_ISL_8767677, EPI_ISL_8767679, EPI_ISL_8767681, EPI_ISL_8767684, EPI_ISL_8767685, EPI_ISL_8767686, EPI_ISL_8767689, EPI_ISL_8767694, EPI_ISL_8767695, EPI_ISL_8767708, EPI_ISL_8767712, EPI_ISL_8767713, EPI_ISL_8767718, EPI_ISL_8767719, EPI_ISL_8767721, EPI_ISL_8767722, EPI_ISL_8767723, EPI_ISL_8767726, EPI_ISL_8767728, EPI_ISL_8767731, EPI_ISL_8767735, EPI_ISL_8767740, EPI_ISL_8767748, EPI_ISL_8767752, EPI_ISL_8767753, EPI_ISL_8767755, EPI_ISL_8767756, EPI_ISL_8767757, EPI_ISL_8767760, EPI_ISL_8767765, EPI_ISL_8767768, EPI_ISL_8767771, EPI_ISL_8767777, EPI_ISL_8767778, EPI_ISL_8767783, EPI_ISL_8767784, EPI_ISL_8767785, EPI_ISL_8767786, EPI_ISL_8767788, EPI_ISL_8767792, EPI_ISL_8767799, EPI_ISL_8767803, EPI_ISL_8767804, EPI_ISL_8767808, EPI_ISL_8767811, EPI_ISL_8767815, EPI_ISL_8767819, EPI_ISL_8767820, EPI_ISL_8767825, EPI_ISL_8767833, EPI_ISL_8767835, EPI_ISL_8767836, EPI_ISL_8767841, EPI_ISL_8767862, EPI_ISL_8767865, EPI_ISL_8767866, EPI_ISL_8767873, EPI_ISL_8767884, EPI_ISL_8767891, EPI_ISL_8767894, EPI_ISL_8767895, EPI_ISL_8767896, EPI_ISL_8767900, EPI_ISL_8767908, EPI_ISL_8767912, EPI_ISL_8767913, EPI_ISL_8767914, EPI_ISL_8767915, EPI_ISL_8767917, EPI_ISL_8767922, EPI_ISL_8767924, EPI_ISL_8767929, EPI_ISL_8767930, EPI_ISL_8767932, EPI_ISL_8767938, EPI_ISL_8767939, EPI_ISL_8767942, EPI_ISL_8767949, EPI_ISL_8767957, EPI_ISL_8767958, EPI_ISL_8767965, EPI_ISL_8767967, EPI_ISL_8767977, EPI_ISL_8767978, EPI_ISL_8767981, EPI_ISL_8767986, EPI_ISL_8767992, EPI_ISL_8767993, EPI_ISL_8768003, EPI_ISL_8768007, EPI_ISL_8768009, EPI_ISL_8768012, EPI_ISL_8768015, EPI_ISL_8768018, EPI_ISL_8768023, EPI_ISL_8768024, EPI_ISL_8768028, EPI_ISL_8768031, EPI_ISL_8768032, EPI_ISL_8768033, EPI_ISL_8768036, EPI_ISL_8768037, EPI_ISL_8768038, EPI_ISL_8768043, EPI_ISL_8768059, EPI_ISL_8768062, EPI_ISL_8768064, EPI_ISL_8768065, EPI_ISL_8768066, EPI_ISL_8768068, EPI_ISL_8768073, EPI_ISL_8768077, EPI_ISL_8768078, EPI_ISL_8768088, EPI_ISL_8768089, EPI_ISL_8768092, EPI_ISL_8768101, EPI_ISL_8768103, EPI_ISL_8768104, EPI_ISL_8768106, EPI_ISL_8768107, EPI_ISL_8768116, EPI_ISL_8768128, EPI_ISL_8768137, EPI_ISL_8768139, EPI_ISL_8768143, EPI_ISL_8768146, EPI_ISL_8768152, EPI_ISL_8768158, EPI_ISL_8768163, EPI_ISL_8768166, EPI_ISL_8768170, EPI_ISL_8768174, EPI_ISL_8768182, EPI_ISL_8768183, EPI_ISL_8768195, EPI_ISL_8768196, EPI_ISL_8768199, EPI_ISL_8768209, EPI_ISL_8768212, EPI_ISL_8768215, EPI_ISL_8768217, EPI_ISL_8768219, EPI_ISL_8768221, EPI_ISL_8768227, EPI_ISL_8768230, EPI_ISL_8768238, EPI_ISL_8768245, EPI_ISL_8768247, EPI_ISL_8768249, EPI_ISL_8768252, EPI_ISL_8768253, EPI_ISL_8768255, EPI_ISL_8768256, EPI_ISL_8768259, EPI_ISL_8768265, EPI_ISL_8768267, EPI_ISL_8768273, EPI_ISL_8768275, EPI_ISL_8768276, EPI_ISL_8768278, EPI_ISL_8768283, EPI_ISL_8768284, EPI_ISL_8768285, EPI_ISL_8768290, EPI_ISL_8768298, EPI_ISL_8768299, EPI_ISL_8768301, EPI_ISL_8768303, EPI_ISL_8768305, EPI_ISL_8768307, EPI_ISL_8768308, EPI_ISL_8768309, EPI_ISL_8768310, EPI_ISL_8768312, EPI_ISL_8768315, EPI_ISL_8768316, EPI_ISL_8768318, EPI_ISL_8768321, EPI_ISL_8768326, EPI_ISL_8768330, EPI_ISL_8768331, EPI_ISL_8768332, EPI_ISL_8768337, EPI_ISL_8768341, EPI_ISL_8768351, EPI_ISL_8768365, EPI_ISL_8768371, EPI_ISL_8768378, EPI_ISL_8768382, EPI_ISL_8768383, EPI_ISL_8768386, EPI_ISL_8768393, EPI_ISL_8768395, EPI_ISL_8768398, EPI_ISL_8768400, EPI_ISL_8768403			
see above	Department of Bacteria, Parasites and Fungi, Statens Serum Institut, Copenhagen, Denmark	Statens Serum Institut Bioinformatics and Microbial Genomics	Danish Covid-19 Genome Consortium
EPI_ISL_8769564	House of Diagnostics	ILBS	Arjun Bhugra; Chhagan Bihari Sharma; Ekta Gupta; Pramod Gautam; Rahul Garg; Reshu Agarwal; Shiv Kumar Sarin; Urvinder Kaur; Varun Suroliya
EPI_ISL_8791239, EPI_ISL_8791244	Invenimus AG	Institute of Medical Virology, University of Zurich	Alexandra Trkola; Annette Audigé; Catharine Aquino; Cyril Shah; Daniel Ehrsam; Gabriela Ziltener; Guido Bloemberg; Hubert Rehrauer; Isabel Stürmer; Joel Wirz; Jon Huder; Jürg Böni; Kevin Steiner; Maria Grünberg; Maryam Zaheri; Michael Huber; Riccarda Capaul; Stefan Schmutz; Verena Kufner; Weihong Qi
EPI_ISL_8791242	Kantonsspital Baden AG	Institute of Medical Virology, University of Zurich	Alexandra Trkola; Annette Audigé; Catharine Aquino; Cyril Shah; Daniel Ehrsam; Gabriela Ziltener; Guido Bloemberg; Hubert Rehrauer; Isabel Stürmer; Joel Wirz; Jon Huder; Jürg Böni; Kevin Steiner; Maria Grünberg; Maryam Zaheri; Michael Huber; Riccarda Capaul; Stefan Schmutz; Verena Kufner; Weihong Qi
EPI_ISL_8787930, EPI_ISL_8787931, EPI_ISL_8787932, EPI_ISL_8787934, EPI_ISL_8787935, EPI_ISL_8787936, EPI_ISL_8787937, EPI_ISL_8787938, EPI_ISL_8787941, EPI_ISL_8787942, EPI_ISL_8787943, EPI_ISL_8787944, EPI_ISL_8787945, EPI_ISL_8787946, EPI_ISL_8788031, EPI_ISL_8788039, EPI_ISL_8791189, EPI_ISL_8791191, EPI_ISL_8791194, EPI_ISL_8791272			
see above	Kantonsspital Winterthur	Institute of Medical Virology, University of Zurich	Alexandra Trkola; Annette Audigé; Catharine Aquino; Cyril Shah; Daniel Ehrsam; Gabriela Ziltener; Guido Bloemberg; Hubert Rehrauer; Isabel Stürmer; Joel Wirz; Jon Huder; Jürg Böni; Kevin Steiner; Maria Grünberg; Maryam Zaheri; Michael Huber; Riccarda Capaul; Stefan Schmutz; Verena Kufner; Weihong Qi
EPI_ISL_8770363	LABORATOIRE BIOALLIANCE	CNR Virus des Infections Respiratoires - France SUD	Antonin Bal; Bruno Lina; Bruno Simon; Gregory Destras; Gwendolyne Burfin; Hadrien Regue; Laurence Josset; Martine Valette; Quentin Semanas; ThÃ©ophile Boyer
EPI_ISL_8769742	Medibird Diagnostics	ILBS	Arjun Bhugra; Chhagan Bihari Sharma; Ekta Gupta; Pramod Gautam; Rahul Garg; Reshu Agarwal; Shiv Kumar Sarin; Urvinder Kaur; Varun Suroliya
EPI_ISL_8769667, EPI_ISL_8769670	NRL, Dr Lal Path Lab Ltds	ILBS	Arjun Bhugra; Chhagan Bihari Sharma; Ekta Gupta; Pramod Gautam; Rahul Garg; Reshu Agarwal; Shiv Kumar Sarin; Urvinder Kaur; Varun Suroliya
EPI_ISL_8770483, EPI_ISL_8770484, EPI_ISL_8770485, EPI_ISL_8770486, EPI_ISL_8770487, EPI_ISL_8770488, EPI_ISL_8770489, EPI_ISL_8770490, EPI_ISL_8770491, EPI_ISL_8770492, EPI_ISL_8770493, EPI_ISL_8770494, EPI_ISL_8770495, EPI_ISL_8770496, EPI_ISL_8770497, EPI_ISL_8770498, EPI_ISL_8770499, EPI_ISL_8770500, EPI_ISL_8770501, EPI_ISL_8770502, EPI_ISL_8770504, EPI_ISL_8770505, EPI_ISL_8770506, EPI_ISL_8770507, EPI_ISL_8770509, EPI_ISL_8770510, EPI_ISL_8770511, EPI_ISL_8770512, EPI_ISL_8770513, EPI_ISL_8770514, EPI_ISL_8770515, EPI_ISL_8770516, EPI_ISL_8770517, EPI_ISL_8770518, EPI_ISL_8770519, EPI_ISL_8770520, EPI_ISL_8770521, EPI_ISL_8770522, EPI_ISL_8770523, EPI_ISL_8770525, EPI_ISL_8770526, EPI_ISL_8770527, EPI_ISL_8770528, EPI_ISL_8770529, EPI_ISL_8770530, EPI_ISL_8770531, EPI_ISL_8770532, EPI_ISL_8770533, EPI_ISL_8770534, EPI_ISL_8770535, EPI_ISL_8770536, EPI_ISL_8770538, EPI_ISL_8770539, EPI_ISL_8770540, EPI_ISL_8770541, EPI_ISL_8770542, EPI_ISL_8770543			
see above	National Public Health Laboratory, National Centre for Infectious Diseases	National Public Health Laboratory, National Centre for Infectious Diseases	Benny Yeo; Lin Cui; Raymond Tzer Pin Lin; Royce Ang; Samuel Loo; Zhenyang Zhou
EPI_ISL_8786600, EPI_ISL_8786641, EPI_ISL_8786643	Nebraska Public Health Laboratory	NPHL COVID-19 Response Team	NPHL COVID-19 Response Team
EPI_ISL_8784572, EPI_ISL_8784610	Originating lab: Wales Specialist Virology Centre Sequencing lab: Pathogen Genomics Unit	Public Health Wales Microbiology Cardiff Wales Specialist Virology Centre	Alec Birchley; Alexander Adams; Amy Gaskin; Angela Marchbank; Bree Gatica-Wilcox; Catherine Moore; Jason Coombes; Joanne Watkins; Joel Southgate; Johnathan Evans; Laura Gifford; Lauren Gilbert; Lee Graham; Malorie Perry; Matthew Bull; Nicole Pacchiarini; Sally Corden; Sara Kumziene-Summerhayes; Sara Rey; Sarah Taylor; Simon Cottrell; Sophie Jones; Tom Connor
EPI_ISL_8783172	Polski Bank Komórek Macierzystych S.A.	WSSE w Warszawie	Dorota Wagrocka - Roczniak
EPI_ISL_8782794, EPI_ISL_8782795, EPI_ISL_8782796, EPI_ISL_8782799, EPI_ISL_8782800, EPI_ISL_8782803	Sarawak General Hospital (Kuching)	Institute of Health and Community Medicine	Chan Chia Jui; Chua Hock Hin; David Perera; Ooi Mong How; Tonnii Sia Loong Loong
EPI_ISL_8782793, EPI_ISL_8782797, EPI_ISL_8782798	Sibu Hospital (Sibu, Sarawak)	Institute of Health and Community Medicine	Chan Chia Jui; Chua Hock Hin; David Perera; Ooi Mong How; Tonnii Sia Loong Loong
EPI_ISL_8787953, EPI_ISL_8791204, EPI_ISL_8791240	Stadtspital Waid	Institute of Medical Virology, University of Zurich	Alexandra Trkola; Annette Audigé; Catharine Aquino; Cyril Shah; Daniel Ehrsam; Gabriela Ziltener; Guido Bloemberg; Hubert Rehrauer; Isabel Stürmer; Joel Wirz; Jon Huder; Jürg Böni; Kevin Steiner; Maria Grünberg; Maryam Zaheri; Michael Huber; Riccarda Capaul; Stefan Schmutz; Verena Kufner; Weihong Qi
EPI_ISL_8791232	USZ Flughafen	Institute of Medical Virology, University of Zurich	Alexandra Trkola; Annette Audigé; Catharine Aquino; Cyril Shah; Daniel Ehrsam; Gabriela Ziltener; Guido Bloemberg; Hubert Rehrauer; Isabel Stürmer; Joel Wirz; Jon Huder; Jürg Böni; Kevin Steiner; Maria Grünberg; Maryam Zaheri; Michael Huber; Riccarda Capaul; Stefan Schmutz; Verena Kufner; Weihong Qi
EPI_ISL_8787950, EPI_ISL_8787952, EPI_ISL_8787955, EPI_ISL_8787961, EPI_ISL_8787963, EPI_ISL_8787965, EPI_ISL_8787967, EPI_ISL_8787968, EPI_ISL_8787970, EPI_ISL_8787971, EPI_ISL_8787973, EPI_ISL_8787974, EPI_ISL_8787975, EPI_ISL_8787976, EPI_ISL_8787997, EPI_ISL_8788000, EPI_ISL_8788004, EPI_ISL_8788007, EPI_ISL_8788019, EPI_ISL_8791214, EPI_ISL_8791216, EPI_ISL_8791217, EPI_ISL_8791218, EPI_ISL_8791219, EPI_ISL_8791221, EPI_ISL_8791222, EPI_ISL_8791223, EPI_ISL_8791233, EPI_ISL_8791234, EPI_ISL_8791235, EPI_ISL_8791236, EPI_ISL_8791237, EPI_ISL_8791249, EPI_ISL_8791251, EPI_ISL_8791252, EPI_ISL_8791253			
see above	Universität Zürich	Institute of Medical Virology, University of Zurich	Alexandra Trkola; Annette Audigé; Catharine Aquino; Cyril Shah; Daniel Ehrsam; Gabriela Ziltener; Guido Bloemberg; Hubert Rehrauer; Isabel Stürmer; Joel Wirz; Jon Huder; Jürg Böni; Kevin Steiner; Maria Grünberg; Maryam Zaheri; Michael Huber; Riccarda Capaul; Stefan Schmutz; Verena Kufner; Weihong Qi
EPI_ISL_8788011, EPI_ISL_8788015, EPI_ISL_8791202	Universitäts-Kinderspital Zürich	Institute of Medical Virology, University of Zurich	Alexandra Trkola; Annette Audigé; Catharine Aquino; Cyril Shah; Daniel Ehrsam; Gabriela Ziltener; Guido Bloemberg; Hubert Rehrauer; Isabel Stürmer; Joel Wirz; Jon Huder; Jürg Böni; Kevin Steiner; Maria Grünberg; Maryam Zaheri; Michael Huber; Riccarda Capaul; Stefan Schmutz; Verena Kufner; Weihong Qi
EPI_ISL_8787954, EPI_ISL_8787978, EPI_ISL_8787980, EPI_ISL_8787983, EPI_ISL_8787990, EPI_ISL_8787991, EPI_ISL_8787992, EPI_ISL_8787994, EPI_ISL_8787995, EPI_ISL_8791195, EPI_ISL_8791197, EPI_ISL_8791199, EPI_ISL_8791200, EPI_ISL_8791205, EPI_ISL_8791206, EPI_ISL_8791208, EPI_ISL_8791209, EPI_ISL_8791211, EPI_ISL_8791243, EPI_ISL_8791245, EPI_ISL_8791246, EPI_ISL_8791247, EPI_ISL_8791254, EPI_ISL_8791256, EPI_ISL_8791260, EPI_ISL_8791261, EPI_ISL_8791264, EPI_ISL_8791265, EPI_ISL_8791266, EPI_ISL_8791267, EPI_ISL_8791270			
see above	UniversitätsSpital Zürich	Institute of Medical Virology, University of Zurich	Alexandra Trkola; Annette Audigé; Catharine Aquino; Cyril Shah; Daniel Ehrsam; Gabriela Ziltener; Guido Bloemberg; Hubert Rehrauer; Isabel Stürmer; Joel Wirz; Jon Huder; Jürg Böni; Kevin Steiner; Maria Grünberg; Maryam Zaheri; Michael Huber; Riccarda Capaul; Stefan Schmutz; Verena Kufner; Weihong Qi
EPI_ISL_8788617, EPI_ISL_8788640	Viollier AG	Department of Biosystems Science and Engineering, ETH Zürich	Andrea Patrignani; Andrea Patrizia Salzmann; Andreia Cabral de Gouvea; Catharine Aquino Fournier; Chaoran Chen; Christian Urban; Christiane Beckmann; Christoph Noppen; Daniel Ehrsam; David Dreifuss; Doris Popovic; Franziska Singer; Griffin White; Hai Bui; Henriette Kurth; Isabel Stürmer; Ivan Topolsky; Jay Tracy; Kim Philipp Jablonski; Lara Fuhrmann; Laura Neff; Lennart Opitz; Louis du Plessis; Maria Domenica Moccia; Matteo Carrara; Maurice Redondo; Niko Beerenwinkel; Olivier Kobel; Pelin Icer; Ralph Schlapbach; Sarah Nadeau; Shuqing Yu; Simon Grüter; Tanja Stadler; Timothy Sykes
EPI_ISL_8791540	Virology Laboratory, Scientific Department, Army Medical Center	Virology Laboratory, Scientific Department, Army Medical Center	Anella Monte; Anna Anselmo; Antonella Fortunato; Filippo Molinari; Florigio Lista; Francesco Giordani; Giancarlo Petralito; Giandomenico Cerreto; Giulia Campoli; Lucia Nicosia; Marzia Cavalli; Pietro Marco D'Angelo; Riccardo De Sanctis; Rossella Brandi; Silvia Fillo; Vanessa Vera Fain

We gratefully acknowledge the following Authors from the Originating laboratories responsible for obtaining the specimens, as well as the Submitting laboratories where the genome data were generated and shared via GISAID, on which this research is based.

All Submitters of data may be contacted directly via www.gisaid.org

Authors are sorted alphabetically.

Accession ID	Originating Laboratory	Submitting Laboratory	Authors
EPI_ISL_8799962	Cell-T Sp. z o.o.	1. Tricity SARS-CoV-2 sequencing consortium: University of Gdansk, Medical University of Gdansk, Vaxican Ltd., Invicta Ltd. 2. National Institute of Public Health - National Institute of Hygiene, Warsaw, Poland	Celina Cybulska; Karolina Gackowska; Katarzyna Groth; Katarzyna Zacharczuk; Krystyna Bienkowska Szewczyk; Lukasz Rabalski; Maciej Grzybek; Maciej Kosinski; Magdalena Nowakowska; Marcin Lubocki; Małgorzata Sadkowska-Todys; Tomasz Wołkowicz
EPI_ISL_8795428, EPI_ISL_8795499, EPI_ISL_8795673, EPI_ISL_8795704	Colorado Department of Public Health and Environment	Colorado Department of Public Health and Environment	Alexandria Rossheim; Arianna Smith; Diana Ir; Emily A. Travanty; Laura Bankers; Mandy Waters; Michael Martin; Molly C. Hetherington-Rauth; Shannon R. Matzinger
EPI_ISL_8803947, EPI_ISL_8804058, EPI_ISL_8804066, EPI_ISL_8804072, EPI_ISL_8804078, EPI_ISL_8804080, EPI_ISL_8804089, EPI_ISL_8804091, EPI_ISL_8804097, EPI_ISL_8804098, EPI_ISL_8804107, EPI_ISL_8804109, EPI_ISL_8804111, EPI_ISL_8804118, EPI_ISL_8804119, EPI_ISL_8804120, EPI_ISL_8804130, EPI_ISL_8804137, EPI_ISL_8804141, EPI_ISL_8804147, EPI_ISL_8804159, EPI_ISL_8804162, EPI_ISL_8804170, EPI_ISL_8804176, EPI_ISL_8804179, EPI_ISL_8804181, EPI_ISL_8804183, EPI_ISL_8804184, EPI_ISL_8804212, EPI_ISL_8804214, EPI_ISL_8804220, EPI_ISL_8804238, EPI_ISL_8804242, EPI_ISL_8804244, EPI_ISL_8804265, EPI_ISL_8804268, EPI_ISL_8804272, EPI_ISL_8804280, EPI_ISL_8804286, EPI_ISL_8804288, EPI_ISL_8804290, EPI_ISL_8804297, EPI_ISL_8804299, EPI_ISL_8804303, EPI_ISL_8804305, EPI_ISL_8804317, EPI_ISL_8804325, EPI_ISL_8804330, EPI_ISL_8804332, EPI_ISL_8804351, EPI_ISL_8804361, EPI_ISL_8804363, EPI_ISL_8804371, EPI_ISL_8804373, EPI_ISL_8804383, EPI_ISL_8804392, EPI_ISL_8804408, EPI_ISL_8804412, EPI_ISL_8804434, EPI_ISL_8804530, EPI_ISL_8804537, EPI_ISL_8804606, EPI_ISL_8804608, EPI_ISL_8804613, EPI_ISL_8804615, EPI_ISL_8804616, EPI_ISL_8804621, EPI_ISL_8804629, EPI_ISL_8804640, EPI_ISL_8804650, EPI_ISL_8804655, EPI_ISL_8804658, EPI_ISL_8804662, EPI_ISL_8804665, EPI_ISL_8804675, EPI_ISL_8804677, EPI_ISL_8804679, EPI_ISL_8804685, EPI_ISL_8804703, EPI_ISL_8804707, EPI_ISL_8804709, EPI_ISL_8804715, EPI_ISL_8804718, EPI_ISL_8804722, EPI_ISL_8804728, EPI_ISL_8804746, EPI_ISL_8804754, EPI_ISL_8804841, EPI_ISL_8804855, EPI_ISL_8804857, EPI_ISL_8804861, EPI_ISL_8804866, EPI_ISL_8804885, EPI_ISL_8804897, EPI_ISL_8804902, EPI_ISL_8804910, EPI_ISL_8804917, EPI_ISL_8804921, EPI_ISL_8804937, EPI_ISL_8804944, EPI_ISL_8804950, EPI_ISL_8804952, EPI_ISL_8804954, EPI_ISL_8804958, EPI_ISL_8804962, EPI_ISL_8804970, EPI_ISL_8804972, EPI_ISL_8804982, EPI_ISL_8804990, EPI_ISL_8804998, EPI_ISL_8805003, EPI_ISL_8805006, EPI_ISL_8805022, EPI_ISL_8805025, EPI_ISL_8805034, EPI_ISL_8805038, EPI_ISL_8805051, EPI_ISL_8805065, EPI_ISL_8805066, EPI_ISL_8805070, EPI_ISL_8805080, EPI_ISL_8805082, EPI_ISL_8805087, EPI_ISL_8805089, EPI_ISL_8805093, EPI_ISL_8805096, EPI_ISL_8805105, EPI_ISL_8805110, EPI_ISL_8805125, EPI_ISL_8805131, EPI_ISL_8805137, EPI_ISL_8805141, EPI_ISL_8805143, EPI_ISL_8805147, EPI_ISL_8805149, EPI_ISL_8805153, EPI_ISL_8805154, EPI_ISL_8805160, EPI_ISL_8805166, EPI_ISL_8805170, EPI_ISL_8805173, EPI_ISL_8805181, EPI_ISL_8805186, EPI_ISL_8805190, EPI_ISL_8805224, EPI_ISL_8805230, EPI_ISL_8805239, EPI_ISL_8805245, EPI_ISL_8805265, EPI_ISL_8805280, EPI_ISL_8805284, EPI_ISL_8805355, EPI_ISL_8805365, EPI_ISL_8805367, EPI_ISL_8805372, EPI_ISL_8805374, EPI_ISL_8805379, EPI_ISL_8805381, EPI_ISL_8805387, EPI_ISL_8805391, EPI_ISL_8805409, EPI_ISL_8805411, EPI_ISL_8805413, EPI_ISL_8805422, EPI_ISL_8805423, EPI_ISL_8805425, EPI_ISL_8805429, EPI_ISL_8805431, EPI_ISL_8805433, EPI_ISL_8805442, EPI_ISL_8805459, EPI_ISL_8805466, EPI_ISL_8805470, EPI_ISL_8805480, EPI_ISL_8805484, EPI_ISL_8805489, EPI_ISL_8805504, EPI_ISL_8805519, EPI_ISL_8805521, EPI_ISL_8805524, EPI_ISL_8805528, EPI_ISL_8805546, EPI_ISL_8805548, EPI_ISL_8805551, EPI_ISL_8805578, EPI_ISL_8805579, EPI_ISL_8805588, EPI_ISL_8805594, EPI_ISL_8805604, EPI_ISL_8805619, EPI_ISL_8805638, EPI_ISL_8805735, EPI_ISL_8805736, EPI_ISL_8805738, EPI_ISL_8805742, EPI_ISL_8805744, EPI_ISL_8805763, EPI_ISL_8805775, EPI_ISL_8805782, EPI_ISL_8805787, EPI_ISL_8805801, EPI_ISL_8805816, EPI_ISL_8805818, EPI_ISL_8805822, EPI_ISL_8805826, EPI_ISL_8805830, EPI_ISL_8805832, EPI_ISL_8805836, EPI_ISL_8805838, EPI_ISL_8805839, EPI_ISL_8805863, EPI_ISL_8805869, EPI_ISL_8805887, EPI_ISL_8805896, EPI_ISL_8805898, EPI_ISL_8805906, EPI_ISL_8805908, EPI_ISL_8805911, EPI_ISL_8805916, EPI_ISL_8805926, EPI_ISL_8805929, EPI_ISL_8805931, EPI_ISL_8805938, EPI_ISL_8805939, EPI_ISL_8805941, EPI_ISL_8805944, EPI_ISL_8805948, EPI_ISL_8805949, EPI_ISL_8805960, EPI_ISL_8805962, EPI_ISL_8805971, EPI_ISL_8805972, EPI_ISL_8805977, EPI_ISL_8805978, EPI_ISL_8805979, EPI_ISL_8805982, EPI_ISL_8806033, EPI_ISL_8806036, EPI_ISL_8806046, EPI_ISL_8806047, EPI_ISL_8806048, EPI_ISL_8806053, EPI_ISL_8806057, EPI_ISL_8806064, EPI_ISL_8806066, EPI_ISL_8806067, EPI_ISL_8806091, EPI_ISL_8806099, EPI_ISL_8806107, EPI_ISL_8806116, EPI_ISL_8806119, EPI_ISL_8806129, EPI_ISL_8806132, EPI_ISL_8806138, EPI_ISL_8806140, EPI_ISL_8806141, EPI_ISL_8806143, EPI_ISL_8806147, EPI_ISL_8806148, EPI_ISL_8806151, EPI_ISL_8806155, EPI_ISL_8806156, EPI_ISL_8806161, EPI_ISL_8806163, EPI_ISL_8806175, EPI_ISL_8806177, EPI_ISL_8806183, EPI_ISL_8806219, EPI_ISL_8806220, EPI_ISL_8806228, EPI_ISL_8806230, EPI_ISL_8806233, EPI_ISL_8806234, EPI_ISL_8806235, EPI_ISL_8806239, EPI_ISL_8806240, EPI_ISL_8806241, EPI_ISL_8806244, EPI_ISL_8806245, EPI_ISL_8806285, EPI_ISL_8806288, EPI_ISL_8806290, EPI_ISL_8806299, EPI_ISL_8806300, EPI_ISL_8806306, EPI_ISL_8806307, EPI_ISL_8806310, EPI_ISL_8806311, EPI_ISL_8806316, EPI_ISL_8806320, EPI_ISL_8806324, EPI_ISL_8806327, EPI_ISL_8806330, EPI_ISL_8806331, EPI_ISL_8806332, EPI_ISL_8806334, EPI_ISL_8806342, EPI_ISL_8806344, EPI_ISL_8806346, EPI_ISL_8806347, EPI_ISL_8806353, EPI_ISL_8806358, EPI_ISL_8806363, EPI_ISL_8806365, EPI_ISL_8806375, EPI_ISL_8806377, EPI_ISL_8806382, EPI_ISL_8806390, EPI_ISL_8806391, EPI_ISL_8806394, EPI_ISL_8806396, EPI_ISL_8806398, EPI_ISL_8806400, EPI_ISL_8806401, EPI_ISL_8806406, EPI_ISL_8806408, EPI_ISL_8806409, EPI_ISL_8806413, EPI_ISL_8806414, EPI_ISL_8806520, EPI_ISL_8806522, EPI_ISL_8806524, EPI_ISL_8806529, EPI_ISL_8806534, EPI_ISL_8806536, EPI_ISL_8806542, EPI_ISL_8806545, EPI_ISL_8806549, EPI_ISL_8806555, EPI_ISL_8806574, EPI_ISL_8806576, EPI_ISL_8806592, EPI_ISL_8806600, EPI_ISL_8806617, EPI_ISL_8806621, EPI_ISL_8806627, EPI_ISL_8806636, EPI_ISL_8806639, EPI_ISL_8806684, EPI_ISL_8806689, EPI_ISL_8806693, EPI_ISL_8806697, EPI_ISL_8807698, EPI_ISL_8807700, EPI_ISL_8807702, EPI_ISL_8807703, EPI_ISL_8807710, EPI_ISL_8807711, EPI_ISL_8807714, EPI_ISL_8807715, EPI_ISL_8807720, EPI_ISL_8807721, EPI_ISL_8807722, EPI_ISL_8807723, EPI_ISL_8807728, EPI_ISL_8807736			
see above	Department of Bacteria, Parasites and Fungi, Statens Serum Institut, Copenhagen, Denmark	Statens Serum Institut Bioinformatics and Microbial Genomics	Danish Covid-19 Genome Consortium
EPI_ISL_8804062, EPI_ISL_8804122, EPI_ISL_8804129, EPI_ISL_8804134, EPI_ISL_8804146, EPI_ISL_8804198, EPI_ISL_8804228, EPI_ISL_8804234, EPI_ISL_8804240, EPI_ISL_8804256, EPI_ISL_8804274, EPI_ISL_8804283, EPI_ISL_8804294, EPI_ISL_8804296, EPI_ISL_8804321, EPI_ISL_8804334, EPI_ISL_8804357, EPI_ISL_8804367, EPI_ISL_8804531, EPI_ISL_8804544, EPI_ISL_8804610, EPI_ISL_8804646, EPI_ISL_8804649, EPI_ISL_8804681, EPI_ISL_8804683, EPI_ISL_8804687, EPI_ISL_8804694, EPI_ISL_8804726, EPI_ISL_8804756, EPI_ISL_8804764, EPI_ISL_8804865, EPI_ISL_8804872, EPI_ISL_8804893, EPI_ISL_8804974, EPI_ISL_8805058, EPI_ISL_8805121, EPI_ISL_8805127, EPI_ISL_8805232, EPI_ISL_8805247, EPI_ISL_8805258, EPI_ISL_8805282, EPI_ISL_8805351, EPI_ISL_8805386, EPI_ISL_8805397, EPI_ISL_8805443, EPI_ISL_8805455, EPI_ISL_8805496, EPI_ISL_8805498, EPI_ISL_8805536, EPI_ISL_8805582, EPI_ISL_8805592, EPI_ISL_8805608, EPI_ISL_8805612, EPI_ISL_8805627, EPI_ISL_8805631, EPI_ISL_8805753, EPI_ISL_8805773, EPI_ISL_8805795, EPI_ISL_8805820, EPI_ISL_8805851, EPI_ISL_8805892, EPI_ISL_8805915, EPI_ISL_8805925, EPI_ISL_8805959, EPI_ISL_8805981, EPI_ISL_8806037, EPI_ISL_8806041, EPI_ISL_8806045, EPI_ISL_8806056, EPI_ISL_8806059, EPI_ISL_8806062, EPI_ISL_8806087, EPI_ISL_8806088, EPI_ISL_8806104, EPI_ISL_8806123, EPI_ISL_8806157, EPI_ISL_8806174, EPI_ISL_8806218, EPI_ISL_8806222, EPI_ISL_8806229, EPI_ISL_8806232, EPI_ISL_8806284, EPI_ISL_8806388, EPI_ISL_8806525, EPI_ISL_8806597			
see above	Department of Clinical Microbiology and Center for Genomic Medicine, Rigshospitalet, Copenhagen, Denmark	Statens Serum Institut Bioinformatics and Microbial Genomics	Danish Covid-19 Genome Consortium
EPI_ISL_8800370	Department of Public Health Iasi	National Institute of Infectious Diseases-Prof. Dr. Matei Bals Molecular Diagnostics Laboratory	Corina Casangiu; Dan Otelea; Leontina Banica; Marius Surleac; Ovidiu Vlaicu; Petre Milu; Robert Hohan; Simona Paraschiv
EPI_ISL_8800328	Department of Public Health Mures	National Institute of Infectious Diseases-Prof. Dr. Matei Bals Molecular Diagnostics Laboratory	Corina Casangiu; Dan Otelea; Leontina Banica; Marius Surleac; Ovidiu Vlaicu; Petre Milu; Robert Hohan; Simona Paraschiv
EPI_ISL_8801461, EPI_ISL_8801462, EPI_ISL_8801463, EPI_ISL_8801466, EPI_ISL_8801467	KKP Kelas 1 Soekarno-Hatta (FarmaLab)	National Institute of Health Research and Development	Arie Ardiansyah Nugraha; Fajar Nur Sulistiyohadi; Hana Apsari Pawestri; Hartanti Dian Ikawati; Kartika Dewi Puspa; Nelly Puspandari; Nur Ika Hariastuti; Putri Widia; Subangkit; Vivi Setiawaty
EPI_ISL_8799194	LEBOWAKGOMO LABORATORY	National Institute for Communicable Diseases of the National Health Laboratory Service	Amoako DG; Bhiman JN; Everatt J; Ismail A; Kekana D; Mahlangu B; Mnguni A; Mohale T; Ntuli N; Scheepers C; Wolter N
EPI_ISL_8799964	Laboratoria Medyczne Bruss grupa ALAB sp. z o.o.; Medyczne Laboratorium Diagnostyczne, Pracownia Genetyki	1. Tricity SARS-CoV-2 sequencing consortium: University of Gdansk, Medical University of Gdansk, Vaxican Ltd., Invicta Ltd. 2. National Institute of Public Health - National Institute of Hygiene, Warsaw, Poland	Celina Cybulska; Karolina Gackowska; Katarzyna Groth; Katarzyna Zacharczuk; Krystyna Bienkowska Szewczyk; Lukasz Rabalski; Maciej Grzybek; Maciej Kosinski; Magdalena Nowakowska; Marcin Lubocki; Małgorzata Sadkowska-Todys; Tomasz Wołkowicz
EPI_ISL_8800203	National Institute of Infectious Diseases-Prof. Dr. Matei Bals Molecular Diagnostics Laboratory	National Institute of Infectious Diseases-Prof. Dr. Matei Bals Molecular Diagnostics Laboratory	Corina Casangiu; Dan Otelea; Leontina Banica; Marius Surleac; Ovidiu Vlaicu; Petre Milu; Robert Hohan; Simona Paraschiv
EPI_ISL_8806579	Respiratory Virus Unit, Microbiology Services Colindale, Public Health England	COVID-19 Genomics UK (COG-UK) Consortium	PHE Covid Sequencing Team
EPI_ISL_8796869	SK-Roy Romanow Provincial Laboratory	Saskatchewan - Roy Romanow Provincial Laboratory (RRPL)	Alanna Senecal; Amanda Lang; Jessica Minion; Kara Loos; Keith MacKenzie; Meredith Faires; Rachel DePaulo; Roy Romanow Provincial Laboratory - Molecular Diagnostics; Ryan McDonald
EPI_ISL_8807361	UniversitätsSpital Zürich	Institute of Medical Virology, University of Zurich	Alexandra Trkola; Annette Audigé; Cyril Shah; Gabriela Ziltener; Guido Bloemberg; Jon Huder; Jürg Böni; Kevin Steiner; Maria Grünberg; Maryam Zaheri; Michael Huber; Riccarda Capaul; Stefan Schmutz; Verena Kufner
EPI_ISL_8801350, EPI_ISL_8801351, EPI_ISL_8801352, EPI_ISL_8801353, EPI_ISL_8801354, EPI_ISL_8801355, EPI_ISL_8801356, EPI_ISL_8801357, EPI_ISL_8801358, EPI_ISL_8801359, EPI_ISL_8801360, EPI_ISL_8801361, EPI_ISL_8801365, EPI_ISL_8801366, EPI_ISL_8801367, EPI_ISL_8801368, EPI_ISL_8801369, EPI_ISL_8801370, EPI_ISL_8801371, EPI_ISL_8801372, EPI_ISL_8801374, EPI_ISL_8801375, EPI_ISL_8801377, EPI_ISL_8801378, EPI_ISL_8801380			
see above	VRDL,GMC,PTA	NIV Influenza	Dr.Rupinder Bakshi

We gratefully acknowledge the following Authors from the Originating laboratories responsible for obtaining the specimens, as well as the Submitting laboratories where the genome data were generated and shared via GISAID, on which this research is based.

All Submitters of data may be contacted directly via www.gisaid.org

Authors are sorted alphabetically.

Accession ID	Originating Laboratory	Submitting Laboratory	Authors
EPI_ISL_8814315, EPI_ISL_8814384, EPI_ISL_8815349	Aegis Sciences Corporation	Centers for Disease Control and Prevention Division of Viral Diseases, Pathogen Discovery	Alec Vest; Benjamin Rambo-Martin; Christopher Gulvick; Clinton Paden; Cyndi Clark; Dakota Howard; Dhwani Batra; Dillon Nall; Duncan MacCannell; Erisa Sula; Ethan Sanders; Holly Houdeshell; Jason Caravas; Kristine Lacek; Matthew Hardison; Matthew Schmerer; Ola Kvalvaag; Patrick Campbell; Peter Cook; Rob Case; Scott Sammons; Shatavia Morrison; Shaun Westlund; Tymeckia Kendall; Victoria Caban Figueroa; Vikramsinha Ghorpade; Yvette Unoarumhi
EPI_ISL_8821241	CDPH VBL	California Department of Public Health	CDPH COVIDNet
EPI_ISL_8807737, EPI_ISL_8807742, EPI_ISL_8807747, EPI_ISL_8807749, EPI_ISL_8807751, EPI_ISL_8807752, EPI_ISL_8807753, EPI_ISL_8807754, EPI_ISL_8807755, EPI_ISL_8807756, EPI_ISL_8807765, EPI_ISL_8807767, EPI_ISL_8807769, EPI_ISL_8807780, EPI_ISL_8807788, EPI_ISL_8807792, EPI_ISL_8807795, EPI_ISL_8807798, EPI_ISL_8807802, EPI_ISL_8807805, EPI_ISL_8807809, EPI_ISL_8807814, EPI_ISL_8807818, EPI_ISL_8807822, EPI_ISL_8807919, EPI_ISL_8807926, EPI_ISL_8807931, EPI_ISL_8807933, EPI_ISL_8807935, EPI_ISL_8807937, EPI_ISL_8807941, EPI_ISL_8807946			
see above	Department of Bacteria, Parasites and Fungi, Statens Serum Institut, Copenhagen, Denmark	Statens Serum Institut Bioinformatics and Microbial Genomics	Danish Covid-19 Genome Consortium
EPI_ISL_8807741, EPI_ISL_8807775, EPI_ISL_8807793, EPI_ISL_8807819, EPI_ISL_8807832, EPI_ISL_8807921, EPI_ISL_8807924			
see above	Department of Clinical Microbiology and Center for Genomic Medicine, Rigshospitalet, Copenhagen, Denmark	Statens Serum Institut Bioinformatics and Microbial Genomics	Danish Covid-19 Genome Consortium
EPI_ISL_8809026	Florida Bureau of Public Health Laboratories	Florida Bureau of Public Health Laboratories	Jason Blanton; Namratha Tarigopula; Sarah Schmedes; Tiffany Splatt
EPI_ISL_8808115, EPI_ISL_8808118	Jessa	Jessa	Severine Berden et al. on behalf of the Jessa_cmdLab
EPI_ISL_8809739, EPI_ISL_8809757	Public Health Laboratory, Minnesota Department of Health	University of Minnesota Genomics Center	Corbin Dirkx; Daryl M. Gohl; Jaquelyn Kuriger-Laber; John Garbe
EPI_ISL_8824283	Tripler Army Medical Center	Tripler Army Medical Center	Jonathan Andrew D'Ambrozio
EPI_ISL_8818108	Wyoming Public Health Laboratory	Wyoming Public Health Laboratory	Ashley Norberg; Brian Dominguez; Cameron Ward; Carina Williams; Channing Weber; Chayse Rowley; Cyrus Rautman; Daniel Bolding; Elliot Thomasson; Emma Snow; Jim Mildenberger; Noah Cheshier; Rob Christensen; Robert Petit; Taylor Fearing; and Joseph Reed

We gratefully acknowledge the following Authors from the Originating laboratories responsible for obtaining the specimens, as well as the Submitting laboratories where the genome data were generated and shared via GISAID, on which this research is based.

All Submitters of data may be contacted directly via www.gisaid.org

Authors are sorted alphabetically.

Acknowledgement EPI_SET Identifier: EPI_SET_20220527nb

Accession ID	Originating Laboratory	Submitting Laboratory	Authors
EPI_ISL_8827119, EPI_ISL_8827901, EPI_ISL_8828165, EPI_ISL_8828166, EPI_ISL_8830893, EPI_ISL_8831308	Aegis Sciences Corporation	Centers for Disease Control and Prevention Division of Viral Diseases, Pathogen Discovery	Alec Vest; Benjamin Rambo-Martin; Christopher Gulvick; Clinton Paden; Cyndi Clark; Dakota Howard; Dhwani Batra; Dillon Nall; Duncan MacCannell; Erisa Sula; Ethan Sanders; Holly Houdeshell; Jason Caravas; Kristine Lacek; Matthew Hardison; Matthew Schmerer; Ola Kvalvaag; Patrick Campbell; Peter Cook; Rob Case; Scott Sammons; Shatavia Morrison; Shaun Westlund; Tymeckia Kendall; Victoria Caban Figueroa; Vikramsinha Ghorpade; Yvette Unoarumhi
EPI_ISL_8826519	Botswana Harvard HIV Reference Laboratory	Botswana Harvard HIV Reference Laboratory	Boitumelo J. L Zuze; Botshelo Radibe; Dorcas Maruapula; Joseph Makhema; Keoratile Ntshambiwa; Kgomotso Moruisi; Legodile Kooepile; Letsibogo Gaoraelwe; Mosepele Mosepele; Mphaphi B. Mbulawa; Ontlametse T. Bareng; Pamela Smith-Lawrence; Roger Shapiro; Sefetogi Ramaologa; Shahin Lockman; Sikhulile Moyo; Simani Gaseitsiwe; Thela Tefelo; Thongbotho Mphoyakgosi; Wonderful T. Choga
EPI_ISL_8831657, EPI_ISL_8831659, EPI_ISL_8831669, EPI_ISL_8831671, EPI_ISL_8831686, EPI_ISL_8831691, EPI_ISL_8831695, EPI_ISL_8831697, EPI_ISL_8831703, EPI_ISL_8831705, EPI_ISL_8831721, EPI_ISL_8831735, EPI_ISL_8831736, EPI_ISL_8831738, EPI_ISL_8831743, EPI_ISL_8831750, EPI_ISL_8831758, EPI_ISL_8831764, EPI_ISL_8831769, EPI_ISL_8831774, EPI_ISL_8831782, EPI_ISL_8831789, EPI_ISL_8831801, EPI_ISL_8831807, EPI_ISL_8831811, EPI_ISL_8831816, EPI_ISL_8831833, EPI_ISL_8831836, EPI_ISL_8831847, EPI_ISL_8831849, EPI_ISL_8831856, EPI_ISL_8831857, EPI_ISL_8831863, EPI_ISL_8831870, EPI_ISL_8831871, EPI_ISL_8831876, EPI_ISL_8831880, EPI_ISL_8831893, EPI_ISL_8831908, EPI_ISL_8831910, EPI_ISL_8831916, EPI_ISL_8831926, EPI_ISL_8831929, EPI_ISL_8831932, EPI_ISL_8831935, EPI_ISL_8831936, EPI_ISL_8831938, EPI_ISL_8831944, EPI_ISL_8831947, EPI_ISL_8831949, EPI_ISL_8831962, EPI_ISL_8831966, EPI_ISL_8831975, EPI_ISL_8831984, EPI_ISL_8831985, EPI_ISL_8831989, EPI_ISL_8831994, EPI_ISL_8831996, EPI_ISL_8832011, EPI_ISL_8832012, EPI_ISL_8832021, EPI_ISL_8832024, EPI_ISL_8832027, EPI_ISL_8832029, EPI_ISL_8832035, EPI_ISL_8832037, EPI_ISL_8832038, EPI_ISL_8832040, EPI_ISL_8832054, EPI_ISL_8832060, EPI_ISL_8832071, EPI_ISL_8832077, EPI_ISL_8832089, EPI_ISL_8832092, EPI_ISL_8832099, EPI_ISL_8832111, EPI_ISL_8832118, EPI_ISL_8832143, EPI_ISL_8832148, EPI_ISL_8832153, EPI_ISL_8832161, EPI_ISL_8832165, EPI_ISL_8832169, EPI_ISL_8832182, EPI_ISL_8832191, EPI_ISL_8832193, EPI_ISL_8832198, EPI_ISL_8832210, EPI_ISL_8832215, EPI_ISL_8832226, EPI_ISL_8832235, EPI_ISL_8832239, EPI_ISL_8832241, EPI_ISL_8832249, EPI_ISL_8832259, EPI_ISL_8832265, EPI_ISL_8832269, EPI_ISL_8832275, EPI_ISL_8832276, EPI_ISL_8832285, EPI_ISL_8832298, EPI_ISL_8832313, EPI_ISL_8832315, EPI_ISL_8832354, EPI_ISL_8832356, EPI_ISL_8832358, EPI_ISL_8832359, EPI_ISL_8832361, EPI_ISL_8832365, EPI_ISL_8832379, EPI_ISL_8832381, EPI_ISL_8832385, EPI_ISL_8832387, EPI_ISL_8832389, EPI_ISL_8832398, EPI_ISL_8832415, EPI_ISL_8832419, EPI_ISL_8832421, EPI_ISL_8832423, EPI_ISL_8832429, EPI_ISL_8832432, EPI_ISL_8832434, EPI_ISL_8832444, EPI_ISL_8832451, EPI_ISL_8832452, EPI_ISL_8832453, EPI_ISL_8832459, EPI_ISL_8832473, EPI_ISL_8832480, EPI_ISL_8832487, EPI_ISL_8832488, EPI_ISL_8832492, EPI_ISL_8832504, EPI_ISL_8832510, EPI_ISL_8832517, EPI_ISL_8832525, EPI_ISL_8832528, EPI_ISL_8832539, EPI_ISL_8832541, EPI_ISL_8832545, EPI_ISL_8832551, EPI_ISL_8832552, EPI_ISL_8832554, EPI_ISL_8832557, EPI_ISL_8832566, EPI_ISL_8832568, EPI_ISL_8832576, EPI_ISL_8832579, EPI_ISL_8832581, EPI_ISL_8832583, EPI_ISL_8832587, EPI_ISL_8832599, EPI_ISL_8832605, EPI_ISL_8832607, EPI_ISL_8832618, EPI_ISL_8832619, EPI_ISL_8832621, EPI_ISL_8832632, EPI_ISL_8832638, EPI_ISL_8832640, EPI_ISL_8832651, EPI_ISL_8832661, EPI_ISL_8832665, EPI_ISL_8832666, EPI_ISL_8832676, EPI_ISL_8832681, EPI_ISL_8832682, EPI_ISL_8832685, EPI_ISL_8832686, EPI_ISL_8832700, EPI_ISL_8832708, EPI_ISL_8832710, EPI_ISL_8832716, EPI_ISL_8832720, EPI_ISL_8832722, EPI_ISL_8832724, EPI_ISL_8832730, EPI_ISL_8832731, EPI_ISL_8832735, EPI_ISL_8832741, EPI_ISL_8832750, EPI_ISL_8832754, EPI_ISL_8832756, EPI_ISL_8832758, EPI_ISL_8832766, EPI_ISL_8832768, EPI_ISL_8832769, EPI_ISL_8832771, EPI_ISL_8832775, EPI_ISL_8832781, EPI_ISL_8832783, EPI_ISL_8832791, EPI_ISL_8832793, EPI_ISL_8832795, EPI_ISL_8832797, EPI_ISL_8832800, EPI_ISL_8832802, EPI_ISL_8832808, EPI_ISL_8832810, EPI_ISL_8832811, EPI_ISL_8832826, EPI_ISL_8832846, EPI_ISL_8832851, EPI_ISL_8832854, EPI_ISL_8832864, EPI_ISL_8832871, EPI_ISL_8832875, EPI_ISL_8832877, EPI_ISL_8832881, EPI_ISL_8832886, EPI_ISL_8832898, EPI_ISL_8832903, EPI_ISL_8832909, EPI_ISL_8832927, EPI_ISL_8832940, EPI_ISL_8832944, EPI_ISL_8832950, EPI_ISL_8832957, EPI_ISL_8832961, EPI_ISL_8832975, EPI_ISL_8832981, EPI_ISL_8832995, EPI_ISL_8833015, EPI_ISL_8833017, EPI_ISL_8833021, EPI_ISL_8833022, EPI_ISL_8833030, EPI_ISL_8833031, EPI_ISL_8833044, EPI_ISL_8833053, EPI_ISL_8833055, EPI_ISL_8833067, EPI_ISL_8833069, EPI_ISL_8833077, EPI_ISL_8833095, EPI_ISL_8833101, EPI_ISL_8833109, EPI_ISL_8833130, EPI_ISL_8833132, EPI_ISL_8833135, EPI_ISL_8833142, EPI_ISL_8833150, EPI_ISL_8833154, EPI_ISL_8833156, EPI_ISL_8833164, EPI_ISL_8833180, EPI_ISL_8833192, EPI_ISL_8833198, EPI_ISL_8833204, EPI_ISL_8833212, EPI_ISL_8833214, EPI_ISL_8833217, EPI_ISL_8833218, EPI_ISL_8833221, EPI_ISL_8833222, EPI_ISL_8833237, EPI_ISL_8833326, EPI_ISL_8833335, EPI_ISL_8833342, EPI_ISL_8833346, EPI_ISL_8833357, EPI_ISL_8833366, EPI_ISL_8833368, EPI_ISL_8833375, EPI_ISL_8833379, EPI_ISL_8833382, EPI_ISL_8833384, EPI_ISL_8833385, EPI_ISL_8833387, EPI_ISL_8833393, EPI_ISL_8833395, EPI_ISL_8833415, EPI_ISL_8833421, EPI_ISL_8833424, EPI_ISL_8833441, EPI_ISL_8833445, EPI_ISL_8833451, EPI_ISL_8833458, EPI_ISL_8833461, EPI_ISL_8833471, EPI_ISL_8833482, EPI_ISL_8833488, EPI_ISL_8833489, EPI_ISL_8833491, EPI_ISL_8833519, EPI_ISL_8833522, EPI_ISL_8833531, EPI_ISL_8833534, EPI_ISL_8833538, EPI_ISL_8833546, EPI_ISL_8833551, EPI_ISL_8833558, EPI_ISL_8833566, EPI_ISL_8833568, EPI_ISL_8833571, EPI_ISL_8833574, EPI_ISL_8833582, EPI_ISL_8833583, EPI_ISL_8833594, EPI_ISL_8833602, EPI_ISL_8833605, EPI_ISL_8833607, EPI_ISL_8833613, EPI_ISL_8833615, EPI_ISL_8833618, EPI_ISL_8833619, EPI_ISL_8833623, EPI_ISL_8833625, EPI_ISL_8833645, EPI_ISL_8833650, EPI_ISL_8833664, EPI_ISL_8833675, EPI_ISL_8833679, EPI_ISL_8833680, EPI_ISL_8833681, EPI_ISL_8833684, EPI_ISL_8833693, EPI_ISL_8833701, EPI_ISL_8833705, EPI_ISL_8833730, EPI_ISL_8833737, EPI_ISL_8833758, EPI_ISL_8833760, EPI_ISL_8833765, EPI_ISL_8833766, EPI_ISL_8833770, EPI_ISL_8833772, EPI_ISL_8833774, EPI_ISL_8833779, EPI_ISL_8833784, EPI_ISL_8833814, EPI_ISL_8833819, EPI_ISL_8833821, EPI_ISL_8833823, EPI_ISL_8833840, EPI_ISL_8833846, EPI_ISL_8833848, EPI_ISL_8833852, EPI_ISL_8833857, EPI_ISL_8833858, EPI_ISL_8833859, EPI_ISL_8833865, EPI_ISL_8833892, EPI_ISL_8833894, EPI_ISL_8833898, EPI_ISL_8833900, EPI_ISL_8833913, EPI_ISL_8833917, EPI_ISL_8833924, EPI_ISL_8833937, EPI_ISL_8833941, EPI_ISL_8833949, EPI_ISL_8833951, EPI_ISL_8833954, EPI_ISL_8833978, EPI_ISL_8833981, EPI_ISL_8833983, EPI_ISL_8833988, EPI_ISL_8833989, EPI_ISL_8833992, EPI_ISL_8833999, EPI_ISL_8834001, EPI_ISL_8834002, EPI_ISL_8834003, EPI_ISL_8834005, EPI_ISL_8834017, EPI_ISL_8834019, EPI_ISL_8834022, EPI_ISL_8834033, EPI_ISL_8834039, EPI_ISL_8834042, EPI_ISL_8834049, EPI_ISL_8834058, EPI_ISL_8834065, EPI_ISL_8834070, EPI_ISL_8834080, EPI_ISL_8834081, EPI_ISL_8834083, EPI_ISL_8834084, EPI_ISL_8834101, EPI_ISL_8834102, EPI_ISL_8834125, EPI_ISL_8834156, EPI_ISL_8834158, EPI_ISL_8834170, EPI_ISL_8834174, EPI_ISL_8834179, EPI_ISL_8834185, EPI_ISL_8834196, EPI_ISL_8834201, EPI_ISL_8834206, EPI_ISL_8834228, EPI_ISL_8834239, EPI_ISL_8834241, EPI_ISL_8834243, EPI_ISL_8834245, EPI_ISL_8834247, EPI_ISL_8834248, EPI_ISL_8834250, EPI_ISL_8834262, EPI_ISL_8834269, EPI_ISL_8834273, EPI_ISL_8834277, EPI_ISL_8834280, EPI_ISL_8834281, EPI_ISL_8834283, EPI_ISL_8834292, EPI_ISL_8834293, EPI_ISL_8834297, EPI_ISL_8834299, EPI_ISL_8834302, EPI_ISL_8834306, EPI_ISL_8834314, EPI_ISL_8834321, EPI_ISL_8834323, EPI_ISL_8834327, EPI_ISL_8834335, EPI_ISL_8834339			
see above	Department of Bacteria, Parasites and Fungi, Statens Serum Institut, Copenhagen, Denmark	Statens Serum Institut Bioinformatics and Microbial Genomics	Danish Covid-19 Genome Consortium
EPI_ISL_8831684, EPI_ISL_8831787, EPI_ISL_8831793, EPI_ISL_8831823, EPI_ISL_8831906, EPI_ISL_8831951, EPI_ISL_8832056, EPI_ISL_8832110, EPI_ISL_8832137, EPI_ISL_8832171, EPI_ISL_8832174, EPI_ISL_8832176, EPI_ISL_8832200, EPI_ISL_8832304, EPI_ISL_8832306, EPI_ISL_8832340, EPI_ISL_8832350, EPI_ISL_8832523, EPI_ISL_8832593, EPI_ISL_8832678, EPI_ISL_8832745, EPI_ISL_8832774, EPI_ISL_8832872, EPI_ISL_8832953, EPI_ISL_8833057, EPI_ISL_8833195, EPI_ISL_8833220, EPI_ISL_8833338, EPI_ISL_8833397, EPI_ISL_8833416, EPI_ISL_8833495, EPI_ISL_8833496, EPI_ISL_8833507, EPI_ISL_8833526, EPI_ISL_8833542, EPI_ISL_8833589, EPI_ISL_8833617, EPI_ISL_8833632, EPI_ISL_8833648, EPI_ISL_8833710, EPI_ISL_8833744, EPI_ISL_8833794, EPI_ISL_8833842, EPI_ISL_8833860, EPI_ISL_8833918, EPI_ISL_8833991, EPI_ISL_8834010, EPI_ISL_8834021, EPI_ISL_8834028, EPI_ISL_8834053, EPI_ISL_8834149, EPI_ISL_8834213, EPI_ISL_8834222, EPI_ISL_8834253, EPI_ISL_8834271			
see above	Department of Clinical Microbiology and Center for Genomic Medicine, Rigshospitalet, Copenhagen, Denmark	Statens Serum Institut Bioinformatics and Microbial Genomics	Danish Covid-19 Genome Consortium
EPI_ISL_8830460, EPI_ISL_8830479	GenePath, Pune	RF-IISER Pune	Aurnab Ghose; Joy Merwin Monteiro; Krishanpal Karmodiya; Nikhil Phadke; Shatakshi Ranade
EPI_ISL_8833405	KKP Kelas 1 Soekarno-Hatta (iLab)	National Institute of Health Research and Development	Arie Ardiansyah Nugraha; Fajar Nur Sulistiyohadi; Hana Apsari Pawestri; Hartanti Dian Ikawati; Kartika Dewi Puspa; Nelly Puspandari; Nur Ika Hariastuti; Putri Widia; Subangkit; Vivi Setiawaty
EPI_ISL_8826405, EPI_ISL_8826406, EPI_ISL_8826408, EPI_ISL_8826429	Lancet Laboratories Gaborone	Botswana Harvard HIV Reference Laboratory	Boitumelo Zuze; Botshelo Radibe; Dorcas Maruapula; Ellen Nkitse; Joseph Makhema; Keoratile Ntshambiwa; Kgomotso Moruisi; Legodile Kooepile; Leyi Ncube; Mosana Moyo; Mosepele Mosepele; Mphaphi B. Mbulawa; Ontlametse T. Bareng; Pamela Smith-Lawrence; Roger Shapiro; Sefetogi Ramaologa; Shahin Lockman; Sikhulile Moyo; Simani Gaseitsiwe; Thongbotho Mphoyakgosi; Wonderful T. Choga
EPI_ISL_8826412, EPI_ISL_8826449	National Health Laboratory	Botswana Harvard HIV Reference Laboratory	Boitumelo J. L Zuze; Botshelo Radibe; Dorcas Maruapula; Joseph Makhema; Keoratile Ntshambiwa; Kgomotso Moruisi; Legodile Kooepile; Letsibogo Gaoraelwe; Mosepele Mosepele; Mphaphi B. Mbulawa; Ontlametse T. Bareng; Pamela Smith-Lawrence; Roger Shapiro; Sefetogi Ramaologa; Shahin Lockman; Sikhulile Moyo; Simani Gaseitsiwe; Thela Tefelo; Thongbotho Mphoyakgosi; Wonderful T. Choga
EPI_ISL_8828687	Nevada State Public Health Laboratory	Nevada State Public Health Laboratory	Andrew Gorzalski; Lynette Gumbleton; Mark Pandori
EPI_ISL_8833404	PRVKP FKUI	National Institute of Health Research and Development	Arie Ardiansyah Nugraha; Fajar Nur Sulistiyohadi; Hana Apsari Pawestri; Hartanti Dian Ikawati; Kartika Dewi Puspa; Nelly Puspandari; Subangkit; Triyani Soekarso; Vivi Setiawaty
EPI_ISL_8826512, EPI_ISL_8826515, EPI_ISL_8826523	Palapye Primary Hospital Laboratory	Botswana Harvard HIV Reference Laboratory	Boitumelo J. L Zuze; Botshelo Radibe; Dorcas Maruapula; Joseph Makhema; Keoratile Ntshambiwa; Kgomotso Moruisi; Legodile Kooepile; Letsibogo Gaoraelwe; Mosepele Mosepele; Mphaphi B. Mbulawa; Ontlametse T. Bareng; Pamela Smith-Lawrence; Roger Shapiro; Sefetogi Ramaologa; Shahin Lockman; Sikhulile Moyo; Simani Gaseitsiwe; Thela Tefelo; Thongbotho Mphoyakgosi; Wonderful T. Choga
EPI_ISL_8829822	Spital Langenthal	Medizinische Klinik, Spital Region Oberaargau	Alexander Imhof; Cedric Howald; Deborah Penet; Henri Pegeot; Ioannis Xenarios; Keith Harshman; Lorenzo Cerutti; Melyssa Elies
EPI_ISL_8828357	University of New Mexico Hospital	Center for Global Health, University of New Mexico Health Sciences Center	Darrell Dinwiddie; Daryl Domman; Jesse Young; Jon Femling; Justin Bacca; Kurt Schwalm; Valerie Morley

We gratefully acknowledge the following Authors from the Originating laboratories responsible for obtaining the specimens, as well as the Submitting laboratories where the genome data were generated and shared via GISAID, on which this research is based.

All Submitters of data may be contacted directly via www.gisaid.org

Authors are sorted alphabetically.

Accession ID	Originating Laboratory	Submitting Laboratory	Authors
EPI_ISL_8870728, EPI_ISL_8870770, EPI_ISL_8871007, EPI_ISL_8871878	Aegis Sciences Corporation	Centers for Disease Control and Prevention Division of Viral Diseases, Pathogen Discovery	Alec Vest; Benjamin Rambo-Martin; Christopher Gulvick; Clinton Paden; Cyndi Clark; Dakota Howard; Dhwani Batra; Dillon Nall; Duncan MacCannell; Erisa Sula; Ethan Sanders; Holly Houdeshell; Jason Caravas; Kristine Lacek; Matthew Hardison; Matthew Schmerer; Ola Kvalvaag; Patrick Campbell; Peter Cook; Rob Case; Scott Sammons; Shatavia Morrison; Shaun Westlund; Tymeckia Kendall; Victoria Caban Figueroa; Vikramsinha Ghorpade; Yvette Unoarumhi
EPI_ISL_8868207	Dept of Microbiology, Maulana Azad Medical College	GENOME SEQUENCING LABORATARY, Dept of Pediatrics, LOK NAYAK Hospital	Arvind Mohan; Avinash Lomash; INSACOG; Meenakshi Bothra; Mohammed Faruq; Oves Siddiqui; Prashanth N Suravajhala; Seema Kapoor.Sandeep Garg; Somesh Kumar; Sonal Saxena; Sunil Kumar Polipalli; Suresh Kumar; Vikas Manchanda
EPI_ISL_8844502, EPI_ISL_8847010, EPI_ISL_8847087	Edmonton Provincial Lab	Public Health Agency of Canada (PHAC) National Microbiology Laboratory	Buss; Croxen M; Deo A; Dieu P; E; Ferrato C; Gill K; Khan F; Koleva P; Li V; Lloyd C; Lynch T; Ma R; Murphy S; Pabbaraju K; Shokoples S; Thayer J; Tipples G; Whitehouse M; Wong A; Yu C; Zelyas N
EPI_ISL_8876222	FYR Diagnostics	FYR Diagnostics	Blohm; Ghai; Tre; Vikas
EPI_ISL_8848580, EPI_ISL_8848581, EPI_ISL_8848582, EPI_ISL_8848584, EPI_ISL_8848797, EPI_ISL_8854084	Lighthouse Lab in Alderley Park	Wellcome Sanger Institute for the COVID-19 Genomics UK (COG-UK) Consortium	Cordelia Langford; David K. Jackson; Dominic Kwiatkowski; Ewan Harrison; Ian Johnston; Jacquelyn Wynn; Jeffrey Barrett; John Sillitoe on behalf of the Wellcome Sanger Institute COVID-19 Surveillance Team; Mairead Hyland; Roberto Amato; Sonia Goncalves; The Lighthouse Lab in Alderley Park and Alex Alderton
EPI_ISL_8864505	Lighthouse Lab in Glasgow	Wellcome Sanger Institute for the COVID-19 Genomics UK (COG-UK) Consortium	Anna Dominiczak and Alex Alderton; Carol Clugston; Cordelia Langford; David Gray; David K. Jackson; Dominic Kwiatkowski; Ewan Harrison; Harper VanSteenhouse; Ian Johnston; Jeffrey Barrett; John Sillitoe on behalf of the Wellcome Sanger Institute COVID-19 Surveillance Team; Roberto Amato; Sonia Goncalves; Yumi Kasai
EPI_ISL_8863120, EPI_ISL_8863128	Michigan Department of Health and Human Services, Bureau of Laboratories	Michigan Department of Health and Human Services, Bureau of Laboratories	Blankenship HM; Riner D; Soehnlen MK
EPI_ISL_8843407	Ministry of Health Turkey	Ministry of Health Turkey	Fatma Bayrakdar; Gülay Korukluoğlu; Gültekin Ünal; Süleyman Yalcin; Yasemin Coşgun
EPI_ISL_8839391, EPI_ISL_8839392, EPI_ISL_8839393, EPI_ISL_8839394, EPI_ISL_8839395, EPI_ISL_8839396, EPI_ISL_8839397, EPI_ISL_8839398, EPI_ISL_8839399			
see above	Nastavni zavod za javno zdravstvo Splitsko- Dalmatinske županije	Hrvatski zavod za javno zdravstvo	Anita Jurić; Dragan Jurić; Irena Tabain; Ivana Ferenčak; Josipa Kuzle
EPI_ISL_8859258, EPI_ISL_8859272	PathWest Laboratory Medicine WA	PathWest Laboratory Medicine WA Microbial Surveillance Unit	PathWest Laboratory Medicine WA Microbial Surveillance Unit
EPI_ISL_8841258	Platform BIS UZA/UAntwerpen	Labo Klinische Biologie, UZA	Basil Britto Xavier; Christine Lammens; Herman Goossens; Ines Verbesselt; Jasmine Coppens; Kathleen Holemans; Marie Le Mercier; Veerle Matheeussen
EPI_ISL_8873222, EPI_ISL_8873234, EPI_ISL_8873236, EPI_ISL_8873241, EPI_ISL_8873243	Quest Diagnostics Incorporated	Centers for Disease Control and Prevention Division of Viral Diseases, Pathogen Discovery	A. Gerasimova; A. Perez; B. Anderson; Benjamin Rambo-Martin; Christopher Gulvick; Clinton Paden; Dakota Howard; Dhwani Batra; Duncan MacCannell; Erisa Sula; F. Lacbawan; I. Shlyakhter; Jason Caravas; K. Livingston; Kristine Lacek; L. Bernstein; M. Hua; Matthew Schmerer; P. Tanpaiboon; Peter Cook; R. Kagan; R. Owen; R. Rolando; S. Rosenthal; Scott Sammons; Shatavia Morrison; Tymeckia Kendall; Victoria Caban Figueroa; Y. Liu; Yvette Unoarumhi
EPI_ISL_8840922, EPI_ISL_8840937, EPI_ISL_8840983, EPI_ISL_8840998	Respiratory Virus Unit, Microbiology Services Colindale, Public Health England	COVID-19 Genomics UK (COG-UK) Consortium	PHE Covid Sequencing Team
EPI_ISL_8841176, EPI_ISL_8841177, EPI_ISL_8841178, EPI_ISL_8841191, EPI_ISL_8841193, EPI_ISL_8841227, EPI_ISL_8841228, EPI_ISL_8841237			
see above	Virology Unit, Institut Pasteur du Cambodge	Virology Unit, Institut Pasteur du Cambodge	Cecile Troupin; Chau Darapheak; Chin Savuth; Erik A Karlsson; Jurre Y Siegers; Kraing Sidonn; Leakhena Pum; Ly Sovann; Sophoannadedh Rath; Veasna Duong; Yi Sengdoeurn
EPI_ISL_8840071	Zavod za javno zdravstvo Istarske županije	Hrvatski zavod za javno zdravstvo	Anita Jurić; Dragan Jurić; Irena Tabain; Ivana Ferenčak; Josipa Kuzle
EPI_ISL_8843990	Zavod za javno zdravstvo Zagrebačke županije	Hrvatski zavod za javno zdravstvo	Anita Jurić; Dragan Jurić; Irena Tabain; Ivana Ferenčak; Josipa Kuzle

We gratefully acknowledge the following Authors from the Originating laboratories responsible for obtaining the specimens, as well as the Submitting laboratories where the genome data were generated and shared via GISAID, on which this research is based.

All Submitters of data may be contacted directly via www.gisaid.org

Authors are sorted alphabetically.

Accession ID	Originating Laboratory	Submitting Laboratory	Authors
EPI_ISL_8835086, EPI_ISL_8835091, EPI_ISL_8835098, EPI_ISL_8835119, EPI_ISL_8835123, EPI_ISL_8835128, EPI_ISL_8835129, EPI_ISL_8835133, EPI_ISL_8835137, EPI_ISL_8835139, EPI_ISL_8835146, EPI_ISL_8835152, EPI_ISL_8835154, EPI_ISL_8835156, EPI_ISL_8835163, EPI_ISL_8835164, EPI_ISL_8835166, EPI_ISL_8835168, EPI_ISL_8835171, EPI_ISL_8835176, EPI_ISL_8835178, EPI_ISL_8835190, EPI_ISL_8835196, EPI_ISL_8835205, EPI_ISL_8835207, EPI_ISL_8835227, EPI_ISL_8835231, EPI_ISL_8835236, EPI_ISL_8835239, EPI_ISL_8835249, EPI_ISL_8835251, EPI_ISL_8835269, EPI_ISL_8835275			
see above	Department of Bacteria, Parasites and Fungi, Statens Serum Institut, Copenhagen, Denmark	Statens Serum Institut Bioinformatics and Microbial Genomics	Danish Covid-19 Genome Consortium
EPI_ISL_8835116, EPI_ISL_8835157, EPI_ISL_8835218, EPI_ISL_8835223, EPI_ISL_8835282	Department of Clinical Microbiology and Center for Genomic Medicine, Rigshospitalet, Copenhagen, Denmark	Statens Serum Institut Bioinformatics and Microbial Genomics	Danish Covid-19 Genome Consortium
EPI_ISL_8836880, EPI_ISL_8836881, EPI_ISL_8836882, EPI_ISL_8836883, EPI_ISL_8836884, EPI_ISL_8836885, EPI_ISL_8836886, EPI_ISL_8836887, EPI_ISL_8836888			
see above	Division of Emerging Infectious Diseases, Bureau of Infectious Diseases Diagnosis Control, Korea Disease Control and Prevention Agency	Division of Emerging Infectious Diseases, Bureau of Infectious Diseases Diagnosis Control, Korea Disease Control and Prevention Agency	Ae Kyung Park; Chae Young Lee; Eun-Jin Kim; Hyuck Jin Lee; Il-Hwan Kim; Jeong-Ah Kim
EPI_ISL_8837764, EPI_ISL_8837795	Vitomed Sp. z o.o.	1. Academic Center for Pathomorphological and Genetic-Molecular Diagnostics ltd, Bialystok, Poland 2. National Institute of Public Health - National Institute of Hygiene, Warsaw, Poland	Anetta Sulewska; Jacek Nikliński; Janusz Dzięcioł; Joanna Kiśluk; Katarzyna Zacharczuk; Konrad Raczkowski; Magdalena Nowakowska; Małgorzata Sadkowska-Todys; Piotr Karabowicz; Piotr Majewski; Przemysław Biecek. Joanna Reszeć; Radosław Charkiewicz; Tomasz Wołkowicz
EPI_ISL_8837797	Wojewódzki Szpital Zespolony w Lesznie	1. Academic Center for Pathomorphological and Genetic-Molecular Diagnostics ltd, Bialystok, Poland 2. National Institute of Public Health - National Institute of Hygiene, Warsaw, Poland	Anetta Sulewska; Jacek Nikliński; Janusz Dzięcioł; Joanna Kiśluk; Katarzyna Zacharczuk; Konrad Raczkowski; Magdalena Nowakowska; Małgorzata Sadkowska-Todys; Piotr Karabowicz; Piotr Majewski; Przemysław Biecek. Joanna Reszeć; Radosław Charkiewicz; Tomasz Wołkowicz

We gratefully acknowledge the following Authors from the Originating laboratories responsible for obtaining the specimens, as well as the Submitting laboratories where the genome data were generated and shared via GISAID, on which this research is based.

All Submitters of data may be contacted directly via www.gisaid.org

Authors are sorted alphabetically.

Acknowledgement EPI_SET Identifier: EPI_SET_20220527by

Accession ID	Originating Laboratory	Submitting Laboratory	Authors
EPI_ISL_8834794, EPI_ISL_8834803, EPI_ISL_8834810, EPI_ISL_8834817, EPI_ISL_8834825, EPI_ISL_8834838, EPI_ISL_8834841, EPI_ISL_8834864, EPI_ISL_8834876, EPI_ISL_8834878, EPI_ISL_8834885, EPI_ISL_8834891, EPI_ISL_8834892, EPI_ISL_8834906, EPI_ISL_8834908, EPI_ISL_8834926, EPI_ISL_8834938, EPI_ISL_8834940, EPI_ISL_8834952, EPI_ISL_8834954, EPI_ISL_8834956, EPI_ISL_8834965, EPI_ISL_8834967, EPI_ISL_8834970, EPI_ISL_8834971, EPI_ISL_8834985, EPI_ISL_8834987, EPI_ISL_8834997, EPI_ISL_8835001, EPI_ISL_8835006, EPI_ISL_8835013, EPI_ISL_8835015, EPI_ISL_8835018, EPI_ISL_8835019, EPI_ISL_8835024, EPI_ISL_8835032, EPI_ISL_8835041, EPI_ISL_8835043, EPI_ISL_8835049, EPI_ISL_8835055, EPI_ISL_8835064, EPI_ISL_8835066, EPI_ISL_8835071, EPI_ISL_8835072, EPI_ISL_8835075, EPI_ISL_8835076, EPI_ISL_8835080			
see above	Department of Bacteria, Parasites and Fungi, Statens Serum Institut, Copenhagen, Denmark	Statens Serum Institut Bioinformatics and Microbial Genomics	Danish Covid-19 Genome Consortium
EPI_ISL_8834857, EPI_ISL_8834934, EPI_ISL_8835068	Department of Clinical Microbiology and Center for Genomic Medicine, Rigshospitalet, Copenhagen, Denmark	Statens Serum Institut Bioinformatics and Microbial Genomics	Danish Covid-19 Genome Consortium

We gratefully acknowledge the following Authors from the Originating laboratories responsible for obtaining the specimens, as well as the Submitting laboratories where the genome data were generated and shared via GISAID, on which this research is based.

All Submitters of data may be contacted directly via www.gisaid.org

Authors are sorted alphabetically.

Accession ID	Originating Laboratory	Submitting Laboratory	Authors
EPI_ISL_8834544, EPI_ISL_8834547, EPI_ISL_8834558, EPI_ISL_8834560, EPI_ISL_8834582, EPI_ISL_8834585, EPI_ISL_8834588, EPI_ISL_8834593, EPI_ISL_8834595, EPI_ISL_8834597, EPI_ISL_8834612, EPI_ISL_8834616, EPI_ISL_8834626, EPI_ISL_8834636, EPI_ISL_8834639, EPI_ISL_8834641, EPI_ISL_8834646, EPI_ISL_8834654, EPI_ISL_8834667, EPI_ISL_8834671, EPI_ISL_8834685, EPI_ISL_8834688, EPI_ISL_8834690, EPI_ISL_8834696, EPI_ISL_8834703, EPI_ISL_8834705, EPI_ISL_8834707, EPI_ISL_8834709, EPI_ISL_8834711, EPI_ISL_8834716, EPI_ISL_8834720, EPI_ISL_8834721, EPI_ISL_8834727, EPI_ISL_8834730, EPI_ISL_8834734, EPI_ISL_8834739, EPI_ISL_8834751, EPI_ISL_8834756, EPI_ISL_8834758, EPI_ISL_8834764, EPI_ISL_8834770, EPI_ISL_8834776, EPI_ISL_8834780, EPI_ISL_8834783, EPI_ISL_8834785, EPI_ISL_8834792			
see above	Department of Bacteria, Parasites and Fungi, Statens Serum Institut, Copenhagen, Denmark	Statens Serum Institut Bioinformatics and Microbial Genomics	Danish Covid-19 Genome Consortium
EPI_ISL_8834580, EPI_ISL_8834591, EPI_ISL_8834599, EPI_ISL_8834729	Department of Clinical Microbiology and Center for Genomic Medicine, Rigshospitalet, Copenhagen, Denmark	Statens Serum Institut Bioinformatics and Microbial Genomics	Danish Covid-19 Genome Consortium

We gratefully acknowledge the following Authors from the Originating laboratories responsible for obtaining the specimens, as well as the Submitting laboratories where the genome data were generated and shared via GISAID, on which this research is based.

All Submitters of data may be contacted directly via www.gisaid.org

Authors are sorted alphabetically.

Accession ID	Originating Laboratory	Submitting Laboratory	Authors
EPI_ISL_8834345, EPI_ISL_8834357, EPI_ISL_8834365, EPI_ISL_8834367, EPI_ISL_8834372, EPI_ISL_8834374, EPI_ISL_8834380, EPI_ISL_8834381, EPI_ISL_8834384, EPI_ISL_8834388, EPI_ISL_8834400, EPI_ISL_8834402, EPI_ISL_8834405, EPI_ISL_8834407, EPI_ISL_8834412, EPI_ISL_8834417, EPI_ISL_8834419, EPI_ISL_8834424, EPI_ISL_8834426, EPI_ISL_8834435, EPI_ISL_8834438, EPI_ISL_8834444, EPI_ISL_8834452, EPI_ISL_8834454, EPI_ISL_8834456, EPI_ISL_8834459, EPI_ISL_8834465, EPI_ISL_8834470, EPI_ISL_8834474, EPI_ISL_8834477, EPI_ISL_8834479, EPI_ISL_8834482, EPI_ISL_8834484, EPI_ISL_8834488, EPI_ISL_8834498, EPI_ISL_8834500, EPI_ISL_8834503, EPI_ISL_8834508, EPI_ISL_8834511, EPI_ISL_8834517, EPI_ISL_8834519, EPI_ISL_8834535, EPI_ISL_8834537, EPI_ISL_8834540			
see above	Department of Bacteria, Parasites and Fungi, Statens Serum Institut, Copenhagen, Denmark	Statens Serum Institut Bioinformatics and Microbial Genomics	Danish Covid-19 Genome Consortium
EPI_ISL_8834344, EPI_ISL_8834354, EPI_ISL_8834391, EPI_ISL_8834396, EPI_ISL_8834449, EPI_ISL_8834496	Department of Clinical Microbiology and Center for Genomic Medicine, Rigshospitalet, Copenhagen, Denmark	Statens Serum Institut Bioinformatics and Microbial Genomics	Danish Covid-19 Genome Consortium

We gratefully acknowledge the following Authors from the Originating laboratories responsible for obtaining the specimens, as well as the Submitting laboratories where the genome data were generated and shared via GISAID, on which this research is based.

All Submitters of data may be contacted directly via www.gisaid.org

Authors are sorted alphabetically.

Acknowledgement EPI_SET Identifier: EPI_SET_20220527ck

Accession ID	Originating Laboratory	Submitting Laboratory	Authors
EPI_ISL_8886634, EPI_ISL_8886635, EPI_ISL_8886637	Addu Equitorial Hospital	Addu Equitorial Hospital	D. Fathmath Nazla Rafeeq; Dr. Ibrahim Afzal; Mr. Ibrahim Nishan Ahmed; Ms. Aishath Shuhudha; Ms. Aminath Shazleena Abdul Rahman; Ms. Soafy Mohamed
EPI_ISL_8878410	Broad Institute Clinical Research Sequencing Platform	Infectious Disease Program, Broad Institute of Harvard and MIT	Adams, G.; B.L.; B.W.; Bauer, M.; Birren; Blumenstiel, B.; Brown, C.; Carter, A.; Chaluvadi, S.; D.J.; DeFelice, M.; DeRuff, K.; Dodge, S.; Gabriel, S.; Gallagher, G.; Gladden-Young, A.; Granger, B.; J.E.; K.J.; Lagerborg, K.; Larkin, K.; Lee, M.; Lemieux; Lennon, N.; Loreth, C.; Madoff, L.; McGovern, S.; Meldrim, J.; Normandin, E.; P.C.; Park; Pearlman, L.; Reilly, S.; Rudy, M.; Sabeti; Siddle; Smole, S.; Tomkins-Tinch, C.; Vicente, G.; and MacInnis
EPI_ISL_8896430, EPI_ISL_8896433, EPI_ISL_8896435, EPI_ISL_8896444	CAPRISA	CERI, Centre for Epidemic Response and Innvoation, Stellenbosch University and KRISP, KZN Research Innovation and Sequencing Platform, UKZN.	Aida Sivro; Giandhari J; Moir M; Naidoo Y; Natasha Samsunder; Pillay S; Ramphal U; Ramphal Y; San JE; Tegally H; Tshiabuila D; Van Wyk S; Wilkinson E; de Oliveira T
EPI_ISL_8885555, EPI_ISL_8885557, EPI_ISL_8885559, EPI_ISL_8885698, EPI_ISL_8885701, EPI_ISL_8885702, EPI_ISL_8885704, EPI_ISL_8885705, EPI_ISL_8885706, EPI_ISL_8885708, EPI_ISL_8885710, EPI_ISL_8885715, EPI_ISL_8885717, EPI_ISL_8885719, EPI_ISL_8885724, EPI_ISL_8885726, EPI_ISL_8885728, EPI_ISL_8885729, EPI_ISL_8885730, EPI_ISL_8885731, EPI_ISL_8885733, EPI_ISL_8885734, EPI_ISL_8885739, EPI_ISL_8885740, EPI_ISL_8885745, EPI_ISL_8885746, EPI_ISL_8885747, EPI_ISL_8885751, EPI_ISL_8885754, EPI_ISL_8885755, EPI_ISL_8885757, EPI_ISL_8885761, EPI_ISL_8885763, EPI_ISL_8885765, EPI_ISL_8885766, EPI_ISL_8885769, EPI_ISL_8885770, EPI_ISL_8885772, EPI_ISL_8885773, EPI_ISL_8885774, EPI_ISL_8885855, EPI_ISL_8885986, EPI_ISL_8885993, EPI_ISL_8885995, EPI_ISL_8885997, EPI_ISL_8886001, EPI_ISL_8886002, EPI_ISL_8886003, EPI_ISL_8886004, EPI_ISL_8886005, EPI_ISL_8886006, EPI_ISL_8886007, EPI_ISL_8886009, EPI_ISL_8886010, EPI_ISL_8886013, EPI_ISL_8886014, EPI_ISL_8886015, EPI_ISL_8886016, EPI_ISL_8886017, EPI_ISL_8886020, EPI_ISL_8886023, EPI_ISL_8886024, EPI_ISL_8886029, EPI_ISL_8886035, EPI_ISL_8886038, EPI_ISL_8886040, EPI_ISL_8886042, EPI_ISL_8886046, EPI_ISL_8886049, EPI_ISL_8886052, EPI_ISL_8886054, EPI_ISL_8886056, EPI_ISL_8886061, EPI_ISL_8886063, EPI_ISL_8886074, EPI_ISL_8886078, EPI_ISL_8886083, EPI_ISL_8886092, EPI_ISL_8886102, EPI_ISL_8886109, EPI_ISL_8886113, EPI_ISL_8886117, EPI_ISL_8886120, EPI_ISL_8886122, EPI_ISL_8886189, EPI_ISL_8886296, EPI_ISL_8886435, EPI_ISL_8886438, EPI_ISL_8886440, EPI_ISL_8886442, EPI_ISL_8886447, EPI_ISL_8886453, EPI_ISL_8886455, EPI_ISL_8886459, EPI_ISL_8886462, EPI_ISL_8886464, EPI_ISL_8886469, EPI_ISL_8886471, EPI_ISL_8886475, EPI_ISL_8886476, EPI_ISL_8886482, EPI_ISL_8886483, EPI_ISL_8886484, EPI_ISL_8886485, EPI_ISL_8886487, EPI_ISL_8886489, EPI_ISL_8886492, EPI_ISL_8886493, EPI_ISL_8886497, EPI_ISL_8886500, EPI_ISL_8886502, EPI_ISL_8886503, EPI_ISL_8886504, EPI_ISL_8886506, EPI_ISL_8886512, EPI_ISL_8886513, EPI_ISL_8886518, EPI_ISL_8886523, EPI_ISL_8886526, EPI_ISL_8886528, EPI_ISL_8886533, EPI_ISL_8886535, EPI_ISL_8886553, EPI_ISL_8886587, EPI_ISL_8886640, EPI_ISL_8886643, EPI_ISL_8886644, EPI_ISL_8886646, EPI_ISL_8886647, EPI_ISL_8886649, EPI_ISL_8886650, EPI_ISL_8886652, EPI_ISL_8886655, EPI_ISL_8886657, EPI_ISL_8886659, EPI_ISL_8886666, EPI_ISL_8886670, EPI_ISL_8886672, EPI_ISL_8886674, EPI_ISL_8886676, EPI_ISL_8886681, EPI_ISL_8886682, EPI_ISL_8886684, EPI_ISL_8886685, EPI_ISL_8886687, EPI_ISL_8886690, EPI_ISL_8886691, EPI_ISL_8886694, EPI_ISL_8886695, EPI_ISL_8886696, EPI_ISL_8886710, EPI_ISL_8886715, EPI_ISL_8886717, EPI_ISL_8886719, EPI_ISL_8886720, EPI_ISL_8886725, EPI_ISL_8886728, EPI_ISL_8886729, EPI_ISL_8886731, EPI_ISL_8886737, EPI_ISL_8886739, EPI_ISL_8886743, EPI_ISL_8886744, EPI_ISL_8886749, EPI_ISL_8886750, EPI_ISL_8886752, EPI_ISL_8886753, EPI_ISL_8886755, EPI_ISL_8886756, EPI_ISL_8886760, EPI_ISL_8886761, EPI_ISL_8886762, EPI_ISL_8886764, EPI_ISL_8886766, EPI_ISL_8886767, EPI_ISL_8886769, EPI_ISL_8886770, EPI_ISL_8886771, EPI_ISL_8886772, EPI_ISL_8886773, EPI_ISL_8886774, EPI_ISL_8886778, EPI_ISL_8886782, EPI_ISL_8886786, EPI_ISL_8886789, EPI_ISL_8886800, EPI_ISL_8886804, EPI_ISL_8886805, EPI_ISL_8886813, EPI_ISL_8886816, EPI_ISL_8886817, EPI_ISL_8886818, EPI_ISL_8886822, EPI_ISL_8886825, EPI_ISL_8886826, EPI_ISL_8886831, EPI_ISL_8886832, EPI_ISL_8886846, EPI_ISL_8886847, EPI_ISL_8886848, EPI_ISL_8886851, EPI_ISL_8886856, EPI_ISL_8886858, EPI_ISL_8886860, EPI_ISL_8886862, EPI_ISL_8886864, EPI_ISL_8886865, EPI_ISL_8886866, EPI_ISL_8886868, EPI_ISL_8886870, EPI_ISL_8886873, EPI_ISL_8886931, EPI_ISL_8886932, EPI_ISL_8886933, EPI_ISL_8886934, EPI_ISL_8886935, EPI_ISL_8886936, EPI_ISL_8886937, EPI_ISL_8886938, EPI_ISL_8886939, EPI_ISL_8886941, EPI_ISL_8886944, EPI_ISL_8886945, EPI_ISL_8886947, EPI_ISL_8886952, EPI_ISL_8886953, EPI_ISL_8886954, EPI_ISL_8886955, EPI_ISL_8886956, EPI_ISL_8886957, EPI_ISL_8886958, EPI_ISL_8886959, EPI_ISL_8886960, EPI_ISL_8886963, EPI_ISL_8886965, EPI_ISL_8886968, EPI_ISL_8886970, EPI_ISL_8886971, EPI_ISL_8886972, EPI_ISL_8886973, EPI_ISL_8886975, EPI_ISL_8886976, EPI_ISL_8886979, EPI_ISL_8886981, EPI_ISL_8886984, EPI_ISL_8886986, EPI_ISL_8886988, EPI_ISL_8886989, EPI_ISL_8886998, EPI_ISL_8886999, EPI_ISL_8887000, EPI_ISL_8887001, EPI_ISL_8887002, EPI_ISL_8887005, EPI_ISL_8887008, EPI_ISL_8887009, EPI_ISL_8887013, EPI_ISL_8887015, EPI_ISL_8887016, EPI_ISL_8887018, EPI_ISL_8887020, EPI_ISL_8887021, EPI_ISL_8887024, EPI_ISL_8887025, EPI_ISL_8887033, EPI_ISL_8887035, EPI_ISL_8887036, EPI_ISL_8887038, EPI_ISL_8887040, EPI_ISL_8887045, EPI_ISL_8887046, EPI_ISL_8887048, EPI_ISL_8887050, EPI_ISL_8887052, EPI_ISL_8887053, EPI_ISL_8887056, EPI_ISL_8887058, EPI_ISL_8887071, EPI_ISL_8887073, EPI_ISL_8887074, EPI_ISL_8887075, EPI_ISL_8887076, EPI_ISL_8887079, EPI_ISL_8887082, EPI_ISL_8887085, EPI_ISL_8887086, EPI_ISL_8887088, EPI_ISL_8887092, EPI_ISL_8887093, EPI_ISL_8887097, EPI_ISL_8887098, EPI_ISL_8887099, EPI_ISL_8887100, EPI_ISL_8887102, EPI_ISL_8887108, EPI_ISL_8887111, EPI_ISL_8887114, EPI_ISL_8887115, EPI_ISL_8887119, EPI_ISL_8887124, EPI_ISL_8887131, EPI_ISL_8887133, EPI_ISL_8887135, EPI_ISL_8887136, EPI_ISL_8887138, EPI_ISL_8887139, EPI_ISL_8887141, EPI_ISL_8887145, EPI_ISL_8887147, EPI_ISL_8887153, EPI_ISL_8887155, EPI_ISL_8887158, EPI_ISL_8887160, EPI_ISL_8887163, EPI_ISL_8887164, EPI_ISL_8887174, EPI_ISL_8887176, EPI_ISL_8887178, EPI_ISL_8887184, EPI_ISL_8887187, EPI_ISL_8887188, EPI_ISL_8887191, EPI_ISL_8887193, EPI_ISL_8887196, EPI_ISL_8887202, EPI_ISL_8887208, EPI_ISL_8887213, EPI_ISL_8887218, EPI_ISL_8887219, EPI_ISL_8887223, EPI_ISL_8887225, EPI_ISL_8887226, EPI_ISL_8887231, EPI_ISL_8887232, EPI_ISL_8887236, EPI_ISL_8887237, EPI_ISL_8887239, EPI_ISL_8887240, EPI_ISL_8887243, EPI_ISL_8887245, EPI_ISL_8887249, EPI_ISL_8887256, EPI_ISL_8887258, EPI_ISL_8887260, EPI_ISL_8887262, EPI_ISL_8887263, EPI_ISL_8887264, EPI_ISL_8887265, EPI_ISL_8887266, EPI_ISL_8887270, EPI_ISL_8887272, EPI_ISL_8887275, EPI_ISL_8887278, EPI_ISL_8887281, EPI_ISL_8887284, EPI_ISL_8887289, EPI_ISL_8887290, EPI_ISL_8887291, EPI_ISL_8887292, EPI_ISL_8887296, EPI_ISL_8887300, EPI_ISL_8887302, EPI_ISL_8887303, EPI_ISL_8887307, EPI_ISL_8887394, EPI_ISL_8887395, EPI_ISL_8887397, EPI_ISL_8887398, EPI_ISL_8887399, EPI_ISL_8887400, EPI_ISL_8887405, EPI_ISL_8887406, EPI_ISL_8887408, EPI_ISL_8887409, EPI_ISL_8887411, EPI_ISL_8887413, EPI_ISL_8887415, EPI_ISL_8887416, EPI_ISL_8887418, EPI_ISL_8887433, EPI_ISL_8887435, EPI_ISL_8887438, EPI_ISL_8887441, EPI_ISL_8887445, EPI_ISL_8887450, EPI_ISL_8887452, EPI_ISL_8887453, EPI_ISL_8887456, EPI_ISL_8887457, EPI_ISL_8887458, EPI_ISL_8887462, EPI_ISL_8887466, EPI_ISL_8887467, EPI_ISL_8887469, EPI_ISL_8887471, EPI_ISL_8887475, EPI_ISL_8887476, EPI_ISL_8887478, EPI_ISL_8887479, EPI_ISL_8887495, EPI_ISL_8887497, EPI_ISL_8887500, EPI_ISL_8887505, EPI_ISL_8887511, EPI_ISL_8887515, EPI_ISL_8887516, EPI_ISL_8887518, EPI_ISL_8887521, EPI_ISL_8887522, EPI_ISL_8887524, EPI_ISL_8887527, EPI_ISL_8887530, EPI_ISL_8887533, EPI_ISL_8887534, EPI_ISL_8887535, EPI_ISL_8887536, EPI_ISL_8887538, EPI_ISL_8887541, EPI_ISL_8887542, EPI_ISL_8887545, EPI_ISL_8887548, EPI_ISL_8887550, EPI_ISL_8887554, EPI_ISL_8887556, EPI_ISL_8887559, EPI_ISL_8887561, EPI_ISL_8887564, EPI_ISL_8887566, EPI_ISL_8887574, EPI_ISL_8887578, EPI_ISL_8887580, EPI_ISL_8887583, EPI_ISL_8887584, EPI_ISL_8887585, EPI_ISL_8887586, EPI_ISL_8887589, EPI_ISL_8887779, EPI_ISL_8887805, EPI_ISL_8887806, EPI_ISL_8887807, EPI_ISL_8887811, EPI_ISL_8887814, EPI_ISL_8887818, EPI_ISL_8887822, EPI_ISL_8887829, EPI_ISL_8887833, EPI_ISL_8887880, EPI_ISL_8887881, EPI_ISL_8887887, EPI_ISL_8887889, EPI_ISL_8887892			
see above	Department of Bacteria, Parasites and Fungi, Statens Serum Institut, Copenhagen, Denmark	Statens Serum Institut Bioinformatics and Microbial Genomics	Danish Covid-19 Genome Consortium
EPI_ISL_8885401	Gujarat Pathology laboratory & Diagnostic Center, Paldi, Ahmedabad	Gujarat Biotechnology Research Centre	Akhilesh Modi; Apurvasinh Puvar; Bhadreshsinh Gohil; Chaitanya Joshi; Disha Vora; Janvi Raval; Jaykumar Rangani; Madhvi Joshi; Nimesh Patel; Niraj Kotha; Nitin Savaliya; NitinShukla; Priyank Chavda; Ramesh Pandit; Roshani Mishra; Sonal Sharma; Tasnim Trivedi; Zarna Patel
EPI_ISL_8886546, EPI_ISL_8886554, EPI_ISL_8886555, EPI_ISL_8886563, EPI_ISL_8886564, EPI_ISL_8886575, EPI_ISL_8886576, EPI_ISL_8886586, EPI_ISL_8886594, EPI_ISL_8886595, EPI_ISL_8886596, EPI_ISL_8886600			
see above	Indira Gandhi Memorial Hospital	Indira Gandhi Memorial Hospital	D. Fathmath Nazla Rafeeq; Dr. Ibrahim Afzal; Mr. Ibrahim Nishan Ahmed; Ms. Aishath Shuhudha; Ms. Aminath Shazleena Abdul Rahman; Ms. Soafy Mohamed
EPI_ISL_8897015	KKP Kelas 1 Soekarno-Hatta (iLab)	National Institute of Health Research and Development	Arie Ardiansyah Nugraha; Fajar Nur Sulistiyohadi; Hana Apsari Pawestri; Hartanti Dian Ikawati; Kartika Dewi Puspa; Nelly Puspandari; Nur Ika Hariastuti; Putri Widia; Subangkit; Vivi Setiawaty
EPI_ISL_8886623, EPI_ISL_8886624	Kudahuvadhoo Atoll Hospital	Kudahuvadhoo Atoll Hospital	D. Fathmath Nazla Rafeeq; Dr. Ibrahim Afzal; Mr. Ibrahim Nishan Ahmed; Ms. Aishath Shuhudha; Ms. Aminath Shazleena Abdul Rahman; Ms. Soafy Mohamed
EPI_ISL_8886609, EPI_ISL_8886610	Kulhudufushi Regional Hospital	Kulhudufushi Regional Hospital	D. Fathmath Nazla Rafeeq; Dr. Ibrahim Afzal; Mr. Ibrahim Nishan Ahmed; Ms. Aishath Shuhudha; Ms. Aminath Shazleena Abdul Rahman; Ms. Soafy Mohamed
EPI_ISL_8892176	Michigan Department of Health and Human Services, Bureau of Laboratories	Michigan Department of Health and Human Services, Bureau of Laboratories	Blankenship HM; Riner D; Soehnlen MK
EPI_ISL_8896467, EPI_ISL_8896476, EPI_ISL_8896477	National Health Laboratory Service, Kwazulu-Natal, South Africa	CERI, Centre for Epidemic Response and Innvoation, Stellenbosch University and KRISP, KZN Research Innovation and Sequencing Platform, UKZN.	Giandhari J; Moir M; Naidoo Y; Nokukhanya Mdlalose; Pillay S; Ramphal U; Ramphal Y; San JE; Tegally H; Tshiabuila D; Van Wyk S; Wilkinson E; de Oliveira T
EPI_ISL_8890649, EPI_ISL_8890650, EPI_ISL_8890651, EPI_ISL_8890652, EPI_ISL_8890653, EPI_ISL_8890654, EPI_ISL_8890655, EPI_ISL_8890656, EPI_ISL_8890657, EPI_ISL_8890658, EPI_ISL_8890659, EPI_ISL_8890675			
see above	National Microbiology Reference Laboratory, Ministry of Health, Harare, Zimbabwe	CERI, Centre for Epidemic Response and Innvoation, Stellenbosch University and KRISP, KZN Research Innovation and Sequencing Platform, UKZN.	Agnes Juru; Air Comodor Dr J. Chimedza; Charles Nyagupe; Dr Raiva Simbi; Giandhari J; Hlanai Gumbo; Kenneth Maeka; Moir M; Naidoo Y; Pillay S; Ramphal U; Ramphal Y; San JE; Tapfumanei Mashe; Tatenda Takawira; Tegally H; Tshiabuila D; Van Wyk S; Wilkinson E; de Oliveira T
EPI_ISL_8879944, EPI_ISL_8879948	Nevada State Public Health Laboratory	Nevada State Public Health Laboratory	Andrew Gorzalski; Lynette Gumbleton; Mark Pandori
EPI_ISL_8900901, EPI_ISL_8901099	Pathology West - NSW Health Pathology	NSW Health Pathology - Institute of Clinical Pathology and Medical Research; Westmead Hospital; University of Sydney	Arnott A.; Draper J.; Gall M.; Martinez E.; Rockett R.; Sintchenko V.; on behalf of ICPMR
EPI_ISL_8884476, EPI_ISL_8884549	Respiratory Virus Unit, Microbiology Services Colindale, Public Health England	COVID-19 Genomics UK (COG-UK) Consortium	PHE Covid Sequencing Team
EPI_ISL_8884441	Viral Research and Diagnostic Laboratories	NIV Influenza	Dr.Rupinder Bakshi

We gratefully acknowledge the following Authors from the Originating laboratories responsible for obtaining the specimens, as well as the Submitting laboratories where the genome data were generated and shared via GISAID, on which this research is based.

All Submitters of data may be contacted directly via www.gisaid.org

Authors are sorted alphabetically.

Accession ID	Originating Laboratory	Submitting Laboratory	Authors
EPI_ISL_8902791, EPI_ISL_8902818, EPI_ISL_8902819	Arkansas Public Health Laboratory, Arkansas Department of Health	University of Minnesota Genomics Center	Corbin Dirkx; Daryl M. Gohl; Jaquelyn Kuriger-Laber; John Garbe
EPI_ISL_8903524, EPI_ISL_8903528, EPI_ISL_8903529	Jessa	Jessa	Severine Berden et al. on behalf of the Jessa_cmdLab
EPI_ISL_8903174, EPI_ISL_8903217, EPI_ISL_8903251, EPI_ISL_8903317, EPI_ISL_8903373, EPI_ISL_8903423	National Virus Reference Laboratory	National Virus Reference Laboratory	Charlene Bennett; Cillian F De Gascun; Gabriel Gonzalez; Jonathan Dean; Michael Carr; Zoe Yandle
EPI_ISL_8901099, EPI_ISL_8901110	Pathology West - NSW Health Pathology	NSW Health Pathology - Institute of Clinical Pathology and Medical Research; Westmead Hospital; University of Sydney	Arnott A.; Draper J.; Gall M.; Martinez E.; Rockett R.; Sintchenko V.; on behalf of ICPMR
EPI_ISL_8907628, EPI_ISL_8909082	UW Virology Lab	UW Virology Lab	Alexander Greninger; Hong Xie; Isabel Arnould; Keith R Jerome; Meei-Li Huang; Nathan Breit; Patrick Mathias; Pavitra Roychoudhury; Pooneh Hajian; Ricardo Perez; Robert J. Livingston; Sean Ellis; Seffir T. Wendm; Shah Mohamed Bakhash

We gratefully acknowledge the following Authors from the Originating laboratories responsible for obtaining the specimens, as well as the Submitting laboratories where the genome data were generated and shared via GISAID, on which this research is based.

All Submitters of data may be contacted directly via www.gisaid.org

Authors are sorted alphabetically.

Accession ID	Originating Laboratory	Submitting Laboratory	Authors
EPI_ISL_9002785, EPI_ISL_9002788	Botswana Harvard HIV Reference Laboratory	Botswana Harvard HIV Reference Laboratory	Boitumelo Zuze; Botshelo Radibe; Dorcas Maruapula; Joseph Makhema; Keoratile Ntshambiwa; Kgomotso Moruisi; Legodile Kooepile; Mosepele Mosepele; Mphaphi B. Mbulawa; Ontlametse T. Bareng; Pamela Smith-Lawrence; Roger Shapiro; Sefetogi Ramaologa; Shahin Lockman; Sikhulile Moyo; Simani Gaseitsiwe; Thongbotho Mphoyakgosi; Wonderful T. Choga
EPI_ISL_9003447	CHEDV	Instituto Nacional de Saude (INSA)	Borges et al
EPI_ISL_9003416	CHTMAD	Instituto Nacional de Saude (INSA)	Borges et al
EPI_ISL_8986579, EPI_ISL_8986650	Clinical Molecular Microbiology Laboratory, UNC Hospitals	Jeremy Wang	Bravada Hill; Corbin Jones; Jeremy Wang; Katie Martin; Melissa Miller; Sayal Guirales-Medrano; Tara Skelly
EPI_ISL_9003527, EPI_ISL_9003556, EPI_ISL_9003563, EPI_ISL_9003581, EPI_ISL_9003583, EPI_ISL_9003594, EPI_ISL_9003597			
see above	Colorado Department of Public Health and Environment	Colorado Department of Public Health and Environment	Alexandria Rossheim; Arianna Smith; Diana Ir; Emily A. Travanty; Laura Bankers; Mandy Waters; Michael Martin; Molly C. Hetherington-Rauth; Shannon R. Matzinger
EPI_ISL_8976723, EPI_ISL_8976724, EPI_ISL_8976725, EPI_ISL_8976726, EPI_ISL_8976728, EPI_ISL_8976729, EPI_ISL_8976730, EPI_ISL_8976731, EPI_ISL_8976732, EPI_ISL_8976733, EPI_ISL_8976734, EPI_ISL_8976735, EPI_ISL_8976736, EPI_ISL_8976737			
see above	Curative Labs	Curative Labs	Elias L. Salfati; Eugenia Khorosheva; George Way; J.Cesar Ignacio-Espinoza; Janet Chen; Mikhail Hanewich-Hollatz; Nabjot Sandhu; Sophia Quasem; Vladimir Slepnev; Zhiyi Xie
EPI_ISL_8995768, EPI_ISL_8995769	DGHP Laboratory	Thailand MOPH - U.S. CDC Collaboration (TUC)	Beth Skaggs; Piroon Jenjaroenpun; Pongpun Sawatwong; Thidathip Wongsurawat
EPI_ISL_8985651	Diagnostyka. Laboratoria Medyczne.	1. ViroGenetics - BSL3 Laboratory of Virology, Małopolska Centre of Biotechnology, Jagiellonian University; 2. Diagtron Laboratoria Łukasz Rąbalski	Gromowski, T.; Kowalski, M.; Labaj; Maciej Kosinski; Mazur-Panasiuk, N.; P.P.; Pyrc, K.; Rabalski L.; Rogalska-Kupiec M.; Swadzba J.; Sylwia Januszczak; Szulc, P.; Wydmanski, W.
EPI_ISL_8998400, EPI_ISL_8998401, EPI_ISL_8998402, EPI_ISL_8998403, EPI_ISL_8998404, EPI_ISL_8998406	Division of Emerging Infectious Diseases, Bureau of Infectious Diseases Diagnosis Control, Korea Disease Control and Prevention Agency	Division of Emerging Infectious Diseases, Bureau of Infectious Diseases Diagnosis Control, Korea Disease Control and Prevention Agency	Ae Kyung Park; Chae Young Lee; Eun-Jin Kim; Hyuck Jin Lee; Il-Hwan Kim; Jeong-Ah Kim
EPI_ISL_8989282	Fakultní nemocnice u sv. Anny v Brně	Fakultní nemocnice u sv. Anny v Brně	Lenka Zamecnikova; Milada Dvořáčková. Jan Vrbsky
EPI_ISL_8955208	Fondazione IRCCS Ca' Granda Ospedale Maggiore Policlinico	Fondazione IRCCS Ca' Granda Ospedale Maggiore Policlinico	Ferruccio Ceriotti; Sara Uceda Renteria
EPI_ISL_8995757, EPI_ISL_8995763, EPI_ISL_8995764, EPI_ISL_8995766, EPI_ISL_8995767	Health Department, Bangkok Metropolitan Administration	Thailand MOPH - U.S. CDC Collaboration (TUC)	Beth Skaggs; Piroon Jenjaroenpun; Pongpun Sawatwong; Thidathip Wongsurawat
EPI_ISL_8995642, EPI_ISL_8995643, EPI_ISL_8995644	Mae Sot General Hospital	Thailand MOPH - U.S. CDC Collaboration (TUC)	Beth Skaggs; Piroon Jenjaroenpun; Pongpun Sawatwong; Thidathip Wongsurawat
EPI_ISL_8982022, EPI_ISL_8983039	Molecular Virology section, department of infection & immunity, KFSHRC	Molecular Virology section, department of infection & immunity, KFSHRC	Ahmed A. Al-Qahtani; Basma M. Alahideb; Fatimah S. Alhamlan; Feda A. Alsuwairi; Maha Al-Abdulkarim; Maysoon S. Mutabagani; Mohamed S. Asiri; Reem S. Almaghrabi Madain S. Alsanea; Sahar I. Althawadi; Sara A. Altamimi
EPI_ISL_9002773, EPI_ISL_9002790, EPI_ISL_9002795, EPI_ISL_9002810, EPI_ISL_9002814, EPI_ISL_9002815, EPI_ISL_9002822, EPI_ISL_9002836, EPI_ISL_9002859			
see above	National Health Laboratory	Botswana Harvard HIV Reference Laboratory	Boitumelo Zuze; Botshelo Radibe; Dorcas Maruapula; Joseph Makhema; Keoratile Ntshambiwa; Kgomotso Moruisi; Legodile Kooepile; Mosepele Mosepele; Mphaphi B. Mbulawa; Ontlametse T. Bareng; Pamela Smith-Lawrence; Roger Shapiro; Sefetogi Ramaologa; Shahin Lockman; Sikhulile Moyo; Simani Gaseitsiwe; Thongbotho Mphoyakgosi; Wonderful T. Choga
EPI_ISL_8958708	Northumbria University / South Tees Hospitals NHS Foundation Trust / North Cumbria Integrated Care NHS Foundation Trust / North Tees and Hartlepool NHS Foundation Trust / Newcastle Hospitals NHS Foundation Trust	COVID-19 Genomics UK (COG-UK) Consortium	Andrew Nelson; Brendan Payne; Clive Graham; Darren L Smith; Debra Padgett; Edward Barton; Emma Swindells; Garren Scott; Gary Black; Gary Eltringham; Giles S Holt; Greg R Young; Jane Greenaway; Jennifer Collins; John Allan; Joshua Loh; Lynn Dover; Matthew Bashton; Mohammad A Tariq; Paul Baker; Sarah Essex; Steve Liggett; Wen C Yew; Yusri Taha
EPI_ISL_8977384, EPI_ISL_8977385, EPI_ISL_8977386, EPI_ISL_8977387, EPI_ISL_8977388, EPI_ISL_8977392, EPI_ISL_8977394, EPI_ISL_8977397, EPI_ISL_8977400, EPI_ISL_8977401			
see above	Ochsner Health	BioInfoExperts	Amy Feehan; Ben Lain; Chris Huston; David J. Nolan; Judy Crabtree; Julia-Garcia-Diaz; Lucio Miele; Rebecca Rose; Samual Moot; Susanna L. Lamers; Tessa LaFleur
EPI_ISL_8960507, EPI_ISL_8960509, EPI_ISL_8961047	Originating lab: Wales Specialist Virology Centre Sequencing lab: Pathogen Genomics Unit	Public Health Wales Microbiology Cardiff Wales Specialist Virology Centre	Alec Birchley; Alexander Adams; Amy Gaskin; Angela Marchbank; Bree Gatica-Wilcox; Catherine Moore; Jason Coombes; Joanne Watkins; Joel Southgate; Johnathan Evans; Laura Gifford; Lauren Gilbert; Lee Graham; Malorie Perry; Matthew Bull; Nicole Pacchiarini; Sally Corden; Sara Kumziene-Summerhayes; Sara Rey; Sarah Taylor; Simon Cottrell; Sophie Jones; Tom Connor
EPI_ISL_8959296, EPI_ISL_8959335, EPI_ISL_8959727	Respiratory Virus Unit, Microbiology Services Colindale, Public Health England	COVID-19 Genomics UK (COG-UK) Consortium	PHE Covid Sequencing Team
EPI_ISL_9003415	SEEBMO	Instituto Nacional de Saude (INSA)	Borges et al
EPI_ISL_8993539, EPI_ISL_8993543	TSGH-CP molecular lab	TSGH-CP molecular lab	Cherng-Lih Perng; Chien-Wen Chen; Chih-Kai Chang; Feng-Yee Chang; Hsing-Yi Chung; Hung-Sheng Shang; Jung-Chung Lin; Kuo-Ming Yeh; Kuo-Sheng Hung; Ming-Jr JIAN; Sheng-Kang Chiu; Shih-Hung Tsai; Tien-Yao Chang
EPI_ISL_8986746	Tokyo Metropolitan Institute of Public Health	Tokyo Metropolitan Institute of Public Health	Akane Negishi; Arisa Amano; Hirofumi Miyake; Jun Suzuki; Kenji Sadamasu; Kenshirou Kuroki; Mami Nagashima; Masaki Hayashi; Maya Isogai; Takako Yamazaki; Takushi Fujiwara
EPI_ISL_8988612, EPI_ISL_8988613, EPI_ISL_8988614, EPI_ISL_8988615, EPI_ISL_8988617, EPI_ISL_8988618, EPI_ISL_8988619, EPI_ISL_8988620, EPI_ISL_8988621, EPI_ISL_8988622, EPI_ISL_8988623, EPI_ISL_8988624, EPI_ISL_8988626			
see above	UAN-LANIIA	Microbial Genomics Laboratory	; Alejandra García-Gasca; Alejandra Hernández-Terán; Alejandro Sánchez-Flores; Alfredo Herrera-Estrella; Alicia Ocaña-Mondragón; Andreu Comas-García; Angel Gustavo Salas-Lais; Antonio Loza Román; Bernardo Martínez-Miguel; Blanca Taboada; Brenda Irasema Maldonado-Meza; Bruno Gómez-Gil; Carla Ivón Herrera-Najera; Carlos F. Arias; Celia Boukadida; Clara Esperanza Santacruz-Tinoco; Concepción Grajales-Muñiz; Consorcio Mexicano de Vigilancia Genómica (CoViGen-Mex). Authors (in alphabetical order): Julio Elias Alvarado-Yaah; Cristóbal Cháidez-Quiróz; Célida Duque Molina; Célida Martínez-Rodríguez; Daniel Fregoso-Rueda; Daniel Lira Morales; Eduardo Becerril-Vargas; Fernando Fontove-Herrera; Fidencio Mejía-Nepomuceno; Francisco Pulido; Gloria Elena Espinosa-Ayala; Gloria María Molina-Salinas; Gloria Vazquez; Hector Esteban Paz-Juárez; Hector Montoya-Fuentes; Helen Haydee Fernanda Ramírez-Plascencia; Irvin González-López; Jean Pierre González; Jesús Hernández; Joel Armando Vázquez-Pérez.; Jorge Salas-Hernández; José Antonio Enciso-Moreno; José Arturo Martínez-Orozco; José Esteban Muñoz-Medina; José de Jesús Nuñez-Contreras; Juan Bautista Chale-Dzul; Julissa Enciso-Ibarra; Luis Alberto Ochoa-Carrera; Manuel Iván Girón Pérez; Margarita Matías-Florentino; Mario Mújica-Sánchez; Marissa Perez-Garcia; María Guadalupe Santiago-Mauricio; María Guadalupe de Jesús Mireles-Rivera; Nelly Sélem-Mojica; Pavel Isa; Ricardo Ciria Merce; Ricardo Grande; Rosa María Gutiérrez Rios; Santiago Ávila-Ríos; Selene Zárate; Susana Lopez; Verónica Mata-Haro; Victor Eduardo García-Arias; Víctor Hugo Borja-Aburto
EPI_ISL_9003407	ULS Castelo Branco	Instituto Nacional de Saude (INSA)	Borges et al
EPI_ISL_9003417	UNILABS	Instituto Nacional de Saude (INSA)	Borges et al
EPI_ISL_8976191	US Air Force School of Aerospace Medicine	US Air Force School of Aerospace Medicine	Anthony Fries; Carol Garrett; Clarise Starr; Deanna Muehleman; Elizabeth Macias; Jennifer Meyer; Kelsey Lanter; Sarah Purves; William Gruner
EPI_ISL_8986063, EPI_ISL_8986109, EPI_ISL_8986203, EPI_ISL_8986254, EPI_ISL_8986397, EPI_ISL_8986481, EPI_ISL_8986500			
see above	UW Virology Lab	UW Virology Lab	Alexander Greninger; Hong Xie; Isabel Arnould; Keith R Jerome; Meei-Li Huang; Nathan Breit; Patrick Mathias; Pavitra Roychoudhury; Pooneh Hajian; Ricardo Perez; Robert J. Livingston; Sean Ellis; Seffir T. Wendm; Shah Mohamed Bakhash
EPI_ISL_8981633	Virology, Universitätsklinikum des Saarlandes	Epigenetics, Saarland University	Barbara Berko-Göttel Sascha Tierling; Jörn Walter; Kathrin Kattler; Nastasia Seiwert; Sigrun Smola; Thorsten Pfuhl
EPI_ISL_8975529	Zakład Diagnostyki Laboratoryjnej Mazowiecki Szpital Bródnowski Sp. z	1. Academic Center for Pathomorphological and Genetic-	Anetta Sulewska; Jacek Nikliński; Janusz Dzięcioł; Joanna Kiśluk; Katarzyna Zacharczuk; Konrad Raczkowski; Magdalena Nowakowska; Małgorzata Sadkowska-Todys; Piotr Karabowicz; Piotr Majewski; Przemysław Biecek. Joanna Reszeć; Radosław Charkiewicz; Tomasz Wołkowicz

o.o. ul. Kondratowicza 8, o3-242 Warszawa

Molecular Diagnostics ltd, Bialystok, Poland 2. National Institute of Public Health - National Institute of Hygiene, Warsaw, Poland

We gratefully acknowledge the following Authors from the Originating laboratories responsible for obtaining the specimens, as well as the Submitting laboratories where the genome data were generated and shared via GISAID, on which this research is based.

All Submitters of data may be contacted directly via www.gisaid.org

Authors are sorted alphabetically.

Acknowledgement EPI_SET Identifier: EPI_SET_20220527bd

Accession ID	Originating Laboratory	Submitting Laboratory	Authors
EPI_ISL_8921333, EPI_ISL_8921336, EPI_ISL_8921337, EPI_ISL_8921342, EPI_ISL_8921344, EPI_ISL_8921346, EPI_ISL_8921350, EPI_ISL_8921358, EPI_ISL_8921363, EPI_ISL_8921368, EPI_ISL_8921377, EPI_ISL_8921387, EPI_ISL_8921388, EPI_ISL_8921390, EPI_ISL_8921392, EPI_ISL_8921396, EPI_ISL_8921399, EPI_ISL_8921400, EPI_ISL_8921402, EPI_ISL_8921406, EPI_ISL_8921412, EPI_ISL_8921426, EPI_ISL_8921430, EPI_ISL_8921431, EPI_ISL_8921438, EPI_ISL_8921442, EPI_ISL_8921445, EPI_ISL_8921449, EPI_ISL_8921451, EPI_ISL_8921453, EPI_ISL_8921455, EPI_ISL_8921462, EPI_ISL_8921465, EPI_ISL_8921466, EPI_ISL_8921467, EPI_ISL_8921472, EPI_ISL_8921477, EPI_ISL_8921486, EPI_ISL_8921490, EPI_ISL_8921492, EPI_ISL_8921494, EPI_ISL_8921496, EPI_ISL_8921498, EPI_ISL_8921505, EPI_ISL_8921509, EPI_ISL_8921515, EPI_ISL_8921517, EPI_ISL_8921519, EPI_ISL_8921521, EPI_ISL_8921527, EPI_ISL_8921534, EPI_ISL_8921536, EPI_ISL_8921539, EPI_ISL_8921543, EPI_ISL_8921551, EPI_ISL_8921557, EPI_ISL_8921560, EPI_ISL_8921564, EPI_ISL_8921570, EPI_ISL_8921577, EPI_ISL_8921579, EPI_ISL_8921585, EPI_ISL_8921594, EPI_ISL_8921599, EPI_ISL_8921601, EPI_ISL_8921605, EPI_ISL_8921607, EPI_ISL_8921611, EPI_ISL_8921613, EPI_ISL_8921620, EPI_ISL_8921624, EPI_ISL_8921628, EPI_ISL_8921630, EPI_ISL_8921634, EPI_ISL_8921638, EPI_ISL_8921645, EPI_ISL_8921648, EPI_ISL_8921650, EPI_ISL_8921655, EPI_ISL_8921657, EPI_ISL_8921662, EPI_ISL_8921666, EPI_ISL_8921674, EPI_ISL_8921678, EPI_ISL_8921680, EPI_ISL_8921682, EPI_ISL_8921692, EPI_ISL_8921696, EPI_ISL_8921698, EPI_ISL_8921699, EPI_ISL_8921701, EPI_ISL_8921705, EPI_ISL_8921713, EPI_ISL_8921727, EPI_ISL_8921730, EPI_ISL_8921736, EPI_ISL_8921738, EPI_ISL_8921740, EPI_ISL_8921747, EPI_ISL_8921749, EPI_ISL_8921757, EPI_ISL_8921762, EPI_ISL_8921764, EPI_ISL_8921770, EPI_ISL_8921772, EPI_ISL_8921777, EPI_ISL_8921781, EPI_ISL_8921783, EPI_ISL_8921787, EPI_ISL_8921791, EPI_ISL_8921798, EPI_ISL_8921800, EPI_ISL_8921802, EPI_ISL_8921820, EPI_ISL_8921821, EPI_ISL_8921823, EPI_ISL_8921825, EPI_ISL_8921829, EPI_ISL_8921831, EPI_ISL_8921834, EPI_ISL_8921836, EPI_ISL_8921839, EPI_ISL_8921844, EPI_ISL_8921849, EPI_ISL_8921863, EPI_ISL_8921878, EPI_ISL_8921880, EPI_ISL_8921884, EPI_ISL_8921885, EPI_ISL_8921887, EPI_ISL_8921889, EPI_ISL_8921891, EPI_ISL_8921893, EPI_ISL_8921895, EPI_ISL_8921901, EPI_ISL_8921907, EPI_ISL_8921909, EPI_ISL_8921913, EPI_ISL_8921916, EPI_ISL_8921918, EPI_ISL_8921920, EPI_ISL_8921922, EPI_ISL_8921923, EPI_ISL_8921925, EPI_ISL_8921927, EPI_ISL_8921939, EPI_ISL_8921941, EPI_ISL_8921949, EPI_ISL_8921950, EPI_ISL_8921954, EPI_ISL_8921957, EPI_ISL_8921958, EPI_ISL_8921960, EPI_ISL_8921964, EPI_ISL_8921966, EPI_ISL_8921970, EPI_ISL_8921974, EPI_ISL_8921981, EPI_ISL_8921988, EPI_ISL_8921990, EPI_ISL_8921992, EPI_ISL_8922001, EPI_ISL_8922011, EPI_ISL_8922015, EPI_ISL_8922017, EPI_ISL_8922027, EPI_ISL_8922030, EPI_ISL_8922032, EPI_ISL_8922034, EPI_ISL_8922036, EPI_ISL_8922038, EPI_ISL_8922043, EPI_ISL_8922047, EPI_ISL_8922049, EPI_ISL_8922051, EPI_ISL_8922059, EPI_ISL_8922060, EPI_ISL_8922064, EPI_ISL_8922070, EPI_ISL_8922076, EPI_ISL_8922087, EPI_ISL_8922089, EPI_ISL_8922091, EPI_ISL_8922093, EPI_ISL_8922096, EPI_ISL_8922098, EPI_ISL_8922102, EPI_ISL_8922104, EPI_ISL_8922106, EPI_ISL_8922113, EPI_ISL_8922115, EPI_ISL_8922117, EPI_ISL_8922121, EPI_ISL_8922123, EPI_ISL_8922126, EPI_ISL_8922130, EPI_ISL_8922134, EPI_ISL_8922143, EPI_ISL_8922147, EPI_ISL_8922149, EPI_ISL_8922151, EPI_ISL_8922156, EPI_ISL_8922170, EPI_ISL_8922177, EPI_ISL_8922182, EPI_ISL_8922185, EPI_ISL_8922190, EPI_ISL_8922195, EPI_ISL_8922199, EPI_ISL_8922202, EPI_ISL_8922206, EPI_ISL_8922221, EPI_ISL_8922223, EPI_ISL_8922225, EPI_ISL_8922233, EPI_ISL_8922237, EPI_ISL_8922239, EPI_ISL_8922243, EPI_ISL_8922245, EPI_ISL_8922247, EPI_ISL_8922250, EPI_ISL_8922254, EPI_ISL_8922258, EPI_ISL_8922265, EPI_ISL_8922267, EPI_ISL_8922269, EPI_ISL_8922286, EPI_ISL_8922290, EPI_ISL_8922292, EPI_ISL_8922302, EPI_ISL_8922304, EPI_ISL_8922314, EPI_ISL_8922316, EPI_ISL_8922318, EPI_ISL_8922319, EPI_ISL_8922321, EPI_ISL_8922327, EPI_ISL_8922329, EPI_ISL_8922331, EPI_ISL_8922336, EPI_ISL_8922338, EPI_ISL_8922344, EPI_ISL_8922350, EPI_ISL_8922352, EPI_ISL_8922354, EPI_ISL_8922356, EPI_ISL_8922357, EPI_ISL_8922369, EPI_ISL_8922372, EPI_ISL_8922377, EPI_ISL_8922379, EPI_ISL_8922384, EPI_ISL_8922387, EPI_ISL_8922389, EPI_ISL_8922391, EPI_ISL_8922396, EPI_ISL_8922397, EPI_ISL_8922399, EPI_ISL_8922403, EPI_ISL_8922405, EPI_ISL_8922412, EPI_ISL_8922414, EPI_ISL_8922418, EPI_ISL_8922419, EPI_ISL_8922421, EPI_ISL_8922423, EPI_ISL_8922435, EPI_ISL_8922439, EPI_ISL_8922445, EPI_ISL_8922447, EPI_ISL_8922450, EPI_ISL_8922458, EPI_ISL_8922464, EPI_ISL_8922469, EPI_ISL_8922471, EPI_ISL_8922475, EPI_ISL_8922481, EPI_ISL_8922482, EPI_ISL_8922488, EPI_ISL_8922489, EPI_ISL_8922491, EPI_ISL_8922507, EPI_ISL_8922516, EPI_ISL_8922523, EPI_ISL_8922533, EPI_ISL_8922701, EPI_ISL_8922703, EPI_ISL_8922705, EPI_ISL_8922711, EPI_ISL_8922713, EPI_ISL_8922717, EPI_ISL_8922721, EPI_ISL_8922726, EPI_ISL_8922728, EPI_ISL_8922734, EPI_ISL_8922737, EPI_ISL_8922741, EPI_ISL_8922743, EPI_ISL_8922745, EPI_ISL_8922749, EPI_ISL_8922751, EPI_ISL_8922767, EPI_ISL_8922770, EPI_ISL_8922772, EPI_ISL_8922777, EPI_ISL_8922783, EPI_ISL_8922785, EPI_ISL_8922787, EPI_ISL_8922790, EPI_ISL_8922798, EPI_ISL_8922799, EPI_ISL_8922801, EPI_ISL_8922805, EPI_ISL_8922808, EPI_ISL_8922810, EPI_ISL_8922816, EPI_ISL_8922817, EPI_ISL_8922822, EPI_ISL_8922830, EPI_ISL_8922847, EPI_ISL_8922851, EPI_ISL_8922853, EPI_ISL_8922855, EPI_ISL_8922856, EPI_ISL_8922860, EPI_ISL_8922862, EPI_ISL_8922864, EPI_ISL_8922866, EPI_ISL_8922868, EPI_ISL_8922877, EPI_ISL_8922885, EPI_ISL_8922888, EPI_ISL_8922890, EPI_ISL_8922892, EPI_ISL_8922895, EPI_ISL_8922897, EPI_ISL_8922918, EPI_ISL_8922922, EPI_ISL_8922923, EPI_ISL_8922927, EPI_ISL_8922928, EPI_ISL_8922930, EPI_ISL_8922934, EPI_ISL_8922949, EPI_ISL_8922954, EPI_ISL_8922956, EPI_ISL_8922961, EPI_ISL_8922963, EPI_ISL_8922968, EPI_ISL_8922970, EPI_ISL_8922972, EPI_ISL_8922976, EPI_ISL_8922980, EPI_ISL_8922982, EPI_ISL_8922987, EPI_ISL_8922991, EPI_ISL_8922992, EPI_ISL_8922997, EPI_ISL_8922998, EPI_ISL_8923000, EPI_ISL_8923006, EPI_ISL_8923008, EPI_ISL_8923012, EPI_ISL_8923014, EPI_ISL_8923016, EPI_ISL_8923018, EPI_ISL_8923023, EPI_ISL_8923027, EPI_ISL_8923033, EPI_ISL_8923042, EPI_ISL_8923048, EPI_ISL_8923085, EPI_ISL_8923087, EPI_ISL_8923089, EPI_ISL_8923091, EPI_ISL_8923093, EPI_ISL_8923101, EPI_ISL_8923104, EPI_ISL_8923109, EPI_ISL_8923116, EPI_ISL_8923125, EPI_ISL_8923127, EPI_ISL_8923133, EPI_ISL_8923155, EPI_ISL_8923165, EPI_ISL_8923169, EPI_ISL_8923170, EPI_ISL_8923174, EPI_ISL_8923403			
see above	Department of Bacteria, Parasites and Fungi, Statens Serum Institut, Copenhagen, Denmark	Statens Serum Institut Bioinformatics and Microbial Genomics	Danish Covid-19 Genome Consortium
EPI_ISL_8920923	INSPI-CRN DE INFLUENZA Y OTROS VIRUS RESPIRATORIOS	INSPI-CRN DE INFLUENZA Y OTROS VIRUS RESPIRATORIOS	Alfredo Bruno; Diego Morales; Fernando Llerena; Jimmy Garcés; Maritza Olmedo; Michelle Páez; Ruben Armas
EPI_ISL_8919813, EPI_ISL_8919825, EPI_ISL_8919846, EPI_ISL_8919893, EPI_ISL_8919903, EPI_ISL_8919904, EPI_ISL_8919906, EPI_ISL_8919907, EPI_ISL_8919908, EPI_ISL_8919923, EPI_ISL_8919959, EPI_ISL_8919960, EPI_ISL_8919961, EPI_ISL_8919971, EPI_ISL_8919978, EPI_ISL_8920033, EPI_ISL_8920034, EPI_ISL_8920036, EPI_ISL_8920041, EPI_ISL_8920044, EPI_ISL_8920063, EPI_ISL_8920064, EPI_ISL_8920070, EPI_ISL_8920071, EPI_ISL_8920072, EPI_ISL_8920120, EPI_ISL_8920130, EPI_ISL_8920131, EPI_ISL_8920132, EPI_ISL_8920133, EPI_ISL_8920143, EPI_ISL_8920144, EPI_ISL_8920233, EPI_ISL_8920236, EPI_ISL_8920252, EPI_ISL_8920266, EPI_ISL_8920267, EPI_ISL_8920268, EPI_ISL_8920269, EPI_ISL_8920296, EPI_ISL_8920297, EPI_ISL_8920298, EPI_ISL_8920320, EPI_ISL_8920334, EPI_ISL_8920345, EPI_ISL_8920346, EPI_ISL_8920356, EPI_ISL_8920359, EPI_ISL_8920362, EPI_ISL_8920366, EPI_ISL_8920367, EPI_ISL_8920389, EPI_ISL_8920393, EPI_ISL_8920394, EPI_ISL_8920407, EPI_ISL_8920415, EPI_ISL_8920419, EPI_ISL_8920420, EPI_ISL_8920421, EPI_ISL_8920438			
see above	SARS-CoV-2 testing team, National Institute of Infectious Diseases	Pathogen Genomics Center, National Institute of Infectious Diseases	Hazuka Y Furihata; Kentaro Itokawa; Makoto Kuroda; Masanori Hashino; Masumichi Saito; Naomi Nojiri; Nozomu Hanaoka; Rina Tanaka; Tsuguto Fujimoto; Tsuyoshi Sekizuka
EPI_ISL_8919712, EPI_ISL_8919721, EPI_ISL_8919724	Tokyo Metropolitan Institute of Public Health	Tokyo Metropolitan Institute of Public Health	Akane Negishi; Arisa Amano; Hirofumi Miyake; Jun Suzuki; Kenji Sadamasu; Kenshirou Kuroki; Mami Nagashima; Masaki Hayashi; Maya Isogai; Takako Yamazaki; Takushi Fujiwara

We gratefully acknowledge the following Authors from the Originating laboratories responsible for obtaining the specimens, as well as the Submitting laboratories where the genome data were generated and shared via GISAID, on which this research is based.

All Submitters of data may be contacted directly via www.gisaid.org

Authors are sorted alphabetically.

Accession ID	Originating Laboratory	Submitting Laboratory	Authors
EPI_ISL_8934094	ASST GOM NIGUARDA	ASST Grande ospedale Metropolitano Niguarda	Alice Nava
EPI_ISL_8943096, EPI_ISL_8947554, EPI_ISL_8947588, EPI_ISL_8950543, EPI_ISL_8950554, EPI_ISL_8950562, EPI_ISL_8950655, EPI_ISL_8951105, EPI_ISL_8951290			
see above	Aegis Sciences Corporation	Centers for Disease Control and Prevention Division of Viral Diseases, Pathogen Discovery	Alec Vest; Benjamin Rambo-Martin; Christopher Gulvick; Clinton Paden; Cyndi Clark; Dakota Howard; Dhwani Batra; Dillon Nall; Duncan MacCannell; Erisa Sula; Ethan Sanders; Holly Houdeshell; Jason Caravas; Kristine Lacek; Matthew Hardison; Matthew Schmerer; Ola Kvalvaag; Patrick Campbell; Peter Cook; Rob Case; Scott Sammons; Shatavia Morrison; Shaun Westlund; Tymeckia Kendall; Victoria Caban Figueroa; Vikramsinha Ghorpade; Yvette Unoarumhi
EPI_ISL_8935629, EPI_ISL_8935630, EPI_ISL_8935631, EPI_ISL_8935632	CDPH VBL	California Department of Public Health	CDPH-COVIDNet
EPI_ISL_8931581, EPI_ISL_8931603, EPI_ISL_8931629	Cliniques universitaires Saint-Luc	UCLouvain/IREC/MBLG-CTMA	Benoit Kabamba Mukadi; Bertrand Bearzatto; Jean-Luc Gala; Nicolas Pinte; Paul Blanpain; Simon Ophélie; Valentin Coste
EPI_ISL_8953795, EPI_ISL_8953796, EPI_ISL_8953801	Colorado Department of Public Health and Environment	Colorado Department of Public Health and Environment	Alexandria Rossheim; Arianna Smith; Diana Ir; Emily A. Travanty; Laura Bankers; Mandy Waters; Michael Martin; Molly C. Hetherington-Rauth; Shannon R. Matzinger
EPI_ISL_8929317, EPI_ISL_8929318, EPI_ISL_8929319, EPI_ISL_8929320, EPI_ISL_8929321, EPI_ISL_8929322	Division of Emerging Infectious Diseases, Bureau of Infectious Diseases Diagnosis Control, Korea Disease Control and Prevention Agency	Division of Emerging Infectious Diseases, Bureau of Infectious Diseases Diagnosis Control, Korea Disease Control and Prevention Agency	Ae Kyung Park; Chae Young Lee; Eun-Jin Kim; Hyuck Jin Lee; Il-Hwan Kim; Jeong-Ah Kim
EPI_ISL_8938767, EPI_ISL_8940304	Fulgent Genetics	Centers for Disease Control and Prevention Division of Viral Diseases, Pathogen Discovery	Becky Tsai; Benafsh Sapra; Benjamin Rambo-Martin; Christopher Gulvick; Clinton Paden; Dakota Howard; Dhwani Batra; Doreen Ng; Duncan MacCannell; Erisa Sula; Harry Gao; James Xie; Jason Caravas; John Gao; Joseph Fierro; Kristine Lacek; Matthew Schmerer; Mickey Li; Peter Cook; Scott Sammons; Shatavia Morrison; Tymeckia Kendall; Victoria Caban Figueroa; Yan Meng; Yvette Unoarumhi
EPI_ISL_8947237	Infinity Biologix	Centers for Disease Control and Prevention Division of Viral Diseases, Pathogen Discovery	Benjamin Rambo-Martin; Chirayu Goswami; Christian Bixby; Christopher Gulvick; Clinton Paden; Dakota Howard; Dhwani Batra; Duncan MacCannell; Erisa Sula; Jason Caravas; Jonathan Schultz; Kristine Lacek; Matthew Schmerer; Peter Cook; Robin Grimwood; Russ Hager; Scott Sammons; Shatavia Morrison; Tymeckia Kendall; Victoria Caban Figueroa; Yihe Wang; Yvette Unoarumhi
EPI_ISL_8938300	Laboratorio Central, Ministerio de Salud Córdoba	Fundacion para el Progreso de la Medicina and Instituto de Patologia Vegetal (IPAVE-CIAP-INTA) on behalf of 'Proyecto Argentino Interinstitucional de genomica de SARS-CoV-2' (PAIS Consortium)	Barbas, G.; Castro, G.; Debat, H.; F.D.; Fernández; Goya, S.; Lucca, A.; M.B.; Marquez, N.; Pisano; Re, V.; Sicilia, P.; Zeballos, M.
EPI_ISL_8953561	Laboratorio De Patología Molecular Secretaria De Marina Centro Medico Naval	Laboratorio De Patología Molecular Secretaria De Marina Centro Medico Naval	Morales Ramirez Omar. Baltierra Uribe Shantall. Meixueiro Calderon Claudia. Garcia Alegria Irma. Zuno Coronado Teutli. Alvarez Jimenez Violeta Dejanira. Cabrera Gonzalez Moises. Romero Espejel Maria Elena. Mora Heredia Jose Eduardo. Rubio Ortiz Margarita
EPI_ISL_8953052	Mako Medical	Centers for Disease Control and Prevention Division of Viral Diseases, Pathogen Discovery	Benjamin Rambo-Martin; Christopher Gulvick; Clinton Paden; Dakota Howard; Dhwani Batra; Duncan MacCannell; Erisa Sula; Jason Caravas; Kristine Lacek; Lauren Moon; Matthew Schmerer; Matthew Tugwell; Peter Cook; Scott Sammons; Shatavia Morrison; Tymeckia Kendall; Victoria Caban Figueroa; Yvette Unoarumhi
EPI_ISL_8929501, EPI_ISL_8929502	Ministry of Health Turkey	Ministry of Health Turkey	Fatma Bayrakdar; Gülay Korukluoğlu; Gültekin Ünal; Süleyman Yalcin; Yasemin Coşgun
EPI_ISL_8937373	PathWest Laboratory Medicine WA	PathWest Laboratory Medicine WA Microbial Surveillance Unit	PathWest Laboratory Medicine WA Microbial Surveillance Unit
EPI_ISL_8931071	Polski Bank Komórek Macierzystych	WSSE w Warszawie	Dorota Wagrocka - Roczniak
EPI_ISL_8946339	Rosalind Franklin Laboratory	Wellcome Sanger Institute for the COVID-19 Genomics UK (COG-UK) Consortium	Cordelia Langford; David K. Jackson; Dominic Kwiatkowski; Donald Fraser; Ewan Harrison; Ian Johnston; Jeffrey Barrett; John Sillitoe on behalf of the Wellcome Sanger Institute COVID-19 Surveillance Team; Rob Howes; Roberto Amato; Sonia Goncalves; Suki Lee; The Rosalind Franklin Laboratory and Alex Alderton
EPI_ISL_8953837, EPI_ISL_8953838	Stanford Health Care	Stanford University School of Medicine, Clinical Virology Laboratory	Daniel Solis; Fumiko Yamamoto; James Zehnder; Malaya K. Sahoo; Mamdouh Sibai; Selamawit Bihon; Suzette Santos; and Benjamin A. Pinsky
EPI_ISL_8936681	Tokyo Metropolitan Institute of Public Health	Tokyo Metropolitan Institute of Public Health	Akane Negishi; Arisa Amano; Hirofumi Miyake; Jun Suzuki; Kenji Sadamasu; Kenshirou Kuroki; Mami Nagashima; Masaki Hayashi; Maya Isogai; Takako Yamazaki; Takushi Fujiwara
EPI_ISL_8937981, EPI_ISL_8937989, EPI_ISL_8938000, EPI_ISL_8938010, EPI_ISL_8938015	URMC LABS	URMC LABS	Andrew Cameron; Dwight Hardy; Kelly DeLary; Stepan Voljehn
EPI_ISL_8950784, EPI_ISL_8952751	Utah Public Health Laboratory	Utah Public Health Laboratory	Erin L. Young; John Arnn; Kelly F. Oakeson; Olinto Linares-Perdomo; Pooja Gupta; Tom Iverson
EPI_ISL_8933676	Vibhavadi Hospital	Center for Medical Genomics Ramathibodi Hospital	Anek mungaomklang; Insee Sensorn; Inthuorn Ratchatasetthakul; Kanyarat Janthima; Parsakorn Tapaopong; Poramate Jiaranai; Vichapon Tiacharoen; Wasun Chantratita
EPI_ISL_8938156, EPI_ISL_8938170	Wyoming Public Health Laboratory	Wyoming Public Health Laboratory	Ashley Norberg; Brian Dominguez; Cari Sloma; Channing Weber; Chayse Rowley; Elliot Thomasson; Jim Mildenberger; Marley Goetz; Robert Petit; Sam Britz; Taylor Fearing; and Rob Christensen

We gratefully acknowledge the following Authors from the Originating laboratories responsible for obtaining the specimens, as well as the Submitting laboratories where the genome data were generated and shared via GISAID, on which this research is based.

All Submitters of data may be contacted directly via www.gisaid.org

Authors are sorted alphabetically.

Acknowledgement EPI_SET Identifier: EPI_SET_20220527nw

Accession ID	Originating Laboratory	Submitting Laboratory	Authors
EPI_ISL_8928232, EPI_ISL_8928234, EPI_ISL_8928236, EPI_ISL_8928240, EPI_ISL_8928248, EPI_ISL_8928250, EPI_ISL_8928255, EPI_ISL_8928257, EPI_ISL_8928262, EPI_ISL_8928264, EPI_ISL_8928266, EPI_ISL_8928276, EPI_ISL_8928282, EPI_ISL_8928284, EPI_ISL_8928285, EPI_ISL_8928287, EPI_ISL_8928289, EPI_ISL_8928293, EPI_ISL_8928296, EPI_ISL_8928304, EPI_ISL_8928307, EPI_ISL_8928309			
see above	Department of Bacteria, Parasites and Fungi, Statens Serum Institut, Copenhagen, Denmark	Statens Serum Institut Bioinformatics and Microbial Genomics	Danish Covid-19 Genome Consortium
EPI_ISL_8929304, EPI_ISL_8929305, EPI_ISL_8929306, EPI_ISL_8929307, EPI_ISL_8929308, EPI_ISL_8929309, EPI_ISL_8929310, EPI_ISL_8929311, EPI_ISL_8929312, EPI_ISL_8929313, EPI_ISL_8929314, EPI_ISL_8929315, EPI_ISL_8929316			
see above	Division of Emerging Infectious Diseases, Bureau of Infectious Diseases Diagnosis Control, Korea Disease Control and Prevention Agency	Division of Emerging Infectious Diseases, Bureau of Infectious Diseases Diagnosis Control, Korea Disease Control and Prevention Agency	Ae Kyung Park; Chae Young Lee; Eun-Jin Kim; Hyuck Jin Lee; Il-Hwan Kim; Jeong-Ah Kim
EPI_ISL_8929209, EPI_ISL_8929225	Dutch COVID-19 response team	Medical Microbiology, Maastricht University Medical Centre	Brian van der Veer*; Carmen Reumkens; Christian Hoebe; Erik Beuken; Jozef Dingemans*; Lieke van Alphen; Paul Savelkoul
EPI_ISL_8928516, EPI_ISL_8928517, EPI_ISL_8928518, EPI_ISL_8928519, EPI_ISL_8928529, EPI_ISL_8928530	Furst Medical Laboratory	Norwegian Institute of Public Health, Department of Virology	Atiya R Ali; Debech Nadia; Engebretsen Serina Beate; Garcia Llorente Ignacio; Hilde Elshaug; Hilde Vollan; Jon Bråte; Kamilla Heddeland Instefjord; Karoline Bragstad; Kathrine Stene-Johansen; Line Victoria Moen; Marie Paulsen Madsen; Olav Hungnes; Pedersen Benedikte Nevjen; Rasmus Riis Kopperud
EPI_ISL_8928326, EPI_ISL_8928388, EPI_ISL_8928431, EPI_ISL_8928434, EPI_ISL_8928436, EPI_ISL_8928460	Oslo University Hospital, Department of Microbiology	Norwegian Institute of Public Health, Department of Virology	Arvind Yegambaram Meenakshi Sundaram; Cathrine Fladeby; Garcia Llorente Ignacio; Gregor D. Gilfillan; Hilde Elshaug; Hilde Vollan; Jon Bråte; Kamilla Heddeland Instefjord; Karoline Bragstad; Kathrine Stene-Johansen; Line Victoria Moen; Lise Andresen; Mariann Nilsen; Mona Holberg-Petersen; Olav Hungnes; Pedersen Benedikte Nevjen; Pål Marius Bjørnstad; Rasmus Riis Kopperud; Teodora Plamenova Ribarska
EPI_ISL_8928596	Polski Bank Komórek Macierzystych	WSSE w Warszawie	Dorota Wagrocka - Roczniak

We gratefully acknowledge the following Authors from the Originating laboratories responsible for obtaining the specimens, as well as the Submitting laboratories where the genome data were generated and shared via GISAID, on which this research is based.

All Submitters of data may be contacted directly via www.gisaid.org

Authors are sorted alphabetically.

Accession ID	Originating Laboratory	Submitting Laboratory	Authors
EPI_ISL_8927645, EPI_ISL_8927647, EPI_ISL_8927649, EPI_ISL_8927652, EPI_ISL_8927756, EPI_ISL_8927765, EPI_ISL_8927769, EPI_ISL_8927778, EPI_ISL_8927780, EPI_ISL_8927787, EPI_ISL_8927789, EPI_ISL_8927801, EPI_ISL_8927803, EPI_ISL_8927806, EPI_ISL_8927812, EPI_ISL_8927823, EPI_ISL_8927935, EPI_ISL_8928098, EPI_ISL_8928101, EPI_ISL_8928107, EPI_ISL_8928113, EPI_ISL_8928119, EPI_ISL_8928126, EPI_ISL_8928130, EPI_ISL_8928132, EPI_ISL_8928136, EPI_ISL_8928142, EPI_ISL_8928148, EPI_ISL_8928155, EPI_ISL_8928163, EPI_ISL_8928165, EPI_ISL_8928172, EPI_ISL_8928174, EPI_ISL_8928176, EPI_ISL_8928177, EPI_ISL_8928179, EPI_ISL_8928183, EPI_ISL_8928185, EPI_ISL_8928188, EPI_ISL_8928190, EPI_ISL_8928192, EPI_ISL_8928200, EPI_ISL_8928202, EPI_ISL_8928204, EPI_ISL_8928208, EPI_ISL_8928212, EPI_ISL_8928227, EPI_ISL_8928230			
see above	Department of Bacteria, Parasites and Fungi, Statens Serum Institut, Copenhagen, Denmark	Statens Serum Institut Bioinformatics and Microbial Genomics	Danish Covid-19 Genome Consortium
EPI_ISL_8928084	Medical Microbiology Unit, Department for Laboratory Medicine, Drammen Hospital, Vestre Viken Health Trust	Norwegian Institute of Public Health, Department of Virology	Atiya R Ali; Debech Nadia; Engebretsen Serina Beate; Garcia Llorente Ignacio; Hilde Elshaug; Hilde Vollan; Jon Bråte; Kamilla Heddeland Instefjord; Karoline Bragstad; Kathrine Stene-Johansen; Line Victoria Moen; Marie Paulsen Madsen; Olav Hungnes; Pedersen Benedikte Nevjen; Rasmus Riis Kopperud
EPI_ISL_8928077	Ostfold Hospital Trust - Kalnes, Centre for Laboratory Medicine, Section for gene technology and infection serology	Norwegian Institute of Public Health, Department of Virology	Atiya R Ali; Debech Nadia; Engebretsen Serina Beate; Garcia Llorente Ignacio; Hilde Elshaug; Hilde Vollan; Jon Bråte; Kamilla Heddeland Instefjord; Karoline Bragstad; Kathrine Stene-Johansen; Line Victoria Moen; Marie Paulsen Madsen; Olav Hungnes; Pedersen Benedikte Nevjen; Rasmus Riis Kopperud

We gratefully acknowledge the following Authors from the Originating laboratories responsible for obtaining the specimens, as well as the Submitting laboratories where the genome data were generated and shared via GISAID, on which this research is based.

All Submitters of data may be contacted directly via www.gisaid.org

Authors are sorted alphabetically.

Accession ID	Originating Laboratory	Submitting Laboratory	Authors
EPI_ISL_8927387, EPI_ISL_8927390, EPI_ISL_8927405, EPI_ISL_8927423, EPI_ISL_8927431, EPI_ISL_8927435, EPI_ISL_8927438, EPI_ISL_8927442, EPI_ISL_8927445, EPI_ISL_8927454, EPI_ISL_8927456, EPI_ISL_8927465, EPI_ISL_8927467, EPI_ISL_8927473, EPI_ISL_8927478, EPI_ISL_8927491, EPI_ISL_8927498, EPI_ISL_8927505, EPI_ISL_8927507, EPI_ISL_8927515, EPI_ISL_8927517, EPI_ISL_8927519, EPI_ISL_8927521, EPI_ISL_8927523, EPI_ISL_8927527, EPI_ISL_8927528, EPI_ISL_8927537, EPI_ISL_8927542, EPI_ISL_8927544, EPI_ISL_8927545, EPI_ISL_8927550, EPI_ISL_8927553, EPI_ISL_8927555, EPI_ISL_8927557, EPI_ISL_8927562, EPI_ISL_8927564, EPI_ISL_8927567, EPI_ISL_8927575, EPI_ISL_8927578, EPI_ISL_8927583, EPI_ISL_8927584, EPI_ISL_8927590, EPI_ISL_8927597, EPI_ISL_8927605, EPI_ISL_8927627, EPI_ISL_8927629, EPI_ISL_8927631, EPI_ISL_8927632, EPI_ISL_8927636, EPI_ISL_8927640			
see above	Department of Bacteria, Parasites and Fungi, Statens Serum Institut, Copenhagen, Denmark	Statens Serum Institut Bioinformatics and Microbial Genomics	Danish Covid-19 Genome Consortium

We gratefully acknowledge the following Authors from the Originating laboratories responsible for obtaining the specimens, as well as the Submitting laboratories where the genome data were generated and shared via GISAID, on which this research is based.

All Submitters of data may be contacted directly via www.gisaid.org

Authors are sorted alphabetically.

Acknowledgement EPI_SET Identifier: EPI_SET_20220527yz

Accession ID	Originating Laboratory	Submitting Laboratory	Authors
EPI_ISL_8927302, EPI_ISL_8927322, EPI_ISL_8927325, EPI_ISL_8927334, EPI_ISL_8927362, EPI_ISL_8927364, EPI_ISL_8927373			
see above	Department of Bacteria, Parasites and Fungi, Statens Serum Institut, Copenhagen, Denmark	Statens Serum Institut Bioinformatics and Microbial Genomics	Danish Covid-19 Genome Consortium
EPI_ISL_8927251, EPI_ISL_8927252, EPI_ISL_8927253, EPI_ISL_8927254, EPI_ISL_8927255, EPI_ISL_8927256, EPI_ISL_8927257, EPI_ISL_8927258, EPI_ISL_8927259, EPI_ISL_8927261, EPI_ISL_8927262, EPI_ISL_8927263, EPI_ISL_8927264, EPI_ISL_8927265, EPI_ISL_8927266, EPI_ISL_8927267, EPI_ISL_8927268, EPI_ISL_8927269, EPI_ISL_8927270, EPI_ISL_8927271, EPI_ISL_8927272, EPI_ISL_8927273, EPI_ISL_8927274, EPI_ISL_8927275, EPI_ISL_8927276, EPI_ISL_8927277, EPI_ISL_8927278, EPI_ISL_8927279, EPI_ISL_8927280, EPI_ISL_8927281, EPI_ISL_8927282, EPI_ISL_8927283, EPI_ISL_8927284, EPI_ISL_8927285, EPI_ISL_8927286, EPI_ISL_8927287, EPI_ISL_8927288, EPI_ISL_8927289, EPI_ISL_8927290, EPI_ISL_8927291, EPI_ISL_8927292, EPI_ISL_8927293, EPI_ISL_8927295			
see above	National Public Health Laboratory, National Centre for Infectious Diseases	National Public Health Laboratory, National Centre for Infectious Diseases	Benny Yeo; Grace Ngan; Lin Cui; Raymond Tzer Pin Lin; Royce Ang; Samuel Loo; Yichen Ding; Zhenyang Zhou

We gratefully acknowledge the following Authors from the Originating laboratories responsible for obtaining the specimens, as well as the Submitting laboratories where the genome data were generated and shared via GISAID, on which this research is based.

All Submitters of data may be contacted directly via www.gisaid.org

Authors are sorted alphabetically.

Accession ID	Originating Laboratory	Submitting Laboratory	Authors
EPI_ISL_8927026, EPI_ISL_8927027, EPI_ISL_8927030, EPI_ISL_8927033, EPI_ISL_8927036, EPI_ISL_8927040, EPI_ISL_8927069, EPI_ISL_8927072, EPI_ISL_8927075, EPI_ISL_8927082, EPI_ISL_8927086, EPI_ISL_8927096, EPI_ISL_8927103, EPI_ISL_8927107, EPI_ISL_8927119, EPI_ISL_8927122, EPI_ISL_8927125, EPI_ISL_8927209			
see above	Department of Bacteria, Parasites and Fungi, Statens Serum Institut, Copenhagen, Denmark	Statens Serum Institut Bioinformatics and Microbial Genomics	Danish Covid-19 Genome Consortium
EPI_ISL_8927219, EPI_ISL_8927220, EPI_ISL_8927221, EPI_ISL_8927222, EPI_ISL_8927223, EPI_ISL_8927224, EPI_ISL_8927225, EPI_ISL_8927226, EPI_ISL_8927227, EPI_ISL_8927228, EPI_ISL_8927229, EPI_ISL_8927230, EPI_ISL_8927231, EPI_ISL_8927232, EPI_ISL_8927233, EPI_ISL_8927234, EPI_ISL_8927235, EPI_ISL_8927236, EPI_ISL_8927237, EPI_ISL_8927238, EPI_ISL_8927239, EPI_ISL_8927240, EPI_ISL_8927241, EPI_ISL_8927242, EPI_ISL_8927243, EPI_ISL_8927244, EPI_ISL_8927245, EPI_ISL_8927246, EPI_ISL_8927247, EPI_ISL_8927248, EPI_ISL_8927249, EPI_ISL_8927250			
see above	National Public Health Laboratory, National Centre for Infectious Diseases	National Public Health Laboratory, National Centre for Infectious Diseases	Benny Yeo; Grace Ngan; Lin Cui; Raymond Tzer Pin Lin; Royce Ang; Samuel Loo; Yichen Ding; Zhenyang Zhou

We gratefully acknowledge the following Authors from the Originating laboratories responsible for obtaining the specimens, as well as the Submitting laboratories where the genome data were generated and shared via GISAID, on which this research is based.

All Submitters of data may be contacted directly via www.gisaid.org

Authors are sorted alphabetically.

Accession ID	Originating Laboratory	Submitting Laboratory	Authors
EPI_ISL_8926743, EPI_ISL_8926745, EPI_ISL_8926747, EPI_ISL_8926749, EPI_ISL_8926751, EPI_ISL_8926759, EPI_ISL_8926764, EPI_ISL_8926773, EPI_ISL_8926776, EPI_ISL_8926780, EPI_ISL_8926783, EPI_ISL_8926786, EPI_ISL_8926793, EPI_ISL_8926797, EPI_ISL_8926802, EPI_ISL_8926805, EPI_ISL_8926808, EPI_ISL_8926811, EPI_ISL_8926813, EPI_ISL_8926816, EPI_ISL_8926818, EPI_ISL_8926821, EPI_ISL_8926828, EPI_ISL_8926838, EPI_ISL_8926842, EPI_ISL_8926843, EPI_ISL_8926849, EPI_ISL_8926857, EPI_ISL_8926861, EPI_ISL_8926868, EPI_ISL_8926877, EPI_ISL_8926879, EPI_ISL_8926881, EPI_ISL_8926888, EPI_ISL_8926890, EPI_ISL_8926893, EPI_ISL_8926896, EPI_ISL_8926904, EPI_ISL_8926908, EPI_ISL_8926910, EPI_ISL_8926914, EPI_ISL_8926923, EPI_ISL_8926931, EPI_ISL_8926945, EPI_ISL_8926993, EPI_ISL_8927003, EPI_ISL_8927010, EPI_ISL_8927014, EPI_ISL_8927018, EPI_ISL_8927025			
see above	Department of Bacteria, Parasites and Fungi, Statens Serum Institut, Copenhagen, Denmark	Statens Serum Institut Bioinformatics and Microbial Genomics	Danish Covid-19 Genome Consortium

We gratefully acknowledge the following Authors from the Originating laboratories responsible for obtaining the specimens, as well as the Submitting laboratories where the genome data were generated and shared via GISAID, on which this research is based.

All Submitters of data may be contacted directly via www.gisaid.org

Authors are sorted alphabetically.

Acknowledgement EPI_SET Identifier: EPI_SET_20220527mz

Accession ID	Originating Laboratory	Submitting Laboratory	Authors
EPI_ISL_8926265, EPI_ISL_8926269, EPI_ISL_8926277, EPI_ISL_8926295, EPI_ISL_8926307, EPI_ISL_8926316, EPI_ISL_8926318, EPI_ISL_8926326, EPI_ISL_8926328, EPI_ISL_8926329, EPI_ISL_8926330, EPI_ISL_8926332, EPI_ISL_8926336, EPI_ISL_8926340, EPI_ISL_8926345, EPI_ISL_8926351, EPI_ISL_8926360, EPI_ISL_8926362, EPI_ISL_8926367, EPI_ISL_8926369, EPI_ISL_8926373, EPI_ISL_8926390, EPI_ISL_8926391, EPI_ISL_8926394, EPI_ISL_8926401, EPI_ISL_8926403, EPI_ISL_8926408, EPI_ISL_8926410, EPI_ISL_8926415, EPI_ISL_8926416, EPI_ISL_8926576, EPI_ISL_8926579, EPI_ISL_8926585, EPI_ISL_8926596, EPI_ISL_8926598, EPI_ISL_8926601, EPI_ISL_8926604, EPI_ISL_8926605, EPI_ISL_8926608, EPI_ISL_8926611, EPI_ISL_8926613, EPI_ISL_8926615, EPI_ISL_8926617, EPI_ISL_8926621, EPI_ISL_8926627, EPI_ISL_8926633, EPI_ISL_8926634, EPI_ISL_8926637, EPI_ISL_8926640, EPI_ISL_8926740			
see above	Department of Bacteria, Parasites and Fungi, Statens Serum Institut, Copenhagen, Denmark	Statens Serum Institut Bioinformatics and Microbial Genomics	Danish Covid-19 Genome Consortium

We gratefully acknowledge the following Authors from the Originating laboratories responsible for obtaining the specimens, as well as the Submitting laboratories where the genome data were generated and shared via GISAID, on which this research is based.

All Submitters of data may be contacted directly via www.gisaid.org

Authors are sorted alphabetically.

Accession ID	Originating Laboratory	Submitting Laboratory	Authors
EPI_ISL_8925531, EPI_ISL_8925532, EPI_ISL_8925533, EPI_ISL_8925534, EPI_ISL_8925541, EPI_ISL_8925542, EPI_ISL_8925547, EPI_ISL_8925553, EPI_ISL_8925566, EPI_ISL_8925568, EPI_ISL_8925570, EPI_ISL_8925574, EPI_ISL_8925581, EPI_ISL_8925585, EPI_ISL_8925587, EPI_ISL_8925589, EPI_ISL_8925592, EPI_ISL_8925594, EPI_ISL_8925596, EPI_ISL_8925600, EPI_ISL_8925602, EPI_ISL_8925611, EPI_ISL_8925637, EPI_ISL_8925639, EPI_ISL_8925648, EPI_ISL_8925650, EPI_ISL_8925651, EPI_ISL_8925652, EPI_ISL_8925653, EPI_ISL_8925654, EPI_ISL_8925655, EPI_ISL_8925656, EPI_ISL_8925657, EPI_ISL_8925658, EPI_ISL_8925659, EPI_ISL_8925660, EPI_ISL_8925661, EPI_ISL_8925662, EPI_ISL_8925670, EPI_ISL_8925681, EPI_ISL_8925685, EPI_ISL_8925686, EPI_ISL_8926037, EPI_ISL_8926246, EPI_ISL_8926249, EPI_ISL_8926251, EPI_ISL_8926255, EPI_ISL_8926259, EPI_ISL_8926263			
see above	Department of Bacteria, Parasites and Fungi, Statens Serum Institut, Copenhagen, Denmark	Statens Serum Institut Bioinformatics and Microbial Genomics	Danish Covid-19 Genome Consortium
EPI_ISL_8925674	Unipath Speciality Laboratory Limited, Ahmedabad	Gujarat Biotechnology Research Centre	Akhilesh Modi; Apurvasinh Puvar; Bhadreshsinh Gohil; Chaitanya Joshi; Disha Vora; Janvi Raval; Jaykumar Rangani; Jwalant Shah; Madhvi Joshi; Nimesh Patel; Nitin Savaliya; NitinShukla; Priyank Chavda; Ramesh Pandit; Roshani Mishra; Sonal Sharma; Tasnim Trivedi; Zarna Patel

We gratefully acknowledge the following Authors from the Originating laboratories responsible for obtaining the specimens, as well as the Submitting laboratories where the genome data were generated and shared via GISAID, on which this research is based.

All Submitters of data may be contacted directly via www.gisaid.org

Authors are sorted alphabetically.

Acknowledgement EPI_SET Identifier: EPI_SET_20220527wc

Accession ID	Originating Laboratory	Submitting Laboratory	Authors
EPI_ISL_8925264, EPI_ISL_8925266, EPI_ISL_8925267, EPI_ISL_8925272, EPI_ISL_8925273, EPI_ISL_8925276, EPI_ISL_8925277, EPI_ISL_8925295, EPI_ISL_8925298, EPI_ISL_8925299, EPI_ISL_8925301, EPI_ISL_8925302, EPI_ISL_8925303, EPI_ISL_8925304, EPI_ISL_8925307, EPI_ISL_8925312, EPI_ISL_8925316, EPI_ISL_8925318, EPI_ISL_8925320, EPI_ISL_8925322, EPI_ISL_8925324, EPI_ISL_8925327, EPI_ISL_8925331, EPI_ISL_8925366, EPI_ISL_8925429, EPI_ISL_8925431, EPI_ISL_8925434, EPI_ISL_8925436, EPI_ISL_8925440, EPI_ISL_8925446, EPI_ISL_8925448, EPI_ISL_8925458, EPI_ISL_8925460, EPI_ISL_8925462, EPI_ISL_8925471, EPI_ISL_8925478, EPI_ISL_8925483, EPI_ISL_8925485, EPI_ISL_8925486, EPI_ISL_8925488, EPI_ISL_8925494, EPI_ISL_8925500, EPI_ISL_8925501, EPI_ISL_8925503, EPI_ISL_8925507, EPI_ISL_8925513, EPI_ISL_8925519, EPI_ISL_8925523, EPI_ISL_8925527, EPI_ISL_8925529			
see above	Department of Bacteria, Parasites and Fungi, Statens Serum Institut, Copenhagen, Denmark	Statens Serum Institut Bioinformatics and Microbial Genomics	Danish Covid-19 Genome Consortium

We gratefully acknowledge the following Authors from the Originating laboratories responsible for obtaining the specimens, as well as the Submitting laboratories where the genome data were generated and shared via GISAID, on which this research is based.

All Submitters of data may be contacted directly via www.gisaid.org

Authors are sorted alphabetically.

Acknowledgement EPI_SET Identifier: EPI_SET_20220527kz

Accession ID	Originating Laboratory	Submitting Laboratory	Authors
EPI_ISL_8925102, EPI_ISL_8925104, EPI_ISL_8925106, EPI_ISL_8925107, EPI_ISL_8925109, EPI_ISL_8925117, EPI_ISL_8925118, EPI_ISL_8925120, EPI_ISL_8925126, EPI_ISL_8925127, EPI_ISL_8925131, EPI_ISL_8925133, EPI_ISL_8925136, EPI_ISL_8925144, EPI_ISL_8925146, EPI_ISL_8925155, EPI_ISL_8925163, EPI_ISL_8925167, EPI_ISL_8925169, EPI_ISL_8925172, EPI_ISL_8925174, EPI_ISL_8925176, EPI_ISL_8925178, EPI_ISL_8925180, EPI_ISL_8925181, EPI_ISL_8925185, EPI_ISL_8925186, EPI_ISL_8925222, EPI_ISL_8925223, EPI_ISL_8925224, EPI_ISL_8925225, EPI_ISL_8925227, EPI_ISL_8925230, EPI_ISL_8925232, EPI_ISL_8925235, EPI_ISL_8925236, EPI_ISL_8925239, EPI_ISL_8925240, EPI_ISL_8925243, EPI_ISL_8925244, EPI_ISL_8925247, EPI_ISL_8925248, EPI_ISL_8925249, EPI_ISL_8925250, EPI_ISL_8925251, EPI_ISL_8925252, EPI_ISL_8925259, EPI_ISL_8925260, EPI_ISL_8925261, EPI_ISL_8925262			
see above	Department of Bacteria, Parasites and Fungi, Statens Serum Institut, Copenhagen, Denmark	Statens Serum Institut Bioinformatics and Microbial Genomics	Danish Covid-19 Genome Consortium

We gratefully acknowledge the following Authors from the Originating laboratories responsible for obtaining the specimens, as well as the Submitting laboratories where the genome data were generated and shared via GISAID, on which this research is based.

All Submitters of data may be contacted directly via www.gisaid.org

Authors are sorted alphabetically.

Accession ID	Originating Laboratory	Submitting Laboratory	Authors
EPI_ISL_8924860, EPI_ISL_8924861, EPI_ISL_8924867, EPI_ISL_8924868, EPI_ISL_8924869, EPI_ISL_8924870, EPI_ISL_8924872, EPI_ISL_8924873, EPI_ISL_8924876, EPI_ISL_8924880, EPI_ISL_8924881, EPI_ISL_8924883, EPI_ISL_8924884, EPI_ISL_8924890, EPI_ISL_8924891, EPI_ISL_8924899, EPI_ISL_8924905, EPI_ISL_8924911, EPI_ISL_8924921, EPI_ISL_8924924, EPI_ISL_8924926, EPI_ISL_8924973, EPI_ISL_8924975, EPI_ISL_8924978, EPI_ISL_8924982, EPI_ISL_8924986, EPI_ISL_8924991, EPI_ISL_8924993, EPI_ISL_8924994, EPI_ISL_8924996, EPI_ISL_8924999, EPI_ISL_8925007, EPI_ISL_8925012, EPI_ISL_8925016, EPI_ISL_8925018, EPI_ISL_8925022, EPI_ISL_8925026, EPI_ISL_8925027, EPI_ISL_8925030, EPI_ISL_8925039, EPI_ISL_8925043, EPI_ISL_8925049, EPI_ISL_8925051, EPI_ISL_8925055, EPI_ISL_8925057, EPI_ISL_8925083, EPI_ISL_8925085, EPI_ISL_8925089, EPI_ISL_8925098, EPI_ISL_8925100			
see above	Department of Bacteria, Parasites and Fungi, Statens Serum Institut, Copenhagen, Denmark	Statens Serum Institut Bioinformatics and Microbial Genomics	Danish Covid-19 Genome Consortium

We gratefully acknowledge the following Authors from the Originating laboratories responsible for obtaining the specimens, as well as the Submitting laboratories where the genome data were generated and shared via GISAID, on which this research is based.

All Submitters of data may be contacted directly via www.gisaid.org

Authors are sorted alphabetically.

Acknowledgement EPI_SET Identifier: EPI_SET_20220527ua

EPI_ISL_8923793, EPI_ISL_8923797, EPI_ISL_8923802, EPI_ISL_8923804, EPI_ISL_8923806, EPI_ISL_8923812, EPI_ISL_8923815, EPI_ISL_8923817, EPI_ISL_8923833, EPI_ISL_8923835, EPI_ISL_8923838, EPI_ISL_8923843, EPI_ISL_8923845, EPI_ISL_8923846, EPI_ISL_8923848, EPI_ISL_8923854, EPI_ISL_8923869, EPI_ISL_8923872, EPI_ISL_8923877, EPI_ISL_8923881, EPI_ISL_8923886, EPI_ISL_8923897, EPI_ISL_8923899, EPI_ISL_8923901, EPI_ISL_8923907, EPI_ISL_8923908, EPI_ISL_8923910, EPI_ISL_8923914, EPI_ISL_8923916, EPI_ISL_8923922, EPI_ISL_8923924, EPI_ISL_8923926, EPI_ISL_8923928, EPI_ISL_8923934, EPI_ISL_8923941, EPI_ISL_8923942, EPI_ISL_8924574, EPI_ISL_8924578, EPI_ISL_8924584, EPI_ISL_8924586, EPI_ISL_8924600, EPI_ISL_8924607, EPI_ISL_8924610, EPI_ISL_8924622, EPI_ISL_8924625, EPI_ISL_8924630, EPI_ISL_8924699, EPI_ISL_8924723, EPI_ISL_8924857, EPI_ISL_8924859

Accession ID	Originating Laboratory	Submitting Laboratory	Authors
see above	Department of Bacteria, Parasites and Fungi, Statens Serum Institut, Copenhagen, Denmark	Statens Serum Institut Bioinformatics and Microbial Genomics	Danish Covid-19 Genome Consortium

We gratefully acknowledge the following Authors from the Originating laboratories responsible for obtaining the specimens, as well as the Submitting laboratories where the genome data were generated and shared via GISAID, on which this research is based.

All Submitters of data may be contacted directly via www.gisaid.org

Authors are sorted alphabetically.

Accession ID	Originating Laboratory	Submitting Laboratory	Authors
EPI_ISL_8923653, EPI_ISL_8923665, EPI_ISL_8923666, EPI_ISL_8923674, EPI_ISL_8923675, EPI_ISL_8923676, EPI_ISL_8923678, EPI_ISL_8923680, EPI_ISL_8923682, EPI_ISL_8923683, EPI_ISL_8923685, EPI_ISL_8923687, EPI_ISL_8923688, EPI_ISL_8923694, EPI_ISL_8923700, EPI_ISL_8923702, EPI_ISL_8923707, EPI_ISL_8923708, EPI_ISL_8923710, EPI_ISL_8923714, EPI_ISL_8923716, EPI_ISL_8923718, EPI_ISL_8923720, EPI_ISL_8923722, EPI_ISL_8923724, EPI_ISL_8923727, EPI_ISL_8923728, EPI_ISL_8923730, EPI_ISL_8923732, EPI_ISL_8923734, EPI_ISL_8923739, EPI_ISL_8923742, EPI_ISL_8923746, EPI_ISL_8923749, EPI_ISL_8923753, EPI_ISL_8923755, EPI_ISL_8923757, EPI_ISL_8923759, EPI_ISL_8923761, EPI_ISL_8923763, EPI_ISL_8923765, EPI_ISL_8923767, EPI_ISL_8923769, EPI_ISL_8923771, EPI_ISL_8923774, EPI_ISL_8923778, EPI_ISL_8923780, EPI_ISL_8923784, EPI_ISL_8923786, EPI_ISL_8923791			
see above	Department of Bacteria, Parasites and Fungi, Statens Serum Institut, Copenhagen, Denmark	Statens Serum Institut Bioinformatics and Microbial Genomics	Danish Covid-19 Genome Consortium

We gratefully acknowledge the following Authors from the Originating laboratories responsible for obtaining the specimens, as well as the Submitting laboratories where the genome data were generated and shared via GISAID, on which this research is based.

All Submitters of data may be contacted directly via www.gisaid.org

Authors are sorted alphabetically.

Acknowledgement EPI_SET Identifier: EPI_SET_20220527nr

Accession ID	Originating Laboratory	Submitting Laboratory	Authors
EPI_ISL_8923434, EPI_ISL_8923436, EPI_ISL_8923438, EPI_ISL_8923441, EPI_ISL_8923443, EPI_ISL_8923445, EPI_ISL_8923455, EPI_ISL_8923464, EPI_ISL_8923467, EPI_ISL_8923469, EPI_ISL_8923471, EPI_ISL_8923473, EPI_ISL_8923475, EPI_ISL_8923477, EPI_ISL_8923478, EPI_ISL_8923486, EPI_ISL_8923487, EPI_ISL_8923489, EPI_ISL_8923491, EPI_ISL_8923493, EPI_ISL_8923494, EPI_ISL_8923496, EPI_ISL_8923498, EPI_ISL_8923507, EPI_ISL_8923509, EPI_ISL_8923514, EPI_ISL_8923516, EPI_ISL_8923523, EPI_ISL_8923524, EPI_ISL_8923525, EPI_ISL_8923526, EPI_ISL_8923539, EPI_ISL_8923542, EPI_ISL_8923543, EPI_ISL_8923546, EPI_ISL_8923548, EPI_ISL_8923550, EPI_ISL_8923560, EPI_ISL_8923583, EPI_ISL_8923621, EPI_ISL_8923625, EPI_ISL_8923626, EPI_ISL_8923628, EPI_ISL_8923630, EPI_ISL_8923634, EPI_ISL_8923636, EPI_ISL_8923638, EPI_ISL_8923644, EPI_ISL_8923646, EPI_ISL_8923651			
see above	Department of Bacteria, Parasites and Fungi, Statens Serum Institut, Copenhagen, Denmark	Statens Serum Institut Bioinformatics and Microbial Genomics	Danish Covid-19 Genome Consortium

We gratefully acknowledge the following Authors from the Originating laboratories responsible for obtaining the specimens, as well as the Submitting laboratories where the genome data were generated and shared via GISAID, on which this research is based.

All Submitters of data may be contacted directly via www.gisaid.org

Authors are sorted alphabetically.

Accession ID	Originating Laboratory	Submitting Laboratory	Authors
EPI_ISL_8923177, EPI_ISL_8923181, EPI_ISL_8923186, EPI_ISL_8923190, EPI_ISL_8923195, EPI_ISL_8923203, EPI_ISL_8923204, EPI_ISL_8923211, EPI_ISL_8923212, EPI_ISL_8923214, EPI_ISL_8923216, EPI_ISL_8923251, EPI_ISL_8923255, EPI_ISL_8923259, EPI_ISL_8923260, EPI_ISL_8923263, EPI_ISL_8923264, EPI_ISL_8923266, EPI_ISL_8923271, EPI_ISL_8923279, EPI_ISL_8923283, EPI_ISL_8923285, EPI_ISL_8923294, EPI_ISL_8923296, EPI_ISL_8923298, EPI_ISL_8923303, EPI_ISL_8923305, EPI_ISL_8923307, EPI_ISL_8923310, EPI_ISL_8923314, EPI_ISL_8923322, EPI_ISL_8923329, EPI_ISL_8923331, EPI_ISL_8923333, EPI_ISL_8923335, EPI_ISL_8923345, EPI_ISL_8923348, EPI_ISL_8923352, EPI_ISL_8923360, EPI_ISL_8923361, EPI_ISL_8923363, EPI_ISL_8923365, EPI_ISL_8923375, EPI_ISL_8923384, EPI_ISL_8923388, EPI_ISL_8923390, EPI_ISL_8923393, EPI_ISL_8923399, EPI_ISL_8923403, EPI_ISL_8923412			
see above	Department of Bacteria, Parasites and Fungi, Statens Serum Institut, Copenhagen, Denmark	Statens Serum Institut Bioinformatics and Microbial Genomics	Danish Covid-19 Genome Consortium

We gratefully acknowledge the following Authors from the Originating laboratories responsible for obtaining the specimens, as well as the Submitting laboratories where the genome data were generated and shared via GISAID, on which this research is based.

All Submitters of data may be contacted directly via www.gisaid.org

Authors are sorted alphabetically.

Acknowledgement EPI_SET Identifier: EPI_SET_20220527pn

Accession ID	Originating Laboratory	Submitting Laboratory	Authors
EPI_ISL_9300753	ABC Algarve	Instituto Nacional de Saude (INSA)	Borges et al
EPI_ISL_9285973	Allergy, Immunology and Cell Biology Unit (AICBU)	Allergy, Immunology and Cell Biology Unit (AICBU)	Ayesha Wijesinghe; Chandima Jeewandara; Deshni Jayathilaka; Dinuka Ariyaratne; Diyanath Ranasinghe; Dumni Guasinghe; Farha Bary; Gathsaurie Neelika Malavige; Heshan Kuruppu; Tibutius Thanesh
EPI_ISL_9300776	Avelab	Instituto Nacional de Saude (INSA)	Borges et al
EPI_ISL_9301587, EPI_ISL_9301588	Betong hospital	Prince of Songkhla University	Anusara Wongkotsila; Chanon Kongkamon; Pongsakorn Choochuen; Samonrapat Surasombatpattana; Sarunyou Chusri; Surakameth Mahasirimongkol; Surasak Sangkhathat; Thammasin Ingviya; Thanit Sila; Virasakdi Chongsuvivatwong; Wison Laochareonsuk
EPI_ISL_9300755	CHTMAD - Vila Real	Instituto Nacional de Saude (INSA)	Borges et al
EPI_ISL_9289788, EPI_ISL_9289789, EPI_ISL_9289790, EPI_ISL_9289795, EPI_ISL_9289798, EPI_ISL_9289806, EPI_ISL_9289823, EPI_ISL_9289824, EPI_ISL_9289829, EPI_ISL_9289831, EPI_ISL_9289836, EPI_ISL_9289841, EPI_ISL_9289842			
see above	CSIR-NEERI, Nagpur Covid-19 Testing Lab	CSIR-NEERI, Nagpur Covid-19 Testing Lab	Krishna Khairnar et al.
EPI_ISL_9300754	H Santarem	Instituto Nacional de Saude (INSA)	Borges et al
EPI_ISL_9287725, EPI_ISL_9287732	Hamera Laboratorium	National Institute of Health Research and Development	Arie Ardiansyah Nugraha; Fajar Nur Sulistiyohadi; Hana Apsari Pawestri; Hartanti Dian Ikawati; Kartika Dewi Puspa; Nelly Puspandari; Nur Ika Hariastuti; Putri Widia; Subangkit; Vivi Setiawaty
EPI_ISL_9287822, EPI_ISL_9287824, EPI_ISL_9287828	Kantor Kesehatan Pelabuhan Kelas II Balikpapan	National Institute of Health Research and Development	Arie Ardiansyah Nugraha; Fajar Nur Sulistiyohadi; Hana Apsari Pawestri; Hartanti Dian Ikawati; Kartika Dewi Puspa; Nelly Puspandari; Nur Ika Hariastuti; Puri Widia; Subangkit; Vivi Setiawaty
EPI_ISL_9293265	Lighthouse Lab in Alderley Park	Wellcome Sanger Institute for the COVID-19 Genomics UK (COG-UK) Consortium	Cordelia Langford; David K. Jackson; Dominic Kwiatkowski; Ewan Harrison; Ian Johnston; Jacquelyn Wynn; Jeffrey Barrett; John Sillitoe on behalf of the Wellcome Sanger Institute COVID-19 Surveillance Team; Mairead Hyland; Roberto Amato; Sonia Goncalves; The Lighthouse Lab in Alderley Park and Alex Alderton
EPI_ISL_9293266	Lighthouse Lab in Glasgow	Wellcome Sanger Institute for the COVID-19 Genomics UK (COG-UK) Consortium	Anna Dominiczak and Alex Alderton; Carol Clugston; Cordelia Langford; David Gray; David K. Jackson; Dominic Kwiatkowski; Ewan Harrison; Harper VanSteenhouse; Ian Johnston; Jeffrey Barrett; John Sillitoe on behalf of the Wellcome Sanger Institute COVID-19 Surveillance Team; Roberto Amato; Sonia Goncalves; Yumi Kasai
EPI_ISL_9302406, EPI_ISL_9302879, EPI_ISL_9303021, EPI_ISL_9303065	Microbiological Diagnostic Unit - Public Health Laboratory (MDU-PHL)	Microbiological Diagnostic Unit - Public Health Laboratory (MDU-PHL)	Horan, K.; N.L.; Seemann, T.; Sherry
EPI_ISL_9301675	N Health Hat Yai	Prince of Songkhla University	Anusara Wongkotsila; Chanon Kongkamon; Pongsakorn Choochuen; Samonrapat Surasombatpattana; Sarunyou Chusri; Surakameth Mahasirimongkol; Surasak Sangkhathat; Thammasin Ingviya; Thanit Sila; Virasakdi Chongsuvivatwong; Wison Laochareonsuk
EPI_ISL_9301625, EPI_ISL_9301626, EPI_ISL_9301627, EPI_ISL_9301629, EPI_ISL_9301630	PR Medical Laboratory.	Prince of Songkhla University	Anusara Wongkotsila; Chanon Kongkamon; Pongsakorn Choochuen; Samonrapat Surasombatpattana; Sarunyou Chusri; Surakameth Mahasirimongkol; Surasak Sangkhathat; Thammasin Ingviya; Thanit Sila; Virasakdi Chongsuvivatwong; Wison Laochareonsuk
EPI_ISL_9301585, EPI_ISL_9301586	Pattani Hospital	Prince of Songkhla University	Anusara Wongkotsila; Chanon Kongkamon; Pongsakorn Choochuen; Samonrapat Surasombatpattana; Sarunyou Chusri; Surakameth Mahasirimongkol; Surasak Sangkhathat; Thammasin Ingviya; Thanit Sila; Virasakdi Chongsuvivatwong; Wison Laochareonsuk
EPI_ISL_9301589, EPI_ISL_9301590, EPI_ISL_9301591, EPI_ISL_9301593, EPI_ISL_9301595, EPI_ISL_9301631, EPI_ISL_9301632			
see above	RAJYINDEE Hospital	Prince of Songkhla University	Anusara Wongkotsila; Chanon Kongkamon; Pongsakorn Choochuen; Samonrapat Surasombatpattana; Sarunyou Chusri; Surakameth Mahasirimongkol; Surasak Sangkhathat; Thammasin Ingviya; Thanit Sila; Virasakdi Chongsuvivatwong; Wison Laochareonsuk
EPI_ISL_9287781	RS Kartika Pulomas	National Institute of Health Research and Development	Arie Ardiansyah Nugraha; Fajar Nur Sulistiyohadi; Hana Apsari Pawestri; Hartanti Dian Ikawati; Kartika Dewi Puspa; Nelly Puspandari; Nur Ika Hariastuti; Putri Widia; Subangkit; Vivi Setiawaty
EPI_ISL_9287817	RSUP Persahabatan	National Institute of Health Research and Development	Arie Ardiansyah Nugraha; Fajar Nur Sulistiyohadi; Hana Apsari Pawestri; Hartanti Dian Ikawati; Kartika Dewi Puspa; Nelly Puspandari; Nur Ika Hariastuti; Putri Widia; Subangkit; Vivi Setiawaty
EPI_ISL_9301655, EPI_ISL_9301656	Rattaphum Hospital	Prince of Songkhla University	Anusara Wongkotsila; Chanon Kongkamon; Pongsakorn Choochuen; Samonrapat Surasombatpattana; Sarunyou Chusri; Surakameth Mahasirimongkol; Surasak Sangkhathat; Thammasin Ingviya; Thanit Sila; Virasakdi Chongsuvivatwong; Wison Laochareonsuk
EPI_ISL_9290286, EPI_ISL_9290322, EPI_ISL_9293221	Respiratory Virus Unit, Microbiology Services Colindale, Public Health England	COVID-19 Genomics UK (COG-UK) Consortium	PHE Covid Sequencing Team
EPI_ISL_9298655	Rumah Sakit Royal Taruma	Genomik Solidaritas Indonesia Laboratorium	Annisa Muthiah Sukirman; Anuraj Shankar; Ariel Pradipta; Carissa Sintca Wijaya; Gracia Felias Enos Korompis; Liman; Louisa Markus; Meutia Ayuputeri Kumaheri; Reinhart Gabriel; Riana; Stefanus Bernard
EPI_ISL_9301652, EPI_ISL_9301653, EPI_ISL_9301654	SOMDEJPRABOROMRACHINEENART NATAWEE HOSPITAL.	Prince of Songkhla University	Anusara Wongkotsila; Chanon Kongkamon; Pongsakorn Choochuen; Samonrapat Surasombatpattana; Sarunyou Chusri; Surakameth Mahasirimongkol; Surasak Sangkhathat; Thammasin Ingviya; Thanit Sila; Virasakdi Chongsuvivatwong; Wison Laochareonsuk
EPI_ISL_9301597	Sikarin Hospital	Prince of Songkhla University	Anusara Wongkotsila; Chanon Kongkamon; Pongsakorn Choochuen; Samonrapat Surasombatpattana; Sarunyou Chusri; Surakameth Mahasirimongkol; Surasak Sangkhathat; Thammasin Ingviya; Thanit Sila; Virasakdi Chongsuvivatwong; Wison Laochareonsuk
EPI_ISL_9301640, EPI_ISL_9301641, EPI_ISL_9301642, EPI_ISL_9301643, EPI_ISL_9301644	Siroros Hospital	Prince of Songkhla University	Anusara Wongkotsila; Chanon Kongkamon; Pongsakorn Choochuen; Samonrapat Surasombatpattana; Sarunyou Chusri; Surakameth Mahasirimongkol; Surasak Sangkhathat; Thammasin Ingviya; Thanit Sila; Virasakdi Chongsuvivatwong; Wison Laochareonsuk
EPI_ISL_9301610, EPI_ISL_9301611, EPI_ISL_9301612, EPI_ISL_9301613, EPI_ISL_9301614, EPI_ISL_9301615, EPI_ISL_9301618, EPI_ISL_9301620, EPI_ISL_9301622, EPI_ISL_9301623			
see above	Songkhla Hospital	Prince of Songkhla University	Anusara Wongkotsila; Chanon Kongkamon; Pongsakorn Choochuen; Samonrapat Surasombatpattana; Sarunyou Chusri; Surakameth Mahasirimongkol; Surasak Sangkhathat; Thammasin Ingviya; Thanit Sila; Virasakdi Chongsuvivatwong; Wison Laochareonsuk
EPI_ISL_9301687, EPI_ISL_9301688, EPI_ISL_9301689, EPI_ISL_9301690, EPI_ISL_9301694	Songklanagarind hospital	Prince of Songkhla University	Anusara Wongkotsila; Chanon Kongkamon; Pongsakorn Choochuen; Samonrapat Surasombatpattana; Sarunyou Chusri; Surakameth Mahasirimongkol; Surasak Sangkhathat; Thammasin Ingviya; Thanit Sila; Virasakdi Chongsuvivatwong; Wison Laochareonsuk
EPI_ISL_9301598, EPI_ISL_9301599, EPI_ISL_9301600, EPI_ISL_9301601, EPI_ISL_9301602, EPI_ISL_9301603, EPI_ISL_9301604, EPI_ISL_9301605, EPI_ISL_9301607, EPI_ISL_9301658, EPI_ISL_9301659, EPI_ISL_9301661, EPI_ISL_9301662, EPI_ISL_9301663			
see above	Su-ngai Kolok Hospital	Prince of Songkhla University	Anusara Wongkotsila; Chanon Kongkamon; Pongsakorn Choochuen; Samonrapat Surasombatpattana; Sarunyou Chusri; Surakameth Mahasirimongkol; Surasak Sangkhathat; Thammasin Ingviya; Thanit Sila; Virasakdi Chongsuvivatwong; Wison Laochareonsuk
EPI_ISL_9300770	ULSNE - Braganca	Instituto Nacional de Saude (INSA)	Borges et al
EPI_ISL_9301868, EPI_ISL_9301875, EPI_ISL_9301881, EPI_ISL_9301903	UW Virology Lab	UW Virology Lab	Alexander Greninger; Hong Xie; Isabel Arnould; Keith R Jerome; Meei-Li Huang; Pavitra Roychoudhury; Pooneh Hajian; Ricardo Perez; Saraswathi Sathees; Sean Ellis; Seffir T. Wendm; Shah Mohamed Bakhash

We gratefully acknowledge the following Authors from the Originating laboratories responsible for obtaining the specimens, as well as the Submitting laboratories where the genome data were generated and shared via GISAID, on which this research is based.

All Submitters of data may be contacted directly via www.gisaid.org

Authors are sorted alphabetically.

Accession ID	Originating Laboratory	Submitting Laboratory	Authors
EPI_ISL_9324346, EPI_ISL_9324347, EPI_ISL_9324348	AIRPORT CLINIC	inStem, NCBS-INSACOG	Aswin SaiNarain; Dasaradhi Palakodeti; Uma Ramakrishnan
EPI_ISL_9324342, EPI_ISL_9324344	ASTER LABS SARJAPUR ROAD	inStem, NCBS-INSACOG	Aswin SaiNarain; Dasaradhi Palakodeti; Uma Ramakrishnan
EPI_ISL_9324340	ASTER R V	inStem, NCBS-INSACOG	Aswin SaiNarain; Dasaradhi Palakodeti; Uma Ramakrishnan
EPI_ISL_9331874, EPI_ISL_9332007	Aegis Sciences Corporation	Centers for Disease Control and Prevention Division of Viral Diseases, Pathogen Discovery	Alec Vest; Benjamin Rambo-Martin; Christopher Gulvick; Clinton Paden; Cyndi Clark; Dakota Howard; Dhwani Batra; Dillon Nall; Duncan MacCannell; Erisa Sula; Ethan Sanders; Holly Houdeshell; Jason Caravas; Kristine Lacek; Matthew Hardison; Matthew Schmerer; Ola Kvalvaag; Patrick Campbell; Peter Cook; Rob Case; Scott Sammons; Shatavia Morrison; Shaun Westlund; Tymeckia Kendall; Victoria Caban Figueroa; Vikramsinha Ghorpade; Yvette Unoarumhi
EPI_ISL_9329641, EPI_ISL_9329651, EPI_ISL_9329672, EPI_ISL_9329675, EPI_ISL_9329790, EPI_ISL_9329812, EPI_ISL_9329821, EPI_ISL_9329827, EPI_ISL_9329839, EPI_ISL_9329850, EPI_ISL_9329858, EPI_ISL_9329862, EPI_ISL_9329863, EPI_ISL_9329864, EPI_ISL_9329865, EPI_ISL_9329870, EPI_ISL_9329874, EPI_ISL_9329880			
see above	BANGALORE BAPTIST HOSPITAL	National Center for Biological Sciences, TIFR - Rockefeller Foundation	Awadhesh Pandit; Bhagyashree Madhav Shelar; Darshan Sreenivas; Dimple Notani; Kathirvel Kandasamy; Lakshminarayanan CP; Satyajit Mayor; Uma Ramakrishnan
EPI_ISL_9336257, EPI_ISL_9336265, EPI_ISL_9336266, EPI_ISL_9336279, EPI_ISL_9336284, EPI_ISL_9336285, EPI_ISL_9336286, EPI_ISL_9336297, EPI_ISL_9336298, EPI_ISL_9336302, EPI_ISL_9336307, EPI_ISL_9336311, EPI_ISL_9336323, EPI_ISL_9336324, EPI_ISL_9336325, EPI_ISL_9336326, EPI_ISL_9336327, EPI_ISL_9336332, EPI_ISL_9336333, EPI_ISL_9336335, EPI_ISL_9336337			
see above	CSIR-NEERI, Nagpur Covid-19 Testing Lab	CSIR-NEERI, Nagpur Covid-19 Testing Lab	Krishna Khairnar et al.
EPI_ISL_9324334	DASARHALLI FPEC ASTER	inStem, NCBS-INSACOG	Aswin SaiNarain; Dasaradhi Palakodeti; Uma Ramakrishnan
EPI_ISL_9325605	Department of Microbiology, University Hospital in Pilsen	Department of Microbiology, University Hospital in Pilsen	Jaroslav Hrabak; Katerina Chudejova; Katerina Vlkova; Petra Kocova; Vendula Heringova
EPI_ISL_9332008	Fulgent Genetics	Centers for Disease Control and Prevention Division of Viral Diseases, Pathogen Discovery	Becky Tsai; Benafsh Sapra; Benjamin Rambo-Martin; Christopher Gulvick; Clinton Paden; Dakota Howard; Dhwani Batra; Doreen Ng; Duncan MacCannell; Erisa Sula; Harry Gao; James Xie; Jason Caravas; John Gao; Joseph Fierro; Kristine Lacek; Matthew Schmerer; Mickey Li; Peter Cook; Scott Sammons; Shatavia Morrison; Tymeckia Kendall; Victoria Caban Figueroa; Yan Meng; Yvette Unoarumhi
EPI_ISL_9324317, EPI_ISL_9324321	HOME COLLECTION	inStem, NCBS-INSACOG	Aswin SaiNarain; Dasaradhi Palakodeti; Uma Ramakrishnan
EPI_ISL_9325921	Hospital Santa Bárbara	Hospital General Universitario de Ciudad Real	Cristina Colmenarejo; José Martínez-Alarcón; Lidia García-Agudo; Marta Torres-Narbona; Soledad Illescas Fernández-Bermejo
EPI_ISL_9347062	Houston Methodist Hospital	Houston Methodist Hospital	Akanksha Batajoo; Ilya J. Finkelstein; James J. Davis; Jessica Cambric; Jimmy Gollihar; Kristina Reppond; Madison N. Shyer; Marcus Nguyen; Matthew Ojeda Saavedra; Paul A. Christensen; Randall J. Olsen; Rashi Thakur; Regan Mangham; Richard Snehall; Robert Olson; Ryan Gadd; S. Wesley Long; Sindy Pena; and James M. Musser
EPI_ISL_9322394	Hrvatski zavod za javno zdravstvo	Hrvatski zavod za javno zdravstvo	Anita Jurić; Dragan Jurić; Irena Tabain; Ivana Ferenčak; Josipa Kuzle
EPI_ISL_9325315	Invenimus AG	Institute of Medical Virology, University of Zurich	Alexandra Trkola; Annette Audigé; Catharine Aquino; Cyril Shah; Daniel Ehrsam; Gabriela Ziltener; Guido Bloemberg; Hubert Rehrauer; Isabel Stürmer; Joel Wirz; Jon Huder; Jürg Böni; Kevin Steiner; Maria Grünberg; Maryam Zaheri; Michael Huber; Riccarda Capaul; Stefan Schmutz; Verena Kufner; Weihong Qi
EPI_ISL_9306076	Israel Central Virology laboratory	Israel Central Virology laboratory	Danit Sofer; Efrat Dahan Bucris; Efrat Glick-Saar; Ella Mendelson; Gideon Rechavi; Hagar Morad; Michal Mandelboim; Miranda Geva; Neta Zuckerman; Or Zilbertzan; Oran Erster; Orna Mor
EPI_ISL_9306388	Jerusalem Central Laboratories	Israel Central Virology laboratory	Danit Sofer; Efrat Dahan Bucris; Ella Mendelson; Evan Nachum; Hagar Morad; Julia Vainer; Maya Davidovich; Michal Mandelboim; Michal Zak; Miranda Geva; Neta Zuckerman; Or Zilbertzan; Oran Erster; Orna Mor; Rona Grossman
EPI_ISL_9325288	Kantonsspital Aarau	Institute of Medical Virology, University of Zurich	Alexandra Trkola; Annette Audigé; Catharine Aquino; Cyril Shah; Daniel Ehrsam; Gabriela Ziltener; Guido Bloemberg; Hubert Rehrauer; Isabel Stürmer; Joel Wirz; Jon Huder; Jürg Böni; Kevin Steiner; Maria Grünberg; Maryam Zaheri; Michael Huber; Riccarda Capaul; Stefan Schmutz; Verena Kufner; Weihong Qi
EPI_ISL_9324292, EPI_ISL_9324293	Kims Hubli	inStem, NCBS-INSACOG	Aswin SaiNarain; Dasaradhi Palakodeti; Uma Ramakrishnan
EPI_ISL_9344681	LABORATORIO DE FRANCA - FRANCA - 2064154	Instituto Butantan	Antonio Jorge Martins; Claudia Renata dos Santos Barros; David Schlesinger; Debora Botequio Moretti; Dimas Tadeu Covas; Elaine Cristina Marqueze; Elaine Vieira Santos; Evandra Strazza Rodrigues; Heidge Fukumasu; Jayme Augusto de Souza-Neto; José Salvatore Leister Patané; Luiz Alcantara; Luiz Lehmann Coutinho; Maria Carolina Elias; Maurício Lacerda Nogueira; Rafael dos Santos Bezerra; Raul Machado Neto; Rejane Maria Tommasini Grotto; Ricardo Haddad; Sandra Coccuzzo Sampaio Vessoni; Simone Kashima; Svetoslav Nanev Slavov; Vincent Louis Viala
EPI_ISL_9321624, EPI_ISL_9321625, EPI_ISL_9321627, EPI_ISL_9321630, EPI_ISL_9321632, EPI_ISL_9321633, EPI_ISL_9321635, EPI_ISL_9321637, EPI_ISL_9321639, EPI_ISL_9321643, EPI_ISL_9321647, EPI_ISL_9321648, EPI_ISL_9321651, EPI_ISL_9321653, EPI_ISL_9321659, EPI_ISL_9321661, EPI_ISL_9321662, EPI_ISL_9321664, EPI_ISL_9321672, EPI_ISL_9321673, EPI_ISL_9321674, EPI_ISL_9321675, EPI_ISL_9321678, EPI_ISL_9321683, EPI_ISL_9321685, EPI_ISL_9321686, EPI_ISL_9321689, EPI_ISL_9321691, EPI_ISL_9321692, EPI_ISL_9321693, EPI_ISL_9321696, EPI_ISL_9321698, EPI_ISL_9321699, EPI_ISL_9321700, EPI_ISL_9321701, EPI_ISL_9321704, EPI_ISL_9321705, EPI_ISL_9321709, EPI_ISL_9321713			
see above	Lifebrain Covid Labor GmbH	Lifebrain Covid Labor GmbH	Alexandra Wagner; Anna Edermayr; Filip Sima
EPI_ISL_9315131, EPI_ISL_9315229	Lighthouse Lab in Milton Keynes	Wellcome Sanger Institute for the COVID-19 Genomics UK (COG-UK) Consortium	Cordelia Langford; David K. Jackson; Dominic Kwiatkowski; Ewan Harrison; Ian Johnston; Jeffrey Barrett; John Sillitoe on behalf of the Wellcome Sanger Institute COVID-19 Surveillance Team; Roberto Amato; Sonia Goncalves; The Lighthouse Lab in Milton Keynes and Alex Alderton
EPI_ISL_9314579	Lighthouse Laboratory Plymouth	Wellcome Sanger Institute for the COVID-19 Genomics UK (COG-UK) Consortium	Cordelia Langford; David K. Jackson; Dominic Kwiatkowski; Ewan Harrison; Ian Johnston; Jeffrey Barrett; John Sillitoe on behalf of the Wellcome Sanger Institute COVID-19 Surveillance Team; Lighthouse Laboratory Plymouth and Alex Alderton; Roberto Amato; Sonia Goncalves
EPI_ISL_9324289	MAHALAXMI LAYOUT	inStem, NCBS-INSACOG	Aswin SaiNarain; Dasaradhi Palakodeti; Uma Ramakrishnan
EPI_ISL_9322383, EPI_ISL_9322384, EPI_ISL_9322385, EPI_ISL_9322386, EPI_ISL_9322387	Nastavni zavod za javno zdravstvo "Andrija Štampar"	Hrvatski zavod za javno zdravstvo	Anita Jurić; Dragan Jurić; Irena Tabain; Ivana Ferenčak; Josipa Kuzle
EPI_ISL_9322388, EPI_ISL_9322389	Opća bolnica Bjelovar	Hrvatski zavod za javno zdravstvo	Anita Jurić; Dragan Jurić; Irena Tabain; Ivana Ferenčak; Josipa Kuzle
EPI_ISL_9303471, EPI_ISL_9303482, EPI_ISL_9303495, EPI_ISL_9303506, EPI_ISL_9303522, EPI_ISL_9303527, EPI_ISL_9303534, EPI_ISL_9303572, EPI_ISL_9303574, EPI_ISL_9303575, EPI_ISL_9303576, EPI_ISL_9303586, EPI_ISL_9303587, EPI_ISL_9303588, EPI_ISL_9303589, EPI_ISL_9303592, EPI_ISL_9303595, EPI_ISL_9303596, EPI_ISL_9303597, EPI_ISL_9303598, EPI_ISL_9303601, EPI_ISL_9303602, EPI_ISL_9303603, EPI_ISL_9303606, EPI_ISL_9303607, EPI_ISL_9303608, EPI_ISL_9303616, EPI_ISL_9303622, EPI_ISL_9303633, EPI_ISL_9303634, EPI_ISL_9303645, EPI_ISL_9303648, EPI_ISL_9303649, EPI_ISL_9303650, EPI_ISL_9303651, EPI_ISL_9303653, EPI_ISL_9303654, EPI_ISL_9303661, EPI_ISL_9303666			
see above	SARS-CoV-2 testing team, National Institute of Infectious Diseases	Pathogen Genomics Center, National Institute of Infectious Diseases	Hazuka Y Furihata; Kentaro Itokawa; Makoto Kuroda; Masanori Hashino; Masumichi Saito; Naomi Nojiri; Nozomu Hanaoka; Rina Tanaka; Tsuguto Fujimoto; Tsuyoshi Sekizuka
EPI_ISL_9324272	SYSTEMATIC LABS KOTHUNOOR FPEC	inStem, NCBS-INSACOG	Aswin SaiNarain; Dasaradhi Palakodeti; Uma Ramakrishnan
EPI_ISL_9346803, EPI_ISL_9346818	Seattle Flu Study	Seattle Flu Study	Amanda Adler; Barry R. Lutz; Benjamin Pelle; Caitlin R. Wolf; Chris D. Frazar; Deborah A. Nickerson; Elisabeth Brandstetter; Erica Ryke; Helen Y. Chu; Janet A. Englund; Jay Shendure; Jover Lee; Kairsten Fay; Kirsten Lacombe; Lea M. Starita; Mark J. Rieder; Matthew Richardson; Matthew Thompson; Melissa Truong; Michael Boeckh; Michael Famulare; Misja Ilcisin; Peter D. Han; Thomas R. Sibley; Trevor Bedford
EPI_ISL_9324984, EPI_ISL_9324985, EPI_ISL_9325099, EPI_ISL_9325220, EPI_ISL_9325222, EPI_ISL_9325232, EPI_ISL_9325304			
see above	Stadtspital Waid	Institute of Medical Virology, University of Zurich	Alexandra Trkola; Annette Audigé; Catharine Aquino; Cyril Shah; Daniel Ehrsam; Gabriela Ziltener; Guido Bloemberg; Hubert Rehrauer; Isabel Stürmer; Joel Wirz; Jon Huder; Jürg Böni; Kevin Steiner; Maria Grünberg; Maryam Zaheri; Michael Huber; Riccarda Capaul; Stefan Schmutz; Verena Kufner; Weihong Qi
EPI_ISL_9332967	The Ohio State University Applied Microbiology Services Laboratory	The Ohio State University Applied Microbiology Services Laboratory	Seth A. Faith PhD
EPI_ISL_9303742	Tokyo Medical and Dental University	Tokyo Medical and Dental University	Akinori Kimura; Hiroaki Takeuchi; Kousuke Tanimoto; Yoko Nukui; Yoshiaki Gu; Yukie Tanaka
EPI_ISL_9324824, EPI_ISL_9324825, EPI_ISL_9324826, EPI_ISL_9324871, EPI_ISL_9324962, EPI_ISL_9324963, EPI_ISL_9324968, EPI_ISL_9324969, EPI_ISL_9324970, EPI_ISL_9324971, EPI_ISL_9325091, EPI_ISL_9325092, EPI_ISL_9325093, EPI_ISL_9325094, EPI_ISL_9325096, EPI_ISL_9325097, EPI_ISL_9325098, EPI_ISL_9325100, EPI_ISL_9325101, EPI_ISL_9325102, EPI_ISL_9325103, EPI_ISL_9325104, EPI_ISL_9325105, EPI_ISL_9325106, EPI_ISL_9325107, EPI_ISL_9325109, EPI_ISL_9325201, EPI_ISL_9325202, EPI_ISL_9325203, EPI_ISL_9325204, EPI_ISL_9325205, EPI_ISL_9325206, EPI_ISL_9325210, EPI_ISL_9325211, EPI_ISL_9325212, EPI_ISL_9325214, EPI_ISL_9325215, EPI_ISL_9325216, EPI_ISL_9325218, EPI_ISL_9325219, EPI_ISL_9325512, EPI_ISL_9325513, EPI_ISL_9325515, EPI_ISL_9325516, EPI_ISL_9325517, EPI_ISL_9325518, EPI_ISL_9325519, EPI_ISL_9325520, EPI_ISL_9325521, EPI_ISL_9325525			
see above	Universität Zürich	Institute of Medical Virology, University of Zurich	Alexandra Trkola; Annette Audigé; Catharine Aquino; Cyril Shah; Daniel Ehrsam; Gabriela Ziltener; Guido Bloemberg; Hubert Rehrauer; Isabel Stürmer; Joel Wirz; Jon Huder; Jürg Böni; Kevin Steiner; Maria Grünberg; Maryam Zaheri; Michael Huber; Riccarda Capaul; Stefan Schmutz; Verena Kufner; Weihong Qi
EPI_ISL_9324996, EPI_ISL_9324998, EPI_ISL_9324999, EPI_ISL_9325228, EPI_ISL_9325229, EPI_ISL_9325230, EPI_ISL_9325234, EPI_ISL_9325237, EPI_ISL_9325305, EPI_ISL_9325309, EPI_ISL_9325310, EPI_ISL_9325311, EPI_ISL_9325312, EPI_ISL_9325313, EPI_ISL_9325505, EPI_ISL_9325506, EPI_ISL_9325507			
see above	Universitäts-Kinderspital Zürich	Institute of Medical Virology, University of Zurich	Alexandra Trkola; Annette Audigé; Catharine Aquino; Cyril Shah; Daniel Ehrsam; Gabriela Ziltener; Guido Bloemberg; Hubert Rehrauer; Isabel Stürmer; Joel Wirz; Jon Huder; Jürg Böni; Kevin Steiner; Maria Grünberg; Maryam Zaheri; Michael Huber; Riccarda Capaul; Stefan Schmutz; Verena Kufner; Weihong Qi
EPI_ISL_9324974, EPI_ISL_9324975, EPI_ISL_9324976, EPI_ISL_9324977, EPI_ISL_9324978, EPI_ISL_9324979, EPI_ISL_9324986, EPI_ISL_9324987, EPI_ISL_9324988, EPI_ISL_9324989, EPI_ISL_9324990, EPI_ISL_9324991, EPI_ISL_9324992, EPI_ISL_9324993, EPI_ISL_9324994, EPI_ISL_9324995, EPI_ISL_9325002, EPI_ISL_9325003, EPI_ISL_9325088, EPI_ISL_9325089, EPI_ISL_9325090, EPI_ISL_9325111, EPI_ISL_9325113, EPI_ISL_9325114, EPI_ISL_9325116, EPI_ISL_9325117, EPI_ISL_9325207, EPI_ISL_9325209, EPI_ISL_9325223, EPI_ISL_9325226, EPI_ISL_9325227, EPI_ISL_9325231, EPI_ISL_9325233, EPI_ISL_9325238, EPI_ISL_9325240, EPI_ISL_9325241, EPI_ISL_9325242, EPI_ISL_9325243, EPI_ISL_9325244, EPI_ISL_9325279, EPI_ISL_9325280, EPI_ISL_9325281, EPI_ISL_9325282, EPI_ISL_9325283, EPI_ISL_9325285, EPI_ISL_9325286, EPI_ISL_9325287, EPI_ISL_9325289, EPI_ISL_9325290, EPI_ISL_9325291, EPI_ISL_9325292, EPI_ISL_9325293, EPI_ISL_9325294, EPI_ISL_9325295, EPI_ISL_9325296, EPI_ISL_9325298, EPI_ISL_9325299, EPI_ISL_9325300, EPI_ISL_9325314, EPI_ISL_9325503, EPI_ISL_9325509, EPI_ISL_9325510, EPI_ISL_9325511			
see above	UniversitätsSpital Zürich	Institute of Medical Virology, University of Zurich	Alexandra Trkola; Annette Audigé; Catharine Aquino; Cyril Shah; Daniel Ehrsam; Gabriela Ziltener; Guido Bloemberg; Hubert Rehrauer; Isabel Stürmer; Joel Wirz; Jon Huder; Jürg Böni; Kevin Steiner; Maria Grünberg; Maryam Zaheri; Michael Huber; Riccarda Capaul; Stefan Schmutz; Verena Kufner; Weihong Qi
EPI_ISL_9322465, EPI_ISL_9322466,	Zavod za javno zdravstvo	Hrvatski zavod za javno zdravstvo	Anita Jurić; Dragan Jurić; Irena Tabain; Ivana Ferenčak; Josipa Kuzle

EPI_ISL_9322566, EPI_ISL_9322568, EPI_ISL_9322569, EPI_ISL_9322570	Šibensko- Kninske županije

We gratefully acknowledge the following Authors from the Originating laboratories responsible for obtaining the specimens, as well as the Submitting laboratories where the genome data were generated and shared via GISAID, on which this research is based.

All Submitters of data may be contacted directly via www.gisaid.org

Authors are sorted alphabetically.

Accession ID	Originating Laboratory	Submitting Laboratory	Authors
EPI_ISL_8439646	ROB FERREIRA LABORATORY	National Institute for Communicable Diseases of the National Health Laboratory Service	Amoako DG; Bhiman JN; Everatt J; Ismail A; Kekana D; Mahlangu B; Mnguni A; Mohale T; Ntuli N; Scheepers C; Wolter N

We gratefully acknowledge the following Authors from the Originating laboratories responsible for obtaining the specimens, as well as the Submitting laboratories where the genome data were generated and shared via GISAID, on which this research is based.

All Submitters of data may be contacted directly via www.gisaid.org

Authors are sorted alphabetically.

Acknowledgement EPI_SET Identifier: EPI_SET_20220527kq

Accession ID	Originating Laboratory	Submitting Laboratory	Authors
EPI_ISL_8476913	Child Health Research Foundation	Child Health Research Foundation	CHRF Bangladesh Genomics Team
EPI_ISL_8452449, EPI_ISL_8452453, EPI_ISL_8452455, EPI_ISL_8452456, EPI_ISL_8452458, EPI_ISL_8452459, EPI_ISL_8452464, EPI_ISL_8452465, EPI_ISL_8452466, EPI_ISL_8452467, EPI_ISL_8452468, EPI_ISL_8452469, EPI_ISL_8452470, EPI_ISL_8452471, EPI_ISL_8452472, EPI_ISL_8452473, EPI_ISL_8452478, EPI_ISL_8452480, EPI_ISL_8452492, EPI_ISL_8452493, EPI_ISL_8452494, EPI_ISL_8452496, EPI_ISL_8452497, EPI_ISL_8452498, EPI_ISL_8452500, EPI_ISL_8452501, EPI_ISL_8452502, EPI_ISL_8452506, EPI_ISL_8452507, EPI_ISL_8452508			
see above	Gandhi Medical College and Hospital (GMCH), Secunderabad	NIV Influenza	D.R.Manisha Rani; Dr.G.Sushma Rajya Lakshmi; Dr.K.Nagamani; Dr.Sunitha Pakalapaty
EPI_ISL_8445176, EPI_ISL_8445224, EPI_ISL_8445225, EPI_ISL_8445227, EPI_ISL_8445229, EPI_ISL_8445230, EPI_ISL_8445234, EPI_ISL_8445236, EPI_ISL_8445237, EPI_ISL_8445238, EPI_ISL_8445239, EPI_ISL_8445240, EPI_ISL_8445241, EPI_ISL_8445242, EPI_ISL_8445248			
see above	ICMR-National Institute of Virology - INSACOG	NIV Influenza	Dr. Varsha Potdar and NIC Team
EPI_ISL_8435250, EPI_ISL_8435282, EPI_ISL_8449933, EPI_ISL_8450019	National Public Health Laboratory, National Centre for Infectious Diseases	National Public Health Laboratory, National Centre for Infectious Diseases	Benny Yeo; Grace Ngan; Lin Cui; Raymond Tzer Pin Lin; Royce Ang; Samuel Loo; Zhenyang Zhou

We gratefully acknowledge the following Authors from the Originating laboratories responsible for obtaining the specimens, as well as the Submitting laboratories where the genome data were generated and shared via GISAID, on which this research is based.

All Submitters of data may be contacted directly via www.gisaid.org

Authors are sorted alphabetically.

Accession ID	Originating Laboratory	Submitting Laboratory	Authors
EPI_ISL_8457787, EPI_ISL_8457848, EPI_ISL_8457866, EPI_ISL_8457869, EPI_ISL_8457900, EPI_ISL_8457968, EPI_ISL_8458005, EPI_ISL_8458066, EPI_ISL_8458091, EPI_ISL_8458107, EPI_ISL_8458112, EPI_ISL_8458115, EPI_ISL_8458202, EPI_ISL_8458234, EPI_ISL_8458251, EPI_ISL_8458287, EPI_ISL_8458295, EPI_ISL_8458315, EPI_ISL_8458335, EPI_ISL_8458358, EPI_ISL_8458365, EPI_ISL_8458395, EPI_ISL_8458401, EPI_ISL_8458441, EPI_ISL_8458499, EPI_ISL_8458519, EPI_ISL_8458533, EPI_ISL_8458549, EPI_ISL_8458581, EPI_ISL_8458697, EPI_ISL_8458709, EPI_ISL_8458712, EPI_ISL_8458719, EPI_ISL_8458720, EPI_ISL_8458725, EPI_ISL_8458805, EPI_ISL_8458827, EPI_ISL_8458882, EPI_ISL_8458893, EPI_ISL_8458949, EPI_ISL_8458987, EPI_ISL_8459001, EPI_ISL_8459015, EPI_ISL_8459054, EPI_ISL_8459102, EPI_ISL_8459153, EPI_ISL_8459200, EPI_ISL_8459211, EPI_ISL_8459223, EPI_ISL_8459226, EPI_ISL_8459231, EPI_ISL_8459237, EPI_ISL_8459273, EPI_ISL_8459292, EPI_ISL_8459303, EPI_ISL_8459314, EPI_ISL_8459318, EPI_ISL_8459340, EPI_ISL_8459365, EPI_ISL_8459409, EPI_ISL_8459443, EPI_ISL_8459450, EPI_ISL_8459452, EPI_ISL_8459457, EPI_ISL_8459458, EPI_ISL_8459460, EPI_ISL_8459462, EPI_ISL_8459468, EPI_ISL_8459472, EPI_ISL_8459480, EPI_ISL_8459496, EPI_ISL_8459500, EPI_ISL_8459553, EPI_ISL_8459569, EPI_ISL_8459616, EPI_ISL_8459644, EPI_ISL_8459664, EPI_ISL_8459728, EPI_ISL_8459737, EPI_ISL_8459747, EPI_ISL_8459753, EPI_ISL_8459775, EPI_ISL_8459800, EPI_ISL_8459852, EPI_ISL_8459872, EPI_ISL_8459884, EPI_ISL_8459932, EPI_ISL_8459947, EPI_ISL_8459957, EPI_ISL_8459972, EPI_ISL_8460062, EPI_ISL_8460088, EPI_ISL_8460097, EPI_ISL_8460119, EPI_ISL_8460132, EPI_ISL_8460139, EPI_ISL_8460165, EPI_ISL_8460171, EPI_ISL_8460178, EPI_ISL_8460189, EPI_ISL_8460312, EPI_ISL_8460322, EPI_ISL_8460345, EPI_ISL_8461029, EPI_ISL_8461911, EPI_ISL_8462122, EPI_ISL_8462203, EPI_ISL_8462224, EPI_ISL_8462240, EPI_ISL_8462259, EPI_ISL_8462279, EPI_ISL_8462434, EPI_ISL_8462475, EPI_ISL_8462489, EPI_ISL_8462517, EPI_ISL_8462580, EPI_ISL_8462585, EPI_ISL_8462604, EPI_ISL_8462611, EPI_ISL_8462613, EPI_ISL_8462614, EPI_ISL_8462622, EPI_ISL_8462628, EPI_ISL_8462649, EPI_ISL_8462658, EPI_ISL_8462661, EPI_ISL_8462674, EPI_ISL_8462686, EPI_ISL_8462688, EPI_ISL_8462701, EPI_ISL_8462703, EPI_ISL_8462709, EPI_ISL_8462717, EPI_ISL_8462724, EPI_ISL_8462730, EPI_ISL_8462732, EPI_ISL_8462739, EPI_ISL_8462760, EPI_ISL_8462772, EPI_ISL_8462797, EPI_ISL_8462811, EPI_ISL_8462812, EPI_ISL_8462817, EPI_ISL_8462818, EPI_ISL_8462824, EPI_ISL_8462829, EPI_ISL_8462831, EPI_ISL_8462842, EPI_ISL_8462878, EPI_ISL_8462892, EPI_ISL_8462897, EPI_ISL_8462898, EPI_ISL_8462899, EPI_ISL_8462902, EPI_ISL_8462904, EPI_ISL_8462908, EPI_ISL_8462912, EPI_ISL_8462929, EPI_ISL_8462930, EPI_ISL_8462935, EPI_ISL_8462956, EPI_ISL_8462972, EPI_ISL_8462979, EPI_ISL_8463053, EPI_ISL_8463055, EPI_ISL_8463061, EPI_ISL_8463082, EPI_ISL_8463087, EPI_ISL_8463091, EPI_ISL_8463097, EPI_ISL_8463101, EPI_ISL_8463111, EPI_ISL_8463112, EPI_ISL_8463119, EPI_ISL_8463120, EPI_ISL_8463121, EPI_ISL_8463124, EPI_ISL_8463128, EPI_ISL_8463130, EPI_ISL_8463136, EPI_ISL_8463143, EPI_ISL_8463151, EPI_ISL_8463153, EPI_ISL_8463165, EPI_ISL_8463182, EPI_ISL_8463199, EPI_ISL_8463207, EPI_ISL_8463215, EPI_ISL_8463230, EPI_ISL_8463243, EPI_ISL_8463261, EPI_ISL_8463279, EPI_ISL_8463289, EPI_ISL_8463290, EPI_ISL_8463292, EPI_ISL_8463296, EPI_ISL_8463302, EPI_ISL_8463303, EPI_ISL_8463306, EPI_ISL_8463324, EPI_ISL_8463328, EPI_ISL_8465069, EPI_ISL_8465070, EPI_ISL_8465082, EPI_ISL_8465083, EPI_ISL_8465088, EPI_ISL_8465089, EPI_ISL_8465090, EPI_ISL_8465095, EPI_ISL_8465096, EPI_ISL_8465100, EPI_ISL_8465119, EPI_ISL_8465121, EPI_ISL_8465123, EPI_ISL_8465125, EPI_ISL_8465128, EPI_ISL_8465133, EPI_ISL_8465135, EPI_ISL_8465137, EPI_ISL_8465140, EPI_ISL_8465141, EPI_ISL_8465142, EPI_ISL_8465151, EPI_ISL_8465154, EPI_ISL_8465156, EPI_ISL_8465157, EPI_ISL_8465165, EPI_ISL_8465169, EPI_ISL_8465170, EPI_ISL_8465178, EPI_ISL_8465181, EPI_ISL_8465187, EPI_ISL_8465190, EPI_ISL_8465192, EPI_ISL_8465193, EPI_ISL_8465200, EPI_ISL_8465206, EPI_ISL_8465207, EPI_ISL_8465210, EPI_ISL_8465213, EPI_ISL_8465214, EPI_ISL_8465220, EPI_ISL_8465222, EPI_ISL_8465226, EPI_ISL_8465243, EPI_ISL_8465244, EPI_ISL_8465246, EPI_ISL_8465247, EPI_ISL_8465248, EPI_ISL_8465251, EPI_ISL_8465258, EPI_ISL_8465262, EPI_ISL_8465268, EPI_ISL_8465273, EPI_ISL_8465280, EPI_ISL_8465283, EPI_ISL_8465284, EPI_ISL_8465285, EPI_ISL_8465288, EPI_ISL_8465290, EPI_ISL_8465291, EPI_ISL_8465293, EPI_ISL_8465294, EPI_ISL_8465296, EPI_ISL_8465301, EPI_ISL_8465303, EPI_ISL_8465307, EPI_ISL_8465309, EPI_ISL_8465314, EPI_ISL_8465315, EPI_ISL_8465319, EPI_ISL_8465322, EPI_ISL_8465331, EPI_ISL_8465333, EPI_ISL_8465343, EPI_ISL_8465351, EPI_ISL_8465355, EPI_ISL_8465356, EPI_ISL_8465361, EPI_ISL_8465367, EPI_ISL_8465370, EPI_ISL_8465371, EPI_ISL_8465372, EPI_ISL_8465373, EPI_ISL_8465377, EPI_ISL_8465383, EPI_ISL_8465385, EPI_ISL_8465386, EPI_ISL_8465394, EPI_ISL_8465396, EPI_ISL_8465401, EPI_ISL_8465410, EPI_ISL_8465414, EPI_ISL_8465416, EPI_ISL_8465418, EPI_ISL_8465419, EPI_ISL_8465421, EPI_ISL_8465422, EPI_ISL_8465425, EPI_ISL_8465427, EPI_ISL_8465441, EPI_ISL_8465445, EPI_ISL_8465449, EPI_ISL_8465450, EPI_ISL_8465456, EPI_ISL_8465458, EPI_ISL_8465462, EPI_ISL_8465465, EPI_ISL_8465477, EPI_ISL_8465490, EPI_ISL_8465491, EPI_ISL_8465495, EPI_ISL_8465497, EPI_ISL_8465498, EPI_ISL_8465499, EPI_ISL_8465502, EPI_ISL_8465508, EPI_ISL_8465513, EPI_ISL_8465518, EPI_ISL_8465521, EPI_ISL_8465522, EPI_ISL_8465525, EPI_ISL_8465527, EPI_ISL_8465797, EPI_ISL_8465798, EPI_ISL_8465799, EPI_ISL_8465800, EPI_ISL_8465801, EPI_ISL_8465806, EPI_ISL_8465810, EPI_ISL_8465815, EPI_ISL_8465816, EPI_ISL_8465819, EPI_ISL_8465829, EPI_ISL_8465834, EPI_ISL_8465837, EPI_ISL_8465840, EPI_ISL_8465847, EPI_ISL_8465850, EPI_ISL_8465855, EPI_ISL_8465856, EPI_ISL_8465860, EPI_ISL_8465864, EPI_ISL_8465876, EPI_ISL_8465879, EPI_ISL_8465889, EPI_ISL_8465895, EPI_ISL_8465898, EPI_ISL_8465900, EPI_ISL_8465902, EPI_ISL_8465904, EPI_ISL_8465915, EPI_ISL_8465918, EPI_ISL_8465921, EPI_ISL_8465930, EPI_ISL_8465931, EPI_ISL_8465944, EPI_ISL_8465950, EPI_ISL_8465953, EPI_ISL_8465956, EPI_ISL_8465957, EPI_ISL_8465958, EPI_ISL_8465959, EPI_ISL_8465962, EPI_ISL_8465971, EPI_ISL_8465972, EPI_ISL_8465974, EPI_ISL_8465975, EPI_ISL_8465976, EPI_ISL_8465977, EPI_ISL_8465979, EPI_ISL_8465989, EPI_ISL_8465991, EPI_ISL_8465998, EPI_ISL_8466001, EPI_ISL_8466004, EPI_ISL_8466019, EPI_ISL_8466022, EPI_ISL_8466023, EPI_ISL_8466027, EPI_ISL_8466031, EPI_ISL_8466033			
see above	Department of Bacteria, Parasites and Fungi, Statens Serum Institut, Copenhagen, Denmark	Statens Serum Institut Bioinformatics and Microbial Genomics	Danish Covid-19 Genome Consortium
EPI_ISL_8461493	Dutch COVID-19 response team	National Institute for Public Health and the Environment (RIVM)	Adam Meijer; AnneMarie van den Brandt; Annelies Kroneman; Bas van der Veer; Chantal Reusken; Dennis Schmitz; Dirk Eggink; Florian Zwagemaker; Harry Vennema; Ivo van Walle; Jeroen Cremer; Jil Kocken; Jordy de Bakker; Karim Hajji; Kim Freriks; Linda van Someren; Lisa Wijsman; Lynn Aarts; Ryanne Jaarsma; Sanne Bos; Sharon van den Brink; Stijn van Rossum; on behalf of the national COVID-19 response team
EPI_ISL_8455623	Jessa	Jessa	Severine Berden et al. on behalf of the Jessa_cmdLab
EPI_ISL_8467775	PZU “Eurofarm - Centar Poliklinika”	University of Sarajevo, Veterinary Faculty, Laboratory for Molecular Diagnostic and Research	Goletic S; Goletic T; Hodzic A; Huseinbegovic E; Jazic A; Nicevic M.; Prguda-Mujic J; Sabic E; Softic A; Terzic I
EPI_ISL_8444273	Public Health Laboratory, Public Health Service Amsterdam, The Netherlands	Department of Medical Microbiology & Infection prevention, Amsterdam University Medical Centers location AMC	Akke Cornelissen; Fokla Zorgdrager; Janke Schinkel; Jelle Koopsen; Judith den Uil; Marcel Jonges; Matthijs Welkers; Menno de Jong; Robin van Houdt; Sebastien Matamoros; Sjoerd Rebers; Sylvia Bruisten; Tjalling Leenstra and Mariken van der Lubben on behalf of the Amsterdam Regional Genomic epidemiology and Outbreak Surveillance (ARGOS) consortium
EPI_ISL_8448885	Rosalind Franklin Laboratory	Wellcome Sanger Institute for the COVID-19 Genomics UK (COG-UK) Consortium	Cordelia Langford; David K. Jackson; Dominic Kwiatkowski; Donald Fraser; Ewan Harrison; Ian Johnston; Jeffrey Barrett; John Sillitoe on behalf of the Wellcome Sanger Institute COVID-19 Surveillance Team; Rob Howes; Roberto Amato; Sonia Goncalves; Suki Lee; The Rosalind Franklin Laboratory and Alex Alderton
EPI_ISL_8467952, EPI_ISL_8468322	University Clinical Hospital of Mostar, Department of Microbiology and Molecular Diagnostics	University of Sarajevo, Veterinary Faculty, Laboratory for Molecular Diagnostic and Research	Goletic S; Goletic T; Herceg I; Hodzic A; Jazic A; Nicevic M; Ostojic M; Sabic E; Skocibusic S; Softic A; Terzic I
EPI_ISL_8466468, EPI_ISL_8466469	University Hospitals of Geneva, Laboratory of Virology	HUG, Laboratory of Virology and the Health2030 Genome Center	Aline Mamin; Ana Rita Goncalves; Cedric Howald; Deborah Penet; Francisco Perez; Henri Pegeot; Ioannis Xenarios; Keith Harshman; Laurent Kaiser; Lorenzo Cerutti; Melyssa Elies; Samuel Cordey
EPI_ISL_8468540	University of Sarajevo, Veterinary Faculty, Laboratory for Molecular Diagnostic and Research	University of Sarajevo, Veterinary Faculty, Laboratory for Molecular Diagnostic and Research	Goletic S; Goletic T; Hodzic A; Jazic A; Nicevic M; Sabic E; Softic A; Terzic I

We gratefully acknowledge the following Authors from the Originating laboratories responsible for obtaining the specimens, as well as the Submitting laboratories where the genome data were generated and shared via GISAID, on which this research is based.

All Submitters of data may be contacted directly via www.gisaid.org

Authors are sorted alphabetically.

Acknowledgement EPI_SET Identifier: EPI_SET_20220527qm

Accession ID	Originating Laboratory	Submitting Laboratory	Authors
EPI_ISL_8471279, EPI_ISL_8472155	Aegis Sciences Corporation	Centers for Disease Control and Prevention Division of Viral Diseases, Pathogen Discovery	Alec Vest; Benjamin Rambo-Martin; Christopher Gulvick; Clinton Paden; Cyndi Clark; Dakota Howard; Dhwani Batra; Dillon Nall; Duncan MacCannell; Erisa Sula; Ethan Sanders; Holly Houdeshell; Jason Caravas; Kristine Lacek; Matthew Hardison; Matthew Schmerer; Ola Kvalvaag; Patrick Campbell; Peter Cook; Rob Case; Scott Sammons; Shatavia Morrison; Shaun Westlund; Tymeckia Kendall; Victoria Caban Figueroa; Vikramsinha Ghorpade; Yvette Unoarumhi
EPI_ISL_8476795, EPI_ISL_8476798, EPI_ISL_8476799, EPI_ISL_8476800	Colorado Department of Public Health and Environment	Colorado Department of Public Health and Environment	Alexandria Rossheim; Arianna Smith; Diana Ir; Emily A. Travanty; Laura Bankers; Mandy Waters; Michael Martin; Molly C. Hetherington-Rauth; Shannon R. Matzinger
EPI_ISL_8475117	Fulgent Genetics	Centers for Disease Control and Prevention Division of Viral Diseases, Pathogen Discovery	Becky Tsai; Benafsh Sapra; Benjamin Rambo-Martin; Christopher Gulvick; Clinton Paden; Dakota Howard; Dhwani Batra; Doreen Ng; Duncan MacCannell; Erisa Sula; Harry Gao; James Xie; Jason Caravas; John Gao; Joseph Fierro; Kristine Lacek; Matthew Schmerer; Mickey Li; Peter Cook; Scott Sammons; Shatavia Morrison; Tymeckia Kendall; Victoria Caban Figueroa; Yan Meng; Yvette Unoarumhi
EPI_ISL_8481779, EPI_ISL_8482526	Houston Methodist Hospital	Houston Methodist Hospital	Akanksha Batajoo; Ilya J. Finkelstein; James J. Davis; Jessica Cambric; Jimmy Gollihar; Kristina Reppond; Madison N. Shyer; Marcus Nguyen; Matthew Ojeda Saavedra; Paul A. Christensen; Randall J. Olsen; Rashi Thakur; Regan Mangham; Richard Snehall; Robert Olson; Ryan Gadd; S. Wesley Long; Sindy Pena; and James M. Musser
EPI_ISL_8473672	Johns Hopkins Hospital Department of Pathology	Johns Hopkins Hospital Department of Pathology	Amary Fall; C. Paul Morris; David Gaston; Heba H. Mostafa; Julie M. Norton; Matthew Schwartz; Michael Forman; Raghda Eldesouki
EPI_ISL_8478365, EPI_ISL_8478587, EPI_ISL_8478658, EPI_ISL_8479317	Kaiser Permanente Southern California	Helix	Helix; Kaiser Permanente Southern California
EPI_ISL_8469485, EPI_ISL_8469703, EPI_ISL_8469732, EPI_ISL_8469868, EPI_ISL_8469869, EPI_ISL_8469990, EPI_ISL_8470301, EPI_ISL_8470302			
see above	Laboratory Corporation of America	Centers for Disease Control and Prevention Division of Viral Diseases, Pathogen Discovery	Amanda Douglas; Amanda Suchanek; Andrea Throop; Ayla Burns; Benjamin Rambo-Martin; Bobbi Croy; Brian Krueger; Brian Norvell; Christopher Gulvick; Christos Petropoulos; Clinton Paden; Craig Lukasik; Dakota Howard; Debbie Boles; Dhwani Batra; Duncan MacCannell; Eyad Almasri; Goran Stevovic; Howard Engler; Hrushikesh Deshmukh; Jake Humphrey; Jana Schroth; Jason Caravas; Joe Voshell; John Pruitt; Jonathan Meltzer; Jonathan Williams; Kimberly Wagner; Kristine Lacek; Lax Iyer; Lisa Pfefferle; Lyndon Tilson; Manoj Jain; Marcia Eisenberg; Mary Cristobal; Mary Williamson; Matthew Robinson; Matthew Schmerer; Michael Levandoski; Mike Sapeta; Mindy Nye; Minoo Agarwal; Mohan Kolli; Nuthawin Charoensri; Oren Cohen; Peter Cook; Prashant Gupta; Qian Zeng; Rama Ghatti; Scott Parker; Scott Ryan; Scott Sammons; Shatavia Morrison; Stanley Letovsky; Steven Ragan; Suresh Selvaraju; Susan Countryman; Susan Hicks; Suzanne Dale; Thomas Urban; Tim Kuphal; Tricia Zwiefelhofer; Tymeckia Kendall; Victoria Caban Figueroa; Vincent Drouillon; Yvette Unoarumhi
EPI_ISL_8479529, EPI_ISL_8479530, EPI_ISL_8479531, EPI_ISL_8479532, EPI_ISL_8479533, EPI_ISL_8479534, EPI_ISL_8479535, EPI_ISL_8479536, EPI_ISL_8479537, EPI_ISL_8479538, EPI_ISL_8479539, EPI_ISL_8479540, EPI_ISL_8479541, EPI_ISL_8479542, EPI_ISL_8479543, EPI_ISL_8479544, EPI_ISL_8479545, EPI_ISL_8479546, EPI_ISL_8479547, EPI_ISL_8479548, EPI_ISL_8479549, EPI_ISL_8479550, EPI_ISL_8479551, EPI_ISL_8479552, EPI_ISL_8479553, EPI_ISL_8479554, EPI_ISL_8479555, EPI_ISL_8479556, EPI_ISL_8479557, EPI_ISL_8479558, EPI_ISL_8479559, EPI_ISL_8479560, EPI_ISL_8479561, EPI_ISL_8479562, EPI_ISL_8479563, EPI_ISL_8479564, EPI_ISL_8479565, EPI_ISL_8479586			
see above	Ochsner Health	BioInfoExperts	Amy Feehan; Ben Lain; Chris Huston; David J. Nolan; Judy Crabtree; Julia-Garcia-Diaz; Lucio Miele; Rebecca Rose; Samual Moot; Susanna L. Lamers; Tessa LaFleur
EPI_ISL_8434986	State Laboratories Division, Hawaii State Department of Health	State Laboratories Division, Hawaii State Department of Health	Ayana Garnet; Briana Ofilas; Cheryl-lynn Daquip; Cheyenne Barela; Daniel Strange; Drew Kuwazaki; Edward Desmond; Jeffrey Au; Mark Nagata; Pamela O'Brien; Razvan Sultana; Remedios Gose; Samantha Cotter; Samantha Sruba
EPI_ISL_8479393, EPI_ISL_8480230, EPI_ISL_8480393, EPI_ISL_8480457	UW Virology Lab	UW Virology Lab	Alexander Greninger; Hong Xie; Isabel Arnould; Keith R Jerome; Meei-Li Huang; Nathan Breit; Patrick Mathias; Pavitra Roychoudhury; Pooneh Hajian; Ricardo Perez; Robert J. Livingston; Saraswathi Sathees; Sean Ellis; Seffir T. Wendm; Shah Mohamed Bakhash; Tien V. Nguyen
EPI_ISL_8435628, EPI_ISL_8435634, EPI_ISL_8435636, EPI_ISL_8435640, EPI_ISL_8435641, EPI_ISL_8435643, EPI_ISL_8435644, EPI_ISL_8435645, EPI_ISL_8435646, EPI_ISL_8435651, EPI_ISL_8435655, EPI_ISL_8435656, EPI_ISL_8435661, EPI_ISL_8435664, EPI_ISL_8435666, EPI_ISL_8435672, EPI_ISL_8435680, EPI_ISL_8435684, EPI_ISL_8435693, EPI_ISL_8435695, EPI_ISL_8435698, EPI_ISL_8435701, EPI_ISL_8435702, EPI_ISL_8435703, EPI_ISL_8435704, EPI_ISL_8435705, EPI_ISL_8435716, EPI_ISL_8435723, EPI_ISL_8435727, EPI_ISL_8435728, EPI_ISL_8435731, EPI_ISL_8435743, EPI_ISL_8435744, EPI_ISL_8435748, EPI_ISL_8435750, EPI_ISL_8435751, EPI_ISL_8435752, EPI_ISL_8435757, EPI_ISL_8435765			
see above	University of Oregon COVID-19 MAP Laboratory	University of Oregon Genomics and Cell Charactericzation Core Facility (GC3F)	Ariana White; Demi Glidden; Douglas Turnbull; Jason Carriere; Jason Sydes; Jeff Bishop; Megan Criss; Peter Batzel; Tina Arredondo

We gratefully acknowledge the following Authors from the Originating laboratories responsible for obtaining the specimens, as well as the Submitting laboratories where the genome data were generated and shared via GISAID, on which this research is based.

All Submitters of data may be contacted directly via www.gisaid.org

Authors are sorted alphabetically.

Accession ID	Originating Laboratory	Submitting Laboratory	Authors
EPI_ISL_8437525, EPI_ISL_8437527, EPI_ISL_8437528, EPI_ISL_8437529, EPI_ISL_8437530, EPI_ISL_8437532, EPI_ISL_8437536, EPI_ISL_8437537, EPI_ISL_8437539, EPI_ISL_8437540, EPI_ISL_8437542, EPI_ISL_8437543, EPI_ISL_8437544, EPI_ISL_8437547, EPI_ISL_8437548, EPI_ISL_8437549, EPI_ISL_8437550, EPI_ISL_8437551, EPI_ISL_8437552, EPI_ISL_8437553, EPI_ISL_8437555, EPI_ISL_8437556, EPI_ISL_8437557, EPI_ISL_8437558, EPI_ISL_8437560, EPI_ISL_8437561, EPI_ISL_8437562, EPI_ISL_8437563, EPI_ISL_8437564, EPI_ISL_8437566, EPI_ISL_8437567, EPI_ISL_8437570, EPI_ISL_8437571, EPI_ISL_8437572, EPI_ISL_8437573, EPI_ISL_8437575, EPI_ISL_8437576, EPI_ISL_8437577, EPI_ISL_8437580, EPI_ISL_8437581, EPI_ISL_8437582, EPI_ISL_8437583, EPI_ISL_8437584, EPI_ISL_8437585, EPI_ISL_8437586, EPI_ISL_8437587, EPI_ISL_8437588, EPI_ISL_8437590, EPI_ISL_8437592, EPI_ISL_8437594, EPI_ISL_8437595, EPI_ISL_8437596, EPI_ISL_8437597, EPI_ISL_8437598, EPI_ISL_8437602, EPI_ISL_8437604, EPI_ISL_8437608, EPI_ISL_8437609, EPI_ISL_8437614, EPI_ISL_8437615, EPI_ISL_8437616, EPI_ISL_8437623, EPI_ISL_8437624, EPI_ISL_8437625, EPI_ISL_8437627, EPI_ISL_8437628, EPI_ISL_8437629, EPI_ISL_8437633, EPI_ISL_8437634, EPI_ISL_8437637, EPI_ISL_8437639, EPI_ISL_8437641, EPI_ISL_8437642, EPI_ISL_8437644, EPI_ISL_8437645, EPI_ISL_8437649, EPI_ISL_8437651, EPI_ISL_8437652, EPI_ISL_8437653, EPI_ISL_8437655, EPI_ISL_8437657, EPI_ISL_8437658, EPI_ISL_8437660, EPI_ISL_8437661, EPI_ISL_8437662, EPI_ISL_8437663, EPI_ISL_8437668, EPI_ISL_8437669, EPI_ISL_8437670, EPI_ISL_8437672, EPI_ISL_8437673, EPI_ISL_8437674, EPI_ISL_8437678, EPI_ISL_8437680, EPI_ISL_8437681, EPI_ISL_8437683, EPI_ISL_8437684, EPI_ISL_8437685, EPI_ISL_8437686, EPI_ISL_8437689, EPI_ISL_8437690, EPI_ISL_8437691, EPI_ISL_8437692, EPI_ISL_8437693, EPI_ISL_8437697, EPI_ISL_8437700, EPI_ISL_8437701, EPI_ISL_8437706, EPI_ISL_8437707, EPI_ISL_8437710, EPI_ISL_8437711, EPI_ISL_8437714, EPI_ISL_8437715, EPI_ISL_8437717, EPI_ISL_8437718, EPI_ISL_8437720, EPI_ISL_8437721, EPI_ISL_8437723, EPI_ISL_8437724, EPI_ISL_8437728, EPI_ISL_8437730, EPI_ISL_8437731, EPI_ISL_8437732, EPI_ISL_8437733, EPI_ISL_8437734, EPI_ISL_8437738, EPI_ISL_8437740, EPI_ISL_8437741, EPI_ISL_8437742, EPI_ISL_8437744, EPI_ISL_8437745, EPI_ISL_8437746, EPI_ISL_8437747, EPI_ISL_8437749, EPI_ISL_8437751, EPI_ISL_8437753, EPI_ISL_8437757, EPI_ISL_8437759, EPI_ISL_8437761, EPI_ISL_8437765, EPI_ISL_8437767, EPI_ISL_8437771, EPI_ISL_8437773, EPI_ISL_8437774, EPI_ISL_8437776, EPI_ISL_8437778, EPI_ISL_8437779, EPI_ISL_8437780, EPI_ISL_8437781, EPI_ISL_8437782, EPI_ISL_8437783, EPI_ISL_8437784, EPI_ISL_8437786, EPI_ISL_8437788, EPI_ISL_8437789, EPI_ISL_8437791, EPI_ISL_8437792, EPI_ISL_8437793, EPI_ISL_8437794, EPI_ISL_8437796, EPI_ISL_8437797, EPI_ISL_8437798, EPI_ISL_8437799, EPI_ISL_8437801, EPI_ISL_8437803, EPI_ISL_8437804, EPI_ISL_8437807, EPI_ISL_8437809, EPI_ISL_8437810, EPI_ISL_8437813, EPI_ISL_8437814, EPI_ISL_8437815, EPI_ISL_8437820, EPI_ISL_8437821, EPI_ISL_8437822, EPI_ISL_8437823, EPI_ISL_8437824, EPI_ISL_8437825, EPI_ISL_8437828, EPI_ISL_8437832, EPI_ISL_8437833, EPI_ISL_8437836, EPI_ISL_8437838, EPI_ISL_8437841, EPI_ISL_8437847, EPI_ISL_8437849, EPI_ISL_8437850, EPI_ISL_8437851, EPI_ISL_8437858, EPI_ISL_8437867, EPI_ISL_8437871, EPI_ISL_8437875, EPI_ISL_8437878, EPI_ISL_8437880, EPI_ISL_8437881, EPI_ISL_8437884, EPI_ISL_8437888, EPI_ISL_8437896, EPI_ISL_8437904, EPI_ISL_8437909, EPI_ISL_8437910, EPI_ISL_8437914, EPI_ISL_8437915, EPI_ISL_8437919, EPI_ISL_8437920, EPI_ISL_8437926, EPI_ISL_8437932, EPI_ISL_8437933, EPI_ISL_8437934, EPI_ISL_8437937, EPI_ISL_8437952, EPI_ISL_8437954, EPI_ISL_8437958, EPI_ISL_8437960, EPI_ISL_8437965, EPI_ISL_8437970, EPI_ISL_8437973, EPI_ISL_8437980, EPI_ISL_8437981, EPI_ISL_8437982, EPI_ISL_8437985, EPI_ISL_8437986, EPI_ISL_8437990, EPI_ISL_8437991, EPI_ISL_8437992, EPI_ISL_8437993, EPI_ISL_8437994, EPI_ISL_8437996, EPI_ISL_8437999, EPI_ISL_8438002, EPI_ISL_8438003, EPI_ISL_8438004, EPI_ISL_8438005, EPI_ISL_8438006, EPI_ISL_8438007, EPI_ISL_8438008, EPI_ISL_8438010, EPI_ISL_8438011, EPI_ISL_8438012, EPI_ISL_8438013, EPI_ISL_8438015, EPI_ISL_8438017, EPI_ISL_8438018, EPI_ISL_8438019, EPI_ISL_8438020, EPI_ISL_8438022, EPI_ISL_8438024, EPI_ISL_8438027, EPI_ISL_8438028, EPI_ISL_8438030, EPI_ISL_8438034, EPI_ISL_8438035, EPI_ISL_8438036, EPI_ISL_8438038, EPI_ISL_8438039, EPI_ISL_8438040, EPI_ISL_8438041, EPI_ISL_8438044, EPI_ISL_8438045, EPI_ISL_8438046, EPI_ISL_8438048, EPI_ISL_8438049, EPI_ISL_8438050, EPI_ISL_8438053, EPI_ISL_8438054, EPI_ISL_8438056, EPI_ISL_8438058, EPI_ISL_8438059, EPI_ISL_8438060, EPI_ISL_8438061, EPI_ISL_8438063, EPI_ISL_8438064, EPI_ISL_8438065, EPI_ISL_8438066, EPI_ISL_8438069, EPI_ISL_8438070, EPI_ISL_8438072, EPI_ISL_8438073, EPI_ISL_8438076, EPI_ISL_8438077, EPI_ISL_8438078, EPI_ISL_8438080, EPI_ISL_8438082, EPI_ISL_8438083, EPI_ISL_8438084, EPI_ISL_8438089, EPI_ISL_8438091, EPI_ISL_8438093, EPI_ISL_8438094, EPI_ISL_8438097, EPI_ISL_8438098, EPI_ISL_8438100, EPI_ISL_8438102, EPI_ISL_8438103, EPI_ISL_8438104, EPI_ISL_8438105, EPI_ISL_8438108, EPI_ISL_8438110, EPI_ISL_8438112, EPI_ISL_8438113, EPI_ISL_8438116, EPI_ISL_8438118, EPI_ISL_8438119, EPI_ISL_8438124, EPI_ISL_8438126, EPI_ISL_8438127, EPI_ISL_8438128, EPI_ISL_8438130, EPI_ISL_8438132, EPI_ISL_8438133, EPI_ISL_8438135, EPI_ISL_8438137, EPI_ISL_8438138, EPI_ISL_8438140, EPI_ISL_8438141, EPI_ISL_8438143, EPI_ISL_8438144, EPI_ISL_8438147, EPI_ISL_8438148, EPI_ISL_8438149, EPI_ISL_8438152, EPI_ISL_8438155, EPI_ISL_8438156, EPI_ISL_8438157, EPI_ISL_8438158, EPI_ISL_8438159, EPI_ISL_8438160, EPI_ISL_8438161, EPI_ISL_8438164, EPI_ISL_8438169, EPI_ISL_8438172, EPI_ISL_8438173, EPI_ISL_8438174, EPI_ISL_8438175, EPI_ISL_8438177, EPI_ISL_8438178, EPI_ISL_8438179, EPI_ISL_8438183, EPI_ISL_8438184, EPI_ISL_8438185, EPI_ISL_8438186, EPI_ISL_8438188, EPI_ISL_8438189, EPI_ISL_8438190, EPI_ISL_8438193, EPI_ISL_8438194, EPI_ISL_8438198, EPI_ISL_8438207, EPI_ISL_8438210, EPI_ISL_8438211, EPI_ISL_8438216, EPI_ISL_8438219, EPI_ISL_8438220, EPI_ISL_8438222, EPI_ISL_8438224, EPI_ISL_8438227, EPI_ISL_8438229, EPI_ISL_8438231, EPI_ISL_8438234, EPI_ISL_8438235, EPI_ISL_8438236, EPI_ISL_8438240, EPI_ISL_8438241, EPI_ISL_8438246, EPI_ISL_8438248, EPI_ISL_8438249, EPI_ISL_8438251, EPI_ISL_8438254, EPI_ISL_8438257, EPI_ISL_8438258, EPI_ISL_8438262, EPI_ISL_8438263, EPI_ISL_8438264, EPI_ISL_8438270, EPI_ISL_8438272, EPI_ISL_8438274, EPI_ISL_8438276, EPI_ISL_8438277, EPI_ISL_8438278, EPI_ISL_8438279, EPI_ISL_8438281, EPI_ISL_8438283, EPI_ISL_8438284, EPI_ISL_8438286, EPI_ISL_8438287, EPI_ISL_8438288, EPI_ISL_8438289, EPI_ISL_8438290, EPI_ISL_8438292, EPI_ISL_8438293, EPI_ISL_8438294, EPI_ISL_8438295, EPI_ISL_8438296, EPI_ISL_8438299, EPI_ISL_8438301, EPI_ISL_8438302, EPI_ISL_8438303, EPI_ISL_8438304, EPI_ISL_8438305, EPI_ISL_8438306, EPI_ISL_8438307, EPI_ISL_8438308, EPI_ISL_8438309, EPI_ISL_8438310, EPI_ISL_8438311, EPI_ISL_8438312, EPI_ISL_8438313, EPI_ISL_8438314, EPI_ISL_8438315, EPI_ISL_8438316, EPI_ISL_8438317, EPI_ISL_8438318, EPI_ISL_8438319, EPI_ISL_8438321, EPI_ISL_8438322, EPI_ISL_8438323, EPI_ISL_8438324, EPI_ISL_8438325, EPI_ISL_8438326, EPI_ISL_8438327, EPI_ISL_8438328, EPI_ISL_8438329, EPI_ISL_8438330, EPI_ISL_8438331, EPI_ISL_8438333, EPI_ISL_8438334, EPI_ISL_8438335, EPI_ISL_8438336, EPI_ISL_8438428, EPI_ISL_8438429, EPI_ISL_8438430, EPI_ISL_8438432, EPI_ISL_8438436, EPI_ISL_8438438, EPI_ISL_8438439, EPI_ISL_8438441, EPI_ISL_8438443, EPI_ISL_8438445, EPI_ISL_8438449, EPI_ISL_8438451, EPI_ISL_8438454, EPI_ISL_8438458, EPI_ISL_8438464, EPI_ISL_8438465, EPI_ISL_8438469			
see above	PHV-FSS	PHV-FSS	Chenwei Wang on behalf of Q-PHIRE Genomics

We gratefully acknowledge the following Authors from the Originating laboratories responsible for obtaining the specimens, as well as the Submitting laboratories where the genome data were generated and shared via GISAID, on which this research is based.

All Submitters of data may be contacted directly via www.gisaid.org

Authors are sorted alphabetically.

Accession ID	Originating Laboratory	Submitting Laboratory	Authors
EPI_ISL_9028453, EPI_ISL_9028454, EPI_ISL_9028455, EPI_ISL_9028456, EPI_ISL_9028457, EPI_ISL_9028458, EPI_ISL_9028459, EPI_ISL_9028460, EPI_ISL_9028461, EPI_ISL_9028462, EPI_ISL_9028463, EPI_ISL_9028464, EPI_ISL_9028465, EPI_ISL_9028466, EPI_ISL_9028467, EPI_ISL_9028468, EPI_ISL_9028469, EPI_ISL_9028470, EPI_ISL_9028471, EPI_ISL_9028472, EPI_ISL_9028473, EPI_ISL_9028474, EPI_ISL_9028475, EPI_ISL_9028477, EPI_ISL_9028478, EPI_ISL_9028480, EPI_ISL_9028481, EPI_ISL_9028482, EPI_ISL_9028483, EPI_ISL_9028484, EPI_ISL_9028485, EPI_ISL_9028486, EPI_ISL_9028487, EPI_ISL_9028488, EPI_ISL_9028489, EPI_ISL_9028490, EPI_ISL_9028491, EPI_ISL_9028492, EPI_ISL_9028493, EPI_ISL_9028495			
see above	Ministry of Public Health / Hamad Medical Corporation	Biomedical Research Center (BRC), Qatar University / Qatar Genome Project (QGP)	Asmaa A. Al-Thani. MOPH and HMC: Abdullatif Al-Khal; BRC: Heba A. Al-Khatib; Chadi Saad; Dana Al-Batsh; Dina Elgakhlab. QGP: Fatima H. Al-Kuwari; Einas A. E. Al-Kuwari; Fatiha M. Benslimane; Fatma Hassan Ali; Hadeel T. Mohammed; Hadi M. Yassine; Hamad E. Al-Romaihi; Hamda Alromaihi; Maria K. Smatti; Mashael A. Al-Bader; Mohammed Al-Thani; Muna A. S. Al-Maslamani; Ola Al-Jamal; Peter V. Coyle; Reham A. El-Kahlout. QBB: Tasneem Al-Hamad; Roberto Bertollini; Salih Al-Marri; Swapna Thomas
EPI_ISL_9019278, EPI_ISL_9019279	RS Ciputra Citra Garden	Genomik Solidaritas Indonesia Laboratorium	Aisyi Sakina Rifani; Annisa Muthiah Sukirman; Anuraj Shankar; Ariel Pradipta; Carissa Sintca Wijaya; Dian Tri Wahyudi; Esti Rahayuning Tias; Filly Mandalie; Gracia Felias Enos Korompis; Louisa Markus; Meutia Ayuputeri Kumaheri; Stefanus Bernard

We gratefully acknowledge the following Authors from the Originating laboratories responsible for obtaining the specimens, as well as the Submitting laboratories where the genome data were generated and shared via GISAID, on which this research is based.

All Submitters of data may be contacted directly via www.gisaid.org

Authors are sorted alphabetically.

Acknowledgement EPI_SET Identifier: EPI_SET_20220527rx

Accession ID	Originating Laboratory	Submitting Laboratory	Authors
EPI_ISL_9082856, EPI_ISL_9082858, EPI_ISL_9082859	CAPRISA	CERI, Centre for Epidemic Response and Innvoation, Stellenbosch University and KRISP, KZN Research Innovation and Sequencing Platform, UKZN.	Aida Sivro; Giandhari J; Naidoo Y; Natasha Samsunder; Pillay S; Ramphal U; Ramphal Y; San JE; Tegally H; Tshiabuila D; Wilkinson E; de Oliveira T; van Wyk S

We gratefully acknowledge the following Authors from the Originating laboratories responsible for obtaining the specimens, as well as the Submitting laboratories where the genome data were generated and shared via GISAID, on which this research is based.

All Submitters of data may be contacted directly via www.gisaid.org

Authors are sorted alphabetically.

Accession ID	Originating Laboratory	Submitting Laboratory	Authors
EPI_ISL_9072095	Allergy, Immunology and Cell Biology Unit (AICBU)	Allergy, Immunology and Cell Biology Unit (AICBU)	Ayesha Wijesinghe; Chandima Jeewandara; Deshni Jayathilaka; Dinuka Ariyaratne; Diyanath Ranasinghe; Dumni Guasinghe; Farha Bary; Gathsaurie Neelika Malavige; Heshan Kuruppu; Tibutius Thanesh
EPI_ISL_9076079, EPI_ISL_9076080, EPI_ISL_9076081, EPI_ISL_9076082, EPI_ISL_9076083, EPI_ISL_9076084, EPI_ISL_9076086, EPI_ISL_9076087, EPI_ISL_9076088, EPI_ISL_9076089, EPI_ISL_9076090, EPI_ISL_9076091, EPI_ISL_9076092, EPI_ISL_9076093, EPI_ISL_9076094, EPI_ISL_9076096, EPI_ISL_9076097, EPI_ISL_9076098, EPI_ISL_9076099, EPI_ISL_9076100, EPI_ISL_9076101, EPI_ISL_9076102, EPI_ISL_9076103, EPI_ISL_9076104, EPI_ISL_9076105, EPI_ISL_9076106, EPI_ISL_9076107, EPI_ISL_9076108, EPI_ISL_9076109			
see above	National Public Health Laboratory, National Centre for Infectious Diseases	National Public Health Laboratory, National Centre for Infectious Diseases	Benny Yeo; Grace Ngan; Lin Cui; Raymond Tzer Pin Lin; Royce Ang; Samuel Loo; Yichen Ding; Zhenyang Zhou

We gratefully acknowledge the following Authors from the Originating laboratories responsible for obtaining the specimens, as well as the Submitting laboratories where the genome data were generated and shared via GISAID, on which this research is based.

All Submitters of data may be contacted directly via www.gisaid.org

Authors are sorted alphabetically.

Acknowledgement EPI_SET Identifier: EPI_SET_20220527ed

Accession ID	Originating Laboratory	Submitting Laboratory	Authors
EPI_ISL_9064941	CHUV	Laboratory of genomics and metagenomics, Institute of Microbiology, University Hospital Centre and University of Lausanne	Claire Bertelli; Damien Jacot; Gilbert Greub; Sébastien Aeby; Trestan Pillonel
EPI_ISL_9063244, EPI_ISL_9063246, EPI_ISL_9063254, EPI_ISL_9063257, EPI_ISL_9063268, EPI_ISL_9063297, EPI_ISL_9063306, EPI_ISL_9063308, EPI_ISL_9063319, EPI_ISL_9063329, EPI_ISL_9063335, EPI_ISL_9063342, EPI_ISL_9063345, EPI_ISL_9063346, EPI_ISL_9063350, EPI_ISL_9063352, EPI_ISL_9063367, EPI_ISL_9063378, EPI_ISL_9063380, EPI_ISL_9063385, EPI_ISL_9063390, EPI_ISL_9063395, EPI_ISL_9063401, EPI_ISL_9063415, EPI_ISL_9063418, EPI_ISL_9063421, EPI_ISL_9063423, EPI_ISL_9063452, EPI_ISL_9063455, EPI_ISL_9063457, EPI_ISL_9063468, EPI_ISL_9063480, EPI_ISL_9063482, EPI_ISL_9063485, EPI_ISL_9063490, EPI_ISL_9063493, EPI_ISL_9063503, EPI_ISL_9063506, EPI_ISL_9063509, EPI_ISL_9063514, EPI_ISL_9063517, EPI_ISL_9063522, EPI_ISL_9063533, EPI_ISL_9063536, EPI_ISL_9063547, EPI_ISL_9063549, EPI_ISL_9063552, EPI_ISL_9063555, EPI_ISL_9063558, EPI_ISL_9063560, EPI_ISL_9063565, EPI_ISL_9063567, EPI_ISL_9063570, EPI_ISL_9063573, EPI_ISL_9063577, EPI_ISL_9063580, EPI_ISL_9063586, EPI_ISL_9063591, EPI_ISL_9063596, EPI_ISL_9063601, EPI_ISL_9063606, EPI_ISL_9063610, EPI_ISL_9063612, EPI_ISL_9063618, EPI_ISL_9063623, EPI_ISL_9063636, EPI_ISL_9063637, EPI_ISL_9063640, EPI_ISL_9063644, EPI_ISL_9063649, EPI_ISL_9063651, EPI_ISL_9063663, EPI_ISL_9063665, EPI_ISL_9063682, EPI_ISL_9063684, EPI_ISL_9063688, EPI_ISL_9063693, EPI_ISL_9063696, EPI_ISL_9063707, EPI_ISL_9063715, EPI_ISL_9063720, EPI_ISL_9063726, EPI_ISL_9063729, EPI_ISL_9063731, EPI_ISL_9063732, EPI_ISL_9063733, EPI_ISL_9063734, EPI_ISL_9063738, EPI_ISL_9063739, EPI_ISL_9063740, EPI_ISL_9063744, EPI_ISL_9063753, EPI_ISL_9063761, EPI_ISL_9063763, EPI_ISL_9063770, EPI_ISL_9063773, EPI_ISL_9063782, EPI_ISL_9063785, EPI_ISL_9063807, EPI_ISL_9063813, EPI_ISL_9063816, EPI_ISL_9063820, EPI_ISL_9063823, EPI_ISL_9063828, EPI_ISL_9063835, EPI_ISL_9063838, EPI_ISL_9063850, EPI_ISL_9063853, EPI_ISL_9063856, EPI_ISL_9063859, EPI_ISL_9063861, EPI_ISL_9063868, EPI_ISL_9063874, EPI_ISL_9063880, EPI_ISL_9063888, EPI_ISL_9063892, EPI_ISL_9063903, EPI_ISL_9063905, EPI_ISL_9063909, EPI_ISL_9063911, EPI_ISL_9063917, EPI_ISL_9063920, EPI_ISL_9063931, EPI_ISL_9063935, EPI_ISL_9063938, EPI_ISL_9063951, EPI_ISL_9063957, EPI_ISL_9063972, EPI_ISL_9063978, EPI_ISL_9063984, EPI_ISL_9063990, EPI_ISL_9063992, EPI_ISL_9063999, EPI_ISL_9064001, EPI_ISL_9064006, EPI_ISL_9064010, EPI_ISL_9064024, EPI_ISL_9064029, EPI_ISL_9064032, EPI_ISL_9064035, EPI_ISL_9064038, EPI_ISL_9064044, EPI_ISL_9064047, EPI_ISL_9064053, EPI_ISL_9064056, EPI_ISL_9064058, EPI_ISL_9064061, EPI_ISL_9064063, EPI_ISL_9064067, EPI_ISL_9064087, EPI_ISL_9064108, EPI_ISL_9064114, EPI_ISL_9064116, EPI_ISL_9064158, EPI_ISL_9064160, EPI_ISL_9064166, EPI_ISL_9064167, EPI_ISL_9064171, EPI_ISL_9064196, EPI_ISL_9064199, EPI_ISL_9064206, EPI_ISL_9064209, EPI_ISL_9064216, EPI_ISL_9064217, EPI_ISL_9064248, EPI_ISL_9064258, EPI_ISL_9064263, EPI_ISL_9064274, EPI_ISL_9064278, EPI_ISL_9064286, EPI_ISL_9064287, EPI_ISL_9064296, EPI_ISL_9064299, EPI_ISL_9064308, EPI_ISL_9064309, EPI_ISL_9064310, EPI_ISL_9064320, EPI_ISL_9064323, EPI_ISL_9064325, EPI_ISL_9064330, EPI_ISL_9064333, EPI_ISL_9064338, EPI_ISL_9064341, EPI_ISL_9064343, EPI_ISL_9064355, EPI_ISL_9064361, EPI_ISL_9064363, EPI_ISL_9064365, EPI_ISL_9064368, EPI_ISL_9064386, EPI_ISL_9064391, EPI_ISL_9064401, EPI_ISL_9064403, EPI_ISL_9064409, EPI_ISL_9064413, EPI_ISL_9064418, EPI_ISL_9064421, EPI_ISL_9064425, EPI_ISL_9064428, EPI_ISL_9064430, EPI_ISL_9064442, EPI_ISL_9064451, EPI_ISL_9064455, EPI_ISL_9064466, EPI_ISL_9064470, EPI_ISL_9064478, EPI_ISL_9064488, EPI_ISL_9064502, EPI_ISL_9064505, EPI_ISL_9064527, EPI_ISL_9064530, EPI_ISL_9064534, EPI_ISL_9064536, EPI_ISL_9064548, EPI_ISL_9064554, EPI_ISL_9064563, EPI_ISL_9064565, EPI_ISL_9064569, EPI_ISL_9064572, EPI_ISL_9064574, EPI_ISL_9064584, EPI_ISL_9064587, EPI_ISL_9064609, EPI_ISL_9064614, EPI_ISL_9064623, EPI_ISL_9064632, EPI_ISL_9064644, EPI_ISL_9064649, EPI_ISL_9064653, EPI_ISL_9064664, EPI_ISL_9064666, EPI_ISL_9064678, EPI_ISL_9064684, EPI_ISL_9064687, EPI_ISL_9064701, EPI_ISL_9064708, EPI_ISL_9064711, EPI_ISL_9064717, EPI_ISL_9064723, EPI_ISL_9064726, EPI_ISL_9064731, EPI_ISL_9064737, EPI_ISL_9064742, EPI_ISL_9064768, EPI_ISL_9064774, EPI_ISL_9064786, EPI_ISL_9064792, EPI_ISL_9064805, EPI_ISL_9064810, EPI_ISL_9064837, EPI_ISL_9064863, EPI_ISL_9064865, EPI_ISL_9064867, EPI_ISL_9064870, EPI_ISL_9064873, EPI_ISL_9064880, EPI_ISL_9064882, EPI_ISL_9064885, EPI_ISL_9064888, EPI_ISL_9064897, EPI_ISL_9064903, EPI_ISL_9064908, EPI_ISL_9064916, EPI_ISL_9064919, EPI_ISL_9064939, EPI_ISL_9064943, EPI_ISL_9064946, EPI_ISL_9064953, EPI_ISL_9064957, EPI_ISL_9064960, EPI_ISL_9064965, EPI_ISL_9064978, EPI_ISL_9064990, EPI_ISL_9064997, EPI_ISL_9065006, EPI_ISL_9065043, EPI_ISL_9065047, EPI_ISL_9065056, EPI_ISL_9065059, EPI_ISL_9065063, EPI_ISL_9065074, EPI_ISL_9065077, EPI_ISL_9065084, EPI_ISL_9065090, EPI_ISL_9065093, EPI_ISL_9065096, EPI_ISL_9065108, EPI_ISL_9065121, EPI_ISL_9065123, EPI_ISL_9065128, EPI_ISL_9065133, EPI_ISL_9065137, EPI_ISL_9065140, EPI_ISL_9065143, EPI_ISL_9065147, EPI_ISL_9065150, EPI_ISL_9065158, EPI_ISL_9065161, EPI_ISL_9065170, EPI_ISL_9065186, EPI_ISL_9065191, EPI_ISL_9065198, EPI_ISL_9065201, EPI_ISL_9065207, EPI_ISL_9065212, EPI_ISL_9065227, EPI_ISL_9065234, EPI_ISL_9065237, EPI_ISL_9065255, EPI_ISL_9065258, EPI_ISL_9065275, EPI_ISL_9065277, EPI_ISL_9065280, EPI_ISL_9065294, EPI_ISL_9065303, EPI_ISL_9065331, EPI_ISL_9065334, EPI_ISL_9065336, EPI_ISL_9065339, EPI_ISL_9065344, EPI_ISL_9065346, EPI_ISL_9065349, EPI_ISL_9065351, EPI_ISL_9065353, EPI_ISL_9065364, EPI_ISL_9065374, EPI_ISL_9065402, EPI_ISL_9065419, EPI_ISL_9065424, EPI_ISL_9065427, EPI_ISL_9065433, EPI_ISL_9065440, EPI_ISL_9065453, EPI_ISL_9065458, EPI_ISL_9065461, EPI_ISL_9065464, EPI_ISL_9065474, EPI_ISL_9065478, EPI_ISL_9065484, EPI_ISL_9065486, EPI_ISL_9065489, EPI_ISL_9065506, EPI_ISL_9065508, EPI_ISL_9065514, EPI_ISL_9065522, EPI_ISL_9065543, EPI_ISL_9065548, EPI_ISL_9065554, EPI_ISL_9065569, EPI_ISL_9065571, EPI_ISL_9065577, EPI_ISL_9065581, EPI_ISL_9065594, EPI_ISL_9065597, EPI_ISL_9065603, EPI_ISL_9065606, EPI_ISL_9065608, EPI_ISL_9065610, EPI_ISL_9065612, EPI_ISL_9065613, EPI_ISL_9065614, EPI_ISL_9065634, EPI_ISL_9065639, EPI_ISL_9065642, EPI_ISL_9065659, EPI_ISL_9065671, EPI_ISL_9065677, EPI_ISL_9065693, EPI_ISL_9065695, EPI_ISL_9065707, EPI_ISL_9065710, EPI_ISL_9065711, EPI_ISL_9065713, EPI_ISL_9065720, EPI_ISL_9065726, EPI_ISL_9065728, EPI_ISL_9065730, EPI_ISL_9065732, EPI_ISL_9065743			
see above	Department of Bacteria, Parasites and Fungi, Statens Serum Institut, Copenhagen, Denmark	Statens Serum Institut Bioinformatics and Microbial Genomics	Danish Covid-19 Genome Consortium
EPI_ISL_9058354, EPI_ISL_9058356, EPI_ISL_9058361, EPI_ISL_9058364, EPI_ISL_9058370, EPI_ISL_9058376, EPI_ISL_9058380, EPI_ISL_9058389, EPI_ISL_9058390, EPI_ISL_9058394, EPI_ISL_9058399, EPI_ISL_9058402, EPI_ISL_9058407, EPI_ISL_9058408, EPI_ISL_9058410, EPI_ISL_9058411, EPI_ISL_9058414, EPI_ISL_9058417, EPI_ISL_9058418, EPI_ISL_9058427, EPI_ISL_9058428, EPI_ISL_9058429, EPI_ISL_9058431, EPI_ISL_9058433, EPI_ISL_9058436, EPI_ISL_9058462, EPI_ISL_9058464, EPI_ISL_9058465, EPI_ISL_9058466, EPI_ISL_9058467, EPI_ISL_9058468, EPI_ISL_9058470, EPI_ISL_9058471, EPI_ISL_9058473, EPI_ISL_9058475, EPI_ISL_9058476, EPI_ISL_9058477, EPI_ISL_9058478, EPI_ISL_9058479, EPI_ISL_9058480, EPI_ISL_9058481, EPI_ISL_9058483, EPI_ISL_9058486, EPI_ISL_9058488, EPI_ISL_9058490, EPI_ISL_9058497, EPI_ISL_9058506, EPI_ISL_9058513, EPI_ISL_9058515, EPI_ISL_9058516, EPI_ISL_9058530, EPI_ISL_9058540, EPI_ISL_9058543, EPI_ISL_9058544, EPI_ISL_9058546, EPI_ISL_9058547, EPI_ISL_9058548, EPI_ISL_9058549, EPI_ISL_9058550, EPI_ISL_9058551, EPI_ISL_9058554, EPI_ISL_9058555, EPI_ISL_9058557, EPI_ISL_9058558, EPI_ISL_9058559, EPI_ISL_9058561, EPI_ISL_9058562, EPI_ISL_9058563, EPI_ISL_9058564, EPI_ISL_9058606, EPI_ISL_9058607, EPI_ISL_9058608, EPI_ISL_9058609, EPI_ISL_9058610, EPI_ISL_9058613, EPI_ISL_9058614, EPI_ISL_9058617, EPI_ISL_9058618, EPI_ISL_9058622, EPI_ISL_9058627			
see above	Lab. Microbiologia e Virologia Cotugno A.O. dei Colli	TIGEM	Antonio Grimaldi Patrizia Annunziata Francesco Panariello Claudia Tiberio Teresa Giuliano Valentina Bouche Chiara Colantuono Michela Daniele Lucio Di Filippo Anna Manfredi Marcello Salvi Antonio Limone Luigi Atripaldi Andrea Ballabio Davide Cacchiarelli
EPI_ISL_9061587	Respiratory Virus Unit, Microbiology Services Colindale, Public Health England	COVID-19 Genomics UK (COG-UK) Consortium	PHE Covid Sequencing Team

We gratefully acknowledge the following Authors from the Originating laboratories responsible for obtaining the specimens, as well as the Submitting laboratories where the genome data were generated and shared via GISAID, on which this research is based.

All Submitters of data may be contacted directly via www.gisaid.org

Authors are sorted alphabetically.

Acknowledgement EPI_SET Identifier: EPI_SET_20220527sy

Accession ID	Originating Laboratory	Submitting Laboratory	Authors
EPI_ISL_9065750, EPI_ISL_9065758, EPI_ISL_9065760, EPI_ISL_9065762, EPI_ISL_9065764, EPI_ISL_9065770, EPI_ISL_9065773, EPI_ISL_9065788, EPI_ISL_9065791, EPI_ISL_9065794, EPI_ISL_9065800, EPI_ISL_9065810, EPI_ISL_9065812, EPI_ISL_9065821, EPI_ISL_9065828, EPI_ISL_9065834, EPI_ISL_9065835, EPI_ISL_9065838, EPI_ISL_9065849, EPI_ISL_9065851, EPI_ISL_9065859, EPI_ISL_9065862, EPI_ISL_9065870, EPI_ISL_9065898, EPI_ISL_9065902, EPI_ISL_9065903, EPI_ISL_9065906, EPI_ISL_9065912, EPI_ISL_9065915, EPI_ISL_9065923, EPI_ISL_9065925, EPI_ISL_9065929, EPI_ISL_9065936, EPI_ISL_9065961, EPI_ISL_9065962, EPI_ISL_9065964, EPI_ISL_9065967, EPI_ISL_9065971, EPI_ISL_9065973, EPI_ISL_9065974, EPI_ISL_9065977, EPI_ISL_9065978, EPI_ISL_9065979, EPI_ISL_9065984, EPI_ISL_9065993, EPI_ISL_9065998, EPI_ISL_9066000, EPI_ISL_9066002, EPI_ISL_9066011, EPI_ISL_9066024, EPI_ISL_9066027, EPI_ISL_9066030, EPI_ISL_9066032, EPI_ISL_9066044, EPI_ISL_9066050, EPI_ISL_9066057, EPI_ISL_9066064, EPI_ISL_9066088, EPI_ISL_9066099, EPI_ISL_9066102, EPI_ISL_9066105, EPI_ISL_9066113, EPI_ISL_9066118, EPI_ISL_9066122, EPI_ISL_9066127, EPI_ISL_9066138, EPI_ISL_9066149, EPI_ISL_9066158, EPI_ISL_9066164, EPI_ISL_9066167, EPI_ISL_9066172, EPI_ISL_9066183, EPI_ISL_9066186, EPI_ISL_9066191, EPI_ISL_9066194, EPI_ISL_9066196, EPI_ISL_9066205, EPI_ISL_9066208, EPI_ISL_9066222, EPI_ISL_9066239, EPI_ISL_9066242, EPI_ISL_9066247, EPI_ISL_9066250, EPI_ISL_9066256, EPI_ISL_9066261, EPI_ISL_9066269, EPI_ISL_9066275, EPI_ISL_9066290, EPI_ISL_9066299, EPI_ISL_9066301, EPI_ISL_9066307, EPI_ISL_9066309, EPI_ISL_9066320, EPI_ISL_9066323, EPI_ISL_9066326, EPI_ISL_9066332, EPI_ISL_9066338, EPI_ISL_9066350, EPI_ISL_9066359, EPI_ISL_9066362, EPI_ISL_9066368, EPI_ISL_9066370, EPI_ISL_9066373, EPI_ISL_9066375, EPI_ISL_9066377, EPI_ISL_9066380, EPI_ISL_9066396, EPI_ISL_9066399, EPI_ISL_9066402, EPI_ISL_9066404, EPI_ISL_9066406, EPI_ISL_9066409, EPI_ISL_9066411, EPI_ISL_9066428, EPI_ISL_9066433, EPI_ISL_9066436, EPI_ISL_9066437, EPI_ISL_9066441, EPI_ISL_9066442, EPI_ISL_9066444, EPI_ISL_9066445, EPI_ISL_9066464, EPI_ISL_9066477, EPI_ISL_9066483, EPI_ISL_9066489, EPI_ISL_9066492, EPI_ISL_9066495, EPI_ISL_9066499, EPI_ISL_9066504, EPI_ISL_9066510, EPI_ISL_9066511, EPI_ISL_9066514, EPI_ISL_9066521, EPI_ISL_9066527, EPI_ISL_9066530, EPI_ISL_9066533, EPI_ISL_9066535, EPI_ISL_9066548, EPI_ISL_9066552, EPI_ISL_9066555, EPI_ISL_9066558, EPI_ISL_9066563, EPI_ISL_9066572, EPI_ISL_9066579, EPI_ISL_9066581, EPI_ISL_9066584, EPI_ISL_9066587, EPI_ISL_9066600, EPI_ISL_9066609, EPI_ISL_9066612, EPI_ISL_9066618, EPI_ISL_9066626, EPI_ISL_9066629, EPI_ISL_9066632, EPI_ISL_9066640, EPI_ISL_9066645, EPI_ISL_9066648, EPI_ISL_9066657, EPI_ISL_9066661, EPI_ISL_9066664, EPI_ISL_9066667, EPI_ISL_9066670, EPI_ISL_9066684, EPI_ISL_9066687, EPI_ISL_9066690, EPI_ISL_9066695, EPI_ISL_9066704, EPI_ISL_9066706, EPI_ISL_9066720, EPI_ISL_9066725, EPI_ISL_9066744, EPI_ISL_9066747, EPI_ISL_9066750, EPI_ISL_9066754, EPI_ISL_9066763, EPI_ISL_9066773, EPI_ISL_9066777, EPI_ISL_9066780, EPI_ISL_9066782, EPI_ISL_9066785, EPI_ISL_9066791, EPI_ISL_9066794, EPI_ISL_9066804, EPI_ISL_9066807, EPI_ISL_9066813, EPI_ISL_9066816, EPI_ISL_9066824, EPI_ISL_9066826, EPI_ISL_9066831, EPI_ISL_9066834, EPI_ISL_9066837, EPI_ISL_9066845, EPI_ISL_9066848, EPI_ISL_9066850, EPI_ISL_9066858, EPI_ISL_9066868, EPI_ISL_9066871, EPI_ISL_9066883, EPI_ISL_9066888, EPI_ISL_9066890, EPI_ISL_9066897, EPI_ISL_9066903, EPI_ISL_9066906, EPI_ISL_9066916, EPI_ISL_9066924, EPI_ISL_9066927, EPI_ISL_9066941, EPI_ISL_9066949, EPI_ISL_9066952, EPI_ISL_9066960, EPI_ISL_9066968, EPI_ISL_9066973, EPI_ISL_9066976, EPI_ISL_9066982, EPI_ISL_9066984, EPI_ISL_9066987, EPI_ISL_9066996, EPI_ISL_9067002, EPI_ISL_9067005, EPI_ISL_9067013, EPI_ISL_9067016, EPI_ISL_9067022, EPI_ISL_9067028, EPI_ISL_9067031, EPI_ISL_9067033, EPI_ISL_9067042, EPI_ISL_9067048, EPI_ISL_9067051, EPI_ISL_9067053, EPI_ISL_9067056, EPI_ISL_9067073, EPI_ISL_9067090, EPI_ISL_9067097, EPI_ISL_9067100, EPI_ISL_9067102, EPI_ISL_9067123, EPI_ISL_9067130, EPI_ISL_9067133, EPI_ISL_9067135, EPI_ISL_9067140, EPI_ISL_9067143, EPI_ISL_9067145, EPI_ISL_9067148, EPI_ISL_9067156, EPI_ISL_9067159, EPI_ISL_9067166, EPI_ISL_9067172, EPI_ISL_9067174, EPI_ISL_9067175, EPI_ISL_9067178, EPI_ISL_9067191, EPI_ISL_9067199, EPI_ISL_9067205, EPI_ISL_9067210, EPI_ISL_9067221, EPI_ISL_9067229, EPI_ISL_9067232, EPI_ISL_9067235, EPI_ISL_9067243, EPI_ISL_9067245, EPI_ISL_9067246, EPI_ISL_9067249, EPI_ISL_9067251, EPI_ISL_9067257, EPI_ISL_9067258, EPI_ISL_9067259, EPI_ISL_9067263, EPI_ISL_9067266, EPI_ISL_9067269, EPI_ISL_9067273, EPI_ISL_9067275, EPI_ISL_9067276, EPI_ISL_9067277, EPI_ISL_9067278, EPI_ISL_9067291, EPI_ISL_9067300, EPI_ISL_9067317, EPI_ISL_9067320, EPI_ISL_9067328, EPI_ISL_9067331, EPI_ISL_9067333, EPI_ISL_9067336, EPI_ISL_9067338, EPI_ISL_9067341, EPI_ISL_9067346, EPI_ISL_9067351, EPI_ISL_9067353, EPI_ISL_9067358, EPI_ISL_9067361, EPI_ISL_9067367, EPI_ISL_9067370, EPI_ISL_9067378, EPI_ISL_9067381, EPI_ISL_9067383, EPI_ISL_9067388, EPI_ISL_9067394, EPI_ISL_9067412, EPI_ISL_9067415, EPI_ISL_9067418, EPI_ISL_9067424, EPI_ISL_9067519, EPI_ISL_9067522, EPI_ISL_9067525, EPI_ISL_9067531, EPI_ISL_9067534, EPI_ISL_9067536, EPI_ISL_9067551, EPI_ISL_9067554, EPI_ISL_9067556, EPI_ISL_9067559, EPI_ISL_9067562, EPI_ISL_9067564, EPI_ISL_9067570, EPI_ISL_9067576, EPI_ISL_9067578, EPI_ISL_9067589, EPI_ISL_9067595, EPI_ISL_9067598, EPI_ISL_9067603, EPI_ISL_9067606, EPI_ISL_9067610, EPI_ISL_9067614, EPI_ISL_9067620, EPI_ISL_9067625, EPI_ISL_9067631, EPI_ISL_9067634, EPI_ISL_9067651, EPI_ISL_9067652, EPI_ISL_9067664, EPI_ISL_9067672, EPI_ISL_9067684, EPI_ISL_9067687, EPI_ISL_9067693, EPI_ISL_9067696, EPI_ISL_9067702, EPI_ISL_9067708, EPI_ISL_9067714, EPI_ISL_9067717, EPI_ISL_9067726, EPI_ISL_9067729, EPI_ISL_9067732, EPI_ISL_9067738, EPI_ISL_9067758, EPI_ISL_9067761, EPI_ISL_9067764, EPI_ISL_9067769, EPI_ISL_9067772, EPI_ISL_9067773, EPI_ISL_9067778, EPI_ISL_9067781, EPI_ISL_9067784, EPI_ISL_9067786, EPI_ISL_9067803, EPI_ISL_9067806, EPI_ISL_9067809, EPI_ISL_9067814, EPI_ISL_9067817, EPI_ISL_9067822, EPI_ISL_9067824, EPI_ISL_9067836, EPI_ISL_9067855, EPI_ISL_9067858, EPI_ISL_9067864, EPI_ISL_9067868, EPI_ISL_9067871, EPI_ISL_9067885, EPI_ISL_9067888, EPI_ISL_9067894, EPI_ISL_9067904, EPI_ISL_9067907, EPI_ISL_9067922, EPI_ISL_9067925, EPI_ISL_9067937, EPI_ISL_9067945, EPI_ISL_9067947, EPI_ISL_9067967, EPI_ISL_9067973, EPI_ISL_9067976, EPI_ISL_9067981, EPI_ISL_9067984, EPI_ISL_9067987, EPI_ISL_9067989, EPI_ISL_9067992, EPI_ISL_9068009, EPI_ISL_9068011, EPI_ISL_9068014, EPI_ISL_9068017, EPI_ISL_9068020, EPI_ISL_9068023, EPI_ISL_9068025, EPI_ISL_9068028, EPI_ISL_9068031, EPI_ISL_9068036, EPI_ISL_9068051, EPI_ISL_9068054, EPI_ISL_9068060, EPI_ISL_9068066, EPI_ISL_9068077, EPI_ISL_9068084, EPI_ISL_9068086, EPI_ISL_9068090, EPI_ISL_9068093, EPI_ISL_9068110, EPI_ISL_9068128, EPI_ISL_9068129, EPI_ISL_9068131, EPI_ISL_9068133, EPI_ISL_9068135, EPI_ISL_9068155, EPI_ISL_9068164, EPI_ISL_9068167, EPI_ISL_9068218, EPI_ISL_9068221, EPI_ISL_9068226, EPI_ISL_9068228, EPI_ISL_9068235, EPI_ISL_9068240, EPI_ISL_9068241, EPI_ISL_9068242, EPI_ISL_9068243, EPI_ISL_9068244, EPI_ISL_9068247, EPI_ISL_9068252, EPI_ISL_9068253, EPI_ISL_9068257, EPI_ISL_9068258, EPI_ISL_9068262, EPI_ISL_9068263, EPI_ISL_9068265, EPI_ISL_9068268, EPI_ISL_9068269, EPI_ISL_9068271, EPI_ISL_9068273, EPI_ISL_9068274, EPI_ISL_9068277, EPI_ISL_9068279, EPI_ISL_9068282, EPI_ISL_9068287, EPI_ISL_9068296, EPI_ISL_9068298, EPI_ISL_9068304, EPI_ISL_9068310, EPI_ISL_9068313, EPI_ISL_9068316, EPI_ISL_9068328, EPI_ISL_9068334, EPI_ISL_9068340, EPI_ISL_9068343, EPI_ISL_9068346, EPI_ISL_9068348, EPI_ISL_9068354, EPI_ISL_9068357, EPI_ISL_9068369, EPI_ISL_9068371, EPI_ISL_9068374, EPI_ISL_9068406, EPI_ISL_9068409, EPI_ISL_9068411, EPI_ISL_9068417, EPI_ISL_9068419, EPI_ISL_9068425, EPI_ISL_9068435, EPI_ISL_9068438, EPI_ISL_9068447, EPI_ISL_9068451, EPI_ISL_9068456, EPI_ISL_9068464, EPI_ISL_9068468, EPI_ISL_9068471, EPI_ISL_9068476, EPI_ISL_9068479, EPI_ISL_9068485, EPI_ISL_9068493, EPI_ISL_9068497, EPI_ISL_9068500, EPI_ISL_9068509, EPI_ISL_9068513, EPI_ISL_9068519, EPI_ISL_9068522, EPI_ISL_9068528, EPI_ISL_9068541, EPI_ISL_9068547, EPI_ISL_9068549, EPI_ISL_9068553, EPI_ISL_9068558, EPI_ISL_9068561, EPI_ISL_9068567, EPI_ISL_9068570, EPI_ISL_9068572, EPI_ISL_9068575, EPI_ISL_9068579, EPI_ISL_9068582, EPI_ISL_9068588, EPI_ISL_9068593, EPI_ISL_9068604, EPI_ISL_9068609, EPI_ISL_9068620, EPI_ISL_9068622, EPI_ISL_9068626, EPI_ISL_9068632			
see above	Department of Bacteria, Parasites and Fungi, Statens Serum Institut, Copenhagen, Denmark	Statens Serum Institut Bioinformatics and Microbial Genomics	Danish Covid-19 Genome Consortium

We gratefully acknowledge the following Authors from the Originating laboratories responsible for obtaining the specimens, as well as the Submitting laboratories where the genome data were generated and shared via GISAID, on which this research is based.

All Submitters of data may be contacted directly via www.gisaid.org

Authors are sorted alphabetically.

Accession ID	Originating Laboratory	Submitting Laboratory	Authors
EPI_ISL_9080634, EPI_ISL_9080635, EPI_ISL_9085223	Dutch COVID-19 response team	National Institute for Public Health and the Environment (RIVM)	Adam Meijer; Afke Vogelzang; AnneMarie van den Brandt; Annelies Kroneman; Bas van der Veer; Chantal Reusken; Dennis Schmitz; Dirk Eggink; Florian Zwagemaker; Harry Vennema; Ivo van Walle; Jeroen Cremer; Jil Kocken; Jordy de Bakker; Karim Hajji; Kim Freriks; Linda van Someren; Lisa Wijsman; Lynn Aarts; Ryanne Jaarsma; Sanne Bos; Sharon van den Brink; on behalf of the national COVID-19 response team
EPI_ISL_9087313, EPI_ISL_9087316, EPI_ISL_9089615	Hopital	National Reference Center for Viruses of Respiratory Infections, Institut Pasteur, Paris	Angela Brisebarre; Camille Capel; Christophe Malabat; Corinne Maufrais; Etienne Simon-Lorière; Frédéric Lemoine; Julien Fumey; Louise Lefrançois; Mama Imene FODIL PACHA; Marion Barbet; Maud Vanpeene; Méline Bizard; Slim El Khiari; Sylvie Behillil; Sylvie Van der Werf; Vincent ESTEVE; Vincent Enouf
EPI_ISL_9089637	Labo Analyses Med	National Reference Center for Viruses of Respiratory Infections, Institut Pasteur, Paris	Angela Brisebarre; Camille Capel; Christophe Malabat; Corinne Maufrais; Etienne Simon-Lorière; Frédéric Lemoine; Julien Fumey; Louise Lefrançois; Marion Barbet; Maud Vanpeene; Méline Bizard; Slim El Khiari; Sylvie Behillil; Sylvie Van der Werf; Thierry GUFFOND; Vincent Enouf
EPI_ISL_9074304, EPI_ISL_9074867	MVZ Labor Dr. Fenner und Kollegen (Standort Hamburg)	Robert Koch Institute	
EPI_ISL_9083481	Platform BIS UZA/UAntwerpen	Labo Klinische Biologie, UZA	Basil Britto Xavier; Christine Lammens; Herman Goossens; Ines Verbesselt; Jasmine Coppens; Kathleen Holemans; Marie Le Mercier; Veerle Matheeussen
EPI_ISL_9084574, EPI_ISL_9084575, EPI_ISL_9084576, EPI_ISL_9084577, EPI_ISL_9084578, EPI_ISL_9084580, EPI_ISL_9084581, EPI_ISL_9084583, EPI_ISL_9084584, EPI_ISL_9084585			
see above	SARS-CoV-2 Sequencing Castilla y Leon-Spain Consortium	SARS-CoV-2 Sequencing Castilla y Leon-Spain Consortium	Antonio Orduña-Domingo; Carlos Fuster Foz; Carmen Aldea-Mansilla; Carmen Gimeno Crespo; David Abad; Gabriel March Rosello; Gregoria Megías Lobón; Jose María Eiros Bouza; M. Isabel Fernandez-Natal; Marta Dominguez-Gil; Marta Hernandez; María Antonia García Castro; Mª Fe Brezmes-Valdivieso; Noelia Arenal Andrés; Silvia Rojo; Sonsoles Garcinuño Pérez
EPI_ISL_9080382	SYNLAB MVZ Leverkusen	Robert Koch Institute	
EPI_ISL_9075006, EPI_ISL_9075007, EPI_ISL_9075008, EPI_ISL_9075009, EPI_ISL_9075010, EPI_ISL_9075011, EPI_ISL_9075012, EPI_ISL_9075013, EPI_ISL_9075014, EPI_ISL_9075015, EPI_ISL_9075016, EPI_ISL_9075017, EPI_ISL_9075018, EPI_ISL_9075019, EPI_ISL_9075020, EPI_ISL_9075021, EPI_ISL_9075022, EPI_ISL_9075023, EPI_ISL_9075024, EPI_ISL_9075025, EPI_ISL_9075026, EPI_ISL_9075027, EPI_ISL_9075028, EPI_ISL_9075029, EPI_ISL_9075030, EPI_ISL_9075031			
see above	University Clinical Hospital of Mostar, Department of Microbiology and Molecular Diagnostics	University of Sarajevo, Veterinary Faculty, Laboratory for Molecular Diagnostic and Research	Goletic S; Goletic T; Herceg I; Hodzic A; Jazic A; Nicevic M; Ostojic M; Sabic E; Skocibusic S; Softic A; Terzic I
EPI_ISL_9075003, EPI_ISL_9075004, EPI_ISL_9075005	University of Sarajevo, Veterinary Faculty, Laboratory for Molecular Diagnostic and Research	University of Sarajevo, Veterinary Faculty, Laboratory for Molecular Diagnostic and Research	Goletic S; Goletic T; Hodzic A; Jazic A; Nicevic M; Sabic E; Softic A; Terzic I

We gratefully acknowledge the following Authors from the Originating laboratories responsible for obtaining the specimens, as well as the Submitting laboratories where the genome data were generated and shared via GISAID, on which this research is based.

All Submitters of data may be contacted directly via www.gisaid.org

Authors are sorted alphabetically.

Accession ID	Originating Laboratory	Submitting Laboratory	Authors
EPI_ISL_9071873, EPI_ISL_9071899, EPI_ISL_9071904, EPI_ISL_9071938, EPI_ISL_9071941, EPI_ISL_9071943, EPI_ISL_9071944, EPI_ISL_9071958, EPI_ISL_9071965, EPI_ISL_9071977, EPI_ISL_9071986, EPI_ISL_9071996			
see above	Bayerisches Landesamt fuer Gesundheit und Lebensmittelsicherheit (LGL)	Bayerisches Landesamt fuer Gesundheit und Lebensmittelsicherheit (LGL)	Alexandra Dangel; Andreas Sing; Annika Sprenger; Carola Berger; Laura Weise; Nikolaus Ackermann; Sabrina Hepner
EPI_ISL_9070769, EPI_ISL_9070771, EPI_ISL_9070778, EPI_ISL_9070785, EPI_ISL_9070787, EPI_ISL_9070788, EPI_ISL_9070801, EPI_ISL_9070869, EPI_ISL_9070870, EPI_ISL_9070874, EPI_ISL_9070877, EPI_ISL_9070879, EPI_ISL_9070882, EPI_ISL_9070892, EPI_ISL_9070894, EPI_ISL_9070903, EPI_ISL_9070906, EPI_ISL_9070912, EPI_ISL_9070915, EPI_ISL_9070917, EPI_ISL_9070937, EPI_ISL_9070940, EPI_ISL_9070943, EPI_ISL_9070945, EPI_ISL_9070948, EPI_ISL_9070951, EPI_ISL_9070957, EPI_ISL_9070967, EPI_ISL_9070970, EPI_ISL_9070989, EPI_ISL_9071008, EPI_ISL_9071015, EPI_ISL_9071018, EPI_ISL_9071021, EPI_ISL_9071022, EPI_ISL_9071023, EPI_ISL_9071024, EPI_ISL_9071025			
see above	Department of Bacteria, Parasites and Fungi, Statens Serum Institut, Copenhagen, Denmark	Statens Serum Institut Bioinformatics and Microbial Genomics	Danish Covid-19 Genome Consortium

We gratefully acknowledge the following Authors from the Originating laboratories responsible for obtaining the specimens, as well as the Submitting laboratories where the genome data were generated and shared via GISAID, on which this research is based.

All Submitters of data may be contacted directly via www.gisaid.org

Authors are sorted alphabetically.

Acknowledgement EPI_SET Identifier: EPI_SET_20220527ae

Accession ID	Originating Laboratory	Submitting Laboratory	Authors
EPI_ISL_9070416, EPI_ISL_9070422, EPI_ISL_9070423, EPI_ISL_9070434, EPI_ISL_9070437, EPI_ISL_9070439, EPI_ISL_9070451, EPI_ISL_9070459, EPI_ISL_9070464, EPI_ISL_9070469, EPI_ISL_9070480, EPI_ISL_9070482, EPI_ISL_9070488, EPI_ISL_9070493, EPI_ISL_9070495, EPI_ISL_9070517, EPI_ISL_9070540, EPI_ISL_9070542, EPI_ISL_9070543, EPI_ISL_9070596, EPI_ISL_9070601, EPI_ISL_9070606, EPI_ISL_9070612, EPI_ISL_9070618, EPI_ISL_9070629, EPI_ISL_9070634, EPI_ISL_9070644, EPI_ISL_9070647, EPI_ISL_9070650, EPI_ISL_9070653, EPI_ISL_9070663, EPI_ISL_9070666, EPI_ISL_9070675, EPI_ISL_9070676, EPI_ISL_9070677, EPI_ISL_9070678, EPI_ISL_9070685, EPI_ISL_9070690, EPI_ISL_9070695, EPI_ISL_9070698, EPI_ISL_9070704, EPI_ISL_9070712, EPI_ISL_9070722, EPI_ISL_9070737, EPI_ISL_9070740, EPI_ISL_9070748, EPI_ISL_9070751, EPI_ISL_9070753, EPI_ISL_9070759, EPI_ISL_9070760			
see above	Department of Bacteria, Parasites and Fungi, Statens Serum Institut, Copenhagen, Denmark	Statens Serum Institut Bioinformatics and Microbial Genomics	Danish Covid-19 Genome Consortium

We gratefully acknowledge the following Authors from the Originating laboratories responsible for obtaining the specimens, as well as the Submitting laboratories where the genome data were generated and shared via GISAID, on which this research is based.

All Submitters of data may be contacted directly via www.gisaid.org

Authors are sorted alphabetically.

Accession ID	Originating Laboratory	Submitting Laboratory	Authors
EPI_ISL_9070067, EPI_ISL_9070080, EPI_ISL_9070083, EPI_ISL_9070087, EPI_ISL_9070091, EPI_ISL_9070092, EPI_ISL_9070099, EPI_ISL_9070102, EPI_ISL_9070105, EPI_ISL_9070111, EPI_ISL_9070132, EPI_ISL_9070163, EPI_ISL_9070171, EPI_ISL_9070174, EPI_ISL_9070179, EPI_ISL_9070183, EPI_ISL_9070188, EPI_ISL_9070189, EPI_ISL_9070190, EPI_ISL_9070191, EPI_ISL_9070192, EPI_ISL_9070198, EPI_ISL_9070201, EPI_ISL_9070211, EPI_ISL_9070217, EPI_ISL_9070220, EPI_ISL_9070222, EPI_ISL_9070232, EPI_ISL_9070238, EPI_ISL_9070264, EPI_ISL_9070270, EPI_ISL_9070273, EPI_ISL_9070322, EPI_ISL_9070325, EPI_ISL_9070331, EPI_ISL_9070333, EPI_ISL_9070342, EPI_ISL_9070347, EPI_ISL_9070351, EPI_ISL_9070358, EPI_ISL_9070362, EPI_ISL_9070372, EPI_ISL_9070375, EPI_ISL_9070380, EPI_ISL_9070383, EPI_ISL_9070391, EPI_ISL_9070394, EPI_ISL_9070408, EPI_ISL_9070410, EPI_ISL_9070413			
see above	Department of Bacteria, Parasites and Fungi, Statens Serum Institut, Copenhagen, Denmark	Statens Serum Institut Bioinformatics and Microbial Genomics	Danish Covid-19 Genome Consortium

We gratefully acknowledge the following Authors from the Originating laboratories responsible for obtaining the specimens, as well as the Submitting laboratories where the genome data were generated and shared via GISAID, on which this research is based.

All Submitters of data may be contacted directly via www.gisaid.org

Authors are sorted alphabetically.

Accession ID	Originating Laboratory	Submitting Laboratory	Authors
EPI_ISL_9069764, EPI_ISL_9069765, EPI_ISL_9069767, EPI_ISL_9069769, EPI_ISL_9069776, EPI_ISL_9069781, EPI_ISL_9069784, EPI_ISL_9069791, EPI_ISL_9069793, EPI_ISL_9069797, EPI_ISL_9069802, EPI_ISL_9069805, EPI_ISL_9069816, EPI_ISL_9069822, EPI_ISL_9069825, EPI_ISL_9069828, EPI_ISL_9069833, EPI_ISL_9069838, EPI_ISL_9069847, EPI_ISL_9069858, EPI_ISL_9069861, EPI_ISL_9069863, EPI_ISL_9069868, EPI_ISL_9069873, EPI_ISL_9069882, EPI_ISL_9069897, EPI_ISL_9069903, EPI_ISL_9069907, EPI_ISL_9069910, EPI_ISL_9069911, EPI_ISL_9069913, EPI_ISL_9069921, EPI_ISL_9069929, EPI_ISL_9069931, EPI_ISL_9069934, EPI_ISL_9069940, EPI_ISL_9069943, EPI_ISL_9069950, EPI_ISL_9069955, EPI_ISL_9069968, EPI_ISL_9069971, EPI_ISL_9069974, EPI_ISL_9069977, EPI_ISL_9069979, EPI_ISL_9069982, EPI_ISL_9069984, EPI_ISL_9069991, EPI_ISL_9069993, EPI_ISL_9070001, EPI_ISL_9070006			
see above	Department of Bacteria, Parasites and Fungi, Statens Serum Institut, Copenhagen, Denmark	Statens Serum Institut Bioinformatics and Microbial Genomics	Danish Covid-19 Genome Consortium

We gratefully acknowledge the following Authors from the Originating laboratories responsible for obtaining the specimens, as well as the Submitting laboratories where the genome data were generated and shared via GISAID, on which this research is based.

All Submitters of data may be contacted directly via www.gisaid.org

Authors are sorted alphabetically.

Acknowledgement EPI_SET Identifier: EPI_SET_20220527pf

Accession ID	Originating Laboratory	Submitting Laboratory	Authors
EPI_ISL_9069357, EPI_ISL_9069364, EPI_ISL_9069367, EPI_ISL_9069370, EPI_ISL_9069379, EPI_ISL_9069393, EPI_ISL_9069396, EPI_ISL_9069397, EPI_ISL_9069400, EPI_ISL_9069403, EPI_ISL_9069406, EPI_ISL_9069409, EPI_ISL_9069412, EPI_ISL_9069415, EPI_ISL_9069427, EPI_ISL_9069433, EPI_ISL_9069436, EPI_ISL_9069439, EPI_ISL_9069442, EPI_ISL_9069444, EPI_ISL_9069450, EPI_ISL_9069465, EPI_ISL_9069487, EPI_ISL_9069492, EPI_ISL_9069495, EPI_ISL_9069500, EPI_ISL_9069503, EPI_ISL_9069509, EPI_ISL_9069512, EPI_ISL_9069523, EPI_ISL_9069526, EPI_ISL_9069534, EPI_ISL_9069539, EPI_ISL_9069550, EPI_ISL_9069576, EPI_ISL_9069587, EPI_ISL_9069592, EPI_ISL_9069597, EPI_ISL_9069598, EPI_ISL_9069602, EPI_ISL_9069607, EPI_ISL_9069614, EPI_ISL_9069616, EPI_ISL_9069617, EPI_ISL_9069624, EPI_ISL_9069627, EPI_ISL_9069633, EPI_ISL_9069666, EPI_ISL_9069672, EPI_ISL_9069741			
see above	Department of Bacteria, Parasites and Fungi, Statens Serum Institut, Copenhagen, Denmark	Statens Serum Institut Bioinformatics and Microbial Genomics	Danish Covid-19 Genome Consortium

We gratefully acknowledge the following Authors from the Originating laboratories responsible for obtaining the specimens, as well as the Submitting laboratories where the genome data were generated and shared via GISAID, on which this research is based.

All Submitters of data may be contacted directly via www.gisaid.org

Authors are sorted alphabetically.

Accession ID	Originating Laboratory	Submitting Laboratory	Authors
EPI_ISL_9068982, EPI_ISL_9068985, EPI_ISL_9069000, EPI_ISL_9069003, EPI_ISL_9069005, EPI_ISL_9069014, EPI_ISL_9069017, EPI_ISL_9069020, EPI_ISL_9069023, EPI_ISL_9069025, EPI_ISL_9069036, EPI_ISL_9069044, EPI_ISL_9069047, EPI_ISL_9069050, EPI_ISL_9069059, EPI_ISL_9069062, EPI_ISL_9069081, EPI_ISL_9069087, EPI_ISL_9069088, EPI_ISL_9069102, EPI_ISL_9069104, EPI_ISL_9069110, EPI_ISL_9069128, EPI_ISL_9069149, EPI_ISL_9069171, EPI_ISL_9069180, EPI_ISL_9069198, EPI_ISL_9069203, EPI_ISL_9069215, EPI_ISL_9069220, EPI_ISL_9069223, EPI_ISL_9069241, EPI_ISL_9069243, EPI_ISL_9069259, EPI_ISL_9069264, EPI_ISL_9069275, EPI_ISL_9069278, EPI_ISL_9069280, EPI_ISL_9069293, EPI_ISL_9069298, EPI_ISL_9069309, EPI_ISL_9069312, EPI_ISL_9069316, EPI_ISL_9069319, EPI_ISL_9069322, EPI_ISL_9069334, EPI_ISL_9069346, EPI_ISL_9069349, EPI_ISL_9069352, EPI_ISL_9069355			
see above	Department of Bacteria, Parasites and Fungi, Statens Serum Institut, Copenhagen, Denmark	Statens Serum Institut Bioinformatics and Microbial Genomics	Danish Covid-19 Genome Consortium

We gratefully acknowledge the following Authors from the Originating laboratories responsible for obtaining the specimens, as well as the Submitting laboratories where the genome data were generated and shared via GISAID, on which this research is based.

All Submitters of data may be contacted directly via www.gisaid.org

Authors are sorted alphabetically.

Accession ID	Originating Laboratory	Submitting Laboratory	Authors
EPI_ISL_9068641, EPI_ISL_9068656, EPI_ISL_9068658, EPI_ISL_9068660, EPI_ISL_9068661, EPI_ISL_9068662, EPI_ISL_9068663, EPI_ISL_9068675, EPI_ISL_9068691, EPI_ISL_9068724, EPI_ISL_9068727, EPI_ISL_9068747, EPI_ISL_9068749, EPI_ISL_9068752, EPI_ISL_9068755, EPI_ISL_9068764, EPI_ISL_9068766, EPI_ISL_9068773, EPI_ISL_9068786, EPI_ISL_9068789, EPI_ISL_9068798, EPI_ISL_9068801, EPI_ISL_9068804, EPI_ISL_9068807, EPI_ISL_9068814, EPI_ISL_9068819, EPI_ISL_9068824, EPI_ISL_9068833, EPI_ISL_9068839, EPI_ISL_9068842, EPI_ISL_9068856, EPI_ISL_9068859, EPI_ISL_9068869, EPI_ISL_9068872, EPI_ISL_9068875, EPI_ISL_9068887, EPI_ISL_9068890, EPI_ISL_9068900, EPI_ISL_9068918, EPI_ISL_9068927, EPI_ISL_9068935, EPI_ISL_9068944, EPI_ISL_9068947, EPI_ISL_9068950, EPI_ISL_9068959, EPI_ISL_9068962, EPI_ISL_9068968, EPI_ISL_9068974, EPI_ISL_9068979			
see above	Department of Bacteria, Parasites and Fungi, Statens Serum Institut, Copenhagen, Denmark	Statens Serum Institut Bioinformatics and Microbial Genomics	Danish Covid-19 Genome Consortium
EPI_ISL_9068734	Lighthouse Lab in Milton Keynes	Wellcome Sanger Institute for the COVID-19 Genomics UK (COG-UK) Consortium	Cordelia Langford; David K. Jackson; Dominic Kwiatkowski; Ewan Harrison; Ian Johnston; Jeffrey Barrett; John Sillitoe on behalf of the Wellcome Sanger Institute COVID-19 Surveillance Team; Roberto Amato; Sonia Goncalves; The Lighthouse Lab in Milton Keynes and Alex Alderton

We gratefully acknowledge the following Authors from the Originating laboratories responsible for obtaining the specimens, as well as the Submitting laboratories where the genome data were generated and shared via GISAID, on which this research is based.

All Submitters of data may be contacted directly via www.gisaid.org

Authors are sorted alphabetically.

Acknowledgement EPI_SET Identifier: EPI_SET_20220527zt

Accession ID	Originating Laboratory	Submitting Laboratory	Authors
EPI_ISL_9149786	National Health Laboratory Services, Virology	National Health Laboratory Services, Virology	Avani Bharuthram; Florette K. Treurnicht; Kathleen Subramoney; Nkhensani Mtileni

We gratefully acknowledge the following Authors from the Originating laboratories responsible for obtaining the specimens, as well as the Submitting laboratories where the genome data were generated and shared via GISAID, on which this research is based.

All Submitters of data may be contacted directly via www.gisaid.org

Authors are sorted alphabetically.

Accession ID	Originating Laboratory	Submitting Laboratory	Authors
EPI_ISL_9102258	Ramathibodi Hospital	Center for Medical Genomics Ramathibodi Hospital	Anek mungaomklang; Insee Sensorn; Inthuorn Ratchatasetthakul; Kanyarat Janthima; Parsakorn Tapaopong; Poramate Jiaranai; Vichapon Tiacharoen; Wasun Chantratita

We gratefully acknowledge the following Authors from the Originating laboratories responsible for obtaining the specimens, as well as the Submitting laboratories where the genome data were generated and shared via GISAID, on which this research is based.

All Submitters of data may be contacted directly via www.gisaid.org

Authors are sorted alphabetically.

Accession ID	Originating Laboratory	Submitting Laboratory	Authors
EPI_ISL_9139503	Aegis Sciences Corporation	Centers for Disease Control and Prevention Division of Viral Diseases, Pathogen Discovery	Alec Vest; Benjamin Rambo-Martin; Christopher Gulvick; Clinton Paden; Cyndi Clark; Dakota Howard; Dhwani Batra; Dillon Nall; Duncan MacCannell; Erisa Sula; Ethan Sanders; Holly Houdeshell; Jason Caravas; Kristine Lacek; Matthew Hardison; Matthew Schmerer; Ola Kvalvaag; Patrick Campbell; Peter Cook; Rob Case; Scott Sammons; Shatavia Morrison; Shaun Westlund; Tymeckia Kendall; Victoria Caban Figueroa; Vikramsinha Ghorpade; Yvette Unoarumhi
EPI_ISL_9129287	Centers for Disease Control and Prevention, Dengue Branch	Centers for Disease Control and Prevention, Dengue Branch	Betzabel Flores; Gabriela Paz-Bailey; Gilberto A. Santiago; Glenda Gonzalez; Jorge L. Munoz-Jordan; Keyla Charriez
EPI_ISL_9112401, EPI_ISL_9112402, EPI_ISL_9112403, EPI_ISL_9112404, EPI_ISL_9112406, EPI_ISL_9112408, EPI_ISL_9112409, EPI_ISL_9112410, EPI_ISL_9112413, EPI_ISL_9112414, EPI_ISL_9112417, EPI_ISL_9112418, EPI_ISL_9112419, EPI_ISL_9112420, EPI_ISL_9112423, EPI_ISL_9112424, EPI_ISL_9112427, EPI_ISL_9112428, EPI_ISL_9112429, EPI_ISL_9112430, EPI_ISL_9112431, EPI_ISL_9112433, EPI_ISL_9112434, EPI_ISL_9112435, EPI_ISL_9112437, EPI_ISL_9112438, EPI_ISL_9112439, EPI_ISL_9112441, EPI_ISL_9112443, EPI_ISL_9112445, EPI_ISL_9112447, EPI_ISL_9112449, EPI_ISL_9112450, EPI_ISL_9112451, EPI_ISL_9112452, EPI_ISL_9112453, EPI_ISL_9112454, EPI_ISL_9112455, EPI_ISL_9112456, EPI_ISL_9112457, EPI_ISL_9112460, EPI_ISL_9112461, EPI_ISL_9112462, EPI_ISL_9112463, EPI_ISL_9112469, EPI_ISL_9112471, EPI_ISL_9112473, EPI_ISL_9112474, EPI_ISL_9112475, EPI_ISL_9112476, EPI_ISL_9112477, EPI_ISL_9112478, EPI_ISL_9112479, EPI_ISL_9112480, EPI_ISL_9112481, EPI_ISL_9112482, EPI_ISL_9112484, EPI_ISL_9112485, EPI_ISL_9112486, EPI_ISL_9112487, EPI_ISL_9112488			
see above	Idaho Bureau of Laboratories	Idaho Bureau of Laboratories	Aimee Ceniseros; Christian Loera; Christopher Ball; Matthew Charles Burns; R. Beukelman; Robert L. Voermans
EPI_ISL_9100723, EPI_ISL_9100728, EPI_ISL_9100744, EPI_ISL_9100745, EPI_ISL_9100748, EPI_ISL_9100751, EPI_ISL_9100752, EPI_ISL_9100753, EPI_ISL_9100756, EPI_ISL_9100757, EPI_ISL_9100759, EPI_ISL_9100760, EPI_ISL_9100761, EPI_ISL_9100763, EPI_ISL_9100769, EPI_ISL_9100774, EPI_ISL_9100776, EPI_ISL_9100778, EPI_ISL_9100783, EPI_ISL_9100784, EPI_ISL_9100785, EPI_ISL_9100786, EPI_ISL_9100787			
see above	Premier Laboratory Servic	BioInfoExperts	Cheryl Metzler; David Nolan; Rebecca Rose; Susanna Lamers; Valerie Murray
EPI_ISL_9132681, EPI_ISL_9132682, EPI_ISL_9133195	Quadrant Laboratories, LLC	SUNY Upstate Medical University	Brian Pavlovitz; Frank Middleton
EPI_ISL_9114519	University of Michigan Clinical Microbiology Laboratory	Lauring Lab, University of Michigan, Department of Microbiology and Immunology	Gilbert
EPI_ISL_9135223	University of Wisconsin-Madison AIDS Vaccine Research Laboratories	University of Wisconsin-Madison AIDS Vaccine Research Laboratories	Gage Moreno; Katarina Braun; et al. AIDS Vaccine Research Laboratories
EPI_ISL_9103201, EPI_ISL_9105109, EPI_ISL_9105124, EPI_ISL_9150547, EPI_ISL_9150551	Utah Public Health Laboratory	Utah Public Health Laboratory	Erin L. Young; John Arnn; Kelly F. Oakeson; Olinto Linares-Perdomo; Pooja Gupta; Tom Iverson

We gratefully acknowledge the following Authors from the Originating laboratories responsible for obtaining the specimens, as well as the Submitting laboratories where the genome data were generated and shared via GISAID, on which this research is based.

All Submitters of data may be contacted directly via www.gisaid.org

Authors are sorted alphabetically.

Accession ID	Originating Laboratory	Submitting Laboratory	Authors
EPI_ISL_9119542, EPI_ISL_9119561, EPI_ISL_9119575, EPI_ISL_9119610, EPI_ISL_9119657, EPI_ISL_9119661, EPI_ISL_9119696, EPI_ISL_9119697, EPI_ISL_9119707, EPI_ISL_9119708, EPI_ISL_9119722, EPI_ISL_9119730, EPI_ISL_9119735, EPI_ISL_9119738, EPI_ISL_9119741, EPI_ISL_9119743, EPI_ISL_9119744, EPI_ISL_9119750, EPI_ISL_9119755, EPI_ISL_9119769, EPI_ISL_9119770, EPI_ISL_9119783, EPI_ISL_9119790, EPI_ISL_9119804, EPI_ISL_9119811, EPI_ISL_9119814, EPI_ISL_9119833, EPI_ISL_9119835, EPI_ISL_9119839, EPI_ISL_9119842, EPI_ISL_9119846, EPI_ISL_9119852, EPI_ISL_9119863, EPI_ISL_9119867, EPI_ISL_9119870, EPI_ISL_9119879, EPI_ISL_9119884, EPI_ISL_9119890, EPI_ISL_9119899, EPI_ISL_9119904, EPI_ISL_9119911, EPI_ISL_9119912, EPI_ISL_9119922, EPI_ISL_9119947, EPI_ISL_9119950, EPI_ISL_9119960, EPI_ISL_9119985, EPI_ISL_9119994, EPI_ISL_9120001, EPI_ISL_9120029, EPI_ISL_9120037, EPI_ISL_9120040, EPI_ISL_9120045, EPI_ISL_9120055, EPI_ISL_9120063, EPI_ISL_9120064, EPI_ISL_9120078, EPI_ISL_9120079, EPI_ISL_9120083, EPI_ISL_9120087, EPI_ISL_9120093, EPI_ISL_9120100, EPI_ISL_9120114, EPI_ISL_9120123, EPI_ISL_9120142, EPI_ISL_9120143, EPI_ISL_9120157, EPI_ISL_9120159, EPI_ISL_9120167, EPI_ISL_9120194, EPI_ISL_9120211, EPI_ISL_9120213, EPI_ISL_9120220, EPI_ISL_9120229, EPI_ISL_9120243, EPI_ISL_9120244, EPI_ISL_9120247, EPI_ISL_9120261, EPI_ISL_9120264, EPI_ISL_9120266, EPI_ISL_9120301, EPI_ISL_9120470, EPI_ISL_9120478, EPI_ISL_9120482, EPI_ISL_9120485, EPI_ISL_9120488, EPI_ISL_9120505, EPI_ISL_9120507, EPI_ISL_9120525, EPI_ISL_9120527, EPI_ISL_9120535, EPI_ISL_9120549, EPI_ISL_9120558, EPI_ISL_9120561, EPI_ISL_9120565, EPI_ISL_9120579, EPI_ISL_9120582, EPI_ISL_9120599, EPI_ISL_9120606, EPI_ISL_9120608, EPI_ISL_9120617, EPI_ISL_9120627, EPI_ISL_9120629, EPI_ISL_9120636, EPI_ISL_9120643, EPI_ISL_9120646, EPI_ISL_9120649, EPI_ISL_9120650, EPI_ISL_9121019, EPI_ISL_9121022, EPI_ISL_9121049, EPI_ISL_9121051, EPI_ISL_9121065, EPI_ISL_9121081, EPI_ISL_9121086, EPI_ISL_9121096, EPI_ISL_9121098, EPI_ISL_9121108, EPI_ISL_9121121, EPI_ISL_9121122, EPI_ISL_9121135, EPI_ISL_9121175, EPI_ISL_9121182, EPI_ISL_9121184, EPI_ISL_9121198, EPI_ISL_9121210, EPI_ISL_9121211, EPI_ISL_9121232, EPI_ISL_9121238, EPI_ISL_9121239, EPI_ISL_9121250, EPI_ISL_9121264, EPI_ISL_9121273, EPI_ISL_9121278, EPI_ISL_9121298, EPI_ISL_9121309, EPI_ISL_9121338, EPI_ISL_9121339, EPI_ISL_9121343, EPI_ISL_9121350, EPI_ISL_9121352, EPI_ISL_9121354, EPI_ISL_9121357, EPI_ISL_9121358, EPI_ISL_9121364, EPI_ISL_9121368, EPI_ISL_9121375, EPI_ISL_9121379, EPI_ISL_9121380, EPI_ISL_9121381, EPI_ISL_9121389, EPI_ISL_9121396, EPI_ISL_9121405, EPI_ISL_9121409, EPI_ISL_9121418, EPI_ISL_9121421, EPI_ISL_9121426, EPI_ISL_9121431, EPI_ISL_9121434, EPI_ISL_9121442, EPI_ISL_9121461, EPI_ISL_9121487, EPI_ISL_9121500, EPI_ISL_9121508, EPI_ISL_9121516, EPI_ISL_9121529, EPI_ISL_9121533, EPI_ISL_9121542, EPI_ISL_9121562, EPI_ISL_9121564, EPI_ISL_9121565, EPI_ISL_9121569, EPI_ISL_9121579, EPI_ISL_9121583, EPI_ISL_9121590, EPI_ISL_9121620, EPI_ISL_9121625, EPI_ISL_9121629, EPI_ISL_9121641, EPI_ISL_9121652, EPI_ISL_9121653, EPI_ISL_9121691, EPI_ISL_9121725, EPI_ISL_9121751, EPI_ISL_9121752, EPI_ISL_9121754, EPI_ISL_9121766, EPI_ISL_9121778, EPI_ISL_9121780, EPI_ISL_9121784, EPI_ISL_9121785, EPI_ISL_9121802, EPI_ISL_9121803, EPI_ISL_9121856, EPI_ISL_9121858, EPI_ISL_9121875, EPI_ISL_9121880, EPI_ISL_9121900, EPI_ISL_9121907, EPI_ISL_9121909, EPI_ISL_9121913, EPI_ISL_9121947, EPI_ISL_9122019, EPI_ISL_9122024, EPI_ISL_9122032, EPI_ISL_9122034, EPI_ISL_9122044, EPI_ISL_9122046, EPI_ISL_9122059, EPI_ISL_9122069, EPI_ISL_9122077, EPI_ISL_9122078, EPI_ISL_9122082, EPI_ISL_9122086, EPI_ISL_9122087, EPI_ISL_9122089, EPI_ISL_9122111, EPI_ISL_9122123, EPI_ISL_9122130, EPI_ISL_9122142, EPI_ISL_9122170, EPI_ISL_9122176, EPI_ISL_9122938, EPI_ISL_9122949, EPI_ISL_9122950, EPI_ISL_9122968, EPI_ISL_9122990, EPI_ISL_9122995, EPI_ISL_9122996, EPI_ISL_9123113, EPI_ISL_9123118, EPI_ISL_9123124, EPI_ISL_9123126, EPI_ISL_9123163, EPI_ISL_9123165, EPI_ISL_9123166, EPI_ISL_9123168, EPI_ISL_9123173, EPI_ISL_9123177, EPI_ISL_9123182, EPI_ISL_9123190, EPI_ISL_9123194, EPI_ISL_9123195, EPI_ISL_9123197, EPI_ISL_9123205, EPI_ISL_9123208, EPI_ISL_9123221, EPI_ISL_9123254, EPI_ISL_9123275, EPI_ISL_9123280, EPI_ISL_9123309, EPI_ISL_9123317, EPI_ISL_9123324, EPI_ISL_9123327, EPI_ISL_9123333, EPI_ISL_9123334, EPI_ISL_9123341, EPI_ISL_9123349, EPI_ISL_9123359, EPI_ISL_9123374, EPI_ISL_9123376, EPI_ISL_9123377, EPI_ISL_9123384, EPI_ISL_9123386, EPI_ISL_9123389, EPI_ISL_9123404, EPI_ISL_9123412, EPI_ISL_9123413, EPI_ISL_9123415, EPI_ISL_9123419, EPI_ISL_9123425, EPI_ISL_9123440, EPI_ISL_9123450, EPI_ISL_9123452, EPI_ISL_9123457, EPI_ISL_9123458, EPI_ISL_9123461, EPI_ISL_9123463, EPI_ISL_9123465, EPI_ISL_9123469, EPI_ISL_9123494, EPI_ISL_9123499, EPI_ISL_9123513, EPI_ISL_9123536, EPI_ISL_9123565, EPI_ISL_9123760, EPI_ISL_9123775, EPI_ISL_9123782, EPI_ISL_9123788, EPI_ISL_9123791, EPI_ISL_9124001, EPI_ISL_9124014, EPI_ISL_9124020, EPI_ISL_9124024, EPI_ISL_9124037, EPI_ISL_9124043, EPI_ISL_9124055, EPI_ISL_9124086, EPI_ISL_9124109, EPI_ISL_9124111, EPI_ISL_9124126, EPI_ISL_9124129, EPI_ISL_9124153, EPI_ISL_9124174, EPI_ISL_9124176, EPI_ISL_9124181, EPI_ISL_9124183, EPI_ISL_9124192, EPI_ISL_9124195, EPI_ISL_9124209, EPI_ISL_9124225, EPI_ISL_9124281, EPI_ISL_9124310, EPI_ISL_9124356, EPI_ISL_9124376, EPI_ISL_9124399, EPI_ISL_9124416, EPI_ISL_9124444, EPI_ISL_9124455, EPI_ISL_9124457, EPI_ISL_9124475, EPI_ISL_9124479, EPI_ISL_9124480, EPI_ISL_9124497, EPI_ISL_9124503, EPI_ISL_9124505, EPI_ISL_9124514, EPI_ISL_9124544, EPI_ISL_9124560, EPI_ISL_9124564, EPI_ISL_9124627, EPI_ISL_9124630, EPI_ISL_9124633, EPI_ISL_9124666, EPI_ISL_9124743, EPI_ISL_9124765, EPI_ISL_9124808, EPI_ISL_9124814, EPI_ISL_9124823, EPI_ISL_9124912, EPI_ISL_9124945, EPI_ISL_9124957, EPI_ISL_9124959, EPI_ISL_9124979, EPI_ISL_9124994, EPI_ISL_9124997, EPI_ISL_9125293, EPI_ISL_9125306, EPI_ISL_9125318, EPI_ISL_9125326, EPI_ISL_9125328, EPI_ISL_9125341, EPI_ISL_9125346, EPI_ISL_9125364, EPI_ISL_9125375, EPI_ISL_9125401, EPI_ISL_9125419, EPI_ISL_9125430, EPI_ISL_9125441, EPI_ISL_9125499, EPI_ISL_9125519, EPI_ISL_9125555, EPI_ISL_9125929, EPI_ISL_9125947, EPI_ISL_9126011, EPI_ISL_9126036, EPI_ISL_9126039, EPI_ISL_9126045, EPI_ISL_9126051, EPI_ISL_9126066, EPI_ISL_9126106, EPI_ISL_9126114, EPI_ISL_9126129, EPI_ISL_9126184, EPI_ISL_9126213, EPI_ISL_9126249, EPI_ISL_9126263, EPI_ISL_9126269, EPI_ISL_9126281, EPI_ISL_9126291, EPI_ISL_9126303, EPI_ISL_9126307, EPI_ISL_9126316, EPI_ISL_9126501, EPI_ISL_9126831, EPI_ISL_9126848, EPI_ISL_9126865, EPI_ISL_9126916, EPI_ISL_9126961, EPI_ISL_9126984, EPI_ISL_9126999, EPI_ISL_9127019, EPI_ISL_9127262, EPI_ISL_9127291, EPI_ISL_9127465, EPI_ISL_9127475, EPI_ISL_9127483, EPI_ISL_9127514, EPI_ISL_9127516, EPI_ISL_9127520, EPI_ISL_9127526, EPI_ISL_9127562, EPI_ISL_9127598, EPI_ISL_9127627, EPI_ISL_9127644, EPI_ISL_9127660, EPI_ISL_9127681, EPI_ISL_9127697, EPI_ISL_9127709, EPI_ISL_9127842, EPI_ISL_9127851, EPI_ISL_9127855, EPI_ISL_9127863, EPI_ISL_9127866, EPI_ISL_9127887, EPI_ISL_9127893, EPI_ISL_9127947, EPI_ISL_9127971, EPI_ISL_9127982, EPI_ISL_9127997, EPI_ISL_9128045, EPI_ISL_9128136, EPI_ISL_9128157, EPI_ISL_9128210, EPI_ISL_9128216, EPI_ISL_9128235, EPI_ISL_9128342, EPI_ISL_9128400, EPI_ISL_9128437, EPI_ISL_9128444, EPI_ISL_9128525, EPI_ISL_9128531, EPI_ISL_9128572, EPI_ISL_9128677, EPI_ISL_9128681, EPI_ISL_9128688, EPI_ISL_9128695, EPI_ISL_9128701, EPI_ISL_9128718, EPI_ISL_9128721, EPI_ISL_9128725, EPI_ISL_9128774, EPI_ISL_9128788, EPI_ISL_9128811, EPI_ISL_9128820, EPI_ISL_9128825, EPI_ISL_9128828, EPI_ISL_9128837, EPI_ISL_9128840, EPI_ISL_9128849, EPI_ISL_9128852, EPI_ISL_9128908, EPI_ISL_9128914, EPI_ISL_9128950, EPI_ISL_9128960, EPI_ISL_9128993, EPI_ISL_9129006, EPI_ISL_9129009, EPI_ISL_9129011, EPI_ISL_9129025, EPI_ISL_9129042, EPI_ISL_9129078, EPI_ISL_9129085, EPI_ISL_9129131, EPI_ISL_9131404, EPI_ISL_9131441, EPI_ISL_9131445, EPI_ISL_9131449, EPI_ISL_9131612, EPI_ISL_9131658, EPI_ISL_9131680, EPI_ISL_9131696, EPI_ISL_9131736, EPI_ISL_9131746, EPI_ISL_9131770, EPI_ISL_9131779, EPI_ISL_9131784, EPI_ISL_9134224, EPI_ISL_9134246, EPI_ISL_9134251, EPI_ISL_9134271, EPI_ISL_9134288, EPI_ISL_9134295, EPI_ISL_9134309, EPI_ISL_9134313, EPI_ISL_9134642			
see above	Department of Bacteria, Parasites and Fungi, Statens Serum Institut, Copenhagen, Denmark	Statens Serum Institut Bioinformatics and Microbial Genomics	Danish Covid-19 Genome Consortium

We gratefully acknowledge the following Authors from the Originating laboratories responsible for obtaining the specimens, as well as the Submitting laboratories where the genome data were generated and shared via GISAID, on which this research is based.

All Submitters of data may be contacted directly via www.gisaid.org

Authors are sorted alphabetically.

Acknowledgement EPI_SET Identifier: EPI_SET_20220527no

Accession ID	Originating Laboratory	Submitting Laboratory	Authors
EPI_ISL_9119563, EPI_ISL_9119618, EPI_ISL_9119689, EPI_ISL_9119693, EPI_ISL_9119701, EPI_ISL_9119712, EPI_ISL_9119720, EPI_ISL_9119732, EPI_ISL_9119756, EPI_ISL_9119764, EPI_ISL_9119779, EPI_ISL_9119817, EPI_ISL_9119829, EPI_ISL_9119847, EPI_ISL_9119855, EPI_ISL_9119883, EPI_ISL_9119894, EPI_ISL_9119898, EPI_ISL_9119905, EPI_ISL_9119929, EPI_ISL_9119968, EPI_ISL_9120015, EPI_ISL_9120048, EPI_ISL_9120060, EPI_ISL_9120073, EPI_ISL_9120085, EPI_ISL_9120090, EPI_ISL_9120096, EPI_ISL_9120128, EPI_ISL_9120172, EPI_ISL_9120174, EPI_ISL_9120215, EPI_ISL_9120224, EPI_ISL_9120233, EPI_ISL_9120245, EPI_ISL_9120258, EPI_ISL_9120364, EPI_ISL_9120472, EPI_ISL_9120476, EPI_ISL_9120479, EPI_ISL_9120494, EPI_ISL_9120511, EPI_ISL_9120516, EPI_ISL_9120530, EPI_ISL_9120557, EPI_ISL_9120574, EPI_ISL_9120581, EPI_ISL_9120609, EPI_ISL_9120614, EPI_ISL_9120620, EPI_ISL_9120624, EPI_ISL_9120626, EPI_ISL_9120632, EPI_ISL_9120639, EPI_ISL_9121029, EPI_ISL_9121030, EPI_ISL_9121040, EPI_ISL_9121042, EPI_ISL_9121043, EPI_ISL_9121068, EPI_ISL_9121100, EPI_ISL_9121102, EPI_ISL_9121134, EPI_ISL_9121173, EPI_ISL_9121192, EPI_ISL_9121193, EPI_ISL_9121204, EPI_ISL_9121214, EPI_ISL_9121221, EPI_ISL_9121222, EPI_ISL_9121226, EPI_ISL_9121261, EPI_ISL_9121269, EPI_ISL_9121290, EPI_ISL_9121299, EPI_ISL_9121300, EPI_ISL_9121303, EPI_ISL_9121304, EPI_ISL_9121308, EPI_ISL_9121331, EPI_ISL_9121382, EPI_ISL_9121399, EPI_ISL_9121406, EPI_ISL_9121424, EPI_ISL_9121471, EPI_ISL_9121475, EPI_ISL_9121496, EPI_ISL_9121517, EPI_ISL_9121527, EPI_ISL_9121546, EPI_ISL_9121549, EPI_ISL_9121551, EPI_ISL_9121557, EPI_ISL_9121576, EPI_ISL_9121606, EPI_ISL_9121611, EPI_ISL_9121631, EPI_ISL_9121635, EPI_ISL_9121637, EPI_ISL_9121643, EPI_ISL_9121704, EPI_ISL_9121736, EPI_ISL_9121763, EPI_ISL_9121787, EPI_ISL_9121797, EPI_ISL_9121844, EPI_ISL_9121848, EPI_ISL_9121853, EPI_ISL_9121899, EPI_ISL_9121902, EPI_ISL_9122023, EPI_ISL_9122047, EPI_ISL_9122054, EPI_ISL_9122057, EPI_ISL_9122064, EPI_ISL_9122068, EPI_ISL_9122075, EPI_ISL_9122145, EPI_ISL_9122146, EPI_ISL_9122147, EPI_ISL_9122174, EPI_ISL_9122918, EPI_ISL_9122925, EPI_ISL_9122939, EPI_ISL_9123119, EPI_ISL_9123127, EPI_ISL_9123141, EPI_ISL_9123147, EPI_ISL_9123154, EPI_ISL_9123199, EPI_ISL_9123224, EPI_ISL_9123250, EPI_ISL_9123274, EPI_ISL_9123288, EPI_ISL_9123297, EPI_ISL_9123311, EPI_ISL_9123329, EPI_ISL_9123361, EPI_ISL_9123435, EPI_ISL_9123446, EPI_ISL_9123467, EPI_ISL_9123484, EPI_ISL_9123485, EPI_ISL_9123486, EPI_ISL_9123516, EPI_ISL_9123538, EPI_ISL_9123540, EPI_ISL_9123544, EPI_ISL_9123548, EPI_ISL_9123550, EPI_ISL_9123551, EPI_ISL_9123764, EPI_ISL_9123776, EPI_ISL_9123790, EPI_ISL_9124010, EPI_ISL_9124025, EPI_ISL_9124039, EPI_ISL_9124040, EPI_ISL_9124143, EPI_ISL_9124162, EPI_ISL_9124167, EPI_ISL_9124182, EPI_ISL_9124201, EPI_ISL_9124223, EPI_ISL_9124228, EPI_ISL_9124243, EPI_ISL_9124258, EPI_ISL_9124302, EPI_ISL_9124373, EPI_ISL_9124494, EPI_ISL_9124517, EPI_ISL_9124587, EPI_ISL_9124610, EPI_ISL_9124802, EPI_ISL_9124835, EPI_ISL_9124896, EPI_ISL_9124942, EPI_ISL_9124956, EPI_ISL_9125016, EPI_ISL_9125060, EPI_ISL_9125289, EPI_ISL_9125304, EPI_ISL_9125361, EPI_ISL_9125372, EPI_ISL_9125394, EPI_ISL_9125445, EPI_ISL_9125531, EPI_ISL_9125919, EPI_ISL_9125988, EPI_ISL_9126000, EPI_ISL_9126083, EPI_ISL_9126229, EPI_ISL_9126845, EPI_ISL_9126858, EPI_ISL_9126873, EPI_ISL_9126925, EPI_ISL_9126966, EPI_ISL_9126978, EPI_ISL_9126994, EPI_ISL_9127050, EPI_ISL_9127054, EPI_ISL_9127077, EPI_ISL_9127273, EPI_ISL_9127336, EPI_ISL_9127340, EPI_ISL_9127373, EPI_ISL_9127409, EPI_ISL_9127455, EPI_ISL_9127522, EPI_ISL_9127559, EPI_ISL_9127610, EPI_ISL_9127657, EPI_ISL_9127668, EPI_ISL_9127671, EPI_ISL_9127736, EPI_ISL_9127739, EPI_ISL_9127753, EPI_ISL_9127848, EPI_ISL_9127873, EPI_ISL_9127910, EPI_ISL_9127955, EPI_ISL_9128010, EPI_ISL_9128060, EPI_ISL_9128177, EPI_ISL_9128194, EPI_ISL_9128365, EPI_ISL_9128377, EPI_ISL_9128456, EPI_ISL_9128474, EPI_ISL_9128480, EPI_ISL_9128483, EPI_ISL_9128501, EPI_ISL_9128665, EPI_ISL_9128685, EPI_ISL_9128739, EPI_ISL_9128763, EPI_ISL_9128872, EPI_ISL_9128917, EPI_ISL_9129056, EPI_ISL_9129066, EPI_ISL_9131419, EPI_ISL_9131434, EPI_ISL_9131443, EPI_ISL_9131534, EPI_ISL_9131651, EPI_ISL_9131740, EPI_ISL_9131742, EPI_ISL_9134221, EPI_ISL_9134267			
see above	Department of Bacteria, Parasites and Fungi, Statens Serum Institut, Copenhagen, Denmark	Statens Serum Institut Bioinformatics and Microbial Genomics	Danish Covid-19 Genome Consortium

We gratefully acknowledge the following Authors from the Originating laboratories responsible for obtaining the specimens, as well as the Submitting laboratories where the genome data were generated and shared via GISAID, on which this research is based.

All Submitters of data may be contacted directly via www.gisaid.org

Authors are sorted alphabetically.

Accession ID	Originating Laboratory	Submitting Laboratory	Authors
EPI_ISL_9119550, EPI_ISL_9119599, EPI_ISL_9119665, EPI_ISL_9119667, EPI_ISL_9119690, EPI_ISL_9119725, EPI_ISL_9119746, EPI_ISL_9119762, EPI_ISL_9119766, EPI_ISL_9119780, EPI_ISL_9119797, EPI_ISL_9119818, EPI_ISL_9119848, EPI_ISL_9119878, EPI_ISL_9119881, EPI_ISL_9119893, EPI_ISL_9119907, EPI_ISL_9119952, EPI_ISL_9119962, EPI_ISL_9119963, EPI_ISL_9119981, EPI_ISL_9119992, EPI_ISL_9119998, EPI_ISL_9120013, EPI_ISL_9120020, EPI_ISL_9120024, EPI_ISL_9120025, EPI_ISL_9120034, EPI_ISL_9120069, EPI_ISL_9120070, EPI_ISL_9120082, EPI_ISL_9120092, EPI_ISL_9120121, EPI_ISL_9120133, EPI_ISL_9120158, EPI_ISL_9120168, EPI_ISL_9120183, EPI_ISL_9120186, EPI_ISL_9120193, EPI_ISL_9120218, EPI_ISL_9120221, EPI_ISL_9120225, EPI_ISL_9120252, EPI_ISL_9120468, EPI_ISL_9120492, EPI_ISL_9120526, EPI_ISL_9120552, EPI_ISL_9120556, EPI_ISL_9120641, EPI_ISL_9121023, EPI_ISL_9121036, EPI_ISL_9121057, EPI_ISL_9121095, EPI_ISL_9121099, EPI_ISL_9121115, EPI_ISL_9121194, EPI_ISL_9121201, EPI_ISL_9121237, EPI_ISL_9121262, EPI_ISL_9121289, EPI_ISL_9121294, EPI_ISL_9121328, EPI_ISL_9121361, EPI_ISL_9121371, EPI_ISL_9121392, EPI_ISL_9121420, EPI_ISL_9121422, EPI_ISL_9121432, EPI_ISL_9121466, EPI_ISL_9121505, EPI_ISL_9121535, EPI_ISL_9121536, EPI_ISL_9121543, EPI_ISL_9121554, EPI_ISL_9121566, EPI_ISL_9121567, EPI_ISL_9121572, EPI_ISL_9121580, EPI_ISL_9121723, EPI_ISL_9121741, EPI_ISL_9121779, EPI_ISL_9121862, EPI_ISL_9121868, EPI_ISL_9122020, EPI_ISL_9122035, EPI_ISL_9122041, EPI_ISL_9122042, EPI_ISL_9122048, EPI_ISL_9122049, EPI_ISL_9122056, EPI_ISL_9122074, EPI_ISL_9122083, EPI_ISL_9122101, EPI_ISL_9122118, EPI_ISL_9122129, EPI_ISL_9122133, EPI_ISL_9122143, EPI_ISL_9122151, EPI_ISL_9122920, EPI_ISL_9122923, EPI_ISL_9122926, EPI_ISL_9122954, EPI_ISL_9122959, EPI_ISL_9122976, EPI_ISL_9123047, EPI_ISL_9123122, EPI_ISL_9123129, EPI_ISL_9123160, EPI_ISL_9123170, EPI_ISL_9123226, EPI_ISL_9123230, EPI_ISL_9123235, EPI_ISL_9123249, EPI_ISL_9123291, EPI_ISL_9123306, EPI_ISL_9123314, EPI_ISL_9123323, EPI_ISL_9123335, EPI_ISL_9123355, EPI_ISL_9123403, EPI_ISL_9123408, EPI_ISL_9123417, EPI_ISL_9123432, EPI_ISL_9123473, EPI_ISL_9123495, EPI_ISL_9123498, EPI_ISL_9123521, EPI_ISL_9123789, EPI_ISL_9123798, EPI_ISL_9124104, EPI_ISL_9124134, EPI_ISL_9124187, EPI_ISL_9124237, EPI_ISL_9124255, EPI_ISL_9124279, EPI_ISL_9124441, EPI_ISL_9124488, EPI_ISL_9124687, EPI_ISL_9124774, EPI_ISL_9124884, EPI_ISL_9124951, EPI_ISL_9125343, EPI_ISL_9125547, EPI_ISL_9126072, EPI_ISL_9126075, EPI_ISL_9126140, EPI_ISL_9126142, EPI_ISL_9126219, EPI_ISL_9126296, EPI_ISL_9126505, EPI_ISL_9126829, EPI_ISL_9126888, EPI_ISL_9126892, EPI_ISL_9127008, EPI_ISL_9127083, EPI_ISL_9127276, EPI_ISL_9127297, EPI_ISL_9127302, EPI_ISL_9127332, EPI_ISL_9127407, EPI_ISL_9127412, EPI_ISL_9127414, EPI_ISL_9127418, EPI_ISL_9127444, EPI_ISL_9127543, EPI_ISL_9127564, EPI_ISL_9127570, EPI_ISL_9127573, EPI_ISL_9127621, EPI_ISL_9127646, EPI_ISL_9127679, EPI_ISL_9127700, EPI_ISL_9127723, EPI_ISL_9127823, EPI_ISL_9127862, EPI_ISL_9127869, EPI_ISL_9127899, EPI_ISL_9127913, EPI_ISL_9127925, EPI_ISL_9128000, EPI_ISL_9128007, EPI_ISL_9128057, EPI_ISL_9128113, EPI_ISL_9128124, EPI_ISL_9128269, EPI_ISL_9128368, EPI_ISL_9128462, EPI_ISL_9128479, EPI_ISL_9128540, EPI_ISL_9128612, EPI_ISL_9128615, EPI_ISL_9128666, EPI_ISL_9128704, EPI_ISL_9128730, EPI_ISL_9128748, EPI_ISL_9128911, EPI_ISL_9128936, EPI_ISL_9128957, EPI_ISL_9128984, EPI_ISL_9128996, EPI_ISL_9129014, EPI_ISL_9131414, EPI_ISL_9131444, EPI_ISL_9131491, EPI_ISL_9131545, EPI_ISL_9131580, EPI_ISL_9131625, EPI_ISL_9131676, EPI_ISL_9131733			
see above	Department of Bacteria, Parasites and Fungi, Statens Serum Institut, Copenhagen, Denmark	Statens Serum Institut Bioinformatics and Microbial Genomics	Danish Covid-19 Genome Consortium

We gratefully acknowledge the following Authors from the Originating laboratories responsible for obtaining the specimens, as well as the Submitting laboratories where the genome data were generated and shared via GISAID, on which this research is based.

All Submitters of data may be contacted directly via www.gisaid.org

Authors are sorted alphabetically.

Accession ID	Originating Laboratory	Submitting Laboratory	Authors
EPI_ISL_9119565, EPI_ISL_9119596, EPI_ISL_9119636, EPI_ISL_9119641, EPI_ISL_9119652, EPI_ISL_9119683, EPI_ISL_9119685, EPI_ISL_9119700, EPI_ISL_9119713, EPI_ISL_9119742, EPI_ISL_9119749, EPI_ISL_9119751, EPI_ISL_9119753, EPI_ISL_9119754, EPI_ISL_9119785, EPI_ISL_9119799, EPI_ISL_9119800, EPI_ISL_9119805, EPI_ISL_9119807, EPI_ISL_9119816, EPI_ISL_9119819, EPI_ISL_9119820, EPI_ISL_9119825, EPI_ISL_9119832, EPI_ISL_9119850, EPI_ISL_9119856, EPI_ISL_9119862, EPI_ISL_9119872, EPI_ISL_9119877, EPI_ISL_9119906, EPI_ISL_9119910, EPI_ISL_9119933, EPI_ISL_9119936, EPI_ISL_9119939, EPI_ISL_9119942, EPI_ISL_9119953, EPI_ISL_9119957, EPI_ISL_9119961, EPI_ISL_9119966, EPI_ISL_9119972, EPI_ISL_9119976, EPI_ISL_9119977, EPI_ISL_9119978, EPI_ISL_9119986, EPI_ISL_9119999, EPI_ISL_9120003, EPI_ISL_9120023, EPI_ISL_9120027, EPI_ISL_9120028, EPI_ISL_9120031, EPI_ISL_9120049, EPI_ISL_9120052, EPI_ISL_9120053, EPI_ISL_9120071, EPI_ISL_9120072, EPI_ISL_9120075, EPI_ISL_9120077, EPI_ISL_9120110, EPI_ISL_9120119, EPI_ISL_9120122, EPI_ISL_9120125, EPI_ISL_9120131, EPI_ISL_9120171, EPI_ISL_9120175, EPI_ISL_9120177, EPI_ISL_9120185, EPI_ISL_9120200, EPI_ISL_9120204, EPI_ISL_9120207, EPI_ISL_9120209, EPI_ISL_9120214, EPI_ISL_9120216, EPI_ISL_9120219, EPI_ISL_9120226, EPI_ISL_9120228, EPI_ISL_9120231, EPI_ISL_9120246, EPI_ISL_9120254, EPI_ISL_9120262, EPI_ISL_9120480, EPI_ISL_9120493, EPI_ISL_9120497, EPI_ISL_9120503, EPI_ISL_9120512, EPI_ISL_9120514, EPI_ISL_9120518, EPI_ISL_9120519, EPI_ISL_9120529, EPI_ISL_9120537, EPI_ISL_9120540, EPI_ISL_9120544, EPI_ISL_9120545, EPI_ISL_9120547, EPI_ISL_9120570, EPI_ISL_9120578, EPI_ISL_9120591, EPI_ISL_9120592, EPI_ISL_9120594, EPI_ISL_9120596, EPI_ISL_9120597, EPI_ISL_9120598, EPI_ISL_9120602, EPI_ISL_9120610, EPI_ISL_9120628, EPI_ISL_9120637, EPI_ISL_9120902, EPI_ISL_9121028, EPI_ISL_9121039, EPI_ISL_9121052, EPI_ISL_9121056, EPI_ISL_9121058, EPI_ISL_9121080, EPI_ISL_9121082, EPI_ISL_9121084, EPI_ISL_9121104, EPI_ISL_9121117, EPI_ISL_9121118, EPI_ISL_9121119, EPI_ISL_9121133, EPI_ISL_9121141, EPI_ISL_9121178, EPI_ISL_9121179, EPI_ISL_9121195, EPI_ISL_9121202, EPI_ISL_9121203, EPI_ISL_9121209, EPI_ISL_9121213, EPI_ISL_9121218, EPI_ISL_9121219, EPI_ISL_9121227, EPI_ISL_9121251, EPI_ISL_9121255, EPI_ISL_9121258, EPI_ISL_9121267, EPI_ISL_9121271, EPI_ISL_9121280, EPI_ISL_9121282, EPI_ISL_9121296, EPI_ISL_9121307, EPI_ISL_9121322, EPI_ISL_9121327, EPI_ISL_9121332, EPI_ISL_9121342, EPI_ISL_9121360, EPI_ISL_9121366, EPI_ISL_9121373, EPI_ISL_9121388, EPI_ISL_9121412, EPI_ISL_9121417, EPI_ISL_9121425, EPI_ISL_9121430, EPI_ISL_9121447, EPI_ISL_9121457, EPI_ISL_9121464, EPI_ISL_9121467, EPI_ISL_9121468, EPI_ISL_9121476, EPI_ISL_9121486, EPI_ISL_9121491, EPI_ISL_9121504, EPI_ISL_9121507, EPI_ISL_9121513, EPI_ISL_9121523, EPI_ISL_9121525, EPI_ISL_9121552, EPI_ISL_9121558, EPI_ISL_9121581, EPI_ISL_9121591, EPI_ISL_9121592, EPI_ISL_9121602, EPI_ISL_9121604, EPI_ISL_9121610, EPI_ISL_9121612, EPI_ISL_9121616, EPI_ISL_9121627, EPI_ISL_9121630, EPI_ISL_9121634, EPI_ISL_9121639, EPI_ISL_9121647, EPI_ISL_9121648, EPI_ISL_9121651, EPI_ISL_9121695, EPI_ISL_9121710, EPI_ISL_9121724, EPI_ISL_9121742, EPI_ISL_9121746, EPI_ISL_9121764, EPI_ISL_9121765, EPI_ISL_9121773, EPI_ISL_9121782, EPI_ISL_9121794, EPI_ISL_9121795, EPI_ISL_9121845, EPI_ISL_9121857, EPI_ISL_9121860, EPI_ISL_9121864, EPI_ISL_9121865, EPI_ISL_9121878, EPI_ISL_9121885, EPI_ISL_9121886, EPI_ISL_9121892, EPI_ISL_9122014, EPI_ISL_9122017, EPI_ISL_9122036, EPI_ISL_9122038, EPI_ISL_9122067, EPI_ISL_9122070, EPI_ISL_9122071, EPI_ISL_9122079, EPI_ISL_9122091, EPI_ISL_9122095, EPI_ISL_9122100, EPI_ISL_9122120, EPI_ISL_9122124, EPI_ISL_9122136, EPI_ISL_9122137, EPI_ISL_9122152, EPI_ISL_9122163, EPI_ISL_9122166, EPI_ISL_9122921, EPI_ISL_9122931, EPI_ISL_9122948, EPI_ISL_9122953, EPI_ISL_9122966, EPI_ISL_9122967, EPI_ISL_9122989, EPI_ISL_9122991, EPI_ISL_9123001, EPI_ISL_9123117, EPI_ISL_9123120, EPI_ISL_9123130, EPI_ISL_9123131, EPI_ISL_9123132, EPI_ISL_9123133, EPI_ISL_9123151, EPI_ISL_9123153, EPI_ISL_9123162, EPI_ISL_9123180, EPI_ISL_9123186, EPI_ISL_9123214, EPI_ISL_9123217, EPI_ISL_9123219, EPI_ISL_9123222, EPI_ISL_9123231, EPI_ISL_9123239, EPI_ISL_9123240, EPI_ISL_9123259, EPI_ISL_9123261, EPI_ISL_9123262, EPI_ISL_9123269, EPI_ISL_9123281, EPI_ISL_9123298, EPI_ISL_9123300, EPI_ISL_9123304, EPI_ISL_9123307, EPI_ISL_9123316, EPI_ISL_9123322, EPI_ISL_9123326, EPI_ISL_9123328, EPI_ISL_9123337, EPI_ISL_9123345, EPI_ISL_9123346, EPI_ISL_9123350, EPI_ISL_9123357, EPI_ISL_9123358, EPI_ISL_9123371, EPI_ISL_9123394, EPI_ISL_9123397, EPI_ISL_9123400, EPI_ISL_9123401, EPI_ISL_9123405, EPI_ISL_9123434, EPI_ISL_9123460, EPI_ISL_9123471, EPI_ISL_9123472, EPI_ISL_9123475, EPI_ISL_9123477, EPI_ISL_9123481, EPI_ISL_9123487, EPI_ISL_9123489, EPI_ISL_9123496, EPI_ISL_9123500, EPI_ISL_9123504, EPI_ISL_9123505, EPI_ISL_9123507, EPI_ISL_9123518, EPI_ISL_9123525, EPI_ISL_9123528, EPI_ISL_9123530, EPI_ISL_9123556, EPI_ISL_9123558, EPI_ISL_9123561, EPI_ISL_9123562, EPI_ISL_9123754, EPI_ISL_9123768, EPI_ISL_9123771, EPI_ISL_9123793, EPI_ISL_9123997, EPI_ISL_9124007, EPI_ISL_9124017, EPI_ISL_9124019, EPI_ISL_9124023, EPI_ISL_9124035, EPI_ISL_9124047, EPI_ISL_9124052, EPI_ISL_9124058, EPI_ISL_9124114, EPI_ISL_9124140, EPI_ISL_9124145, EPI_ISL_9124156, EPI_ISL_9124161, EPI_ISL_9124190, EPI_ISL_9124215, EPI_ISL_9124217, EPI_ISL_9124220, EPI_ISL_9124240, EPI_ISL_9124284, EPI_ISL_9124296, EPI_ISL_9124308, EPI_ISL_9124323, EPI_ISL_9124344, EPI_ISL_9124370, EPI_ISL_9124379, EPI_ISL_9124382, EPI_ISL_9124405, EPI_ISL_9124418, EPI_ISL_9124421, EPI_ISL_9124424, EPI_ISL_9124432, EPI_ISL_9124435, EPI_ISL_9124482, EPI_ISL_9124546, EPI_ISL_9124561, EPI_ISL_9124562, EPI_ISL_9124581, EPI_ISL_9124624, EPI_ISL_9124647, EPI_ISL_9124663, EPI_ISL_9124668, EPI_ISL_9124783, EPI_ISL_9124829, EPI_ISL_9124832, EPI_ISL_9124849, EPI_ISL_9124859, EPI_ISL_9124907, EPI_ISL_9124923, EPI_ISL_9124960, EPI_ISL_9124986, EPI_ISL_9125000, EPI_ISL_9125338, EPI_ISL_9125357, EPI_ISL_9125366, EPI_ISL_9125422, EPI_ISL_9125438, EPI_ISL_9125443, EPI_ISL_9125444, EPI_ISL_9125446, EPI_ISL_9125452, EPI_ISL_9125466, EPI_ISL_9125475, EPI_ISL_9125478, EPI_ISL_9125489, EPI_ISL_9125496, EPI_ISL_9125515, EPI_ISL_9125522, EPI_ISL_9125529, EPI_ISL_9125905, EPI_ISL_9125924, EPI_ISL_9125953, EPI_ISL_9125956, EPI_ISL_9125962, EPI_ISL_9125997, EPI_ISL_9126019, EPI_ISL_9126033, EPI_ISL_9126174, EPI_ISL_9126200, EPI_ISL_9126222, EPI_ISL_9126250, EPI_ISL_9126256, EPI_ISL_9126272, EPI_ISL_9126319, EPI_ISL_9126895, EPI_ISL_9126911, EPI_ISL_9126940, EPI_ISL_9126958, EPI_ISL_9126981, EPI_ISL_9127087, EPI_ISL_9127268, EPI_ISL_9127334, EPI_ISL_9127389, EPI_ISL_9127430, EPI_ISL_9127450, EPI_ISL_9127525, EPI_ISL_9127528, EPI_ISL_9127532, EPI_ISL_9127537, EPI_ISL_9127556, EPI_ISL_9127582, EPI_ISL_9127585, EPI_ISL_9127599, EPI_ISL_9127616, EPI_ISL_9127632, EPI_ISL_9127692, EPI_ISL_9127695, EPI_ISL_9127703, EPI_ISL_9127720, EPI_ISL_9127759, EPI_ISL_9127784, EPI_ISL_9127818, EPI_ISL_9127826, EPI_ISL_9127846, EPI_ISL_9127854, EPI_ISL_9127858, EPI_ISL_9127859, EPI_ISL_9127860, EPI_ISL_9127870, EPI_ISL_9127883, EPI_ISL_9127896, EPI_ISL_9127916, EPI_ISL_9127927, EPI_ISL_9127950, EPI_ISL_9127994, EPI_ISL_9128021, EPI_ISL_9128047, EPI_ISL_9128048, EPI_ISL_9128062, EPI_ISL_9128108, EPI_ISL_9128121, EPI_ISL_9128128, EPI_ISL_9128146, EPI_ISL_9128174, EPI_ISL_9128238, EPI_ISL_9128290, EPI_ISL_9128293, EPI_ISL_9128316, EPI_ISL_9128397, EPI_ISL_9128469, EPI_ISL_9128495, EPI_ISL_9128514, EPI_ISL_9128517, EPI_ISL_9128520, EPI_ISL_9128529, EPI_ISL_9128543, EPI_ISL_9128547, EPI_ISL_9128550, EPI_ISL_9128554, EPI_ISL_9128560, EPI_ISL_9128566, EPI_ISL_9128581, EPI_ISL_9128633, EPI_ISL_9128637, EPI_ISL_9128642, EPI_ISL_9128645, EPI_ISL_9128654, EPI_ISL_9128672, EPI_ISL_9128676, EPI_ISL_9128683, EPI_ISL_9128684, EPI_ISL_9128742, EPI_ISL_9128757, EPI_ISL_9128776, EPI_ISL_9128780, EPI_ISL_9128791, EPI_ISL_9128798, EPI_ISL_9128808, EPI_ISL_9128866, EPI_ISL_9128875, EPI_ISL_9128942, EPI_ISL_9129017, EPI_ISL_9129030, EPI_ISL_9129040, EPI_ISL_9129082, EPI_ISL_9129114, EPI_ISL_9131411, EPI_ISL_9131436, EPI_ISL_9131517, EPI_ISL_9131524, EPI_ISL_9131566, EPI_ISL_9131573, EPI_ISL_9131589, EPI_ISL_9131593, EPI_ISL_9131596, EPI_ISL_9131606, EPI_ISL_9131666, EPI_ISL_9131711, EPI_ISL_9131720, EPI_ISL_9131759, EPI_ISL_9131773, EPI_ISL_9131776, EPI_ISL_9131781, EPI_ISL_9131809, EPI_ISL_9134208, EPI_ISL_9134212, EPI_ISL_9134228, EPI_ISL_9134243, EPI_ISL_9134250, EPI_ISL_9134265, EPI_ISL_9134320, EPI_ISL_9134618, EPI_ISL_9134663			
see above	Department of Bacteria, Parasites and Fungi, Statens Serum Institut, Copenhagen, Denmark	Statens Serum Institut Bioinformatics and Microbial Genomics	Danish Covid-19 Genome Consortium

We gratefully acknowledge the following Authors from the Originating laboratories responsible for obtaining the specimens, as well as the Submitting laboratories where the genome data were generated and shared via GISAID, on which this research is based.

All Submitters of data may be contacted directly via www.gisaid.org

Authors are sorted alphabetically.

Acknowledgement EPI_SET Identifier: EPI_SET_20220527yx

Accession ID	Originating Laboratory	Submitting Laboratory	Authors
EPI_ISL_9119558, EPI_ISL_9119568, EPI_ISL_9119573, EPI_ISL_9119578, EPI_ISL_9119581, EPI_ISL_9119593, EPI_ISL_9119627, EPI_ISL_9119639, EPI_ISL_9119647, EPI_ISL_9119649, EPI_ISL_9119656, EPI_ISL_9119658, EPI_ISL_9119659, EPI_ISL_9119660, EPI_ISL_9119675, EPI_ISL_9119682, EPI_ISL_9119686, EPI_ISL_9119687, EPI_ISL_9119698, EPI_ISL_9119703, EPI_ISL_9119704, EPI_ISL_9119714, EPI_ISL_9119717, EPI_ISL_9119719, EPI_ISL_9119737, EPI_ISL_9119745, EPI_ISL_9119747, EPI_ISL_9119748, EPI_ISL_9119765, EPI_ISL_9119771, EPI_ISL_9119794, EPI_ISL_9119821, EPI_ISL_9119823, EPI_ISL_9119826, EPI_ISL_9119831, EPI_ISL_9119836, EPI_ISL_9119840, EPI_ISL_9119858, EPI_ISL_9119865, EPI_ISL_9119874, EPI_ISL_9119886, EPI_ISL_9119896, EPI_ISL_9119900, EPI_ISL_9119903, EPI_ISL_9119908, EPI_ISL_9119909, EPI_ISL_9119916, EPI_ISL_9119924, EPI_ISL_9119925, EPI_ISL_9119932, EPI_ISL_9119948, EPI_ISL_9119956, EPI_ISL_9119959, EPI_ISL_9119970, EPI_ISL_9119971, EPI_ISL_9119975, EPI_ISL_9119979, EPI_ISL_9119983, EPI_ISL_9119991, EPI_ISL_9119996, EPI_ISL_9120002, EPI_ISL_9120010, EPI_ISL_9120012, EPI_ISL_9120019, EPI_ISL_9120021, EPI_ISL_9120022, EPI_ISL_9120043, EPI_ISL_9120044, EPI_ISL_9120050, EPI_ISL_9120051, EPI_ISL_9120061, EPI_ISL_9120088, EPI_ISL_9120099, EPI_ISL_9120101, EPI_ISL_9120107, EPI_ISL_9120108, EPI_ISL_9120112, EPI_ISL_9120116, EPI_ISL_9120120, EPI_ISL_9120127, EPI_ISL_9120140, EPI_ISL_9120141, EPI_ISL_9120147, EPI_ISL_9120153, EPI_ISL_9120155, EPI_ISL_9120164, EPI_ISL_9120165, EPI_ISL_9120166, EPI_ISL_9120169, EPI_ISL_9120176, EPI_ISL_9120179, EPI_ISL_9120184, EPI_ISL_9120190, EPI_ISL_9120191, EPI_ISL_9120198, EPI_ISL_9120201, EPI_ISL_9120238, EPI_ISL_9120241, EPI_ISL_9120255, EPI_ISL_9120256, EPI_ISL_9120267, EPI_ISL_9120268, EPI_ISL_9120473, EPI_ISL_9120475, EPI_ISL_9120481, EPI_ISL_9120483, EPI_ISL_9120490, EPI_ISL_9120496, EPI_ISL_9120498, EPI_ISL_9120499, EPI_ISL_9120500, EPI_ISL_9120506, EPI_ISL_9120510, EPI_ISL_9120517, EPI_ISL_9120520, EPI_ISL_9120523, EPI_ISL_9120528, EPI_ISL_9120532, EPI_ISL_9120539, EPI_ISL_9120542, EPI_ISL_9120550, EPI_ISL_9120551, EPI_ISL_9120559, EPI_ISL_9120560, EPI_ISL_9120562, EPI_ISL_9120563, EPI_ISL_9120568, EPI_ISL_9120573, EPI_ISL_9120585, EPI_ISL_9120595, EPI_ISL_9120604, EPI_ISL_9120605, EPI_ISL_9120612, EPI_ISL_9120613, EPI_ISL_9120615, EPI_ISL_9120621, EPI_ISL_9120633, EPI_ISL_9120645, EPI_ISL_9120648, EPI_ISL_9121037, EPI_ISL_9121041, EPI_ISL_9121047, EPI_ISL_9121053, EPI_ISL_9121061, EPI_ISL_9121063, EPI_ISL_9121064, EPI_ISL_9121067, EPI_ISL_9121072, EPI_ISL_9121075, EPI_ISL_9121079, EPI_ISL_9121106, EPI_ISL_9121116, EPI_ISL_9121120, EPI_ISL_9121124, EPI_ISL_9121127, EPI_ISL_9121128, EPI_ISL_9121132, EPI_ISL_9121137, EPI_ISL_9121171, EPI_ISL_9121174, EPI_ISL_9121177, EPI_ISL_9121187, EPI_ISL_9121188, EPI_ISL_9121191, EPI_ISL_9121197, EPI_ISL_9121205, EPI_ISL_9121208, EPI_ISL_9121216, EPI_ISL_9121217, EPI_ISL_9121225, EPI_ISL_9121229, EPI_ISL_9121231, EPI_ISL_9121244, EPI_ISL_9121245, EPI_ISL_9121253, EPI_ISL_9121257, EPI_ISL_9121272, EPI_ISL_9121287, EPI_ISL_9121292, EPI_ISL_9121293, EPI_ISL_9121297, EPI_ISL_9121311, EPI_ISL_9121313, EPI_ISL_9121314, EPI_ISL_9121317, EPI_ISL_9121329, EPI_ISL_9121344, EPI_ISL_9121348, EPI_ISL_9121349, EPI_ISL_9121356, EPI_ISL_9121365, EPI_ISL_9121370, EPI_ISL_9121385, EPI_ISL_9121398, EPI_ISL_9121403, EPI_ISL_9121404, EPI_ISL_9121413, EPI_ISL_9121414, EPI_ISL_9121423, EPI_ISL_9121428, EPI_ISL_9121438, EPI_ISL_9121443, EPI_ISL_9121446, EPI_ISL_9121451, EPI_ISL_9121469, EPI_ISL_9121472, EPI_ISL_9121484, EPI_ISL_9121497, EPI_ISL_9121498, EPI_ISL_9121501, EPI_ISL_9121502, EPI_ISL_9121503, EPI_ISL_9121509, EPI_ISL_9121510, EPI_ISL_9121514, EPI_ISL_9121526, EPI_ISL_9121531, EPI_ISL_9121547, EPI_ISL_9121548, EPI_ISL_9121550, EPI_ISL_9121553, EPI_ISL_9121556, EPI_ISL_9121559, EPI_ISL_9121561, EPI_ISL_9121568, EPI_ISL_9121570, EPI_ISL_9121575, EPI_ISL_9121578, EPI_ISL_9121584, EPI_ISL_9121588, EPI_ISL_9121594, EPI_ISL_9121598, EPI_ISL_9121615, EPI_ISL_9121618, EPI_ISL_9121619, EPI_ISL_9121623, EPI_ISL_9121624, EPI_ISL_9121628, EPI_ISL_9121640, EPI_ISL_9121645, EPI_ISL_9121692, EPI_ISL_9121698, EPI_ISL_9121726, EPI_ISL_9121728, EPI_ISL_9121738, EPI_ISL_9121739, EPI_ISL_9121743, EPI_ISL_9121745, EPI_ISL_9121747, EPI_ISL_9121748, EPI_ISL_9121750, EPI_ISL_9121758, EPI_ISL_9121759, EPI_ISL_9121768, EPI_ISL_9121769, EPI_ISL_9121772, EPI_ISL_9121786, EPI_ISL_9121789, EPI_ISL_9121791, EPI_ISL_9121832, EPI_ISL_9121833, EPI_ISL_9121836, EPI_ISL_9121838, EPI_ISL_9121851, EPI_ISL_9121852, EPI_ISL_9121854, EPI_ISL_9121855, EPI_ISL_9121872, EPI_ISL_9121874, EPI_ISL_9121876, EPI_ISL_9121882, EPI_ISL_9121888, EPI_ISL_9121890, EPI_ISL_9121897, EPI_ISL_9121904, EPI_ISL_9122021, EPI_ISL_9122025, EPI_ISL_9122029, EPI_ISL_9122081, EPI_ISL_9122088, EPI_ISL_9122090, EPI_ISL_9122096, EPI_ISL_9122097, EPI_ISL_9122098, EPI_ISL_9122104, EPI_ISL_9122108, EPI_ISL_9122109, EPI_ISL_9122113, EPI_ISL_9122126, EPI_ISL_9122131, EPI_ISL_9122134, EPI_ISL_9122139, EPI_ISL_9122140, EPI_ISL_9122141, EPI_ISL_9122144, EPI_ISL_9122148, EPI_ISL_9122155, EPI_ISL_9122158, EPI_ISL_9122161, EPI_ISL_9122162, EPI_ISL_9122165, EPI_ISL_9122167, EPI_ISL_9122168, EPI_ISL_9122915, EPI_ISL_9122916, EPI_ISL_9122922, EPI_ISL_9122924, EPI_ISL_9122930, EPI_ISL_9122932, EPI_ISL_9122935, EPI_ISL_9122940, EPI_ISL_9122944, EPI_ISL_9122945, EPI_ISL_9122946, EPI_ISL_9122947, EPI_ISL_9122958, EPI_ISL_9122974, EPI_ISL_9122982, EPI_ISL_9122987, EPI_ISL_9122998, EPI_ISL_9123123, EPI_ISL_9123135, EPI_ISL_9123137, EPI_ISL_9123139, EPI_ISL_9123140, EPI_ISL_9123142, EPI_ISL_9123144, EPI_ISL_9123156, EPI_ISL_9123171, EPI_ISL_9123172, EPI_ISL_9123175, EPI_ISL_9123179, EPI_ISL_9123191, EPI_ISL_9123193, EPI_ISL_9123201, EPI_ISL_9123215, EPI_ISL_9123216, EPI_ISL_9123229, EPI_ISL_9123234, EPI_ISL_9123236, EPI_ISL_9123248, EPI_ISL_9123253, EPI_ISL_9123260, EPI_ISL_9123263, EPI_ISL_9123264, EPI_ISL_9123267, EPI_ISL_9123272, EPI_ISL_9123273, EPI_ISL_9123276, EPI_ISL_9123290, EPI_ISL_9123292, EPI_ISL_9123295, EPI_ISL_9123299, EPI_ISL_9123305, EPI_ISL_9123310, EPI_ISL_9123312, EPI_ISL_9123313, EPI_ISL_9123318, EPI_ISL_9123343, EPI_ISL_9123351, EPI_ISL_9123362, EPI_ISL_9123363, EPI_ISL_9123366, EPI_ISL_9123369, EPI_ISL_9123373, EPI_ISL_9123378, EPI_ISL_9123380, EPI_ISL_9123382, EPI_ISL_9123383, EPI_ISL_9123392, EPI_ISL_9123398, EPI_ISL_9123399, EPI_ISL_9123410, EPI_ISL_9123416, EPI_ISL_9123426, EPI_ISL_9123430, EPI_ISL_9123433, EPI_ISL_9123438, EPI_ISL_9123443, EPI_ISL_9123447, EPI_ISL_9123454, EPI_ISL_9123455, EPI_ISL_9123464, EPI_ISL_9123479, EPI_ISL_9123480, EPI_ISL_9123488, EPI_ISL_9123490, EPI_ISL_9123491, EPI_ISL_9123493, EPI_ISL_9123501, EPI_ISL_9123506, EPI_ISL_9123515, EPI_ISL_9123529, EPI_ISL_9123533, EPI_ISL_9123537, EPI_ISL_9123547, EPI_ISL_9123549, EPI_ISL_9123553, EPI_ISL_9123554, EPI_ISL_9123560, EPI_ISL_9123563, EPI_ISL_9123755, EPI_ISL_9123757, EPI_ISL_9123759, EPI_ISL_9123761, EPI_ISL_9123763, EPI_ISL_9123770, EPI_ISL_9123772, EPI_ISL_9123773, EPI_ISL_9123779, EPI_ISL_9123785, EPI_ISL_9123786, EPI_ISL_9123792, EPI_ISL_9123797, EPI_ISL_9123994, EPI_ISL_9124003, EPI_ISL_9124004, EPI_ISL_9124005, EPI_ISL_9124008, EPI_ISL_9124009, EPI_ISL_9124018, EPI_ISL_9124022, EPI_ISL_9124027, EPI_ISL_9124030, EPI_ISL_9124031, EPI_ISL_9124034, EPI_ISL_9124046, EPI_ISL_9124049, EPI_ISL_9124080, EPI_ISL_9124097, EPI_ISL_9124100, EPI_ISL_9124120, EPI_ISL_9124132, EPI_ISL_9124137, EPI_ISL_9124158, EPI_ISL_9124166, EPI_ISL_9124168, EPI_ISL_9124267, EPI_ISL_9124276, EPI_ISL_9124299, EPI_ISL_9124329, EPI_ISL_9124347, EPI_ISL_9124364, EPI_ISL_9124367, EPI_ISL_9124390, EPI_ISL_9124393, EPI_ISL_9124413, EPI_ISL_9124429, EPI_ISL_9124447, EPI_ISL_9124452, EPI_ISL_9124485, EPI_ISL_9124491, EPI_ISL_9124500, EPI_ISL_9124520, EPI_ISL_9124526, EPI_ISL_9124537, EPI_ISL_9124542, EPI_ISL_9124553, EPI_ISL_9124558, EPI_ISL_9124565, EPI_ISL_9124567, EPI_ISL_9124571, EPI_ISL_9124576, EPI_ISL_9124584, EPI_ISL_9124592, EPI_ISL_9124613, EPI_ISL_9124635, EPI_ISL_9124652, EPI_ISL_9124694, EPI_ISL_9124721, EPI_ISL_9124726, EPI_ISL_9124729, EPI_ISL_9124733			
see above	Department of Bacteria, Parasites and Fungi, Statens Serum Institut, Copenhagen, Denmark	Statens Serum Institut Bioinformatics and Microbial Genomics	Danish Covid-19 Genome Consortium

We gratefully acknowledge the following Authors from the Originating laboratories responsible for obtaining the specimens, as well as the Submitting laboratories where the genome data were generated and shared via GISAID, on which this research is based.

All Submitters of data may be contacted directly via www.gisaid.org

Authors are sorted alphabetically.

Accession ID	Originating Laboratory	Submitting Laboratory	Authors
EPI_ISL_9128636, EPI_ISL_9128638, EPI_ISL_9128664, EPI_ISL_9128670, EPI_ISL_9128679, EPI_ISL_9128751, EPI_ISL_9128771, EPI_ISL_9128786, EPI_ISL_9128793, EPI_ISL_9128800, EPI_ISL_9128817, EPI_ISL_9128835, EPI_ISL_9128869, EPI_ISL_9128878, EPI_ISL_9128919, EPI_ISL_9128934, EPI_ISL_9128970, EPI_ISL_9128973, EPI_ISL_9129020, EPI_ISL_9129036, EPI_ISL_9129072, EPI_ISL_9129100, EPI_ISL_9129109, EPI_ISL_9131406, EPI_ISL_9131409, EPI_ISL_9131422, EPI_ISL_9131438, EPI_ISL_9131446, EPI_ISL_9131453, EPI_ISL_9131484, EPI_ISL_9131520, EPI_ISL_9131559, EPI_ISL_9131576, EPI_ISL_9131599, EPI_ISL_9131617, EPI_ISL_9131623, EPI_ISL_9131632, EPI_ISL_9131647, EPI_ISL_9131684, EPI_ISL_9131727, EPI_ISL_9131786, EPI_ISL_9132000, EPI_ISL_9134248, EPI_ISL_9134301, EPI_ISL_9134316, EPI_ISL_9134323, EPI_ISL_9134326, EPI_ISL_9134332, EPI_ISL_9134627, EPI_ISL_9134644			
see above	Department of Bacteria, Parasites and Fungi, Statens Serum Institut, Copenhagen, Denmark	Statens Serum Institut Bioinformatics and Microbial Genomics	Danish Covid-19 Genome Consortium

We gratefully acknowledge the following Authors from the Originating laboratories responsible for obtaining the specimens, as well as the Submitting laboratories where the genome data were generated and shared via GISAID, on which this research is based.

All Submitters of data may be contacted directly via www.gisaid.org

Authors are sorted alphabetically.

Accession ID	Originating Laboratory	Submitting Laboratory	Authors
EPI_ISL_9127795, EPI_ISL_9127798, EPI_ISL_9127805, EPI_ISL_9127816, EPI_ISL_9127820, EPI_ISL_9127836, EPI_ISL_9127839, EPI_ISL_9127844, EPI_ISL_9127856, EPI_ISL_9127865, EPI_ISL_9127868, EPI_ISL_9127879, EPI_ISL_9127905, EPI_ISL_9127933, EPI_ISL_9127938, EPI_ISL_9128031, EPI_ISL_9128034, EPI_ISL_9128042, EPI_ISL_9128074, EPI_ISL_9128093, EPI_ISL_9128099, EPI_ISL_9128116, EPI_ISL_9128118, EPI_ISL_9128151, EPI_ISL_9128180, EPI_ISL_9128191, EPI_ISL_9128207, EPI_ISL_9128222, EPI_ISL_9128224, EPI_ISL_9128230, EPI_ISL_9128241, EPI_ISL_9128276, EPI_ISL_9128282, EPI_ISL_9128285, EPI_ISL_9128299, EPI_ISL_9128336, EPI_ISL_9128339, EPI_ISL_9128351, EPI_ISL_9128371, EPI_ISL_9128382, EPI_ISL_9128429, EPI_ISL_9128431, EPI_ISL_9128471, EPI_ISL_9128476, EPI_ISL_9128482, EPI_ISL_9128502, EPI_ISL_9128504, EPI_ISL_9128523, EPI_ISL_9128590, EPI_ISL_9128630			
see above	Department of Bacteria, Parasites and Fungi, Statens Serum Institut, Copenhagen, Denmark	Statens Serum Institut Bioinformatics and Microbial Genomics	Danish Covid-19 Genome Consortium

We gratefully acknowledge the following Authors from the Originating laboratories responsible for obtaining the specimens, as well as the Submitting laboratories where the genome data were generated and shared via GISAID, on which this research is based.

All Submitters of data may be contacted directly via www.gisaid.org

Authors are sorted alphabetically.

Acknowledgement EPI_SET Identifier: EPI_SET_20220527go

Accession ID	Originating Laboratory	Submitting Laboratory	Authors
EPI_ISL_9126266, EPI_ISL_9126274, EPI_ISL_9126284, EPI_ISL_9126293, EPI_ISL_9126498, EPI_ISL_9126500, EPI_ISL_9126502, EPI_ISL_9126843, EPI_ISL_9126853, EPI_ISL_9126861, EPI_ISL_9126898, EPI_ISL_9126908, EPI_ISL_9126922, EPI_ISL_9126946, EPI_ISL_9126963, EPI_ISL_9126971, EPI_ISL_9127005, EPI_ISL_9127035, EPI_ISL_9127065, EPI_ISL_9127067, EPI_ISL_9127071, EPI_ISL_9127146, EPI_ISL_9127285, EPI_ISL_9127289, EPI_ISL_9127307, EPI_ISL_9127313, EPI_ISL_9127324, EPI_ISL_9127343, EPI_ISL_9127346, EPI_ISL_9127383, EPI_ISL_9127392, EPI_ISL_9127420, EPI_ISL_9127424, EPI_ISL_9127439, EPI_ISL_9127447, EPI_ISL_9127461, EPI_ISL_9127481, EPI_ISL_9127489, EPI_ISL_9127494, EPI_ISL_9127518, EPI_ISL_9127527, EPI_ISL_9127540, EPI_ISL_9127618, EPI_ISL_9127624, EPI_ISL_9127649, EPI_ISL_9127666, EPI_ISL_9127706, EPI_ISL_9127742, EPI_ISL_9127756, EPI_ISL_9127792			
see above	Department of Bacteria, Parasites and Fungi, Statens Serum Institut, Copenhagen, Denmark	Statens Serum Institut Bioinformatics and Microbial Genomics	Danish Covid-19 Genome Consortium

We gratefully acknowledge the following Authors from the Originating laboratories responsible for obtaining the specimens, as well as the Submitting laboratories where the genome data were generated and shared via GISAID, on which this research is based.

All Submitters of data may be contacted directly via www.gisaid.org

Authors are sorted alphabetically.

Accession ID	Originating Laboratory	Submitting Laboratory	Authors
EPI_ISL_9124736, EPI_ISL_9124790, EPI_ISL_9124796, EPI_ISL_9124818, EPI_ISL_9124821, EPI_ISL_9124826, EPI_ISL_9124841, EPI_ISL_9124844, EPI_ISL_9124878, EPI_ISL_9124926, EPI_ISL_9124929, EPI_ISL_9124932, EPI_ISL_9124937, EPI_ISL_9124950, EPI_ISL_9124958, EPI_ISL_9124977, EPI_ISL_9124982, EPI_ISL_9125291, EPI_ISL_9125295, EPI_ISL_9125388, EPI_ISL_9125410, EPI_ISL_9125424, EPI_ISL_9125427, EPI_ISL_9125449, EPI_ISL_9125457, EPI_ISL_9125472, EPI_ISL_9125481, EPI_ISL_9125483, EPI_ISL_9125526, EPI_ISL_9125534, EPI_ISL_9125881, EPI_ISL_9125920, EPI_ISL_9125934, EPI_ISL_9125937, EPI_ISL_9125955, EPI_ISL_9125964, EPI_ISL_9125994, EPI_ISL_9126002, EPI_ISL_9126005, EPI_ISL_9126009, EPI_ISL_9126025, EPI_ISL_9126042, EPI_ISL_9126086, EPI_ISL_9126100, EPI_ISL_9126117, EPI_ISL_9126131, EPI_ISL_9126143, EPI_ISL_9126145, EPI_ISL_9126225, EPI_ISL_9126247			
see above	Department of Bacteria, Parasites and Fungi, Statens Serum Institut, Copenhagen, Denmark	Statens Serum Institut Bioinformatics and Microbial Genomics	Danish Covid-19 Genome Consortium

We gratefully acknowledge the following Authors from the Originating laboratories responsible for obtaining the specimens, as well as the Submitting laboratories where the genome data were generated and shared via GISAID, on which this research is based.

All Submitters of data may be contacted directly via www.gisaid.org

Authors are sorted alphabetically.

Accession ID	Originating Laboratory	Submitting Laboratory	Authors
EPI_ISL_9123002, EPI_ISL_9123003, EPI_ISL_9123004	Lifebrain Covid Labor GmbH	Lifebrain Covid Labor GmbH	Alexandra Wagner; Anna Edermayr; Filip Sima

We gratefully acknowledge the following Authors from the Originating laboratories responsible for obtaining the specimens, as well as the Submitting laboratories where the genome data were generated and shared via GISAID, on which this research is based.

All Submitters of data may be contacted directly via www.gisaid.org

Authors are sorted alphabetically.

Accession ID	Originating Laboratory	Submitting Laboratory	Authors
EPI_ISL_9143636	Hrvatski zavod za javno zdravstvo	Hrvatski zavod za javno zdravstvo	Anita Jurić; Dragan Jurić; Irena Tabain; Ivana Ferenčak; Josipa Kuzle

We gratefully acknowledge the following Authors from the Originating laboratories responsible for obtaining the specimens, as well as the Submitting laboratories where the genome data were generated and shared via GISAID, on which this research is based.

All Submitters of data may be contacted directly via www.gisaid.org

Authors are sorted alphabetically.

Accession ID	Originating Laboratory	Submitting Laboratory	Authors
EPI_ISL_9143643	Zavod za javno zdravstvo Istarske županije	Hrvatski zavod za javno zdravstvo	Anita Jurić; Dragan Jurić; Irena Tabain; Ivana Ferenčak; Josipa Kuzle

We gratefully acknowledge the following Authors from the Originating laboratories responsible for obtaining the specimens, as well as the Submitting laboratories where the genome data were generated and shared via GISAID, on which this research is based.

All Submitters of data may be contacted directly via www.gisaid.org

Authors are sorted alphabetically.

Acknowledgement EPI_SET Identifier: EPI_SET_20220527xb

Accession ID	Originating Laboratory	Submitting Laboratory	Authors
EPI_ISL_9143539, EPI_ISL_9143540	Zavod za javno zdravstvo Ličko-senjske županije	Hrvatski zavod za javno zdravstvo	Anita Jurić; Dragan Jurić; Irena Tabain; Ivana Ferenčak; Josipa Kuzle

We gratefully acknowledge the following Authors from the Originating laboratories responsible for obtaining the specimens, as well as the Submitting laboratories where the genome data were generated and shared via GISAID, on which this research is based.

All Submitters of data may be contacted directly via www.gisaid.org

Authors are sorted alphabetically.

Accession ID	Originating Laboratory	Submitting Laboratory	Authors
EPI_ISL_9143535, EPI_ISL_9143536, EPI_ISL_9143537, EPI_ISL_9143538	Zavod za javno zdravstvo Šibensko- Kninske županije	Hrvatski zavod za javno zdravstvo	Anita Jurić; Dragan Jurić; Irena Tabain; Ivana Ferenčak; Josipa Kuzle

We gratefully acknowledge the following Authors from the Originating laboratories responsible for obtaining the specimens, as well as the Submitting laboratories where the genome data were generated and shared via GISAID, on which this research is based.

All Submitters of data may be contacted directly via www.gisaid.org

Authors are sorted alphabetically.

Accession ID	Originating Laboratory	Submitting Laboratory	Authors
EPI_ISL_9143619	Hrvatski zavod za javno zdravstvo	Hrvatski zavod za javno zdravstvo	Anita Jurić; Dragan Jurić; Irena Tabain; Ivana Ferenčak; Josipa Kuzle

We gratefully acknowledge the following Authors from the Originating laboratories responsible for obtaining the specimens, as well as the Submitting laboratories where the genome data were generated and shared via GISAID, on which this research is based.

All Submitters of data may be contacted directly via www.gisaid.org

Authors are sorted alphabetically.

Accession ID	Originating Laboratory	Submitting Laboratory	Authors
EPI_ISL_9143619	Hrvatski zavod za javno zdravstvo	Hrvatski zavod za javno zdravstvo	Anita Jurić; Dragan Jurić; Irena Tabain; Ivana Ferenčak; Josipa Kuzle

We gratefully acknowledge the following Authors from the Originating laboratories responsible for obtaining the specimens, as well as the Submitting laboratories where the genome data were generated and shared via GISAID, on which this research is based.

All Submitters of data may be contacted directly via www.gisaid.org

Authors are sorted alphabetically.

Accession ID	Originating Laboratory	Submitting Laboratory	Authors
EPI_ISL_9143611, EPI_ISL_9143612, EPI_ISL_9143614	Hrvatski zavod za javno zdravstvo	Hrvatski zavod za javno zdravstvo	Anita Jurić; Dragan Jurić; Irena Tabain; Ivana Ferenčak; Josipa Kuzle

We gratefully acknowledge the following Authors from the Originating laboratories responsible for obtaining the specimens, as well as the Submitting laboratories where the genome data were generated and shared via GISAID, on which this research is based.

All Submitters of data may be contacted directly via www.gisaid.org

Authors are sorted alphabetically.

Accession ID	Originating Laboratory	Submitting Laboratory	Authors
EPI_ISL_9143613, EPI_ISL_9143615, EPI_ISL_9143616, EPI_ISL_9143617, EPI_ISL_9143618, EPI_ISL_9143620, EPI_ISL_9143621, EPI_ISL_9143622, EPI_ISL_9143623, EPI_ISL_9143624, EPI_ISL_9143625, EPI_ISL_9143626, EPI_ISL_9143627, EPI_ISL_9143628, EPI_ISL_9143629, EPI_ISL_9143630, EPI_ISL_9143631, EPI_ISL_9143632, EPI_ISL_9143633, EPI_ISL_9143634, EPI_ISL_9143635, EPI_ISL_9143637, EPI_ISL_9143638, EPI_ISL_9143639, EPI_ISL_9143640, EPI_ISL_9143641, EPI_ISL_9143642, EPI_ISL_9143644			
see above	Hrvatski zavod za javno zdravstvo	Hrvatski zavod za javno zdravstvo	Anita Jurić; Dragan Jurić; Irena Tabain; Ivana Ferenčak; Josipa Kuzle
EPI_ISL_9143679, EPI_ISL_9143680, EPI_ISL_9143681, EPI_ISL_9143682, EPI_ISL_9143683, EPI_ISL_9143684, EPI_ISL_9143685, EPI_ISL_9143686, EPI_ISL_9143687, EPI_ISL_9143688, EPI_ISL_9143689, EPI_ISL_9143690, EPI_ISL_9143691, EPI_ISL_9143692, EPI_ISL_9143693, EPI_ISL_9143694, EPI_ISL_9143695, EPI_ISL_9143696, EPI_ISL_9143697, EPI_ISL_9143698, EPI_ISL_9143699, EPI_ISL_9143700, EPI_ISL_9143701, EPI_ISL_9143702, EPI_ISL_9143703, EPI_ISL_9143704, EPI_ISL_9143705, EPI_ISL_9143706, EPI_ISL_9143707, EPI_ISL_9143708, EPI_ISL_9143709, EPI_ISL_9143710, EPI_ISL_9143711, EPI_ISL_9143712, EPI_ISL_9143713, EPI_ISL_9143714, EPI_ISL_9143715, EPI_ISL_9143716, EPI_ISL_9143717, EPI_ISL_9143718, EPI_ISL_9143719, EPI_ISL_9143720, EPI_ISL_9143721, EPI_ISL_9143722, EPI_ISL_9143723, EPI_ISL_9143724, EPI_ISL_9143725, EPI_ISL_9143726, EPI_ISL_9143727, EPI_ISL_9143728, EPI_ISL_9143729, EPI_ISL_9143730, EPI_ISL_9143731, EPI_ISL_9143732, EPI_ISL_9143734, EPI_ISL_9143735, EPI_ISL_9143736, EPI_ISL_9143737, EPI_ISL_9143738, EPI_ISL_9143739			
see above	Klinika za infektivne bolesti "Dr. Fran Mihaljević"	Hrvatski zavod za javno zdravstvo	Anita Jurić; Dragan Jurić; Irena Tabain; Ivana Ferenčak; Josipa Kuzle

We gratefully acknowledge the following Authors from the Originating laboratories responsible for obtaining the specimens, as well as the Submitting laboratories where the genome data were generated and shared via GISAID, on which this research is based.

All Submitters of data may be contacted directly via www.gisaid.org

Authors are sorted alphabetically.

Acknowledgement EPI_SET Identifier: EPI_SET_20220527bt

Accession ID	Originating Laboratory	Submitting Laboratory	Authors
EPI_ISL_9106321, EPI_ISL_9106341, EPI_ISL_9106347	Limbach - MVZ Labor Ludwigsburg	Robert Koch Institute	
EPI_ISL_9107465, EPI_ISL_9107469, EPI_ISL_9107476, EPI_ISL_9107494, EPI_ISL_9108599	MVZ Labor Dr. Limbach & Kollegen GbR	Robert Koch Institute	
EPI_ISL_9107666, EPI_ISL_9107668	SYNLAB MVZ Leinfelden-Echterdingen	Robert Koch Institute	

We gratefully acknowledge the following Authors from the Originating laboratories responsible for obtaining the specimens, as well as the Submitting laboratories where the genome data were generated and shared via GISAID, on which this research is based.

All Submitters of data may be contacted directly via www.gisaid.org

Authors are sorted alphabetically.

Accession ID	Originating Laboratory	Submitting Laboratory	Authors
EPI_ISL_9110249	IMD - Institut für Medizinische Diagnostik Berlin-Potsdam	Robert Koch Institute	
EPI_ISL_9108810, EPI_ISL_9108829, EPI_ISL_9108835, EPI_ISL_9108852	MDI Limbach Berlin GmbH; MVZ Labor Berlin	Robert Koch Institute	

We gratefully acknowledge the following Authors from the Originating laboratories responsible for obtaining the specimens, as well as the Submitting laboratories where the genome data were generated and shared via GISAID, on which this research is based.

All Submitters of data may be contacted directly via www.gisaid.org

Authors are sorted alphabetically.

Accession ID	Originating Laboratory	Submitting Laboratory	Authors
EPI_ISL_9108907	MVZ Medizinisches Labor Hannover GmbH	Robert Koch Institute	

We gratefully acknowledge the following Authors from the Originating laboratories responsible for obtaining the specimens, as well as the Submitting laboratories where the genome data were generated and shared via GISAID, on which this research is based.

All Submitters of data may be contacted directly via www.gisaid.org

Authors are sorted alphabetically.

Accession ID	Originating Laboratory	Submitting Laboratory	Authors
EPI_ISL_9107511, EPI_ISL_9107535	Clemenshospital - Alexianer Clemenshospital Münster	Robert Koch Institute	
EPI_ISL_9109828	MVZ Labor Dr. Quade & Kollegen GmbH	Robert Koch Institute	

We gratefully acknowledge the following Authors from the Originating laboratories responsible for obtaining the specimens, as well as the Submitting laboratories where the genome data were generated and shared via GISAID, on which this research is based.

All Submitters of data may be contacted directly via www.gisaid.org

Authors are sorted alphabetically.

Accession ID	Originating Laboratory	Submitting Laboratory	Authors
EPI_ISL_9111052, EPI_ISL_9111116	A.S.L. ALESSANDRIA	Fondazione del Piemonte per l'Oncologia IRCCS	Antonino Sottile; Giorgio Giardina; Paola Marino; Silvia Brossa
EPI_ISL_9110835, EPI_ISL_9110844, EPI_ISL_9110860, EPI_ISL_9110864	Arcispedale Santa Maria Nuova Autoimmunità Allergologia e Biotecnologie Innovative	Istituto Zooprofilattico Sperimentale della Lombardia e dell'Emilia Romagna (IZSLER), Risk Analysis and Genomic Epidemiology Unit	Alessandro Zerbini; Erika Scaltriti; Ilaria Menozzi; Lucia Belloni; Marina Morganti; Stefania Croci; Stefano Pongolini
EPI_ISL_9110686, EPI_ISL_9110695	Azienda Sanitaria Locale di Piacenza - Presidio Ospedaliero - Laboratorio di Microbiologia	Istituto Zooprofilattico Sperimentale della Lombardia e dell'Emilia Romagna (IZSLER), Risk Analysis and Genomic Epidemiology Unit	Erika Scaltriti; Ilaria Menozzi; Lo Cascio Giuliana; Marina Morganti; Peroni Irene; Schiavo Roberta; Stefano Pongolini
EPI_ISL_9110772	Universita di Parma Laboratorio di Igiene e Sanita Pubblica	Istituto Zooprofilattico Sperimentale della Lombardia e dell'Emilia Romagna (IZSLER), Risk Analysis and Genomic Epidemiology Unit	Erika Scaltriti; Ilaria Menozzi; Marina Morganti; Stefano Pongolini

We gratefully acknowledge the following Authors from the Originating laboratories responsible for obtaining the specimens, as well as the Submitting laboratories where the genome data were generated and shared via GISAID, on which this research is based.

All Submitters of data may be contacted directly via www.gisaid.org

Authors are sorted alphabetically.

Accession ID	Originating Laboratory	Submitting Laboratory	Authors
EPI_ISL_9139390	Microvida	Microvida	Jaco J. Verweij; Joep J. J. M. Stohr; Suzan D. Pas

We gratefully acknowledge the following Authors from the Originating laboratories responsible for obtaining the specimens, as well as the Submitting laboratories where the genome data were generated and shared via GISAID, on which this research is based.

All Submitters of data may be contacted directly via www.gisaid.org

Authors are sorted alphabetically.

Accession ID	Originating Laboratory	Submitting Laboratory	Authors
EPI_ISL_9129404	Lighthouse Lab in Milton Keynes	Wellcome Sanger Institute for the COVID-19 Genomics UK (COG-UK) Consortium	Cordelia Langford; David K. Jackson; Dominic Kwiatkowski; Ewan Harrison; Ian Johnston; Jeffrey Barrett; John Sillitoe on behalf of the Wellcome Sanger Institute COVID-19 Surveillance Team; Roberto Amato; Sonia Goncalves; The Lighthouse Lab in Milton Keynes and Alex Alderton

We gratefully acknowledge the following Authors from the Originating laboratories responsible for obtaining the specimens, as well as the Submitting laboratories where the genome data were generated and shared via GISAID, on which this research is based.

All Submitters of data may be contacted directly via www.gisaid.org

Authors are sorted alphabetically.

Acknowledgement EPI_SET Identifier: EPI_SET_20220527qv

Accession ID	Originating Laboratory	Submitting Laboratory	Authors
EPI_ISL_9175910, EPI_ISL_9175911, EPI_ISL_9175996, EPI_ISL_9175998, EPI_ISL_9175999, EPI_ISL_9176000, EPI_ISL_9176001, EPI_ISL_9176002			
see above	BBMP- Mahadevapura	inStem, NCBS-INSACOG	Aswin SaiNarain; Dasaradhi Palakodeti; Uma Ramakrishnan
EPI_ISL_9175915, EPI_ISL_9175931, EPI_ISL_9175932, EPI_ISL_9175935, EPI_ISL_9175936, EPI_ISL_9175937, EPI_ISL_9175938, EPI_ISL_9175939, EPI_ISL_9175940, EPI_ISL_9176034, EPI_ISL_9176041, EPI_ISL_9176043, EPI_ISL_9176045, EPI_ISL_9176046			
see above	BBMP- West	inStem, NCBS-INSACOG	Aswin SaiNarain; Dasaradhi Palakodeti; Uma Ramakrishnan
EPI_ISL_9175916, EPI_ISL_9175919, EPI_ISL_9175920, EPI_ISL_9175921, EPI_ISL_9175923, EPI_ISL_9175925, EPI_ISL_9175926, EPI_ISL_9175927			
see above	BBMP- Yelahanka	inStem, NCBS-INSACOG	Aswin SaiNarain; Dasaradhi Palakodeti; Uma Ramakrishnan
EPI_ISL_9176003, EPI_ISL_9176009, EPI_ISL_9176011, EPI_ISL_9176016, EPI_ISL_9176018, EPI_ISL_9176019, EPI_ISL_9176020, EPI_ISL_9176022, EPI_ISL_9176023			
see above	BBMP-Dasarahalli	inStem, NCBS-INSACOG	Aswin SaiNarain; Dasaradhi Palakodeti; Uma Ramakrishnan
EPI_ISL_9175909	BBMP-Mahadevapura	inStem, NCBS-INSACOG	Aswin SaiNarain; Dasaradhi Palakodeti; Uma Ramakrishnan
EPI_ISL_9175941, EPI_ISL_9175943, EPI_ISL_9175946, EPI_ISL_9175947, EPI_ISL_9175949, EPI_ISL_9175953, EPI_ISL_9175954, EPI_ISL_9175955, EPI_ISL_9175956, EPI_ISL_9175957, EPI_ISL_9175959, EPI_ISL_9175960, EPI_ISL_9175961, EPI_ISL_9175965, EPI_ISL_9175967, EPI_ISL_9175970, EPI_ISL_9175971, EPI_ISL_9175973, EPI_ISL_9175978, EPI_ISL_9175979, EPI_ISL_9175981, EPI_ISL_9175982, EPI_ISL_9175983, EPI_ISL_9175984, EPI_ISL_9175985, EPI_ISL_9175988, EPI_ISL_9175991, EPI_ISL_9175992, EPI_ISL_9175993			
see above	Bangalore Airport	inStem, NCBS-INSACOG	Aswin SaiNarain; Dasaradhi Palakodeti; Uma Ramakrishnan
EPI_ISL_9176024, EPI_ISL_9176025, EPI_ISL_9176026, EPI_ISL_9176028, EPI_ISL_9176030, EPI_ISL_9176031, EPI_ISL_9176032			
see above	Bengaluru Rural	inStem, NCBS-INSACOG	Aswin SaiNarain; Dasaradhi Palakodeti; Uma Ramakrishnan
EPI_ISL_9161266, EPI_ISL_9161312, EPI_ISL_9161326, EPI_ISL_9164578	Genome Information Research Center, Research Institute for Microbial Diseases, Osaka University	Genome Information Research Center, Research Institute for Microbial Diseases, Osaka University	Daisuke Motooka; Hiroya Oki; Kentaro Tanaka; and Shota Nakamura
EPI_ISL_9166255	MapMyGenome	CSIR-Centre for Cellular and Molecular Biology-INSACOG	Amareshwar Vodapalli; Anuradha Acharya; Ara Sreenivas; Archana Bharadwaj Siva; B Himasri; Divya Tej Sowpati; Jandhyala Sai Krishna; Karthik Bharadwaj Tallapaka; Lamuk Zaveri; M. Aravind Kumar; Malini Nemalikanti; Priya Nurkurthy; Rakesh K Mishra; S.P.S. Satish; Sandhya Kiran; Shreekant Verma; Sreelekshmi MS; Sumedha Avadhanula; Surabhi Srivastava; Tulasi Nagabandi; Valli Nagalakshmi Undamatla; Vidhyadhari Methuku

We gratefully acknowledge the following Authors from the Originating laboratories responsible for obtaining the specimens, as well as the Submitting laboratories where the genome data were generated and shared via GISAID, on which this research is based.

All Submitters of data may be contacted directly via www.gisaid.org

Authors are sorted alphabetically.

Accession ID	Originating Laboratory	Submitting Laboratory	Authors
EPI_ISL_9197851	Edmonton Provincial Lab	Public Health Agency of Canada (PHAC) National Microbiology Laboratory	Buss; Croxen M; Deo A; Dieu P; E; Ferrato C; Gill K; Koleva P; Li V; Lloyd C; Lynch T; Ma R; Murphy S; Pabbaraju K; Shideler S.; Shokoples S; Skitsko T; Thayer J; Tipples G; Wong A; Yu C; Zelyas N
EPI_ISL_9196371, EPI_ISL_9197958, EPI_ISL_9197966, EPI_ISL_9197992, EPI_ISL_9198059, EPI_ISL_9198061, EPI_ISL_9198127, EPI_ISL_9198150, EPI_ISL_9198156, EPI_ISL_9198230, EPI_ISL_9198317, EPI_ISL_9198325, EPI_ISL_9198375, EPI_ISL_9198394, EPI_ISL_9198431, EPI_ISL_9198639, EPI_ISL_9198737, EPI_ISL_9198908, EPI_ISL_9199414, EPI_ISL_9199528, EPI_ISL_9199533, EPI_ISL_9199543, EPI_ISL_9199652, EPI_ISL_9199684, EPI_ISL_9199742, EPI_ISL_9199922, EPI_ISL_9199938, EPI_ISL_9200022, EPI_ISL_9200126, EPI_ISL_9200129, EPI_ISL_9200291, EPI_ISL_9200390, EPI_ISL_9200435, EPI_ISL_9200441, EPI_ISL_9200462, EPI_ISL_9200476, EPI_ISL_9200478, EPI_ISL_9200507, EPI_ISL_9200522, EPI_ISL_9200544, EPI_ISL_9200629, EPI_ISL_9200714, EPI_ISL_9200724, EPI_ISL_9200725, EPI_ISL_9200866			
see above	Houston Methodist Hospital	Houston Methodist Hospital	Akanksha Batajoo; Ilya J. Finkelstein; James J. Davis; Jessica Cambric; Jimmy Gollihar; Kristina Reppond; Madison N. Shyer; Marcus Nguyen; Matthew Ojeda Saavedra; Paul A. Christensen; Randall J. Olsen; Rashi Thakur; Regan Mangham; Richard Snehall; Robert Olson; Ryan Gadd; S. Wesley Long; Sindy Pena; and James M. Musser
EPI_ISL_9177653	Kansas Health and Environmental Lab	Kansas Health and Environmental Lab	Amanda Bradley; Carrie Welch; Gary Buruss; Jonathan Barnell; Mike Grose; Victor Anderson; and Phil Adam
EPI_ISL_9188890, EPI_ISL_9188891, EPI_ISL_9188892, EPI_ISL_9188920	Laboratory Corporation of America	Centers for Disease Control and Prevention Division of Viral Diseases, Pathogen Discovery	Amanda Douglas; Amanda Suchanek; Andrea Throop; Ayla Burns; Benjamin Rambo-Martin; Bobbi Croy; Brian Krueger; Brian Norvell; Christopher Gulvick; Christos Petropoulos; Clinton Paden; Craig Lukasik; Dakota Howard; Debbie Boles; Dhwani Batra; Duncan MacCannell; Eyad Almasri; Goran Stevovic; Howard Engler; Hrushikesh Deshmukh; Jake Humphrey; Jana Schroth; Jason Caravas; Joe Voshell; John Pruitt; Jonathan Meltzer; Jonathan Williams; Kimberly Wagner; Kristine Lacek; Lax Iyer; Lisa Pfefferle; Lyndon Tilson; Manoj Jain; Marcia Eisenberg; Mary Cristobal; Mary Williamson; Matthew Robinson; Matthew Schmerer; Michael Levandoski; Mike Sapeta; Mindy Nye; Minoo Agarwal; Mohan Kolli; Nuthawin Charoensri; Oren Cohen; Peter Cook; Prashant Gupta; Qian Zeng; Rama Ghatti; Scott Parker; Scott Ryan; Scott Sammons; Shatavia Morrison; Stanley Letovsky; Steven Ragan; Suresh Selvaraju; Susan Countryman; Susan Hicks; Suzanne Dale; Thomas Urban; Tim Kuphal; Tricia Zwiefelhofer; Tymeckia Kendall; Victoria Caban Figueroa; Vincent Drouillon; Yvette Unoarumhi
EPI_ISL_9184105, EPI_ISL_9184106, EPI_ISL_9184107, EPI_ISL_9184108	Ochsner Health	BioInfoExperts	Amy Feehan; Ben Lain; Chris Huston; David J. Nolan; Judy Crabtree; Julia-Garcia-Diaz; Lucio Miele; Rebecca Rose; Samual Moot; Susanna L. Lamers; Tessa LaFleur
EPI_ISL_9167820	SALUD DIGNA	Instituto Nacional de Medicina Genomica	Abraham Campos-Romero; Cedro-Tanda A; Cisneros-Villanueva M; Escobar-Arrazola MA; Garnica-Lopez Dora; Herrera-Montalvo LA.; Hidalgo-Miranda A; Luna-Ruiz Marco; Mendoza-Vargas A; Moreno-Camacho José Luis; Ramirez-Vega O; Rangel-DeLeon D; Reyes-Grajeda JP; Rodriguez-Gallegos Jorge; Yair Alfaro-Mora
EPI_ISL_9196278, EPI_ISL_9196321	State Laboratories Division, Hawaii State Department of Health	State Laboratories Division, Hawaii State Department of Health	Ayana Garnet; Briana Ofilas; Cheryl-lynn Daquip; Cheyenne Barela; Daniel Strange; Drew Kuwazaki; Edward Desmond; Jeffrey Au; Mark Nagata; Pamela O'Brien; Razvan Sultana; Remedios Gose; Samantha Cotter; Samantha Sruba
EPI_ISL_9154978	UW Virology Lab	UW Virology Lab	Alexander Greninger; Hong Xie; Isabel Arnould; Keith R Jerome; Meei-Li Huang; Nathan Breit; Patrick Mathias; Pavitra Roychoudhury; Pooneh Hajian; Ricardo Perez; Robert J. Livingston; Sean Ellis; Seffir T. Wendm; Shah Mohamed Bakhash

We gratefully acknowledge the following Authors from the Originating laboratories responsible for obtaining the specimens, as well as the Submitting laboratories where the genome data were generated and shared via GISAID, on which this research is based.

All Submitters of data may be contacted directly via www.gisaid.org

Authors are sorted alphabetically.

Accession ID	Originating Laboratory	Submitting Laboratory	Authors
EPI_ISL_9165857	Lifebrain Covid Labor GmbH	Lifebrain Covid Labor GmbH	Alexandra Wagner; Anna Edermayr; Filip Sima

We gratefully acknowledge the following Authors from the Originating laboratories responsible for obtaining the specimens, as well as the Submitting laboratories where the genome data were generated and shared via GISAID, on which this research is based.

All Submitters of data may be contacted directly via www.gisaid.org

Authors are sorted alphabetically.

Acknowledgement EPI_SET Identifier: EPI_SET_20220527dg

Accession ID	Originating Laboratory	Submitting Laboratory	Authors
EPI_ISL_9164048, EPI_ISL_9164064, EPI_ISL_9164068, EPI_ISL_9164109	Cliniques universitaires Saint-Luc	UCLouvain/IREC/MBLG-CTMA	Benoit Kabamba Mukadi; Bertrand Bearzatto; Jean-Luc Gala; Nicolas Pinte; Paul Blanpain; Simon Ophélie; Valentin Coste

We gratefully acknowledge the following Authors from the Originating laboratories responsible for obtaining the specimens, as well as the Submitting laboratories where the genome data were generated and shared via GISAID, on which this research is based.

All Submitters of data may be contacted directly via www.gisaid.org

Authors are sorted alphabetically.

Accession ID	Originating Laboratory	Submitting Laboratory	Authors
EPI_ISL_9177472, EPI_ISL_9177474, EPI_ISL_9178070, EPI_ISL_9178071	CHU UCL Namur, Avenue G. Thérasse 1, 5530 Yvoir, Belgium	Plateforme de testing Namuroise	Degosserie Jonathan; Demars Aurore; Denis Olivier; Drugmand Jonathan; Maschietto Céline; Mullier François; Nobis Chloé; Otto Gaetan

We gratefully acknowledge the following Authors from the Originating laboratories responsible for obtaining the specimens, as well as the Submitting laboratories where the genome data were generated and shared via GISAID, on which this research is based.

All Submitters of data may be contacted directly via www.gisaid.org

Authors are sorted alphabetically.

Accession ID	Originating Laboratory	Submitting Laboratory	Authors
EPI_ISL_9162360, EPI_ISL_9162374, EPI_ISL_9162378, EPI_ISL_9162422, EPI_ISL_9162426, EPI_ISL_9162434, EPI_ISL_9162436, EPI_ISL_9162442, EPI_ISL_9162455, EPI_ISL_9162467, EPI_ISL_9162471, EPI_ISL_9162503, EPI_ISL_9162511, EPI_ISL_9162535, EPI_ISL_9162538, EPI_ISL_9162546, EPI_ISL_9162550, EPI_ISL_9162602, EPI_ISL_9162607, EPI_ISL_9162608, EPI_ISL_9162612, EPI_ISL_9162621, EPI_ISL_9162645, EPI_ISL_9162653, EPI_ISL_9162654, EPI_ISL_9162665, EPI_ISL_9162675, EPI_ISL_9162679, EPI_ISL_9162687, EPI_ISL_9162699, EPI_ISL_9162710, EPI_ISL_9162712, EPI_ISL_9162731, EPI_ISL_9162763, EPI_ISL_9162768, EPI_ISL_9162770, EPI_ISL_9162775, EPI_ISL_9162785, EPI_ISL_9162804, EPI_ISL_9162806, EPI_ISL_9162819, EPI_ISL_9162829, EPI_ISL_9162857, EPI_ISL_9162869, EPI_ISL_9162897, EPI_ISL_9162920, EPI_ISL_9162936, EPI_ISL_9162944, EPI_ISL_9162989, EPI_ISL_9163016, EPI_ISL_9163030, EPI_ISL_9163042, EPI_ISL_9163044, EPI_ISL_9163056, EPI_ISL_9163062, EPI_ISL_9163076, EPI_ISL_9163096, EPI_ISL_9163116, EPI_ISL_9163180, EPI_ISL_9163188, EPI_ISL_9163189, EPI_ISL_9163227, EPI_ISL_9163258, EPI_ISL_9163268, EPI_ISL_9163290, EPI_ISL_9163301, EPI_ISL_9163317, EPI_ISL_9163322, EPI_ISL_9163343, EPI_ISL_9163347, EPI_ISL_9163383, EPI_ISL_9163388, EPI_ISL_9163396, EPI_ISL_9163401, EPI_ISL_9163445, EPI_ISL_9163448, EPI_ISL_9163450, EPI_ISL_9163453, EPI_ISL_9163461, EPI_ISL_9163486, EPI_ISL_9163492, EPI_ISL_9163493, EPI_ISL_9163495, EPI_ISL_9163508, EPI_ISL_9163536, EPI_ISL_9163580, EPI_ISL_9163587, EPI_ISL_9163600, EPI_ISL_9163606, EPI_ISL_9163616, EPI_ISL_9163620, EPI_ISL_9163622, EPI_ISL_9163666, EPI_ISL_9163690, EPI_ISL_9163692, EPI_ISL_9163710, EPI_ISL_9163748, EPI_ISL_9163758, EPI_ISL_9163760, EPI_ISL_9163773, EPI_ISL_9163788, EPI_ISL_9163789, EPI_ISL_9163794, EPI_ISL_9163807, EPI_ISL_9163817, EPI_ISL_9163834, EPI_ISL_9163841, EPI_ISL_9163853, EPI_ISL_9163867, EPI_ISL_9163896, EPI_ISL_9163902, EPI_ISL_9163908, EPI_ISL_9163922, EPI_ISL_9163928, EPI_ISL_9163932, EPI_ISL_9163938, EPI_ISL_9163982, EPI_ISL_9163986, EPI_ISL_9163992, EPI_ISL_9164002, EPI_ISL_9164004, EPI_ISL_9164021, EPI_ISL_9164147, EPI_ISL_9164152, EPI_ISL_9164169, EPI_ISL_9164171, EPI_ISL_9164182, EPI_ISL_9164206, EPI_ISL_9164212, EPI_ISL_9164227, EPI_ISL_9164244, EPI_ISL_9164268, EPI_ISL_9164277, EPI_ISL_9164287, EPI_ISL_9164298, EPI_ISL_9164311, EPI_ISL_9164313, EPI_ISL_9164322, EPI_ISL_9164345, EPI_ISL_9164369, EPI_ISL_9164383, EPI_ISL_9164388, EPI_ISL_9164414, EPI_ISL_9164442, EPI_ISL_9164445, EPI_ISL_9164458, EPI_ISL_9164464, EPI_ISL_9164466, EPI_ISL_9164472, EPI_ISL_9164475, EPI_ISL_9164479, EPI_ISL_9164496, EPI_ISL_9164529, EPI_ISL_9164537, EPI_ISL_9164566, EPI_ISL_9164599, EPI_ISL_9164606, EPI_ISL_9164613, EPI_ISL_9164618, EPI_ISL_9164624, EPI_ISL_9164645, EPI_ISL_9164647, EPI_ISL_9164661, EPI_ISL_9164682, EPI_ISL_9164691, EPI_ISL_9164693, EPI_ISL_9164697, EPI_ISL_9164703, EPI_ISL_9164717, EPI_ISL_9164734, EPI_ISL_9164738, EPI_ISL_9164764, EPI_ISL_9164770, EPI_ISL_9164806, EPI_ISL_9164832, EPI_ISL_9164851, EPI_ISL_9164874, EPI_ISL_9164891, EPI_ISL_9164905, EPI_ISL_9164986, EPI_ISL_9164993, EPI_ISL_9165014, EPI_ISL_9165016, EPI_ISL_9165038, EPI_ISL_9165041, EPI_ISL_9165058, EPI_ISL_9165064, EPI_ISL_9165071, EPI_ISL_9165094, EPI_ISL_9165096, EPI_ISL_9165101, EPI_ISL_9165124, EPI_ISL_9165135, EPI_ISL_9165140, EPI_ISL_9165149, EPI_ISL_9165159, EPI_ISL_9165197, EPI_ISL_9165199, EPI_ISL_9165214, EPI_ISL_9165219, EPI_ISL_9165223, EPI_ISL_9165240, EPI_ISL_9165242, EPI_ISL_9165243, EPI_ISL_9165255, EPI_ISL_9165259, EPI_ISL_9165262, EPI_ISL_9165264, EPI_ISL_9165265, EPI_ISL_9165291, EPI_ISL_9165292, EPI_ISL_9165294, EPI_ISL_9165312, EPI_ISL_9165314, EPI_ISL_9165332, EPI_ISL_9165348, EPI_ISL_9165394, EPI_ISL_9165402, EPI_ISL_9165420, EPI_ISL_9165464, EPI_ISL_9165499, EPI_ISL_9165507, EPI_ISL_9165518, EPI_ISL_9165524, EPI_ISL_9165528, EPI_ISL_9165531, EPI_ISL_9165543, EPI_ISL_9165547, EPI_ISL_9165568, EPI_ISL_9165590, EPI_ISL_9165616, EPI_ISL_9165620, EPI_ISL_9165622, EPI_ISL_9165624, EPI_ISL_9165642, EPI_ISL_9165644, EPI_ISL_9165649, EPI_ISL_9165659, EPI_ISL_9165675, EPI_ISL_9165683, EPI_ISL_9165687, EPI_ISL_9165700, EPI_ISL_9165714, EPI_ISL_9165715, EPI_ISL_9165720, EPI_ISL_9165741, EPI_ISL_9165743, EPI_ISL_9165745, EPI_ISL_9165764, EPI_ISL_9165778, EPI_ISL_9165780, EPI_ISL_9165785, EPI_ISL_9165806, EPI_ISL_9165813, EPI_ISL_9165908, EPI_ISL_9165919, EPI_ISL_9165929, EPI_ISL_9165952, EPI_ISL_9165987, EPI_ISL_9165996, EPI_ISL_9166009, EPI_ISL_9166025, EPI_ISL_9166028, EPI_ISL_9166030, EPI_ISL_9166033, EPI_ISL_9166034, EPI_ISL_9166049, EPI_ISL_9166055, EPI_ISL_9166113, EPI_ISL_9166122, EPI_ISL_9166146, EPI_ISL_9166150, EPI_ISL_9166158, EPI_ISL_9166164, EPI_ISL_9166214, EPI_ISL_9166275, EPI_ISL_9166280, EPI_ISL_9166289, EPI_ISL_9166312, EPI_ISL_9166329, EPI_ISL_9166338, EPI_ISL_9166396, EPI_ISL_9166439, EPI_ISL_9166445, EPI_ISL_9166450, EPI_ISL_9166477, EPI_ISL_9166538, EPI_ISL_9166571, EPI_ISL_9166695, EPI_ISL_9166714, EPI_ISL_9166721, EPI_ISL_9166785, EPI_ISL_9166788, EPI_ISL_9166792, EPI_ISL_9166794, EPI_ISL_9166796, EPI_ISL_9166819, EPI_ISL_9166829, EPI_ISL_9166844, EPI_ISL_9166856, EPI_ISL_9166871, EPI_ISL_9166877, EPI_ISL_9166879, EPI_ISL_9166881, EPI_ISL_9166922, EPI_ISL_9166930, EPI_ISL_9166932, EPI_ISL_9166940, EPI_ISL_9166956, EPI_ISL_9166962, EPI_ISL_9166972, EPI_ISL_9167001, EPI_ISL_9167006, EPI_ISL_9167010, EPI_ISL_9167015, EPI_ISL_9167029, EPI_ISL_9167036, EPI_ISL_9167050, EPI_ISL_9167054, EPI_ISL_9167084, EPI_ISL_9167087, EPI_ISL_9167097, EPI_ISL_9167100, EPI_ISL_9167125, EPI_ISL_9167131, EPI_ISL_9167147, EPI_ISL_9167151, EPI_ISL_9167154, EPI_ISL_9167156, EPI_ISL_9167220, EPI_ISL_9167244, EPI_ISL_9167255, EPI_ISL_9167257, EPI_ISL_9167271, EPI_ISL_9167275, EPI_ISL_9167279, EPI_ISL_9167283, EPI_ISL_9167287, EPI_ISL_9167311, EPI_ISL_9167315, EPI_ISL_9167332, EPI_ISL_9167347, EPI_ISL_9167350, EPI_ISL_9167362, EPI_ISL_9167373, EPI_ISL_9167377, EPI_ISL_9167387, EPI_ISL_9167391, EPI_ISL_9167399, EPI_ISL_9167416, EPI_ISL_9167421, EPI_ISL_9167469, EPI_ISL_9167471, EPI_ISL_9167488, EPI_ISL_9167496, EPI_ISL_9167553, EPI_ISL_9167576, EPI_ISL_9167582, EPI_ISL_9167594, EPI_ISL_9167617, EPI_ISL_9167658, EPI_ISL_9167691, EPI_ISL_9167695, EPI_ISL_9167712, EPI_ISL_9167721, EPI_ISL_9167727, EPI_ISL_9167734, EPI_ISL_9167764, EPI_ISL_9167778, EPI_ISL_9167782, EPI_ISL_9167837, EPI_ISL_9167839, EPI_ISL_9167847, EPI_ISL_9167851, EPI_ISL_9167868, EPI_ISL_9167870, EPI_ISL_9167883, EPI_ISL_9167899, EPI_ISL_9167905, EPI_ISL_9167911, EPI_ISL_9167924, EPI_ISL_9167944, EPI_ISL_9167946, EPI_ISL_9167976, EPI_ISL_9168014, EPI_ISL_9168016, EPI_ISL_9168022, EPI_ISL_9168040, EPI_ISL_9168044, EPI_ISL_9168086, EPI_ISL_9168131, EPI_ISL_9168155, EPI_ISL_9168157, EPI_ISL_9168166, EPI_ISL_9168167, EPI_ISL_9168170, EPI_ISL_9168194, EPI_ISL_9168213, EPI_ISL_9168215, EPI_ISL_9168219, EPI_ISL_9168242, EPI_ISL_9168248, EPI_ISL_9168252, EPI_ISL_9168256, EPI_ISL_9168260, EPI_ISL_9168269, EPI_ISL_9168271, EPI_ISL_9168286, EPI_ISL_9168290, EPI_ISL_9168304, EPI_ISL_9168333, EPI_ISL_9168339, EPI_ISL_9168347, EPI_ISL_9168348, EPI_ISL_9168362, EPI_ISL_9168366, EPI_ISL_9168374, EPI_ISL_9168388, EPI_ISL_9168400, EPI_ISL_9168407, EPI_ISL_9168450, EPI_ISL_9168462, EPI_ISL_9168469, EPI_ISL_9168474, EPI_ISL_9168482, EPI_ISL_9168484, EPI_ISL_9168488, EPI_ISL_9168491, EPI_ISL_9168498, EPI_ISL_9168509, EPI_ISL_9168511, EPI_ISL_9168515, EPI_ISL_9168518, EPI_ISL_9168528, EPI_ISL_9168543, EPI_ISL_9168558, EPI_ISL_9168561, EPI_ISL_9168565, EPI_ISL_9168566, EPI_ISL_9168567, EPI_ISL_9168573, EPI_ISL_9168575, EPI_ISL_9168579, EPI_ISL_9168585, EPI_ISL_9168589, EPI_ISL_9168593, EPI_ISL_9168595, EPI_ISL_9168599, EPI_ISL_9168601, EPI_ISL_9168623, EPI_ISL_9168628, EPI_ISL_9168636, EPI_ISL_9168638, EPI_ISL_9168672, EPI_ISL_9168680, EPI_ISL_9168682, EPI_ISL_9168695, EPI_ISL_9168699, EPI_ISL_9168752, EPI_ISL_9168764, EPI_ISL_9168781, EPI_ISL_9168785, EPI_ISL_9168814, EPI_ISL_9168815, EPI_ISL_9168823, EPI_ISL_9168896, EPI_ISL_9168901, EPI_ISL_9168963, EPI_ISL_9168978, EPI_ISL_9168989, EPI_ISL_9168990, EPI_ISL_9168992, EPI_ISL_9169000, EPI_ISL_9169004, EPI_ISL_9169010, EPI_ISL_9169024, EPI_ISL_9169025, EPI_ISL_9169030, EPI_ISL_9169040, EPI_ISL_9169058, EPI_ISL_9169069, EPI_ISL_9169070, EPI_ISL_9169071, EPI_ISL_9169072, EPI_ISL_9169075, EPI_ISL_9169086, EPI_ISL_9169095, EPI_ISL_9169096, EPI_ISL_9169097, EPI_ISL_9169099, EPI_ISL_9169100, EPI_ISL_9169106, EPI_ISL_9169107, EPI_ISL_9169111, EPI_ISL_9169119, EPI_ISL_9169122, EPI_ISL_9169125, EPI_ISL_9169131, EPI_ISL_9169138			
see above	Department of Bacteria, Parasites and Fungi, Statens Serum Institut, Copenhagen, Denmark	Statens Serum Institut Bioinformatics and Microbial Genomics	Danish Covid-19 Genome Consortium

We gratefully acknowledge the following Authors from the Originating laboratories responsible for obtaining the specimens, as well as the Submitting laboratories where the genome data were generated and shared via GISAID, on which this research is based.

All Submitters of data may be contacted directly via www.gisaid.org

Authors are sorted alphabetically.

Accession ID	Originating Laboratory	Submitting Laboratory	Authors
EPI_ISL_9169142, EPI_ISL_9169197, EPI_ISL_9169200, EPI_ISL_9169210, EPI_ISL_9169224, EPI_ISL_9169235, EPI_ISL_9169236, EPI_ISL_9169242, EPI_ISL_9169249, EPI_ISL_9169254, EPI_ISL_9169255, EPI_ISL_9169263, EPI_ISL_9169270, EPI_ISL_9169276, EPI_ISL_9169281, EPI_ISL_9169291, EPI_ISL_9169302, EPI_ISL_9169310, EPI_ISL_9169322, EPI_ISL_9169324, EPI_ISL_9169326, EPI_ISL_9169327, EPI_ISL_9169333, EPI_ISL_9169336, EPI_ISL_9169358, EPI_ISL_9169366, EPI_ISL_9169370, EPI_ISL_9169388, EPI_ISL_9169391, EPI_ISL_9169397, EPI_ISL_9169504, EPI_ISL_9169505			
see above	Department of Bacteria, Parasites and Fungi, Statens Serum Institut, Copenhagen, Denmark	Statens Serum Institut Bioinformatics and Microbial Genomics	Danish Covid-19 Genome Consortium

We gratefully acknowledge the following Authors from the Originating laboratories responsible for obtaining the specimens, as well as the Submitting laboratories where the genome data were generated and shared via GISAID, on which this research is based.

All Submitters of data may be contacted directly via www.gisaid.org

Authors are sorted alphabetically.

Acknowledgement EPI_SET Identifier: EPI_SET_20220527qy

Accession ID	Originating Laboratory	Submitting Laboratory	Authors
EPI_ISL_9162368, EPI_ISL_9162372, EPI_ISL_9162410, EPI_ISL_9162462, EPI_ISL_9162464, EPI_ISL_9162479, EPI_ISL_9162505, EPI_ISL_9162513, EPI_ISL_9162519, EPI_ISL_9162525, EPI_ISL_9162529, EPI_ISL_9162552, EPI_ISL_9162580, EPI_ISL_9162587, EPI_ISL_9162593, EPI_ISL_9162610, EPI_ISL_9162638, EPI_ISL_9162640, EPI_ISL_9162641, EPI_ISL_9162656, EPI_ISL_9162669, EPI_ISL_9162686, EPI_ISL_9162695, EPI_ISL_9162714, EPI_ISL_9162718, EPI_ISL_9162722, EPI_ISL_9162732, EPI_ISL_9162746, EPI_ISL_9162748, EPI_ISL_9162750, EPI_ISL_9162754, EPI_ISL_9162796, EPI_ISL_9162802, EPI_ISL_9162808, EPI_ISL_9162823, EPI_ISL_9162837, EPI_ISL_9162841, EPI_ISL_9162845, EPI_ISL_9162871, EPI_ISL_9162904, EPI_ISL_9162917, EPI_ISL_9162927, EPI_ISL_9162962, EPI_ISL_9162968, EPI_ISL_9162976, EPI_ISL_9163001, EPI_ISL_9163003, EPI_ISL_9163006, EPI_ISL_9163012, EPI_ISL_9163046, EPI_ISL_9163065, EPI_ISL_9163073, EPI_ISL_9163077, EPI_ISL_9163102, EPI_ISL_9163106, EPI_ISL_9163129, EPI_ISL_9163134, EPI_ISL_9163146, EPI_ISL_9163198, EPI_ISL_9163222, EPI_ISL_9163237, EPI_ISL_9163263, EPI_ISL_9163298, EPI_ISL_9163335, EPI_ISL_9163338, EPI_ISL_9163346, EPI_ISL_9163368, EPI_ISL_9163376, EPI_ISL_9163380, EPI_ISL_9163385, EPI_ISL_9163412, EPI_ISL_9163423, EPI_ISL_9163457, EPI_ISL_9163466, EPI_ISL_9163484, EPI_ISL_9163503, EPI_ISL_9163526, EPI_ISL_9163555, EPI_ISL_9163565, EPI_ISL_9163576, EPI_ISL_9163593, EPI_ISL_9163598, EPI_ISL_9163610, EPI_ISL_9163638, EPI_ISL_9163640, EPI_ISL_9163642, EPI_ISL_9163644, EPI_ISL_9163653, EPI_ISL_9163662, EPI_ISL_9163672, EPI_ISL_9163673, EPI_ISL_9163700, EPI_ISL_9163703, EPI_ISL_9163715, EPI_ISL_9163728, EPI_ISL_9163743, EPI_ISL_9163754, EPI_ISL_9163756, EPI_ISL_9163762, EPI_ISL_9163764, EPI_ISL_9163766, EPI_ISL_9163796, EPI_ISL_9163802, EPI_ISL_9163819, EPI_ISL_9163855, EPI_ISL_9163857, EPI_ISL_9163859, EPI_ISL_9163862, EPI_ISL_9163880, EPI_ISL_9163889, EPI_ISL_9163894, EPI_ISL_9163900, EPI_ISL_9163912, EPI_ISL_9163914, EPI_ISL_9163936, EPI_ISL_9163940, EPI_ISL_9163948, EPI_ISL_9163984, EPI_ISL_9164015, EPI_ISL_9164017, EPI_ISL_9164023, EPI_ISL_9164141, EPI_ISL_9164157, EPI_ISL_9164163, EPI_ISL_9164194, EPI_ISL_9164196, EPI_ISL_9164204, EPI_ISL_9164210, EPI_ISL_9164228, EPI_ISL_9164283, EPI_ISL_9164300, EPI_ISL_9164329, EPI_ISL_9164357, EPI_ISL_9164358, EPI_ISL_9164363, EPI_ISL_9164365, EPI_ISL_9164367, EPI_ISL_9164387, EPI_ISL_9164390, EPI_ISL_9164397, EPI_ISL_9164402, EPI_ISL_9164405, EPI_ISL_9164418, EPI_ISL_9164428, EPI_ISL_9164439, EPI_ISL_9164444, EPI_ISL_9164447, EPI_ISL_9164452, EPI_ISL_9164470, EPI_ISL_9164502, EPI_ISL_9164512, EPI_ISL_9164523, EPI_ISL_9164604, EPI_ISL_9164611, EPI_ISL_9164614, EPI_ISL_9164615, EPI_ISL_9164629, EPI_ISL_9164665, EPI_ISL_9164667, EPI_ISL_9164678, EPI_ISL_9164684, EPI_ISL_9164686, EPI_ISL_9164695, EPI_ISL_9164780, EPI_ISL_9164787, EPI_ISL_9164800, EPI_ISL_9164802, EPI_ISL_9164807, EPI_ISL_9164814, EPI_ISL_9164816, EPI_ISL_9164866, EPI_ISL_9164876, EPI_ISL_9164910, EPI_ISL_9164948, EPI_ISL_9164953, EPI_ISL_9164955, EPI_ISL_9164968, EPI_ISL_9165019, EPI_ISL_9165048, EPI_ISL_9165061, EPI_ISL_9165069, EPI_ISL_9165074, EPI_ISL_9165079, EPI_ISL_9165080, EPI_ISL_9165095, EPI_ISL_9165119, EPI_ISL_9165145, EPI_ISL_9165147, EPI_ISL_9165161, EPI_ISL_9165179, EPI_ISL_9165184, EPI_ISL_9165203, EPI_ISL_9165205, EPI_ISL_9165221, EPI_ISL_9165271, EPI_ISL_9165286, EPI_ISL_9165289, EPI_ISL_9165299, EPI_ISL_9165324, EPI_ISL_9165346, EPI_ISL_9165398, EPI_ISL_9165400, EPI_ISL_9165414, EPI_ISL_9165422, EPI_ISL_9165456, EPI_ISL_9165458, EPI_ISL_9165466, EPI_ISL_9165470, EPI_ISL_9165475, EPI_ISL_9165476, EPI_ISL_9165491, EPI_ISL_9165502, EPI_ISL_9165504, EPI_ISL_9165512, EPI_ISL_9165520, EPI_ISL_9165530, EPI_ISL_9165541, EPI_ISL_9165581, EPI_ISL_9165610, EPI_ISL_9165640, EPI_ISL_9165646, EPI_ISL_9165657, EPI_ISL_9165689, EPI_ISL_9165698, EPI_ISL_9165704, EPI_ISL_9165710, EPI_ISL_9165721, EPI_ISL_9165740, EPI_ISL_9165762, EPI_ISL_9165782, EPI_ISL_9165804, EPI_ISL_9165814, EPI_ISL_9165911, EPI_ISL_9165920, EPI_ISL_9165935, EPI_ISL_9165937, EPI_ISL_9165944, EPI_ISL_9165954, EPI_ISL_9165989, EPI_ISL_9165993, EPI_ISL_9165998, EPI_ISL_9166002, EPI_ISL_9166027, EPI_ISL_9166036, EPI_ISL_9166041, EPI_ISL_9166047, EPI_ISL_9166072, EPI_ISL_9166078, EPI_ISL_9166093, EPI_ISL_9166095, EPI_ISL_9166097, EPI_ISL_9166116, EPI_ISL_9166135, EPI_ISL_9166139, EPI_ISL_9166159, EPI_ISL_9166166, EPI_ISL_9166188, EPI_ISL_9166191, EPI_ISL_9166209, EPI_ISL_9166270, EPI_ISL_9166284, EPI_ISL_9166309, EPI_ISL_9166343, EPI_ISL_9166353, EPI_ISL_9166355, EPI_ISL_9166365, EPI_ISL_9166375, EPI_ISL_9166419, EPI_ISL_9166421, EPI_ISL_9166427, EPI_ISL_9166448, EPI_ISL_9166454, EPI_ISL_9166466, EPI_ISL_9166548, EPI_ISL_9166554, EPI_ISL_9166556, EPI_ISL_9166567, EPI_ISL_9166579, EPI_ISL_9166693, EPI_ISL_9166701, EPI_ISL_9166708, EPI_ISL_9166710, EPI_ISL_9166738, EPI_ISL_9166739, EPI_ISL_9166750, EPI_ISL_9166774, EPI_ISL_9166780, EPI_ISL_9166782, EPI_ISL_9166806, EPI_ISL_9166815, EPI_ISL_9166852, EPI_ISL_9166861, EPI_ISL_9166867, EPI_ISL_9166885, EPI_ISL_9166890, EPI_ISL_9166896, EPI_ISL_9166936, EPI_ISL_9166976, EPI_ISL_9166997, EPI_ISL_9167008, EPI_ISL_9167017, EPI_ISL_9167022, EPI_ISL_9167031, EPI_ISL_9167034, EPI_ISL_9167040, EPI_ISL_9167042, EPI_ISL_9167044, EPI_ISL_9167068, EPI_ISL_9167103, EPI_ISL_9167108, EPI_ISL_9167123, EPI_ISL_9167132, EPI_ISL_9167141, EPI_ISL_9167158, EPI_ISL_9167175, EPI_ISL_9167185, EPI_ISL_9167189, EPI_ISL_9167194, EPI_ISL_9167196, EPI_ISL_9167209, EPI_ISL_9167218, EPI_ISL_9167226, EPI_ISL_9167232, EPI_ISL_9167273, EPI_ISL_9167285, EPI_ISL_9167297, EPI_ISL_9167308, EPI_ISL_9167319, EPI_ISL_9167324, EPI_ISL_9167340, EPI_ISL_9167342, EPI_ISL_9167410, EPI_ISL_9167449, EPI_ISL_9167467, EPI_ISL_9167473, EPI_ISL_9167541, EPI_ISL_9167555, EPI_ISL_9167563, EPI_ISL_9167611, EPI_ISL_9167621, EPI_ISL_9167653, EPI_ISL_9167677, EPI_ISL_9167681, EPI_ISL_9167707, EPI_ISL_9167723, EPI_ISL_9167750, EPI_ISL_9167754, EPI_ISL_9167760, EPI_ISL_9167784, EPI_ISL_9167787, EPI_ISL_9167793, EPI_ISL_9167795, EPI_ISL_9167801, EPI_ISL_9167811, EPI_ISL_9167825, EPI_ISL_9167827, EPI_ISL_9167862, EPI_ISL_9167872, EPI_ISL_9167893, EPI_ISL_9167913, EPI_ISL_9167919, EPI_ISL_9167932, EPI_ISL_9167936, EPI_ISL_9167950, EPI_ISL_9167964, EPI_ISL_9167972, EPI_ISL_9168006, EPI_ISL_9168008, EPI_ISL_9168024, EPI_ISL_9168026, EPI_ISL_9168048, EPI_ISL_9168052, EPI_ISL_9168057, EPI_ISL_9168061, EPI_ISL_9168071, EPI_ISL_9168073, EPI_ISL_9168077, EPI_ISL_9168092, EPI_ISL_9168106, EPI_ISL_9168108, EPI_ISL_9168149, EPI_ISL_9168159, EPI_ISL_9168208, EPI_ISL_9168240, EPI_ISL_9168265, EPI_ISL_9168267, EPI_ISL_9168283, EPI_ISL_9168317, EPI_ISL_9168326, EPI_ISL_9168327, EPI_ISL_9168334, EPI_ISL_9168340, EPI_ISL_9168341, EPI_ISL_9168346, EPI_ISL_9168356, EPI_ISL_9168369, EPI_ISL_9168370, EPI_ISL_9168373, EPI_ISL_9168381, EPI_ISL_9168386, EPI_ISL_9168390, EPI_ISL_9168394, EPI_ISL_9168398, EPI_ISL_9168403, EPI_ISL_9168405, EPI_ISL_9168411, EPI_ISL_9168413, EPI_ISL_9168414, EPI_ISL_9168453, EPI_ISL_9168457, EPI_ISL_9168463, EPI_ISL_9168466, EPI_ISL_9168476, EPI_ISL_9168481, EPI_ISL_9168487, EPI_ISL_9168513, EPI_ISL_9168517, EPI_ISL_9168522, EPI_ISL_9168525, EPI_ISL_9168537, EPI_ISL_9168544, EPI_ISL_9168546, EPI_ISL_9168551, EPI_ISL_9168554, EPI_ISL_9168563, EPI_ISL_9168568, EPI_ISL_9168576, EPI_ISL_9168577, EPI_ISL_9168578, EPI_ISL_9168584, EPI_ISL_9168586, EPI_ISL_9168591, EPI_ISL_9168604, EPI_ISL_9168609, EPI_ISL_9168614, EPI_ISL_9168617, EPI_ISL_9168634, EPI_ISL_9168635, EPI_ISL_9168641, EPI_ISL_9168644, EPI_ISL_9168647, EPI_ISL_9168648, EPI_ISL_9168660, EPI_ISL_9168665, EPI_ISL_9168676, EPI_ISL_9168684, EPI_ISL_9168686, EPI_ISL_9168687, EPI_ISL_9168692, EPI_ISL_9168698, EPI_ISL_9168710, EPI_ISL_9168712, EPI_ISL_9168756, EPI_ISL_9168757, EPI_ISL_9168761, EPI_ISL_9168765, EPI_ISL_9168768, EPI_ISL_9168783, EPI_ISL_9168784, EPI_ISL_9168790, EPI_ISL_9168793, EPI_ISL_9168799, EPI_ISL_9168808, EPI_ISL_9168903, EPI_ISL_9168907, EPI_ISL_9168969, EPI_ISL_9168975, EPI_ISL_9168984, EPI_ISL_9168999, EPI_ISL_9169002, EPI_ISL_9169007, EPI_ISL_9169013, EPI_ISL_9169014, EPI_ISL_9169015, EPI_ISL_9169016, EPI_ISL_9169018, EPI_ISL_9169022, EPI_ISL_9169023, EPI_ISL_9169031, EPI_ISL_9169046, EPI_ISL_9169048, EPI_ISL_9169052, EPI_ISL_9169057, EPI_ISL_9169059, EPI_ISL_9169063, EPI_ISL_9169064, EPI_ISL_9169068, EPI_ISL_9169074			
see above	Department of Bacteria, Parasites and Fungi, Statens Serum Institut, Copenhagen, Denmark	Statens Serum Institut Bioinformatics and Microbial Genomics	Danish Covid-19 Genome Consortium

We gratefully acknowledge the following Authors from the Originating laboratories responsible for obtaining the specimens, as well as the Submitting laboratories where the genome data were generated and shared via GISAID, on which this research is based.

All Submitters of data may be contacted directly via www.gisaid.org

Authors are sorted alphabetically.

Accession ID	Originating Laboratory	Submitting Laboratory	Authors
EPI_ISL_9169080, EPI_ISL_9169091, EPI_ISL_9169094, EPI_ISL_9169103, EPI_ISL_9169105, EPI_ISL_9169108, EPI_ISL_9169113, EPI_ISL_9169123, EPI_ISL_9169136, EPI_ISL_9169146, EPI_ISL_9169194, EPI_ISL_9169202, EPI_ISL_9169204, EPI_ISL_9169207, EPI_ISL_9169211, EPI_ISL_9169212, EPI_ISL_9169218, EPI_ISL_9169220, EPI_ISL_9169229, EPI_ISL_9169243, EPI_ISL_9169248, EPI_ISL_9169252, EPI_ISL_9169253, EPI_ISL_9169256, EPI_ISL_9169259, EPI_ISL_9169260, EPI_ISL_9169269, EPI_ISL_9169273, EPI_ISL_9169278, EPI_ISL_9169299, EPI_ISL_9169303, EPI_ISL_9169307, EPI_ISL_9169328, EPI_ISL_9169331, EPI_ISL_9169335, EPI_ISL_9169338, EPI_ISL_9169341, EPI_ISL_9169342, EPI_ISL_9169343, EPI_ISL_9169345, EPI_ISL_9169365, EPI_ISL_9169372, EPI_ISL_9169376, EPI_ISL_9169378, EPI_ISL_9169383, EPI_ISL_9169386, EPI_ISL_9169394, EPI_ISL_9169396, EPI_ISL_9169400, EPI_ISL_9169503			
see above	Department of Bacteria, Parasites and Fungi, Statens Serum Institut, Copenhagen, Denmark	Statens Serum Institut Bioinformatics and Microbial Genomics	Danish Covid-19 Genome Consortium

We gratefully acknowledge the following Authors from the Originating laboratories responsible for obtaining the specimens, as well as the Submitting laboratories where the genome data were generated and shared via GISAID, on which this research is based.

All Submitters of data may be contacted directly via www.gisaid.org

Authors are sorted alphabetically.

Accession ID	Originating Laboratory	Submitting Laboratory	Authors
EPI_ISL_9162354, EPI_ISL_9162458, EPI_ISL_9162465, EPI_ISL_9162477, EPI_ISL_9162496, EPI_ISL_9162537, EPI_ISL_9162542, EPI_ISL_9162556, EPI_ISL_9162568, EPI_ISL_9162651, EPI_ISL_9162667, EPI_ISL_9162673, EPI_ISL_9162744, EPI_ISL_9162798, EPI_ISL_9162825, EPI_ISL_9162880, EPI_ISL_9162938, EPI_ISL_9162952, EPI_ISL_9162981, EPI_ISL_9163052, EPI_ISL_9163113, EPI_ISL_9163137, EPI_ISL_9163256, EPI_ISL_9163261, EPI_ISL_9163310, EPI_ISL_9163332, EPI_ISL_9163352, EPI_ISL_9163358, EPI_ISL_9163452, EPI_ISL_9163488, EPI_ISL_9163518, EPI_ISL_9163549, EPI_ISL_9163557, EPI_ISL_9163634, EPI_ISL_9163667, EPI_ISL_9163733, EPI_ISL_9163770, EPI_ISL_9163801, EPI_ISL_9163823, EPI_ISL_9163904, EPI_ISL_9164175, EPI_ISL_9164180, EPI_ISL_9164198, EPI_ISL_9164270, EPI_ISL_9164501, EPI_ISL_9164506, EPI_ISL_9164518, EPI_ISL_9164653, EPI_ISL_9164655, EPI_ISL_9164723, EPI_ISL_9164731, EPI_ISL_9164751, EPI_ISL_9164753, EPI_ISL_9164782, EPI_ISL_9164822, EPI_ISL_9164826, EPI_ISL_9164895, EPI_ISL_9164958, EPI_ISL_9164982, EPI_ISL_9165187, EPI_ISL_9165207, EPI_ISL_9165249, EPI_ISL_9165408, EPI_ISL_9165442, EPI_ISL_9165522, EPI_ISL_9165601, EPI_ISL_9165605, EPI_ISL_9165614, EPI_ISL_9165628, EPI_ISL_9165708, EPI_ISL_9165733, EPI_ISL_9165735, EPI_ISL_9165738, EPI_ISL_9165739, EPI_ISL_9165796, EPI_ISL_9165905, EPI_ISL_9165942, EPI_ISL_9165991, EPI_ISL_9166137, EPI_ISL_9166141, EPI_ISL_9166154, EPI_ISL_9166172, EPI_ISL_9166324, EPI_ISL_9166358, EPI_ISL_9166459, EPI_ISL_9166505, EPI_ISL_9166510, EPI_ISL_9166516, EPI_ISL_9166520, EPI_ISL_9166522, EPI_ISL_9166523, EPI_ISL_9166544, EPI_ISL_9166552, EPI_ISL_9166752, EPI_ISL_9166800, EPI_ISL_9166823, EPI_ISL_9166874, EPI_ISL_9166901, EPI_ISL_9166979, EPI_ISL_9167020, EPI_ISL_9167027, EPI_ISL_9167038, EPI_ISL_9167065, EPI_ISL_9167095, EPI_ISL_9167138, EPI_ISL_9167212, EPI_ISL_9167238, EPI_ISL_9167259, EPI_ISL_9167295, EPI_ISL_9167302, EPI_ISL_9167323, EPI_ISL_9167354, EPI_ISL_9167371, EPI_ISL_9167407, EPI_ISL_9167419, EPI_ISL_9167493, EPI_ISL_9167675, EPI_ISL_9167679, EPI_ISL_9167758, EPI_ISL_9167803, EPI_ISL_9167845, EPI_ISL_9167917, EPI_ISL_9168042, EPI_ISL_9168046, EPI_ISL_9168119, EPI_ISL_9168124, EPI_ISL_9168180, EPI_ISL_9168221, EPI_ISL_9168225, EPI_ISL_9168236, EPI_ISL_9168238, EPI_ISL_9168246, EPI_ISL_9168279, EPI_ISL_9168302, EPI_ISL_9168344, EPI_ISL_9168350, EPI_ISL_9168352, EPI_ISL_9168378, EPI_ISL_9168395, EPI_ISL_9168454, EPI_ISL_9168529, EPI_ISL_9168536, EPI_ISL_9168547, EPI_ISL_9168612, EPI_ISL_9168646, EPI_ISL_9168651, EPI_ISL_9168654, EPI_ISL_9168678, EPI_ISL_9168694, EPI_ISL_9168751, EPI_ISL_9168914, EPI_ISL_9168918, EPI_ISL_9168970, EPI_ISL_9169006, EPI_ISL_9169021, EPI_ISL_9169027, EPI_ISL_9169028, EPI_ISL_9169045, EPI_ISL_9169054, EPI_ISL_9169120, EPI_ISL_9169143, EPI_ISL_9169144, EPI_ISL_9169201, EPI_ISL_9169223, EPI_ISL_9169237, EPI_ISL_9169251, EPI_ISL_9169257, EPI_ISL_9169265, EPI_ISL_9169272, EPI_ISL_9169294, EPI_ISL_9169298, EPI_ISL_9169309, EPI_ISL_9169330, EPI_ISL_9169360, EPI_ISL_9169389, EPI_ISL_9169392			
see above	Department of Bacteria, Parasites and Fungi, Statens Serum Institut, Copenhagen, Denmark	Statens Serum Institut Bioinformatics and Microbial Genomics	Danish Covid-19 Genome Consortium

We gratefully acknowledge the following Authors from the Originating laboratories responsible for obtaining the specimens, as well as the Submitting laboratories where the genome data were generated and shared via GISAID, on which this research is based.

All Submitters of data may be contacted directly via www.gisaid.org

Authors are sorted alphabetically.

Accession ID	Originating Laboratory	Submitting Laboratory	Authors
EPI_ISL_9162416, EPI_ISL_9162430, EPI_ISL_9162489, EPI_ISL_9162521, EPI_ISL_9162544, EPI_ISL_9162596, EPI_ISL_9162705, EPI_ISL_9162709, EPI_ISL_9162742, EPI_ISL_9162839, EPI_ISL_9162851, EPI_ISL_9162863, EPI_ISL_9162867, EPI_ISL_9162923, EPI_ISL_9162960, EPI_ISL_9162964, EPI_ISL_9162983, EPI_ISL_9162985, EPI_ISL_9163050, EPI_ISL_9163081, EPI_ISL_9163173, EPI_ISL_9163243, EPI_ISL_9163313, EPI_ISL_9163435, EPI_ISL_9163459, EPI_ISL_9163542, EPI_ISL_9163582, EPI_ISL_9163614, EPI_ISL_9163629, EPI_ISL_9163648, EPI_ISL_9163650, EPI_ISL_9163670, EPI_ISL_9163705, EPI_ISL_9163717, EPI_ISL_9163719, EPI_ISL_9163752, EPI_ISL_9163777, EPI_ISL_9163783, EPI_ISL_9163827, EPI_ISL_9163878, EPI_ISL_9163950, EPI_ISL_9164006, EPI_ISL_9164254, EPI_ISL_9164285, EPI_ISL_9164301, EPI_ISL_9164305, EPI_ISL_9164392, EPI_ISL_9164398, EPI_ISL_9164434, EPI_ISL_9164637, EPI_ISL_9164659, EPI_ISL_9164706, EPI_ISL_9164709, EPI_ISL_9164715, EPI_ISL_9164719, EPI_ISL_9164737, EPI_ISL_9164745, EPI_ISL_9164786, EPI_ISL_9164789, EPI_ISL_9164864, EPI_ISL_9164901, EPI_ISL_9164922, EPI_ISL_9164963, EPI_ISL_9164991, EPI_ISL_9165001, EPI_ISL_9165009, EPI_ISL_9165129, EPI_ISL_9165130, EPI_ISL_9165166, EPI_ISL_9165193, EPI_ISL_9165201, EPI_ISL_9165236, EPI_ISL_9165267, EPI_ISL_9165278, EPI_ISL_9165303, EPI_ISL_9165308, EPI_ISL_9165406, EPI_ISL_9165430, EPI_ISL_9165432, EPI_ISL_9165434, EPI_ISL_9165446, EPI_ISL_9165473, EPI_ISL_9165562, EPI_ISL_9165572, EPI_ISL_9165681, EPI_ISL_9165694, EPI_ISL_9165723, EPI_ISL_9165853, EPI_ISL_9165903, EPI_ISL_9165913, EPI_ISL_9165917, EPI_ISL_9166007, EPI_ISL_9166010, EPI_ISL_9166143, EPI_ISL_9166178, EPI_ISL_9166231, EPI_ISL_9166282, EPI_ISL_9166370, EPI_ISL_9166389, EPI_ISL_9166495, EPI_ISL_9166535, EPI_ISL_9166616, EPI_ISL_9166746, EPI_ISL_9166803, EPI_ISL_9166808, EPI_ISL_9166846, EPI_ISL_9166863, EPI_ISL_9166869, EPI_ISL_9166883, EPI_ISL_9166887, EPI_ISL_9166902, EPI_ISL_9166963, EPI_ISL_9166989, EPI_ISL_9167048, EPI_ISL_9167086, EPI_ISL_9167090, EPI_ISL_9167152, EPI_ISL_9167205, EPI_ISL_9167214, EPI_ISL_9167281, EPI_ISL_9167396, EPI_ISL_9167461, EPI_ISL_9167504, EPI_ISL_9167567, EPI_ISL_9167578, EPI_ISL_9167629, EPI_ISL_9167641, EPI_ISL_9167647, EPI_ISL_9167663, EPI_ISL_9167725, EPI_ISL_9167736, EPI_ISL_9167774, EPI_ISL_9167829, EPI_ISL_9167833, EPI_ISL_9167942, EPI_ISL_9167958, EPI_ISL_9167968, EPI_ISL_9167993, EPI_ISL_9167997, EPI_ISL_9168035, EPI_ISL_9168038, EPI_ISL_9168055, EPI_ISL_9168081, EPI_ISL_9168200, EPI_ISL_9168365, EPI_ISL_9168478, EPI_ISL_9168538, EPI_ISL_9168611, EPI_ISL_9168655, EPI_ISL_9168658, EPI_ISL_9168664, EPI_ISL_9168666, EPI_ISL_9168673, EPI_ISL_9168685, EPI_ISL_9168704, EPI_ISL_9168748, EPI_ISL_9168753, EPI_ISL_9168758, EPI_ISL_9168792, EPI_ISL_9168797, EPI_ISL_9168805, EPI_ISL_9168813, EPI_ISL_9168894, EPI_ISL_9168902, EPI_ISL_9168915, EPI_ISL_9168973, EPI_ISL_9168977, EPI_ISL_9168993, EPI_ISL_9168995, EPI_ISL_9169008, EPI_ISL_9169041, EPI_ISL_9169042, EPI_ISL_9169090, EPI_ISL_9169117, EPI_ISL_9169118, EPI_ISL_9169137, EPI_ISL_9169147, EPI_ISL_9169208, EPI_ISL_9169215, EPI_ISL_9169250, EPI_ISL_9169261, EPI_ISL_9169267, EPI_ISL_9169293, EPI_ISL_9169325, EPI_ISL_9169329, EPI_ISL_9169347, EPI_ISL_9169355, EPI_ISL_9169380, EPI_ISL_9169382, EPI_ISL_9169387			
see above	Department of Bacteria, Parasites and Fungi, Statens Serum Institut, Copenhagen, Denmark	Statens Serum Institut Bioinformatics and Microbial Genomics	Danish Covid-19 Genome Consortium

We gratefully acknowledge the following Authors from the Originating laboratories responsible for obtaining the specimens, as well as the Submitting laboratories where the genome data were generated and shared via GISAID, on which this research is based.

All Submitters of data may be contacted directly via www.gisaid.org

Authors are sorted alphabetically.

Accession ID	Originating Laboratory	Submitting Laboratory	Authors
EPI_ISL_9162358, EPI_ISL_9162413, EPI_ISL_9162415, EPI_ISL_9162427, EPI_ISL_9162432, EPI_ISL_9162440, EPI_ISL_9162449, EPI_ISL_9162452, EPI_ISL_9162453, EPI_ISL_9162473, EPI_ISL_9162491, EPI_ISL_9162501, EPI_ISL_9162507, EPI_ISL_9162531, EPI_ISL_9162540, EPI_ISL_9162548, EPI_ISL_9162558, EPI_ISL_9162564, EPI_ISL_9162570, EPI_ISL_9162582, EPI_ISL_9162658, EPI_ISL_9162671, EPI_ISL_9162682, EPI_ISL_9162688, EPI_ISL_9162690, EPI_ISL_9162752, EPI_ISL_9162766, EPI_ISL_9162777, EPI_ISL_9162811, EPI_ISL_9162817, EPI_ISL_9162835, EPI_ISL_9162853, EPI_ISL_9162865, EPI_ISL_9162906, EPI_ISL_9162913, EPI_ISL_9162929, EPI_ISL_9162942, EPI_ISL_9162946, EPI_ISL_9162966, EPI_ISL_9162978, EPI_ISL_9163020, EPI_ISL_9163022, EPI_ISL_9163028, EPI_ISL_9163036, EPI_ISL_9163067, EPI_ISL_9163090, EPI_ISL_9163108, EPI_ISL_9163111, EPI_ISL_9163139, EPI_ISL_9163152, EPI_ISL_9163166, EPI_ISL_9163184, EPI_ISL_9163192, EPI_ISL_9163248, EPI_ISL_9163273, EPI_ISL_9163281, EPI_ISL_9163283, EPI_ISL_9163287, EPI_ISL_9163303, EPI_ISL_9163319, EPI_ISL_9163340, EPI_ISL_9163404, EPI_ISL_9163408, EPI_ISL_9163413, EPI_ISL_9163440, EPI_ISL_9163441, EPI_ISL_9163446, EPI_ISL_9163455, EPI_ISL_9163478, EPI_ISL_9163499, EPI_ISL_9163511, EPI_ISL_9163515, EPI_ISL_9163540, EPI_ISL_9163547, EPI_ISL_9163573, EPI_ISL_9163574, EPI_ISL_9163585, EPI_ISL_9163618, EPI_ISL_9163659, EPI_ISL_9163675, EPI_ISL_9163682, EPI_ISL_9163684, EPI_ISL_9163694, EPI_ISL_9163725, EPI_ISL_9163732, EPI_ISL_9163735, EPI_ISL_9163799, EPI_ISL_9163847, EPI_ISL_9163849, EPI_ISL_9163869, EPI_ISL_9163874, EPI_ISL_9163881, EPI_ISL_9163888, EPI_ISL_9163906, EPI_ISL_9163910, EPI_ISL_9163920, EPI_ISL_9163930, EPI_ISL_9163946, EPI_ISL_9163958, EPI_ISL_9163966, EPI_ISL_9164000, EPI_ISL_9164027, EPI_ISL_9164029, EPI_ISL_9164031, EPI_ISL_9164033, EPI_ISL_9164132, EPI_ISL_9164188, EPI_ISL_9164241, EPI_ISL_9164246, EPI_ISL_9164266, EPI_ISL_9164309, EPI_ISL_9164316, EPI_ISL_9164347, EPI_ISL_9164353, EPI_ISL_9164360, EPI_ISL_9164408, EPI_ISL_9164420, EPI_ISL_9164449, EPI_ISL_9164454, EPI_ISL_9164456, EPI_ISL_9164462, EPI_ISL_9164510, EPI_ISL_9164521, EPI_ISL_9164609, EPI_ISL_9164625, EPI_ISL_9164649, EPI_ISL_9164651, EPI_ISL_9164673, EPI_ISL_9164733, EPI_ISL_9164747, EPI_ISL_9164776, EPI_ISL_9164830, EPI_ISL_9164847, EPI_ISL_9164852, EPI_ISL_9164862, EPI_ISL_9164868, EPI_ISL_9164884, EPI_ISL_9164887, EPI_ISL_9164897, EPI_ISL_9164913, EPI_ISL_9164929, EPI_ISL_9164932, EPI_ISL_9164945, EPI_ISL_9164961, EPI_ISL_9164989, EPI_ISL_9164998, EPI_ISL_9165005, EPI_ISL_9165028, EPI_ISL_9165076, EPI_ISL_9165116, EPI_ISL_9165133, EPI_ISL_9165143, EPI_ISL_9165171, EPI_ISL_9165209, EPI_ISL_9165216, EPI_ISL_9165253, EPI_ISL_9165257, EPI_ISL_9165284, EPI_ISL_9165305, EPI_ISL_9165307, EPI_ISL_9165340, EPI_ISL_9165436, EPI_ISL_9165452, EPI_ISL_9165468, EPI_ISL_9165481, EPI_ISL_9165484, EPI_ISL_9165486, EPI_ISL_9165495, EPI_ISL_9165535, EPI_ISL_9165536, EPI_ISL_9165549, EPI_ISL_9165564, EPI_ISL_9165574, EPI_ISL_9165579, EPI_ISL_9165582, EPI_ISL_9165626, EPI_ISL_9165636, EPI_ISL_9165654, EPI_ISL_9165670, EPI_ISL_9165702, EPI_ISL_9165719, EPI_ISL_9165747, EPI_ISL_9165758, EPI_ISL_9165766, EPI_ISL_9165802, EPI_ISL_9165925, EPI_ISL_9165979, EPI_ISL_9166016, EPI_ISL_9166045, EPI_ISL_9166057, EPI_ISL_9166061, EPI_ISL_9166131, EPI_ISL_9166148, EPI_ISL_9166176, EPI_ISL_9166190, EPI_ISL_9166193, EPI_ISL_9166212, EPI_ISL_9166277, EPI_ISL_9166315, EPI_ISL_9166363, EPI_ISL_9166373, EPI_ISL_9166394, EPI_ISL_9166416, EPI_ISL_9166429, EPI_ISL_9166444, EPI_ISL_9166464, EPI_ISL_9166469, EPI_ISL_9166515, EPI_ISL_9166519, EPI_ISL_9166569, EPI_ISL_9166585, EPI_ISL_9166587, EPI_ISL_9166591, EPI_ISL_9166703, EPI_ISL_9166706, EPI_ISL_9166716, EPI_ISL_9166729, EPI_ISL_9166735, EPI_ISL_9166741, EPI_ISL_9166760, EPI_ISL_9166821, EPI_ISL_9166850, EPI_ISL_9166892, EPI_ISL_9166899, EPI_ISL_9166908, EPI_ISL_9166924, EPI_ISL_9166942, EPI_ISL_9166944, EPI_ISL_9166958, EPI_ISL_9166960, EPI_ISL_9166965, EPI_ISL_9166967, EPI_ISL_9166974, EPI_ISL_9166981, EPI_ISL_9166985, EPI_ISL_9166987, EPI_ISL_9166993, EPI_ISL_9166999, EPI_ISL_9167046, EPI_ISL_9167092, EPI_ISL_9167094, EPI_ISL_9167127, EPI_ISL_9167134, EPI_ISL_9167149, EPI_ISL_9167191, EPI_ISL_9167199, EPI_ISL_9167216, EPI_ISL_9167222, EPI_ISL_9167224, EPI_ISL_9167228, EPI_ISL_9167240, EPI_ISL_9167242, EPI_ISL_9167267, EPI_ISL_9167269, EPI_ISL_9167299, EPI_ISL_9167309, EPI_ISL_9167317, EPI_ISL_9167326, EPI_ISL_9167328, EPI_ISL_9167336, EPI_ISL_9167358, EPI_ISL_9167360, EPI_ISL_9167369, EPI_ISL_9167383, EPI_ISL_9167385, EPI_ISL_9167401, EPI_ISL_9167451, EPI_ISL_9167463, EPI_ISL_9167481, EPI_ISL_9167498, EPI_ISL_9167500, EPI_ISL_9167539, EPI_ISL_9167580, EPI_ISL_9167590, EPI_ISL_9167615, EPI_ISL_9167625, EPI_ISL_9167635, EPI_ISL_9167637, EPI_ISL_9167673, EPI_ISL_9167689, EPI_ISL_9167693, EPI_ISL_9167697, EPI_ISL_9167740, EPI_ISL_9167744, EPI_ISL_9167756, EPI_ISL_9167766, EPI_ISL_9167770, EPI_ISL_9167780, EPI_ISL_9167791, EPI_ISL_9167805, EPI_ISL_9167854, EPI_ISL_9167858, EPI_ISL_9167860, EPI_ISL_9167887, EPI_ISL_9167889, EPI_ISL_9167907, EPI_ISL_9167909, EPI_ISL_9167926, EPI_ISL_9167995, EPI_ISL_9168029, EPI_ISL_9168063, EPI_ISL_9168078, EPI_ISL_9168084, EPI_ISL_9168112, EPI_ISL_9168114, EPI_ISL_9168118, EPI_ISL_9168122, EPI_ISL_9168176, EPI_ISL_9168184, EPI_ISL_9168226, EPI_ISL_9168244, EPI_ISL_9168273, EPI_ISL_9168275, EPI_ISL_9168294, EPI_ISL_9168329, EPI_ISL_9168335, EPI_ISL_9168354, EPI_ISL_9168359, EPI_ISL_9168361, EPI_ISL_9168364, EPI_ISL_9168367, EPI_ISL_9168372, EPI_ISL_9168376, EPI_ISL_9168377, EPI_ISL_9168379, EPI_ISL_9168385, EPI_ISL_9168387, EPI_ISL_9168391, EPI_ISL_9168402, EPI_ISL_9168409, EPI_ISL_9168412, EPI_ISL_9168438, EPI_ISL_9168448, EPI_ISL_9168464, EPI_ISL_9168470, EPI_ISL_9168475, EPI_ISL_9168479, EPI_ISL_9168486, EPI_ISL_9168492, EPI_ISL_9168494, EPI_ISL_9168497, EPI_ISL_9168499, EPI_ISL_9168501, EPI_ISL_9168502, EPI_ISL_9168508, EPI_ISL_9168520, EPI_ISL_9168527, EPI_ISL_9168542, EPI_ISL_9168545, EPI_ISL_9168548, EPI_ISL_9168552, EPI_ISL_9168553, EPI_ISL_9168569, EPI_ISL_9168570, EPI_ISL_9168582, EPI_ISL_9168590, EPI_ISL_9168594, EPI_ISL_9168603, EPI_ISL_9168610, EPI_ISL_9168615, EPI_ISL_9168630, EPI_ISL_9168631, EPI_ISL_9168639, EPI_ISL_9168650, EPI_ISL_9168656, EPI_ISL_9168661, EPI_ISL_9168677, EPI_ISL_9168681, EPI_ISL_9168683, EPI_ISL_9168691, EPI_ISL_9168696, EPI_ISL_9168697, EPI_ISL_9168702, EPI_ISL_9168705, EPI_ISL_9168749, EPI_ISL_9168766, EPI_ISL_9168770, EPI_ISL_9168775, EPI_ISL_9168791, EPI_ISL_9168795, EPI_ISL_9168900, EPI_ISL_9168909, EPI_ISL_9168910, EPI_ISL_9168911, EPI_ISL_9168916, EPI_ISL_9168917, EPI_ISL_9168986, EPI_ISL_9168987, EPI_ISL_9168994, EPI_ISL_9168996, EPI_ISL_9169003, EPI_ISL_9169019, EPI_ISL_9169020, EPI_ISL_9169026, EPI_ISL_9169034, EPI_ISL_9169035, EPI_ISL_9169039, EPI_ISL_9169044, EPI_ISL_9169050, EPI_ISL_9169051, EPI_ISL_9169053, EPI_ISL_9169055, EPI_ISL_9169060, EPI_ISL_9169065, EPI_ISL_9169085, EPI_ISL_9169087, EPI_ISL_9169092, EPI_ISL_9169102, EPI_ISL_9169104, EPI_ISL_9169139, EPI_ISL_9169149, EPI_ISL_9169196, EPI_ISL_9169203, EPI_ISL_9169213, EPI_ISL_9169219, EPI_ISL_9169227, EPI_ISL_9169228, EPI_ISL_9169234, EPI_ISL_9169240, EPI_ISL_9169241, EPI_ISL_9169258, EPI_ISL_9169280, EPI_ISL_9169283, EPI_ISL_9169285, EPI_ISL_9169286, EPI_ISL_9169287, EPI_ISL_9169289, EPI_ISL_9169292, EPI_ISL_9169296, EPI_ISL_9169312, EPI_ISL_9169315, EPI_ISL_9169319, EPI_ISL_9169320, EPI_ISL_9169349, EPI_ISL_9169351, EPI_ISL_9169361, EPI_ISL_9169362, EPI_ISL_9169368, EPI_ISL_9169374, EPI_ISL_9169375, EPI_ISL_9169385, EPI_ISL_9169399, EPI_ISL_9169500			
see above	Department of Bacteria, Parasites and Fungi, Statens Serum Institut, Copenhagen, Denmark	Statens Serum Institut Bioinformatics and Microbial Genomics	Danish Covid-19 Genome Consortium

We gratefully acknowledge the following Authors from the Originating laboratories responsible for obtaining the specimens, as well as the Submitting laboratories where the genome data were generated and shared via GISAID, on which this research is based.

All Submitters of data may be contacted directly via www.gisaid.org

Authors are sorted alphabetically.

Acknowledgement EPI_SET Identifier: EPI_SET_20220527ts

Accession ID	Originating Laboratory	Submitting Laboratory	Authors
EPI_ISL_9186382, EPI_ISL_9186384, EPI_ISL_9186385, EPI_ISL_9186412, EPI_ISL_9186447	A. Krumbholz, Labor Dr. Krause und Kollegen MVZ GmbH, Kiel	Charité Universitätsmedizin Berlin, Institut für Virologie	Andi Krumbholz; Barbara Mühlemann; Christian Drosten; Julia Schneider; Julia Tesch; Jörn Beheim-Schwarzbach; Talitha Veith; Terry Jones; Tobias Bleicker; Victor M Corman

We gratefully acknowledge the following Authors from the Originating laboratories responsible for obtaining the specimens, as well as the Submitting laboratories where the genome data were generated and shared via GISAID, on which this research is based.

All Submitters of data may be contacted directly via www.gisaid.org

Authors are sorted alphabetically.

Accession ID	Originating Laboratory	Submitting Laboratory	Authors
EPI_ISL_9171918, EPI_ISL_9171922	Azienda Sanitaria Locale di Piacenza - Presidio Ospedaliero - Laboratorio di Microbiologia	Istituto Zooprofilattico Sperimentale della Lombardia e dell'Emilia Romagna (IZSLER), Risk Analysis and Genomic Epidemiology Unit	Erika Scaltriti; Ilaria Menozzi; Lo Cascio Giuliana; Marina Morganti; Peroni Irene; Schiavo Roberta; Stefano Pongolini

We gratefully acknowledge the following Authors from the Originating laboratories responsible for obtaining the specimens, as well as the Submitting laboratories where the genome data were generated and shared via GISAID, on which this research is based.

All Submitters of data may be contacted directly via www.gisaid.org

Authors are sorted alphabetically.

Accession ID	Originating Laboratory	Submitting Laboratory	Authors
EPI_ISL_9181711, EPI_ISL_9181713	Laboratoire national de sante, Microbiology, Virology	Laboratoire national de sante, Microbiology, Microbial Genomics Platform	Anke Wienecke-Baldacchino; Catherine Ragimbeau; Elodie Solarino; Fatu Djabi; Jessica Tapp; Lise Pignon; Raoul Salmon; Sibel Berger; Tamir Abdelrahman; Trung Nguyen Nguyen; Virginie Jover

We gratefully acknowledge the following Authors from the Originating laboratories responsible for obtaining the specimens, as well as the Submitting laboratories where the genome data were generated and shared via GISAID, on which this research is based.

All Submitters of data may be contacted directly via www.gisaid.org

Authors are sorted alphabetically.

Accession ID	Originating Laboratory	Submitting Laboratory	Authors
EPI_ISL_9154325	Medyczne Laboratorium Diagnostyczne Medrex	1. Academic Center for Pathomorphological and Genetic-Molecular Diagnostics ltd, Bialystok, Poland 2. National Institute of Public Health - National Institute of Hygiene, Warsaw, Poland	Anetta Sulewska; Jacek Nikliński; Janusz Dzięcioł; Joanna Kiśluk; Katarzyna Zacharczuk; Konrad Raczkowski; Magdalena Nowakowska; Małgorzata Sadkowska-Todys; Piotr Karabowicz; Piotr Majewski; Przemysław Biecek. Joanna Reszeć; Radosław Charkiewicz; Tomasz Wołkowicz
EPI_ISL_9154334, EPI_ISL_9154349	Szpital Wojewódzki im. Kardynała Stefana Wyszyńskiego w Łomży	1. Academic Center for Pathomorphological and Genetic-Molecular Diagnostics ltd, Bialystok, Poland 2. National Institute of Public Health - National Institute of Hygiene, Warsaw, Poland	Anetta Sulewska; Jacek Nikliński; Janusz Dzięcioł; Joanna Kiśluk; Katarzyna Zacharczuk; Konrad Raczkowski; Magdalena Nowakowska; Małgorzata Sadkowska-Todys; Piotr Karabowicz; Piotr Majewski; Przemysław Biecek. Joanna Reszeć; Radosław Charkiewicz; Tomasz Wołkowicz
EPI_ISL_9154644	Vitomed Sp. z o.o.	1. Academic Center for Pathomorphological and Genetic-Molecular Diagnostics ltd, Bialystok, Poland 2. National Institute of Public Health - National Institute of Hygiene, Warsaw, Poland	Anetta Sulewska; Jacek Nikliński; Janusz Dzięcioł; Joanna Kiśluk; Katarzyna Zacharczuk; Konrad Raczkowski; Magdalena Nowakowska; Małgorzata Sadkowska-Todys; Piotr Karabowicz; Piotr Majewski; Przemysław Biecek. Joanna Reszeć; Radosław Charkiewicz; Tomasz Wołkowicz

We gratefully acknowledge the following Authors from the Originating laboratories responsible for obtaining the specimens, as well as the Submitting laboratories where the genome data were generated and shared via GISAID, on which this research is based.

All Submitters of data may be contacted directly via www.gisaid.org

Authors are sorted alphabetically.

Accession ID	Originating Laboratory	Submitting Laboratory	Authors
EPI_ISL_9156927	Center for Laboratory Medicine	Center for Laboratory Medicine, ZLMSG	Yannick Gerth

We gratefully acknowledge the following Authors from the Originating laboratories responsible for obtaining the specimens, as well as the Submitting laboratories where the genome data were generated and shared via GISAID, on which this research is based.

All Submitters of data may be contacted directly via www.gisaid.org

Authors are sorted alphabetically.

Accession ID	Originating Laboratory	Submitting Laboratory	Authors
EPI_ISL_9163539	Originating lab: Wales Specialist Virology Centre Sequencing lab: Pathogen Genomics Unit	Public Health Wales Microbiology Cardiff Wales Specialist Virology Centre	Alec Birchley; Alexander Adams; Amy Gaskin; Angela Marchbank; Bree Gatica-Wilcox; Catherine Moore; Jason Coombes; Joanne Watkins; Joel Southgate; Johnathan Evans; Laura Gifford; Lauren Gilbert; Lee Graham; Malorie Perry; Matthew Bull; Nicole Pacchiarini; Sally Corden; Sara Kumziene-Summerhayes; Sara Rey; Sarah Taylor; Simon Cottrell; Sophie Jones; Tom Connor
EPI_ISL_9159155, EPI_ISL_9159156, EPI_ISL_9159159, EPI_ISL_9159160, EPI_ISL_9159164, EPI_ISL_9159166, EPI_ISL_9159170, EPI_ISL_9159171, EPI_ISL_9159177, EPI_ISL_9159178, EPI_ISL_9159184, EPI_ISL_9159190, EPI_ISL_9159195, EPI_ISL_9159197, EPI_ISL_9159199, EPI_ISL_9159200, EPI_ISL_9159203, EPI_ISL_9159204, EPI_ISL_9159207, EPI_ISL_9159208, EPI_ISL_9159210, EPI_ISL_9159216, EPI_ISL_9159238, EPI_ISL_9159240, EPI_ISL_9159243, EPI_ISL_9159246, EPI_ISL_9159249, EPI_ISL_9159251, EPI_ISL_9159252, EPI_ISL_9159253, EPI_ISL_9159256, EPI_ISL_9159260, EPI_ISL_9159261, EPI_ISL_9159263, EPI_ISL_9159264, EPI_ISL_9159270, EPI_ISL_9159271, EPI_ISL_9159272, EPI_ISL_9159297, EPI_ISL_9159299, EPI_ISL_9159304, EPI_ISL_9159336, EPI_ISL_9159341, EPI_ISL_9159344, EPI_ISL_9159346, EPI_ISL_9159351, EPI_ISL_9159409, EPI_ISL_9159418, EPI_ISL_9159423, EPI_ISL_9159432, EPI_ISL_9159439, EPI_ISL_9159466, EPI_ISL_9159467, EPI_ISL_9159468, EPI_ISL_9159471, EPI_ISL_9159473, EPI_ISL_9159475, EPI_ISL_9159478, EPI_ISL_9159479, EPI_ISL_9159481, EPI_ISL_9159484, EPI_ISL_9159518, EPI_ISL_9159519, EPI_ISL_9159538, EPI_ISL_9159570, EPI_ISL_9160198, EPI_ISL_9160202, EPI_ISL_9160204, EPI_ISL_9160207, EPI_ISL_9160261, EPI_ISL_9160265, EPI_ISL_9160266, EPI_ISL_9160268, EPI_ISL_9160273, EPI_ISL_9160274, EPI_ISL_9160278, EPI_ISL_9160281, EPI_ISL_9160282, EPI_ISL_9160283, EPI_ISL_9160313, EPI_ISL_9160316, EPI_ISL_9160322, EPI_ISL_9160323, EPI_ISL_9160326, EPI_ISL_9160329, EPI_ISL_9160330, EPI_ISL_9160333, EPI_ISL_9160337, EPI_ISL_9160564, EPI_ISL_9160637, EPI_ISL_9160638, EPI_ISL_9160639, EPI_ISL_9160649, EPI_ISL_9160697			
see above	Respiratory Virus Unit, Microbiology Services Colindale, Public Health England	COVID-19 Genomics UK (COG-UK) Consortium	PHE Covid Sequencing Team

We gratefully acknowledge the following Authors from the Originating laboratories responsible for obtaining the specimens, as well as the Submitting laboratories where the genome data were generated and shared via GISAID, on which this research is based.

All Submitters of data may be contacted directly via www.gisaid.org

Authors are sorted alphabetically.

Acknowledgement EPI_SET Identifier: EPI_SET_20220527my

Accession ID	Originating Laboratory	Submitting Laboratory	Authors
EPI_ISL_9201580	VERMAAK	National Institute for Communicable Diseases of the National Health Laboratory Service	Amoako DG; Bhiman JN; Everatt J; Ismail A; Kekana D; Mahlangu B; Mnguni A; Mohale T; Ntuli N; Scheepers C; Wolter N

We gratefully acknowledge the following Authors from the Originating laboratories responsible for obtaining the specimens, as well as the Submitting laboratories where the genome data were generated and shared via GISAID, on which this research is based.

All Submitters of data may be contacted directly via www.gisaid.org

Authors are sorted alphabetically.

Accession ID	Originating Laboratory	Submitting Laboratory	Authors
EPI_ISL_9232037, EPI_ISL_9232038, EPI_ISL_9232039, EPI_ISL_9232044, EPI_ISL_9232045, EPI_ISL_9232046, EPI_ISL_9232048, EPI_ISL_9232060, EPI_ISL_9232086, EPI_ISL_9232088, EPI_ISL_9232089, EPI_ISL_9232090, EPI_ISL_9232091, EPI_ISL_9232097, EPI_ISL_9232102, EPI_ISL_9232108, EPI_ISL_9232130, EPI_ISL_9232136, EPI_ISL_9232146, EPI_ISL_9232154, EPI_ISL_9232155, EPI_ISL_9232156, EPI_ISL_9232160, EPI_ISL_9232161, EPI_ISL_9232163, EPI_ISL_9232168, EPI_ISL_9232178, EPI_ISL_9232179, EPI_ISL_9232256, EPI_ISL_9232266, EPI_ISL_9232278, EPI_ISL_9232279, EPI_ISL_9232308			
see above	AP SSO	CSIR-Centre for Cellular and Molecular Biology-INSACOG	Amareshwar Vodapalli; Ara Sreenivas; Archana Bharadwaj Siva; B Himasri; Divya Tej Sowpati; Jandhyala Sai Krishna; Karthik Bharadwaj Tallapaka; Lamuk Zaveri; Malini Nemalikanti; Priya Nurkurthy; Rakesh K Mishra; Shreekant Verma; Sreelekshmi MS; Sumedha Avadhanula; Surabhi Srivastava; Tulasi Nagabandi; Valli Nagalakshmi Undamatla; Vidhyadhari Methuku
EPI_ISL_9236277	BJ Medical College, Ahmedabad	Gujarat Biotechnology Research Centre	Akhilesh Modi; Apurvasinh Puvar; Bhadreshsinh Gohil; Chaitanya Joshi; Disha Vora; Janvi Raval; Jaykumar Rangani; Madhvi Joshi; Nimesh Patel; Nitin Savaliya; NitinShukla; Priyank Chavda; Ramesh Pandit; Roshani Mishra; Sonal Sharma; Sumita Soni; Tasnim Trivedi; Zarna Patel
EPI_ISL_9232398	Continental Hospital	CSIR-Centre for Cellular and Molecular Biology	Amareshwar Vodapalli; Ara Sreenivas; Archana Bharadwaj Siva; B Himasri; Divya Tej Sowpati; Jandhyala Sai Krishna; Karthik Bharadwaj Tallapaka; Lamuk Zaveri; Malini Nemalikanti; Priya Nurkurthy; Rakesh K Mishra; Shreekant Verma; Sreelekshmi MS; Sumedha Avadhanula; Surabhi Srivastava; Tulasi Nagabandi; Valli Nagalakshmi Undamatla; Vidhyadhari Methuku
EPI_ISL_9233793	Fukushima Prefectural Institute of Public Health	Fukushima Prefectural Institute of Public Health	Kadzuhiro Kitakawa; Kazunori Suzuki; Kentaro Itokawa; Makoto Kuroda; Masanori Hashino; Naoko Kashiwabara; Rie Suzuki; Rina Tanaka; Shohei Fujita; Tsuyoshi Sekizuka
EPI_ISL_9234081, EPI_ISL_9234082	GMERS Medical College, Valsad	Gujarat Biotechnology Research Centre	Akhilesh Modi; Apurvasinh Puvar; Bhadreshsinh Gohil; Chaitanya Joshi; Disha Vora; Janvi Raval; Jaykumar Rangani; Madhvi Joshi; Nimesh Patel; Nitin Savaliya; NitinShukla; Priyank Chavda; Ramesh Pandit; Roshani Mishra; Sonal Sharma; Tasnim Trivedi; Vicky Gandhi; Zarna Patel
EPI_ISL_9236268	General Hospital, Lunavada, Mahisagar	Gujarat Biotechnology Research Centre	Akhilesh Modi; Apurvasinh Puvar; Bhadreshsinh Gohil; C.K.Solanki; Chaitanya Joshi; Disha Vora; Janvi Raval; Jaykumar Rangani; Madhvi Joshi; Nimesh Patel; Nitin Savaliya; NitinShukla; Priyank Chavda; Ramesh Pandit; Roshani Mishra; Sonal Sharma; Tasnim Trivedi; Zarna Patel
EPI_ISL_9234083	Green Cross Genetics Lab Pvt Ltd, Ahmedabad	Gujarat Biotechnology Research Centre	Akhilesh Modi; Apurvasinh Puvar; Bhadreshsinh Gohil; Chaitanya Joshi; Disha Vora; Janvi Raval; Jaykumar Rangani; Madhvi Joshi; Nimesh Patel; Nitin Savaliya; NitinShukla; Priyank Chavda; Ramesh Pandit; Roshani Mishra; Sonal Sharma; Tasnim Trivedi; Umang Patel; Zarna Patel
EPI_ISL_9236266	Manglam In Vitro Laboratory, Rajkot	Gujarat Biotechnology Research Centre	Akhilesh Modi; Apurvasinh Puvar; Bhadreshsinh Gohil; Chaitanya Joshi; Disha Vora; Janvi Raval; Jaykumar Rangani; Madhvi Joshi; Nimesh Patel; Nitin Savaliya; NitinShukla; Priyank Chavda; Rahul Thumbar; Ramesh Pandit; Roshani Mishra; Sonal Sharma; Tasnim Trivedi; Zarna Patel
EPI_ISL_9232081, EPI_ISL_9232100, EPI_ISL_9232101, EPI_ISL_9232105, EPI_ISL_9232106, EPI_ISL_9232126, EPI_ISL_9232128, EPI_ISL_9232141, EPI_ISL_9232184, EPI_ISL_9232185, EPI_ISL_9232186, EPI_ISL_9232231, EPI_ISL_9232232, EPI_ISL_9232285			
see above	MapMyGenome	CSIR-Centre for Cellular and Molecular Biology-INSACOG	Amareshwar Vodapalli; Anuradha Acharya; Ara Sreenivas; Archana Bharadwaj Siva; B Himasri; Divya Tej Sowpati; Jandhyala Sai Krishna; Karthik Bharadwaj Tallapaka; Lamuk Zaveri; M. Aravind Kumar; Malini Nemalikanti; Priya Nurkurthy; Rakesh K Mishra; S.P.S. Satish; Sandhya Kiran; Shreekant Verma; Sreelekshmi MS; Sumedha Avadhanula; Surabhi Srivastava; Tulasi Nagabandi; Valli Nagalakshmi Undamatla; Vidhyadhari Methuku
EPI_ISL_9219357	Mie Prefecture Health and Environment Research Institute	Mie Prefecture Health and Environment Research Institute	Kentaro Itokawa; Makoto Kuroda; Masanori Hashino; Rina Tanaka; Takuya Yano; Tsuyoshi Sekizuka
EPI_ISL_9234084, EPI_ISL_9234085	Molecular lab, Shree Krishna Hospital, Karamsad, Anand	Gujarat Biotechnology Research Centre	Akhilesh Modi; Apurvasinh Puvar; Bhadreshsinh Gohil; Chaitanya Joshi; Chirag; Disha Vora; Janvi Raval; Jaykumar Rangani; Madhvi Joshi; Mustafa; Nimesh Patel; Nitin Savaliya; NitinShukla; Priyank Chavda; Ramesh Pandit; Roshani Mishra; Sonal Sharma; Tasnim Trivedi; Zarna Patel
EPI_ISL_9248707	Molecular unit, Clinical Pathology Laboratory, Medical correctional Institution	Center for Medical Genomics Ramathibodi Hospital	Anek mungaomklang; Insee Sensorn; Inthuorn Ratchatasetthakul; Kanyarat Janthima; Parsakorn Tapaopong; Poramate Jiaranai; Vichapon Tiacharoen; Wasun Chantratita
EPI_ISL_9233081, EPI_ISL_9233082, EPI_ISL_9233083, EPI_ISL_9233084, EPI_ISL_9233085, EPI_ISL_9233086, EPI_ISL_9233087, EPI_ISL_9233088, EPI_ISL_9233090, EPI_ISL_9233091, EPI_ISL_9233092, EPI_ISL_9233093, EPI_ISL_9233094, EPI_ISL_9233095, EPI_ISL_9233096, EPI_ISL_9233097, EPI_ISL_9233098, EPI_ISL_9233099, EPI_ISL_9233100, EPI_ISL_9233101, EPI_ISL_9233102, EPI_ISL_9233103, EPI_ISL_9233104, EPI_ISL_9233105, EPI_ISL_9233106, EPI_ISL_9233107, EPI_ISL_9233108, EPI_ISL_9233109, EPI_ISL_9233110, EPI_ISL_9233111, EPI_ISL_9233113, EPI_ISL_9233114, EPI_ISL_9233115, EPI_ISL_9233116, EPI_ISL_9233117, EPI_ISL_9233118, EPI_ISL_9233119, EPI_ISL_9233120, EPI_ISL_9233121, EPI_ISL_9233122, EPI_ISL_9233123, EPI_ISL_9233124, EPI_ISL_9233125, EPI_ISL_9233126, EPI_ISL_9233127, EPI_ISL_9233128, EPI_ISL_9233129, EPI_ISL_9233130, EPI_ISL_9233131, EPI_ISL_9233132, EPI_ISL_9233133, EPI_ISL_9233134, EPI_ISL_9233135, EPI_ISL_9233136, EPI_ISL_9233137, EPI_ISL_9233138, EPI_ISL_9233139, EPI_ISL_9233140, EPI_ISL_9233141, EPI_ISL_9233142, EPI_ISL_9233143, EPI_ISL_9233144, EPI_ISL_9233145, EPI_ISL_9233146, EPI_ISL_9233147, EPI_ISL_9233148, EPI_ISL_9233149, EPI_ISL_9233150, EPI_ISL_9233151, EPI_ISL_9233152, EPI_ISL_9233153, EPI_ISL_9233154, EPI_ISL_9233156, EPI_ISL_9233157, EPI_ISL_9233158, EPI_ISL_9233159, EPI_ISL_9233160, EPI_ISL_9233161, EPI_ISL_9233162, EPI_ISL_9233163, EPI_ISL_9233164, EPI_ISL_9233165, EPI_ISL_9233166, EPI_ISL_9233167, EPI_ISL_9233168, EPI_ISL_9233169, EPI_ISL_9233170, EPI_ISL_9233171, EPI_ISL_9233172, EPI_ISL_9233173, EPI_ISL_9233174, EPI_ISL_9233175, EPI_ISL_9233176			
see above	National Public Health Laboratory, National Centre for Infectious Diseases	National Public Health Laboratory, National Centre for Infectious Diseases	Benny Yeo; Grace Ngan; Lin Cui; Raymond Tzer Pin Lin; Royce Ang; Samuel Loo; Yichen Ding; Zhenyang Zhou
EPI_ISL_9236270	Nidan Path Lab, Gandhinagar	Gujarat Biotechnology Research Centre	Akhilesh Modi; Apurvasinh Puvar; Bhadreshsinh Gohil; Chaitanya Joshi; Disha Vora; Himani Patel; Janvi Raval; Jaykumar Rangani; Madhvi Joshi; Nimesh Patel; Nitin Savaliya; NitinShukla; Priyank Chavda; Ramesh Pandit; Roshani Mishra; Sonal Sharma; Tasnim Trivedi; Zarna Patel
EPI_ISL_9236271	Northstar Pathology Laboratory, Ahmedabad	Gujarat Biotechnology Research Centre	Akhilesh Modi; Apurvasinh Puvar; Bhadreshsinh Gohil; Chaitanya Joshi; Disha Vora; Janvi Raval; Jaykumar Rangani; Kanupriya; Madhvi Joshi; Nimesh Patel; Nitin Savaliya; NitinShukla; Priyank Chavda; Ramesh Pandit; Roshani Mishra; Sonal Sharma; Tasnim Trivedi; Zarna Patel
EPI_ISL_9236267	Pathocare Pathology Laboratory, Vadodara	Gujarat Biotechnology Research Centre	Akhilesh Modi; Apurvasinh Puvar; Bhadreshsinh Gohil; Chaitanya Joshi; Disha Vora; Janvi Raval; Jaykumar Rangani; Madhvi Joshi; Nimesh Patel; Nitin Savaliya; NitinShukla; Priyank Chavda; Ramesh Pandit; Roshani Mishra; Sonal Sharma; Tasnim Trivedi; Viral Patel; Zarna Patel
EPI_ISL_9232416, EPI_ISL_9232419, EPI_ISL_9232422, EPI_ISL_9232426, EPI_ISL_9232428, EPI_ISL_9232429, EPI_ISL_9232435, EPI_ISL_9232437, EPI_ISL_9232447, EPI_ISL_9232448, EPI_ISL_9232449, EPI_ISL_9232454, EPI_ISL_9232455, EPI_ISL_9232457, EPI_ISL_9232459, EPI_ISL_9232464			
see above	Rainbow Hospital	CSIR-Centre for Cellular and Molecular Biology	Amareshwar Vodapalli; Ara Sreenivas; Archana Bharadwaj Siva; B Himasri; Divya Tej Sowpati; Jandhyala Sai Krishna; Kalyan Ram Uppaluri; Karthik Bharadwaj Tallapaka; Lamuk Zaveri; Malini Nemalikanti; Priya Nurkurthy; Rakesh K Mishra; Shreekant Verma; Smita Juvvadi; Sreelekshmi MS; Sricharan Devineni; Sumedha Avadhanula; Surabhi Srivastava; Tulasi Nagabandi; Valli Nagalakshmi Undamatla; Vidhyadhari Methuku
EPI_ISL_9248700, EPI_ISL_9248701	Ramathibodi Hospital	Center for Medical Genomics Ramathibodi Hospital	Anek mungaomklang; Insee Sensorn; Inthuorn Ratchatasetthakul; Kanyarat Janthima; Parsakorn Tapaopong; Poramate Jiaranai; Vichapon Tiacharoen; Wasun Chantratita
EPI_ISL_9236274	SSG Hospital Vadodara	Gujarat Biotechnology Research Centre	Akhilesh Modi; Apurvasinh Puvar; Bhadreshsinh Gohil; Chaitanya Joshi; Disha Vora; Janvi Raval; Jaykumar Rangani; Jigna Karia; Madhvi Joshi; Nimesh Patel; Nitin Savaliya; NitinShukla; Priyank Chavda; Ramesh Pandit; Roshani Mishra; Sonal Sharma; Tasnim Trivedi; Zarna Patel
EPI_ISL_9234086	Supratech Micropath Diagnostics & Research laboratory Ahmedabad	Gujarat Biotechnology Research Centre	Akhilesh Modi; Amee Shukla; Apurvasinh Puvar; Bhadreshsinh Gohil; Chaitanya Joshi; Disha Vora; Janvi Raval; Jaykumar Rangani; Madhvi Joshi; Nimesh Patel; Nitin Savaliya; NitinShukla; Priyank Chavda; Ramesh Pandit; Roshani Mishra; Sonal Sharma; Tasnim Trivedi; Zarna Patel
EPI_ISL_9236272, EPI_ISL_9236273, EPI_ISL_9236276	Symmers Pathocare Laboratory -Ahmedabad	Gujarat Biotechnology Research Centre	Akhilesh Modi; Apurvasinh Puvar; Bhadreshsinh Gohil; Chaitanya Joshi; Disha Vora; Janvi Raval; Jaykumar Rangani; Madhvi Joshi; Nimesh Patel; Nitin Savaliya; NitinShukla; Priyank Chavda; Ramesh Pandit; Roshani Mishra; Sonal Sharma; Tasnim Trivedi; Umang Gandhi; Zarna Patel
EPI_ISL_9234080	Universal Pathology & Molocular Lab, New Ranip, Ahmedabad	Gujarat Biotechnology Research Centre	Akhilesh Modi; Apurvasinh Puvar; Bhadreshsinh Gohil; Bipin Patel; Chaitanya Joshi; Disha Vora; Janvi Raval; Jaykumar Rangani; Madhvi Joshi; Nimesh Patel; Nitin Savaliya; NitinShukla; Priyank Chavda; Ramesh Pandit; Roshani Mishra; Sonal Sharma; Tasnim Trivedi; Zarna Patel
EPI_ISL_9236269	Zydus Hospital & Healthcare Research Pvt. Ltd.	Gujarat Biotechnology Research Centre	Akhilesh Modi; Apurvasinh Puvar; Bhadreshsinh Gohil; Chaitanya Joshi; Disha Vora; Janvi Raval; Jaykumar Rangani; Jaymin Patel; Madhvi Joshi; Nimesh Patel; Nitin Savaliya; NitinShukla; Priyank Chavda; Ramesh Pandit; Roshani Mishra; Sonal Sharma; Tasnim Trivedi; Zarna Patel

We gratefully acknowledge the following Authors from the Originating laboratories responsible for obtaining the specimens, as well as the Submitting laboratories where the genome data were generated and shared via GISAID, on which this research is based.

All Submitters of data may be contacted directly via www.gisaid.org

Authors are sorted alphabetically.

Accession ID	Originating Laboratory	Submitting Laboratory	Authors
EPI_ISL_9252474	CHU UCL Namur, Avenue G. Thérasse 1, 5530 Yvoir, Belgium	Plateforme de testing Namuroise	Degosserie Jonathan; Demars Aurore; Denis Olivier; Drugmand Jonathan; Maschietto Céline; Mullier François; Nobis Chloé; Otto Gaetan
EPI_ISL_9246983	Department of Clinical Microbiology	GIGA Medical Genomics	Claire Gourzonès; Cécile Meex; Keith Durkin; Laurent Gillet; Maria Artesi; Marie-Pierre Hayette; Nadine Cambisano; Nathalie Renotte; Olivier Ek; Sébastien Bontems; Vincent Bours

We gratefully acknowledge the following Authors from the Originating laboratories responsible for obtaining the specimens, as well as the Submitting laboratories where the genome data were generated and shared via GISAID, on which this research is based.

All Submitters of data may be contacted directly via www.gisaid.org

Authors are sorted alphabetically.

Accession ID	Originating Laboratory	Submitting Laboratory	Authors
EPI_ISL_9235998, EPI_ISL_9236007, EPI_ISL_9236015, EPI_ISL_9236022, EPI_ISL_9236043, EPI_ISL_9236060, EPI_ISL_9236062, EPI_ISL_9236079, EPI_ISL_9236112, EPI_ISL_9236118, EPI_ISL_9236127, EPI_ISL_9236292, EPI_ISL_9236297, EPI_ISL_9236302, EPI_ISL_9236316, EPI_ISL_9236335, EPI_ISL_9236505, EPI_ISL_9236541, EPI_ISL_9236582, EPI_ISL_9236592, EPI_ISL_9236624, EPI_ISL_9236646, EPI_ISL_9236667, EPI_ISL_9236674, EPI_ISL_9236696, EPI_ISL_9236699, EPI_ISL_9236707, EPI_ISL_9236720, EPI_ISL_9236722, EPI_ISL_9236728, EPI_ISL_9236730, EPI_ISL_9236737, EPI_ISL_9236744, EPI_ISL_9236751, EPI_ISL_9236755, EPI_ISL_9236772, EPI_ISL_9236774, EPI_ISL_9236775, EPI_ISL_9236788, EPI_ISL_9236800, EPI_ISL_9236840, EPI_ISL_9236841, EPI_ISL_9236847, EPI_ISL_9236855, EPI_ISL_9236858, EPI_ISL_9236867, EPI_ISL_9236880, EPI_ISL_9236887, EPI_ISL_9236889, EPI_ISL_9236929, EPI_ISL_9236942, EPI_ISL_9236947, EPI_ISL_9236958, EPI_ISL_9236981, EPI_ISL_9237008, EPI_ISL_9237021, EPI_ISL_9237029, EPI_ISL_9237043, EPI_ISL_9237047, EPI_ISL_9237052, EPI_ISL_9237059, EPI_ISL_9237065, EPI_ISL_9237088, EPI_ISL_9237096, EPI_ISL_9237100, EPI_ISL_9237119, EPI_ISL_9237153, EPI_ISL_9237157, EPI_ISL_9237213, EPI_ISL_9237227, EPI_ISL_9237234, EPI_ISL_9237245, EPI_ISL_9237305, EPI_ISL_9237308, EPI_ISL_9237327, EPI_ISL_9237329, EPI_ISL_9237397, EPI_ISL_9237416, EPI_ISL_9237421, EPI_ISL_9237429, EPI_ISL_9237435, EPI_ISL_9237539, EPI_ISL_9237628, EPI_ISL_9237657, EPI_ISL_9237688, EPI_ISL_9237702, EPI_ISL_9237734, EPI_ISL_9237765, EPI_ISL_9237797, EPI_ISL_9237807, EPI_ISL_9237816, EPI_ISL_9237835, EPI_ISL_9237872, EPI_ISL_9237925, EPI_ISL_9238185, EPI_ISL_9238218, EPI_ISL_9238241, EPI_ISL_9238254, EPI_ISL_9238289, EPI_ISL_9238327, EPI_ISL_9238383, EPI_ISL_9238410, EPI_ISL_9238450, EPI_ISL_9238476, EPI_ISL_9238708, EPI_ISL_9238743, EPI_ISL_9238751, EPI_ISL_9238910, EPI_ISL_9238916, EPI_ISL_9238937, EPI_ISL_9238946, EPI_ISL_9238965, EPI_ISL_9239031, EPI_ISL_9239091, EPI_ISL_9239101, EPI_ISL_9239133, EPI_ISL_9239157, EPI_ISL_9239245, EPI_ISL_9239277, EPI_ISL_9239285, EPI_ISL_9239325, EPI_ISL_9239331, EPI_ISL_9239389, EPI_ISL_9239396, EPI_ISL_9239431, EPI_ISL_9239449, EPI_ISL_9239490, EPI_ISL_9239503, EPI_ISL_9239621, EPI_ISL_9239704, EPI_ISL_9239755, EPI_ISL_9239789, EPI_ISL_9239808, EPI_ISL_9239836, EPI_ISL_9239852, EPI_ISL_9239922, EPI_ISL_9239934, EPI_ISL_9239946, EPI_ISL_9240080, EPI_ISL_9240121, EPI_ISL_9240161, EPI_ISL_9240173, EPI_ISL_9240221, EPI_ISL_9240256, EPI_ISL_9240260, EPI_ISL_9240265, EPI_ISL_9240270, EPI_ISL_9240342, EPI_ISL_9240365, EPI_ISL_9240391, EPI_ISL_9240418, EPI_ISL_9240483, EPI_ISL_9240512, EPI_ISL_9240562, EPI_ISL_9240593, EPI_ISL_9240608, EPI_ISL_9240614, EPI_ISL_9240630, EPI_ISL_9240676, EPI_ISL_9240695, EPI_ISL_9240797, EPI_ISL_9240807, EPI_ISL_9240814, EPI_ISL_9240907, EPI_ISL_9240948, EPI_ISL_9240975, EPI_ISL_9241011, EPI_ISL_9241017, EPI_ISL_9241039, EPI_ISL_9241051, EPI_ISL_9241091, EPI_ISL_9241139, EPI_ISL_9241189, EPI_ISL_9241247, EPI_ISL_9241251, EPI_ISL_9241290, EPI_ISL_9241312, EPI_ISL_9241323, EPI_ISL_9241355, EPI_ISL_9241442, EPI_ISL_9241453, EPI_ISL_9241474, EPI_ISL_9241490, EPI_ISL_9241515, EPI_ISL_9241604, EPI_ISL_9241735, EPI_ISL_9241745, EPI_ISL_9241778, EPI_ISL_9241789, EPI_ISL_9241798, EPI_ISL_9241819, EPI_ISL_9241832, EPI_ISL_9241871, EPI_ISL_9241873, EPI_ISL_9241892, EPI_ISL_9241899, EPI_ISL_9241921, EPI_ISL_9241929, EPI_ISL_9241930, EPI_ISL_9241984, EPI_ISL_9241990, EPI_ISL_9242004, EPI_ISL_9242006, EPI_ISL_9242017, EPI_ISL_9242085, EPI_ISL_9242106, EPI_ISL_9242110, EPI_ISL_9242145, EPI_ISL_9242160, EPI_ISL_9242167, EPI_ISL_9242183, EPI_ISL_9242185, EPI_ISL_9242255, EPI_ISL_9242305, EPI_ISL_9242341, EPI_ISL_9242349, EPI_ISL_9242356, EPI_ISL_9242359, EPI_ISL_9242370, EPI_ISL_9242389, EPI_ISL_9242392, EPI_ISL_9242396, EPI_ISL_9242401, EPI_ISL_9242407, EPI_ISL_9242600, EPI_ISL_9242605, EPI_ISL_9242614, EPI_ISL_9242618, EPI_ISL_9242621, EPI_ISL_9242623, EPI_ISL_9242648, EPI_ISL_9242659, EPI_ISL_9242669, EPI_ISL_9242681, EPI_ISL_9242687, EPI_ISL_9242691, EPI_ISL_9242715, EPI_ISL_9242736, EPI_ISL_9242742, EPI_ISL_9242765, EPI_ISL_9242777, EPI_ISL_9242795, EPI_ISL_9242818, EPI_ISL_9242823, EPI_ISL_9242825, EPI_ISL_9242833, EPI_ISL_9242837, EPI_ISL_9242840, EPI_ISL_9242851, EPI_ISL_9242874, EPI_ISL_9242900, EPI_ISL_9242902, EPI_ISL_9242910, EPI_ISL_9242940, EPI_ISL_9242954, EPI_ISL_9242959, EPI_ISL_9242965, EPI_ISL_9242974, EPI_ISL_9242977, EPI_ISL_9243022, EPI_ISL_9243025, EPI_ISL_9243046, EPI_ISL_9243050, EPI_ISL_9243075, EPI_ISL_9243082, EPI_ISL_9243112, EPI_ISL_9243132, EPI_ISL_9243146, EPI_ISL_9243148, EPI_ISL_9243161, EPI_ISL_9243165, EPI_ISL_9243169, EPI_ISL_9243175, EPI_ISL_9243187, EPI_ISL_9243192, EPI_ISL_9243215, EPI_ISL_9243233, EPI_ISL_9243239, EPI_ISL_9243241, EPI_ISL_9243245			
see above	Department of Bacteria, Parasites and Fungi, Statens Serum Institut, Copenhagen, Denmark	Statens Serum Institut Bioinformatics and Microbial Genomics	Danish Covid-19 Genome Consortium
EPI_ISL_9236011, EPI_ISL_9236037, EPI_ISL_9236085, EPI_ISL_9236114, EPI_ISL_9236123, EPI_ISL_9236161, EPI_ISL_9236300, EPI_ISL_9236310, EPI_ISL_9236453, EPI_ISL_9236527, EPI_ISL_9236529, EPI_ISL_9236537, EPI_ISL_9236555, EPI_ISL_9236578, EPI_ISL_9236614, EPI_ISL_9236644, EPI_ISL_9236663, EPI_ISL_9236677, EPI_ISL_9236705, EPI_ISL_9236709, EPI_ISL_9236798, EPI_ISL_9236813, EPI_ISL_9236852, EPI_ISL_9236860, EPI_ISL_9236864, EPI_ISL_9236878, EPI_ISL_9236903, EPI_ISL_9236935, EPI_ISL_9236968, EPI_ISL_9236975, EPI_ISL_9237000, EPI_ISL_9237002, EPI_ISL_9237207, EPI_ISL_9237260, EPI_ISL_9237372, EPI_ISL_9237558, EPI_ISL_9237643, EPI_ISL_9237670, EPI_ISL_9237751, EPI_ISL_9237782, EPI_ISL_9237909, EPI_ISL_9237935, EPI_ISL_9238027, EPI_ISL_9238102, EPI_ISL_9238141, EPI_ISL_9238322, EPI_ISL_9238333, EPI_ISL_9238470, EPI_ISL_9238604, EPI_ISL_9238662, EPI_ISL_9238823, EPI_ISL_9239058, EPI_ISL_9239129, EPI_ISL_9239166, EPI_ISL_9239170, EPI_ISL_9239197, EPI_ISL_9239225, EPI_ISL_9239268, EPI_ISL_9239559, EPI_ISL_9239760, EPI_ISL_9239762, EPI_ISL_9239771, EPI_ISL_9239823, EPI_ISL_9239826, EPI_ISL_9239918, EPI_ISL_9239936, EPI_ISL_9240310, EPI_ISL_9240459, EPI_ISL_9240554, EPI_ISL_9240640, EPI_ISL_9240702, EPI_ISL_9240718, EPI_ISL_9240782, EPI_ISL_9240841, EPI_ISL_9240863, EPI_ISL_9241066, EPI_ISL_9241068, EPI_ISL_9241151, EPI_ISL_9241277, EPI_ISL_9241344, EPI_ISL_9241372, EPI_ISL_9241411, EPI_ISL_9241437, EPI_ISL_9241457, EPI_ISL_9241491, EPI_ISL_9241597, EPI_ISL_9241612, EPI_ISL_9241702, EPI_ISL_9241720, EPI_ISL_9241810, EPI_ISL_9241876, EPI_ISL_9241894, EPI_ISL_9241919, EPI_ISL_9241974, EPI_ISL_9241999, EPI_ISL_9242026, EPI_ISL_9242043, EPI_ISL_9242058, EPI_ISL_9242080, EPI_ISL_9242093, EPI_ISL_9242122, EPI_ISL_9242124, EPI_ISL_9242129, EPI_ISL_9242140, EPI_ISL_9242163, EPI_ISL_9242178, EPI_ISL_9242240, EPI_ISL_9242326, EPI_ISL_9242330, EPI_ISL_9242351, EPI_ISL_9242366, EPI_ISL_9242671, EPI_ISL_9242747, EPI_ISL_9242749, EPI_ISL_9242754, EPI_ISL_9242782, EPI_ISL_9242853, EPI_ISL_9242860, EPI_ISL_9242945, EPI_ISL_9242956, EPI_ISL_9242970, EPI_ISL_9242978, EPI_ISL_9242988, EPI_ISL_9243031, EPI_ISL_9243084, EPI_ISL_9243087, EPI_ISL_9243130, EPI_ISL_9243136, EPI_ISL_9243205, EPI_ISL_9243206, EPI_ISL_9243270			
see above	Department of Clinical Microbiology and Center for Genomic Medicine, Rigshospitalet, Copenhagen, Denmark	Statens Serum Institut Bioinformatics and Microbial Genomics	Danish Covid-19 Genome Consortium

We gratefully acknowledge the following Authors from the Originating laboratories responsible for obtaining the specimens, as well as the Submitting laboratories where the genome data were generated and shared via GISAID, on which this research is based.

All Submitters of data may be contacted directly via www.gisaid.org

Authors are sorted alphabetically.

Accession ID	Originating Laboratory	Submitting Laboratory	Authors
EPI_ISL_9235984, EPI_ISL_9236000, EPI_ISL_9236002, EPI_ISL_9236017, EPI_ISL_9236028, EPI_ISL_9236033, EPI_ISL_9236047, EPI_ISL_9236049, EPI_ISL_9236103, EPI_ISL_9236110, EPI_ISL_9236152, EPI_ISL_9236286, EPI_ISL_9236306, EPI_ISL_9236314, EPI_ISL_9236508, EPI_ISL_9236544, EPI_ISL_9236552, EPI_ISL_9236564, EPI_ISL_9236567, EPI_ISL_9236590, EPI_ISL_9236618, EPI_ISL_9236626, EPI_ISL_9236628, EPI_ISL_9236633, EPI_ISL_9236636, EPI_ISL_9236638, EPI_ISL_9236653, EPI_ISL_9236686, EPI_ISL_9236731, EPI_ISL_9236781, EPI_ISL_9236784, EPI_ISL_9236790, EPI_ISL_9236820, EPI_ISL_9236835, EPI_ISL_9236856, EPI_ISL_9236873, EPI_ISL_9236949, EPI_ISL_9236961, EPI_ISL_9236966, EPI_ISL_9236969, EPI_ISL_9236971, EPI_ISL_9237015, EPI_ISL_9237019, EPI_ISL_9237034, EPI_ISL_9237085, EPI_ISL_9237092, EPI_ISL_9237108, EPI_ISL_9237111, EPI_ISL_9237130, EPI_ISL_9237159, EPI_ISL_9237171, EPI_ISL_9237187, EPI_ISL_9237204, EPI_ISL_9237221, EPI_ISL_9237273, EPI_ISL_9237547, EPI_ISL_9237585, EPI_ISL_9237604, EPI_ISL_9237824, EPI_ISL_9237876, EPI_ISL_9238046, EPI_ISL_9238082, EPI_ISL_9238088, EPI_ISL_9238093, EPI_ISL_9238118, EPI_ISL_9238126, EPI_ISL_9238164, EPI_ISL_9238265, EPI_ISL_9238309, EPI_ISL_9238358, EPI_ISL_9238557, EPI_ISL_9238641, EPI_ISL_9238661, EPI_ISL_9238672, EPI_ISL_9238719, EPI_ISL_9238757, EPI_ISL_9238790, EPI_ISL_9238818, EPI_ISL_9238855, EPI_ISL_9238943, EPI_ISL_9239231, EPI_ISL_9239235, EPI_ISL_9239320, EPI_ISL_9239441, EPI_ISL_9239476, EPI_ISL_9239496, EPI_ISL_9239545, EPI_ISL_9239606, EPI_ISL_9239627, EPI_ISL_9239730, EPI_ISL_9239884, EPI_ISL_9239893, EPI_ISL_9239950, EPI_ISL_9239955, EPI_ISL_9239957, EPI_ISL_9239973, EPI_ISL_9240067, EPI_ISL_9240133, EPI_ISL_9240170, EPI_ISL_9240235, EPI_ISL_9240240, EPI_ISL_9240244, EPI_ISL_9240278, EPI_ISL_9240287, EPI_ISL_9240344, EPI_ISL_9240350, EPI_ISL_9240357, EPI_ISL_9240368, EPI_ISL_9240408, EPI_ISL_9240487, EPI_ISL_9240530, EPI_ISL_9240588, EPI_ISL_9240619, EPI_ISL_9240665, EPI_ISL_9240932, EPI_ISL_9240999, EPI_ISL_9241080, EPI_ISL_9241205, EPI_ISL_9241213, EPI_ISL_9241341, EPI_ISL_9241416, EPI_ISL_9241601, EPI_ISL_9241705, EPI_ISL_9241707, EPI_ISL_9241729, EPI_ISL_9241773, EPI_ISL_9241881, EPI_ISL_9241916, EPI_ISL_9241986, EPI_ISL_9242046, EPI_ISL_9242055, EPI_ISL_9242060, EPI_ISL_9242068, EPI_ISL_9242088, EPI_ISL_9242111, EPI_ISL_9242118, EPI_ISL_9242132, EPI_ISL_9242199, EPI_ISL_9242209, EPI_ISL_9242230, EPI_ISL_9242322, EPI_ISL_9242337, EPI_ISL_9242346, EPI_ISL_9242602, EPI_ISL_9242617, EPI_ISL_9242636, EPI_ISL_9242657, EPI_ISL_9242678, EPI_ISL_9242693, EPI_ISL_9242702, EPI_ISL_9242728, EPI_ISL_9242731, EPI_ISL_9242788, EPI_ISL_9242791, EPI_ISL_9242845, EPI_ISL_9242911, EPI_ISL_9242915, EPI_ISL_9242918, EPI_ISL_9242923, EPI_ISL_9242929, EPI_ISL_9242972, EPI_ISL_9242985, EPI_ISL_9243006, EPI_ISL_9243010, EPI_ISL_9243029, EPI_ISL_9243062, EPI_ISL_9243079, EPI_ISL_9243094, EPI_ISL_9243107, EPI_ISL_9243120, EPI_ISL_9243126, EPI_ISL_9243128, EPI_ISL_9243138, EPI_ISL_9243159, EPI_ISL_9243163, EPI_ISL_9243172, EPI_ISL_9243184, EPI_ISL_9243190, EPI_ISL_9243230, EPI_ISL_9243235			
see above	Department of Bacteria, Parasites and Fungi, Statens Serum Institut, Copenhagen, Denmark	Statens Serum Institut Bioinformatics and Microbial Genomics	Danish Covid-19 Genome Consortium

We gratefully acknowledge the following Authors from the Originating laboratories responsible for obtaining the specimens, as well as the Submitting laboratories where the genome data were generated and shared via GISAID, on which this research is based.

All Submitters of data may be contacted directly via www.gisaid.org

Authors are sorted alphabetically.

Accession ID	Originating Laboratory	Submitting Laboratory	Authors
EPI_ISL_9236051, EPI_ISL_9236141, EPI_ISL_9236285, EPI_ISL_9236317, EPI_ISL_9236616, EPI_ISL_9236726, EPI_ISL_9236747, EPI_ISL_9236771, EPI_ISL_9236808, EPI_ISL_9236833, EPI_ISL_9236888, EPI_ISL_9236897, EPI_ISL_9236912, EPI_ISL_9236979, EPI_ISL_9236989, EPI_ISL_9237005, EPI_ISL_9237082, EPI_ISL_9237125, EPI_ISL_9237216, EPI_ISL_9237268, EPI_ISL_9237335, EPI_ISL_9237674, EPI_ISL_9237810, EPI_ISL_9237820, EPI_ISL_9237914, EPI_ISL_9237938, EPI_ISL_9237971, EPI_ISL_9238069, EPI_ISL_9238260, EPI_ISL_9238724, EPI_ISL_9238951, EPI_ISL_9238971, EPI_ISL_9239219, EPI_ISL_9239272, EPI_ISL_9239380, EPI_ISL_9239437, EPI_ISL_9239487, EPI_ISL_9239511, EPI_ISL_9239554, EPI_ISL_9239593, EPI_ISL_9239650, EPI_ISL_9240148, EPI_ISL_9240186, EPI_ISL_9241109, EPI_ISL_9241261, EPI_ISL_9241318, EPI_ISL_9241507, EPI_ISL_9241713, EPI_ISL_9241787, EPI_ISL_9241853, EPI_ISL_9241972, EPI_ISL_9242083, EPI_ISL_9242503, EPI_ISL_9242639, EPI_ISL_9242641, EPI_ISL_9242674, EPI_ISL_9242849, EPI_ISL_9242896, EPI_ISL_9242920, EPI_ISL_9243048, EPI_ISL_9243064, EPI_ISL_9243262			
see above	Department of Bacteria, Parasites and Fungi, Statens Serum Institut, Copenhagen, Denmark	Statens Serum Institut Bioinformatics and Microbial Genomics	Danish Covid-19 Genome Consortium

We gratefully acknowledge the following Authors from the Originating laboratories responsible for obtaining the specimens, as well as the Submitting laboratories where the genome data were generated and shared via GISAID, on which this research is based.

All Submitters of data may be contacted directly via www.gisaid.org

Authors are sorted alphabetically.

Accession ID	Originating Laboratory	Submitting Laboratory	Authors
EPI_ISL_9235987, EPI_ISL_9236053, EPI_ISL_9236107, EPI_ISL_9236136, EPI_ISL_9236284, EPI_ISL_9236296, EPI_ISL_9236561, EPI_ISL_9236640, EPI_ISL_9236671, EPI_ISL_9236684, EPI_ISL_9236694, EPI_ISL_9236735, EPI_ISL_9236739, EPI_ISL_9236760, EPI_ISL_9236764, EPI_ISL_9236794, EPI_ISL_9236802, EPI_ISL_9236883, EPI_ISL_9236923, EPI_ISL_9236973, EPI_ISL_9236983, EPI_ISL_9236991, EPI_ISL_9237012, EPI_ISL_9237017, EPI_ISL_9237056, EPI_ISL_9237074, EPI_ISL_9237250, EPI_ISL_9237263, EPI_ISL_9237286, EPI_ISL_9237300, EPI_ISL_9237348, EPI_ISL_9237443, EPI_ISL_9237475, EPI_ISL_9237543, EPI_ISL_9237553, EPI_ISL_9237593, EPI_ISL_9237693, EPI_ISL_9237722, EPI_ISL_9237918, EPI_ISL_9238098, EPI_ISL_9238190, EPI_ISL_9238273, EPI_ISL_9238338, EPI_ISL_9238353, EPI_ISL_9238424, EPI_ISL_9238462, EPI_ISL_9238531, EPI_ISL_9238534, EPI_ISL_9238955, EPI_ISL_9239062, EPI_ISL_9239072, EPI_ISL_9239086, EPI_ISL_9239115, EPI_ISL_9239143, EPI_ISL_9239454, EPI_ISL_9239474, EPI_ISL_9239500, EPI_ISL_9239538, EPI_ISL_9239598, EPI_ISL_9239741, EPI_ISL_9239779, EPI_ISL_9239909, EPI_ISL_9240031, EPI_ISL_9240058, EPI_ISL_9240062, EPI_ISL_9240088, EPI_ISL_9240112, EPI_ISL_9240212, EPI_ISL_9240248, EPI_ISL_9240328, EPI_ISL_9240336, EPI_ISL_9240432, EPI_ISL_9240446, EPI_ISL_9240738, EPI_ISL_9240768, EPI_ISL_9240774, EPI_ISL_9240880, EPI_ISL_9241103, EPI_ISL_9241179, EPI_ISL_9241224, EPI_ISL_9241265, EPI_ISL_9241351, EPI_ISL_9241676, EPI_ISL_9241757, EPI_ISL_9241801, EPI_ISL_9241826, EPI_ISL_9241960, EPI_ISL_9241976, EPI_ISL_9241996, EPI_ISL_9242015, EPI_ISL_9242121, EPI_ISL_9242125, EPI_ISL_9242214, EPI_ISL_9242226, EPI_ISL_9242312, EPI_ISL_9242319, EPI_ISL_9242323, EPI_ISL_9242334, EPI_ISL_9242344, EPI_ISL_9242402, EPI_ISL_9242695, EPI_ISL_9242717, EPI_ISL_9242756, EPI_ISL_9242768, EPI_ISL_9242787, EPI_ISL_9242814, EPI_ISL_9242828, EPI_ISL_9242830, EPI_ISL_9242883, EPI_ISL_9242894, EPI_ISL_9242936, EPI_ISL_9242948, EPI_ISL_9242950, EPI_ISL_9242992, EPI_ISL_9243032, EPI_ISL_9243072, EPI_ISL_9243092, EPI_ISL_9243140, EPI_ISL_9243142, EPI_ISL_9243171, EPI_ISL_9243183, EPI_ISL_9243194, EPI_ISL_9243210, EPI_ISL_9243219, EPI_ISL_9243268, EPI_ISL_9243272, EPI_ISL_9243277			
see above	Department of Bacteria, Parasites and Fungi, Statens Serum Institut, Copenhagen, Denmark	Statens Serum Institut Bioinformatics and Microbial Genomics	Danish Covid-19 Genome Consortium
EPI_ISL_9237756, EPI_ISL_9241431, EPI_ISL_9241687	Department of Clinical Microbiology and Center for Genomic Medicine, Rigshospitalet, Copenhagen, Denmark	Statens Serum Institut Bioinformatics and Microbial Genomics	Danish Covid-19 Genome Consortium

We gratefully acknowledge the following Authors from the Originating laboratories responsible for obtaining the specimens, as well as the Submitting laboratories where the genome data were generated and shared via GISAID, on which this research is based.

All Submitters of data may be contacted directly via www.gisaid.org

Authors are sorted alphabetically.

Acknowledgement EPI_SET Identifier: EPI_SET_20220527tw

Accession ID	Originating Laboratory	Submitting Laboratory	Authors
EPI_ISL_9235982, EPI_ISL_9236041, EPI_ISL_9236077, EPI_ISL_9236099, EPI_ISL_9236105, EPI_ISL_9236122, EPI_ISL_9236140, EPI_ISL_9236147, EPI_ISL_9236160, EPI_ISL_9236282, EPI_ISL_9236288, EPI_ISL_9236313, EPI_ISL_9236512, EPI_ISL_9236531, EPI_ISL_9236539, EPI_ISL_9236548, EPI_ISL_9236550, EPI_ISL_9236570, EPI_ISL_9236599, EPI_ISL_9236601, EPI_ISL_9236676, EPI_ISL_9236681, EPI_ISL_9236697, EPI_ISL_9236724, EPI_ISL_9236786, EPI_ISL_9236796, EPI_ISL_9236804, EPI_ISL_9236872, EPI_ISL_9236895, EPI_ISL_9236996, EPI_ISL_9237040, EPI_ISL_9237041, EPI_ISL_9237054, EPI_ISL_9237098, EPI_ISL_9237183, EPI_ISL_9237229, EPI_ISL_9237313, EPI_ISL_9237340, EPI_ISL_9237439, EPI_ISL_9237513, EPI_ISL_9237608, EPI_ISL_9237613, EPI_ISL_9237903, EPI_ISL_9237931, EPI_ISL_9237966, EPI_ISL_9238041, EPI_ISL_9238054, EPI_ISL_9238079, EPI_ISL_9238136, EPI_ISL_9238181, EPI_ISL_9238203, EPI_ISL_9238554, EPI_ISL_9238566, EPI_ISL_9238650, EPI_ISL_9238666, EPI_ISL_9238703, EPI_ISL_9238869, EPI_ISL_9238977, EPI_ISL_9238979, EPI_ISL_9239034, EPI_ISL_9239119, EPI_ISL_9239124, EPI_ISL_9239176, EPI_ISL_9239305, EPI_ISL_9239375, EPI_ISL_9239602, EPI_ISL_9239631, EPI_ISL_9239722, EPI_ISL_9239775, EPI_ISL_9239798, EPI_ISL_9239882, EPI_ISL_9239901, EPI_ISL_9239941, EPI_ISL_9239983, EPI_ISL_9239988, EPI_ISL_9240033, EPI_ISL_9240086, EPI_ISL_9240119, EPI_ISL_9240154, EPI_ISL_9240166, EPI_ISL_9240169, EPI_ISL_9240355, EPI_ISL_9240399, EPI_ISL_9240402, EPI_ISL_9240501, EPI_ISL_9240558, EPI_ISL_9240578, EPI_ISL_9240604, EPI_ISL_9240688, EPI_ISL_9240711, EPI_ISL_9240818, EPI_ISL_9240923, EPI_ISL_9241030, EPI_ISL_9241044, EPI_ISL_9241054, EPI_ISL_9241074, EPI_ISL_9241129, EPI_ISL_9241136, EPI_ISL_9241201, EPI_ISL_9241284, EPI_ISL_9241294, EPI_ISL_9241332, EPI_ISL_9241336, EPI_ISL_9241478, EPI_ISL_9241539, EPI_ISL_9241545, EPI_ISL_9241743, EPI_ISL_9241774, EPI_ISL_9241780, EPI_ISL_9241792, EPI_ISL_9241818, EPI_ISL_9241865, EPI_ISL_9241885, EPI_ISL_9241901, EPI_ISL_9241939, EPI_ISL_9241942, EPI_ISL_9242065, EPI_ISL_9242077, EPI_ISL_9242114, EPI_ISL_9242143, EPI_ISL_9242150, EPI_ISL_9242152, EPI_ISL_9242166, EPI_ISL_9242172, EPI_ISL_9242176, EPI_ISL_9242180, EPI_ISL_9242187, EPI_ISL_9242212, EPI_ISL_9242221, EPI_ISL_9242223, EPI_ISL_9242228, EPI_ISL_9242253, EPI_ISL_9242607, EPI_ISL_9242625, EPI_ISL_9242630, EPI_ISL_9242664, EPI_ISL_9242666, EPI_ISL_9242763, EPI_ISL_9242793, EPI_ISL_9242843, EPI_ISL_9242871, EPI_ISL_9242881, EPI_ISL_9242884, EPI_ISL_9242891, EPI_ISL_9242963, EPI_ISL_9243004, EPI_ISL_9243066, EPI_ISL_9243105, EPI_ISL_9243144, EPI_ISL_9243191, EPI_ISL_9243203, EPI_ISL_9243223, EPI_ISL_9243243, EPI_ISL_9243248, EPI_ISL_9243279			
see above	Department of Bacteria, Parasites and Fungi, Statens Serum Institut, Copenhagen, Denmark	Statens Serum Institut Bioinformatics and Microbial Genomics	Danish Covid-19 Genome Consortium

We gratefully acknowledge the following Authors from the Originating laboratories responsible for obtaining the specimens, as well as the Submitting laboratories where the genome data were generated and shared via GISAID, on which this research is based.

All Submitters of data may be contacted directly via www.gisaid.org

Authors are sorted alphabetically.

Accession ID	Originating Laboratory	Submitting Laboratory	Authors
EPI_ISL_9256852	Accolab Sud Ouest	CERBA HealthCare	Bénédicte Roquebert; Laura Verdurme; Mathilde Roussel; Sabine Trombert; Stéphanie Haim-Boukobza
EPI_ISL_9257606	BIO DOMES UNILABS SITE DANZIGER	CERBA HealthCare	Bénédicte Roquebert; Laura Verdurme; Mathilde Roussel; Sabine Trombert; Stéphanie Haim-Boukobza
EPI_ISL_9256823, EPI_ISL_9256824	Bio Domes Unilabs Site Danziger	CERBA HealthCare	Bénédicte Roquebert; Laura Verdurme; Mathilde Roussel; Sabine Trombert; Stéphanie Haim-Boukobza
EPI_ISL_9256851	Bio Lbs - Lbm De Lillebonne	CERBA HealthCare	Bénédicte Roquebert; Laura Verdurme; Mathilde Roussel; Sabine Trombert; Stéphanie Haim-Boukobza
EPI_ISL_9256822	Biolab Unilabs Site Boucicaut	CERBA HealthCare	Bénédicte Roquebert; Laura Verdurme; Mathilde Roussel; Sabine Trombert; Stéphanie Haim-Boukobza
EPI_ISL_9257607	CERBA	CERBA HealthCare	Bénédicte Roquebert; Laura Verdurme; Mathilde Roussel; Sabine Trombert; Stéphanie Haim-Boukobza
EPI_ISL_9257727	CERBALLIANCE ALPES DURANCE	CERBA HealthCare	Bénédicte Roquebert; Laura Verdurme; Mathilde Roussel; Sabine Trombert; Stéphanie Haim-Boukobza
EPI_ISL_9257730	CERBALLIANCE ALPES DURANCE SITE TOKORO	CERBA HealthCare	Bénédicte Roquebert; Laura Verdurme; Mathilde Roussel; Sabine Trombert; Stéphanie Haim-Boukobza
EPI_ISL_9257722, EPI_ISL_9257723, EPI_ISL_9257725	CERBALLIANCE ARTOIS	CERBA HealthCare	Bénédicte Roquebert; Laura Verdurme; Mathilde Roussel; Sabine Trombert; Stéphanie Haim-Boukobza
EPI_ISL_9257726	CERBALLIANCE COTE D'AZUR	CERBA HealthCare	Bénédicte Roquebert; Laura Verdurme; Mathilde Roussel; Sabine Trombert; Stéphanie Haim-Boukobza
EPI_ISL_9257728	CERBALLIANCE COTE D'AZUR SITE ST LAURENT	CERBA HealthCare	Bénédicte Roquebert; Laura Verdurme; Mathilde Roussel; Sabine Trombert; Stéphanie Haim-Boukobza
EPI_ISL_9257729	CERBALLIANCE COTE D'AZUR SITE STRASBOURG	CERBA HealthCare	Bénédicte Roquebert; Laura Verdurme; Mathilde Roussel; Sabine Trombert; Stéphanie Haim-Boukobza
EPI_ISL_9257847	CERBALLIANCE IDF OUEST SITE FOCH	CERBA HealthCare	Bénédicte Roquebert; Laura Verdurme; Mathilde Roussel; Sabine Trombert; Stéphanie Haim-Boukobza
EPI_ISL_9257846	CERBALLIANCE IDF OUEST SITE LES CLAYES	CERBA HealthCare	Bénédicte Roquebert; Laura Verdurme; Mathilde Roussel; Sabine Trombert; Stéphanie Haim-Boukobza
EPI_ISL_9257605, EPI_ISL_9257608, EPI_ISL_9257609, EPI_ISL_9257610, EPI_ISL_9257611, EPI_ISL_9257612, EPI_ISL_9257613, EPI_ISL_9257614, EPI_ISL_9257615, EPI_ISL_9257616, EPI_ISL_9257617, EPI_ISL_9257618, EPI_ISL_9257619, EPI_ISL_9257731, EPI_ISL_9257732, EPI_ISL_9257733, EPI_ISL_9257734, EPI_ISL_9257735, EPI_ISL_9257736, EPI_ISL_9257737, EPI_ISL_9257738, EPI_ISL_9257739, EPI_ISL_9257740, EPI_ISL_9257741, EPI_ISL_9257742, EPI_ISL_9257743, EPI_ISL_9257744, EPI_ISL_9257745, EPI_ISL_9257848, EPI_ISL_9257849, EPI_ISL_9257850, EPI_ISL_9257851, EPI_ISL_9257856			
see above	CERBALLIANCE PARIS ET IDF EST	CERBA HealthCare	Bénédicte Roquebert; Laura Verdurme; Mathilde Roussel; Sabine Trombert; Stéphanie Haim-Boukobza
EPI_ISL_9257852, EPI_ISL_9257853, EPI_ISL_9257855	CERBALLIANCE PARIS IDF EST SITE PYRENEES	CERBA HealthCare	Bénédicte Roquebert; Laura Verdurme; Mathilde Roussel; Sabine Trombert; Stéphanie Haim-Boukobza
EPI_ISL_9257854	CERBALLIANCE PARIS IDF EST TROCADER0	CERBA HealthCare	Bénédicte Roquebert; Laura Verdurme; Mathilde Roussel; Sabine Trombert; Stéphanie Haim-Boukobza
EPI_ISL_9257604, EPI_ISL_9257620, EPI_ISL_9257621, EPI_ISL_9257622, EPI_ISL_9257623, EPI_ISL_9257624, EPI_ISL_9257625, EPI_ISL_9257626, EPI_ISL_9257627, EPI_ISL_9257628, EPI_ISL_9257629, EPI_ISL_9257630, EPI_ISL_9257631, EPI_ISL_9257632, EPI_ISL_9257633, EPI_ISL_9257634, EPI_ISL_9257635, EPI_ISL_9257636, EPI_ISL_9257640, EPI_ISL_9257641			
see above	CERBALLIANCE RHONE ALPES	CERBA HealthCare	Bénédicte Roquebert; Laura Verdurme; Mathilde Roussel; Sabine Trombert; Stéphanie Haim-Boukobza
EPI_ISL_9257721, EPI_ISL_9257724	CERBALLIANCE SOMME	CERBA HealthCare	Bénédicte Roquebert; Laura Verdurme; Mathilde Roussel; Sabine Trombert; Stéphanie Haim-Boukobza
EPI_ISL_9246081	CHRU BREST-Site Carhaix	laboratoire de Virologie CHRU Brest	Adissa Tran Minoui; Christopher Payan; Léa Pilorgé; Pauline Bargain; Simon Rambaut; Sophie Vallet
EPI_ISL_9246091	CHU BREST	laboratoire de Virologie CHRU Brest	Adissa Tran Minoui; Christopher Payan; Léa Pilorgé; Pauline Bargain; Simon Rambaut; Sophie Vallet
EPI_ISL_9257018	Centre Municipal De Sante	CERBA HealthCare	Bénédicte Roquebert; Laura Verdurme; Sabine Trombert; Stéphanie Haim-Boukobza
EPI_ISL_9256853	Cerballiance Cote D'azur	CERBA HealthCare	Bénédicte Roquebert; Laura Verdurme; Mathilde Roussel; Sabine Trombert; Stéphanie Haim-Boukobza
EPI_ISL_9256835	Cerballiance Idf Ouest Site Les Clayes	CERBA HealthCare	Bénédicte Roquebert; Laura Verdurme; Mathilde Roussel; Sabine Trombert; Stéphanie Haim-Boukobza
EPI_ISL_9256825, EPI_ISL_9256826, EPI_ISL_9256827, EPI_ISL_9256828, EPI_ISL_9256830, EPI_ISL_9256831, EPI_ISL_9256847, EPI_ISL_9256848, EPI_ISL_9256849, EPI_ISL_9256850, EPI_ISL_9256854, EPI_ISL_9257019			
see above	Cerballiance Paris Et Idf Est	CERBA HealthCare	Bénédicte Roquebert; Laura Verdurme; Mathilde Roussel; Sabine Trombert; Stéphanie Haim-Boukobza
EPI_ISL_9257637, EPI_ISL_9257638	EXALAB BISCARROSSE	CERBA HealthCare	Bénédicte Roquebert; Laura Verdurme; Mathilde Roussel; Sabine Trombert; Stéphanie Haim-Boukobza
EPI_ISL_9256832	Hopital Foch	CERBA HealthCare	Bénédicte Roquebert; Laura Verdurme; Mathilde Roussel; Sabine Trombert; Stéphanie Haim-Boukobza
EPI_ISL_9257718, EPI_ISL_9257719	LABM CANDIA MADONUCCIA	CERBA HealthCare	Bénédicte Roquebert; Laura Verdurme; Mathilde Roussel; Sabine Trombert; Stéphanie Haim-Boukobza
EPI_ISL_9257639	LABORATOIRE ACCOLAB	CERBA HealthCare	Bénédicte Roquebert; Laura Verdurme; Mathilde Roussel; Sabine Trombert; Stéphanie Haim-Boukobza
EPI_ISL_9257720	LABORATOIRE AUDOLYS - LONGUENESSE	CERBA HealthCare	Bénédicte Roquebert; Laura Verdurme; Mathilde Roussel; Sabine Trombert; Stéphanie Haim-Boukobza
EPI_ISL_9256845, EPI_ISL_9256846	Labm Bonhoure	CERBA HealthCare	Bénédicte Roquebert; Laura Verdurme; Mathilde Roussel; Sabine Trombert; Stéphanie Haim-Boukobza
EPI_ISL_9256821	Labm Eylau Unilabs Suchet	CERBA HealthCare	Bénédicte Roquebert; Laura Verdurme; Mathilde Roussel; Sabine Trombert; Stéphanie Haim-Boukobza
EPI_ISL_9256836, EPI_ISL_9256837, EPI_ISL_9256838, EPI_ISL_9256839, EPI_ISL_9256840, EPI_ISL_9256841, EPI_ISL_9256842, EPI_ISL_9256843, EPI_ISL_9256844			
see above	Labm Kuate	CERBA HealthCare	Bénédicte Roquebert; Laura Verdurme; Mathilde Roussel; Sabine Trombert; Stéphanie Haim-Boukobza
EPI_ISL_9245672, EPI_ISL_9245684	Labo Analyses Med	National Reference Center for Viruses of Respiratory Infections, Institut Pasteur, Paris	Angela Brisebarre; Camille Capel; Christophe Malabat; Corinne Maufrais; Etienne Simon-Lorière; Frédéric Lemoine; J.M CORCY; Julien Fumey; Louise Lefrançois; Marion Barbet; Maud Vanpeene; Méline Bizard; Slim El Khiari; Sylvie Behillil; Sylvie Van der Werf; Vincent Enouf
EPI_ISL_9256834	Laboratoire Clinique De Meudon La Foret	CERBA HealthCare	Bénédicte Roquebert; Laura Verdurme; Mathilde Roussel; Sabine Trombert; Stéphanie Haim-Boukobza
EPI_ISL_9256829	Laboratoire De La Cpam De Paris	CERBA HealthCare	Bénédicte Roquebert; Laura Verdurme; Mathilde Roussel; Sabine Trombert; Stéphanie Haim-Boukobza

We gratefully acknowledge the following Authors from the Originating laboratories responsible for obtaining the specimens, as well as the Submitting laboratories where the genome data were generated and shared via GISAID, on which this research is based.

All Submitters of data may be contacted directly via www.gisaid.org

Authors are sorted alphabetically.

Accession ID	Originating Laboratory	Submitting Laboratory	Authors
EPI_ISL_9220562, EPI_ISL_9220563, EPI_ISL_9220734	Bioscientia Labor Wermsdorf	Robert Koch Institute	
EPI_ISL_9210610	CENTOGENE Frankfurt Laboratory: Niederlassung Industriepark Höchst	Robert Koch Institute	
EPI_ISL_9214040, EPI_ISL_9214049	Clemenshospital - Alexianer Clemenshospital Münster	Robert Koch Institute	
EPI_ISL_9227746, EPI_ISL_9227774	Department für Labormedizin Abteilung III Bereich Molekulare Diagnostik Universitätsklinikum Halle(Saale)	Robert Koch Institute	
EPI_ISL_9230811	Helios Universitätsklinikum Wuppertal: Universität Witten/Herdecke: Institut für Medizinische Labordiagnostik	Robert Koch Institute	
EPI_ISL_9215680, EPI_ISL_9215705, EPI_ISL_9215706, EPI_ISL_9215737, EPI_ISL_9215856	IMD Labor Frankfurt	Robert Koch Institute	
EPI_ISL_9233956, EPI_ISL_9233967, EPI_ISL_9234559	Institut für Immunologie und Genetik Kaiserslautern: Medizinisches Labor Dr. med. B. Thiele	Robert Koch Institute	
EPI_ISL_9228558	Kerckhoff-Klinik GmbH	Robert Koch Institute	
EPI_ISL_9212611, EPI_ISL_9212614, EPI_ISL_9212617, EPI_ISL_9212624, EPI_ISL_9212625, EPI_ISL_9212627, EPI_ISL_9212634, EPI_ISL_9212635, EPI_ISL_9212637, EPI_ISL_9212641, EPI_ISL_9212643, EPI_ISL_9212644, EPI_ISL_9212647, EPI_ISL_9212649, EPI_ISL_9212652, EPI_ISL_9212655			
see above	Klinikum Stuttgart	Robert Koch Institute	
EPI_ISL_9211268, EPI_ISL_9216283, EPI_ISL_9216611, EPI_ISL_9216619, EPI_ISL_9216626, EPI_ISL_9216644, EPI_ISL_9216646, EPI_ISL_9216662, EPI_ISL_9221467, EPI_ISL_9221483, EPI_ISL_9223230, EPI_ISL_9223234, EPI_ISL_9228669			
see above	Labor Mönchengladbach MVZ Dr. Stein + Kollegen GbR	Robert Koch Institute	
EPI_ISL_9215843, EPI_ISL_9215852	Labormedizin Darmstadt	Robert Koch Institute	
EPI_ISL_9230344	Landesamt für Verbraucherschutz des Landes Sachsen-Anhalt	Robert Koch Institute	
EPI_ISL_9223182	Limbach - MVZ Labor Vorpommern-Rügen Stralsund	Robert Koch Institute	
EPI_ISL_9216728, EPI_ISL_9216738, EPI_ISL_9216756, EPI_ISL_9216819, EPI_ISL_9216845, EPI_ISL_9216861, EPI_ISL_9216896, EPI_ISL_9216901, EPI_ISL_9217082, EPI_ISL_9217101, EPI_ISL_9217109, EPI_ISL_9217199, EPI_ISL_9217315, EPI_ISL_9217364, EPI_ISL_9217375, EPI_ISL_9217407, EPI_ISL_9217423			
see above	Limbach - MVZ Labor Dr. Volkmann & Kollegen	Robert Koch Institute	
EPI_ISL_9216058, EPI_ISL_9216098, EPI_ISL_9216100, EPI_ISL_9216190, EPI_ISL_9216989, EPI_ISL_9221391	MDI Limbach Berlin GmbH; MVZ Labor Berlin	Robert Koch Institute	
EPI_ISL_9211745, EPI_ISL_9222028, EPI_ISL_9222032, EPI_ISL_9222037, EPI_ISL_9222085, EPI_ISL_9222110, EPI_ISL_9222169, EPI_ISL_9222251			
see above	MVZ Labor Dr. Fenner und Kollegen (Standort Hamburg)	Robert Koch Institute	
EPI_ISL_9217018, EPI_ISL_9221237, EPI_ISL_9221264, EPI_ISL_9221422, EPI_ISL_9223212, EPI_ISL_9227890, EPI_ISL_9227931, EPI_ISL_9228410, EPI_ISL_9228449, EPI_ISL_9228465, EPI_ISL_9228521, EPI_ISL_9228533, EPI_ISL_9228539, EPI_ISL_9228592, EPI_ISL_9228654, EPI_ISL_9228701, EPI_ISL_9230255			
see above	MVZ Labor Dr. Limbach & Kollegen GbR	Robert Koch Institute	
EPI_ISL_9217306	MVZ Labor Dr. Reising-Ackermann und Kollegen	Robert Koch Institute	
EPI_ISL_9228029, EPI_ISL_9228115, EPI_ISL_9228244	MVZ Labor Krone GbR	Robert Koch Institute	
EPI_ISL_9221270, EPI_ISL_9221389	MVZ Medizinisches Labor Hannover GmbH	Robert Koch Institute	
EPI_ISL_9216903	Medizinisches Labor Ostsachsen MVZ GbR Nebenbetriebsstätte Dresden	Robert Koch Institute	
EPI_ISL_9218276	SYNLAB MVZ Leinfelden-Echterdingen	Robert Koch Institute	
EPI_ISL_9218317	SYNLAB MVZ Leverkusen	Robert Koch Institute	
EPI_ISL_9220560	Sonic - Labor Dr. von Froreich GmbH	Robert Koch Institute	
EPI_ISL_9229725, EPI_ISL_9229810	Uniklinikum Carl Gustav Carus an der TU Dresden; Institut für Virologie	Robert Koch Institute	
EPI_ISL_9225281	nordlab - Partnerschaftspraxis für Laboratoriumsmedizin	Robert Koch Institute	

We gratefully acknowledge the following Authors from the Originating laboratories responsible for obtaining the specimens, as well as the Submitting laboratories where the genome data were generated and shared via GISAID, on which this research is based.

All Submitters of data may be contacted directly via www.gisaid.org

Authors are sorted alphabetically.

Accession ID	Originating Laboratory	Submitting Laboratory	Authors
EPI_ISL_9244732	INT Fondazione Pascale	INT Fondazione Pascale	N. Normanno INT Fondazione Pascale

We gratefully acknowledge the following Authors from the Originating laboratories responsible for obtaining the specimens, as well as the Submitting laboratories where the genome data were generated and shared via GISAID, on which this research is based.

All Submitters of data may be contacted directly via www.gisaid.org

Authors are sorted alphabetically.

Acknowledgement EPI_SET Identifier: EPI_SET_20220527wn

Accession ID	Originating Laboratory	Submitting Laboratory	Authors
EPI_ISL_9247372	Atrium Health Wake Forest Baptist Microbiology Lab	Wake Forest School of Medicine, Internal Medicine	Gregory Hawkins; Kimberly Reeves
EPI_ISL_9260026, EPI_ISL_9260031, EPI_ISL_9260034	Community Labs, San Antonio	STRL UT Health San Antonio, Greehey Children's Cancer Research Institute	Bethany Landry; Dawn Garcia; Guillermo Nunez; Hongxin Fan; Josefina Stoever; Korri Weldon; Kumari Vadlamudi; Marjorie Parker David; San Antonio Metropolitan Health District; Texas Department of State Health Services; Weijing He; Yidong Chen; Zhao Lai; Zhenqing Ye
EPI_ISL_9212993, EPI_ISL_9212999, EPI_ISL_9213000, EPI_ISL_9213040, EPI_ISL_9213148, EPI_ISL_9213151, EPI_ISL_9213170, EPI_ISL_9215135, EPI_ISL_9215138, EPI_ISL_9215151, EPI_ISL_9215156, EPI_ISL_9215175, EPI_ISL_9215197, EPI_ISL_9215205, EPI_ISL_9215214, EPI_ISL_9215232, EPI_ISL_9215307, EPI_ISL_9215319, EPI_ISL_9215323, EPI_ISL_9215324, EPI_ISL_9215336, EPI_ISL_9215338, EPI_ISL_9215352, EPI_ISL_9215402, EPI_ISL_9215459, EPI_ISL_9215463, EPI_ISL_9215466, EPI_ISL_9215472			
see above	Curative Labs	Curative Labs	Elias L. Salfati; Eugenia Khorosheva; George Way; J.Cesar Ignacio-Espinoza; Janet Chen; Mikhail Hanewich-Hollatz; Nabjot Sandhu; Sophia Quasem; Vladimir Slepnev; Zhiyi Xie
EPI_ISL_9203423, EPI_ISL_9205186, EPI_ISL_9205211, EPI_ISL_9205233, EPI_ISL_9205301, EPI_ISL_9205589, EPI_ISL_9205638			
see above	Houston Methodist Hospital	Houston Methodist Hospital	Akanksha Batajoo; Ilya J. Finkelstein; James J. Davis; Jessica Cambric; Jimmy Gollihar; Kristina Reppond; Madison N. Shyer; Marcus Nguyen; Matthew Ojeda Saavedra; Paul A. Christensen; Randall J. Olsen; Rashi Thakur; Regan Mangham; Richard Snehall; Robert Olson; Ryan Gadd; S. Wesley Long; Sindy Pena; and James M. Musser
EPI_ISL_9249877	Kansas Health and Environmental Lab	Kansas Health and Environmental Lab	Amanda Bradley; Carrie Welch; Gary Buruss; Jonathan Barnell; Mike Grose; Victor Anderson; and Phil Adam
EPI_ISL_9257017	LABORATORIO DE PATOLOGÍA MOLECULAR CENTRO MEDICO NAVAL SECRETARIA DE MARINA ARMADA DE MÉXICO	LABORATORIO DE PATOLOGÍA MOLECULAR CENTRO MEDICO NAVAL SECRETARIA DE MARINA ARMADA DE MÉXICO	ALVAREZ JIMENEZ VIOLETA DEJANIRA; BALTIERRA URIBE SHANTAL; GARCIA ALEGRIA IRMA.; MEIXUEIRO CALDERON CLAUDIA; MORALES RAMIREZ OMAR; ZUNO CORONADO TEUTLI
EPI_ISL_9257041, EPI_ISL_9257047, EPI_ISL_9257052, EPI_ISL_9257056, EPI_ISL_9257062, EPI_ISL_9257104, EPI_ISL_9257126			
see above	Michigan Department of Health and Human Services, Bureau of Laboratories	Michigan Department of Health and Human Services, Bureau of Laboratories	Blankenship HM; Riner D; Soehnlen MK
EPI_ISL_9206155, EPI_ISL_9206179	Stanford Health Care	Stanford University School of Medicine, Clinical Virology Laboratory	Malaya K. Sahoo; Mamdouh Sibai; Selamawit Bihon; Suzette Santos; and Benjamin A. Pinsky
EPI_ISL_9242509	The Jackson Laboratory	The Jackson Laboratory	Adams M; Kelly K; Li L; Long E; Omerza G; Renzette N

We gratefully acknowledge the following Authors from the Originating laboratories responsible for obtaining the specimens, as well as the Submitting laboratories where the genome data were generated and shared via GISAID, on which this research is based.

All Submitters of data may be contacted directly via www.gisaid.org

Authors are sorted alphabetically.

Accession ID	Originating Laboratory	Submitting Laboratory	Authors
EPI_ISL_9209546	LabPLUS	Institute of Environmental Science and Research (ESR)	Anja Werno; Antje van der Linden; Arlo Upton; Chris Mansell; Clare Gebbie; David Hammer; Dhanisha Patel; Dragana Drinkovic; Erasmus Smit; Gary McAuliffe; Hana Sofia Andersson; Hermes Perez; James Ussher; Jill Sherwood; Jing Wang; Joep de Ligt; Josh Freeman; Julia Howard; Juliet Elvy; Lauren Jelly; Mary DeAlmeida; Matt Blakiston; Matt Storey; Matthew Rogers; Max Bloomfield; Michael Addidle; Michelle Balm; Muhammad Faisal; Nikki Freed; Olin Silander; Olivia Stroeven; Rachel Boyle; Sally Roberts; SallyAnn Harbison; Sarah Cockerton; Sarah Jefferies; Sharmini Muttaiyah; Susan Morpeth; Susan Taylor; Timothy Blackmore; Vani Sathyendran; Veronica Playle; Virginia Hope; Xiaoyun Ren

We gratefully acknowledge the following Authors from the Originating laboratories responsible for obtaining the specimens, as well as the Submitting laboratories where the genome data were generated and shared via GISAID, on which this research is based.

All Submitters of data may be contacted directly via www.gisaid.org

Authors are sorted alphabetically.

Accession ID	Originating Laboratory	Submitting Laboratory	Authors

EPI_ISL_9268793, EPI_ISL_9268813, EPI_ISL_9268821, EPI_ISL_9268835, EPI_ISL_9268852, EPI_ISL_9268857, EPI_ISL_9268877, EPI_ISL_9268886, EPI_ISL_9268891, EPI_ISL_9268892, EPI_ISL_9268893, EPI_ISL_9268896, EPI_ISL_9268900, EPI_ISL_9268902, EPI_ISL_9268930, EPI_ISL_9268932, EPI_ISL_9268946, EPI_ISL_9268956, EPI_ISL_9268959, EPI_ISL_9268962, EPI_ISL_9268964, EPI_ISL_9268974, EPI_ISL_9268991, EPI_ISL_9268994, EPI_ISL_9268996, EPI_ISL_9269010, EPI_ISL_9269011, EPI_ISL_9269019, EPI_ISL_9269023, EPI_ISL_9269030, EPI_ISL_9269031, EPI_ISL_9269032, EPI_ISL_9269034, EPI_ISL_9269041, EPI_ISL_9269043, EPI_ISL_9269049, EPI_ISL_9269051, EPI_ISL_9269055, EPI_ISL_9269056, EPI_ISL_9269057, EPI_ISL_9269063, EPI_ISL_9269071, EPI_ISL_9269078, EPI_ISL_9269101, EPI_ISL_9269102, EPI_ISL_9269105, EPI_ISL_9269106, EPI_ISL_9269111, EPI_ISL_9269112, EPI_ISL_9269117, EPI_ISL_9269118, EPI_ISL_9269125, EPI_ISL_9269126, EPI_ISL_9269131, EPI_ISL_9269139, EPI_ISL_9269152, EPI_ISL_9269164, EPI_ISL_9269188, EPI_ISL_9269189, EPI_ISL_9269191, EPI_ISL_9269197, EPI_ISL_9269198, EPI_ISL_9269200, EPI_ISL_9269204, EPI_ISL_9269211, EPI_ISL_9269212, EPI_ISL_9269216, EPI_ISL_9269229, EPI_ISL_9269234, EPI_ISL_9269242, EPI_ISL_9269258, EPI_ISL_9269283, EPI_ISL_9269307, EPI_ISL_9269313, EPI_ISL_9269325, EPI_ISL_9269332, EPI_ISL_9269340, EPI_ISL_9269347, EPI_ISL_9269349, EPI_ISL_9269350, EPI_ISL_9269363, EPI_ISL_9269381, EPI_ISL_9269383, EPI_ISL_9269388, EPI_ISL_9269390, EPI_ISL_9269393, EPI_ISL_9269394, EPI_ISL_9269401, EPI_ISL_9269403, EPI_ISL_9269406, EPI_ISL_9269427, EPI_ISL_9269455, EPI_ISL_9269459, EPI_ISL_9269467, EPI_ISL_9269470, EPI_ISL_9269472, EPI_ISL_9269477, EPI_ISL_9269482, EPI_ISL_9269487, EPI_ISL_9269490, EPI_ISL_9269502, EPI_ISL_9269503, EPI_ISL_9269505, EPI_ISL_9269509, EPI_ISL_9269510, EPI_ISL_9269512, EPI_ISL_9269514, EPI_ISL_9269515, EPI_ISL_9269516, EPI_ISL_9269520, EPI_ISL_9269528, EPI_ISL_9269531, EPI_ISL_9269533, EPI_ISL_9269554, EPI_ISL_9269555, EPI_ISL_9269559, EPI_ISL_9269562, EPI_ISL_9269568, EPI_ISL_9269584, EPI_ISL_9269591, EPI_ISL_9269592, EPI_ISL_9269599, EPI_ISL_9269603, EPI_ISL_9269608, EPI_ISL_9269618, EPI_ISL_9269627, EPI_ISL_9269628, EPI_ISL_9269631, EPI_ISL_9269643, EPI_ISL_9269652, EPI_ISL_9269653, EPI_ISL_9269669, EPI_ISL_9269689, EPI_ISL_9269703, EPI_ISL_9269710, EPI_ISL_9269721, EPI_ISL_9269728, EPI_ISL_9269731, EPI_ISL_9269734, EPI_ISL_9269737, EPI_ISL_9269739, EPI_ISL_9269741, EPI_ISL_9269744, EPI_ISL_9269754, EPI_ISL_9269758, EPI_ISL_9269781, EPI_ISL_9269787, EPI_ISL_9269788, EPI_ISL_9269790, EPI_ISL_9269791, EPI_ISL_9269799, EPI_ISL_9269819, EPI_ISL_9269832, EPI_ISL_9269833, EPI_ISL_9269834, EPI_ISL_9269843, EPI_ISL_9269845, EPI_ISL_9269852, EPI_ISL_9269856, EPI_ISL_9269857, EPI_ISL_9269861, EPI_ISL_9269866, EPI_ISL_9269869, EPI_ISL_9269873, EPI_ISL_9269898, EPI_ISL_9269899, EPI_ISL_9269912, EPI_ISL_9269928, EPI_ISL_9269930, EPI_ISL_9269945, EPI_ISL_9269956, EPI_ISL_9269968, EPI_ISL_9269970, EPI_ISL_9269977, EPI_ISL_9269979, EPI_ISL_9269980, EPI_ISL_9269998, EPI_ISL_9270004, EPI_ISL_9270032, EPI_ISL_9270033, EPI_ISL_9270034, EPI_ISL_9270039, EPI_ISL_9270063, EPI_ISL_9270068, EPI_ISL_9270069, EPI_ISL_9270072, EPI_ISL_9270074, EPI_ISL_9270080, EPI_ISL_9270084, EPI_ISL_9270088, EPI_ISL_9270092, EPI_ISL_9270096, EPI_ISL_9270103, EPI_ISL_9270105, EPI_ISL_9270106, EPI_ISL_9270118, EPI_ISL_9270121, EPI_ISL_9270122, EPI_ISL_9270123, EPI_ISL_9270141, EPI_ISL_9270150, EPI_ISL_9270164, EPI_ISL_9270172, EPI_ISL_9270191, EPI_ISL_9270196, EPI_ISL_9270197, EPI_ISL_9270203, EPI_ISL_9270207, EPI_ISL_9270220, EPI_ISL_9270221, EPI_ISL_9270224, EPI_ISL_9270239, EPI_ISL_9270255, EPI_ISL_9270259, EPI_ISL_9270288, EPI_ISL_9270293, EPI_ISL_9270296, EPI_ISL_9270298, EPI_ISL_9270311, EPI_ISL_9270330, EPI_ISL_9270331, EPI_ISL_9270334, EPI_ISL_9270341, EPI_ISL_9270346, EPI_ISL_9270357, EPI_ISL_9270361, EPI_ISL_9270390, EPI_ISL_9270392, EPI_ISL_9270395, EPI_ISL_9270406, EPI_ISL_9270415, EPI_ISL_9270416, EPI_ISL_9270420, EPI_ISL_9270424, EPI_ISL_9270426, EPI_ISL_9270428, EPI_ISL_9270429, EPI_ISL_9270431, EPI_ISL_9270433, EPI_ISL_9270437, EPI_ISL_9270439, EPI_ISL_9270440, EPI_ISL_9270442, EPI_ISL_9270445, EPI_ISL_9270447, EPI_ISL_9270458, EPI_ISL_9270461, EPI_ISL_9270466, EPI_ISL_9270470, EPI_ISL_9270473, EPI_ISL_9270478, EPI_ISL_9270479, EPI_ISL_9270491, EPI_ISL_9270494, EPI_ISL_9270501, EPI_ISL_9270511, EPI_ISL_9270518, EPI_ISL_9270522, EPI_ISL_9270523, EPI_ISL_9270528, EPI_ISL_9270531, EPI_ISL_9270538, EPI_ISL_9270542, EPI_ISL_9270555, EPI_ISL_9270556, EPI_ISL_9270559, EPI_ISL_9270560, EPI_ISL_9270562, EPI_ISL_9270567, EPI_ISL_9270576, EPI_ISL_9270577, EPI_ISL_9270582, EPI_ISL_9270587, EPI_ISL_9270604, EPI_ISL_9270618, EPI_ISL_9270621, EPI_ISL_9270643, EPI_ISL_9270644, EPI_ISL_9270655, EPI_ISL_9270673, EPI_ISL_9270684, EPI_ISL_9270690, EPI_ISL_9270691, EPI_ISL_9270699, EPI_ISL_9270700, EPI_ISL_9270714, EPI_ISL_9270727, EPI_ISL_9270738, EPI_ISL_9270749, EPI_ISL_9270763, EPI_ISL_9270773, EPI_ISL_9270776, EPI_ISL_9270784, EPI_ISL_9270789, EPI_ISL_9270800, EPI_ISL_9270801, EPI_ISL_9270802, EPI_ISL_9270807, EPI_ISL_9270813, EPI_ISL_9270817, EPI_ISL_9270835, EPI_ISL_9270842, EPI_ISL_9270846, EPI_ISL_9270849, EPI_ISL_9270853, EPI_ISL_9270862, EPI_ISL_9270872, EPI_ISL_9270893, EPI_ISL_9270901, EPI_ISL_9270902, EPI_ISL_9270908, EPI_ISL_9270914, EPI_ISL_9270924, EPI_ISL_9270928, EPI_ISL_9270930, EPI_ISL_9270932, EPI_ISL_9270934, EPI_ISL_9270935, EPI_ISL_9270940, EPI_ISL_9270944, EPI_ISL_9270947, EPI_ISL_9270952, EPI_ISL_9270957, EPI_ISL_9270958, EPI_ISL_9270960, EPI_ISL_9270961, EPI_ISL_9270968, EPI_ISL_9270972, EPI_ISL_9270979, EPI_ISL_9270986, EPI_ISL_9270990, EPI_ISL_9270998, EPI_ISL_9271011, EPI_ISL_9271012, EPI_ISL_9271015, EPI_ISL_9271020, EPI_ISL_9271021, EPI_ISL_9271027, EPI_ISL_9271032, EPI_ISL_9271037, EPI_ISL_9271041, EPI_ISL_9271053, EPI_ISL_9271066, EPI_ISL_9271079, EPI_ISL_9271095, EPI_ISL_9271100, EPI_ISL_9271110, EPI_ISL_9271116, EPI_ISL_9271119, EPI_ISL_9271120, EPI_ISL_9271121, EPI_ISL_9271126, EPI_ISL_9271129, EPI_ISL_9271133, EPI_ISL_9271144, EPI_ISL_9271146, EPI_ISL_9271147, EPI_ISL_9271164, EPI_ISL_9271168, EPI_ISL_9271173, EPI_ISL_9271176, EPI_ISL_9271208, EPI_ISL_9271212, EPI_ISL_9271213, EPI_ISL_9271214, EPI_ISL_9271218, EPI_ISL_9271229, EPI_ISL_9271232, EPI_ISL_9271248, EPI_ISL_9271272, EPI_ISL_9271280, EPI_ISL_9271291, EPI_ISL_9271293, EPI_ISL_9271294, EPI_ISL_9271313, EPI_ISL_9271321, EPI_ISL_9271326, EPI_ISL_9271327, EPI_ISL_9271330, EPI_ISL_9271374, EPI_ISL_9271377, EPI_ISL_9271382, EPI_ISL_9271386, EPI_ISL_9271390, EPI_ISL_9271392, EPI_ISL_9271403, EPI_ISL_9271413, EPI_ISL_9271451, EPI_ISL_9271463, EPI_ISL_9271465, EPI_ISL_9271484, EPI_ISL_9271489, EPI_ISL_9271496, EPI_ISL_9271500, EPI_ISL_9271501, EPI_ISL_9271505, EPI_ISL_9271526, EPI_ISL_9271527, EPI_ISL_9271541, EPI_ISL_9271546, EPI_ISL_9271547, EPI_ISL_9271559, EPI_ISL_9271563, EPI_ISL_9271571, EPI_ISL_9271572, EPI_ISL_9271575, EPI_ISL_9271576, EPI_ISL_9271577, EPI_ISL_9271579, EPI_ISL_9271583, EPI_ISL_9271590, EPI_ISL_9271594, EPI_ISL_9271605, EPI_ISL_9271615, EPI_ISL_9271621

Accession ID	Originating Laboratory	Submitting Laboratory	Authors
see above	Department of Bacteria, Parasites and Fungi, Statens Serum Institut, Copenhagen, Denmark	Statens Serum Institut Bioinformatics and Microbial Genomics	Danish Covid-19 Genome Consortium

We gratefully acknowledge the following Authors from the Originating laboratories responsible for obtaining the specimens, as well as the Submitting laboratories where the genome data were generated and shared via GISAID, on which this research is based.

All Submitters of data may be contacted directly via www.gisaid.org

Authors are sorted alphabetically.

EPI_ISL_9268790, EPI_ISL_9268791, EPI_ISL_9268798, EPI_ISL_9268802, EPI_ISL_9268803, EPI_ISL_9268824, EPI_ISL_9268825, EPI_ISL_9268830, EPI_ISL_9268831, EPI_ISL_9268832, EPI_ISL_9268844, EPI_ISL_9268849, EPI_ISL_9268858, EPI_ISL_9268862, EPI_ISL_9268863, EPI_ISL_9268865, EPI_ISL_9268876, EPI_ISL_9268885, EPI_ISL_9268894, EPI_ISL_9268907, EPI_ISL_9268909, EPI_ISL_9268917, EPI_ISL_9268923, EPI_ISL_9268925, EPI_ISL_9268936, EPI_ISL_9268952, EPI_ISL_9268960, EPI_ISL_9268966, EPI_ISL_9268967, EPI_ISL_9268981, EPI_ISL_9268983, EPI_ISL_9268985, EPI_ISL_9268989, EPI_ISL_9269000, EPI_ISL_9269003, EPI_ISL_9269004, EPI_ISL_9269005, EPI_ISL_9269016, EPI_ISL_9269018, EPI_ISL_9269028, EPI_ISL_9269037, EPI_ISL_9269042, EPI_ISL_9269050, EPI_ISL_9269052, EPI_ISL_9269061, EPI_ISL_9269062, EPI_ISL_9269064, EPI_ISL_9269076, EPI_ISL_9269083, EPI_ISL_9269086, EPI_ISL_9269096, EPI_ISL_9269113, EPI_ISL_9269120, EPI_ISL_9269121, EPI_ISL_9269134, EPI_ISL_9269150, EPI_ISL_9269161, EPI_ISL_9269171, EPI_ISL_9269176, EPI_ISL_9269178, EPI_ISL_9269184, EPI_ISL_9269186, EPI_ISL_9269206, EPI_ISL_9269210, EPI_ISL_9269223, EPI_ISL_9269228, EPI_ISL_9269231, EPI_ISL_9269232, EPI_ISL_9269260, EPI_ISL_9269261, EPI_ISL_9269269, EPI_ISL_9269281, EPI_ISL_9269296, EPI_ISL_9269298, EPI_ISL_9269303, EPI_ISL_9269315, EPI_ISL_9269316, EPI_ISL_9269319, EPI_ISL_9269326, EPI_ISL_9269338, EPI_ISL_9269356, EPI_ISL_9269360, EPI_ISL_9269361, EPI_ISL_9269365, EPI_ISL_9269366, EPI_ISL_9269376, EPI_ISL_9269378, EPI_ISL_9269398, EPI_ISL_9269404, EPI_ISL_9269405, EPI_ISL_9269414, EPI_ISL_9269417, EPI_ISL_9269422, EPI_ISL_9269424, EPI_ISL_9269438, EPI_ISL_9269449, EPI_ISL_9269457, EPI_ISL_9269460, EPI_ISL_9269463, EPI_ISL_9269466, EPI_ISL_9269506, EPI_ISL_9269523, EPI_ISL_9269538, EPI_ISL_9269544, EPI_ISL_9269545, EPI_ISL_9269546, EPI_ISL_9269551, EPI_ISL_9269557, EPI_ISL_9269564, EPI_ISL_9269570, EPI_ISL_9269571, EPI_ISL_9269588, EPI_ISL_9269594, EPI_ISL_9269595, EPI_ISL_9269596, EPI_ISL_9269597, EPI_ISL_9269598, EPI_ISL_9269600, EPI_ISL_9269610, EPI_ISL_9269611, EPI_ISL_9269614, EPI_ISL_9269616, EPI_ISL_9269619, EPI_ISL_9269642, EPI_ISL_9269648, EPI_ISL_9269651, EPI_ISL_9269666, EPI_ISL_9269671, EPI_ISL_9269680, EPI_ISL_9269686, EPI_ISL_9269688, EPI_ISL_9269692, EPI_ISL_9269696, EPI_ISL_9269697, EPI_ISL_9269698, EPI_ISL_9269700, EPI_ISL_9269705, EPI_ISL_9269726, EPI_ISL_9269733, EPI_ISL_9269753, EPI_ISL_9269755, EPI_ISL_9269756, EPI_ISL_9269762, EPI_ISL_9269769, EPI_ISL_9269775, EPI_ISL_9269784, EPI_ISL_9269803, EPI_ISL_9269820, EPI_ISL_9269821, EPI_ISL_9269825, EPI_ISL_9269840, EPI_ISL_9269849, EPI_ISL_9269854, EPI_ISL_9269859, EPI_ISL_9269862, EPI_ISL_9269864, EPI_ISL_9269874, EPI_ISL_9269885, EPI_ISL_9269886, EPI_ISL_9269890, EPI_ISL_9269910, EPI_ISL_9269911, EPI_ISL_9269957, EPI_ISL_9269966, EPI_ISL_9269974, EPI_ISL_9269985, EPI_ISL_9269987, EPI_ISL_9270026, EPI_ISL_9270027, EPI_ISL_9270031, EPI_ISL_9270046, EPI_ISL_9270048, EPI_ISL_9270054, EPI_ISL_9270061, EPI_ISL_9270062, EPI_ISL_9270075, EPI_ISL_9270093, EPI_ISL_9270100, EPI_ISL_9270104, EPI_ISL_9270126, EPI_ISL_9270128, EPI_ISL_9270132, EPI_ISL_9270134, EPI_ISL_9270137, EPI_ISL_9270148, EPI_ISL_9270149, EPI_ISL_9270155, EPI_ISL_9270157, EPI_ISL_9270168, EPI_ISL_9270169, EPI_ISL_9270174, EPI_ISL_9270176, EPI_ISL_9270185, EPI_ISL_9270194, EPI_ISL_9270216, EPI_ISL_9270225, EPI_ISL_9270226, EPI_ISL_9270237, EPI_ISL_9270241, EPI_ISL_9270245, EPI_ISL_9270246, EPI_ISL_9270249, EPI_ISL_9270271, EPI_ISL_9270275, EPI_ISL_9270278, EPI_ISL_9270282, EPI_ISL_9270284, EPI_ISL_9270290, EPI_ISL_9270297, EPI_ISL_9270299, EPI_ISL_9270302, EPI_ISL_9270305, EPI_ISL_9270310, EPI_ISL_9270316, EPI_ISL_9270348, EPI_ISL_9270350, EPI_ISL_9270353, EPI_ISL_9270359, EPI_ISL_9270368, EPI_ISL_9270379, EPI_ISL_9270397, EPI_ISL_9270418, EPI_ISL_9270438, EPI_ISL_9270441, EPI_ISL_9270444, EPI_ISL_9270452, EPI_ISL_9270471, EPI_ISL_9270474, EPI_ISL_9270476, EPI_ISL_9270480, EPI_ISL_9270495, EPI_ISL_9270496, EPI_ISL_9270500, EPI_ISL_9270502, EPI_ISL_9270507, EPI_ISL_9270515, EPI_ISL_9270516, EPI_ISL_9270519, EPI_ISL_9270525, EPI_ISL_9270529, EPI_ISL_9270533, EPI_ISL_9270535, EPI_ISL_9270539, EPI_ISL_9270554, EPI_ISL_9270563, EPI_ISL_9270568, EPI_ISL_9270571, EPI_ISL_9270574, EPI_ISL_9270607, EPI_ISL_9270611, EPI_ISL_9270614, EPI_ISL_9270622, EPI_ISL_9270625, EPI_ISL_9270628, EPI_ISL_9270650, EPI_ISL_9270652, EPI_ISL_9270653, EPI_ISL_9270654, EPI_ISL_9270669, EPI_ISL_9270679, EPI_ISL_9270683, EPI_ISL_9270692, EPI_ISL_9270694, EPI_ISL_9270697, EPI_ISL_9270701, EPI_ISL_9270702, EPI_ISL_9270726, EPI_ISL_9270734, EPI_ISL_9270737, EPI_ISL_9270741, EPI_ISL_9270744, EPI_ISL_9270777, EPI_ISL_9270779, EPI_ISL_9270781, EPI_ISL_9270787, EPI_ISL_9270794, EPI_ISL_9270795, EPI_ISL_9270799, EPI_ISL_9270810, EPI_ISL_9270812, EPI_ISL_9270815, EPI_ISL_9270818, EPI_ISL_9270820, EPI_ISL_9270824, EPI_ISL_9270864, EPI_ISL_9270868, EPI_ISL_9270881, EPI_ISL_9270888, EPI_ISL_9270892, EPI_ISL_9270899, EPI_ISL_9270910, EPI_ISL_9270919, EPI_ISL_9270920, EPI_ISL_9270936, EPI_ISL_9270941, EPI_ISL_9270946, EPI_ISL_9270955, EPI_ISL_9270966, EPI_ISL_9270983, EPI_ISL_9270985, EPI_ISL_9270996, EPI_ISL_9271005, EPI_ISL_9271009, EPI_ISL_9271023, EPI_ISL_9271034, EPI_ISL_9271056, EPI_ISL_9271068, EPI_ISL_9271070, EPI_ISL_9271073, EPI_ISL_9271074, EPI_ISL_9271084, EPI_ISL_9271085, EPI_ISL_9271092, EPI_ISL_9271103, EPI_ISL_9271112, EPI_ISL_9271127, EPI_ISL_9271137, EPI_ISL_9271150, EPI_ISL_9271154, EPI_ISL_9271155, EPI_ISL_9271156, EPI_ISL_9271159, EPI_ISL_9271160, EPI_ISL_9271175, EPI_ISL_9271182, EPI_ISL_9271192, EPI_ISL_9271202, EPI_ISL_9271205, EPI_ISL_9271209, EPI_ISL_9271215, EPI_ISL_9271223, EPI_ISL_9271225, EPI_ISL_9271227, EPI_ISL_9271238, EPI_ISL_9271239, EPI_ISL_9271241, EPI_ISL_9271244, EPI_ISL_9271247, EPI_ISL_9271250, EPI_ISL_9271261, EPI_ISL_9271287, EPI_ISL_9271295, EPI_ISL_9271299, EPI_ISL_9271300, EPI_ISL_9271301, EPI_ISL_9271304, EPI_ISL_9271312, EPI_ISL_9271314, EPI_ISL_9271325, EPI_ISL_9271331, EPI_ISL_9271337, EPI_ISL_9271339, EPI_ISL_9271340, EPI_ISL_9271341, EPI_ISL_9271349, EPI_ISL_9271359, EPI_ISL_9271375, EPI_ISL_9271383, EPI_ISL_9271387, EPI_ISL_9271391, EPI_ISL_9271405, EPI_ISL_9271411, EPI_ISL_9271429, EPI_ISL_9271431, EPI_ISL_9271435, EPI_ISL_9271441, EPI_ISL_9271444, EPI_ISL_9271448, EPI_ISL_9271450, EPI_ISL_9271453, EPI_ISL_9271459, EPI_ISL_9271468, EPI_ISL_9271476, EPI_ISL_9271481, EPI_ISL_9271492, EPI_ISL_9271493, EPI_ISL_9271494, EPI_ISL_9271502, EPI_ISL_9271514, EPI_ISL_9271521, EPI_ISL_9271528, EPI_ISL_9271530, EPI_ISL_9271534, EPI_ISL_9271540, EPI_ISL_9271555, EPI_ISL_9271566, EPI_ISL_9271567, EPI_ISL_9271578, EPI_ISL_9271585, EPI_ISL_9271591, EPI_ISL_9271595, EPI_ISL_9271604, EPI_ISL_9271618, EPI_ISL_9271627

**Accession ID**	**Originating Laboratory**	**Submitting Laboratory**	**Authors**
see above	Department of Bacteria, Parasites and Fungi, Statens Serum Institut, Copenhagen, Denmark	Statens Serum Institut Bioinformatics and Microbial Genomics	Danish Covid-19 Genome Consortium

We gratefully acknowledge the following Authors from the Originating laboratories responsible for obtaining the specimens, as well as the Submitting laboratories where the genome data were generated and shared via GISAID, on which this research is based.

All Submitters of data may be contacted directly via www.gisaid.org

Authors are sorted alphabetically.

Accession ID	Originating Laboratory	Submitting Laboratory	Authors
EPI_ISL_9268856, EPI_ISL_9268908, EPI_ISL_9268915, EPI_ISL_9268927, EPI_ISL_9268931, EPI_ISL_9268943, EPI_ISL_9268949, EPI_ISL_9268971, EPI_ISL_9268972, EPI_ISL_9268980, EPI_ISL_9269014, EPI_ISL_9269053, EPI_ISL_9269080, EPI_ISL_9269103, EPI_ISL_9269108, EPI_ISL_9269133, EPI_ISL_9269158, EPI_ISL_9269259, EPI_ISL_9269282, EPI_ISL_9269317, EPI_ISL_9269330, EPI_ISL_9269343, EPI_ISL_9269345, EPI_ISL_9269375, EPI_ISL_9269387, EPI_ISL_9269407, EPI_ISL_9269411, EPI_ISL_9269419, EPI_ISL_9269431, EPI_ISL_9269444, EPI_ISL_9269462, EPI_ISL_9269491, EPI_ISL_9269507, EPI_ISL_9269582, EPI_ISL_9269630, EPI_ISL_9269636, EPI_ISL_9269656, EPI_ISL_9269658, EPI_ISL_9269714, EPI_ISL_9269715, EPI_ISL_9269718, EPI_ISL_9269729, EPI_ISL_9269730, EPI_ISL_9269800, EPI_ISL_9269835, EPI_ISL_9269860, EPI_ISL_9269915, EPI_ISL_9269922, EPI_ISL_9269923, EPI_ISL_9269949, EPI_ISL_9269955, EPI_ISL_9269958, EPI_ISL_9269960, EPI_ISL_9269973, EPI_ISL_9269993, EPI_ISL_9269999, EPI_ISL_9270009, EPI_ISL_9270014, EPI_ISL_9270023, EPI_ISL_9270035, EPI_ISL_9270041, EPI_ISL_9270055, EPI_ISL_9270060, EPI_ISL_9270085, EPI_ISL_9270089, EPI_ISL_9270133, EPI_ISL_9270138, EPI_ISL_9270175, EPI_ISL_9270181, EPI_ISL_9270183, EPI_ISL_9270192, EPI_ISL_9270200, EPI_ISL_9270219, EPI_ISL_9270242, EPI_ISL_9270257, EPI_ISL_9270295, EPI_ISL_9270324, EPI_ISL_9270333, EPI_ISL_9270335, EPI_ISL_9270358, EPI_ISL_9270377, EPI_ISL_9270382, EPI_ISL_9270399, EPI_ISL_9270421, EPI_ISL_9270435, EPI_ISL_9270455, EPI_ISL_9270486, EPI_ISL_9270508, EPI_ISL_9270550, EPI_ISL_9270566, EPI_ISL_9270570, EPI_ISL_9270586, EPI_ISL_9270589, EPI_ISL_9270602, EPI_ISL_9270631, EPI_ISL_9270708, EPI_ISL_9270709, EPI_ISL_9270710, EPI_ISL_9270712, EPI_ISL_9270713, EPI_ISL_9270722, EPI_ISL_9270728, EPI_ISL_9270730, EPI_ISL_9270758, EPI_ISL_9270783, EPI_ISL_9270804, EPI_ISL_9270827, EPI_ISL_9270830, EPI_ISL_9270844, EPI_ISL_9270850, EPI_ISL_9270852, EPI_ISL_9270858, EPI_ISL_9270876, EPI_ISL_9270880, EPI_ISL_9270906, EPI_ISL_9270911, EPI_ISL_9270943, EPI_ISL_9270963, EPI_ISL_9270964, EPI_ISL_9270969, EPI_ISL_9270971, EPI_ISL_9270982, EPI_ISL_9270987, EPI_ISL_9270989, EPI_ISL_9271064, EPI_ISL_9271077, EPI_ISL_9271178, EPI_ISL_9271188, EPI_ISL_9271245, EPI_ISL_9271273, EPI_ISL_9271278, EPI_ISL_9271286, EPI_ISL_9271289, EPI_ISL_9271307, EPI_ISL_9271351, EPI_ISL_9271428, EPI_ISL_9271432, EPI_ISL_9271437, EPI_ISL_9271462, EPI_ISL_9271466, EPI_ISL_9271532, EPI_ISL_9271544, EPI_ISL_9271545, EPI_ISL_9271548, EPI_ISL_9271550, EPI_ISL_9271561, EPI_ISL_9271562, EPI_ISL_9271564, EPI_ISL_9271588, EPI_ISL_9271592, EPI_ISL_9271600, EPI_ISL_9271628			
see above	Department of Bacteria, Parasites and Fungi, Statens Serum Institut, Copenhagen, Denmark	Statens Serum Institut Bioinformatics and Microbial Genomics	Danish Covid-19 Genome Consortium

We gratefully acknowledge the following Authors from the Originating laboratories responsible for obtaining the specimens, as well as the Submitting laboratories where the genome data were generated and shared via GISAID, on which this research is based.

All Submitters of data may be contacted directly via www.gisaid.org

Authors are sorted alphabetically.

Acknowledgement EPI_SET Identifier: EPI_SET_20220527kt

Accession ID	Originating Laboratory	Submitting Laboratory	Authors
EPI_ISL_9268794, EPI_ISL_9268827, EPI_ISL_9268834, EPI_ISL_9268846, EPI_ISL_9268860, EPI_ISL_9268864, EPI_ISL_9268901, EPI_ISL_9268919, EPI_ISL_9268934, EPI_ISL_9268997, EPI_ISL_9269007, EPI_ISL_9269012, EPI_ISL_9269073, EPI_ISL_9269095, EPI_ISL_9269155, EPI_ISL_9269156, EPI_ISL_9269168, EPI_ISL_9269175, EPI_ISL_9269215, EPI_ISL_9269221, EPI_ISL_9269224, EPI_ISL_9269227, EPI_ISL_9269233, EPI_ISL_9269275, EPI_ISL_9269290, EPI_ISL_9269305, EPI_ISL_9269323, EPI_ISL_9269333, EPI_ISL_9269359, EPI_ISL_9269389, EPI_ISL_9269402, EPI_ISL_9269430, EPI_ISL_9269443, EPI_ISL_9269445, EPI_ISL_9269468, EPI_ISL_9269478, EPI_ISL_9269499, EPI_ISL_9269513, EPI_ISL_9269547, EPI_ISL_9269563, EPI_ISL_9269625, EPI_ISL_9269641, EPI_ISL_9269660, EPI_ISL_9269665, EPI_ISL_9269674, EPI_ISL_9269675, EPI_ISL_9269702, EPI_ISL_9269717, EPI_ISL_9269724, EPI_ISL_9269740, EPI_ISL_9269765, EPI_ISL_9269768, EPI_ISL_9269795, EPI_ISL_9269814, EPI_ISL_9269815, EPI_ISL_9269818, EPI_ISL_9269884, EPI_ISL_9269887, EPI_ISL_9269892, EPI_ISL_9269903, EPI_ISL_9269916, EPI_ISL_9269963, EPI_ISL_9269976, EPI_ISL_9269982, EPI_ISL_9269992, EPI_ISL_9270008, EPI_ISL_9270022, EPI_ISL_9270044, EPI_ISL_9270091, EPI_ISL_9270107, EPI_ISL_9270110, EPI_ISL_9270111, EPI_ISL_9270144, EPI_ISL_9270166, EPI_ISL_9270190, EPI_ISL_9270211, EPI_ISL_9270222, EPI_ISL_9270228, EPI_ISL_9270232, EPI_ISL_9270240, EPI_ISL_9270258, EPI_ISL_9270268, EPI_ISL_9270320, EPI_ISL_9270322, EPI_ISL_9270360, EPI_ISL_9270383, EPI_ISL_9270391, EPI_ISL_9270403, EPI_ISL_9270432, EPI_ISL_9270449, EPI_ISL_9270460, EPI_ISL_9270475, EPI_ISL_9270482, EPI_ISL_9270504, EPI_ISL_9270506, EPI_ISL_9270572, EPI_ISL_9270573, EPI_ISL_9270590, EPI_ISL_9270597, EPI_ISL_9270599, EPI_ISL_9270605, EPI_ISL_9270613, EPI_ISL_9270639, EPI_ISL_9270648, EPI_ISL_9270674, EPI_ISL_9270682, EPI_ISL_9270716, EPI_ISL_9270759, EPI_ISL_9270762, EPI_ISL_9270765, EPI_ISL_9270768, EPI_ISL_9270786, EPI_ISL_9270825, EPI_ISL_9270831, EPI_ISL_9270851, EPI_ISL_9270866, EPI_ISL_9270886, EPI_ISL_9270889, EPI_ISL_9270916, EPI_ISL_9270933, EPI_ISL_9270945, EPI_ISL_9270949, EPI_ISL_9270995, EPI_ISL_9271008, EPI_ISL_9271018, EPI_ISL_9271042, EPI_ISL_9271048, EPI_ISL_9271062, EPI_ISL_9271114, EPI_ISL_9271158, EPI_ISL_9271163, EPI_ISL_9271167, EPI_ISL_9271186, EPI_ISL_9271187, EPI_ISL_9271194, EPI_ISL_9271206, EPI_ISL_9271220, EPI_ISL_9271240, EPI_ISL_9271259, EPI_ISL_9271275, EPI_ISL_9271281, EPI_ISL_9271308, EPI_ISL_9271317, EPI_ISL_9271345, EPI_ISL_9271346, EPI_ISL_9271348, EPI_ISL_9271356, EPI_ISL_9271357, EPI_ISL_9271364, EPI_ISL_9271365, EPI_ISL_9271366, EPI_ISL_9271367, EPI_ISL_9271379, EPI_ISL_9271388, EPI_ISL_9271393, EPI_ISL_9271410, EPI_ISL_9271423, EPI_ISL_9271430, EPI_ISL_9271438, EPI_ISL_9271449, EPI_ISL_9271454, EPI_ISL_9271479, EPI_ISL_9271490, EPI_ISL_9271497, EPI_ISL_9271522, EPI_ISL_9271539, EPI_ISL_9271549, EPI_ISL_9271586, EPI_ISL_9271598, EPI_ISL_9271607, EPI_ISL_9271609, EPI_ISL_9271611, EPI_ISL_9271612, EPI_ISL_9271614			
see above	Department of Bacteria, Parasites and Fungi, Statens Serum Institut, Copenhagen, Denmark	Statens Serum Institut Bioinformatics and Microbial Genomics	Danish Covid-19 Genome Consortium

We gratefully acknowledge the following Authors from the Originating laboratories responsible for obtaining the specimens, as well as the Submitting laboratories where the genome data were generated and shared via GISAID, on which this research is based.

All Submitters of data may be contacted directly via www.gisaid.org

Authors are sorted alphabetically.

Accession ID	Originating Laboratory	Submitting Laboratory	Authors
EPI_ISL_9268792, EPI_ISL_9268795, EPI_ISL_9268797, EPI_ISL_9268799, EPI_ISL_9268804, EPI_ISL_9268806, EPI_ISL_9268811, EPI_ISL_9268812, EPI_ISL_9268814, EPI_ISL_9268815, EPI_ISL_9268816, EPI_ISL_9268819, EPI_ISL_9268826, EPI_ISL_9268839, EPI_ISL_9268843, EPI_ISL_9268867, EPI_ISL_9268868, EPI_ISL_9268874, EPI_ISL_9268884, EPI_ISL_9268887, EPI_ISL_9268889, EPI_ISL_9268904, EPI_ISL_9268920, EPI_ISL_9268954, EPI_ISL_9268965, EPI_ISL_9268968, EPI_ISL_9268970, EPI_ISL_9268995, EPI_ISL_9269008, EPI_ISL_9269024, EPI_ISL_9269025, EPI_ISL_9269035, EPI_ISL_9269059, EPI_ISL_9269065, EPI_ISL_9269066, EPI_ISL_9269067, EPI_ISL_9269091, EPI_ISL_9269107, EPI_ISL_9269116, EPI_ISL_9269135, EPI_ISL_9269146, EPI_ISL_9269148, EPI_ISL_9269149, EPI_ISL_9269159, EPI_ISL_9269166, EPI_ISL_9269170, EPI_ISL_9269174, EPI_ISL_9269177, EPI_ISL_9269181, EPI_ISL_9269199, EPI_ISL_9269201, EPI_ISL_9269202, EPI_ISL_9269205, EPI_ISL_9269208, EPI_ISL_9269209, EPI_ISL_9269217, EPI_ISL_9269226, EPI_ISL_9269230, EPI_ISL_9269235, EPI_ISL_9269237, EPI_ISL_9269241, EPI_ISL_9269244, EPI_ISL_9269245, EPI_ISL_9269248, EPI_ISL_9269251, EPI_ISL_9269253, EPI_ISL_9269263, EPI_ISL_9269268, EPI_ISL_9269274, EPI_ISL_9269280, EPI_ISL_9269284, EPI_ISL_9269287, EPI_ISL_9269289, EPI_ISL_9269293, EPI_ISL_9269304, EPI_ISL_9269314, EPI_ISL_9269321, EPI_ISL_9269324, EPI_ISL_9269335, EPI_ISL_9269336, EPI_ISL_9269337, EPI_ISL_9269341, EPI_ISL_9269344, EPI_ISL_9269371, EPI_ISL_9269386, EPI_ISL_9269392, EPI_ISL_9269397, EPI_ISL_9269408, EPI_ISL_9269413, EPI_ISL_9269451, EPI_ISL_9269452, EPI_ISL_9269453, EPI_ISL_9269454, EPI_ISL_9269469, EPI_ISL_9269480, EPI_ISL_9269483, EPI_ISL_9269486, EPI_ISL_9269494, EPI_ISL_9269497, EPI_ISL_9269498, EPI_ISL_9269508, EPI_ISL_9269511, EPI_ISL_9269522, EPI_ISL_9269525, EPI_ISL_9269530, EPI_ISL_9269532, EPI_ISL_9269536, EPI_ISL_9269537, EPI_ISL_9269539, EPI_ISL_9269540, EPI_ISL_9269541, EPI_ISL_9269548, EPI_ISL_9269550, EPI_ISL_9269566, EPI_ISL_9269572, EPI_ISL_9269580, EPI_ISL_9269583, EPI_ISL_9269586, EPI_ISL_9269587, EPI_ISL_9269612, EPI_ISL_9269620, EPI_ISL_9269629, EPI_ISL_9269633, EPI_ISL_9269637, EPI_ISL_9269640, EPI_ISL_9269657, EPI_ISL_9269663, EPI_ISL_9269664, EPI_ISL_9269679, EPI_ISL_9269681, EPI_ISL_9269693, EPI_ISL_9269695, EPI_ISL_9269711, EPI_ISL_9269713, EPI_ISL_9269719, EPI_ISL_9269720, EPI_ISL_9269722, EPI_ISL_9269725, EPI_ISL_9269736, EPI_ISL_9269749, EPI_ISL_9269763, EPI_ISL_9269773, EPI_ISL_9269785, EPI_ISL_9269789, EPI_ISL_9269796, EPI_ISL_9269801, EPI_ISL_9269802, EPI_ISL_9269811, EPI_ISL_9269812, EPI_ISL_9269823, EPI_ISL_9269827, EPI_ISL_9269831, EPI_ISL_9269844, EPI_ISL_9269846, EPI_ISL_9269870, EPI_ISL_9269875, EPI_ISL_9269878, EPI_ISL_9269879, EPI_ISL_9269881, EPI_ISL_9269889, EPI_ISL_9269900, EPI_ISL_9269904, EPI_ISL_9269907, EPI_ISL_9269913, EPI_ISL_9269921, EPI_ISL_9269926, EPI_ISL_9269927, EPI_ISL_9269932, EPI_ISL_9269940, EPI_ISL_9269942, EPI_ISL_9269944, EPI_ISL_9269946, EPI_ISL_9269962, EPI_ISL_9269965, EPI_ISL_9269975, EPI_ISL_9269990, EPI_ISL_9269997, EPI_ISL_9270013, EPI_ISL_9270017, EPI_ISL_9270019, EPI_ISL_9270020, EPI_ISL_9270021, EPI_ISL_9270028, EPI_ISL_9270030, EPI_ISL_9270036, EPI_ISL_9270047, EPI_ISL_9270049, EPI_ISL_9270071, EPI_ISL_9270077, EPI_ISL_9270081, EPI_ISL_9270083, EPI_ISL_9270095, EPI_ISL_9270101, EPI_ISL_9270109, EPI_ISL_9270113, EPI_ISL_9270115, EPI_ISL_9270124, EPI_ISL_9270139, EPI_ISL_9270140, EPI_ISL_9270145, EPI_ISL_9270147, EPI_ISL_9270154, EPI_ISL_9270156, EPI_ISL_9270158, EPI_ISL_9270160, EPI_ISL_9270162, EPI_ISL_9270163, EPI_ISL_9270165, EPI_ISL_9270167, EPI_ISL_9270171, EPI_ISL_9270179, EPI_ISL_9270187, EPI_ISL_9270188, EPI_ISL_9270189, EPI_ISL_9270199, EPI_ISL_9270209, EPI_ISL_9270210, EPI_ISL_9270214, EPI_ISL_9270217, EPI_ISL_9270218, EPI_ISL_9270229, EPI_ISL_9270230, EPI_ISL_9270234, EPI_ISL_9270235, EPI_ISL_9270248, EPI_ISL_9270250, EPI_ISL_9270262, EPI_ISL_9270265, EPI_ISL_9270272, EPI_ISL_9270273, EPI_ISL_9270277, EPI_ISL_9270292, EPI_ISL_9270300, EPI_ISL_9270307, EPI_ISL_9270313, EPI_ISL_9270315, EPI_ISL_9270323, EPI_ISL_9270325, EPI_ISL_9270332, EPI_ISL_9270351, EPI_ISL_9270352, EPI_ISL_9270355, EPI_ISL_9270364, EPI_ISL_9270367, EPI_ISL_9270380, EPI_ISL_9270381, EPI_ISL_9270400, EPI_ISL_9270409, EPI_ISL_9270411, EPI_ISL_9270417, EPI_ISL_9270425, EPI_ISL_9270430, EPI_ISL_9270436, EPI_ISL_9270443, EPI_ISL_9270446, EPI_ISL_9270450, EPI_ISL_9270464, EPI_ISL_9270481, EPI_ISL_9270483, EPI_ISL_9270485, EPI_ISL_9270487, EPI_ISL_9270490, EPI_ISL_9270505, EPI_ISL_9270512, EPI_ISL_9270513, EPI_ISL_9270517, EPI_ISL_9270520, EPI_ISL_9270530, EPI_ISL_9270541, EPI_ISL_9270543, EPI_ISL_9270548, EPI_ISL_9270557, EPI_ISL_9270564, EPI_ISL_9270575, EPI_ISL_9270583, EPI_ISL_9270585, EPI_ISL_9270588, EPI_ISL_9270592, EPI_ISL_9270595, EPI_ISL_9270596, EPI_ISL_9270600, EPI_ISL_9270601, EPI_ISL_9270603, EPI_ISL_9270610, EPI_ISL_9270630, EPI_ISL_9270640, EPI_ISL_9270641, EPI_ISL_9270651, EPI_ISL_9270667, EPI_ISL_9270678, EPI_ISL_9270685, EPI_ISL_9270686, EPI_ISL_9270689, EPI_ISL_9270705, EPI_ISL_9270718, EPI_ISL_9270723, EPI_ISL_9270724, EPI_ISL_9270731, EPI_ISL_9270735, EPI_ISL_9270739, EPI_ISL_9270743, EPI_ISL_9270745, EPI_ISL_9270752, EPI_ISL_9270757, EPI_ISL_9270772, EPI_ISL_9270793, EPI_ISL_9270796, EPI_ISL_9270805, EPI_ISL_9270811, EPI_ISL_9270816, EPI_ISL_9270829, EPI_ISL_9270832, EPI_ISL_9270833, EPI_ISL_9270854, EPI_ISL_9270855, EPI_ISL_9270857, EPI_ISL_9270861, EPI_ISL_9270863, EPI_ISL_9270874, EPI_ISL_9270877, EPI_ISL_9270878, EPI_ISL_9270879, EPI_ISL_9270890, EPI_ISL_9270891, EPI_ISL_9270897, EPI_ISL_9270907, EPI_ISL_9270913, EPI_ISL_9270917, EPI_ISL_9270921, EPI_ISL_9270923, EPI_ISL_9270931, EPI_ISL_9270938, EPI_ISL_9270956, EPI_ISL_9270959, EPI_ISL_9270977, EPI_ISL_9270984, EPI_ISL_9270988, EPI_ISL_9270993, EPI_ISL_9271001, EPI_ISL_9271002, EPI_ISL_9271004, EPI_ISL_9271006, EPI_ISL_9271010, EPI_ISL_9271013, EPI_ISL_9271024, EPI_ISL_9271028, EPI_ISL_9271031, EPI_ISL_9271036, EPI_ISL_9271038, EPI_ISL_9271044, EPI_ISL_9271051, EPI_ISL_9271052, EPI_ISL_9271054, EPI_ISL_9271057, EPI_ISL_9271058, EPI_ISL_9271083, EPI_ISL_9271088, EPI_ISL_9271089, EPI_ISL_9271096, EPI_ISL_9271101, EPI_ISL_9271102, EPI_ISL_9271113, EPI_ISL_9271115, EPI_ISL_9271122, EPI_ISL_9271131, EPI_ISL_9271132, EPI_ISL_9271135, EPI_ISL_9271143, EPI_ISL_9271174, EPI_ISL_9271177, EPI_ISL_9271179, EPI_ISL_9271185, EPI_ISL_9271190, EPI_ISL_9271198, EPI_ISL_9271200, EPI_ISL_9271210, EPI_ISL_9271224, EPI_ISL_9271228, EPI_ISL_9271231, EPI_ISL_9271235, EPI_ISL_9271254, EPI_ISL_9271258, EPI_ISL_9271263, EPI_ISL_9271268, EPI_ISL_9271269, EPI_ISL_9271270, EPI_ISL_9271277, EPI_ISL_9271297, EPI_ISL_9271302, EPI_ISL_9271303, EPI_ISL_9271305, EPI_ISL_9271315, EPI_ISL_9271318, EPI_ISL_9271320, EPI_ISL_9271324, EPI_ISL_9271328, EPI_ISL_9271344, EPI_ISL_9271352, EPI_ISL_9271354, EPI_ISL_9271368, EPI_ISL_9271376, EPI_ISL_9271398, EPI_ISL_9271400, EPI_ISL_9271401, EPI_ISL_9271404, EPI_ISL_9271408, EPI_ISL_9271412, EPI_ISL_9271414, EPI_ISL_9271417, EPI_ISL_9271418, EPI_ISL_9271422, EPI_ISL_9271426, EPI_ISL_9271427, EPI_ISL_9271439, EPI_ISL_9271446, EPI_ISL_9271460, EPI_ISL_9271473, EPI_ISL_9271474, EPI_ISL_9271478, EPI_ISL_9271495, EPI_ISL_9271499, EPI_ISL_9271510, EPI_ISL_9271513, EPI_ISL_9271517, EPI_ISL_9271531, EPI_ISL_9271535, EPI_ISL_9271537, EPI_ISL_9271553, EPI_ISL_9271565, EPI_ISL_9271574, EPI_ISL_9271580, EPI_ISL_9271602, EPI_ISL_9271608, EPI_ISL_9271613, EPI_ISL_9271619, EPI_ISL_9271623, EPI_ISL_9271625			
see above	Department of Bacteria, Parasites and Fungi, Statens Serum Institut, Copenhagen, Denmark	Statens Serum Institut Bioinformatics and Microbial Genomics	Danish Covid-19 Genome Consortium

We gratefully acknowledge the following Authors from the Originating laboratories responsible for obtaining the specimens, as well as the Submitting laboratories where the genome data were generated and shared via GISAID, on which this research is based.

All Submitters of data may be contacted directly via www.gisaid.org

Authors are sorted alphabetically.

Accession ID	Originating Laboratory	Submitting Laboratory	Authors
EPI_ISL_9267862	Respiratory Virus Unit, Microbiology Services Colindale, Public Health England	COVID-19 Genomics UK (COG-UK) Consortium	PHE Covid Sequencing Team

We gratefully acknowledge the following Authors from the Originating laboratories responsible for obtaining the specimens, as well as the Submitting laboratories where the genome data were generated and shared via GISAID, on which this research is based.

All Submitters of data may be contacted directly via www.gisaid.org

Authors are sorted alphabetically.

Accession ID	Originating Laboratory	Submitting Laboratory	Authors
EPI_ISL_9264723, EPI_ISL_9264727	CDPH VBL	California Department of Public Health	CDPH COVIDNet
EPI_ISL_9264440, EPI_ISL_9264441, EPI_ISL_9264442, EPI_ISL_9264447, EPI_ISL_9264448, EPI_ISL_9264449, EPI_ISL_9264451			
see above	Pathology, UAB	Pathology, UAB	E.J.; Hendrickson; Leal; Lefkowitz; Moates, D.; R.C.; S.M.; Van Der Pol; W.J.
EPI_ISL_9263791	State Laboratories Division, Hawaii State Department of Health	State Laboratories Division, Hawaii State Department of Health	Ayana Garnet; Briana Ofilas; Cheryl-lynn Daquip; Cheyenne Barela; Daniel Strange; Drew Kuwazaki; Edward Desmond; Jeffrey Au; Mark Nagata; Pamela O'Brien; Razvan Sultana; Remedios Gose; Samantha Cotter; Samantha Sruba

We gratefully acknowledge the following Authors from the Originating laboratories responsible for obtaining the specimens, as well as the Submitting laboratories where the genome data were generated and shared via GISAID, on which this research is based.

All Submitters of data may be contacted directly via www.gisaid.org

Authors are sorted alphabetically.

Accession ID	Originating Laboratory	Submitting Laboratory	Authors
EPI_ISL_9274900	Hospital da Baleia	Instituto Rene Rachou / Fiocruz Minas	Alana Oliveira; Anna Salim; Camila Corsini; Daniel Miranda; Gabriel Fernandes; Mozar de Castro; Nathalie Almeida; Pedro Alves; Priscilla Filgueiras; Rafaella Fortini; Raphael Silva; Raquel Vilela; Rubens do Monte Neto; Sarah Gomes; Thais Silva; Wander Jeremias
EPI_ISL_9274974, EPI_ISL_9274978, EPI_ISL_9274982, EPI_ISL_9274983, EPI_ISL_9275005, EPI_ISL_9275023, EPI_ISL_9275024, EPI_ISL_9275031, EPI_ISL_9275047, EPI_ISL_9275059, EPI_ISL_9275061			
see above	Laboratorio Municipal de Biologia Molecular	Instituto Rene Rachou / Fiocruz Minas	Andre Menezes; Anna Salim; Eneida Oliveira; Gabriel Fernandes; Pedro Alves; Rubens do Monte Neto; Thais Silva
